# Drug-specific safety signal prioritization of antibody-drug conjugates in breast cancer: integrating FAERS pharmacovigilance, machine learning, and clinical contextualization

**DOI:** 10.3389/fphar.2026.1941296

**Published:** 2026-09-09

**Authors:** Yuhan Tang, Shaochun Liu, Yingze Zhu, Linlin Fan, Xiaoxi Han, Wenjie Ma, Haolu Zhang, Zhiqi Sui, Hui Pang, Wenhui Zhao

**Affiliations:** 1 Department of Oncology, Harbin Medical University Cancer Hospital, Harbin, China; 2 Department of General Surgery, West China Ya’an Hospital, Sichuan University, Ya’an, China

**Keywords:** antibody-drug conjugate, breast cancer, disproportionality, FAERS, machine learning, pharmacovigilance, signal prioritization

## Abstract

**Background:**

Antibody–drug conjugates (ADCs) are increasingly used across breast cancer subtypes, but their postmarketing safety-reporting patterns differ across agents and are difficult to interpret across spontaneous-reporting and clinical settings. We integrated breast-cancer-restricted FDA Adverse Event Reporting System (FAERS) analyses, temporal machine-learning prioritization, and an independent institutional cohort to characterize drug-specific safety patterns across complementary evidence layers.

**Methods:**

FAERS quarterly files from 2013Q1 through 2025Q4 were deduplicated and analyzed at the report level. The primary dataset comprised 13,529 breast-cancer primary-suspect reports involving trastuzumab emtansine (T-DM1), trastuzumab deruxtecan (T-DXd), or sacituzumab govitecan; 20,526 all-indication reports were retained for supportive sensitivity analysis. Analyses included clinically reviewed preferred-term disproportionality, 11 prespecified adverse events of special interest (AESIs), intraclass adjusted reporting odds, reported time to onset, and temporally validated exploratory machine-learning models for report-level AESI prioritization. An independent retrospective cohort of 105 patients was analyzed descriptively for clinical contextualization.

**Results:**

The primary dataset included 3,962 T-DM1, 6,452 T-DXd, and 3,115 sacituzumab govitecan reports. Compared with T-DM1, T-DXd showed higher adjusted reporting odds for interstitial lung disease (ILD)/pneumonitis (adjusted reporting odds ratio [aROR], 3.23; 95% CI, 2.55–4.09) and gastrointestinal toxicity (aROR, 2.45; 95% CI, 2.07–2.90), whereas sacituzumab govitecan showed higher reporting odds for hematologic (aROR, 1.95; 95% CI, 1.67–2.28) and gastrointestinal toxicity (aROR, 2.91; 95% CI, 2.44–3.46). T-DM1 showed higher hepatobiliary reporting odds than both comparators. Temporal-validation AUROC values ranged from 0.593 to 0.772. In the institutional cohort, hepatobiliary toxicity was most frequent with T-DM1 (32.3%), ILD/pneumonitis with T-DXd (12.0%), and hematologic toxicity with sacituzumab govitecan (66.7%).

**Conclusion:**

The three ADCs showed distinct breast-cancer FAERS safety-reporting patterns. Machine learning provided exploratory report-level prioritization, while institutional observations provided clinical context. These findings support pharmacovigilance signal prioritization but should not be interpreted as incidence, comparative clinical risk, causal effects, or patient-level toxicity prediction.

## 1 Introduction

Antibody–drug conjugates (ADCs) have become an important component of systemic therapy for breast cancer by combining target-directed antibodies with potent cytotoxic payloads. Their clinical applications now span multiple biomarker-defined populations. Trastuzumab emtansine (T-DM1) established the role of ADC therapy in human epidermal growth factor receptor 2 (HER2)-positive advanced and residual early breast cancer, whereas trastuzumab deruxtecan (T-DXd) expanded ADC treatment across HER2-positive, HER2-low, and HER2-ultralow disease (Verma et al., 2012; von et al., 2019; Krop et al., 2014; Modi et al., 2020; Cortés et al., 2022; Modi et al., 2022). Sacituzumab govitecan further broadened ADC application through trophoblast cell-surface antigen 2 (Trop-2) targeting in previously treated triple-negative and hormone receptor-positive/HER2-negative metastatic breast cancer (Bardia et al., 2021; Rugo et al., 2023), while datopotamab deruxtecan (Dato-DXd) represents an emerging Trop-2-directed strategy in hormone receptor-positive/HER2-negative disease (Bardia et al., 2025; Bardia et al., 2024a). As ADC utilization expands across increasingly diverse clinical settings, systematic characterization of their evolving postmarketing safety-reporting patterns has become increasingly important.

Existing clinical evidence indicates that ADCs exhibit distinct toxicity patterns related to differences in antibody targets, linker chemistry, payload characteristics, drug-to-antibody ratio, pharmacokinetics, and treated populations. T-DM1 is associated with thrombocytopenia and hepatobiliary toxicity, whereas T-DXd requires particular attention to interstitial lung disease (ILD)/pneumonitis and gastrointestinal toxicity. Sacituzumab govitecan is characterized by hematologic toxicity and diarrhea, while emerging Trop-2-directed ADCs introduce additional safety considerations, including oral and ocular events (Bardia et al., 2024b; Powell et al., 2022; Swain et al., 2022; Park et al., 2025; Tolaney et al., 2025; Rugo et al., 2025; Xu et al., 2025; Dong et al., 2025; Food and Administration, 2026a; Food and Administration, 2026b; Food and Administration, 2025a; Food and Administration, 2025b). Although randomized trials and prescribing information provide essential denominator-based safety evidence, they may not fully characterize uncommon preferred terms, geographic variation, temporal reporting patterns, or safety signals emerging after broader clinical adoption.

Spontaneous-reporting systems provide a complementary means of examining postmarketing safety-reporting patterns at scale. However, FDA Adverse Event Reporting System (FAERS) analyses require careful interpretation because spontaneous reports lack exposure denominators and are affected by underreporting, stimulated reporting, incomplete clinical information, follow-up submissions, and comparator selection (Hazell and Shakir, 2006; García-Abeijon et al., 2023; Kant et al., 2022; van Puijenbroek et al., 2002; Cutroneo et al., 2023; Gravel et al., 2024; Bate et al., 1998; Food and Administration, 2026c). Previous pharmacovigilance studies have evaluated individual ADCs or specific ADC classes (Zhu et al., 2026; Yang et al., 2026; Jeral et al., 2026; Hosonaka et al., 2026), but broad all-indication analyses may introduce heterogeneity arising from different disease contexts and treatment settings. Moreover, conventional disproportionality analyses identify reporting signals but do not inherently incorporate temporal validation or report-level prioritization. Machine-learning approaches may complement conventional pharmacovigilance by prioritizing submitted reports for expert review, provided that their outputs are interpreted as report-level prioritization rather than patient-level risk prediction (Al-Azzawi et al., 2023; Battini et al., 2024; Gosselt et al., 2022; Dauner et al., 2023; Collins et al., 2024; Moons et al., 2025; Christodoulou et al., 2019). Integrating large-scale pharmacovigilance with independently characterized institutional data may therefore provide a complementary framework for assessing the clinical context of ADC safety signals.

**FIGURE 1:**
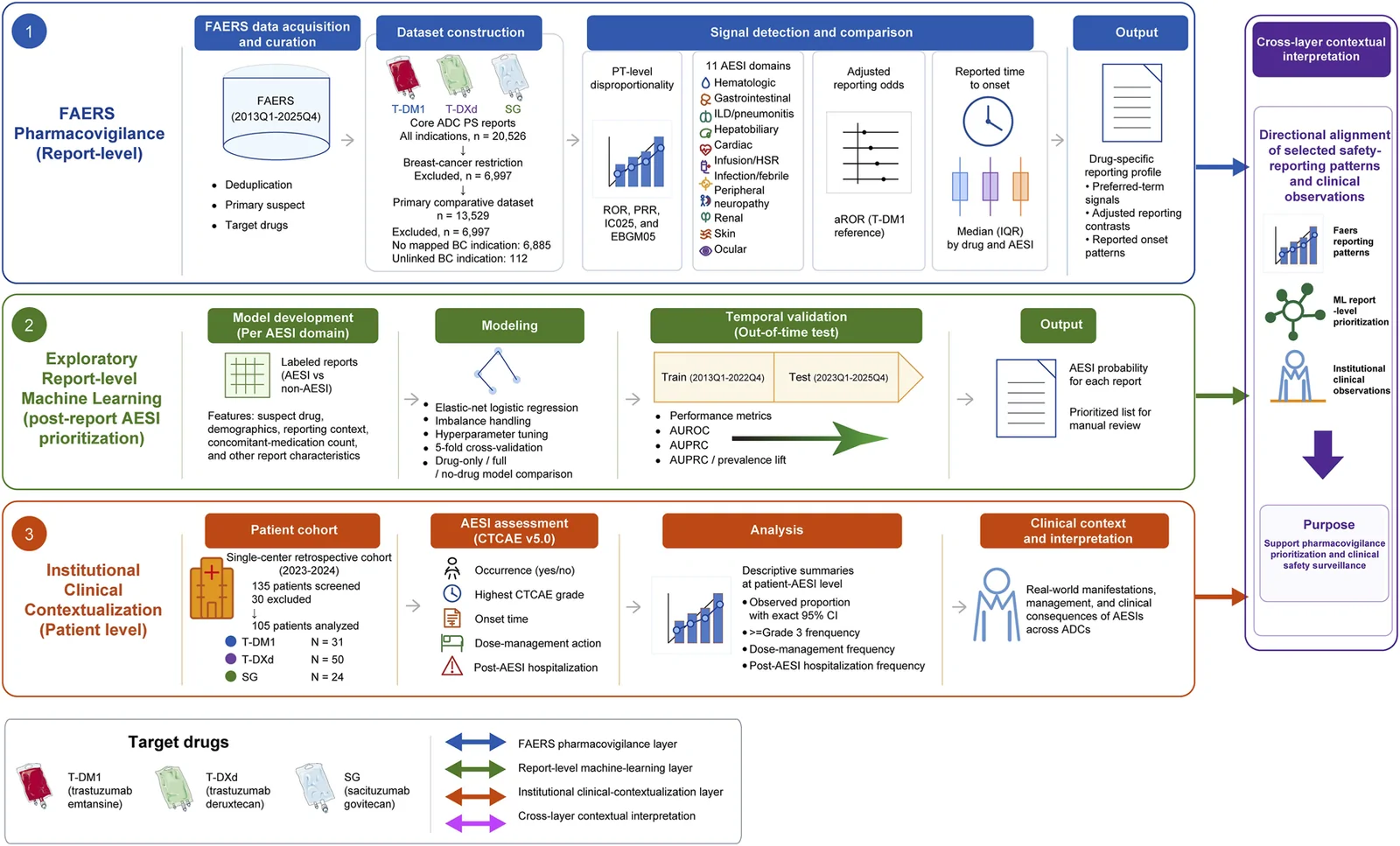
Study flow and prespecified three-layer analytical framework. FAERS quarterly files from 2013 Q1 through 2025 Q4 were imported and deduplicated before candidate ADC primary-suspect reports were identified. The primary pharmacovigilance analysis was conducted in the breast-cancer-restricted core ADC dataset comprising T-DM1, T-DXd, and sacituzumab govitecan (N = 13,529), whereas the corresponding all-indication core dataset (N = 20,526) was retained for supportive sensitivity analysis. Reports excluded by the breast-cancer restriction were classified according to indication mapping, including reports with no mapped breast-cancer indication and reports in which a breast-cancer indication was present but not directly linked to the target ADC drug sequence. The second analytical layer used temporally validated machine-learning models for report-level AESI signal prioritization within the breast-cancer core dataset. The third layer comprised an independent institutional cohort (N = 105) used solely for descriptive clinical contextualization of AESI patterns, severity, treatment management, and temporal clinical consequences. Dato-DXd and SKB264 were retained as cold-start and sparse exploratory cohorts, respectively, whereas RC48 had no captured reports. The three analytical layers were evaluated separately and were not pooled because they represent different analytical units, denominators, and data-generating mechanisms. ADC, antibody–drug conjugate; AESI, adverse event of special interest; Dato-DXd, datopotamab deruxtecan; FAERS, FDA Adverse Event Reporting System; ML, machine learning; PS, primary suspect; SG, sacituzumab govitecan; T-DM1, trastuzumab emtansine; T-DXd, trastuzumab deruxtecan.

Here, we performed a multi-layered analysis integrating breast-cancer-restricted FAERS pharmacovigilance, temporal machine-learning prioritization, and independent institutional clinical contextualization. We first characterized Preferred Term (PT) and adverse event of special interest (AESI) reporting patterns for T-DM1, T-DXd, and sacituzumab govitecan using clinically reviewed signal detection and intraclass adjusted reporting analyses, while separately describing emerging ADCs with limited postmarketing capture. We then developed temporally validated report-level machine-learning models to prioritize AESI-containing FAERS reports for pharmacovigilance review. Finally, we characterized treatment-emergent AESIs, source-recorded severity, dose-management actions, and temporal clinical consequences in an independent cohort of 105 ADC-treated patients with breast cancer. Together, this framework was designed to characterize drug-specific ADC safety-reporting patterns across complementary evidence layers and to support postmarketing signal prioritization, clinical contextualization, and toxicity-focused monitoring (Figure 1).

## 2 Methods

### 2.1 Study design and data sources

This study integrated three complementary analytical components: FDA Adverse Event Reporting System (FAERS)-based pharmacovigilance signal detection, exploratory FAERS-based machine-learning signal prioritization, and a single-center retrospective institutional clinical contextualization cohort. FAERS was analyzed as a report-level spontaneous reporting system, in which disproportionality analyses were interpreted as signal-prioritization measures rather than estimates of treatment incidence or causal risk. The institutional cohort was analyzed as a patient-level denominator-based dataset and was used exclusively to contextualize clinically documented adverse event of special interest (AESI) patterns, severity, treatment management, and temporal consequences observed in routine practice. The overall analytical framework was designed to evaluate whether drug-specific safety-reporting patterns identified in FAERS showed directional clinical contextualization in an independent institutional cohort. The three evidence layers were analyzed separately because FAERS reports and institutional patients represent different analytical units, denominators, and data-generating mechanisms. Detailed data-processing procedures, AESI dictionaries, model specifications, and institutional variable definitions are provided in the Supplementary Methods.

### 2.2 FAERS data source and antibody–drug conjugate (ADC) eligibility

Quarterly FAERS public data files from 2013Q1 through 2025Q4 were downloaded from the FDA website. Individual case safety reports were constructed using FDA case identifiers and version information. Duplicate and follow-up records were handled according to FDA case-version principles, and each final report was treated as the unit of analysis. Six ADC agents were screened for postmarketing safety evaluation: trastuzumab emtansine (T-DM1), trastuzumab deruxtecan (T-DXd), sacituzumab govitecan (SG), datopotamab deruxtecan (Dato-DXd), SKB264, and disitamab vedotin (RC48). Drug names, generic names, brand names, and relevant synonyms were standardized before report construction.

Primary comparative analyses were prespecified for T-DM1, T-DXd, and SG because these agents had sufficient breast-cancer FAERS capture and represented clinically established ADC treatment populations. Dato-DXd and SKB264 were retained as exploratory cohorts because of limited postmarketing capture, whereas RC48 was retained for capture assessment only. Agent eligibility and prespecified analytic roles are summarized in Table 1, and the complete dataset derivation is shown in Figure 1.

**TABLE 1:** Drug eligibility and analytic roles.

ADC	Target	Payload or class	All-indication PS reports, n	Breast-cancer PS reports, n	Analytic role	Included in primary comparative models
T-DM1	HER2	DM1, a maytansinoid microtubule inhibitor	5,261	3,962	Core comparative agent	Yes
T-DXd	HER2	Deruxtecan, a topoisomerase I inhibitor	10,290	6,452	Core comparative agent	Yes
Sacituzumab govitecan	Trop-2	SN-38, a topoisomerase I inhibitor	4,975	3,115	Core comparative agent	Yes
Dato-DXd	Trop-2	Deruxtecan, a topoisomerase I inhibitor	299	148	Early postmarketing descriptive cohort	No
SKB264	Trop-2	Topoisomerase I inhibitor payload	19	8	Sparse exploratory cohort	No
Disitamab vedotin (RC48/RC48-ADC)	HER2	MMAE, a microtubule inhibitor	0	0	No FAERS capture	No

Candidate ADC, names and synonyms, targets, payload classes, all-indication and breast-cancer primary-suspect report counts, and prespecified analytic roles. T-DM1, T-DXd, and sacituzumab govitecan formed the core comparison; other candidates did not enter the primary comparative models. ADC, antibody–drug conjugate; HER2, human epidermal growth factor receptor 2; PS, primary suspect; T-DM1, trastuzumab emtansine; T-DXd, trastuzumab deruxtecan; Dato-DXd, datopotamab deruxtecan; Trop-2, trophoblast cell-surface antigen 2.

### 2.3 Breast-cancer restriction and report derivation

Reports were classified according to recorded indication information using prespecified breast-cancer indication terms. The primary comparative dataset was restricted to breast-cancer reports involving T-DM1, T-DXd, or SG as primary-suspect drugs, whereas the corresponding all-indication dataset was retained for supportive sensitivity analyses. To ensure indication specificity, a mapped breast-cancer indication was required to be directly linked to the target ADC through the corresponding FAERS drug sequence. Accordingly, reports containing a mapped breast-cancer indication elsewhere in the same report but not directly linked to the target ADC drug sequence were excluded from the breast-cancer–restricted dataset.

Among 20,526 all-indication primary-suspect reports involving the three core ADCs, 13,529 met the prespecified breast-cancer restriction and constituted the primary comparative dataset. Of the 6,997 reports excluded by this restriction, 6,885 contained no mapped breast-cancer indication anywhere in the report, whereas 112 contained a mapped breast-cancer indication elsewhere in the report but not one directly linked to the target ADC drug sequence. These excluded reports remained eligible, where applicable, for the supportive all-indication analyses. The complete report derivation process and dataset composition are shown in Figure 1.

### 2.4 AESI definitions and preferred-term curation

Eleven AESI domains were prespecified and mapped using predefined Medical Dictionary for Regulatory Activities (MedDRA) Preferred Term (PT) dictionaries. PT-level signal screening was conducted within the breast-cancer primary dataset. Statistical eligibility required at least 10 reports, a lower 95% confidence bound for the reporting odds ratio (ROR025) >1, and either a lower 5% empirical Bayes bound (EB05) >2 or a lower information-component bound (IC025) >0. Statistically eligible PTs underwent independent clinical review by Yuhan Tang and Shaochun Liu, followed by cross-checking by Yingze Zhu. Terms primarily reflecting disease progression or metastatic involvement, treatment administration or product use, radiation-related effects, or nonspecific clinical deterioration were excluded from the clinically interpretable main panel. Disagreements or ambiguous classifications were resolved by joint review and, when necessary, final adjudication by Wenhui Zhao. Detailed PT-level eligibility, clinical categories, exclusion rationales, and final dispositions are provided in Supplementary Table S2A.

### 2.5 Pharmacovigilance signal detection and adjusted reporting analyses

Disproportionality analyses were performed using established spontaneous-reporting measures, including reporting odds ratio (ROR), proportional reporting ratio (PRR), information component (IC), and empirical Bayes geometric mean (EBGM), where applicable. These measures were interpreted as relative reporting disproportionality within the FAERS database and not as incidence estimates. For prespecified AESI domains, intraclass adjusted reporting-odds models were fitted within breast-cancer ADC reports using T-DM1 as the reference comparator. Models were adjusted for available report-level covariates, including age group, sex, report year, reporter region, reporter type, and concomitant medication burden when model convergence was achieved. Reported time-to-onset (TTO) was calculated for reports containing plausible treatment initiation and event dates. TTO summaries were restricted to valid observations and interpreted as reported temporal patterns rather than biological latency estimates. The domain-specific adjusted reporting analyses were prespecified and exploratory; corresponding P values were therefore reported as nominal without multiplicity adjustment and were interpreted together with effect estimates and 95% confidence intervals rather than as confirmatory hypothesis tests.

### 2.6 Exploratory machine-learning signal prioritization

Exploratory report-level classifiers were developed separately for each AESI using a strict temporal split. Reports from 2013 through 2022 were used for model development, and reports from 2023 through 2025 were reserved for validation. To assess the contribution of drug identity, three penalized logistic-regression models were compared: a drug-only baseline (M0), a full contextual model including drug identity (M1), and an otherwise identical contextual model without drug identity (M2). Contextual predictors comprised age group, sex, geographic region, reporter type, report year, concomitant-medication count, and TTO availability and duration. Categorical predictors were imputed using the most frequent category and one-hot encoded, whereas numeric predictors were standardized after imputation where applicable. Missing TTO duration was set to zero and represented jointly with a separate TTO-availability indicator. Hyperparameters were selected by five-fold stratified cross-validation restricted entirely to the development period, and class imbalance was addressed using balanced class weights. Full preprocessing steps, hyperparameter grids, and performance definitions are provided in the Supplementary Table S4.

### 2.7 Institutional cohort and clinical contextualization

Institutional cohort and patient selection. We retrospectively screened 135 patients with breast cancer who were prescribed trastuzumab emtansine (T-DM1), trastuzumab deruxtecan (T-DXd), or sacituzumab govitecan (SG) at Harbin Medical University Cancer Hospital between January 18 and 8 July 2023. Eligibility required receipt of ADC treatment, available baseline clinical information, and at least one evaluable post-treatment safety assessment. After source-record review, 30 patients were excluded: 12 did not ultimately receive an ADC infusion, 10 participated in concurrent interventional clinical trials, and eight lacked any evaluable post-treatment safety assessment. The final analytical cohort comprised 105 patients (T-DM1, n = 31; T-DXd, n = 50; SG, n = 24). Baseline characteristics of the institutional cohort are summarized in Supplementary Table S1. The cohort was used solely for descriptive clinical contextualization and was not incorporated into FAERS analyses, machine-learning development, calibration, threshold selection, or external validation.

Clinical data abstraction and quality control. Clinical characteristics, treatment exposure, adverse events, laboratory findings, and safety outcomes were independently extracted from the hospital information system and electronic medical records by Yuhan Tang and Shaochun Liu. Yingze Zhu cross-checked the dataset and performed source-record verification in a randomly selected 20% sample, with particular attention to AESI ascertainment and Common Terminology Criteria for Adverse Events (CTCAE) v5.0 grading. Discrepancies were resolved by joint source-record review and, when necessary, final adjudication by Wenhui Zhao.

AESI definitions and clinical outcomes. Clinically meaningful treatment-emergent AESIs were defined as new events or documented worsening after ADC initiation supported by laboratory, imaging, or clinical documentation. Each patient was counted once within each AESI domain; when multiple events occurred, the highest CTCAE grade and earliest valid onset date were retained. Hospitalization and death after AESI onset were treated as temporal clinical-consequence markers rather than evidence of causal attribution. Drug-specific and overall institutional AESI summaries are provided in Supplementary Tables S5, S6.

Follow-up and vital-status ascertainment. Follow-up extended from index ADC initiation to the earliest of the last available clinical record, death, or 27 August 2024. Vital status was determined from electronic medical records and routine hospital follow-up by telephone or WeChat approximately every 2–3 months. Patients lost to follow-up remained in the analytical cohort if they had contributed at least one evaluable post-treatment safety assessment; all observations before loss to follow-up were retained. Cause of death was determined by case-by-case review of available records.

### 2.8 Institutional statistical analysis

Institutional analyses were conducted at the patient level. Continuous variables were summarized as medians and interquartile ranges and categorical variables as counts and percentages. AESI frequencies used fixed treatment-group denominators of 31, 50, and 24 for T-DM1, T-DXd, and sacituzumab govitecan, respectively, with exact Clopper–Pearson 95% confidence intervals. Overall group differences were evaluated using Fisher–Freeman–Halton exact tests, and prespecified pairwise comparisons for four AESI domains used Fisher exact tests with Holm correction across 12 comparisons. All analyses were exploratory and unadjusted for baseline covariates because the treatment groups were indication-defined and clinically heterogeneous. Detailed exact-test results are provided in Supplementary Table S7.

### 2.9 Cross-layer contextualization

Cross-layer contextualization was descriptive. FAERS reporting patterns were considered alongside institutional AESI frequencies and clinical consequences, but the two data sources were not pooled because they differed in analytical unit, denominator, ascertainment, and data-generating mechanism. Similarities across layers were interpreted qualitatively and not as external validation, causal confirmation, or comparative incidence. Domains with sparse institutional events were considered insufficient for stable interpretation. Machine-learning outputs remained an independent report-level prioritization layer. Cross-layer summaries are provided in Supplementary Tables S8A, B.

## 3 Results

### 3.1 Study derivation and analytic datasets

Figure 1 summarizes FAERS report derivation and the prespecified separation between the primary breast-cancer-restricted analysis and the supportive all-indication analysis. Across 52 quarterly FAERS releases from 2013Q1 through 2025Q4, 19,478,153 DEMO rows were imported. After FDA-style case-version deduplication, 16,779,363 unique reports remained, of which 16,777,558 contained at least one primary-suspect drug. Screening for the six candidate ADCs identified 20,844 primary-suspect reports, including 13,685 with a mapped breast-cancer indication. Eligibility for the breast-cancer-restricted analytic dataset additionally required the mapped breast-cancer indication to be directly linked to the target ADC drug sequence, as prespecified in the Section 2.

The primary comparative dataset comprised 13,529 breast-cancer reports involving T-DM1 (n = 3,962), T-DXd (n = 6,452), or sacituzumab govitecan (n = 3,115). The corresponding all-indication core dataset contained 20,526 reports and was retained only for supportive sensitivity analysis. Of the 6,997 core-ADC reports excluded by the breast-cancer restriction, 6,885 contained no mapped breast-cancer indication anywhere in the report, whereas 112 contained a mapped breast-cancer indication elsewhere in the report but not one directly linked to the target ADC drug sequence. Dato-DXd was designated as an early postmarketing descriptive cohort, with 299 all-indication reports and 148 breast-cancer reports. SKB264 was retained as a sparse exploratory cohort, with 19 and 8 reports, respectively. No report involving disitamab vedotin, RC48, or RC48-ADC was captured. Dato-DXd, SKB264, and RC48 were therefore excluded from the primary comparative adjusted and machine-learning analyses (Figure 1; Table 1).

The institutional cohort was assembled independently of the FAERS datasets and the machine-learning pipeline. It comprised 105 patients treated with T-DM1 (n = 31), T-DXd (n = 50), or sacituzumab govitecan (n = 24). The cohort predominantly consisted of patients with metastatic disease (97.1%), with a median age of 52 years (IQR, 47–57) and a median of three prior treatment lines (Supplementary Table S1). The institutional cohort was intended solely to provide descriptive clinical context for the FAERS observations and was not designed or used to validate the FAERS analyses or machine-learning models. The institutional data were not merged with FAERS reports or used for model development, temporal validation, calibration, threshold selection, or external performance evaluation (Supplementary Table S1).

### 3.2 Report characteristics, temporal trends, and reporter geography

**FIGURE 2:**
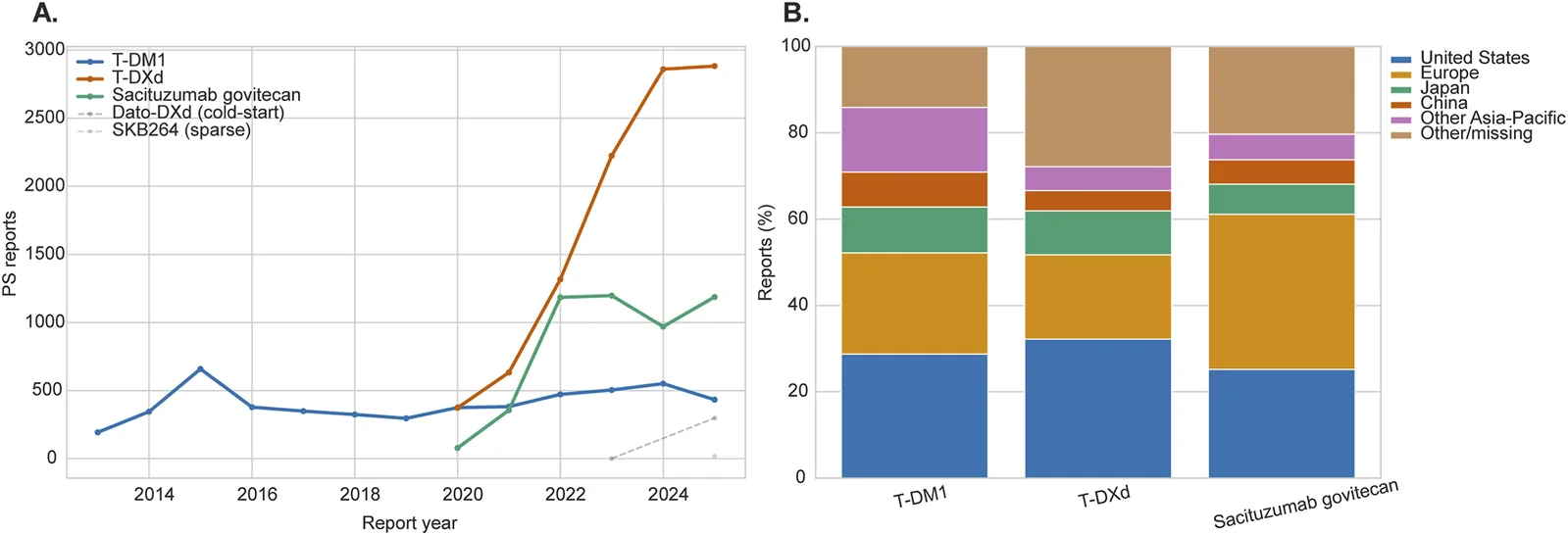
Temporal reporting trends and reporter-region composition of candidate ADCs. **(A)** Annual all-indication primary-suspect report counts in the candidate-ADC dataset (N = 20,844) from 2013Q1 through 2025Q4. Dato-DXd and SKB264 are shown with reduced visual weight because they were retained as cold-start and sparse exploratory cohorts, respectively. **(B)** Reporter-region composition within the breast-cancer core comparative dataset (N = 13,529) for T-DM1, T-DXd, and sacituzumab govitecan. Percentages represent the distribution of submitted reports within each drug-specific report set and should not be interpreted as treated-population distributions. ADC, antibody–drug conjugate; Dato-DXd, datopotamab deruxtecan; PS, primary suspect; SG, sacituzumab govitecan; T-DM1, trastuzumab emtansine; T-DXd, trastuzumab deruxtecan.

The three core ADCs showed distinct temporal reporting patterns and reporter-region distributions. Across the all-indication candidate-ADC dataset, T-DM1 reports were represented throughout the study period, whereas T-DXd and sacituzumab govitecan appeared from 2020 onward and increased substantially thereafter. From 2023 onward, T-DXd contributed the largest annual number of primary-suspect reports among the evaluated ADCs (Figure 2A). Within the breast-cancer core comparative dataset, women accounted for 84.9% of T-DM1, 91.2% of T-DXd, and 95.1% of sacituzumab govitecan reports. Age information was unavailable in 33.6%, 54.1%, and 37.9% of reports, respectively. The United States contributed the largest proportion of reports for T-DM1 (28.7%) and T-DXd (32.2%), whereas Europe represented the largest reporting region for sacituzumab govitecan (35.9%) (Figure 2B; Table 2). Physicians were the most frequently identified reporter category for all three agents, followed by consumers or other healthcare professionals (Table 2).

**TABLE 2:** Characteristics and adverse-event reporting patterns in the breast-cancer core comparative FAERS dataset.

Characteristic	T-DM1 (N = 3,962)	T-DXd (N = 6,452)	Sacituzumab govitecan (N = 3,115)
Age group, n (%)			
<50 years	769 (19.4)	729 (11.3)	616 (19.8)
50–64 years	1,212 (30.6)	1,236 (19.2)	809 (26.0)
65–74 years	490 (12.4)	660 (10.2)	357 (11.5)
≥75 years	161 (4.1)	339 (5.3)	151 (4.8)
Missing	1,330 (33.6)	3,488 (54.1)	1,182 (37.9)
Sex, n (%)			
Female	3,365 (84.9)	5,882 (91.2)	2,961 (95.1)
Male	41 (1.0)	75 (1.2)	12 (0.4)
Missing or unknown	556 (14.0)	495 (7.7)	142 (4.6)
Reporter region, n (%)			
United States	1,139 (28.7)	2,078 (32.2)	785 (25.2)
Europe	930 (23.5)	1,260 (19.5)	1,118 (35.9)
Japan	418 (10.6)	655 (10.2)	217 (7.0)
China	321 (8.1)	304 (4.7)	177 (5.7)
Other Asia-Pacific	595 (15.0)	358 (5.5)	184 (5.9)
Other or missing	559 (14.1)	1,797 (27.9)	634 (20.4)
Reporter type, n (%)			
Physician	2,137 (53.9)	3,341 (51.8)	1,531 (49.1)
Consumer	868 (21.9)	1,365 (21.2)	468 (15.0)
Other health professional	431 (10.9)	1,302 (20.2)	781 (25.1)
Pharmacist	303 (7.6)	439 (6.8)	333 (10.7)
Other, lawyer, or missing	223 (5.6)	5 (0.1)	2 (0.1)
Serious outcome markers, n (%)			
Any serious outcome marker	1,792 (45.2)	2,995 (46.4)	1,415 (45.4)
Hospitalization	1,261 (31.8)	1,743 (27.0)	785 (25.2)
Death	593 (15.0)	1,557 (24.1)	701 (22.5)
Life-threatening event	135 (3.4)	231 (3.6)	110 (3.5)
Reported time to onset			
TTO available, n (%)	1,479 (37.3)	1,691 (26.2)	1,125 (36.1)
TTO, median (IQR), days	49 (7–189)	31 (7–120)	14 (7–55)
AESI reporting patterns, n (%)			
ILD/pneumonitis	139 (3.5)	863 (13.4)	55 (1.8)
Hematologic toxicity	585 (14.8)	588 (9.1)	792 (25.4)
Gastrointestinal toxicity	376 (9.5)	1,209 (18.7)	678 (21.8)
Hepatobiliary toxicity	281 (7.1)	157 (2.4)	77 (2.5)
Cardiac toxicity	112 (2.8)	133 (2.1)	17 (0.5)
Ocular toxicity	85 (2.1)	84 (1.3)	18 (0.6)
Skin toxicity	136 (3.4)	299 (4.6)	207 (6.6)
Infection/febrile events	311 (7.8)	368 (5.7)	182 (5.8)
Infusion/hypersensitivity	65 (1.6)	29 (0.4)	39 (1.3)
Renal toxicity	43 (1.1)	91 (1.4)	38 (1.2)
Peripheral neuropathy	186 (4.7)	91 (1.4)	42 (1.3)

Values are n (%) unless otherwise indicated, with percentages calculated within each drug-specific report set. Serious-outcome markers and AESI, domains are not mutually exclusive; TTO, summaries include only reports with valid onset information; AESI, proportions represent reporting patterns among submitted FAERS, reports and should not be interpreted as incidence or comparative clinical risk. AESI, adverse event of special interest; FAERS, FDA, adverse event reporting system; T-DM1, trastuzumab emtansine; T-DXd, trastuzumab deruxtecan; TTO, time to onset; IQR, interquartile range.

The overall proportion of reports containing at least one serious outcome marker was comparable across the three ADCs, ranging from 45.2% to 46.4%. However, the composition of serious outcomes differed. Hospitalization was most frequently reported for T-DM1 (31.8%), whereas death was reported more frequently for T-DXd (24.1%) and sacituzumab govitecan (22.5%). Life-threatening events were uncommon and occurred at similar frequencies across the three agents (3.4%–3.6%) (Table 2).

Valid time-to-onset (TTO) information was available for only 37.3% of T-DM1, 26.2% of T-DXd, and 36.1% of sacituzumab govitecan reports. Among reports with available onset dates, the median TTO was 49 days (IQR, 7–189) for T-DM1, 31 days (IQR, 7–120) for T-DXd, and 14 days (IQR, 7–55) for sacituzumab govitecan (Table 2). Across the prespecified adverse events of special interest (AESI), marked differences in reporting patterns were observed. ILD/pneumonitis was reported most frequently with T-DXd (13.4%), hematologic toxicity with sacituzumab govitecan (25.4%), and hepatobiliary toxicity with T-DM1 (7.1%), whereas the remaining AESI domains demonstrated drug-specific reporting profiles that are examined in detail in the following sections (Table 2).

Because FAERS is a spontaneous reporting system, these characteristics describe reporting patterns of submitted safety reports rather than the demographic, geographic, or clinical characteristics of treated patients.

### 3.3 Serious outcome markers and reported time to onset

**FIGURE 3:**
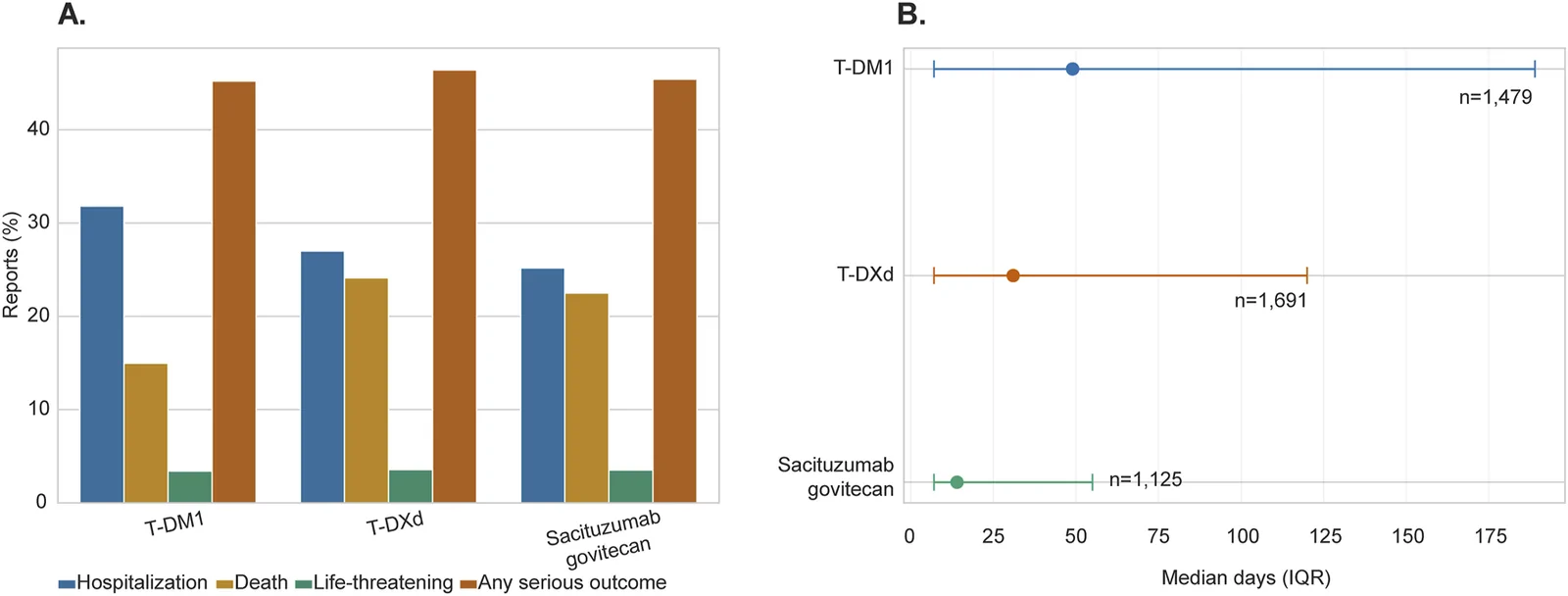
Serious outcome reporting patterns and reported time to onset in the breast-cancer core comparative FAERS dataset (N = 13,529). **(A)** Proportions of reports containing hospitalization, death, life-threatening, or any serious-outcome markers. Outcome markers were not mutually exclusive. **(B)** Reported time to onset among reports with valid treatment-start and event dates. Points indicate medians and horizontal bars indicate interquartile ranges; n denotes the number of reports with evaluable TTO. Serious-outcome markers and TTO summaries describe submitted FAERS reports and should not be interpreted as event-attributable outcome rates or clinical incidence. FAERS, FDA Adverse Event Reporting System; IQR, interquartile range; TTO, time to onset.

Serious outcome markers were common and were not mutually exclusive. Hospitalization was recorded in 31.8% of T-DM1, 27.0% of T-DXd, and 25.2% of sacituzumab govitecan reports. The corresponding death markers were 15.0%, 24.1%, and 22.5%, while life-threatening markers were recorded in 3.4%, 3.6%, and 3.5%, respectively. At least one serious outcome marker was present in 45.2% of T-DM1, 46.4% of T-DXd, and 45.4% of sacituzumab govitecan reports. These values describe fields recorded within submitted FAERS reports rather than event-attributable clinical outcome rates (Figure 3A; Table 2).

Valid reported time to onset was available for 1,479 T-DM1, 1,691 T-DXd, and 1,125 sacituzumab govitecan reports. Median reported time to onset was 49 days (IQR, 7–189) for T-DM1, 31 days (IQR, 7–120) for T-DXd, and 14 days (IQR, 7–55) for sacituzumab govitecan. These estimates were restricted to reports meeting the prespecified TTO validity criteria (Figure 3B; Table 2). Domain-specific TTO analyses further characterized reported onset patterns across the three ADCs (Supplementary Table S3B). Median reported TTO for ILD/pneumonitis was 109 days for T-DM1, 78 days for T-DXd, and 35 days for sacituzumab govitecan; corresponding medians for hematologic toxicity were 25, 21, and 11.5 days, and those for gastrointestinal toxicity were 20.5, 11, and 11 days, respectively. TTO completeness ranged from 26.1% to 46.7% across these drug–AESI combinations; these estimates therefore describe reported temporal patterns among records with valid onset information rather than comparative biological latency.

### 3.4 Clinically reviewed preferred-term panel and AESI reporting patterns

**FIGURE 4:**
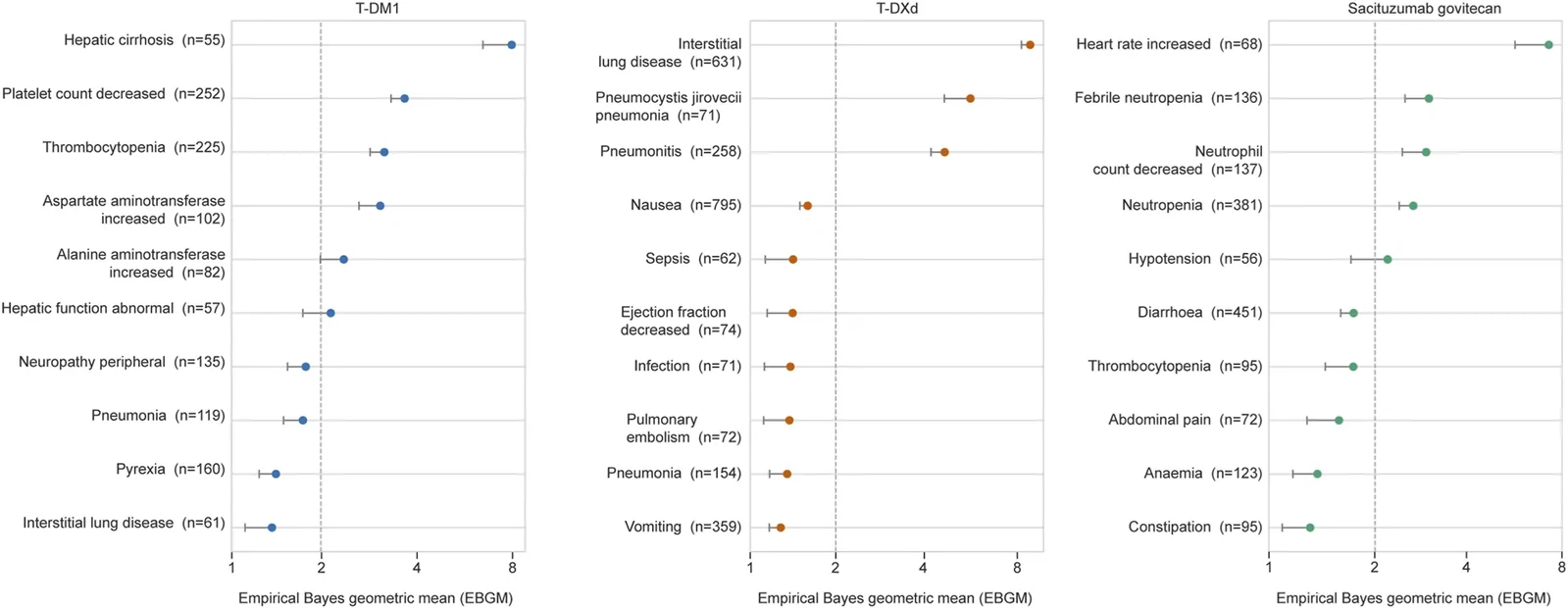
Reporting spectra of the highest-priority clinically interpretable preferred terms across the three core ADCs in the breast-cancer primary dataset (N = 13,529). Preferred terms first underwent prespecified statistical filtering followed by clinical review. Among statistically eligible preferred terms, clinically interpretable signals were retained in the main PT panel, from which the 30 highest-priority preferred terms (10 per core ADC) were selected for graphical display according to the predefined ranking strategy. Complete clinical-review categories, exclusion rationales, retained/excluded status, and final dispositions of statistically eligible preferred terms are provided in Supplementary Table S2A, whereas supportive all-indication, cold-start, sparse, and statistically ineligible terms are presented separately in Supplementary Table S2B. Circles indicate the empirical Bayes geometric mean (EBGM), and horizontal bars indicate the corresponding empirical Bayes interval (EB05–EB95). ADC, antibody–drug conjugate; EB05, lower 5% empirical Bayes bound; EB95, upper 95% empirical Bayes bound; EBGM, empirical Bayes geometric mean; PT, Preferred Term; SG, sacituzumab govitecan; T-DM1, trastuzumab emtansine; T-DXd, trastuzumab deruxtecan.

To improve the clinical interpretability of FAERS signals, preferred terms (PTs) underwent a prespecified multistep review process after statistical signal detection. Terms representing nonspecific symptoms, underlying disease context, drug administration procedures, product-related events, or radiation-related effects were clinically evaluated before construction of the final PT panel. Among 1,794 breast-cancer primary PTs, 236 met the predefined statistical eligibility criteria and entered clinical review. Of these, 138 clinically interpretable PTs were retained in the main signal panel, including the 30 highest-priority PTs displayed in Figure 4, whereas 98 PTs were excluded because they lacked clinical specificity or primarily reflected treatment context rather than drug-related toxicity. The complete review process, clinical-review categories, exclusion rationale, and final disposition of all statistically eligible PTs are provided in Supplementary Table S2A, while supportive all-indication, cold-start, sparse, and statistically ineligible PTs are presented separately in Supplementary Table S2B.

The retained PT panel demonstrated distinct toxicity-reporting spectra across the three core ADCs (Figure 4). T-DM1 was characterized predominantly by platelet-related and hepatobiliary signals, including platelet count decreased (n = 252), thrombocytopenia (n = 225), aspartate aminotransferase increased (n = 102), and hepatic cirrhosis (n = 55). T-DXd demonstrated prominent pulmonary and gastrointestinal PTs, including interstitial lung disease (n = 631), pneumonitis (n = 258), nausea (n = 795), and vomiting (n = 359). Sacituzumab govitecan showed predominant gastrointestinal and hematologic PTs, including diarrhea (n = 451), neutropenia (n = 381), neutrophil count decreased (n = 137), and febrile neutropenia (n = 136).

Mapping of clinically reviewed PTs to the prespecified adverse events of special interest (AESI) demonstrated consistent drug-specific reporting patterns (Table 2). ILD/pneumonitis was reported most frequently for T-DXd (13.4%), compared with T-DM1 (3.5%) and sacituzumab govitecan (1.8%). Hematologic toxicity was most frequently reported for sacituzumab govitecan (25.4%), whereas hepatobiliary toxicity was most prominent for T-DM1 (7.1%). Gastrointestinal toxicity was reported in 21.8% of sacituzumab govitecan and 18.7% of T-DXd reports, substantially exceeding the frequency observed for T-DM1 (9.5%). Sacituzumab govitecan also showed the highest reporting proportion of skin toxicity (6.6%). These analyses describe report-level signal concentrations after standardized statistical filtering and clinical PT review and should not be interpreted as estimates of toxicity incidence among treated patients.

### 3.5 Intraclass adjusted reporting odds

**FIGURE 5:**
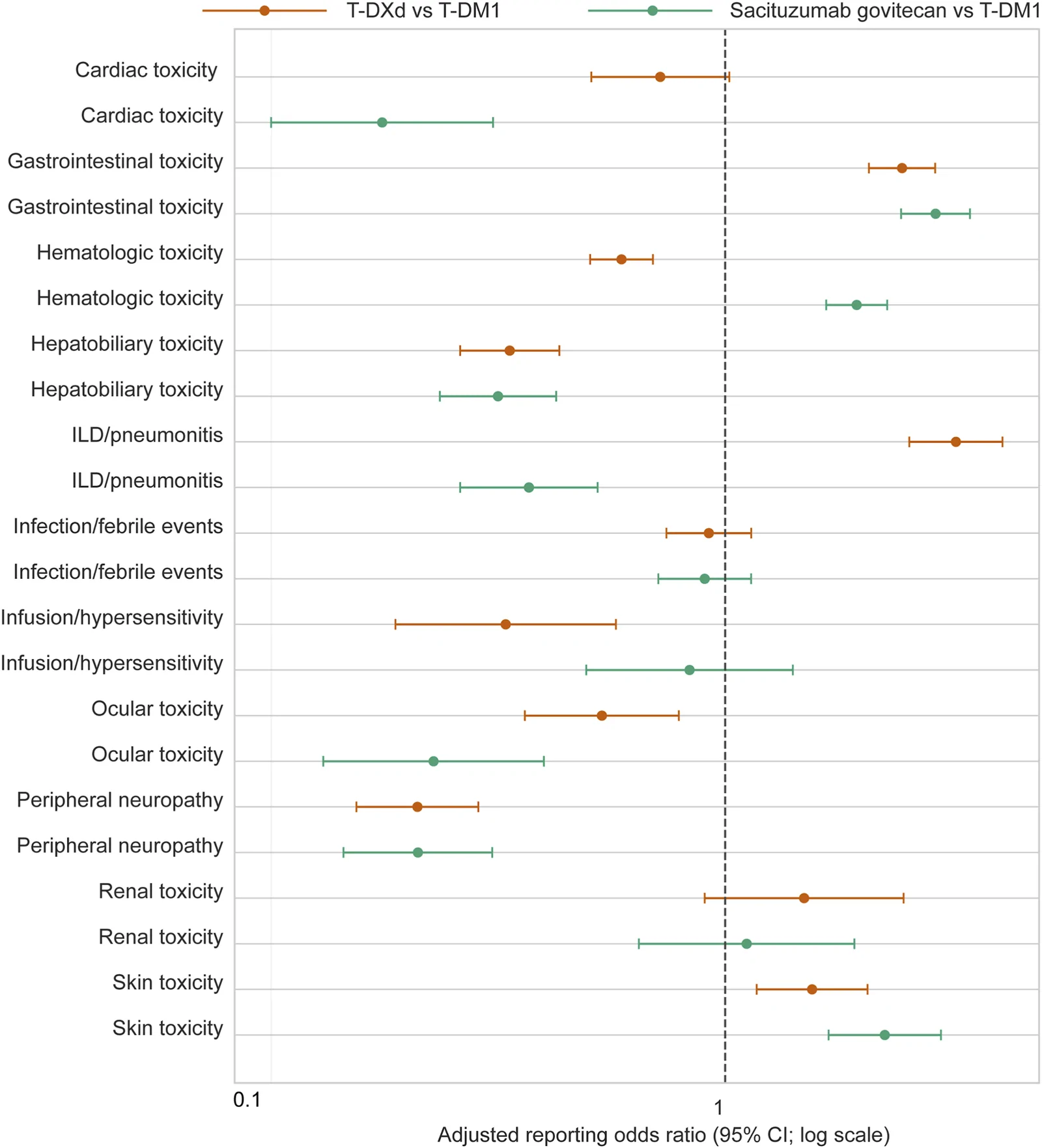
Intraclass adjusted reporting odds for prespecified AESI domains. Adjusted reporting odds ratios (aRORs) and 95% confidence intervals compare T-DXd and sacituzumab govitecan with T-DM1 in the breast-cancer core dataset (N = 13,529). Models were adjusted for age group, sex group, report year, reporter region, reporter type, and concomitant-medication count. The x-axis is logarithmic, and the vertical dashed line denotes the null value (aROR = 1). AESI, adverse event of special interest; aROR, adjusted reporting odds ratio; CI, confidence interval; SG, sacituzumab govitecan; T-DM1, trastuzumab emtansine; T-DXd, trastuzumab deruxtecan.

All 22 prespecified drug–AESI contrasts successfully converged in the primary breast-cancer comparative dataset (N = 13,529). The adjusted reporting models compared T-DXd and sacituzumab govitecan with T-DM1 while accounting for age group, sex group, report year, reporter region, reporter type, and concomitant-medication count. These analyses were prespecified exploratory comparisons within the submitted breast-cancer FAERS report set and therefore quantify relative reporting patterns rather than comparative clinical risk (Figure 5; Table 3).

**TABLE 3:** Intraclass adjusted reporting odds in the breast-cancer core comparative dataset.

AESI domain	Events, n	T-DXd vs. T-DM1, aROR (95% CI)	P value	Sacituzumab govitecan vs. T-DM1, aROR (95% CI)	P value
ILD/pneumonitis	1,057	3.23 (2.55–4.09)	<0.001	0.37 (0.26–0.52)	<0.001
Hematologic toxicity	1,965	0.59 (0.50–0.69)	<0.001	1.95 (1.67–2.28)	<0.001
Gastrointestinal toxicity	2,263	2.45 (2.07–2.90)	<0.001	2.91 (2.44–3.46)	<0.001
Hepatobiliary toxicity	515	0.34 (0.26–0.43)	<0.001	0.32 (0.24–0.42)	<0.001
Cardiac toxicity	262	0.72 (0.51–1.02)	0.065	0.18 (0.10–0.31)	<0.001
Ocular toxicity	187	0.53 (0.36–0.79)	0.002	0.23 (0.13–0.40)	<0.001
Skin toxicity	642	1.55 (1.17–2.06)	0.002	2.25 (1.69–2.99)	<0.001
Infection/febrile events	861	0.92 (0.74–1.14)	0.451	0.90 (0.71–1.14)	0.387
Infusion/hypersensitivity	133	0.33 (0.19–0.57)	<0.001	0.83 (0.49–1.41)	0.5
Renal toxicity	172	1.49 (0.90–2.47)	0.119	1.12 (0.65–1.93)	0.696
Peripheral neuropathy	319	0.21 (0.15–0.29)	<0.001	0.21 (0.14–0.31)	<0.001

Domain-specific adjusted reporting odds ratios compare T-DXd, and sacituzumab govitecan with T-DM1. Models include age group, sex group, report year, reporter region, reporter type, and concomitant-medication count. Estimates represent relative reporting within the submitted core ADC, report set. Analyses were prespecified and exploratory; P values are nominal and were not adjusted for multiplicity. ADC, antibody–drug conjugate; AESI, adverse event of special interest; aROR, adjusted reporting odds ratio; CI, confidence interval; T-DM1, trastuzumab emtansine; T-DXd, trastuzumab deruxtecan.

Compared with T-DM1, T-DXd demonstrated higher adjusted reporting odds for ILD/pneumonitis (adjusted reporting odds ratio [aROR], 3.23; 95% confidence interval [CI], 2.55–4.09), gastrointestinal toxicity (aROR, 2.45; 95% CI, 2.07–2.90), and skin toxicity (aROR, 1.55; 95% CI, 1.17–2.06). In contrast, lower reporting odds were observed for hematologic toxicity (aROR, 0.59; 95% CI, 0.50–0.69), hepatobiliary toxicity (aROR, 0.34; 95% CI, 0.26–0.43), ocular toxicity (aROR, 0.53; 95% CI, 0.36–0.79), infusion/hypersensitivity reactions (aROR, 0.33; 95% CI, 0.19–0.57), and peripheral neuropathy (aROR, 0.21; 95% CI, 0.15–0.29). No statistically distinguishable differences were observed for cardiac toxicity, infection/febrile events, or renal toxicity, as the corresponding confidence intervals included the null value.

Relative to T-DM1, sacituzumab govitecan demonstrated higher adjusted reporting odds for hematologic toxicity (aROR, 1.95; 95% CI, 1.67–2.28), gastrointestinal toxicity (aROR, 2.91; 95% CI, 2.44–3.46), and skin toxicity (aROR, 2.25; 95% CI, 1.69–2.99). Lower reporting odds were observed for ILD/pneumonitis (aROR, 0.37; 95% CI, 0.26–0.52), hepatobiliary toxicity (aROR, 0.32; 95% CI, 0.24–0.42), cardiac toxicity (aROR, 0.18; 95% CI, 0.10–0.31), ocular toxicity (aROR, 0.23; 95% CI, 0.13–0.40), and peripheral neuropathy (aROR, 0.21; 95% CI, 0.14–0.31). By contrast, infection/febrile events, infusion/hypersensitivity reactions, and renal toxicity did not demonstrate statistically distinguishable differences from T-DM1 because the corresponding confidence intervals crossed the null value.

### 3.6 Machine-learning signal prioritization in temporal validation

To complement rule-based preferred-term (PT) identification and expert clinical review, we developed report-level machine-learning models to prioritize submitted FAERS reports containing prespecified adverse events of special interest (AESIs). Model development used 5,288 reports submitted through 2022Q4, whereas temporal validation was performed in an independent set of 8,241 reports submitted from 2023Q1 onward. Because the intended application was post-report pharmacovigilance prioritization, the models were designed to classify already submitted reports according to the presence of predefined AESIs rather than to estimate individual patient risk or causal drug–toxicity relationships.

**FIGURE 6:**
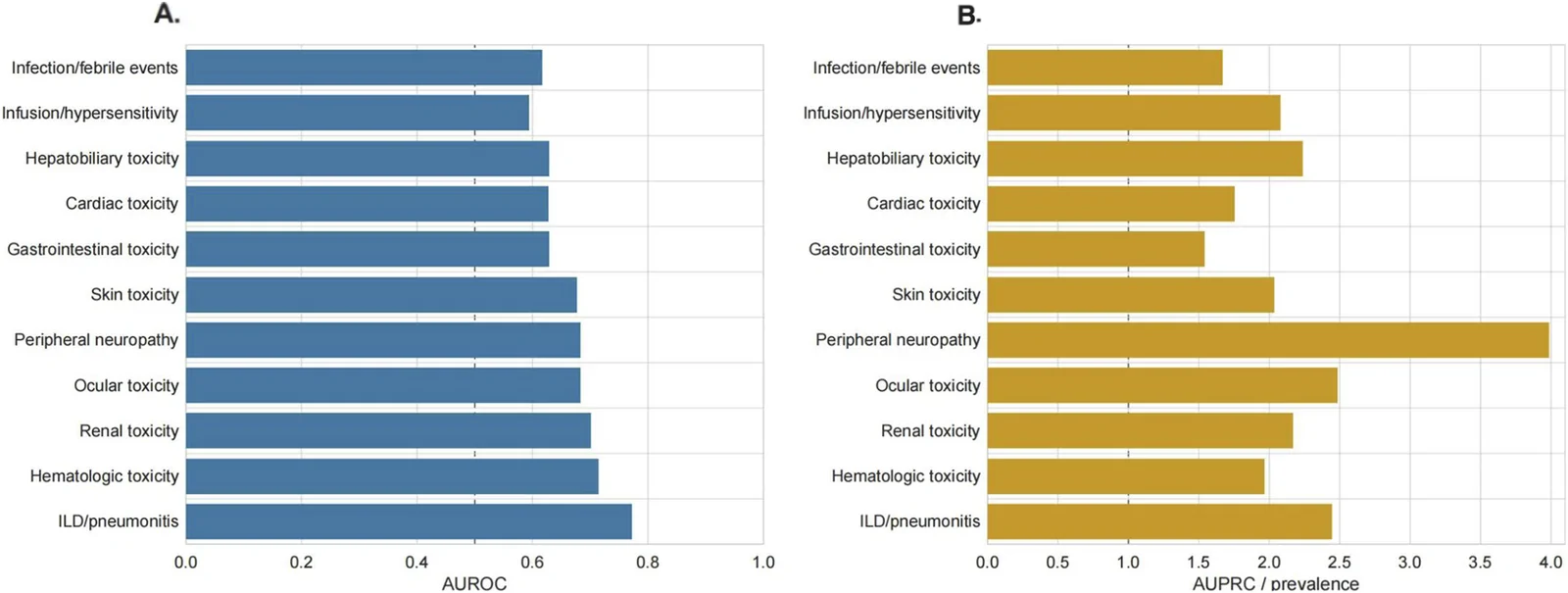
Machine-learning signal prioritization in the breast-cancer core dataset. Penalized logistic-regression models were developed using reports through 2022 Q4 and temporally validated in 8,241 reports from 2023 Q1 to 2025 Q4. **(A)** Temporal-validation area under the receiver operating characteristic curve (AUROC) across the 11 prespecified adverse events of special interest (AESIs); the dashed vertical line at AUROC = 0.5 indicates chance-level discrimination. **(B)** Area under the precision–recall curve (AUPRC) normalized to the corresponding AESI prevalence in the temporal-validation set; the dashed vertical line at AUPRC/prevalence = 1 indicates the prevalence baseline. The primary panels show the full contextual model (M1). Complementary feature-ablation analyses comparing the drug-only model (M0), the full contextual model including drug identity (M1), and the same contextual model excluding drug identity (M2) are reported in Supplementary Table S4. Models were evaluated solely as report-level signal-prioritization tools for pharmacovigilance review and were not intended for patient-level risk prediction or comparative drug-safety inference. AUPRC, area under the precision–recall curve; AUROC, area under the receiver operating characteristic curve; ML, machine learning.

Across the 11 prespecified AESI domains, temporal-validation performance varied by outcome (Figure 6; Supplementary Table S4). In the full contextual model (M1), AUROC ranged from 0.593 for infusion/hypersensitivity reactions to 0.772 for ILD/pneumonitis. The ILD/pneumonitis model achieved an AUPRC of 0.216 against a validation prevalence of 0.089, corresponding to a 2.44-fold precision–recall lift over prevalence. Hematologic toxicity achieved an AUROC of 0.714 and an AUPRC lift of 1.96, whereas peripheral neuropathy achieved an AUROC of 0.683 and the highest AUPRC lift, at 3.99-fold over prevalence. Top-decile enrichment also varied across domains, reaching 3.39-fold for ocular toxicity and 3.31-fold for peripheral neuropathy.

To determine the extent to which model performance depended on suspect-drug identity, we performed a prespecified feature-ablation analysis comparing a drug-only baseline model (M0), the full contextual model including drug identity (M1), and the same contextual model with drug identity removed (M2) (Supplementary Table S4). M1 achieved a higher AUROC than M0 in 10 of the 11 AESI domains, indicating that drug identity alone did not account for most of the observed discrimination. The largest AUROC improvements over M0 were observed for renal toxicity (+0.194), ocular toxicity (+0.180), ILD/pneumonitis (+0.112), skin toxicity (+0.112), gastrointestinal toxicity (+0.109), infection/febrile events (+0.109), and peripheral neuropathy (+0.108). Conversely, comparison of M1 with M2 demonstrated that the incremental contribution of drug identity was domain dependent. Removing drug identity reduced AUROC for ILD/pneumonitis (0.772–0.706), cardiac toxicity (0.627–0.574), hematologic toxicity (0.714–0.666), hepatobiliary toxicity (0.628–0.587), and peripheral neuropathy (0.683–0.651), whereas discrimination was largely preserved for gastrointestinal, skin, infection/febrile, and renal toxicity. Collectively, these ablation analyses indicate that contextual report features contributed discriminatory information beyond drug identity alone, while drug identity provided additional information for selected AESI domains.

Overall, the machine-learning analyses demonstrated heterogeneous, domain-specific performance rather than a uniform drug-identity-driven pattern. Detailed discrimination, precision–recall, prevalence, calibration, confidence-interval, and top-decile enrichment metrics are provided in Supplementary Table S4.

These machine-learning analyses were intended solely to support post-report pharmacovigilance workflows by prioritizing submitted reports for subsequent manual review. They complement, rather than replace, rule-based PT identification and expert clinical assessment and should not be interpreted as comparative drug-safety estimates, patient-level toxicity prediction, or clinical decision-support models.

### 3.7 Supportive all-indication sensitivity and cold-start analyses

To evaluate the robustness of the primary breast-cancer-restricted analyses, we repeated the adjusted reporting analyses in the supportive all-indication core comparative dataset (N = 20,526). The principal reporting patterns remained unchanged (Supplementary Figure S1; Supplementary Table S3). Compared with T-DM1, T-DXd continued to demonstrate higher adjusted reporting odds for ILD/pneumonitis (aROR, 3.41; 95% CI, 2.77–4.18) and gastrointestinal toxicity (aROR, 2.56; 95% CI, 2.22–2.95), whereas sacituzumab govitecan retained higher adjusted reporting odds for hematologic toxicity (aROR, 2.02; 95% CI, 1.76–2.32) and gastrointestinal toxicity (aROR, 2.39; 95% CI, 2.06–2.78). No material reversal of the principal drug-specific reporting profiles was observed, supporting the stability of the primary breast-cancer findings across the broader all-indication FAERS population.

Because post-marketing exposure to newly introduced ADCs remained limited during the study period, Dato-DXd and SKB264 were evaluated separately as descriptive cold-start cohorts rather than being incorporated into comparative analyses. Within the all-indication Dato-DXd cohort, the most frequently captured clinically interpretable preferred terms included stomatitis (n = 42), interstitial lung disease (n = 11), dry eye (n = 8), pneumonitis (n = 5), and keratitis (n = 3) (Supplementary Figure S2; Supplementary Table S2B). Given the limited number of reports and resulting statistical instability, these observations were considered descriptive and hypothesis-generating, were not included in adjusted reporting or machine-learning analyses, and should not be interpreted as head-to-head toxicity comparisons with the three core ADCs. SKB264 remained too sparsely represented for stable signal characterization, whereas no FAERS reports involving disitamab vedotin (RC48/RC48-ADC) were identified during the study period. Accordingly, the absence of RC48 signals reflects the absence of report capture rather than evidence of clinical safety.

### 3.8 Institutional cohort characteristics

The independently assembled institutional cohort comprised 105 consecutive patients treated with T-DM1 (n = 31), T-DXd (n = 50), or sacituzumab govitecan (n = 24). Overall, the cohort represented a heavily pretreated advanced breast cancer population, with a median age of 52 years (interquartile range [IQR], 47–57), 97 of 105 patients (92.4%) having an Eastern Cooperative Oncology Group (ECOG) performance status of 0–1, and 102 patients (97.1%) presenting with stage IV disease. Patients had received a median of three prior systemic treatment lines (IQR, 2–4), while visceral, bone, and brain metastases were documented in 75 (71.4%), 59 (56.2%), and 11 (10.5%) patients, respectively. Previous exposure to another antibody–drug conjugate (ADC) was recorded in 10 patients (9.5%). The median duration of treatment with the index ADC was eight cycles (IQR, 5–11), and the median follow-up duration was 324 days (IQR, 281–388).

The three treatment groups reflected their approved clinical indications rather than comparable study populations. All patients receiving T-DM1 had HER2-positive disease, whereas the T-DXd cohort comprised both HER2-positive (33/50, 66.0%) and HER2-low (17/50, 34.0%) disease. In contrast, the sacituzumab govitecan cohort consisted predominantly of patients with triple-negative breast cancer (18/24, 75.0%), with the remainder having hormone receptor-positive/HER2-negative disease (6/24, 25.0%). Treatment history also differed across groups, with median prior treatment lines increasing from two in the T-DM1 cohort to three in the T-DXd cohort and four in the sacituzumab govitecan cohort. Prior exposure to another ADC was absent among patients receiving T-DM1 but was observed in six T-DXd recipients and four sacituzumab govitecan recipients.

These baseline differences primarily reflect differences in approved indications, treatment sequencing, and routine clinical practice rather than differences arising from study design. Accordingly, the institutional cohort was used exclusively for descriptive clinical contextualization, and between-group comparisons without baseline-covariate adjustment were interpreted as exploratory observations rather than comparative assessments of treatment safety or effectiveness (Supplementary Table S7).

### 3.9 Institutional AESI patterns

**FIGURE 7:**
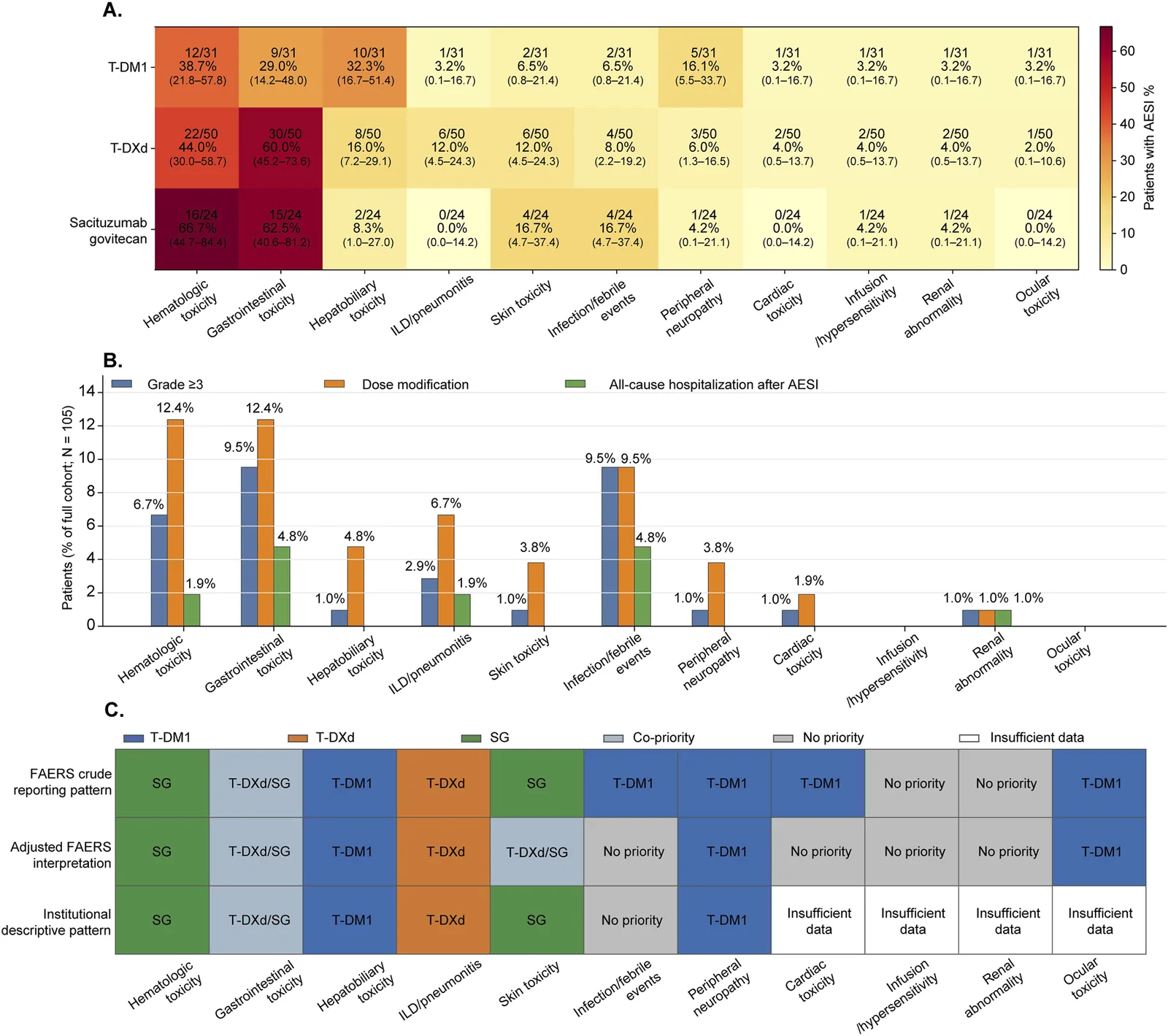
Institutional AESI patterns, clinical consequences, and cross-layer contextualization. **(A)** Heatmap of institutional AESI proportions by ADC (T-DM1, n = 31; T-DXd, n = 50; sacituzumab govitecan, n = 24). Each cell shows the number of patients with the AESI over the treatment-group denominator, the observed percentage, and the Clopper–Pearson 95% exact CI in parentheses. **(B)** AESI severity and clinical consequences across the full institutional cohort (N = 105), including Grade ≥3 events, dose modification, and all-cause hospitalization after AESI onset. Hospitalization indicates temporal association rather than causal attribution. All 12 deaths during follow-up were attributed to disease progression and are not shown as AESI-specific outcomes. **(C)** Cross-layer contextualization of crude FAERS reporting patterns, adjusted FAERS reporting contrasts, and institutional observations across prespecified AESI domains. Co-priority indicates similarly prominent patterns for two ADCs; No priority indicates no clear directional priority; Insufficient data indicates inadequate institutional evidence for stable interpretation. Cross-layer patterns are descriptive and do not indicate statistical concordance, external validation, incidence, or comparative safety. ADC, antibody–drug conjugate; AESI, adverse event of special interest; CI, confidence interval; FAERS, FDA Adverse Event Reporting System; SG, sacituzumab govitecan; T-DM1, trastuzumab emtansine; T-DXd, trastuzumab deruxtecan.

The institutional cohort demonstrated distinct AESI patterns across the three indication-defined treatment groups (Figure 7A; Supplementary Table S5). Hematologic toxicity was most frequently documented among patients receiving sacituzumab govitecan, gastrointestinal toxicity was common with both T-DXd and sacituzumab govitecan, hepatobiliary toxicity occurred most frequently in the T-DM1 cohort, and ILD/pneumonitis was concentrated among T-DXd recipients. Given the indication-defined composition and baseline heterogeneity of the three groups, these observations should be interpreted descriptively rather than as comparative estimates of treatment safety.

Hematologic toxicity was documented in 12 of 31 T-DM1 recipients (38.7%; 95% confidence interval [CI], 21.8%–57.8%), 22 of 50 T-DXd recipients (44.0%; 95% CI, 30.0%–58.7%), and 16 of 24 sacituzumab govitecan recipients (66.7%; 95% CI, 44.7%–84.4%). Platelet-related abnormalities predominated in the T-DM1 cohort, whereas neutropenia, leukopenia, anemia, and febrile neutropenia were more prominent among T-DXd and sacituzumab govitecan recipients. Gastrointestinal toxicity occurred in 9 of 31 T-DM1 recipients (29.0%), 30 of 50 T-DXd recipients (60.0%), and 15 of 24 sacituzumab govitecan recipients (62.5%). Nausea, vomiting, and appetite reduction were prominent in the T-DXd cohort, whereas diarrhea was a characteristic gastrointestinal manifestation among sacituzumab govitecan recipients.

Hepatobiliary toxicity was documented in 10 of 31 T-DM1 recipients (32.3%), compared with 8 of 50 T-DXd recipients (16.0%) and 2 of 24 sacituzumab govitecan recipients (8.3%), and consisted mainly of elevations in alanine aminotransferase, aspartate aminotransferase, and bilirubin. ILD/pneumonitis occurred in 7 of 105 patients overall, including 6 of 50 T-DXd recipients (12.0%), 1 of 31 T-DM1 recipients (3.2%), and no sacituzumab govitecan recipients. Source-record re-audit of all seven ILD/pneumonitis cases, including available respiratory consultation, imaging, and treatment records, confirmed three Grade 3 events, all in the T-DXd group, with no Grade 4 or Grade 5 events. Among the six T-DXd cases, the median onset was 182 days (IQR, 128–205.2); all six were associated with at least one treatment interruption, treatment delay, or permanent discontinuation, and two were followed by hospitalization. No ILD/pneumonitis-related death was identified during follow-up (Supplementary Table S5).

Other AESI domains occurred less frequently. Skin toxicity was documented in 2 of 31 T-DM1 recipients (6.5%), 6 of 50 T-DXd recipients (12.0%), and 4 of 24 sacituzumab govitecan recipients (16.7%). Infection/febrile events occurred in 2, 4, and 4 patients, respectively, with five patients subsequently hospitalized. Peripheral neuropathy was most frequent among T-DM1 recipients (5/31, 16.1%). Cardiac, infusion/hypersensitivity, and renal toxicities were uncommon across the cohort. Source-record re-audit identified two ocular AESIs: one Grade 1 dry-eye event in a T-DXd recipient and one Grade 1 conjunctivitis/dry-eye event in a T-DM1 recipient. Both were managed with topical symptomatic treatment and neither required dose reduction, treatment interruption, or discontinuation. No ocular AESI occurred in the sacituzumab govitecan group, and no Grade ≥2 ocular toxicity was identified (Supplementary Table S5).

### 3.10 Institutional severity, dose management, and temporal clinical consequences

Across the full institutional cohort, gastrointestinal and hematologic AESIs represented the largest overall documented burdens, affecting 54 of 105 patients (51.4%) and 50 of 105 patients (47.6%), respectively. Grade ≥3 events were recorded in 10 patients with gastrointestinal toxicity and 7 with hematologic toxicity. Dose interruption, dose reduction, treatment delay, or permanent discontinuation occurred in 13 patients in each domain. All-cause hospitalization after AESI onset was documented in five patients with gastrointestinal toxicity and 2 with hematologic toxicity (Figure 7B; Supplementary Table S6).

Clinically consequential events were also observed in several less frequent AESI domains. Hepatobiliary toxicity affected 20 patients, including one Grade ≥3 event and five cases associated with dose modification. ILD/pneumonitis occurred in seven patients; source-record re-audit confirmed three Grade 3 events, all in T-DXd recipients, with no Grade 4 or Grade 5 events. All seven patients underwent at least one dose-management action, and two were subsequently hospitalized. Infection/febrile events were documented in 10 patients; all 10 were Grade ≥3, all were associated with a dose-management intervention, and five were followed by hospitalization. Other AESI domains contributed a smaller overall burden and are summarized in Supplementary Table S6.

Twelve all-cause deaths occurred during cohort follow-up. Case-by-case review of the electronic medical records and follow-up documentation attributed all 12 deaths to disease progression, and no death was attributed to an institutional AESI. Death was therefore summarized at the cohort level rather than as an AESI-specific temporal consequence. Hospitalization after AESI onset was retained as an all-cause temporal clinical-consequence marker and was not interpreted as evidence of causal attribution to the corresponding AESI. These findings describe the severity and treatment consequences observed within the institutional cohort and were used for descriptive clinical contextualization rather than formal comparative safety assessment.

### 3.11 Exploratory institutional comparisons

Exploratory comparisons were performed to characterize differences in the distribution of documented AESIs across the three indication-defined institutional treatment groups. These analyses were not adjusted for baseline covariates. Among the prespecified AESI domains, gastrointestinal toxicity showed the strongest evidence of overall group heterogeneity in the three-group Fisher–Freeman–Halton exact test (nominal P = 0.0128). Hematologic toxicity (nominal P = 0.0954), hepatobiliary toxicity (nominal P = 0.0723), and ILD/pneumonitis (nominal P = 0.1742) did not show statistically distinguishable overall distributions, and no clear overall differences were identified for the remaining AESI domains.

In prespecified pairwise analyses, nominal differences were observed for gastrointestinal toxicity between T-DM1 and T-DXd (nominal P = 0.0113) and between T-DM1 and sacituzumab govitecan (nominal P = 0.0161), as well as for hepatobiliary toxicity between T-DM1 and sacituzumab govitecan (nominal P = 0.0485). Holm correction was applied across the 12 prespecified pairwise comparisons to account for multiple testing, and none of these contrasts remained statistically significant after adjustment.

Because the treatment groups were small and clinically heterogeneous, and ADC selection was determined by disease subtype, treatment history, and clinical indication, these analyses should be interpreted as exploratory descriptions of observed AESI distributions rather than comparative estimates of drug-associated safety or causal treatment effects (Supplementary Table S7).

### 3.12 Cross-layer clinical contextualization

Several major toxicity patterns showed directional alignment across the FAERS and institutional evidence layers (Figure 7C; Supplementary Tables S8A,B). Cross-layer interpretation was descriptive and considered the direction and precision of adjusted FAERS contrasts together with the amount of available institutional evidence. Domains with non-distinguishable adjusted contrasts or sparse institutional events were classified as having no clear priority or insufficient data.

T-DM1 showed a consistent hepatobiliary pattern, with the highest crude FAERS reporting proportion, higher adjusted reporting odds than both comparator ADCs, and the highest institutional proportion. T-DXd showed the clearest ILD/pneumonitis pattern, with higher adjusted reporting odds than T-DM1 and six of the seven institutional cases occurring among T-DXd recipients. Sacituzumab govitecan showed higher adjusted hematologic reporting odds than T-DM1 and the highest institutional hematologic proportion. Gastrointestinal toxicity was prominent for both T-DXd and sacituzumab govitecan across FAERS and institutional observations. Peripheral neuropathy was directionally aligned with T-DM1, whereas skin toxicity showed a broadly similar but less definitive cross-layer pattern. In contrast, infection/febrile events and renal abnormality showed no distinguishable adjusted FAERS priority, and cardiac, infusion/hypersensitivity, renal, and ocular events were too sparse in the institutional cohort for stable cross-layer interpretation. These differences are expected because FAERS is influenced by selective reporting, notoriety, incomplete information, and differential exposure, whereas institutional ascertainment reflects active clinical monitoring and may capture events that are less likely to be spontaneously reported.

No formal statistical pooling was performed across FAERS reports and institutional patients because the two sources differed in analytical units, denominators, ascertainment, and data-generating mechanisms. Machine-learning analyses remained an independent report-level prioritization layer. Accordingly, cross-layer alignment represents descriptive clinical contextualization rather than external validation, confirmation of toxicity incidence, comparative safety assessment, or evidence of patient-level causal risk.

## 4 Discussion

This breast-cancer-restricted FAERS analysis identified distinct postmarketing safety-reporting patterns for T-DM1, T-DXd, and sacituzumab govitecan across complementary pharmacovigilance, machine-learning, and institutional evidence layers. FAERS characterized relative event concentrations within submitted reports, the machine-learning component provided exploratory report-level prioritization, and the institutional cohort supplied patient-level clinical context. These layers were analyzed separately because they differ in denominator, ascertainment, and inferential scope and should not be interpreted as interchangeable estimates of comparative clinical risk. Newer ADCs with limited postmarketing capture were evaluated separately to avoid unstable comparisons with the three core agents.

T-DM1 showed prominent platelet-related and hepatobiliary reporting patterns, including thrombocytopenia, platelet-count reductions, aminotransferase elevations, and chronic hepatic terms. These findings are consistent with established clinical safety evidence and recent postmarketing analyses of hepatotoxicity and HER2-targeting ADCs (Verma et al., 2012; von et al., 2019; Krop et al., 2014; Dong et al., 2025; Yang et al., 2026). In the institutional cohort, hematologic and hepatobiliary AESIs occurred in 12/31 and 10/31 T-DM1 recipients, respectively, with platelet abnormalities predominating among hematologic events. Together, these observations reinforce the importance of platelet-count and hepatic laboratory monitoring during T-DM1 treatment. Chronic hepatic terms identified in FAERS should nevertheless prompt case-level assessment of baseline liver disease, cumulative exposure, and competing causes rather than being interpreted as direct evidence of drug-attributable chronic liver injury.

T-DXd was characterized primarily by ILD/pneumonitis and gastrointestinal toxicity. ILD/pneumonitis showed higher adjusted reporting odds than T-DM1, consistent with clinical-trial, pooled safety, pharmacovigilance, and regulatory evidence identifying pulmonary toxicity as a major T-DXd safety concern (Modi et al., 2020; Cortés et al., 2022; Modi et al., 2022; Bardia et al., 2024b; Powell et al., 2022; Swain et al., 2022; Park et al., 2025). ILD/pneumonitis also showed the highest temporal-validation AUROC among the evaluated AESIs, indicating comparatively stronger report-level discrimination within the exploratory prioritization framework rather than greater clinical risk. Six of seven institutional ILD/pneumonitis cases occurred among T-DXd recipients, with a median onset of approximately 182 days; all six required treatment interruption, delay, or discontinuation, and two were followed by hospitalization. Gastrointestinal AESIs were documented in 30/50 T-DXd recipients, most commonly involving nausea, vomiting, and appetite reduction. These findings support continued pulmonary and gastrointestinal surveillance throughout treatment, while the small number of institutional ILD cases and absence of independent radiologic adjudication limit stronger causal interpretation.

Sacituzumab govitecan showed prominent hematologic and gastrointestinal reporting patterns, with higher adjusted reporting odds for both domains relative to T-DM1. Neutropenia, febrile neutropenia, and diarrhea were among the major retained PT signals, consistent with ASCENT, TROPiCS-02, current prescribing information, and recent pharmacovigilance evidence for sacituzumab govitecan-associated neutropenia (Bardia et al., 2021; Rugo et al., 2023; Xu et al., 2025). Institutional hematologic AESIs occurred in 16/24 recipients and gastrointestinal AESIs in 15/24, with diarrhea predominating in the latter. These findings support proactive hematologic monitoring, assessment of febrile complications, and gastrointestinal supportive care during treatment.

Previous pharmacovigilance studies have identified pulmonary toxicity with T-DXd, hematologic toxicity with sacituzumab govitecan, hepatotoxicity across ADCs, and broader drug-specific safety patterns among HER2-targeting ADCs (Zhu et al., 2026; Yang et al., 2026; Jeral et al., 2026; Hosonaka et al., 2026). However, many prior analyses focused on individual agents, specific toxicity domains, or broader reporting populations. The present study extends this literature through breast-cancer-restricted intraclass comparisons, clinically curated PT-level analysis, reported TTO characterization, temporal report-level prioritization, and an independently assembled clinical cohort. Its principal contribution is therefore not the identification of previously unknown toxicities, but refinement of the relative reporting context, temporal characteristics, and clinical interpretation of established safety domains across complementary evidence sources.

Newer ADCs require more cautious interpretation. Dato-DXd showed descriptive signals involving stomatitis, ILD/pneumonitis, and ocular events, but limited report numbers produced substantial statistical instability and precluded adjusted comparative or machine-learning analyses. These findings are hypothesis-generating and should not be interpreted as head-to-head safety comparisons with mature ADCs (Bardia et al., 2025; Bardia et al., 2024a; Rugo et al., 2025). SKB264 capture was insufficient for stable characterization, whereas the absence of RC48 reports under the prespecified search strategy indicates limited database capture rather than favorable safety.

Cross-layer interpretation also requires caution because spontaneous reporting and institutional clinical observation represent different data-generating processes. FAERS is affected by underreporting, stimulated reporting, reporting notoriety, missing information, comparator choice, and differential exposure, and neither spontaneous report counts nor disproportionality estimates provide incidence measures (Kant et al., 2022; van Puijenbroek et al., 2002; Cutroneo et al., 2023; Gravel et al., 2024). Institutional ascertainment, by contrast, reflects active clinical monitoring, treatment context, and screening intensity. Directional alignment was most apparent for T-DXd-associated ILD/pneumonitis, T-DM1-associated hepatobiliary toxicity and peripheral neuropathy, and sacituzumab govitecan-associated hematologic toxicity; gastrointestinal and skin toxicity showed broader shared patterns involving T-DXd and sacituzumab govitecan. Other domains showed no clear alignment or were not assessable because adjusted FAERS contrasts were non-distinguishable or institutional events were sparse (Figure 7C; Supplementary Tables S8A,B). Cross-layer similarity therefore represents clinical contextualization rather than statistical concordance, external validation, or confirmation of comparative risk.

The institutional cohort further illustrates the limitations of direct between-group comparison. Treatment groups were indication-defined and differed in tumor subtype, prior treatment, prior ADC exposure, treatment duration, metastatic burden, and follow-up. Institutional statistical analyses were therefore exploratory and were intended to describe observed heterogeneity rather than treatment effects. Although gastrointestinal AESIs showed overall group heterogeneity, none of the prespecified pairwise comparisons remained statistically distinguishable after Holm adjustment.

The machine-learning component was designed for report-level pharmacovigilance prioritization rather than individual-patient prediction. Previous studies have explored artificial intelligence, multivariable prediction, timing features, and machine-learning methods for signal detection and prioritization of individual case safety reports (Al-Azzawi et al., 2023; Battini et al., 2024; Gosselt et al., 2022; Dauner et al., 2023). In the present study, feature-ablation analyses comparing the drug-only model, the full contextual model including drug identity, and the corresponding model without drug identity showed that discrimination reflected varying contributions from both suspect-drug identity and report-level context. Temporal validation and precision–recall analyses therefore quantify enrichment of later FAERS reports for focused review rather than individualized toxicity risk. Consistent with current prediction-model reporting and appraisal principles, these models should complement rather than replace disproportionality screening, clinical PT review, and expert assessment (Collins et al., 2024; Moons et al., 2025; Christodoulou et al., 2019).

From a translational perspective, the framework has two complementary applications. For pharmacovigilance teams, temporal report-level models may assist in prioritizing submitted reports for focused review when used alongside conventional signal-detection methods. For clinical practice, surveillance should remain ADC specific: platelet and hepatic monitoring for T-DM1, continued pulmonary vigilance for T-DXd, and proactive hematologic, febrile, and gastrointestinal monitoring for sacituzumab govitecan. Treatment modification should continue to follow established prescribing information and toxicity-management guidance rather than FAERS-derived reporting contrasts.

Several methodological features strengthen the study, including audited processing of FAERS quarterly files from 2013Q1 through 2025Q4, restriction of the primary analysis to breast-cancer reports, explicit separation of primary and supportive populations, transparent PT eligibility and clinical-review procedures, adjusted intraclass reporting contrasts, reported TTO analyses, temporal machine-learning validation with feature ablation, and integration of an independently assembled institutional cohort.

## 5 Limitations and future directions

Several limitations should be considered. First, FAERS is subject to underreporting, stimulated reporting, incomplete information, unavailable exposure denominators, and differential reporting maturity; disproportionality estimates therefore reflect reporting patterns rather than incidence or causal risk. TTO was available only for subsets of reports and differed in completeness across drugs and AESI domains. Second, the institutional cohort was retrospective, single-center, relatively small, and composed of clinically heterogeneous indication-defined groups, limiting generalizability and precluding formal comparative safety inference. Third, the machine-learning models were developed to prioritize submitted reports containing prespecified AESIs and were not designed for patient-level toxicity prediction. Fourth, postmarketing capture for Dato-DXd and SKB264 remained limited, while no RC48 reports were identified under the prespecified search strategy. Future work should include multicenter clinical evaluation, integration of complementary pharmacovigilance and real-world databases, prospective assessment of report-prioritization workflows, and development of independently validated patient-level toxicity prediction models.

## 6 Conclusion

This breast-cancer-restricted study integrated FAERS pharmacovigilance analyses, temporal machine-learning prioritization, and descriptive clinical contextualization to characterize postmarketing safety-reporting patterns of T-DM1, T-DXd, and sacituzumab govitecan. T-DM1 demonstrated predominant platelet-related and hepatobiliary reporting patterns, T-DXd showed prominent ILD/pneumonitis and gastrointestinal reporting patterns, and sacituzumab govitecan showed prominent hematologic and gastrointestinal reporting patterns. The independently assembled institutional cohort of 105 ADC-treated patients provided clinical context for several major FAERS patterns, including platelet and hepatobiliary abnormalities with T-DM1, ILD/pneumonitis predominantly observed with T-DXd, and frequent hematologic toxicity and diarrhea among sacituzumab govitecan recipients. Temporal machine-learning models provided an additional report-level triage layer for prioritizing submitted safety reports for manual review, while supportive sensitivity and cold-start analyses informed interpretation across ADCs with different reporting maturity. Together, these complementary evidence layers provide a framework for pharmacovigilance signal refinement, toxicity-focused monitoring, and safety-hypothesis generation. However, the findings should not be interpreted as estimates of treatment incidence, causal drug effects, or individual patient risk and require confirmation in larger denominator-defined prospective cohorts.

## Data Availability

The original contributions presented in the study are included in the article/Supplementary Material, further inquiries can be directed to the corresponding author.

## Supplementary material

SUPPLEMENTARY FIGURE S1 Supportive all-indication adjusted reporting-odds sensitivity analysis. The primary adjusted reporting-odds analysis was repeated in the all-indication core comparative dataset (N = 20,526). Adjusted reporting odds ratios (aRORs) and 95% confidence intervals compare T-DXd and sacituzumab govitecan with T-DM1 across the 11 prespecified AESI domains. The x-axis is logarithmic, and the vertical dashed line denotes the null value (aROR = 1). This analysis was supportive and did not replace the prespecified breast-cancer primary analysis. AESI, adverse event of special interest; aROR, adjusted reporting odds ratio; CI, confidence interval; SG, sacituzumab govitecan; T-DM1, trastuzumab emtansine; T-DXd, trastuzumab deruxtecan.

SUPPLEMENTARY FIGURE S2 Dato-DXd cold-start preferred-term capture. Descriptive all-indication preferred-term estimates are shown for Dato-DXd during its early FAERS capture period (N = 299 primary-suspect reports). Points represent empirical Bayes geometric mean (EBGM) estimates for the displayed preferred terms, with the x-axis shown on a logarithmic scale; labels report the corresponding preferred-term report counts. The panel is intended to document early postmarketing reporting patterns and includes both adverse-event and non-adverse-event preferred terms captured in FAERS. Given the limited report volume and early exposure window, these cold-start estimates are descriptive and should not be interpreted as stable comparative safety signals or incidence estimates. Dato-DXd, datopotamab deruxtecan; EBGM, empirical Bayes geometric mean; FAERS, FDA Adverse Event Reporting System; PT, Preferred Term.
