# Supplementary material for: Drug-specific safety signal prioritization of antibody-drug conjugates in breast cancer: integrating FAERS pharmacovigilance, machine learning, and clinical contextualization
Source: Front Pharmacol. 2026 Sep 9;17:1941296. doi: 10.3389/fphar.2026.1941296 (PMC13597761; doi:10.3389/fphar.2026.1941296)
Supplement: Supplementary file 1 [file Table1.docx]

**Supplementary Material**

**Preferred-term statistical screening and clinical review**
Preferred-term statistical screening and clinical review. Drug–Preferred Term (PT) disproportionality estimates were generated separately within each prespecified analytic population. For the breast-cancer primary analysis, statistical eligibility required a report count ≥10, ROR025 >1, and either EB05 >2 or IC025 >0. ROR025 denotes the lower 95% confidence bound for the reporting odds ratio, EB05 the lower 5% empirical Bayes bound, and IC025 the lower information-component bound. Statistical screening preceded clinical interpretation. PTs meeting these criteria were independently reviewed by Yuhan Tang and Shaochun Liu using prespecified clinical categories and the adverse event of special interest (AESI) dictionaries, with Yingze Zhu subsequently cross-checking the classifications. Review considered whether each PT represented a clinically interpretable adverse event or primarily reflected disease progression or metastatic involvement, treatment administration or product use, radiation-related effects, or nonspecific clinical deterioration. Discrepant or ambiguous classifications were resolved through joint source review and discussion; unresolved disagreements were adjudicated by Wenhui Zhao. PTs classified as clinically interpretable or rare but clinically relevant were retained in the main panel. The final breast-cancer primary panel contained 138 retained PTs. For Figure 4, retained PTs were ranked separately within each core antibody–drug conjugate (ADC) by descending report count and then descending EB05, and the top 10 PTs per ADC were displayed. PT estimates from different analytic populations were maintained separately and were not pooled.

**Institutional cohort and clinical AESI analysis**

The institutional cohort was analyzed independently from the FDA Adverse Event Reporting System (FAERS) datasets and was used for descriptive clinical contextualization. Following patient-level deduplication using unique institutional health identifiers, baseline characteristics were summarized at the individual-patient level, whereas safety outcomes were summarized at the patient–AESI domain level. Patients could contribute to multiple AESI domains but were counted only once within each domain. When multiple events occurred within the same domain, the highest valid Common Terminology Criteria for Adverse Events (CTCAE) grade and earliest valid onset date were retained for the patient–domain summary.

Drug-specific AESI frequencies were calculated using fixed treatment-group denominators of 31 patients for trastuzumab emtansine (T-DM1), 50 for trastuzumab deruxtecan (T-DXd), and 24 for sacituzumab govitecan. These denominators represent the corresponding treated populations and do not imply uniform protocol-driven ascertainment of every toxicity domain. Time-to-onset (TTO) analyses were restricted to events with complete dates, non-negative intervals, and onset within the documented observation window. For dose-management actions and temporal clinical consequences, an outcome was coded as present only when explicitly documented in the medical record; otherwise, it was coded as absent.

Dose-management outcomes included any documented treatment interruption, dose reduction, treatment delay, or permanent discontinuation associated with the corresponding AESI. Hospitalization and death occurring after AESI onset were summarized separately as all-cause temporal outcomes and were not assumed to be causally attributable to the AESI. For overall-cohort summaries of clinical consequences, Grade ≥3 events, dose-management actions, hospitalization, and death were expressed using the full institutional cohort denominator (N = 105), rather than conditional denominators restricted to patients with the corresponding AESI.

Between-group analyses were exploratory because the three treatment groups represented indication-defined and clinically heterogeneous populations rather than randomized or exchangeable treatment groups. Accordingly, institutional comparisons were used to describe observed AESI distributions and provide clinical context for the FAERS findings, not to estimate comparative treatment effects or establish causal safety differences.

**Machine-learning report-triage framework, and validation**

Separate report-level classifiers were developed for each of the 11 AESI domains. The development set comprised breast-cancer primary reports from 2013–2022, and reports from 2023–2025 were held out as a temporal validation set. Three models were prespecified: M0 included suspect-drug identity alone; M1 included drug identity, age group, sex, geographic region, reporter type, report year, concomitant-medication count, TTO availability, and TTO duration; and M2 included the same contextual predictors as M1 but excluded drug identity. Categorical predictors were imputed using the most frequent category and one-hot encoded, with previously unseen validation categories ignored. Numeric predictors were median-imputed where applicable and standardized. Missing TTO duration was set to zero and represented jointly with a separate TTO-availability indicator.

Penalized logistic regression was fitted using the SAGA solver with balanced class weights. Regularization parameters were selected exclusively within the development set using five-fold stratified cross-validation maximizing the area under the receiver operating characteristic curve (AUROC). The candidate inverse-regularization strengths were C = 0.01, 0.1, 1.0, and 3.0, with l1 ratios of 0, 0.5, and 1.0. Model performance was evaluated in the untouched temporal validation set using AUROC, area under the precision–recall curve (AUPRC), AUPRC relative to outcome prevalence, Brier score, calibration slope, and top-10% enrichment. Ninety-five percent confidence intervals (CIs) for AUROC and AUPRC were obtained using 1,000 bootstrap resamples. Incremental contextual value was assessed by comparing M1 with the drug-only M0 model and with M2 after removal of drug identity.

**Supplementary Table S1. Baseline characteristics of the institutional cohort**

Rows were reconstructed from the deduplicated institutional patient-level dataset, with baseline variables summarized once for each unique patient identifier. Continuous variables are presented as median (interquartile range [IQR]) and categorical variables as n/N (%). P values were calculated using the Kruskal–Wallis test for continuous variables and the χ² test or Fisher's exact test, as appropriate, for categorical variables. Owing to expected differences in treatment indications, molecular subtype, and prior treatment history across ADC groups, these baseline comparisons are descriptive and are provided to characterize cohort composition rather than establish group comparability. Consequently, all institutional between-group safety analyses were considered exploratory and used exclusively for clinical contextualization. ADC, antibody–drug conjugate; ECOG, Eastern Cooperative Oncology Group; HER2, human epidermal growth factor receptor 2; HR, hormone receptor; IQR, interquartile range; T-DM1, trastuzumab emtansine; T-DXd, trastuzumab deruxtecan.

| **Characteristic** | **Overall (N = 105)** | **T-DM1 (N = 31)** | **T-DXd (N = 50)** | **Sacituzumab govitecan (N = 24)** | **P value*** |
| --- | --- | --- | --- | --- | --- |
| Patients, n | 105 | 31 | 50 | 24 | — |
| Age, years, median (IQR) | 52 (47–57) | 53 (50–59) | 52 (47–57) | 49 (42–52.3) |  |
| ECOG performance status, n/N (%) |  |  |  |  |  |
| ECOG 0 | 51/105 (48.6) | 17/31 (54.8) | 24/50 (48.0) | 10/24 (41.7) |  |
| ECOG 1 | 46/105 (43.8) | 12/31 (38.7) | 22/50 (44.0) | 12/24 (50.0) |  |
| ECOG 2 | 8/105 (7.6) | 2/31 (6.5) | 4/50 (8.0) | 2/24 (8.3) |  |
| Molecular subtype, n/N (%) |  |  |  |  | <0.001 |
| HER2-positive | 64/105 (61.0) | 31/31 (100.0) | 33/50 (66.0) | 0/24 (0.0) |  |
| HER2-low | 17/105 (16.2) | 0/31 (0.0) | 17/50 (34.0) | 0/24 (0.0) |  |
| Triple-negative breast cancer | 18/105 (17.1) | 0/31 (0.0) | 0/50 (0.0) | 18/24 (75.0) |  |
| HR-positive/HER2-negative | 6/105 (5.7) | 0/31 (0.0) | 0/50 (0.0) | 6/24 (25.0) |  |
| Disease stage, n/N (%) |  |  |  |  |  |
| Stage III (locally advanced) | 3/105 (2.9) | 1/31 (3.2) | 1/50 (2.0) | 1/24 (4.2) |  |
| Stage IV (metastatic) | 102/105 (97.1) | 30/31 (96.8) | 49/50 (98.0) | 23/24 (95.8) |  |
| Prior treatment lines, median (IQR) | 3 (2–4) | 2 (2–3) | 3 (2–3) | 4 (3–5.3) |  |
| Metastatic sites, n/N (%) |  |  |  |  |  |
| Visceral metastasis | 75/105 (71.4) | 23/31 (74.2) | 34/50 (68.0) | 18/24 (75.0) |  |
| Bone metastasis | 59/105 (56.2) | 19/31 (61.3) | 26/50 (52.0) | 14/24 (58.3) |  |
| Brain metastasis | 11/105 (10.5) | 1/31 (3.2) | 9/50 (18.0) | 1/24 (4.2) |  |
| Any prior ADC exposure, n/N (%) | 10/105 (9.5) | 0/31 (0.0) | 6/50 (12.0) | 4/24 (16.7) |  |
| Treatment cycles, median (IQR) | 8 (5–11) | 8 (6.5–10.5) | 10.5 (7.3–14.8) | 4.5 (4–7.3) |  |
| Follow-up duration, days, median (IQR) | 324 (281–388) | 365 (288–396.5) | 300.5 (262.8–360) | 351 (280.5–397) |  |

**Supplementary Table S2A. Clinical review, analytic eligibility, and final disposition of statistically eligible preferred terms in the breast-cancer primary dataset.**

All preferred terms in Supplementary Table S2A originate from the breast-cancer primary analytic dataset and met the predefined statistical eligibility criteria before standardized clinical review. Clinical-review category, exclusion rationale, retained/excluded status, and final disposition are provided for transparency. CI, confidence interval; EB05, lower 5% empirical Bayes bound; PT, Preferred Term; ROR, reporting odds ratio; SG, sacituzumab govitecan; T-DM1, trastuzumab emtansine; T-DXd, trastuzumab deruxtecan.

| **Drug** | **Preferred Term** | **Report Count (N)** | **ROR (95% CI)** | **EB05** | **Statistical Eligibility** | **Included in Main Panel** | **Clinical Review Category** | **Exclusion Rationale** | **Final Disposition** |
| --- | --- | --- | --- | --- | --- | --- | --- | --- | --- |
| SG | Diarrhoea | 451 | 1.91 (1.73-2.12) | 1.62 | Eligible | Yes | Core Clinical Event | None (Retained) | Retained (Displayed in Figure 4 Top 30) |
| SG | Neutropenia | 381 | 2.82 (2.53-3.14) | 2.32 | Eligible | Yes | Core Clinical Event | None (Retained) | Retained (Displayed in Figure 4 Top 30) |
| SG | Neutrophil count decreased | 137 | 2.95 (2.48-3.51) | 2.36 | Eligible | Yes | Core Clinical Event | None (Retained) | Retained (Displayed in Figure 4 Top 30) |
| SG | Febrile neutropenia | 136 | 3.00 (2.52-3.58) | 2.40 | Eligible | Yes | Core Clinical Event | None (Retained) | Retained (Displayed in Figure 4 Top 30) |
| SG | Anaemia | 123 | 1.45 (1.21-1.74) | 1.22 | Eligible | Yes | Core Clinical Event | None (Retained) | Retained (Displayed in Figure 4 Top 30) |
| SG | Constipation | 95 | 1.39 (1.13-1.70) | 1.14 | Eligible | Yes | Core Clinical Event | None (Retained) | Retained (Displayed in Figure 4 Top 30) |
| SG | Thrombocytopenia | 95 | 1.84 (1.50-2.26) | 1.48 | Eligible | Yes | Core Clinical Event | None (Retained) | Retained (Displayed in Figure 4 Top 30) |
| SG | Abdominal pain | 72 | 1.68 (1.33-2.13) | 1.32 | Eligible | Yes | Core Clinical Event | None (Retained) | Retained (Displayed in Figure 4 Top 30) |
| SG | Heart rate increased | 68 | 6.20 (4.84-7.96) | 4.68 | Eligible | Yes | Core Clinical Event | None (Retained) | Retained (Displayed in Figure 4 Top 30) |
| SG | Hypotension | 56 | 2.35 (1.80-3.07) | 1.73 | Eligible | Yes | Core Clinical Event | None (Retained) | Retained (Displayed in Figure 4 Top 30) |
| SG | Colitis | 44 | 3.61 (2.66-4.89) | 2.45 | Eligible | Yes | Core Clinical Event | None (Retained) | Retained (Not displayed in Figure 4 Top 30) |
| SG | Neutropenic colitis | 43 | 28.61 (20.23-40.45) | 16.04 | Eligible | Yes | Core Clinical Event | None (Retained) | Retained (Not displayed in Figure 4 Top 30) |
| SG | Sepsis | 41 | 2.08 (1.52-2.84) | 1.47 | Eligible | Yes | Core Clinical Event | None (Retained) | Retained (Not displayed in Figure 4 Top 30) |
| SG | Pancytopenia | 34 | 1.73 (1.23-2.43) | 1.21 | Eligible | Yes | Core Clinical Event | None (Retained) | Retained (Not displayed in Figure 4 Top 30) |
| SG | Febrile bone marrow aplasia | 31 | 11.02 (7.57-16.05) | 7.15 | Eligible | Yes | Core Clinical Event | None (Retained) | Retained (Not displayed in Figure 4 Top 30) |
| SG | Neutrophil count abnormal | 26 | 9.50 (6.33-14.27) | 6.11 | Eligible | Yes | Core Clinical Event | None (Retained) | Retained (Not displayed in Figure 4 Top 30) |
| SG | Hepatic failure | 25 | 2.06 (1.39-3.07) | 1.32 | Eligible | Yes | Core Clinical Event | None (Retained) | Retained (Not displayed in Figure 4 Top 30) |
| SG | Liver disorder | 22 | 1.77 (1.16-2.71) | 1.13 | Eligible | Yes | Core Clinical Event | None (Retained) | Retained (Not displayed in Figure 4 Top 30) |
| SG | Cholestasis | 21 | 3.52 (2.27-5.46) | 1.94 | Eligible | Yes | Core Clinical Event | None (Retained) | Retained (Not displayed in Figure 4 Top 30) |
| SG | Haematotoxicity | 19 | 2.43 (1.54-3.83) | 1.39 | Eligible | Yes | Core Clinical Event | None (Retained) | Retained (Not displayed in Figure 4 Top 30) |
| SG | Hyponatraemia | 19 | 1.80 (1.14-2.83) | 1.10 | Eligible | Yes | Core Clinical Event | None (Retained) | Retained (Not displayed in Figure 4 Top 30) |
| SG | Cytopenia | 18 | 2.81 (1.76-4.51) | 1.53 | Eligible | Yes | Core Clinical Event | None (Retained) | Retained (Not displayed in Figure 4 Top 30) |
| SG | Hepatic cytolysis | 17 | 1.72 (1.07-2.79) | 1.04 | Eligible | Yes | Core Clinical Event | None (Retained) | Retained (Not displayed in Figure 4 Top 30) |
| SG | SARS-CoV-2 test positive | 17 | 6.99 (4.26-11.48) | 4.08 | Eligible | Yes | Core Clinical Event | None (Retained) | Retained (Not displayed in Figure 4 Top 30) |
| SG | Neutropenic sepsis | 16 | 3.40 (2.06-5.61) | 1.69 | Eligible | Yes | Core Clinical Event | None (Retained) | Retained (Not displayed in Figure 4 Top 30) |
| SG | Enteritis | 14 | 9.52 (5.47-16.55) | 5.32 | Eligible | Yes | Core Clinical Event | None (Retained) | Retained (Not displayed in Figure 4 Top 30) |
| SG | Blood test abnormal | 13 | 1.89 (1.09-3.27) | 1.03 | Eligible | Yes | Core Clinical Event | None (Retained) | Retained (Not displayed in Figure 4 Top 30) |
| SG | Enterocolitis | 12 | 5.18 (2.89-9.28) | 2.18 | Eligible | Yes | Core Clinical Event | None (Retained) | Retained (Not displayed in Figure 4 Top 30) |
| SG | Therapeutic response decreased | 12 | 3.19 (1.79-5.69) | 1.43 | Eligible | Yes | Core Clinical Event | None (Retained) | Retained (Not displayed in Figure 4 Top 30) |
| SG | Multiple organ dysfunction syndrome | 11 | 1.95 (1.07-3.55) | 1.00 | Eligible | Yes | Core Clinical Event | None (Retained) | Retained (Not displayed in Figure 4 Top 30) |
| T-DM1 | Platelet count decreased | 252 | 4.30 (3.77-4.90) | 3.47 | Eligible | Yes | Core Clinical Event | None (Retained) | Retained (Displayed in Figure 4 Top 30) |
| T-DM1 | Thrombocytopenia | 225 | 3.62 (3.15-4.15) | 2.95 | Eligible | Yes | Core Clinical Event | None (Retained) | Retained (Displayed in Figure 4 Top 30) |
| T-DM1 | Pyrexia | 160 | 1.44 (1.23-1.69) | 1.23 | Eligible | Yes | Core Clinical Event | None (Retained) | Retained (Displayed in Figure 4 Top 30) |
| T-DM1 | Neuropathy peripheral | 135 | 1.85 (1.55-2.20) | 1.54 | Eligible | Yes | Core Clinical Event | None (Retained) | Retained (Displayed in Figure 4 Top 30) |
| T-DM1 | Pneumonia | 119 | 1.80 (1.50-2.17) | 1.49 | Eligible | Yes | Core Clinical Event | None (Retained) | Retained (Displayed in Figure 4 Top 30) |
| T-DM1 | Aspartate aminotransferase increased | 102 | 3.48 (2.85-4.26) | 2.70 | Eligible | Yes | Core Clinical Event | None (Retained) | Retained (Displayed in Figure 4 Top 30) |
| T-DM1 | Alanine aminotransferase increased | 82 | 2.56 (2.05-3.20) | 1.99 | Eligible | Yes | Core Clinical Event | None (Retained) | Retained (Displayed in Figure 4 Top 30) |
| T-DM1 | Interstitial lung disease | 61 | 1.39 (1.08-1.80) | 1.10 | Eligible | Yes | Core Clinical Event | None (Retained) | Retained (Displayed in Figure 4 Top 30) |
| T-DM1 | Hepatic function abnormal | 57 | 2.31 (1.77-3.02) | 1.74 | Eligible | Yes | Core Clinical Event | None (Retained) | Retained (Displayed in Figure 4 Top 30) |
| T-DM1 | Hepatic cirrhosis | 55 | 10.37 (7.79-13.79) | 7.14 | Eligible | Yes | Core Clinical Event | None (Retained) | Retained (Displayed in Figure 4 Top 30) |
| T-DM1 | Pneumonitis | 52 | 1.52 (1.15-2.01) | 1.18 | Eligible | Yes | Core Clinical Event | None (Retained) | Retained (Not displayed in Figure 4 Top 30) |
| T-DM1 | Blood bilirubin increased | 48 | 3.52 (2.63-4.72) | 2.45 | Eligible | Yes | Core Clinical Event | None (Retained) | Retained (Not displayed in Figure 4 Top 30) |
| T-DM1 | Ejection fraction decreased | 48 | 1.58 (1.19-2.11) | 1.21 | Eligible | Yes | Core Clinical Event | None (Retained) | Retained (Not displayed in Figure 4 Top 30) |
| T-DM1 | Cardiac failure | 45 | 1.48 (1.10-2.00) | 1.13 | Eligible | Yes | Core Clinical Event | None (Retained) | Retained (Not displayed in Figure 4 Top 30) |
| T-DM1 | Hepatic enzyme increased | 42 | 1.95 (1.43-2.65) | 1.43 | Eligible | Yes | Core Clinical Event | None (Retained) | Retained (Not displayed in Figure 4 Top 30) |
| T-DM1 | Portal hypertension | 41 | 19.93 (14.05-28.27) | 11.56 | Eligible | Yes | Core Clinical Event | None (Retained) | Retained (Not displayed in Figure 4 Top 30) |
| T-DM1 | Liver disorder | 40 | 2.58 (1.88-3.54) | 1.81 | Eligible | Yes | Core Clinical Event | None (Retained) | Retained (Not displayed in Figure 4 Top 30) |
| T-DM1 | Vision blurred | 40 | 2.05 (1.50-2.82) | 1.48 | Eligible | Yes | Core Clinical Event | None (Retained) | Retained (Not displayed in Figure 4 Top 30) |
| T-DM1 | Hepatotoxicity | 38 | 2.18 (1.58-3.02) | 1.55 | Eligible | Yes | Core Clinical Event | None (Retained) | Retained (Not displayed in Figure 4 Top 30) |
| T-DM1 | Infusion related reaction | 38 | 2.07 (1.50-2.87) | 1.48 | Eligible | Yes | Core Clinical Event | None (Retained) | Retained (Not displayed in Figure 4 Top 30) |
| T-DM1 | Nodular regenerative hyperplasia | 36 | 58.29 (37.30-91.07) | 21.81 | Eligible | Yes | Core Clinical Event | None (Retained) | Retained (Not displayed in Figure 4 Top 30) |
| T-DM1 | Gingival bleeding | 32 | 7.41 (5.14-10.69) | 4.83 | Eligible | Yes | Core Clinical Event | None (Retained) | Retained (Not displayed in Figure 4 Top 30) |
| T-DM1 | Cardiotoxicity | 29 | 1.53 (1.06-2.21) | 1.08 | Eligible | Yes | Core Clinical Event | None (Retained) | Retained (Not displayed in Figure 4 Top 30) |
| T-DM1 | Cerebral haemorrhage | 29 | 6.60 (4.50-9.68) | 4.13 | Eligible | Yes | Core Clinical Event | None (Retained) | Retained (Not displayed in Figure 4 Top 30) |
| T-DM1 | Drug-induced liver injury | 26 | 2.04 (1.38-3.02) | 1.36 | Eligible | Yes | Core Clinical Event | None (Retained) | Retained (Not displayed in Figure 4 Top 30) |
| T-DM1 | Haemorrhage | 26 | 1.94 (1.32-2.88) | 1.31 | Eligible | Yes | Core Clinical Event | None (Retained) | Retained (Not displayed in Figure 4 Top 30) |
| T-DM1 | Hepatic failure | 26 | 1.68 (1.14-2.48) | 1.15 | Eligible | Yes | Core Clinical Event | None (Retained) | Retained (Not displayed in Figure 4 Top 30) |
| T-DM1 | Splenomegaly | 25 | 16.14 (10.42-25.00) | 9.05 | Eligible | Yes | Core Clinical Event | None (Retained) | Retained (Not displayed in Figure 4 Top 30) |
| T-DM1 | Pericardial effusion | 22 | 1.99 (1.30-3.05) | 1.29 | Eligible | Yes | Core Clinical Event | None (Retained) | Retained (Not displayed in Figure 4 Top 30) |
| T-DM1 | Liver function test abnormal | 21 | 3.11 (2.00-4.82) | 1.81 | Eligible | Yes | Core Clinical Event | None (Retained) | Retained (Not displayed in Figure 4 Top 30) |
| T-DM1 | Gastrointestinal haemorrhage | 20 | 3.43 (2.19-5.39) | 1.93 | Eligible | Yes | Core Clinical Event | None (Retained) | Retained (Not displayed in Figure 4 Top 30) |
| T-DM1 | Hyperbilirubinaemia | 20 | 3.54 (2.26-5.56) | 1.98 | Eligible | Yes | Core Clinical Event | None (Retained) | Retained (Not displayed in Figure 4 Top 30) |
| T-DM1 | Blood potassium decreased | 18 | 1.66 (1.04-2.66) | 1.06 | Eligible | Yes | Core Clinical Event | None (Retained) | Retained (Not displayed in Figure 4 Top 30) |
| T-DM1 | Brain oedema | 18 | 4.24 (2.63-6.84) | 2.20 | Eligible | Yes | Core Clinical Event | None (Retained) | Retained (Not displayed in Figure 4 Top 30) |
| T-DM1 | Jaundice | 18 | 1.98 (1.24-3.17) | 1.22 | Eligible | Yes | Core Clinical Event | None (Retained) | Retained (Not displayed in Figure 4 Top 30) |
| T-DM1 | Varices oesophageal | 18 | 19.65 (11.62-33.24) | 9.69 | Eligible | Yes | Core Clinical Event | None (Retained) | Retained (Not displayed in Figure 4 Top 30) |
| T-DM1 | Hepatitis | 17 | 1.95 (1.20-3.15) | 1.19 | Eligible | Yes | Core Clinical Event | None (Retained) | Retained (Not displayed in Figure 4 Top 30) |
| T-DM1 | Pulmonary arterial hypertension | 16 | 8.39 (4.99-14.12) | 4.46 | Eligible | Yes | Core Clinical Event | None (Retained) | Retained (Not displayed in Figure 4 Top 30) |
| T-DM1 | Hepatopulmonary syndrome | 15 | 126.84 (53.75-299.34) | 21.73 | Eligible | Yes | Core Clinical Event | None (Retained) | Retained (Not displayed in Figure 4 Top 30) |
| T-DM1 | Spider naevus | 15 | 338.25 (97.88-1168.87) | 25.73 | Eligible | Yes | Core Clinical Event | None (Retained) | Retained (Not displayed in Figure 4 Top 30) |
| T-DM1 | Cholestasis | 14 | 1.81 (1.06-3.08) | 1.07 | Eligible | Yes | Core Clinical Event | None (Retained) | Retained (Not displayed in Figure 4 Top 30) |
| T-DM1 | Neuralgia | 14 | 2.05 (1.20-3.49) | 1.17 | Eligible | Yes | Core Clinical Event | None (Retained) | Retained (Not displayed in Figure 4 Top 30) |
| T-DM1 | Pancreatitis | 14 | 2.95 (1.72-5.04) | 1.53 | Eligible | Yes | Core Clinical Event | None (Retained) | Retained (Not displayed in Figure 4 Top 30) |
| T-DM1 | Pulmonary fibrosis | 14 | 2.72 (1.59-4.64) | 1.44 | Eligible | Yes | Core Clinical Event | None (Retained) | Retained (Not displayed in Figure 4 Top 30) |
| T-DM1 | Telangiectasia | 14 | 26.30 (14.17-48.80) | 10.91 | Eligible | Yes | Core Clinical Event | None (Retained) | Retained (Not displayed in Figure 4 Top 30) |
| T-DM1 | Venoocclusive liver disease | 14 | 25.59 (13.82-47.37) | 10.73 | Eligible | Yes | Core Clinical Event | None (Retained) | Retained (Not displayed in Figure 4 Top 30) |
| T-DM1 | Acute respiratory distress syndrome | 13 | 2.73 (1.56-4.75) | 1.41 | Eligible | Yes | Core Clinical Event | None (Retained) | Retained (Not displayed in Figure 4 Top 30) |
| T-DM1 | Cerebral disorder | 13 | 4.70 (2.67-8.25) | 2.09 | Eligible | Yes | Core Clinical Event | None (Retained) | Retained (Not displayed in Figure 4 Top 30) |
| T-DM1 | Hypercalcaemia | 13 | 2.44 (1.40-4.24) | 1.30 | Eligible | Yes | Core Clinical Event | None (Retained) | Retained (Not displayed in Figure 4 Top 30) |
| T-DM1 | Non-cirrhotic portal hypertension | 13 | 46.26 (22.83-93.73) | 14.37 | Eligible | Yes | Core Clinical Event | None (Retained) | Retained (Not displayed in Figure 4 Top 30) |
| T-DM1 | Rectal haemorrhage | 13 | 1.95 (1.12-3.39) | 1.11 | Eligible | Yes | Core Clinical Event | None (Retained) | Retained (Not displayed in Figure 4 Top 30) |
| T-DM1 | Cholecystitis | 12 | 4.43 (2.47-7.95) | 1.92 | Eligible | Yes | Core Clinical Event | None (Retained) | Retained (Not displayed in Figure 4 Top 30) |
| T-DM1 | Drug resistance | 12 | 2.05 (1.15-3.64) | 1.12 | Eligible | Yes | Core Clinical Event | None (Retained) | Retained (Not displayed in Figure 4 Top 30) |
| T-DM1 | Monocyte count increased | 12 | 6.43 (3.56-11.64) | 2.68 | Eligible | Yes | Core Clinical Event | None (Retained) | Retained (Not displayed in Figure 4 Top 30) |
| T-DM1 | Red cell distribution width increased | 12 | 2.04 (1.15-3.63) | 1.12 | Eligible | Yes | Core Clinical Event | None (Retained) | Retained (Not displayed in Figure 4 Top 30) |
| T-DM1 | Encephalopathy | 11 | 4.50 (2.44-8.30) | 1.86 | Eligible | Yes | Core Clinical Event | None (Retained) | Retained (Not displayed in Figure 4 Top 30) |
| T-DM1 | Epilepsy | 11 | 2.31 (1.27-4.22) | 1.19 | Eligible | Yes | Core Clinical Event | None (Retained) | Retained (Not displayed in Figure 4 Top 30) |
| T-DM1 | Haemoptysis | 11 | 3.33 (1.82-6.11) | 1.52 | Eligible | Yes | Core Clinical Event | None (Retained) | Retained (Not displayed in Figure 4 Top 30) |
| T-DM1 | Hepatic encephalopathy | 11 | 6.19 (3.34-11.49) | 2.44 | Eligible | Yes | Core Clinical Event | None (Retained) | Retained (Not displayed in Figure 4 Top 30) |
| T-DM1 | Maternal exposure during pregnancy | 11 | 2.16 (1.18-3.94) | 1.13 | Eligible | Yes | Core Clinical Event | None (Retained) | Retained (Not displayed in Figure 4 Top 30) |
| T-DM1 | Pulmonary toxicity | 11 | 2.22 (1.21-4.04) | 1.15 | Eligible | Yes | Core Clinical Event | None (Retained) | Retained (Not displayed in Figure 4 Top 30) |
| T-DM1 | Anaphylactic shock | 10 | 2.11 (1.12-3.96) | 1.08 | Eligible | Yes | Core Clinical Event | None (Retained) | Retained (Not displayed in Figure 4 Top 30) |
| T-DM1 | Blood albumin decreased | 10 | 3.84 (2.03-7.26) | 1.60 | Eligible | Yes | Core Clinical Event | None (Retained) | Retained (Not displayed in Figure 4 Top 30) |
| T-DM1 | Body temperature increased | 10 | 2.10 (1.12-3.94) | 1.08 | Eligible | Yes | Core Clinical Event | None (Retained) | Retained (Not displayed in Figure 4 Top 30) |
| T-DM1 | Corneal disorder | 10 | 14.69 (7.41-29.12) | 6.50 | Eligible | Yes | Core Clinical Event | None (Retained) | Retained (Not displayed in Figure 4 Top 30) |
| T-DM1 | Haematuria | 10 | 2.82 (1.50-5.32) | 1.31 | Eligible | Yes | Core Clinical Event | None (Retained) | Retained (Not displayed in Figure 4 Top 30) |
| T-DM1 | Mouth haemorrhage | 10 | 4.63 (2.44-8.78) | 1.81 | Eligible | Yes | Core Clinical Event | None (Retained) | Retained (Not displayed in Figure 4 Top 30) |
| T-DM1 | Nervous system disorder | 10 | 2.11 (1.12-3.96) | 1.08 | Eligible | Yes | Core Clinical Event | None (Retained) | Retained (Not displayed in Figure 4 Top 30) |
| T-DM1 | Pulmonary hypertension | 10 | 2.69 (1.43-5.06) | 1.27 | Eligible | Yes | Core Clinical Event | None (Retained) | Retained (Not displayed in Figure 4 Top 30) |
| T-DXd | Nausea | 795 | 1.74 (1.61-1.87) | 1.52 | Eligible | Yes | Core Clinical Event | None (Retained) | Retained (Displayed in Figure 4 Top 30) |
| T-DXd | Interstitial lung disease | 631 | 11.86 (10.83-13.00) | 8.18 | Eligible | Yes | Core Clinical Event | None (Retained) | Retained (Displayed in Figure 4 Top 30) |
| T-DXd | Vomiting | 359 | 1.35 (1.21-1.51) | 1.21 | Eligible | Yes | Core Clinical Event | None (Retained) | Retained (Displayed in Figure 4 Top 30) |
| T-DXd | Pneumonitis | 258 | 5.18 (4.54-5.91) | 4.12 | Eligible | Yes | Core Clinical Event | None (Retained) | Retained (Displayed in Figure 4 Top 30) |
| T-DXd | Pneumonia | 154 | 1.42 (1.21-1.67) | 1.21 | Eligible | Yes | Core Clinical Event | None (Retained) | Retained (Displayed in Figure 4 Top 30) |
| T-DXd | Ejection fraction decreased | 74 | 1.50 (1.19-1.90) | 1.19 | Eligible | Yes | Core Clinical Event | None (Retained) | Retained (Displayed in Figure 4 Top 30) |
| T-DXd | Pulmonary embolism | 72 | 1.46 (1.15-1.85) | 1.16 | Eligible | Yes | Core Clinical Event | None (Retained) | Retained (Displayed in Figure 4 Top 30) |
| T-DXd | Infection | 71 | 1.47 (1.16-1.87) | 1.16 | Eligible | Yes | Core Clinical Event | None (Retained) | Retained (Displayed in Figure 4 Top 30) |
| T-DXd | Pneumocystis jirovecii pneumonia | 71 | 6.39 (4.97-8.21) | 4.55 | Eligible | Yes | Core Clinical Event | None (Retained) | Retained (Displayed in Figure 4 Top 30) |
| T-DXd | Sepsis | 62 | 1.51 (1.17-1.95) | 1.17 | Eligible | Yes | Core Clinical Event | None (Retained) | Retained (Displayed in Figure 4 Top 30) |
| T-DXd | Pancytopenia | 54 | 1.32 (1.01-1.73) | 1.02 | Eligible | Yes | Core Clinical Event | None (Retained) | Retained (Not displayed in Figure 4 Top 30) |
| T-DXd | Pulmonary toxicity | 52 | 7.30 (5.43-9.82) | 4.91 | Eligible | Yes | Core Clinical Event | None (Retained) | Retained (Not displayed in Figure 4 Top 30) |
| T-DXd | Hepatic failure | 51 | 2.06 (1.55-2.73) | 1.50 | Eligible | Yes | Core Clinical Event | None (Retained) | Retained (Not displayed in Figure 4 Top 30) |
| T-DXd | Hepatic cytolysis | 46 | 2.31 (1.71-3.11) | 1.64 | Eligible | Yes | Core Clinical Event | None (Retained) | Retained (Not displayed in Figure 4 Top 30) |
| T-DXd | Blood bilirubin increased | 33 | 1.44 (1.02-2.04) | 1.01 | Eligible | Yes | Core Clinical Event | None (Retained) | Retained (Not displayed in Figure 4 Top 30) |
| T-DXd | Cholestasis | 30 | 2.44 (1.69-3.53) | 1.58 | Eligible | Yes | Core Clinical Event | None (Retained) | Retained (Not displayed in Figure 4 Top 30) |
| T-DXd | Pulmonary fibrosis | 27 | 3.31 (2.24-4.91) | 2.07 | Eligible | Yes | Core Clinical Event | None (Retained) | Retained (Not displayed in Figure 4 Top 30) |
| T-DXd | Hepatic cirrhosis | 25 | 2.62 (1.75-3.93) | 1.61 | Eligible | Yes | Core Clinical Event | None (Retained) | Retained (Not displayed in Figure 4 Top 30) |
| T-DXd | Pneumonia bacterial | 21 | 8.38 (5.24-13.41) | 4.67 | Eligible | Yes | Core Clinical Event | None (Retained) | Retained (Not displayed in Figure 4 Top 30) |
| T-DXd | Acute respiratory distress syndrome | 19 | 2.47 (1.55-3.93) | 1.41 | Eligible | Yes | Core Clinical Event | None (Retained) | Retained (Not displayed in Figure 4 Top 30) |
| T-DXd | Acute respiratory failure | 18 | 2.67 (1.66-4.30) | 1.48 | Eligible | Yes | Core Clinical Event | None (Retained) | Retained (Not displayed in Figure 4 Top 30) |
| T-DXd | Brain oedema | 18 | 2.58 (1.60-4.15) | 1.43 | Eligible | Yes | Core Clinical Event | None (Retained) | Retained (Not displayed in Figure 4 Top 30) |
| T-DXd | Pneumothorax | 18 | 1.93 (1.20-3.10) | 1.13 | Eligible | Yes | Core Clinical Event | None (Retained) | Retained (Not displayed in Figure 4 Top 30) |
| T-DXd | Therapeutic response unexpected | 18 | 2.97 (1.84-4.79) | 1.63 | Eligible | Yes | Core Clinical Event | None (Retained) | Retained (Not displayed in Figure 4 Top 30) |
| T-DXd | Hypersomnia | 17 | 2.00 (1.23-3.26) | 1.14 | Eligible | Yes | Core Clinical Event | None (Retained) | Retained (Not displayed in Figure 4 Top 30) |
| T-DXd | Clostridium difficile infection | 15 | 2.20 (1.31-3.70) | 1.18 | Eligible | Yes | Core Clinical Event | None (Retained) | Retained (Not displayed in Figure 4 Top 30) |
| T-DXd | Chromaturia | 14 | 2.13 (1.24-3.64) | 1.12 | Eligible | Yes | Core Clinical Event | None (Retained) | Retained (Not displayed in Figure 4 Top 30) |
| T-DXd | Dysarthria | 14 | 2.52 (1.47-4.33) | 1.29 | Eligible | Yes | Core Clinical Event | None (Retained) | Retained (Not displayed in Figure 4 Top 30) |
| T-DXd | Bacterial infection | 13 | 3.05 (1.74-5.36) | 1.47 | Eligible | Yes | Core Clinical Event | None (Retained) | Retained (Not displayed in Figure 4 Top 30) |
| T-DXd | Clostridium difficile colitis | 13 | 3.49 (1.98-6.15) | 1.67 | Eligible | Yes | Core Clinical Event | None (Retained) | Retained (Not displayed in Figure 4 Top 30) |
| T-DXd | Haematological infection | 13 | 10.90 (5.91-20.10) | 4.93 | Eligible | Yes | Core Clinical Event | None (Retained) | Retained (Not displayed in Figure 4 Top 30) |
| T-DXd | COVID-19 pneumonia | 12 | 4.69 (2.58-8.53) | 2.20 | Eligible | Yes | Core Clinical Event | None (Retained) | Retained (Not displayed in Figure 4 Top 30) |
| T-DXd | Pulmonary thrombosis | 12 | 2.04 (1.14-3.65) | 1.03 | Eligible | Yes | Core Clinical Event | None (Retained) | Retained (Not displayed in Figure 4 Top 30) |
| T-DXd | Enterocolitis | 10 | 2.03 (1.08-3.83) | 0.96 | Eligible | Yes | Core Clinical Event | None (Retained) | Retained (Not displayed in Figure 4 Top 30) |
| T-DXd | Haemolytic anaemia | 10 | 3.02 (1.59-5.74) | 1.29 | Eligible | Yes | Core Clinical Event | None (Retained) | Retained (Not displayed in Figure 4 Top 30) |
| T-DXd | Keratitis | 10 | 4.06 (2.12-7.79) | 1.69 | Eligible | Yes | Core Clinical Event | None (Retained) | Retained (Not displayed in Figure 4 Top 30) |
| T-DXd | Ocular toxicity | 10 | 22.81 (10.53-49.44) | 6.74 | Eligible | Yes | Core Clinical Event | None (Retained) | Retained (Not displayed in Figure 4 Top 30) |
| T-DXd | Pneumocystis jirovecii infection | 10 | 9.12 (4.60-18.11) | 3.91 | Eligible | Yes | Core Clinical Event | None (Retained) | Retained (Not displayed in Figure 4 Top 30) |
| T-DXd | Treatment noncompliance | 10 | 2.60 (1.37-4.92) | 1.14 | Eligible | Yes | Core Clinical Event | None (Retained) | Retained (Not displayed in Figure 4 Top 30) |
| SG | Disease progression | 920 | 14.08 (13.00-15.26) | 8.75 | Eligible | No | Disease/Context Related | Term reflects underlying disease progression or oncological context | Excluded from main panel |
| SG | Death | 400 | 2.19 (1.97-2.43) | 1.84 | Eligible | No | Nonspecific | Term lacks clear clinical definition or specificity | Excluded from main panel |
| SG | Inappropriate schedule of product administration | 306 | 23.22 (20.38-26.45) | 15.49 | Eligible | No | Administration Related | Term reflects drug administration procedures or device issues | Excluded from main panel |
| SG | Off label use | 208 | 1.60 (1.39-1.85) | 1.37 | Eligible | No | Administration Related | Term reflects drug administration procedures or device issues | Excluded from main panel |
| SG | Asthenia | 193 | 1.69 (1.46-1.95) | 1.43 | Eligible | No | Nonspecific | Term lacks clear clinical definition or specificity | Excluded from main panel |
| SG | Weight fluctuation | 161 | 91.76 (73.49-114.59) | 37.47 | Eligible | No | Nonspecific | Term lacks clear clinical definition or specificity | Excluded from main panel |
| SG | Triple negative breast cancer | 150 | 107.50 (84.60-136.60) | 40.12 | Eligible | No | Disease/Context Related | Term reflects underlying disease progression or oncological context | Excluded from main panel |
| SG | Weight decreased | 134 | 2.17 (1.82-2.58) | 1.77 | Eligible | No | Nonspecific | Term lacks clear clinical definition or specificity | Excluded from main panel |
| SG | Malaise | 107 | 1.29 (1.07-1.57) | 1.08 | Eligible | No | Nonspecific | Term lacks clear clinical definition or specificity | Excluded from main panel |
| SG | Myelosuppression | 96 | 1.51 (1.23-1.85) | 1.23 | Eligible | No | Nonspecific | Term lacks clear clinical definition or specificity | Excluded from main panel |
| SG | Weight increased | 91 | 3.69 (2.98-4.56) | 2.82 | Eligible | No | Nonspecific | Term lacks clear clinical definition or specificity | Excluded from main panel |
| SG | COVID-19 | 61 | 2.16 (1.67-2.79) | 1.63 | Eligible | No | Nonspecific | Term lacks clear clinical definition or specificity | Excluded from main panel |
| SG | Metastases to central nervous system | 60 | 2.25 (1.74-2.92) | 1.68 | Eligible | No | Disease/Context Related | Term reflects underlying disease progression or oncological context | Excluded from main panel |
| SG | General physical health deterioration | 56 | 1.53 (1.18-2.00) | 1.18 | Eligible | No | Nonspecific | Term lacks clear clinical definition or specificity | Excluded from main panel |
| SG | Hypertension | 56 | 1.75 (1.34-2.29) | 1.33 | Eligible | No | Nonspecific | Term lacks clear clinical definition or specificity | Excluded from main panel |
| SG | Septic shock | 42 | 5.75 (4.20-7.88) | 4.09 | Eligible | No | Nonspecific | Term lacks clear clinical definition or specificity | Excluded from main panel |
| SG | Ill-defined disorder | 30 | 3.56 (2.47-5.14) | 2.19 | Eligible | No | Nonspecific | Term lacks clear clinical definition or specificity | Excluded from main panel |
| SG | General physical condition abnormal | 28 | 18.97 (12.59-28.60) | 10.97 | Eligible | No | Nonspecific | Term lacks clear clinical definition or specificity | Excluded from main panel |
| SG | Intentional dose omission | 26 | 11.86 (7.86-17.89) | 7.39 | Eligible | No | Administration Related | Term reflects drug administration procedures or device issues | Excluded from main panel |
| SG | Oxygen saturation decreased | 24 | 2.41 (1.60-3.61) | 1.48 | Eligible | No | Nonspecific | Term lacks clear clinical definition or specificity | Excluded from main panel |
| SG | Agranulocytosis | 21 | 1.67 (1.08-2.58) | 1.07 | Eligible | No | Nonspecific | Term lacks clear clinical definition or specificity | Excluded from main panel |
| SG | Poor venous access | 15 | 13.36 (7.75-23.03) | 7.25 | Eligible | No | Administration Related | Term reflects drug administration procedures or device issues | Excluded from main panel |
| SG | Productive cough | 15 | 1.97 (1.18-3.30) | 1.11 | Eligible | No | Administration Related | Term reflects drug administration procedures or device issues | Excluded from main panel |
| SG | Metastases to meninges | 14 | 3.76 (2.20-6.43) | 1.74 | Eligible | No | Disease/Context Related | Term reflects underlying disease progression or oncological context | Excluded from main panel |
| SG | Product preparation error | 13 | 20.41 (11.14-37.39) | 9.62 | Eligible | No | Administration Related | Term reflects drug administration procedures or device issues | Excluded from main panel |
| SG | Cell death | 12 | 7.45 (4.13-13.45) | 3.74 | Eligible | No | Nonspecific | Term lacks clear clinical definition or specificity | Excluded from main panel |
| SG | Complication associated with device | 12 | 22.52 (11.92-42.54) | 10.05 | Eligible | No | Administration Related | Term reflects drug administration procedures or device issues | Excluded from main panel |
| SG | Product storage error | 11 | 6.16 (3.34-11.37) | 2.58 | Eligible | No | Administration Related | Term reflects drug administration procedures or device issues | Excluded from main panel |
| T-DM1 | Disease progression | 260 | 2.16 (1.90-2.46) | 1.84 | Eligible | No | Disease/Context Related | Term reflects underlying disease progression or oncological context | Excluded from main panel |
| T-DM1 | Myelosuppression | 158 | 1.99 (1.69-2.34) | 1.67 | Eligible | No | Nonspecific | Term lacks clear clinical definition or specificity | Excluded from main panel |
| T-DM1 | Drug ineffective | 140 | 1.66 (1.40-1.97) | 1.40 | Eligible | No | Nonspecific | Term lacks clear clinical definition or specificity | Excluded from main panel |
| T-DM1 | Epistaxis | 131 | 3.51 (2.94-4.20) | 2.78 | Eligible | No | Nonspecific | Term lacks clear clinical definition or specificity | Excluded from main panel |
| T-DM1 | Metastases to central nervous system | 120 | 3.67 (3.05-4.42) | 2.88 | Eligible | No | Disease/Context Related | Term reflects underlying disease progression or oncological context | Excluded from main panel |
| T-DM1 | Myalgia | 62 | 1.44 (1.12-1.86) | 1.14 | Eligible | No | Nonspecific | Term lacks clear clinical definition or specificity | Excluded from main panel |
| T-DM1 | Hypoaesthesia | 59 | 1.54 (1.19-1.99) | 1.21 | Eligible | No | Nonspecific | Term lacks clear clinical definition or specificity | Excluded from main panel |
| T-DM1 | Chills | 56 | 1.58 (1.21-2.06) | 1.23 | Eligible | No | Nonspecific | Term lacks clear clinical definition or specificity | Excluded from main panel |
| T-DM1 | Blood alkaline phosphatase increased | 45 | 3.56 (2.63-4.81) | 2.44 | Eligible | No | Nonspecific | Term lacks clear clinical definition or specificity | Excluded from main panel |
| T-DM1 | Hypokalaemia | 45 | 1.93 (1.43-2.60) | 1.43 | Eligible | No | Nonspecific | Term lacks clear clinical definition or specificity | Excluded from main panel |
| T-DM1 | Seizure | 38 | 2.38 (1.72-3.29) | 1.67 | Eligible | No | Nonspecific | Term lacks clear clinical definition or specificity | Excluded from main panel |
| T-DM1 | Dry mouth | 32 | 1.43 (1.01-2.03) | 1.04 | Eligible | No | Nonspecific | Term lacks clear clinical definition or specificity | Excluded from main panel |
| T-DM1 | Transaminases increased | 32 | 2.11 (1.48-3.01) | 1.46 | Eligible | No | Nonspecific | Term lacks clear clinical definition or specificity | Excluded from main panel |
| T-DM1 | Radiation necrosis | 31 | 25.67 (16.96-38.85) | 13.16 | Eligible | No | Radiation Related | Term reflects concomitant radiation therapy effects | Excluded from main panel |
| T-DM1 | Ill-defined disorder | 29 | 2.69 (1.85-3.90) | 1.75 | Eligible | No | Nonspecific | Term lacks clear clinical definition or specificity | Excluded from main panel |
| T-DM1 | Respiratory failure | 29 | 1.63 (1.13-2.36) | 1.15 | Eligible | No | Nonspecific | Term lacks clear clinical definition or specificity | Excluded from main panel |
| T-DM1 | Neoplasm malignant | 28 | 3.00 (2.05-4.38) | 1.90 | Eligible | No | Disease/Context Related | Term reflects underlying disease progression or oncological context | Excluded from main panel |
| T-DM1 | Gamma-glutamyltransferase increased | 27 | 1.92 (1.31-2.82) | 1.30 | Eligible | No | Nonspecific | Term lacks clear clinical definition or specificity | Excluded from main panel |
| T-DM1 | Hyponatraemia | 27 | 2.02 (1.38-2.97) | 1.36 | Eligible | No | Nonspecific | Term lacks clear clinical definition or specificity | Excluded from main panel |
| T-DM1 | Contusion | 22 | 1.56 (1.02-2.38) | 1.05 | Eligible | No | Nonspecific | Term lacks clear clinical definition or specificity | Excluded from main panel |
| T-DM1 | Blood lactate dehydrogenase increased | 21 | 2.84 (1.83-4.40) | 1.69 | Eligible | No | Nonspecific | Term lacks clear clinical definition or specificity | Excluded from main panel |
| T-DM1 | Lymphocyte count decreased | 20 | 2.09 (1.34-3.26) | 1.31 | Eligible | No | Nonspecific | Term lacks clear clinical definition or specificity | Excluded from main panel |
| T-DM1 | Recurrent cancer | 17 | 5.04 (3.08-8.26) | 2.51 | Eligible | No | Disease/Context Related | Term reflects underlying disease progression or oncological context | Excluded from main panel |
| T-DM1 | Tumour haemorrhage | 16 | 11.27 (6.63-19.16) | 6.12 | Eligible | No | Disease/Context Related | Term reflects underlying disease progression or oncological context | Excluded from main panel |
| T-DM1 | Device related infection | 15 | 2.64 (1.57-4.42) | 1.44 | Eligible | No | Administration Related | Term reflects drug administration procedures or device issues | Excluded from main panel |
| T-DM1 | Extravasation | 13 | 10.10 (5.63-18.11) | 5.07 | Eligible | No | Administration Related | Term reflects drug administration procedures or device issues | Excluded from main panel |
| T-DM1 | Radiation pneumonitis | 13 | 5.60 (3.18-9.86) | 2.43 | Eligible | No | Radiation Related | Term reflects concomitant radiation therapy effects | Excluded from main panel |
| T-DM1 | Brain neoplasm | 12 | 2.72 (1.53-4.85) | 1.37 | Eligible | No | Disease/Context Related | Term reflects underlying disease progression or oncological context | Excluded from main panel |
| T-DXd | Death | 700 | 1.82 (1.68-1.97) | 1.59 | Eligible | No | Nonspecific | Term lacks clear clinical definition or specificity | Excluded from main panel |
| T-DXd | Disease progression | 529 | 2.82 (2.57-3.09) | 2.38 | Eligible | No | Disease/Context Related | Term reflects underlying disease progression or oncological context | Excluded from main panel |
| T-DXd | Myelosuppression | 255 | 1.99 (1.75-2.26) | 1.70 | Eligible | No | Nonspecific | Term lacks clear clinical definition or specificity | Excluded from main panel |
| T-DXd | Intentional product use issue | 198 | 11.70 (9.97-13.72) | 8.03 | Eligible | No | Administration Related | Term reflects drug administration procedures or device issues | Excluded from main panel |
| T-DXd | Toxicity to various agents | 161 | 5.43 (4.60-6.41) | 4.21 | Eligible | No | Nonspecific | Term lacks clear clinical definition or specificity | Excluded from main panel |
| T-DXd | Weight decreased | 157 | 1.19 (1.02-1.40) | 1.03 | Eligible | No | Nonspecific | Term lacks clear clinical definition or specificity | Excluded from main panel |
| T-DXd | Metastases to central nervous system | 124 | 2.28 (1.90-2.74) | 1.85 | Eligible | No | Disease/Context Related | Term reflects underlying disease progression or oncological context | Excluded from main panel |
| T-DXd | General physical health deterioration | 112 | 1.49 (1.23-1.80) | 1.23 | Eligible | No | Nonspecific | Term lacks clear clinical definition or specificity | Excluded from main panel |
| T-DXd | No adverse event | 102 | 3.31 (2.70-4.06) | 2.59 | Eligible | No | Nonspecific | Term lacks clear clinical definition or specificity | Excluded from main panel |
| T-DXd | Pleural effusion | 99 | 1.30 (1.06-1.59) | 1.07 | Eligible | No | Nonspecific | Term lacks clear clinical definition or specificity | Excluded from main panel |
| T-DXd | COVID-19 | 97 | 1.66 (1.35-2.03) | 1.34 | Eligible | No | Nonspecific | Term lacks clear clinical definition or specificity | Excluded from main panel |
| T-DXd | Inappropriate schedule of product administration | 87 | 2.45 (1.97-3.04) | 1.90 | Eligible | No | Administration Related | Term reflects drug administration procedures or device issues | Excluded from main panel |
| T-DXd | Ascites | 79 | 2.38 (1.89-2.99) | 1.83 | Eligible | No | Nonspecific | Term lacks clear clinical definition or specificity | Excluded from main panel |
| T-DXd | Lung disorder | 68 | 2.83 (2.21-3.63) | 2.12 | Eligible | No | Nonspecific | Term lacks clear clinical definition or specificity | Excluded from main panel |
| T-DXd | Respiratory failure | 63 | 2.23 (1.72-2.87) | 1.67 | Eligible | No | Nonspecific | Term lacks clear clinical definition or specificity | Excluded from main panel |
| T-DXd | Underdose | 60 | 7.04 (5.35-9.27) | 4.85 | Eligible | No | Administration Related | Term reflects drug administration procedures or device issues | Excluded from main panel |
| T-DXd | Gastrointestinal disorder | 57 | 1.64 (1.25-2.13) | 1.24 | Eligible | No | Nonspecific | Term lacks clear clinical definition or specificity | Excluded from main panel |
| T-DXd | Incorrect dose administered | 52 | 3.78 (2.84-5.02) | 2.71 | Eligible | No | Administration Related | Term reflects drug administration procedures or device issues | Excluded from main panel |
| T-DXd | Adverse drug reaction | 48 | 3.81 (2.83-5.12) | 2.70 | Eligible | No | Nonspecific | Term lacks clear clinical definition or specificity | Excluded from main panel |
| T-DXd | Seizure | 41 | 1.56 (1.14-2.14) | 1.13 | Eligible | No | Nonspecific | Term lacks clear clinical definition or specificity | Excluded from main panel |
| T-DXd | Therapy change | 38 | 5.47 (3.90-7.69) | 3.67 | Eligible | No | Administration Related | Term reflects drug administration procedures or device issues | Excluded from main panel |
| T-DXd | Septic shock | 36 | 2.31 (1.65-3.23) | 1.57 | Eligible | No | Nonspecific | Term lacks clear clinical definition or specificity | Excluded from main panel |
| T-DXd | Adverse event | 33 | 3.11 (2.18-4.43) | 2.04 | Eligible | No | Nonspecific | Term lacks clear clinical definition or specificity | Excluded from main panel |
| T-DXd | Hyponatraemia | 32 | 1.46 (1.03-2.08) | 1.02 | Eligible | No | Nonspecific | Term lacks clear clinical definition or specificity | Excluded from main panel |
| T-DXd | Lung opacity | 29 | 9.25 (6.18-13.85) | 5.40 | Eligible | No | Nonspecific | Term lacks clear clinical definition or specificity | Excluded from main panel |
| T-DXd | Unevaluable event | 28 | 2.99 (2.04-4.39) | 1.88 | Eligible | No | Nonspecific | Term lacks clear clinical definition or specificity | Excluded from main panel |
| T-DXd | Gastrointestinal toxicity | 27 | 4.05 (2.73-6.02) | 2.55 | Eligible | No | Nonspecific | Term lacks clear clinical definition or specificity | Excluded from main panel |
| T-DXd | Metastases to meninges | 27 | 3.60 (2.43-5.35) | 2.26 | Eligible | No | Disease/Context Related | Term reflects underlying disease progression or oncological context | Excluded from main panel |
| T-DXd | Haematotoxicity | 25 | 1.53 (1.03-2.29) | 1.01 | Eligible | No | Nonspecific | Term lacks clear clinical definition or specificity | Excluded from main panel |
| T-DXd | Cell death | 23 | 7.39 (4.74-11.53) | 4.31 | Eligible | No | Nonspecific | Term lacks clear clinical definition or specificity | Excluded from main panel |
| T-DXd | Quality of life decreased | 23 | 6.02 (3.89-9.34) | 3.63 | Eligible | No | Nonspecific | Term lacks clear clinical definition or specificity | Excluded from main panel |
| T-DXd | Lung infiltration | 21 | 2.93 (1.88-4.57) | 1.70 | Eligible | No | Nonspecific | Term lacks clear clinical definition or specificity | Excluded from main panel |
| T-DXd | Brain fog | 20 | 2.87 (1.82-4.52) | 1.64 | Eligible | No | Nonspecific | Term lacks clear clinical definition or specificity | Excluded from main panel |
| T-DXd | Product administration error | 20 | 9.14 (5.62-14.84) | 4.93 | Eligible | No | Administration Related | Term reflects drug administration procedures or device issues | Excluded from main panel |
| T-DXd | Respiratory disorder | 20 | 2.55 (1.62-4.01) | 1.47 | Eligible | No | Nonspecific | Term lacks clear clinical definition or specificity | Excluded from main panel |
| T-DXd | Radiation necrosis | 17 | 7.28 (4.34-12.20) | 3.95 | Eligible | No | Radiation Related | Term reflects concomitant radiation therapy effects | Excluded from main panel |
| T-DXd | Bedridden | 15 | 6.70 (3.88-11.57) | 3.54 | Eligible | No | Nonspecific | Term lacks clear clinical definition or specificity | Excluded from main panel |
| T-DXd | Prescribed underdose | 15 | 7.52 (4.33-13.04) | 3.90 | Eligible | No | Administration Related | Term reflects drug administration procedures or device issues | Excluded from main panel |
| T-DXd | Weight abnormal | 15 | 13.40 (7.48-24.01) | 5.92 | Eligible | No | Nonspecific | Term lacks clear clinical definition or specificity | Excluded from main panel |
| T-DXd | Lymphangiosis carcinomatosa | 14 | 3.09 (1.79-5.32) | 1.53 | Eligible | No | Disease/Context Related | Term reflects underlying disease progression or oncological context | Excluded from main panel |
| T-DXd | Radiation pneumonitis | 12 | 3.12 (1.73-5.61) | 1.44 | Eligible | No | Radiation Related | Term reflects concomitant radiation therapy effects | Excluded from main panel |
| T-DXd | Disease complication | 11 | 8.07 (4.22-15.40) | 3.69 | Eligible | No | Disease/Context Related | Term reflects underlying disease progression or oncological context | Excluded from main panel |

**Supplementary Table S2B. Supportive preferred-term signal inventory from secondary analytic populations and statistically ineligible preferred terms.**

Preferred terms in Supplementary Table S2B were not eligible for inclusion in the breast-cancer primary clinically reviewed PT panel because they originated from supportive analytic populations (all-indication, cold-start, or sparse datasets) or did not meet the predefined statistical eligibility criteria in the breast-cancer primary analysis. These terms were therefore not considered for Figure 4. Findings from cold-start and sparse-reporting analyses are descriptive and hypothesis-generating and should not be interpreted as head-to-head safety comparisons with the three core ADCs. ADC, antibody–drug conjugate; CI, confidence interval; EB05, lower 5% empirical Bayes bound; PT, Preferred Term; ROR, reporting odds ratio; SG, sacituzumab govitecan; T-DM1, trastuzumab emtansine; T-DXd, trastuzumab deruxtecan; Dato-DXd, datopotamab deruxtecan.

| **Drug** | **Preferred Term** | **Analytic Population** | **Report Count (N)** | **ROR (95% CI)** | **EB05** | **Overall Statistical Eligibility** |
| --- | --- | --- | --- | --- | --- | --- |
| SG | Disease progression | all_indication_supportive | 1468 | 74.63 (70.19-79.34) | 49.86 | Eligible |
| SG | Death | all_indication_supportive | 686 | 3.65 (3.37-3.96) | 3.07 | Eligible |
| SG | Diarrhoea | all_indication_supportive | 583 | 4.12 (3.78-4.49) | 3.48 | Eligible |
| SG | Neutropenia | all_indication_supportive | 519 | 17.70 (16.16-19.39) | 14.76 | Eligible |
| SG | Inappropriate schedule of product administration | all_indication_supportive | 315 | 6.89 (6.15-7.72) | 5.89 | Eligible |
| SG | Fatigue | all_indication_supportive | 281 | 1.51 (1.34-1.70) | 1.34 | Eligible |
| SG | Nausea | all_indication_supportive | 264 | 1.50 (1.32-1.70) | 1.33 | Eligible |
| SG | Off label use | all_indication_supportive | 255 | 1.08 (0.95-1.23) | 0.97 | Ineligible |
| SG | Asthenia | all_indication_supportive | 225 | 2.69 (2.36-3.08) | 2.32 | Eligible |
| SG | Febrile neutropenia | all_indication_supportive | 225 | 15.24 (13.33-17.43) | 13.00 | Eligible |
| SG | Alopecia | all_indication_supportive | 189 | 3.88 (3.36-4.49) | 3.29 | Eligible |
| SG | Weight fluctuation | all_indication_supportive | 164 | 64.53 (55.16-75.51) | 53.40 | Eligible |
| SG | Neutrophil count decreased | all_indication_supportive | 161 | 17.35 (14.82-20.31) | 14.65 | Eligible |
| SG | Anaemia | all_indication_supportive | 158 | 3.76 (3.21-4.40) | 3.16 | Eligible |
| SG | Triple negative breast cancer | all_indication_supportive | 151 | 1349.62 (1116.04-1632.08) | 719.67 | Eligible |
| SG | Vomiting | all_indication_supportive | 148 | 1.43 (1.21-1.68) | 1.23 | Eligible |
| SG | Weight decreased | all_indication_supportive | 142 | 2.24 (1.89-2.64) | 1.90 | Eligible |
| SG | Thrombocytopenia | all_indication_supportive | 139 | 5.78 (4.88-6.84) | 4.80 | Eligible |
| SG | Malaise | all_indication_supportive | 122 | 1.15 (0.96-1.38) | 0.98 | Ineligible |
| SG | Myelosuppression | all_indication_supportive | 120 | 14.21 (11.85-17.04) | 11.86 | Eligible |
| SG | Pyrexia | all_indication_supportive | 117 | 1.52 (1.27-1.83) | 1.29 | Eligible |
| SG | Constipation | all_indication_supportive | 108 | 2.12 (1.76-2.57) | 1.77 | Eligible |
| SG | Dyspnoea | all_indication_supportive | 104 | 0.79 (0.65-0.96) | 0.68 | Ineligible |
| SG | White blood cell count decreased | all_indication_supportive | 104 | 4.09 (3.37-4.97) | 3.32 | Eligible |
| SG | Sepsis | all_indication_supportive | 99 | 4.02 (3.29-4.90) | 3.24 | Eligible |
| SG | Weight increased | all_indication_supportive | 91 | 1.82 (1.48-2.23) | 1.49 | Eligible |
| SG | Drug ineffective | all_indication_supportive | 90 | 0.26 (0.21-0.32) | 0.23 | Ineligible |
| SG | Metastases to central nervous system | all_indication_supportive | 90 | 30.10 (24.41-37.11) | 24.41 | Eligible |
| SG | Abdominal pain | all_indication_supportive | 88 | 1.70 (1.38-2.10) | 1.40 | Eligible |
| SG | Decreased appetite | all_indication_supportive | 86 | 1.54 (1.24-1.90) | 1.27 | Eligible |
| SG | Colitis | all_indication_supportive | 82 | 9.17 (7.37-11.40) | 7.43 | Eligible |
| SG | Pain | all_indication_supportive | 78 | 0.51 (0.41-0.64) | 0.43 | Ineligible |
| SG | Hypotension | all_indication_supportive | 76 | 1.70 (1.35-2.13) | 1.38 | Eligible |
| SG | COVID-19 | all_indication_supportive | 70 | 1.38 (1.09-1.75) | 1.12 | Eligible |
| SG | Heart rate increased | all_indication_supportive | 70 | 3.28 (2.59-4.16) | 2.57 | Eligible |
| SG | General physical health deterioration | all_indication_supportive | 69 | 2.59 (2.04-3.29) | 2.05 | Eligible |
| SG | Pneumonitis | all_indication_supportive | 69 | 10.78 (8.50-13.68) | 8.63 | Eligible |
| SG | Septic shock | all_indication_supportive | 67 | 6.94 (5.46-8.84) | 5.41 | Eligible |
| SG | Neutropenic colitis | all_indication_supportive | 64 | 131.11 (101.98-168.55) | 96.81 | Eligible |
| SG | Rash | all_indication_supportive | 63 | 0.60 (0.47-0.78) | 0.50 | Ineligible |
| SG | Pneumonia | all_indication_supportive | 61 | 0.79 (0.62-1.02) | 0.64 | Ineligible |
| SG | Cough | all_indication_supportive | 60 | 0.88 (0.68-1.13) | 0.71 | Ineligible |
| SG | Headache | all_indication_supportive | 60 | 0.40 (0.31-0.52) | 0.33 | Ineligible |
| SG | Metastases to liver | all_indication_supportive | 59 | 14.30 (11.06-18.50) | 11.27 | Eligible |
| SG | Pancytopenia | all_indication_supportive | 59 | 4.90 (3.79-6.33) | 3.69 | Eligible |
| SG | Hypertension | all_indication_supportive | 58 | 1.22 (0.94-1.58) | 0.97 | Ineligible |
| SG | Dizziness | all_indication_supportive | 55 | 0.48 (0.37-0.63) | 0.39 | Ineligible |
| SG | Dehydration | all_indication_supportive | 46 | 1.61 (1.20-2.15) | 1.23 | Eligible |
| SG | Platelet count decreased | all_indication_supportive | 45 | 1.83 (1.37-2.46) | 1.39 | Eligible |
| SG | Neutropenic sepsis | all_indication_supportive | 44 | 28.51 (21.17-38.42) | 21.46 | Eligible |
| SG | Infection | all_indication_supportive | 43 | 1.22 (0.91-1.65) | 0.94 | Ineligible |
| SG | Pleural effusion | all_indication_supportive | 43 | 3.28 (2.43-4.43) | 2.39 | Eligible |
| SG | Leukopenia | all_indication_supportive | 42 | 3.83 (2.83-5.19) | 2.75 | Eligible |
| SG | Acute kidney injury | all_indication_supportive | 41 | 0.96 (0.70-1.30) | 0.74 | Ineligible |
| SG | Mucosal inflammation | all_indication_supportive | 40 | 6.90 (5.05-9.42) | 4.92 | Eligible |
| SG | Abdominal pain upper | all_indication_supportive | 37 | 0.78 (0.56-1.07) | 0.59 | Ineligible |
| SG | Urinary tract infection | all_indication_supportive | 36 | 0.88 (0.63-1.22) | 0.67 | Ineligible |
| SG | Hypokalaemia | all_indication_supportive | 35 | 3.48 (2.50-4.85) | 2.42 | Eligible |
| SG | Metastases to bone | all_indication_supportive | 35 | 8.78 (6.30-12.25) | 6.32 | Eligible |
| SG | Febrile bone marrow aplasia | all_indication_supportive | 34 | 34.41 (24.52-48.30) | 24.81 | Eligible |
| SG | Ill-defined disorder | all_indication_supportive | 34 | 2.26 (1.61-3.17) | 1.62 | Eligible |
| SG | Hepatic failure | all_indication_supportive | 33 | 5.50 (3.91-7.75) | 3.69 | Eligible |
| SG | Interstitial lung disease | all_indication_supportive | 33 | 3.03 (2.15-4.27) | 2.11 | Eligible |
| SG | Back pain | all_indication_supportive | 32 | 0.59 (0.41-0.83) | 0.44 | Ineligible |
| SG | Neuropathy peripheral | all_indication_supportive | 31 | 1.34 (0.94-1.90) | 0.97 | Ineligible |
| SG | Neutrophil count abnormal | all_indication_supportive | 31 | 46.82 (32.81-66.81) | 32.82 | Eligible |
| SG | Product dose omission issue | all_indication_supportive | 31 | 0.41 (0.29-0.59) | 0.31 | Ineligible |
| SG | Stomatitis | all_indication_supportive | 31 | 2.11 (1.48-3.00) | 1.49 | Eligible |
| SG | General physical condition abnormal | all_indication_supportive | 30 | 18.54 (12.93-26.56) | 13.30 | Eligible |
| SG | Infusion related reaction | all_indication_supportive | 30 | 1.91 (1.33-2.73) | 1.35 | Eligible |
| SG | Nasopharyngitis | all_indication_supportive | 29 | 0.63 (0.43-0.90) | 0.47 | Ineligible |
| SG | Ascites | all_indication_supportive | 28 | 4.35 (3.00-6.31) | 2.82 | Eligible |
| SG | Metastases to lung | all_indication_supportive | 28 | 10.12 (6.97-14.67) | 7.09 | Eligible |
| SG | Hyponatraemia | all_indication_supportive | 27 | 2.22 (1.52-3.24) | 1.53 | Eligible |
| SG | Intentional dose omission | all_indication_supportive | 27 | 3.76 (2.57-5.49) | 2.45 | Eligible |
| SG | Liver disorder | all_indication_supportive | 27 | 2.82 (1.93-4.12) | 1.89 | Eligible |
| SG | Arthralgia | all_indication_supportive | 26 | 0.26 (0.17-0.38) | 0.19 | Ineligible |
| SG | Pain in extremity | all_indication_supportive | 26 | 0.37 (0.25-0.55) | 0.28 | Ineligible |
| SG | Pruritus | all_indication_supportive | 26 | 0.29 (0.20-0.42) | 0.22 | Ineligible |
| SG | Chills | all_indication_supportive | 25 | 0.95 (0.64-1.40) | 0.68 | Ineligible |
| SG | Haematotoxicity | all_indication_supportive | 25 | 11.01 (7.43-16.32) | 7.60 | Eligible |
| SG | Haemoglobin decreased | all_indication_supportive | 25 | 1.11 (0.75-1.65) | 0.79 | Ineligible |
| SG | Product preparation error | all_indication_supportive | 25 | 4.25 (2.87-6.30) | 2.68 | Eligible |
| SG | Oxygen saturation decreased | all_indication_supportive | 24 | 1.80 (1.21-2.69) | 1.23 | Eligible |
| SG | Product use issue | all_indication_supportive | 24 | 0.46 (0.31-0.68) | 0.33 | Ineligible |
| SG | Pulmonary embolism | all_indication_supportive | 24 | 1.26 (0.84-1.88) | 0.88 | Ineligible |
| SG | Enteritis | all_indication_supportive | 23 | 14.77 (9.80-22.27) | 10.13 | Eligible |
| SG | Hepatic function abnormal | all_indication_supportive | 23 | 2.87 (1.91-4.33) | 1.85 | Eligible |
| SG | Neoplasm progression | all_indication_supportive | 23 | 2.50 (1.66-3.77) | 1.64 | Eligible |
| SG | Somnolence | all_indication_supportive | 23 | 0.50 (0.33-0.76) | 0.37 | Ineligible |
| SG | Agranulocytosis | all_indication_supportive | 22 | 5.38 (3.54-8.18) | 3.21 | Eligible |
| SG | Cytopenia | all_indication_supportive | 22 | 6.78 (4.46-10.32) | 4.09 | Eligible |
| SG | Hepatic cytolysis | all_indication_supportive | 22 | 6.91 (4.54-10.51) | 4.18 | Eligible |
| SG | Cholestasis | all_indication_supportive | 21 | 5.07 (3.30-7.79) | 2.99 | Eligible |
| SG | Condition aggravated | all_indication_supportive | 21 | 0.28 (0.18-0.43) | 0.20 | Ineligible |
| SG | Fall | all_indication_supportive | 21 | 0.27 (0.18-0.41) | 0.20 | Ineligible |
| SG | Metastases to lymph nodes | all_indication_supportive | 21 | 13.36 (8.70-20.53) | 8.98 | Eligible |
| SG | Bone pain | all_indication_supportive | 20 | 1.47 (0.95-2.28) | 0.98 | Ineligible |
| SG | Chest pain | all_indication_supportive | 20 | 0.52 (0.34-0.81) | 0.37 | Ineligible |
| SG | Erythema | all_indication_supportive | 20 | 0.40 (0.26-0.62) | 0.29 | Ineligible |
| SG | Illness | all_indication_supportive | 20 | 0.74 (0.48-1.15) | 0.52 | Ineligible |
| SG | Incorrect dose administered | all_indication_supportive | 20 | 0.35 (0.23-0.54) | 0.25 | Ineligible |
| SG | Malignant neoplasm progression | all_indication_supportive | 20 | 0.77 (0.50-1.20) | 0.54 | Ineligible |
| SG | Product preparation issue | all_indication_supportive | 20 | 13.68 (8.81-21.25) | 9.10 | Eligible |
| SG | Syncope | all_indication_supportive | 20 | 0.92 (0.59-1.43) | 0.64 | Ineligible |
| SG | Toxicity to various agents | all_indication_supportive | 20 | 0.45 (0.29-0.70) | 0.32 | Ineligible |
| SG | Dysphagia | all_indication_supportive | 19 | 0.89 (0.57-1.40) | 0.61 | Ineligible |
| SG | Hypersensitivity | all_indication_supportive | 19 | 0.43 (0.27-0.67) | 0.30 | Ineligible |
| SG | Metastases to meninges | all_indication_supportive | 19 | 34.77 (22.11-54.69) | 22.34 | Eligible |
| SG | Respiratory failure | all_indication_supportive | 19 | 1.23 (0.78-1.93) | 0.82 | Ineligible |
| SG | Seizure | all_indication_supportive | 19 | 0.59 (0.38-0.93) | 0.41 | Ineligible |
| SG | Breast cancer metastatic | all_indication_supportive | 18 | 8.71 (5.48-13.85) | 5.16 | Eligible |
| SG | Electrolyte imbalance | all_indication_supportive | 18 | 7.05 (4.44-11.21) | 3.97 | Eligible |
| SG | Oropharyngeal pain | all_indication_supportive | 18 | 0.79 (0.50-1.25) | 0.54 | Ineligible |
| SG | Product storage error | all_indication_supportive | 18 | 0.75 (0.47-1.19) | 0.51 | Ineligible |
| SG | SARS-CoV-2 test positive | all_indication_supportive | 18 | 4.67 (2.94-7.41) | 2.63 | Eligible |
| SG | Tachycardia | all_indication_supportive | 18 | 0.94 (0.59-1.49) | 0.64 | Ineligible |
| SG | Alanine aminotransferase increased | all_indication_supportive | 17 | 1.50 (0.93-2.42) | 0.97 | Ineligible |
| SG | Blood bilirubin increased | all_indication_supportive | 17 | 3.30 (2.05-5.31) | 1.92 | Eligible |
| SG | Feeling abnormal | all_indication_supportive | 17 | 0.30 (0.19-0.49) | 0.21 | Ineligible |
| SG | Product use in unapproved indication | all_indication_supportive | 17 | 0.25 (0.15-0.40) | 0.18 | Ineligible |
| SG | Atrial fibrillation | all_indication_supportive | 16 | 0.73 (0.45-1.20) | 0.49 | Ineligible |
| SG | Cholinergic syndrome | all_indication_supportive | 16 | 118.41 (71.87-195.09) | 64.12 | Eligible |
| SG | Hypoaesthesia | all_indication_supportive | 16 | 0.47 (0.29-0.77) | 0.32 | Ineligible |
| SG | Metastasis | all_indication_supportive | 16 | 9.56 (5.85-15.63) | 5.56 | Eligible |
| SG | Productive cough | all_indication_supportive | 16 | 1.36 (0.83-2.22) | 0.87 | Ineligible |
| SG | Anxiety | all_indication_supportive | 15 | 0.23 (0.14-0.39) | 0.16 | Ineligible |
| SG | Breast cancer | all_indication_supportive | 15 | 0.69 (0.42-1.15) | 0.46 | Ineligible |
| SG | Cardiac failure | all_indication_supportive | 15 | 0.83 (0.50-1.38) | 0.55 | Ineligible |
| SG | Dry skin | all_indication_supportive | 15 | 0.43 (0.26-0.72) | 0.30 | Ineligible |
| SG | Enterocolitis | all_indication_supportive | 15 | 11.04 (6.65-18.35) | 6.56 | Eligible |
| SG | Flushing | all_indication_supportive | 15 | 0.74 (0.45-1.24) | 0.49 | Ineligible |
| SG | Full blood count decreased | all_indication_supportive | 15 | 2.76 (1.66-4.58) | 1.59 | Eligible |
| SG | Gastrointestinal disorder | all_indication_supportive | 15 | 0.70 (0.42-1.16) | 0.47 | Ineligible |
| SG | Hepatotoxicity | all_indication_supportive | 15 | 2.94 (1.77-4.88) | 1.67 | Eligible |
| SG | Myalgia | all_indication_supportive | 15 | 0.40 (0.24-0.66) | 0.27 | Ineligible |
| SG | Poor venous access | all_indication_supportive | 15 | 5.87 (3.54-9.76) | 3.02 | Eligible |
| SG | Renal failure | all_indication_supportive | 15 | 0.52 (0.31-0.86) | 0.35 | Ineligible |
| SG | Therapy partial responder | all_indication_supportive | 15 | 3.77 (2.27-6.27) | 2.06 | Eligible |
| SG | Unevaluable event | all_indication_supportive | 15 | 0.83 (0.50-1.38) | 0.55 | Ineligible |
| SG | Blood test abnormal | all_indication_supportive | 14 | 3.90 (2.31-6.59) | 2.06 | Eligible |
| SG | Chest discomfort | all_indication_supportive | 14 | 0.60 (0.36-1.02) | 0.40 | Ineligible |
| SG | Gamma-glutamyltransferase increased | all_indication_supportive | 14 | 3.59 (2.12-6.06) | 1.93 | Eligible |
| SG | Hepatic enzyme increased | all_indication_supportive | 14 | 0.90 (0.53-1.51) | 0.58 | Ineligible |
| SG | Insomnia | all_indication_supportive | 14 | 0.23 (0.14-0.39) | 0.16 | Ineligible |
| SG | Intentional product use issue | all_indication_supportive | 14 | 0.56 (0.33-0.95) | 0.37 | Ineligible |
| SG | Paraesthesia | all_indication_supportive | 14 | 0.40 (0.23-0.67) | 0.27 | Ineligible |
| SG | Thrombosis | all_indication_supportive | 14 | 0.78 (0.46-1.31) | 0.51 | Ineligible |
| SG | Abdominal discomfort | all_indication_supportive | 13 | 0.31 (0.18-0.53) | 0.21 | Ineligible |
| SG | Abdominal distension | all_indication_supportive | 13 | 0.54 (0.32-0.94) | 0.36 | Ineligible |
| SG | Aspartate aminotransferase increased | all_indication_supportive | 13 | 1.38 (0.80-2.38) | 0.84 | Ineligible |
| SG | Cell death | all_indication_supportive | 13 | 27.97 (16.19-48.31) | 16.47 | Eligible |
| SG | Depression | all_indication_supportive | 13 | 0.27 (0.16-0.46) | 0.18 | Ineligible |
| SG | Drug-induced liver injury | all_indication_supportive | 13 | 1.56 (0.90-2.68) | 0.94 | Ineligible |
| SG | Dyspepsia | all_indication_supportive | 13 | 0.59 (0.35-1.02) | 0.39 | Ineligible |
| SG | Gastrointestinal toxicity | all_indication_supportive | 13 | 11.44 (6.63-19.74) | 6.46 | Eligible |
| SG | Herpes zoster | all_indication_supportive | 13 | 0.93 (0.54-1.61) | 0.59 | Ineligible |
| SG | Hypoxia | all_indication_supportive | 13 | 1.68 (0.97-2.89) | 1.00 | Ineligible |
| SG | Liver function test abnormal | all_indication_supportive | 13 | 2.57 (1.49-4.42) | 1.43 | Eligible |
| SG | Red blood cell count decreased | all_indication_supportive | 13 | 1.95 (1.13-3.36) | 1.14 | Eligible |
| SG | Complication associated with device | all_indication_supportive | 12 | 3.21 (1.82-5.66) | 1.66 | Eligible |
| SG | Confusional state | all_indication_supportive | 12 | 0.34 (0.19-0.59) | 0.22 | Ineligible |
| SG | Gastrooesophageal reflux disease | all_indication_supportive | 12 | 0.68 (0.38-1.19) | 0.43 | Ineligible |
| SG | Hypophagia | all_indication_supportive | 12 | 2.00 (1.14-3.53) | 1.14 | Eligible |
| SG | Respiratory tract infection | all_indication_supportive | 12 | 1.89 (1.07-3.33) | 1.08 | Eligible |
| SG | Therapeutic response decreased | all_indication_supportive | 12 | 0.86 (0.49-1.52) | 0.54 | Ineligible |
| SG | Therapy change | all_indication_supportive | 12 | 4.54 (2.58-8.01) | 2.19 | Eligible |
| SG | Bacteraemia | all_indication_supportive | 11 | 4.04 (2.24-7.31) | 1.92 | Eligible |
| SG | Dry mouth | all_indication_supportive | 11 | 0.60 (0.33-1.09) | 0.38 | Ineligible |
| SG | Epistaxis | all_indication_supportive | 11 | 0.62 (0.34-1.12) | 0.39 | Ineligible |
| SG | Hyperglycaemia | all_indication_supportive | 11 | 1.46 (0.81-2.64) | 0.85 | Ineligible |
| SG | Intestinal obstruction | all_indication_supportive | 11 | 1.25 (0.69-2.26) | 0.74 | Ineligible |
| SG | Laboratory test abnormal | all_indication_supportive | 11 | 1.45 (0.80-2.63) | 0.84 | Ineligible |
| SG | Liver function test increased | all_indication_supportive | 11 | 1.85 (1.03-3.35) | 1.04 | Eligible |
| SG | Memory impairment | all_indication_supportive | 11 | 0.33 (0.18-0.59) | 0.21 | Ineligible |
| SG | Multiple organ dysfunction syndrome | all_indication_supportive | 11 | 1.44 (0.80-2.60) | 0.84 | Ineligible |
| SG | Muscle spasms | all_indication_supportive | 11 | 0.26 (0.14-0.46) | 0.17 | Ineligible |
| SG | Sleep disorder | all_indication_supportive | 11 | 0.66 (0.36-1.19) | 0.41 | Ineligible |
| SG | Tremor | all_indication_supportive | 11 | 0.31 (0.17-0.55) | 0.20 | Ineligible |
| SG | Urticaria | all_indication_supportive | 11 | 0.29 (0.16-0.52) | 0.19 | Ineligible |
| SG | Vision blurred | all_indication_supportive | 11 | 0.36 (0.20-0.65) | 0.23 | Ineligible |
| SG | Aplasia | all_indication_supportive | 10 | 23.54 (12.63-43.87) | 12.89 | Eligible |
| SG | Blood creatinine increased | all_indication_supportive | 10 | 0.74 (0.40-1.37) | 0.45 | Ineligible |
| SG | Cerebrovascular accident | all_indication_supportive | 10 | 0.29 (0.16-0.55) | 0.19 | Ineligible |
| SG | Circumstance or information capable of leading to medication error | all_indication_supportive | 10 | 1.12 (0.60-2.08) | 0.65 | Ineligible |
| SG | Gait disturbance | all_indication_supportive | 10 | 0.22 (0.12-0.40) | 0.14 | Ineligible |
| SG | Hyperthermia | all_indication_supportive | 10 | 6.27 (3.37-11.68) | 2.62 | Eligible |
| SG | Lung disorder | all_indication_supportive | 10 | 0.87 (0.47-1.62) | 0.52 | Ineligible |
| SG | Lymphopenia | all_indication_supportive | 10 | 3.01 (1.62-5.59) | 1.47 | Eligible |
| SG | Muscular weakness | all_indication_supportive | 10 | 0.39 (0.21-0.72) | 0.25 | Ineligible |
| SG | Peripheral swelling | all_indication_supportive | 10 | 0.23 (0.12-0.43) | 0.15 | Ineligible |
| SG | Rash pruritic | all_indication_supportive | 10 | 0.81 (0.43-1.50) | 0.49 | Ineligible |
| SG | White blood cell count abnormal | all_indication_supportive | 10 | 6.19 (3.33-11.52) | 2.59 | Eligible |
| SG | Anaphylactic reaction | all_indication_supportive | 9 | 0.72 (0.38-1.39) | 0.43 | Ineligible |
| SG | Blood pressure decreased | all_indication_supportive | 9 | 0.63 (0.33-1.21) | 0.38 | Ineligible |
| SG | Bone marrow failure | all_indication_supportive | 9 | 1.95 (1.01-3.75) | 1.01 | Ineligible |
| SG | Cardiac arrest | all_indication_supportive | 9 | 0.54 (0.28-1.04) | 0.33 | Ineligible |
| SG | Device related infection | all_indication_supportive | 9 | 2.37 (1.23-4.55) | 1.18 | Ineligible |
| SG | Diarrhoea haemorrhagic | all_indication_supportive | 9 | 4.26 (2.22-8.20) | 1.82 | Ineligible |
| SG | Drug intolerance | all_indication_supportive | 9 | 0.35 (0.18-0.68) | 0.22 | Ineligible |
| SG | Dyspnoea exertional | all_indication_supportive | 9 | 0.99 (0.51-1.90) | 0.57 | Ineligible |
| SG | Epilepsy | all_indication_supportive | 9 | 1.29 (0.67-2.48) | 0.72 | Ineligible |
| SG | Gastrointestinal haemorrhage | all_indication_supportive | 9 | 0.46 (0.24-0.88) | 0.28 | Ineligible |
| SG | Hot flush | all_indication_supportive | 9 | 0.49 (0.26-0.94) | 0.30 | Ineligible |
| SG | Hypercalcaemia | all_indication_supportive | 9 | 3.27 (1.70-6.28) | 1.50 | Ineligible |
| SG | Hyperhidrosis | all_indication_supportive | 9 | 0.32 (0.17-0.61) | 0.20 | Ineligible |
| SG | Hypocalcaemia | all_indication_supportive | 9 | 2.12 (1.10-4.08) | 1.08 | Ineligible |
| SG | Lymphadenopathy | all_indication_supportive | 9 | 1.15 (0.60-2.22) | 0.65 | Ineligible |
| SG | Metastases to spine | all_indication_supportive | 9 | 11.36 (5.90-21.87) | 5.04 | Ineligible |
| SG | Nervous system disorder | all_indication_supportive | 9 | 1.81 (0.94-3.49) | 0.95 | Ineligible |
| SG | Product administration error | all_indication_supportive | 9 | 1.64 (0.85-3.15) | 0.88 | Ineligible |
| SG | Respiratory distress | all_indication_supportive | 9 | 1.57 (0.82-3.02) | 0.85 | Ineligible |
| SG | Rhinorrhoea | all_indication_supportive | 9 | 0.56 (0.29-1.07) | 0.34 | Ineligible |
| SG | Skin lesion | all_indication_supportive | 9 | 1.42 (0.74-2.74) | 0.78 | Ineligible |
| SG | Surgery | all_indication_supportive | 9 | 0.65 (0.34-1.25) | 0.39 | Ineligible |
| SG | Swelling | all_indication_supportive | 9 | 0.39 (0.20-0.75) | 0.24 | Ineligible |
| SG | Therapy non-responder | all_indication_supportive | 9 | 0.77 (0.40-1.49) | 0.46 | Ineligible |
| SG | Blood disorder | all_indication_supportive | 8 | 4.46 (2.23-8.92) | 1.77 | Ineligible |
| SG | Blood glucose increased | all_indication_supportive | 8 | 0.19 (0.10-0.39) | 0.12 | Ineligible |
| SG | Breast cancer recurrent | all_indication_supportive | 8 | 11.69 (5.84-23.42) | 4.76 | Ineligible |
| SG | Discomfort | all_indication_supportive | 8 | 0.52 (0.26-1.04) | 0.31 | Ineligible |
| SG | Dysgeusia | all_indication_supportive | 8 | 0.50 (0.25-0.99) | 0.30 | Ineligible |
| SG | Haemoglobin abnormal | all_indication_supportive | 8 | 4.99 (2.49-9.98) | 1.92 | Ineligible |
| SG | Inflammation | all_indication_supportive | 8 | 0.64 (0.32-1.29) | 0.38 | Ineligible |
| SG | Loss of consciousness | all_indication_supportive | 8 | 0.30 (0.15-0.60) | 0.19 | Ineligible |
| SG | Lower respiratory tract infection | all_indication_supportive | 8 | 0.70 (0.35-1.39) | 0.41 | Ineligible |
| SG | Lymphocyte count decreased | all_indication_supportive | 8 | 1.77 (0.89-3.55) | 0.90 | Ineligible |
| SG | Lymphoedema | all_indication_supportive | 8 | 4.86 (2.43-9.73) | 1.88 | Ineligible |
| SG | Metastases to skin | all_indication_supportive | 8 | 31.27 (15.58-62.75) | 15.51 | Ineligible |
| SG | Neoplasm | all_indication_supportive | 8 | 2.83 (1.41-5.66) | 1.28 | Ineligible |
| SG | Renal impairment | all_indication_supportive | 8 | 0.40 (0.20-0.81) | 0.24 | Ineligible |
| SG | Transaminases increased | all_indication_supportive | 8 | 1.59 (0.79-3.18) | 0.82 | Ineligible |
| SG | Transfusion | all_indication_supportive | 8 | 2.88 (1.44-5.76) | 1.30 | Ineligible |
| SG | Balance disorder | all_indication_supportive | 7 | 0.35 (0.17-0.74) | 0.21 | Ineligible |
| SG | Blood alkaline phosphatase increased | all_indication_supportive | 7 | 1.66 (0.79-3.49) | 0.82 | Ineligible |
| SG | Candida infection | all_indication_supportive | 7 | 1.50 (0.72-3.16) | 0.76 | Ineligible |
| SG | Cellulitis | all_indication_supportive | 7 | 0.61 (0.29-1.27) | 0.35 | Ineligible |
| SG | Central nervous system lesion | all_indication_supportive | 7 | 2.06 (0.98-4.32) | 0.96 | Ineligible |
| SG | Clostridium difficile infection | all_indication_supportive | 7 | 1.16 (0.55-2.44) | 0.61 | Ineligible |
| SG | Cystitis | all_indication_supportive | 7 | 0.94 (0.45-1.97) | 0.51 | Ineligible |
| SG | Depressed mood | all_indication_supportive | 7 | 0.55 (0.26-1.15) | 0.32 | Ineligible |
| SG | Diverticulitis | all_indication_supportive | 7 | 1.07 (0.51-2.24) | 0.57 | Ineligible |
| SG | Dysphonia | all_indication_supportive | 7 | 0.50 (0.24-1.06) | 0.29 | Ineligible |
| SG | Enterocolitis infectious | all_indication_supportive | 7 | 40.33 (19.13-85.01) | 18.42 | Ineligible |
| SG | Feeding disorder | all_indication_supportive | 7 | 1.32 (0.63-2.77) | 0.68 | Ineligible |
| SG | Femur fracture | all_indication_supportive | 7 | 1.09 (0.52-2.28) | 0.58 | Ineligible |
| SG | Granulocyte count decreased | all_indication_supportive | 7 | 17.53 (8.34-36.86) | 8.01 | Ineligible |
| SG | Hospice care | all_indication_supportive | 7 | 2.59 (1.23-5.44) | 1.14 | Ineligible |
| SG | Hyperbilirubinaemia | all_indication_supportive | 7 | 3.02 (1.44-6.35) | 1.27 | Ineligible |
| SG | Hypomagnesaemia | all_indication_supportive | 7 | 2.17 (1.04-4.56) | 1.00 | Ineligible |
| SG | Ileus | all_indication_supportive | 7 | 2.71 (1.29-5.69) | 1.17 | Ineligible |
| SG | Influenza | all_indication_supportive | 7 | 0.25 (0.12-0.52) | 0.15 | Ineligible |
| SG | Jaundice | all_indication_supportive | 7 | 1.37 (0.65-2.88) | 0.70 | Ineligible |
| SG | Neoplasm malignant | all_indication_supportive | 7 | 0.43 (0.21-0.91) | 0.25 | Ineligible |
| SG | Palpitations | all_indication_supportive | 7 | 0.27 (0.13-0.57) | 0.16 | Ineligible |
| SG | Pericardial effusion | all_indication_supportive | 7 | 1.36 (0.65-2.86) | 0.70 | Ineligible |
| SG | Stress | all_indication_supportive | 7 | 0.41 (0.20-0.86) | 0.24 | Ineligible |
| SG | Throat irritation | all_indication_supportive | 7 | 0.67 (0.32-1.41) | 0.38 | Ineligible |
| SG | Vertigo | all_indication_supportive | 7 | 0.50 (0.24-1.04) | 0.29 | Ineligible |
| SG | Visual impairment | all_indication_supportive | 7 | 0.23 (0.11-0.48) | 0.14 | Ineligible |
| SG | Acute respiratory failure | all_indication_supportive | 6 | 1.31 (0.59-2.92) | 0.64 | Ineligible |
| SG | Anaphylactic shock | all_indication_supportive | 6 | 1.09 (0.49-2.44) | 0.56 | Ineligible |
| SG | Blood magnesium decreased | all_indication_supportive | 6 | 2.93 (1.31-6.52) | 1.15 | Ineligible |
| SG | C-reactive protein increased | all_indication_supportive | 6 | 0.72 (0.32-1.60) | 0.39 | Ineligible |
| SG | Cerebral haemorrhage | all_indication_supportive | 6 | 0.74 (0.33-1.65) | 0.40 | Ineligible |
| SG | Cholecystitis | all_indication_supportive | 6 | 2.56 (1.15-5.70) | 1.05 | Ineligible |
| SG | Clostridium difficile colitis | all_indication_supportive | 6 | 2.49 (1.12-5.54) | 1.03 | Ineligible |
| SG | Deep vein thrombosis | all_indication_supportive | 6 | 0.46 (0.21-1.03) | 0.26 | Ineligible |
| SG | Disseminated intravascular coagulation | all_indication_supportive | 6 | 2.33 (1.05-5.19) | 0.99 | Ineligible |
| SG | Dry eye | all_indication_supportive | 6 | 0.50 (0.23-1.12) | 0.28 | Ineligible |
| SG | Eating disorder | all_indication_supportive | 6 | 1.20 (0.54-2.67) | 0.60 | Ineligible |
| SG | Erysipelas | all_indication_supportive | 6 | 5.10 (2.29-11.36) | 1.64 | Ineligible |
| SG | Faeces soft | all_indication_supportive | 6 | 2.99 (1.34-6.65) | 1.17 | Ineligible |
| SG | Feeling hot | all_indication_supportive | 6 | 0.45 (0.20-1.01) | 0.26 | Ineligible |
| SG | Full blood count abnormal | all_indication_supportive | 6 | 2.08 (0.93-4.63) | 0.91 | Ineligible |
| SG | Gait inability | all_indication_supportive | 6 | 0.65 (0.29-1.45) | 0.36 | Ineligible |
| SG | Haemorrhoids | all_indication_supportive | 6 | 1.39 (0.62-3.09) | 0.67 | Ineligible |
| SG | Hepatic cirrhosis | all_indication_supportive | 6 | 1.41 (0.63-3.13) | 0.68 | Ineligible |
| SG | Hypersomnia | all_indication_supportive | 6 | 0.91 (0.41-2.02) | 0.48 | Ineligible |
| SG | Hypothyroidism | all_indication_supportive | 6 | 0.82 (0.37-1.82) | 0.43 | Ineligible |
| SG | Large intestine perforation | all_indication_supportive | 6 | 3.97 (1.78-8.85) | 1.40 | Ineligible |
| SG | Lethargy | all_indication_supportive | 6 | 0.48 (0.22-1.08) | 0.27 | Ineligible |
| SG | Mass | all_indication_supportive | 6 | 1.78 (0.80-3.96) | 0.81 | Ineligible |
| SG | Metastases to pleura | all_indication_supportive | 6 | 24.20 (10.83-54.04) | 10.59 | Ineligible |
| SG | Musculoskeletal chest pain | all_indication_supportive | 6 | 1.69 (0.76-3.77) | 0.78 | Ineligible |
| SG | Oedema peripheral | all_indication_supportive | 6 | 0.29 (0.13-0.64) | 0.17 | Ineligible |
| SG | Palliative care | all_indication_supportive | 6 | 14.72 (6.60-32.83) | 5.11 | Ineligible |
| SG | Pulmonary mass | all_indication_supportive | 6 | 1.52 (0.68-3.39) | 0.72 | Ineligible |
| SG | Pyelonephritis | all_indication_supportive | 6 | 3.06 (1.37-6.81) | 1.18 | Ineligible |
| SG | Radiotherapy | all_indication_supportive | 6 | 12.80 (5.74-28.55) | 4.02 | Ineligible |
| SG | Red cell distribution width increased | all_indication_supportive | 6 | 5.61 (2.52-12.51) | 1.75 | Ineligible |
| SG | Sinusitis | all_indication_supportive | 6 | 0.24 (0.11-0.54) | 0.14 | Ineligible |
| SG | Swelling face | all_indication_supportive | 6 | 0.42 (0.19-0.93) | 0.24 | Ineligible |
| SG | Treatment failure | all_indication_supportive | 6 | 0.26 (0.12-0.59) | 0.15 | Ineligible |
| SG | Urinary retention | all_indication_supportive | 6 | 0.80 (0.36-1.78) | 0.43 | Ineligible |
| SG | Viral infection | all_indication_supportive | 6 | 0.78 (0.35-1.73) | 0.42 | Ineligible |
| SG | White blood cell count increased | all_indication_supportive | 6 | 0.75 (0.34-1.67) | 0.40 | Ineligible |
| SG | Accidental overdose | all_indication_supportive | 5 | 0.59 (0.25-1.43) | 0.31 | Ineligible |
| SG | Blood lactate dehydrogenase increased | all_indication_supportive | 5 | 1.79 (0.74-4.30) | 0.76 | Ineligible |
| SG | Body temperature increased | all_indication_supportive | 5 | 1.16 (0.48-2.79) | 0.55 | Ineligible |
| SG | Brain oedema | all_indication_supportive | 5 | 1.90 (0.79-4.56) | 0.79 | Ineligible |
| SG | Breast mass | all_indication_supportive | 5 | 3.51 (1.46-8.44) | 1.17 | Ineligible |
| SG | Bronchitis | all_indication_supportive | 5 | 0.28 (0.12-0.68) | 0.16 | Ineligible |
| SG | Cardio-respiratory arrest | all_indication_supportive | 5 | 0.61 (0.25-1.47) | 0.32 | Ineligible |
| SG | Cholangitis | all_indication_supportive | 5 | 3.60 (1.50-8.66) | 1.19 | Ineligible |
| SG | Cholecystitis acute | all_indication_supportive | 5 | 4.52 (1.88-10.87) | 1.36 | Ineligible |
| SG | Cognitive disorder | all_indication_supportive | 5 | 0.45 (0.19-1.09) | 0.25 | Ineligible |
| SG | Device issue | all_indication_supportive | 5 | 0.21 (0.09-0.50) | 0.12 | Ineligible |
| SG | Device malfunction | all_indication_supportive | 5 | 0.30 (0.12-0.72) | 0.17 | Ineligible |
| SG | Device occlusion | all_indication_supportive | 5 | 1.35 (0.56-3.24) | 0.62 | Ineligible |
| SG | Disease recurrence | all_indication_supportive | 5 | 0.40 (0.17-0.97) | 0.22 | Ineligible |
| SG | Eastern Cooperative Oncology Group performance status worsened | all_indication_supportive | 5 | 14.89 (6.18-35.86) | 4.01 | Ineligible |
| SG | Gastroenteritis | all_indication_supportive | 5 | 1.59 (0.66-3.81) | 0.70 | Ineligible |
| SG | Gastrointestinal inflammation | all_indication_supportive | 5 | 2.54 (1.06-6.11) | 0.96 | Ineligible |
| SG | Generalised oedema | all_indication_supportive | 5 | 1.98 (0.82-4.76) | 0.81 | Ineligible |
| SG | Generalised tonic-clonic seizure | all_indication_supportive | 5 | 1.09 (0.45-2.62) | 0.52 | Ineligible |
| SG | Hair growth abnormal | all_indication_supportive | 5 | 2.99 (1.24-7.19) | 1.06 | Ineligible |
| SG | Hepatic lesion | all_indication_supportive | 5 | 5.06 (2.11-12.18) | 1.45 | Ineligible |
| SG | Hepatic neoplasm | all_indication_supportive | 5 | 6.89 (2.86-16.57) | 1.77 | Ineligible |
| SG | Hydronephrosis | all_indication_supportive | 5 | 3.08 (1.28-7.41) | 1.08 | Ineligible |
| SG | Hyperkalaemia | all_indication_supportive | 5 | 0.67 (0.28-1.60) | 0.35 | Ineligible |
| SG | Hypertransaminasaemia | all_indication_supportive | 5 | 2.83 (1.18-6.80) | 1.03 | Ineligible |
| SG | Hypoproteinaemia | all_indication_supportive | 5 | 13.92 (5.78-33.52) | 3.60 | Ineligible |
| SG | Immunodeficiency | all_indication_supportive | 5 | 1.28 (0.53-3.09) | 0.59 | Ineligible |
| SG | Ischaemic stroke | all_indication_supportive | 5 | 1.19 (0.49-2.86) | 0.56 | Ineligible |
| SG | Loss of personal independence in daily activities | all_indication_supportive | 5 | 0.37 (0.16-0.90) | 0.21 | Ineligible |
| SG | Malignant pleural effusion | all_indication_supportive | 5 | 13.53 (5.62-32.58) | 3.46 | Ineligible |
| SG | Maternal exposure during pregnancy | all_indication_supportive | 5 | 0.22 (0.09-0.52) | 0.12 | Ineligible |
| SG | Medication error | all_indication_supportive | 5 | 0.50 (0.21-1.21) | 0.27 | Ineligible |
| SG | Mouth ulceration | all_indication_supportive | 5 | 1.05 (0.44-2.52) | 0.51 | Ineligible |
| SG | Muscle atrophy | all_indication_supportive | 5 | 1.81 (0.75-4.36) | 0.77 | Ineligible |
| SG | Nasal congestion | all_indication_supportive | 5 | 0.35 (0.14-0.83) | 0.19 | Ineligible |
| SG | Neck pain | all_indication_supportive | 5 | 0.38 (0.16-0.92) | 0.21 | Ineligible |
| SG | Neuralgia | all_indication_supportive | 5 | 0.84 (0.35-2.02) | 0.42 | Ineligible |
| SG | Neurotoxicity | all_indication_supportive | 5 | 1.18 (0.49-2.84) | 0.56 | Ineligible |
| SG | Nutritional condition abnormal | all_indication_supportive | 5 | 32.70 (13.55-78.93) | 12.79 | Ineligible |
| SG | Oesophagitis | all_indication_supportive | 5 | 2.33 (0.97-5.60) | 0.91 | Ineligible |
| SG | Overdose | all_indication_supportive | 5 | 0.09 (0.04-0.22) | 0.05 | Ineligible |
| SG | Pancreatitis | all_indication_supportive | 5 | 0.48 (0.20-1.16) | 0.26 | Ineligible |
| SG | Prerenal failure | all_indication_supportive | 5 | 12.92 (5.37-31.11) | 3.24 | Ineligible |
| SG | Presyncope | all_indication_supportive | 5 | 0.88 (0.37-2.12) | 0.44 | Ineligible |
| SG | Product dispensing error | all_indication_supportive | 5 | 0.83 (0.35-1.99) | 0.42 | Ineligible |
| SG | Product temperature excursion issue | all_indication_supportive | 5 | 3.97 (1.65-9.56) | 1.26 | Ineligible |
| SG | Rash erythematous | all_indication_supportive | 5 | 0.48 (0.20-1.14) | 0.26 | Ineligible |
| SG | Retching | all_indication_supportive | 5 | 1.04 (0.43-2.50) | 0.50 | Ineligible |
| SG | Shock | all_indication_supportive | 5 | 1.07 (0.45-2.57) | 0.52 | Ineligible |
| SG | Sinus tachycardia | all_indication_supportive | 5 | 1.78 (0.74-4.29) | 0.76 | Ineligible |
| SG | Skin infection | all_indication_supportive | 5 | 1.82 (0.75-4.36) | 0.77 | Ineligible |
| SG | Skin ulcer | all_indication_supportive | 5 | 0.84 (0.35-2.01) | 0.42 | Ineligible |
| SG | Small intestinal obstruction | all_indication_supportive | 5 | 1.82 (0.76-4.39) | 0.77 | Ineligible |
| SG | Staphylococcal sepsis | all_indication_supportive | 5 | 5.31 (2.21-12.78) | 1.49 | Ineligible |
| SG | Therapy interrupted | all_indication_supportive | 5 | 0.27 (0.11-0.65) | 0.15 | Ineligible |
| SG | Tubulointerstitial nephritis | all_indication_supportive | 5 | 1.05 (0.44-2.52) | 0.51 | Ineligible |
| SG | Tumour marker increased | all_indication_supportive | 5 | 4.00 (1.66-9.61) | 1.26 | Ineligible |
| SG | Upper respiratory tract infection | all_indication_supportive | 5 | 0.45 (0.19-1.09) | 0.25 | Ineligible |
| SG | Urosepsis | all_indication_supportive | 5 | 2.37 (0.99-5.70) | 0.92 | Ineligible |
| SG | Vein collapse | all_indication_supportive | 5 | 23.21 (9.63-55.95) | 8.77 | Ineligible |
| SG | Wound | all_indication_supportive | 5 | 0.69 (0.29-1.66) | 0.36 | Ineligible |
| SG | Abscess | all_indication_supportive | 4 | 1.10 (0.41-2.94) | 0.49 | Ineligible |
| SG | Acute myocardial infarction | all_indication_supportive | 4 | 0.66 (0.25-1.76) | 0.32 | Ineligible |
| SG | Bedridden | all_indication_supportive | 4 | 1.35 (0.51-3.60) | 0.57 | Ineligible |
| SG | Blood calcium decreased | all_indication_supportive | 4 | 1.61 (0.60-4.29) | 0.64 | Ineligible |
| SG | Brain neoplasm malignant | all_indication_supportive | 4 | 3.34 (1.25-8.92) | 1.00 | Ineligible |
| SG | Breast pain | all_indication_supportive | 4 | 1.71 (0.64-4.56) | 0.67 | Ineligible |
| SG | COVID-19 pneumonia | all_indication_supportive | 4 | 1.18 (0.44-3.15) | 0.51 | Ineligible |
| SG | Cachexia | all_indication_supportive | 4 | 3.62 (1.36-9.64) | 1.04 | Ineligible |
| SG | Cancer pain | all_indication_supportive | 4 | 6.06 (2.27-16.18) | 1.37 | Ineligible |
| SG | Cardiac disorder | all_indication_supportive | 4 | 0.20 (0.07-0.52) | 0.11 | Ineligible |
| SG | Cataract | all_indication_supportive | 4 | 0.28 (0.11-0.75) | 0.15 | Ineligible |
| SG | Cerebral disorder | all_indication_supportive | 4 | 2.14 (0.80-5.71) | 0.77 | Ineligible |
| SG | Cold sweat | all_indication_supportive | 4 | 1.06 (0.40-2.82) | 0.47 | Ineligible |
| SG | Colitis ischaemic | all_indication_supportive | 4 | 2.95 (1.11-7.88) | 0.93 | Ineligible |
| SG | Decreased activity | all_indication_supportive | 4 | 1.54 (0.58-4.11) | 0.62 | Ineligible |
| SG | Dermatitis | all_indication_supportive | 4 | 0.80 (0.30-2.13) | 0.38 | Ineligible |
| SG | Dermatitis contact | all_indication_supportive | 4 | 1.48 (0.55-3.94) | 0.61 | Ineligible |
| SG | Device infusion issue | all_indication_supportive | 4 | 5.98 (2.24-15.96) | 1.36 | Ineligible |
| SG | Diplopia | all_indication_supportive | 4 | 0.72 (0.27-1.92) | 0.35 | Ineligible |
| SG | Dose calculation error | all_indication_supportive | 4 | 24.40 (9.12-65.28) | 6.93 | Ineligible |
| SG | Drug eruption | all_indication_supportive | 4 | 1.03 (0.39-2.75) | 0.47 | Ineligible |
| SG | Drug hypersensitivity | all_indication_supportive | 4 | 0.08 (0.03-0.20) | 0.04 | Ineligible |
| SG | Eastern Cooperative Oncology Group performance status abnormal | all_indication_supportive | 4 | 145.12 (53.32-394.97) | 32.64 | Ineligible |
| SG | Ejection fraction decreased | all_indication_supportive | 4 | 1.16 (0.43-3.09) | 0.51 | Ineligible |
| SG | Embolism | all_indication_supportive | 4 | 2.28 (0.85-6.07) | 0.80 | Ineligible |
| SG | Encephalopathy | all_indication_supportive | 4 | 0.75 (0.28-2.00) | 0.36 | Ineligible |
| SG | Escherichia infection | all_indication_supportive | 4 | 2.22 (0.83-5.93) | 0.79 | Ineligible |
| SG | Escherichia urinary tract infection | all_indication_supportive | 4 | 4.26 (1.60-11.35) | 1.14 | Ineligible |
| SG | Feeling cold | all_indication_supportive | 4 | 0.64 (0.24-1.70) | 0.32 | Ineligible |
| SG | Feeling jittery | all_indication_supportive | 4 | 1.01 (0.38-2.69) | 0.46 | Ineligible |
| SG | Flatulence | all_indication_supportive | 4 | 0.32 (0.12-0.85) | 0.17 | Ineligible |
| SG | Fluid retention | all_indication_supportive | 4 | 0.32 (0.12-0.86) | 0.17 | Ineligible |
| SG | Foot fracture | all_indication_supportive | 4 | 0.89 (0.33-2.37) | 0.41 | Ineligible |
| SG | Fracture | all_indication_supportive | 4 | 0.86 (0.32-2.30) | 0.40 | Ineligible |
| SG | Gingival pain | all_indication_supportive | 4 | 2.30 (0.86-6.12) | 0.81 | Ineligible |
| SG | Glossodynia | all_indication_supportive | 4 | 0.76 (0.28-2.02) | 0.37 | Ineligible |
| SG | Haematoma | all_indication_supportive | 4 | 0.70 (0.26-1.86) | 0.34 | Ineligible |
| SG | Haematuria | all_indication_supportive | 4 | 0.52 (0.19-1.38) | 0.26 | Ineligible |
| SG | Hallucination | all_indication_supportive | 4 | 0.24 (0.09-0.65) | 0.13 | Ineligible |
| SG | Hepatomegaly | all_indication_supportive | 4 | 2.15 (0.80-5.72) | 0.77 | Ineligible |
| SG | Hypoglycaemia | all_indication_supportive | 4 | 0.40 (0.15-1.07) | 0.21 | Ineligible |
| SG | Hypophosphataemia | all_indication_supportive | 4 | 2.32 (0.87-6.20) | 0.81 | Ineligible |
| SG | Hypovolaemia | all_indication_supportive | 4 | 3.62 (1.36-9.66) | 1.04 | Ineligible |
| SG | Hypovolaemic shock | all_indication_supportive | 4 | 3.69 (1.38-9.83) | 1.05 | Ineligible |
| SG | Ileus paralytic | all_indication_supportive | 4 | 4.70 (1.76-12.55) | 1.20 | Ineligible |
| SG | Incorrect dosage administered | all_indication_supportive | 4 | 2.87 (1.08-7.67) | 0.92 | Ineligible |
| SG | Infusion related hypersensitivity reaction | all_indication_supportive | 4 | 27.05 (10.11-72.37) | 8.28 | Ineligible |
| SG | Intestinal ischaemia | all_indication_supportive | 4 | 3.48 (1.31-9.29) | 1.02 | Ineligible |
| SG | Large intestinal obstruction | all_indication_supportive | 4 | 6.83 (2.56-18.23) | 1.47 | Ineligible |
| SG | Localised infection | all_indication_supportive | 4 | 0.66 (0.25-1.77) | 0.33 | Ineligible |
| SG | Meningitis | all_indication_supportive | 4 | 2.77 (1.04-7.37) | 0.90 | Ineligible |
| SG | Mental status changes | all_indication_supportive | 4 | 0.73 (0.28-1.96) | 0.36 | Ineligible |
| SG | Metabolic acidosis | all_indication_supportive | 4 | 0.56 (0.21-1.49) | 0.28 | Ineligible |
| SG | Migraine | all_indication_supportive | 4 | 0.18 (0.07-0.47) | 0.10 | Ineligible |
| SG | Myocardial infarction | all_indication_supportive | 4 | 0.13 (0.05-0.34) | 0.07 | Ineligible |
| SG | Oesophageal stenosis | all_indication_supportive | 4 | 6.78 (2.54-18.10) | 1.47 | Ineligible |
| SG | Oral pain | all_indication_supportive | 4 | 0.76 (0.28-2.02) | 0.37 | Ineligible |
| SG | Pain of skin | all_indication_supportive | 4 | 0.56 (0.21-1.49) | 0.28 | Ineligible |
| SG | Pelvic pain | all_indication_supportive | 4 | 1.01 (0.38-2.69) | 0.46 | Ineligible |
| SG | Periorbital oedema | all_indication_supportive | 4 | 3.63 (1.36-9.68) | 1.05 | Ineligible |
| SG | Pharyngitis | all_indication_supportive | 4 | 1.39 (0.52-3.72) | 0.58 | Ineligible |
| SG | Platelet count abnormal | all_indication_supportive | 4 | 2.62 (0.98-6.98) | 0.87 | Ineligible |
| SG | Pneumocystis jirovecii pneumonia | all_indication_supportive | 4 | 1.43 (0.54-3.81) | 0.59 | Ineligible |
| SG | Pneumonia aspiration | all_indication_supportive | 4 | 0.71 (0.27-1.89) | 0.34 | Ineligible |
| SG | Pneumothorax | all_indication_supportive | 4 | 1.08 (0.40-2.87) | 0.48 | Ineligible |
| SG | Pulmonary sepsis | all_indication_supportive | 4 | 10.78 (4.04-28.78) | 2.05 | Ineligible |
| SG | Pulmonary toxicity | all_indication_supportive | 4 | 2.37 (0.89-6.32) | 0.82 | Ineligible |
| SG | Rash macular | all_indication_supportive | 4 | 0.49 (0.18-1.29) | 0.25 | Ineligible |
| SG | Rectal haemorrhage | all_indication_supportive | 4 | 0.42 (0.16-1.11) | 0.22 | Ineligible |
| SG | Skin reaction | all_indication_supportive | 4 | 1.18 (0.44-3.15) | 0.52 | Ineligible |
| SG | Skin tightness | all_indication_supportive | 4 | 3.69 (1.38-9.83) | 1.05 | Ineligible |
| SG | Speech disorder | all_indication_supportive | 4 | 0.35 (0.13-0.93) | 0.19 | Ineligible |
| SG | Tooth extraction | all_indication_supportive | 4 | 1.61 (0.60-4.29) | 0.64 | Ineligible |
| SG | Tumour haemorrhage | all_indication_supportive | 4 | 6.01 (2.25-16.04) | 1.37 | Ineligible |
| SG | Underdose | all_indication_supportive | 4 | 0.20 (0.07-0.53) | 0.11 | Ineligible |
| SG | Wound infection | all_indication_supportive | 4 | 1.55 (0.58-4.13) | 0.62 | Ineligible |
| SG | Xerophthalmia | all_indication_supportive | 4 | 27.32 (10.21-73.11) | 8.41 | Ineligible |
| SG | Abdominal pain lower | all_indication_supportive | 3 | 0.53 (0.17-1.64) | 0.25 | Ineligible |
| SG | Acne | all_indication_supportive | 3 | 0.15 (0.05-0.45) | 0.08 | Ineligible |
| SG | Acute respiratory distress syndrome | all_indication_supportive | 3 | 0.82 (0.27-2.56) | 0.36 | Ineligible |
| SG | Alanine aminotransferase decreased | all_indication_supportive | 3 | 19.50 (6.27-60.68) | 2.52 | Ineligible |
| SG | Altered state of consciousness | all_indication_supportive | 3 | 0.59 (0.19-1.85) | 0.28 | Ineligible |
| SG | Anal fissure | all_indication_supportive | 3 | 2.28 (0.74-7.08) | 0.69 | Ineligible |
| SG | Aphasia | all_indication_supportive | 3 | 0.45 (0.15-1.40) | 0.22 | Ineligible |
| SG | Arthritis | all_indication_supportive | 3 | 0.16 (0.05-0.49) | 0.08 | Ineligible |
| SG | Bacterial sepsis | all_indication_supportive | 3 | 4.35 (1.40-13.51) | 0.95 | Ineligible |
| SG | Bladder cancer recurrent | all_indication_supportive | 3 | 11.85 (3.81-36.83) | 1.61 | Ineligible |
| SG | Blood bilirubin abnormal | all_indication_supportive | 3 | 10.41 (3.35-32.35) | 1.48 | Ineligible |
| SG | Blood creatinine decreased | all_indication_supportive | 3 | 4.18 (1.35-12.99) | 0.93 | Ineligible |
| SG | Blood glucose abnormal | all_indication_supportive | 3 | 0.65 (0.21-2.00) | 0.30 | Ineligible |
| SG | Blood lactic acid increased | all_indication_supportive | 3 | 2.51 (0.81-7.80) | 0.73 | Ineligible |
| SG | Blood potassium decreased | all_indication_supportive | 3 | 0.46 (0.15-1.42) | 0.22 | Ineligible |
| SG | Blood pressure fluctuation | all_indication_supportive | 3 | 0.49 (0.16-1.53) | 0.24 | Ineligible |
| SG | Blood pressure increased | all_indication_supportive | 3 | 0.09 (0.03-0.26) | 0.04 | Ineligible |
| SG | Blood urea increased | all_indication_supportive | 3 | 1.11 (0.36-3.44) | 0.44 | Ineligible |
| SG | Brain injury | all_indication_supportive | 3 | 1.19 (0.38-3.68) | 0.47 | Ineligible |
| SG | Brain neoplasm | all_indication_supportive | 3 | 1.38 (0.44-4.27) | 0.51 | Ineligible |
| SG | Breast discharge | all_indication_supportive | 3 | 8.74 (2.81-27.14) | 1.33 | Ineligible |
| SG | Breast haemorrhage | all_indication_supportive | 3 | 38.05 (12.19-118.76) | 8.14 | Ineligible |
| SG | Breast ulceration | all_indication_supportive | 3 | 75.52 (24.04-237.22) | 16.69 | Ineligible |
| SG | Bronchospasm | all_indication_supportive | 3 | 1.02 (0.33-3.16) | 0.42 | Ineligible |
| SG | Burning sensation | all_indication_supportive | 3 | 0.19 (0.06-0.59) | 0.10 | Ineligible |
| SG | Cardiomegaly | all_indication_supportive | 3 | 1.43 (0.46-4.45) | 0.53 | Ineligible |
| SG | Catheter site infection | all_indication_supportive | 3 | 3.30 (1.06-10.23) | 0.84 | Ineligible |
| SG | Central venous catheterisation | all_indication_supportive | 3 | 3.82 (1.23-11.87) | 0.89 | Ineligible |
| SG | Cholangiocarcinoma | all_indication_supportive | 3 | 7.30 (2.35-22.67) | 1.21 | Ineligible |
| SG | Clostridial infection | all_indication_supportive | 3 | 13.04 (4.20-40.54) | 1.73 | Ineligible |
| SG | Coma | all_indication_supportive | 3 | 0.30 (0.10-0.93) | 0.15 | Ineligible |
| SG | Congenital aplasia | all_indication_supportive | 3 | 38.77 (12.42-121.05) | 8.40 | Ineligible |
| SG | Contusion | all_indication_supportive | 3 | 0.13 (0.04-0.41) | 0.07 | Ineligible |
| SG | Counterfeit product administered | all_indication_supportive | 3 | 8.93 (2.88-27.74) | 1.34 | Ineligible |
| SG | Delirium | all_indication_supportive | 3 | 0.39 (0.13-1.21) | 0.19 | Ineligible |
| SG | Diabetes mellitus | all_indication_supportive | 3 | 0.20 (0.06-0.60) | 0.10 | Ineligible |
| SG | Discontinued product administered | all_indication_supportive | 3 | 34.19 (10.96-106.65) | 6.59 | Ineligible |
| SG | Dizziness postural | all_indication_supportive | 3 | 1.31 (0.42-4.07) | 0.50 | Ineligible |
| SG | Drug resistance | all_indication_supportive | 3 | 0.44 (0.14-1.38) | 0.22 | Ineligible |
| SG | Dysuria | all_indication_supportive | 3 | 0.37 (0.12-1.14) | 0.18 | Ineligible |
| SG | Electrocardiogram T wave abnormal | all_indication_supportive | 3 | 11.57 (3.72-35.94) | 1.58 | Ineligible |
| SG | Emotional disorder | all_indication_supportive | 3 | 0.43 (0.14-1.34) | 0.21 | Ineligible |
| SG | Energy increased | all_indication_supportive | 3 | 2.20 (0.71-6.82) | 0.68 | Ineligible |
| SG | Eye disorder | all_indication_supportive | 3 | 0.39 (0.13-1.21) | 0.19 | Ineligible |
| SG | Flashback | all_indication_supportive | 3 | 28.27 (9.07-88.09) | 4.33 | Ineligible |
| SG | Formication | all_indication_supportive | 3 | 2.05 (0.66-6.36) | 0.65 | Ineligible |
| SG | Frequent bowel movements | all_indication_supportive | 3 | 0.44 (0.14-1.38) | 0.22 | Ineligible |
| SG | Gastric disorder | all_indication_supportive | 3 | 0.35 (0.11-1.10) | 0.18 | Ineligible |
| SG | Gastric ulcer | all_indication_supportive | 3 | 0.75 (0.24-2.31) | 0.33 | Ineligible |
| SG | Gastroenteritis Escherichia coli | all_indication_supportive | 3 | 24.10 (7.74-75.04) | 3.31 | Ineligible |
| SG | Gastrointestinal pain | all_indication_supportive | 3 | 1.14 (0.37-3.53) | 0.45 | Ineligible |
| SG | Haematocrit decreased | all_indication_supportive | 3 | 0.83 (0.27-2.56) | 0.36 | Ineligible |
| SG | Head discomfort | all_indication_supportive | 3 | 0.65 (0.21-2.02) | 0.30 | Ineligible |
| SG | Hemiparesis | all_indication_supportive | 3 | 0.84 (0.27-2.61) | 0.36 | Ineligible |
| SG | Hepatitis | all_indication_supportive | 3 | 0.57 (0.19-1.78) | 0.27 | Ineligible |
| SG | Hepatitis acute | all_indication_supportive | 3 | 2.25 (0.72-6.97) | 0.69 | Ineligible |
| SG | Hepatocellular injury | all_indication_supportive | 3 | 0.80 (0.26-2.49) | 0.35 | Ineligible |
| SG | Hyperthyroidism | all_indication_supportive | 3 | 0.90 (0.29-2.79) | 0.38 | Ineligible |
| SG | Hypokinesia | all_indication_supportive | 3 | 0.87 (0.28-2.70) | 0.37 | Ineligible |
| SG | Immune system disorder | all_indication_supportive | 3 | 0.93 (0.30-2.87) | 0.39 | Ineligible |
| SG | Incorrect product administration duration | all_indication_supportive | 3 | 0.52 (0.17-1.62) | 0.25 | Ineligible |
| SG | Intestinal perforation | all_indication_supportive | 3 | 1.20 (0.39-3.72) | 0.47 | Ineligible |
| SG | Intra-abdominal fluid collection | all_indication_supportive | 3 | 6.04 (1.94-18.75) | 1.10 | Ineligible |
| SG | Joint swelling | all_indication_supportive | 3 | 0.10 (0.03-0.31) | 0.05 | Ineligible |
| SG | Lung infiltration | all_indication_supportive | 3 | 2.11 (0.68-6.55) | 0.66 | Ineligible |
| SG | Lymphangiosis carcinomatosa | all_indication_supportive | 3 | 13.14 (4.23-40.85) | 1.74 | Ineligible |
| SG | Mental disorder | all_indication_supportive | 3 | 0.32 (0.10-0.98) | 0.16 | Ineligible |
| SG | Metastases to peritoneum | all_indication_supportive | 3 | 4.92 (1.59-15.28) | 1.00 | Ineligible |
| SG | Necrotising colitis | all_indication_supportive | 3 | 11.42 (3.68-35.49) | 1.57 | Ineligible |
| SG | Neutrophil count increased | all_indication_supportive | 3 | 1.26 (0.40-3.90) | 0.48 | Ineligible |
| SG | Non-cardiac chest pain | all_indication_supportive | 3 | 5.23 (1.69-16.24) | 1.03 | Ineligible |
| SG | Oedema | all_indication_supportive | 3 | 0.25 (0.08-0.77) | 0.13 | Ineligible |
| SG | Oral fungal infection | all_indication_supportive | 3 | 5.00 (1.61-15.52) | 1.01 | Ineligible |
| SG | Oral herpes | all_indication_supportive | 3 | 0.62 (0.20-1.91) | 0.28 | Ineligible |
| SG | Osteomyelitis | all_indication_supportive | 3 | 0.77 (0.25-2.38) | 0.34 | Ineligible |
| SG | Osteonecrosis of jaw | all_indication_supportive | 3 | 0.51 (0.16-1.57) | 0.24 | Ineligible |
| SG | Palmar-plantar erythrodysaesthesia syndrome | all_indication_supportive | 3 | 0.54 (0.18-1.68) | 0.26 | Ineligible |
| SG | Pancreatitis acute | all_indication_supportive | 3 | 0.64 (0.21-1.98) | 0.29 | Ineligible |
| SG | Performance status decreased | all_indication_supportive | 3 | 3.09 (1.00-9.59) | 0.81 | Ineligible |
| SG | Peritonitis | all_indication_supportive | 3 | 0.63 (0.20-1.95) | 0.29 | Ineligible |
| SG | Pharyngeal inflammation | all_indication_supportive | 3 | 10.16 (3.27-31.57) | 1.45 | Ineligible |
| SG | Pollakiuria | all_indication_supportive | 3 | 0.32 (0.10-0.98) | 0.16 | Ineligible |
| SG | Poor quality sleep | all_indication_supportive | 3 | 0.58 (0.19-1.80) | 0.27 | Ineligible |
| SG | Portal hypertension | all_indication_supportive | 3 | 4.61 (1.48-14.30) | 0.97 | Ineligible |
| SG | Product container issue | all_indication_supportive | 3 | 1.31 (0.42-4.05) | 0.50 | Ineligible |
| SG | Product label confusion | all_indication_supportive | 3 | 1.87 (0.60-5.81) | 0.62 | Ineligible |
| SG | Product reconstitution quality issue | all_indication_supportive | 3 | 12.87 (4.14-40.02) | 1.71 | Ineligible |
| SG | Quality of life decreased | all_indication_supportive | 3 | 1.05 (0.34-3.26) | 0.43 | Ineligible |
| SG | Renal disorder | all_indication_supportive | 3 | 0.27 (0.09-0.85) | 0.14 | Ineligible |
| SG | Respiratory disorder | all_indication_supportive | 3 | 0.45 (0.14-1.38) | 0.22 | Ineligible |
| SG | Scar | all_indication_supportive | 3 | 0.80 (0.26-2.50) | 0.35 | Ineligible |
| SG | Sedation | all_indication_supportive | 3 | 0.52 (0.17-1.60) | 0.25 | Ineligible |
| SG | Skin cancer | all_indication_supportive | 3 | 0.54 (0.18-1.69) | 0.26 | Ineligible |
| SG | Skin discolouration | all_indication_supportive | 3 | 0.28 (0.09-0.86) | 0.14 | Ineligible |
| SG | Skin hyperpigmentation | all_indication_supportive | 3 | 1.48 (0.48-4.59) | 0.54 | Ineligible |
| SG | Spinal cord compression | all_indication_supportive | 3 | 3.35 (1.08-10.41) | 0.84 | Ineligible |
| SG | Spinal pain | all_indication_supportive | 3 | 0.92 (0.30-2.86) | 0.39 | Ineligible |
| SG | Staphylococcal infection | all_indication_supportive | 3 | 0.44 (0.14-1.36) | 0.21 | Ineligible |
| SG | Tachypnoea | all_indication_supportive | 3 | 1.01 (0.32-3.13) | 0.41 | Ineligible |
| SG | Taste disorder | all_indication_supportive | 3 | 0.55 (0.18-1.70) | 0.26 | Ineligible |
| SG | Therapeutic product effect decreased | all_indication_supportive | 3 | 0.24 (0.08-0.75) | 0.13 | Ineligible |
| SG | Therapy cessation | all_indication_supportive | 3 | 0.21 (0.07-0.65) | 0.11 | Ineligible |
| SG | Tongue coated | all_indication_supportive | 3 | 8.03 (2.59-24.94) | 1.27 | Ineligible |
| SG | Tonsillitis | all_indication_supportive | 3 | 2.37 (0.76-7.34) | 0.71 | Ineligible |
| SG | Troponin increased | all_indication_supportive | 3 | 1.80 (0.58-5.58) | 0.61 | Ineligible |
| SG | Type 2 diabetes mellitus | all_indication_supportive | 3 | 0.38 (0.12-1.18) | 0.19 | Ineligible |
| SG | Vascular device infection | all_indication_supportive | 3 | 2.41 (0.78-7.47) | 0.71 | Ineligible |
| SG | Vascular device occlusion | all_indication_supportive | 3 | 33.62 (10.78-104.87) | 6.35 | Ineligible |
| SG | Wrong technique in product usage process | all_indication_supportive | 3 | 0.05 (0.02-0.16) | 0.03 | Ineligible |
| T-DM1 | Death | all_indication_supportive | 389 | 1.82 (1.64-2.02) | 1.62 | Eligible |
| T-DM1 | Disease progression | all_indication_supportive | 336 | 12.02 (10.76-13.42) | 10.29 | Eligible |
| T-DM1 | Fatigue | all_indication_supportive | 306 | 1.55 (1.39-1.75) | 1.38 | Eligible |
| T-DM1 | Platelet count decreased | all_indication_supportive | 290 | 11.76 (10.44-13.24) | 10.09 | Eligible |
| T-DM1 | Thrombocytopenia | all_indication_supportive | 262 | 10.55 (9.32-11.95) | 9.05 | Eligible |
| T-DM1 | Nausea | all_indication_supportive | 230 | 1.22 (1.07-1.39) | 1.09 | Eligible |
| T-DM1 | Pyrexia | all_indication_supportive | 200 | 2.50 (2.17-2.88) | 2.15 | Eligible |
| T-DM1 | Neuropathy peripheral | all_indication_supportive | 193 | 8.13 (7.04-9.39) | 6.93 | Eligible |
| T-DM1 | Myelosuppression | all_indication_supportive | 170 | 19.23 (16.50-22.42) | 16.27 | Eligible |
| T-DM1 | Epistaxis | all_indication_supportive | 164 | 9.02 (7.72-10.54) | 7.66 | Eligible |
| T-DM1 | Drug ineffective | all_indication_supportive | 162 | 0.45 (0.38-0.52) | 0.41 | Ineligible |
| T-DM1 | Metastases to central nervous system | all_indication_supportive | 162 | 52.27 (44.65-61.20) | 43.38 | Eligible |
| T-DM1 | No adverse event | all_indication_supportive | 159 | 3.36 (2.87-3.93) | 2.84 | Eligible |
| T-DM1 | Headache | all_indication_supportive | 152 | 0.98 (0.83-1.15) | 0.86 | Ineligible |
| T-DM1 | Dyspnoea | all_indication_supportive | 142 | 1.03 (0.87-1.22) | 0.90 | Ineligible |
| T-DM1 | Off label use | all_indication_supportive | 141 | 0.55 (0.47-0.65) | 0.49 | Ineligible |
| T-DM1 | Pneumonia | all_indication_supportive | 140 | 1.75 (1.48-2.07) | 1.49 | Eligible |
| T-DM1 | Pain | all_indication_supportive | 138 | 0.87 (0.73-1.03) | 0.76 | Ineligible |
| T-DM1 | Diarrhoea | all_indication_supportive | 121 | 0.73 (0.61-0.87) | 0.63 | Ineligible |
| T-DM1 | Asthenia | all_indication_supportive | 117 | 1.29 (1.08-1.55) | 1.10 | Eligible |
| T-DM1 | Aspartate aminotransferase increased | all_indication_supportive | 114 | 11.73 (9.74-14.13) | 9.77 | Eligible |
| T-DM1 | Anaemia | all_indication_supportive | 112 | 2.49 (2.07-3.00) | 2.07 | Eligible |
| T-DM1 | Vomiting | all_indication_supportive | 111 | 1.00 (0.83-1.21) | 0.86 | Ineligible |
| T-DM1 | Weight decreased | all_indication_supportive | 100 | 1.48 (1.21-1.80) | 1.24 | Eligible |
| T-DM1 | Constipation | all_indication_supportive | 94 | 1.74 (1.42-2.14) | 1.45 | Eligible |
| T-DM1 | Decreased appetite | all_indication_supportive | 94 | 1.59 (1.30-1.95) | 1.32 | Eligible |
| T-DM1 | Rash | all_indication_supportive | 94 | 0.86 (0.70-1.05) | 0.73 | Ineligible |
| T-DM1 | Alanine aminotransferase increased | all_indication_supportive | 93 | 7.90 (6.43-9.69) | 6.44 | Eligible |
| T-DM1 | Cough | all_indication_supportive | 90 | 1.25 (1.02-1.54) | 1.05 | Eligible |
| T-DM1 | Arthralgia | all_indication_supportive | 81 | 0.76 (0.61-0.95) | 0.64 | Ineligible |
| T-DM1 | Malaise | all_indication_supportive | 77 | 0.68 (0.54-0.85) | 0.57 | Ineligible |
| T-DM1 | Dizziness | all_indication_supportive | 74 | 0.62 (0.49-0.78) | 0.52 | Ineligible |
| T-DM1 | Myalgia | all_indication_supportive | 72 | 1.83 (1.45-2.31) | 1.48 | Eligible |
| T-DM1 | Chills | all_indication_supportive | 71 | 2.57 (2.03-3.25) | 2.04 | Eligible |
| T-DM1 | Hypoaesthesia | all_indication_supportive | 71 | 1.99 (1.58-2.52) | 1.60 | Eligible |
| T-DM1 | Interstitial lung disease | all_indication_supportive | 68 | 5.94 (4.68-7.55) | 4.61 | Eligible |
| T-DM1 | Pneumonitis | all_indication_supportive | 68 | 10.04 (7.90-12.75) | 8.01 | Eligible |
| T-DM1 | Blood bilirubin increased | all_indication_supportive | 65 | 12.07 (9.45-15.42) | 9.61 | Eligible |
| T-DM1 | Hepatic cirrhosis | all_indication_supportive | 64 | 14.41 (11.25-18.44) | 11.44 | Eligible |
| T-DM1 | Hepatic function abnormal | all_indication_supportive | 62 | 7.39 (5.75-9.50) | 5.74 | Eligible |
| T-DM1 | Abdominal pain | all_indication_supportive | 58 | 1.05 (0.81-1.36) | 0.85 | Ineligible |
| T-DM1 | Hepatic enzyme increased | all_indication_supportive | 56 | 3.42 (2.62-4.45) | 2.60 | Eligible |
| T-DM1 | Neutropenia | all_indication_supportive | 54 | 1.57 (1.20-2.05) | 1.24 | Eligible |
| T-DM1 | Pain in extremity | all_indication_supportive | 54 | 0.74 (0.56-0.97) | 0.60 | Ineligible |
| T-DM1 | Paraesthesia | all_indication_supportive | 54 | 1.46 (1.12-1.91) | 1.15 | Eligible |
| T-DM1 | Pleural effusion | all_indication_supportive | 54 | 3.90 (2.99-5.11) | 2.94 | Eligible |
| T-DM1 | Cardiac failure | all_indication_supportive | 53 | 2.80 (2.14-3.67) | 2.14 | Eligible |
| T-DM1 | Liver disorder | all_indication_supportive | 52 | 5.17 (3.93-6.79) | 3.83 | Eligible |
| T-DM1 | Ejection fraction decreased | all_indication_supportive | 51 | 14.16 (10.74-18.67) | 10.97 | Eligible |
| T-DM1 | Erythema | all_indication_supportive | 51 | 0.97 (0.74-1.28) | 0.77 | Ineligible |
| T-DM1 | White blood cell count decreased | all_indication_supportive | 51 | 1.87 (1.42-2.47) | 1.45 | Eligible |
| T-DM1 | Hypokalaemia | all_indication_supportive | 49 | 4.62 (3.49-6.12) | 3.40 | Eligible |
| T-DM1 | Infusion related reaction | all_indication_supportive | 49 | 2.96 (2.23-3.92) | 2.23 | Eligible |
| T-DM1 | Seizure | all_indication_supportive | 49 | 1.45 (1.10-1.93) | 1.13 | Eligible |
| T-DM1 | Blood alkaline phosphatase increased | all_indication_supportive | 48 | 10.90 (8.20-14.49) | 8.38 | Eligible |
| T-DM1 | Portal hypertension | all_indication_supportive | 48 | 71.75 (53.83-95.64) | 52.19 | Eligible |
| T-DM1 | Hepatotoxicity | all_indication_supportive | 47 | 8.77 (6.58-11.70) | 6.65 | Eligible |
| T-DM1 | Malignant neoplasm progression | all_indication_supportive | 47 | 1.73 (1.30-2.30) | 1.33 | Eligible |
| T-DM1 | Nasopharyngitis | all_indication_supportive | 47 | 0.96 (0.72-1.28) | 0.76 | Ineligible |
| T-DM1 | Vision blurred | all_indication_supportive | 47 | 1.47 (1.10-1.96) | 1.14 | Eligible |
| T-DM1 | Alopecia | all_indication_supportive | 46 | 0.87 (0.65-1.16) | 0.68 | Ineligible |
| T-DM1 | Ill-defined disorder | all_indication_supportive | 46 | 2.90 (2.17-3.88) | 2.17 | Eligible |
| T-DM1 | Intentional product use issue | all_indication_supportive | 46 | 1.76 (1.32-2.35) | 1.35 | Eligible |
| T-DM1 | Urinary tract infection | all_indication_supportive | 46 | 1.07 (0.80-1.43) | 0.84 | Ineligible |
| T-DM1 | Hypertension | all_indication_supportive | 44 | 0.87 (0.65-1.17) | 0.68 | Ineligible |
| T-DM1 | Metastases to liver | all_indication_supportive | 44 | 10.04 (7.46-13.51) | 7.61 | Eligible |
| T-DM1 | Back pain | all_indication_supportive | 43 | 0.75 (0.55-1.01) | 0.59 | Ineligible |
| T-DM1 | Pruritus | all_indication_supportive | 43 | 0.45 (0.33-0.61) | 0.36 | Ineligible |
| T-DM1 | Metastases to lung | all_indication_supportive | 42 | 14.40 (10.62-19.53) | 10.89 | Eligible |
| T-DM1 | Nodular regenerative hyperplasia | all_indication_supportive | 41 | 275.02 (199.69-378.76) | 160.56 | Eligible |
| T-DM1 | Abdominal pain upper | all_indication_supportive | 40 | 0.79 (0.58-1.08) | 0.62 | Ineligible |
| T-DM1 | Dry mouth | all_indication_supportive | 40 | 2.08 (1.52-2.84) | 1.55 | Eligible |
| T-DM1 | Fall | all_indication_supportive | 40 | 0.49 (0.36-0.67) | 0.39 | Ineligible |
| T-DM1 | Sepsis | all_indication_supportive | 39 | 1.48 (1.08-2.02) | 1.12 | Eligible |
| T-DM1 | Breast cancer | all_indication_supportive | 38 | 1.67 (1.21-2.30) | 1.25 | Eligible |
| T-DM1 | Respiratory failure | all_indication_supportive | 38 | 2.33 (1.69-3.21) | 1.71 | Eligible |
| T-DM1 | General physical health deterioration | all_indication_supportive | 37 | 1.30 (0.94-1.80) | 0.99 | Ineligible |
| T-DM1 | Peripheral swelling | all_indication_supportive | 37 | 0.81 (0.58-1.12) | 0.62 | Ineligible |
| T-DM1 | Transaminases increased | all_indication_supportive | 37 | 6.99 (5.06-9.67) | 4.94 | Eligible |
| T-DM1 | Gingival bleeding | all_indication_supportive | 36 | 10.86 (7.82-15.08) | 8.01 | Eligible |
| T-DM1 | Cardiotoxicity | all_indication_supportive | 35 | 15.16 (10.86-21.16) | 11.16 | Eligible |
| T-DM1 | Bone pain | all_indication_supportive | 34 | 2.37 (1.69-3.32) | 1.71 | Eligible |
| T-DM1 | Cerebral haemorrhage | all_indication_supportive | 34 | 3.99 (2.84-5.59) | 2.76 | Eligible |
| T-DM1 | Dehydration | all_indication_supportive | 34 | 1.12 (0.80-1.57) | 0.84 | Ineligible |
| T-DM1 | Haemoglobin decreased | all_indication_supportive | 34 | 1.43 (1.02-2.00) | 1.06 | Eligible |
| T-DM1 | Radiation necrosis | all_indication_supportive | 34 | 234.61 (165.45-332.69) | 134.27 | Eligible |
| T-DM1 | Neoplasm malignant | all_indication_supportive | 32 | 1.88 (1.33-2.67) | 1.37 | Eligible |
| T-DM1 | Gamma-glutamyltransferase increased | all_indication_supportive | 31 | 7.54 (5.30-10.74) | 5.17 | Eligible |
| T-DM1 | Hyponatraemia | all_indication_supportive | 31 | 2.41 (1.70-3.44) | 1.71 | Eligible |
| T-DM1 | Insomnia | all_indication_supportive | 31 | 0.49 (0.34-0.70) | 0.37 | Ineligible |
| T-DM1 | Metastases to bone | all_indication_supportive | 31 | 7.35 (5.16-10.46) | 5.01 | Eligible |
| T-DM1 | Muscle spasms | all_indication_supportive | 31 | 0.68 (0.48-0.97) | 0.52 | Ineligible |
| T-DM1 | Ascites | all_indication_supportive | 30 | 4.41 (3.08-6.31) | 2.94 | Eligible |
| T-DM1 | Haemorrhage | all_indication_supportive | 30 | 1.16 (0.81-1.66) | 0.85 | Ineligible |
| T-DM1 | Hypotension | all_indication_supportive | 30 | 0.63 (0.44-0.90) | 0.47 | Ineligible |
| T-DM1 | Infection | all_indication_supportive | 30 | 0.80 (0.56-1.15) | 0.60 | Ineligible |
| T-DM1 | Muscular weakness | all_indication_supportive | 30 | 1.11 (0.78-1.59) | 0.82 | Ineligible |
| T-DM1 | Neutrophil count decreased | all_indication_supportive | 30 | 2.96 (2.07-4.24) | 2.05 | Eligible |
| T-DM1 | Splenomegaly | all_indication_supportive | 30 | 10.97 (7.66-15.72) | 7.85 | Eligible |
| T-DM1 | Visual impairment | all_indication_supportive | 30 | 0.92 (0.65-1.32) | 0.69 | Ineligible |
| T-DM1 | COVID-19 | all_indication_supportive | 29 | 0.54 (0.37-0.77) | 0.41 | Ineligible |
| T-DM1 | Hypersensitivity | all_indication_supportive | 29 | 0.62 (0.43-0.89) | 0.47 | Ineligible |
| T-DM1 | Stomatitis | all_indication_supportive | 29 | 1.86 (1.29-2.68) | 1.33 | Eligible |
| T-DM1 | Blood pressure increased | all_indication_supportive | 28 | 0.75 (0.52-1.09) | 0.56 | Ineligible |
| T-DM1 | Cellulitis | all_indication_supportive | 28 | 2.30 (1.59-3.34) | 1.60 | Eligible |
| T-DM1 | Drug-induced liver injury | all_indication_supportive | 28 | 3.18 (2.19-4.61) | 2.15 | Eligible |
| T-DM1 | Hepatic failure | all_indication_supportive | 28 | 4.41 (3.04-6.39) | 2.89 | Eligible |
| T-DM1 | Liver function test abnormal | all_indication_supportive | 28 | 5.24 (3.62-7.60) | 3.41 | Eligible |
| T-DM1 | Liver function test increased | all_indication_supportive | 28 | 4.48 (3.09-6.49) | 2.93 | Eligible |
| T-DM1 | Product storage error | all_indication_supportive | 28 | 1.10 (0.76-1.60) | 0.81 | Ineligible |
| T-DM1 | Chest pain | all_indication_supportive | 27 | 0.66 (0.46-0.97) | 0.49 | Ineligible |
| T-DM1 | Gait disturbance | all_indication_supportive | 27 | 0.56 (0.38-0.81) | 0.42 | Ineligible |
| T-DM1 | Lung disorder | all_indication_supportive | 27 | 2.24 (1.53-3.27) | 1.55 | Eligible |
| T-DM1 | Pulmonary oedema | all_indication_supportive | 27 | 2.63 (1.80-3.84) | 1.80 | Eligible |
| T-DM1 | Red blood cell count decreased | all_indication_supportive | 27 | 3.84 (2.63-5.60) | 2.52 | Eligible |
| T-DM1 | Breast cancer metastatic | all_indication_supportive | 26 | 11.93 (8.11-17.55) | 8.34 | Eligible |
| T-DM1 | Cardiac disorder | all_indication_supportive | 26 | 1.21 (0.82-1.78) | 0.87 | Ineligible |
| T-DM1 | Dry eye | all_indication_supportive | 26 | 2.07 (1.41-3.05) | 1.44 | Eligible |
| T-DM1 | Chest discomfort | all_indication_supportive | 25 | 1.02 (0.69-1.51) | 0.74 | Ineligible |
| T-DM1 | Febrile neutropenia | all_indication_supportive | 25 | 1.53 (1.03-2.27) | 1.08 | Eligible |
| T-DM1 | Pericardial effusion | all_indication_supportive | 25 | 4.63 (3.12-6.86) | 2.93 | Eligible |
| T-DM1 | Cerebrovascular accident | all_indication_supportive | 24 | 0.67 (0.45-1.00) | 0.49 | Ineligible |
| T-DM1 | Contusion | all_indication_supportive | 24 | 1.00 (0.67-1.49) | 0.72 | Ineligible |
| T-DM1 | Gastrointestinal haemorrhage | all_indication_supportive | 24 | 1.16 (0.77-1.73) | 0.82 | Ineligible |
| T-DM1 | Intercepted product storage error | all_indication_supportive | 24 | 25.05 (16.75-37.46) | 17.03 | Eligible |
| T-DM1 | Lymphocyte count decreased | all_indication_supportive | 24 | 5.05 (3.38-7.54) | 3.15 | Eligible |
| T-DM1 | Metastasis | all_indication_supportive | 24 | 13.60 (9.10-20.33) | 9.39 | Eligible |
| T-DM1 | Neoplasm progression | all_indication_supportive | 24 | 2.47 (1.65-3.69) | 1.66 | Eligible |
| T-DM1 | Swelling | all_indication_supportive | 24 | 0.99 (0.66-1.48) | 0.71 | Ineligible |
| T-DM1 | Acute kidney injury | all_indication_supportive | 23 | 0.50 (0.34-0.76) | 0.37 | Ineligible |
| T-DM1 | Blood lactate dehydrogenase increased | all_indication_supportive | 23 | 7.83 (5.19-11.79) | 4.96 | Eligible |
| T-DM1 | Leukopenia | all_indication_supportive | 23 | 1.98 (1.31-2.98) | 1.34 | Eligible |
| T-DM1 | Weight increased | all_indication_supportive | 23 | 0.43 (0.28-0.64) | 0.32 | Ineligible |
| T-DM1 | Blood potassium decreased | all_indication_supportive | 22 | 3.19 (2.10-4.84) | 2.04 | Eligible |
| T-DM1 | Hyperbilirubinaemia | all_indication_supportive | 22 | 9.03 (5.94-13.73) | 5.83 | Eligible |
| T-DM1 | Oxygen saturation decreased | all_indication_supportive | 22 | 1.56 (1.03-2.38) | 1.07 | Eligible |
| T-DM1 | Brain oedema | all_indication_supportive | 21 | 7.57 (4.93-11.62) | 4.62 | Eligible |
| T-DM1 | Drug intolerance | all_indication_supportive | 21 | 0.78 (0.51-1.19) | 0.55 | Ineligible |
| T-DM1 | Product preparation error | all_indication_supportive | 21 | 3.38 (2.20-5.18) | 2.12 | Eligible |
| T-DM1 | Tachycardia | all_indication_supportive | 21 | 1.04 (0.67-1.59) | 0.73 | Ineligible |
| T-DM1 | Urticaria | all_indication_supportive | 21 | 0.52 (0.34-0.80) | 0.38 | Ineligible |
| T-DM1 | Varices oesophageal | all_indication_supportive | 21 | 30.84 (20.05-47.44) | 20.18 | Eligible |
| T-DM1 | Confusional state | all_indication_supportive | 20 | 0.53 (0.34-0.82) | 0.38 | Ineligible |
| T-DM1 | Depression | all_indication_supportive | 20 | 0.39 (0.25-0.61) | 0.28 | Ineligible |
| T-DM1 | Hepatitis | all_indication_supportive | 20 | 3.64 (2.34-5.64) | 2.23 | Eligible |
| T-DM1 | Jaundice | all_indication_supportive | 20 | 3.72 (2.39-5.76) | 2.27 | Eligible |
| T-DM1 | Lacrimation increased | all_indication_supportive | 20 | 2.60 (1.68-4.04) | 1.67 | Eligible |
| T-DM1 | Musculoskeletal pain | all_indication_supportive | 20 | 1.56 (1.01-2.43) | 1.06 | Eligible |
| T-DM1 | Pulmonary embolism | all_indication_supportive | 20 | 0.99 (0.64-1.53) | 0.69 | Ineligible |
| T-DM1 | Dyspepsia | all_indication_supportive | 19 | 0.82 (0.52-1.29) | 0.57 | Ineligible |
| T-DM1 | Extravasation | all_indication_supportive | 19 | 21.22 (13.50-33.34) | 13.82 | Eligible |
| T-DM1 | Loss of consciousness | all_indication_supportive | 19 | 0.68 (0.43-1.06) | 0.48 | Ineligible |
| T-DM1 | Memory impairment | all_indication_supportive | 19 | 0.53 (0.34-0.84) | 0.38 | Ineligible |
| T-DM1 | Pulmonary fibrosis | all_indication_supportive | 19 | 4.20 (2.68-6.59) | 2.48 | Eligible |
| T-DM1 | Renal failure | all_indication_supportive | 19 | 0.62 (0.39-0.97) | 0.44 | Ineligible |
| T-DM1 | Somnolence | all_indication_supportive | 19 | 0.39 (0.25-0.62) | 0.28 | Ineligible |
| T-DM1 | Abdominal distension | all_indication_supportive | 18 | 0.71 (0.45-1.13) | 0.50 | Ineligible |
| T-DM1 | Intercepted medication error | all_indication_supportive | 18 | 13.29 (8.36-21.13) | 8.59 | Eligible |
| T-DM1 | Liver injury | all_indication_supportive | 18 | 2.93 (1.84-4.65) | 1.79 | Eligible |
| T-DM1 | Lymphadenopathy | all_indication_supportive | 18 | 2.19 (1.38-3.48) | 1.39 | Eligible |
| T-DM1 | Palpitations | all_indication_supportive | 18 | 0.66 (0.41-1.05) | 0.46 | Ineligible |
| T-DM1 | Pulmonary arterial hypertension | all_indication_supportive | 18 | 4.52 (2.85-7.19) | 2.60 | Eligible |
| T-DM1 | Recurrent cancer | all_indication_supportive | 18 | 16.93 (10.64-26.92) | 10.97 | Eligible |
| T-DM1 | Spider naevus | all_indication_supportive | 18 | 581.63 (351.74-961.78) | 172.11 | Eligible |
| T-DM1 | Tremor | all_indication_supportive | 18 | 0.47 (0.30-0.75) | 0.34 | Ineligible |
| T-DM1 | Anxiety | all_indication_supportive | 17 | 0.25 (0.15-0.40) | 0.18 | Ineligible |
| T-DM1 | Blood creatinine increased | all_indication_supportive | 17 | 1.19 (0.74-1.92) | 0.80 | Ineligible |
| T-DM1 | Breast cancer recurrent | all_indication_supportive | 17 | 23.63 (14.65-38.10) | 14.92 | Eligible |
| T-DM1 | Dysgeusia | all_indication_supportive | 17 | 1.00 (0.62-1.61) | 0.68 | Ineligible |
| T-DM1 | Hypoxia | all_indication_supportive | 17 | 2.08 (1.29-3.35) | 1.31 | Eligible |
| T-DM1 | Metastases to lymph nodes | all_indication_supportive | 17 | 10.21 (6.34-16.45) | 6.24 | Eligible |
| T-DM1 | Metastatic neoplasm | all_indication_supportive | 17 | 17.68 (10.97-28.50) | 11.29 | Eligible |
| T-DM1 | Neoplasm | all_indication_supportive | 17 | 5.69 (3.54-9.17) | 3.15 | Eligible |
| T-DM1 | Neuralgia | all_indication_supportive | 17 | 2.71 (1.69-4.37) | 1.66 | Eligible |
| T-DM1 | Oedema peripheral | all_indication_supportive | 17 | 0.78 (0.48-1.25) | 0.53 | Ineligible |
| T-DM1 | Pancreatitis | all_indication_supportive | 17 | 1.56 (0.97-2.51) | 1.02 | Ineligible |
| T-DM1 | Renal impairment | all_indication_supportive | 17 | 0.81 (0.50-1.31) | 0.56 | Ineligible |
| T-DM1 | Tumour haemorrhage | all_indication_supportive | 17 | 24.36 (15.10-39.28) | 15.36 | Eligible |
| T-DM1 | Blood glucose increased | all_indication_supportive | 16 | 0.37 (0.22-0.60) | 0.26 | Ineligible |
| T-DM1 | Cerebral disorder | all_indication_supportive | 16 | 8.14 (4.98-13.30) | 4.54 | Eligible |
| T-DM1 | Cholestasis | all_indication_supportive | 16 | 3.65 (2.23-5.96) | 2.09 | Eligible |
| T-DM1 | Dysphagia | all_indication_supportive | 16 | 0.71 (0.43-1.16) | 0.49 | Ineligible |
| T-DM1 | Heart rate increased | all_indication_supportive | 16 | 0.70 (0.43-1.15) | 0.48 | Ineligible |
| T-DM1 | Mucosal inflammation | all_indication_supportive | 16 | 2.59 (1.59-4.24) | 1.57 | Eligible |
| T-DM1 | Toxicity to various agents | all_indication_supportive | 16 | 0.34 (0.21-0.56) | 0.24 | Ineligible |
| T-DM1 | Blindness | all_indication_supportive | 15 | 1.52 (0.92-2.52) | 0.97 | Ineligible |
| T-DM1 | Device related infection | all_indication_supportive | 15 | 3.73 (2.25-6.20) | 2.08 | Eligible |
| T-DM1 | Dry skin | all_indication_supportive | 15 | 0.41 (0.25-0.68) | 0.28 | Ineligible |
| T-DM1 | Feeling abnormal | all_indication_supportive | 15 | 0.25 (0.15-0.42) | 0.18 | Ineligible |
| T-DM1 | Flushing | all_indication_supportive | 15 | 0.70 (0.42-1.17) | 0.48 | Ineligible |
| T-DM1 | Haematuria | all_indication_supportive | 15 | 1.84 (1.11-3.06) | 1.15 | Eligible |
| T-DM1 | Hepatopulmonary syndrome | all_indication_supportive | 15 | 439.97 (256.28-755.33) | 133.98 | Eligible |
| T-DM1 | Medication error | all_indication_supportive | 15 | 1.43 (0.86-2.37) | 0.91 | Ineligible |
| T-DM1 | Palmar-plantar erythrodysaesthesia syndrome | all_indication_supportive | 15 | 2.57 (1.55-4.27) | 1.53 | Eligible |
| T-DM1 | Pulmonary hypertension | all_indication_supportive | 15 | 3.14 (1.89-5.22) | 1.81 | Eligible |
| T-DM1 | Telangiectasia | all_indication_supportive | 15 | 67.35 (40.36-112.40) | 37.26 | Eligible |
| T-DM1 | Balance disorder | all_indication_supportive | 14 | 0.66 (0.39-1.12) | 0.45 | Ineligible |
| T-DM1 | Condition aggravated | all_indication_supportive | 14 | 0.18 (0.10-0.30) | 0.12 | Ineligible |
| T-DM1 | Drug resistance | all_indication_supportive | 14 | 1.96 (1.16-3.32) | 1.19 | Eligible |
| T-DM1 | Epilepsy | all_indication_supportive | 14 | 1.90 (1.13-3.21) | 1.16 | Eligible |
| T-DM1 | Hepatic encephalopathy | all_indication_supportive | 14 | 6.49 (3.84-10.97) | 3.30 | Eligible |
| T-DM1 | Hypercalcaemia | all_indication_supportive | 14 | 4.81 (2.85-8.13) | 2.51 | Eligible |
| T-DM1 | Inflammation | all_indication_supportive | 14 | 1.07 (0.63-1.80) | 0.69 | Ineligible |
| T-DM1 | Influenza like illness | all_indication_supportive | 14 | 0.73 (0.43-1.23) | 0.49 | Ineligible |
| T-DM1 | Maternal exposure during pregnancy | all_indication_supportive | 14 | 0.57 (0.34-0.97) | 0.39 | Ineligible |
| T-DM1 | Rhinorrhoea | all_indication_supportive | 14 | 0.82 (0.49-1.39) | 0.54 | Ineligible |
| T-DM1 | Venoocclusive liver disease | all_indication_supportive | 14 | 11.81 (6.98-19.97) | 6.95 | Eligible |
| T-DM1 | Acute respiratory distress syndrome | all_indication_supportive | 13 | 3.39 (1.97-5.84) | 1.83 | Eligible |
| T-DM1 | Blister | all_indication_supportive | 13 | 0.93 (0.54-1.59) | 0.60 | Ineligible |
| T-DM1 | Cardiac failure congestive | all_indication_supportive | 13 | 0.77 (0.45-1.32) | 0.50 | Ineligible |
| T-DM1 | Colitis | all_indication_supportive | 13 | 1.35 (0.78-2.33) | 0.84 | Ineligible |
| T-DM1 | Gastrointestinal disorder | all_indication_supportive | 13 | 0.57 (0.33-0.99) | 0.38 | Ineligible |
| T-DM1 | Haemoptysis | all_indication_supportive | 13 | 1.95 (1.13-3.36) | 1.16 | Eligible |
| T-DM1 | Hot flush | all_indication_supportive | 13 | 0.67 (0.39-1.16) | 0.44 | Ineligible |
| T-DM1 | Monocyte count increased | all_indication_supportive | 13 | 14.29 (8.28-24.66) | 8.45 | Eligible |
| T-DM1 | Non-cirrhotic portal hypertension | all_indication_supportive | 13 | 137.57 (78.91-239.84) | 61.72 | Eligible |
| T-DM1 | Onychoclasis | all_indication_supportive | 13 | 7.04 (4.08-12.14) | 3.47 | Eligible |
| T-DM1 | Pulmonary toxicity | all_indication_supportive | 13 | 7.31 (4.24-12.60) | 3.62 | Eligible |
| T-DM1 | Radiation pneumonitis | all_indication_supportive | 13 | 23.52 (13.62-40.62) | 13.84 | Eligible |
| T-DM1 | Rectal haemorrhage | all_indication_supportive | 13 | 1.28 (0.74-2.21) | 0.81 | Ineligible |
| T-DM1 | Vertigo | all_indication_supportive | 13 | 0.87 (0.51-1.50) | 0.57 | Ineligible |
| T-DM1 | Wrong technique in product usage process | all_indication_supportive | 13 | 0.21 (0.12-0.37) | 0.15 | Ineligible |
| T-DM1 | Anaphylactic reaction | all_indication_supportive | 12 | 0.91 (0.52-1.60) | 0.58 | Ineligible |
| T-DM1 | Brain neoplasm | all_indication_supportive | 12 | 5.23 (2.96-9.21) | 2.52 | Eligible |
| T-DM1 | Cardiac dysfunction | all_indication_supportive | 12 | 11.24 (6.37-19.83) | 6.08 | Eligible |
| T-DM1 | Cholecystitis | all_indication_supportive | 12 | 4.84 (2.75-8.54) | 2.37 | Eligible |
| T-DM1 | Cytopenia | all_indication_supportive | 12 | 3.49 (1.98-6.15) | 1.82 | Eligible |
| T-DM1 | Disease recurrence | all_indication_supportive | 12 | 0.92 (0.52-1.61) | 0.58 | Ineligible |
| T-DM1 | Gastritis | all_indication_supportive | 12 | 2.07 (1.18-3.65) | 1.20 | Eligible |
| T-DM1 | Haematocrit decreased | all_indication_supportive | 12 | 3.13 (1.78-5.52) | 1.67 | Eligible |
| T-DM1 | Herpes zoster | all_indication_supportive | 12 | 0.82 (0.46-1.44) | 0.53 | Ineligible |
| T-DM1 | Illness | all_indication_supportive | 12 | 0.42 (0.24-0.74) | 0.28 | Ineligible |
| T-DM1 | Influenza | all_indication_supportive | 12 | 0.40 (0.23-0.71) | 0.27 | Ineligible |
| T-DM1 | Nervous system disorder | all_indication_supportive | 12 | 2.29 (1.30-4.03) | 1.30 | Eligible |
| T-DM1 | Red cell distribution width increased | all_indication_supportive | 12 | 10.64 (6.03-18.77) | 5.63 | Eligible |
| T-DM1 | Skin toxicity | all_indication_supportive | 12 | 8.42 (4.78-14.85) | 4.10 | Eligible |
| T-DM1 | Thrombosis | all_indication_supportive | 12 | 0.63 (0.36-1.11) | 0.41 | Ineligible |
| T-DM1 | Amnesia | all_indication_supportive | 11 | 0.77 (0.43-1.40) | 0.49 | Ineligible |
| T-DM1 | Body temperature increased | all_indication_supportive | 11 | 2.42 (1.34-4.38) | 1.32 | Eligible |
| T-DM1 | Burning sensation | all_indication_supportive | 11 | 0.66 (0.36-1.19) | 0.42 | Ineligible |
| T-DM1 | Cataract | all_indication_supportive | 11 | 0.73 (0.40-1.32) | 0.47 | Ineligible |
| T-DM1 | Corneal disorder | all_indication_supportive | 11 | 22.41 (12.38-40.57) | 12.56 | Eligible |
| T-DM1 | Dyspnoea exertional | all_indication_supportive | 11 | 1.14 (0.63-2.06) | 0.70 | Ineligible |
| T-DM1 | Encephalopathy | all_indication_supportive | 11 | 1.95 (1.08-3.53) | 1.11 | Eligible |
| T-DM1 | Gastric haemorrhage | all_indication_supportive | 11 | 4.20 (2.32-7.59) | 2.04 | Eligible |
| T-DM1 | Hepatic cytolysis | all_indication_supportive | 11 | 3.26 (1.80-5.88) | 1.67 | Eligible |
| T-DM1 | Hepatic steatosis | all_indication_supportive | 11 | 2.72 (1.50-4.91) | 1.45 | Eligible |
| T-DM1 | Hyperglycaemia | all_indication_supportive | 11 | 1.38 (0.77-2.50) | 0.83 | Ineligible |
| T-DM1 | Hypertransaminasaemia | all_indication_supportive | 11 | 5.89 (3.26-10.65) | 2.69 | Eligible |
| T-DM1 | Lower respiratory tract infection | all_indication_supportive | 11 | 0.91 (0.50-1.64) | 0.57 | Ineligible |
| T-DM1 | Lung infection | all_indication_supportive | 11 | 2.93 (1.62-5.30) | 1.54 | Eligible |
| T-DM1 | Metastases to spine | all_indication_supportive | 11 | 13.14 (7.26-23.77) | 7.16 | Eligible |
| T-DM1 | Oropharyngeal pain | all_indication_supportive | 11 | 0.45 (0.25-0.82) | 0.30 | Ineligible |
| T-DM1 | Polyneuropathy | all_indication_supportive | 11 | 3.71 (2.05-6.70) | 1.85 | Eligible |
| T-DM1 | Septic shock | all_indication_supportive | 11 | 1.06 (0.59-1.92) | 0.66 | Ineligible |
| T-DM1 | Speech disorder | all_indication_supportive | 11 | 0.91 (0.50-1.64) | 0.57 | Ineligible |
| T-DM1 | Upper respiratory tract infection | all_indication_supportive | 11 | 0.94 (0.52-1.70) | 0.59 | Ineligible |
| T-DM1 | White blood cell count increased | all_indication_supportive | 11 | 1.30 (0.72-2.35) | 0.78 | Ineligible |
| T-DM1 | Abdominal discomfort | all_indication_supportive | 10 | 0.22 (0.12-0.42) | 0.15 | Ineligible |
| T-DM1 | Anaphylactic shock | all_indication_supportive | 10 | 1.73 (0.93-3.21) | 0.98 | Ineligible |
| T-DM1 | Blood albumin decreased | all_indication_supportive | 10 | 6.74 (3.62-12.53) | 2.89 | Eligible |
| T-DM1 | Blood sodium decreased | all_indication_supportive | 10 | 2.50 (1.34-4.64) | 1.31 | Eligible |
| T-DM1 | Brain neoplasm malignant | all_indication_supportive | 10 | 7.92 (4.26-14.75) | 3.43 | Eligible |
| T-DM1 | Breast disorder | all_indication_supportive | 10 | 21.11 (11.33-39.34) | 11.49 | Eligible |
| T-DM1 | C-reactive protein increased | all_indication_supportive | 10 | 1.13 (0.61-2.11) | 0.68 | Ineligible |
| T-DM1 | Cardiac arrest | all_indication_supportive | 10 | 0.57 (0.31-1.06) | 0.36 | Ineligible |
| T-DM1 | Cognitive disorder | all_indication_supportive | 10 | 0.85 (0.46-1.59) | 0.53 | Ineligible |
| T-DM1 | Discomfort | all_indication_supportive | 10 | 0.61 (0.33-1.14) | 0.39 | Ineligible |
| T-DM1 | Drug hypersensitivity | all_indication_supportive | 10 | 0.18 (0.10-0.34) | 0.12 | Ineligible |
| T-DM1 | Feeding disorder | all_indication_supportive | 10 | 1.78 (0.96-3.31) | 1.00 | Ineligible |
| T-DM1 | Gastrooesophageal reflux disease | all_indication_supportive | 10 | 0.53 (0.29-0.99) | 0.34 | Ineligible |
| T-DM1 | Hyperhidrosis | all_indication_supportive | 10 | 0.33 (0.18-0.62) | 0.22 | Ineligible |
| T-DM1 | Incorrect product storage | all_indication_supportive | 10 | 2.48 (1.33-4.61) | 1.31 | Eligible |
| T-DM1 | Migraine | all_indication_supportive | 10 | 0.41 (0.22-0.77) | 0.27 | Ineligible |
| T-DM1 | Mouth haemorrhage | all_indication_supportive | 10 | 5.76 (3.10-10.72) | 2.51 | Eligible |
| T-DM1 | Neurotoxicity | all_indication_supportive | 10 | 2.24 (1.20-4.16) | 1.21 | Eligible |
| T-DM1 | Ocular hyperaemia | all_indication_supportive | 10 | 0.85 (0.46-1.58) | 0.53 | Ineligible |
| T-DM1 | Oedema | all_indication_supportive | 10 | 0.78 (0.42-1.46) | 0.49 | Ineligible |
| T-DM1 | Pancytopenia | all_indication_supportive | 10 | 0.78 (0.42-1.44) | 0.48 | Ineligible |
| T-DM1 | Product preparation issue | all_indication_supportive | 10 | 6.44 (3.46-11.99) | 2.77 | Eligible |
| T-DM1 | Radiation skin injury | all_indication_supportive | 10 | 39.48 (21.15-73.70) | 20.32 | Eligible |
| T-DM1 | Stress | all_indication_supportive | 10 | 0.56 (0.30-1.03) | 0.36 | Ineligible |
| T-DM1 | Syncope | all_indication_supportive | 10 | 0.43 (0.23-0.81) | 0.28 | Ineligible |
| T-DM1 | Tumour marker increased | all_indication_supportive | 10 | 7.57 (4.07-14.10) | 3.26 | Eligible |
| T-DM1 | Visual acuity reduced | all_indication_supportive | 10 | 1.51 (0.81-2.81) | 0.87 | Ineligible |
| T-DM1 | Bone disorder | all_indication_supportive | 9 | 2.32 (1.21-4.46) | 1.20 | Ineligible |
| T-DM1 | Bone marrow failure | all_indication_supportive | 9 | 1.85 (0.96-3.55) | 1.00 | Ineligible |
| T-DM1 | Breast mass | all_indication_supportive | 9 | 5.99 (3.11-11.52) | 2.46 | Ineligible |
| T-DM1 | Breast neoplasm | all_indication_supportive | 9 | 37.42 (19.39-72.24) | 18.62 | Ineligible |
| T-DM1 | Cardiomyopathy | all_indication_supportive | 9 | 2.85 (1.48-5.48) | 1.40 | Ineligible |
| T-DM1 | Eye disorder | all_indication_supportive | 9 | 1.11 (0.58-2.13) | 0.65 | Ineligible |
| T-DM1 | Femur fracture | all_indication_supportive | 9 | 1.32 (0.69-2.54) | 0.76 | Ineligible |
| T-DM1 | Gastric ulcer | all_indication_supportive | 9 | 2.12 (1.10-4.07) | 1.12 | Ineligible |
| T-DM1 | Haemorrhage intracranial | all_indication_supportive | 9 | 2.46 (1.28-4.73) | 1.25 | Ineligible |
| T-DM1 | Hypoalbuminaemia | all_indication_supportive | 9 | 5.28 (2.74-10.15) | 2.22 | Ineligible |
| T-DM1 | Lung infiltration | all_indication_supportive | 9 | 6.00 (3.12-11.55) | 2.46 | Ineligible |
| T-DM1 | Lymphoedema | all_indication_supportive | 9 | 5.17 (2.69-9.95) | 2.18 | Ineligible |
| T-DM1 | Mean platelet volume decreased | all_indication_supportive | 9 | 40.88 (21.17-78.95) | 20.09 | Ineligible |
| T-DM1 | Metastases to skin | all_indication_supportive | 9 | 33.30 (17.26-64.26) | 16.81 | Ineligible |
| T-DM1 | Neck pain | all_indication_supportive | 9 | 0.66 (0.34-1.26) | 0.41 | Ineligible |
| T-DM1 | Osteonecrosis of jaw | all_indication_supportive | 9 | 1.44 (0.75-2.77) | 0.82 | Ineligible |
| T-DM1 | Osteoporosis | all_indication_supportive | 9 | 0.85 (0.44-1.63) | 0.51 | Ineligible |
| T-DM1 | Peripheral sensory neuropathy | all_indication_supportive | 9 | 6.27 (3.26-12.07) | 2.56 | Ineligible |
| T-DM1 | Pulmonary mass | all_indication_supportive | 9 | 2.16 (1.12-4.15) | 1.13 | Ineligible |
| T-DM1 | Sleep disorder | all_indication_supportive | 9 | 0.51 (0.26-0.98) | 0.32 | Ineligible |
| T-DM1 | Upper gastrointestinal haemorrhage | all_indication_supportive | 9 | 2.05 (1.07-3.94) | 1.09 | Ineligible |
| T-DM1 | Arrhythmia | all_indication_supportive | 8 | 0.78 (0.39-1.56) | 0.46 | Ineligible |
| T-DM1 | Blood creatinine decreased | all_indication_supportive | 8 | 10.58 (5.28-21.20) | 4.21 | Ineligible |
| T-DM1 | Blood magnesium decreased | all_indication_supportive | 8 | 3.69 (1.84-7.39) | 1.61 | Ineligible |
| T-DM1 | Blood pressure decreased | all_indication_supportive | 8 | 0.53 (0.26-1.05) | 0.32 | Ineligible |
| T-DM1 | Blood pressure diastolic decreased | all_indication_supportive | 8 | 4.52 (2.26-9.05) | 1.86 | Ineligible |
| T-DM1 | Carcinoembryonic antigen increased | all_indication_supportive | 8 | 16.36 (8.16-32.79) | 7.96 | Ineligible |
| T-DM1 | Central nervous system lesion | all_indication_supportive | 8 | 2.22 (1.11-4.45) | 1.11 | Ineligible |
| T-DM1 | Conjunctivitis | all_indication_supportive | 8 | 1.70 (0.85-3.40) | 0.90 | Ineligible |
| T-DM1 | Diabetes mellitus | all_indication_supportive | 8 | 0.49 (0.25-0.99) | 0.30 | Ineligible |
| T-DM1 | Drug interaction | all_indication_supportive | 8 | 0.22 (0.11-0.44) | 0.14 | Ineligible |
| T-DM1 | Eye irritation | all_indication_supportive | 8 | 0.57 (0.28-1.13) | 0.35 | Ineligible |
| T-DM1 | Focal nodular hyperplasia | all_indication_supportive | 8 | 76.02 (37.69-153.34) | 31.33 | Ineligible |
| T-DM1 | Fracture | all_indication_supportive | 8 | 1.63 (0.82-3.26) | 0.87 | Ineligible |
| T-DM1 | Haematemesis | all_indication_supportive | 8 | 1.41 (0.71-2.82) | 0.78 | Ineligible |
| T-DM1 | Haemolysis | all_indication_supportive | 8 | 4.46 (2.23-8.92) | 1.84 | Ineligible |
| T-DM1 | Hemiplegia | all_indication_supportive | 8 | 4.33 (2.16-8.67) | 1.80 | Ineligible |
| T-DM1 | Hepatic fibrosis | all_indication_supportive | 8 | 11.04 (5.51-22.11) | 4.47 | Ineligible |
| T-DM1 | Hyperaesthesia | all_indication_supportive | 8 | 4.05 (2.02-8.11) | 1.72 | Ineligible |
| T-DM1 | Hypocalcaemia | all_indication_supportive | 8 | 1.78 (0.89-3.56) | 0.94 | Ineligible |
| T-DM1 | Impaired healing | all_indication_supportive | 8 | 1.03 (0.52-2.07) | 0.60 | Ineligible |
| T-DM1 | Lung neoplasm | all_indication_supportive | 8 | 13.61 (6.79-27.26) | 6.17 | Ineligible |
| T-DM1 | Lymphangiosis carcinomatosa | all_indication_supportive | 8 | 33.39 (16.63-67.04) | 16.06 | Ineligible |
| T-DM1 | Melaena | all_indication_supportive | 8 | 1.55 (0.78-3.11) | 0.84 | Ineligible |
| T-DM1 | Metastases to meninges | all_indication_supportive | 8 | 13.73 (6.85-27.51) | 6.25 | Ineligible |
| T-DM1 | Musculoskeletal stiffness | all_indication_supportive | 8 | 0.35 (0.18-0.71) | 0.22 | Ineligible |
| T-DM1 | Oesophageal varices haemorrhage | all_indication_supportive | 8 | 15.48 (7.72-31.02) | 7.41 | Ineligible |
| T-DM1 | Pancreatitis acute | all_indication_supportive | 8 | 1.61 (0.80-3.22) | 0.86 | Ineligible |
| T-DM1 | Petechiae | all_indication_supportive | 8 | 3.48 (1.74-6.96) | 1.54 | Ineligible |
| T-DM1 | Respiratory tract infection | all_indication_supportive | 8 | 1.19 (0.60-2.38) | 0.67 | Ineligible |
| T-DM1 | Skin exfoliation | all_indication_supportive | 8 | 0.36 (0.18-0.72) | 0.22 | Ineligible |
| T-DM1 | Taste disorder | all_indication_supportive | 8 | 1.38 (0.69-2.76) | 0.76 | Ineligible |
| T-DM1 | Tumour lysis syndrome | all_indication_supportive | 8 | 3.62 (1.81-7.25) | 1.59 | Ineligible |
| T-DM1 | Unevaluable event | all_indication_supportive | 8 | 0.42 (0.21-0.84) | 0.26 | Ineligible |
| T-DM1 | Acute hepatic failure | all_indication_supportive | 7 | 2.08 (0.99-4.36) | 1.01 | Ineligible |
| T-DM1 | Aphasia | all_indication_supportive | 7 | 1.00 (0.47-2.09) | 0.56 | Ineligible |
| T-DM1 | Asthma | all_indication_supportive | 7 | 0.25 (0.12-0.52) | 0.15 | Ineligible |
| T-DM1 | Breast pain | all_indication_supportive | 7 | 2.83 (1.35-5.95) | 1.26 | Ineligible |
| T-DM1 | Bronchitis | all_indication_supportive | 7 | 0.38 (0.18-0.79) | 0.23 | Ineligible |
| T-DM1 | Cardio-respiratory arrest | all_indication_supportive | 7 | 0.81 (0.39-1.70) | 0.47 | Ineligible |
| T-DM1 | Cystitis | all_indication_supportive | 7 | 0.89 (0.42-1.87) | 0.51 | Ineligible |
| T-DM1 | Disseminated intravascular coagulation | all_indication_supportive | 7 | 2.57 (1.23-5.40) | 1.18 | Ineligible |
| T-DM1 | Ear pain | all_indication_supportive | 7 | 1.46 (0.70-3.07) | 0.77 | Ineligible |
| T-DM1 | Eating disorder | all_indication_supportive | 7 | 1.32 (0.63-2.78) | 0.71 | Ineligible |
| T-DM1 | Eye pain | all_indication_supportive | 7 | 0.50 (0.24-1.05) | 0.30 | Ineligible |
| T-DM1 | Eye pruritus | all_indication_supportive | 7 | 0.82 (0.39-1.72) | 0.47 | Ineligible |
| T-DM1 | Gastroenteritis | all_indication_supportive | 7 | 2.10 (1.00-4.41) | 1.02 | Ineligible |
| T-DM1 | Gastrointestinal toxicity | all_indication_supportive | 7 | 5.81 (2.77-12.20) | 2.08 | Ineligible |
| T-DM1 | Hepatic pain | all_indication_supportive | 7 | 6.67 (3.17-14.00) | 2.31 | Ineligible |
| T-DM1 | Hepatocellular injury | all_indication_supportive | 7 | 1.77 (0.84-3.71) | 0.89 | Ineligible |
| T-DM1 | Hepatomegaly | all_indication_supportive | 7 | 3.55 (1.69-7.46) | 1.48 | Ineligible |
| T-DM1 | Hyperpyrexia | all_indication_supportive | 7 | 6.69 (3.18-14.05) | 2.32 | Ineligible |
| T-DM1 | Intracranial tumour haemorrhage | all_indication_supportive | 7 | 75.75 (35.78-160.36) | 29.04 | Ineligible |
| T-DM1 | Joint swelling | all_indication_supportive | 7 | 0.22 (0.11-0.46) | 0.14 | Ineligible |
| T-DM1 | Mastectomy | all_indication_supportive | 7 | 17.55 (8.35-36.91) | 7.98 | Ineligible |
| T-DM1 | Mean cell haemoglobin concentration decreased | all_indication_supportive | 7 | 15.00 (7.13-31.53) | 6.39 | Ineligible |
| T-DM1 | Multiple organ dysfunction syndrome | all_indication_supportive | 7 | 0.87 (0.41-1.82) | 0.49 | Ineligible |
| T-DM1 | Onycholysis | all_indication_supportive | 7 | 17.76 (8.45-37.35) | 8.10 | Ineligible |
| T-DM1 | Pneumocystis jirovecii pneumonia | all_indication_supportive | 7 | 2.36 (1.13-4.96) | 1.11 | Ineligible |
| T-DM1 | Pneumothorax | all_indication_supportive | 7 | 1.79 (0.85-3.75) | 0.90 | Ineligible |
| T-DM1 | Product administration error | all_indication_supportive | 7 | 1.20 (0.57-2.52) | 0.65 | Ineligible |
| T-DM1 | Respiratory distress | all_indication_supportive | 7 | 1.15 (0.55-2.42) | 0.63 | Ineligible |
| T-DM1 | Retinal detachment | all_indication_supportive | 7 | 3.24 (1.54-6.80) | 1.39 | Ineligible |
| T-DM1 | Skin disorder | all_indication_supportive | 7 | 0.82 (0.39-1.72) | 0.47 | Ineligible |
| T-DM1 | Suspected counterfeit product | all_indication_supportive | 7 | 6.15 (2.93-12.93) | 2.17 | Ineligible |
| T-DM1 | Tenderness | all_indication_supportive | 7 | 2.70 (1.29-5.67) | 1.22 | Ineligible |
| T-DM1 | Throat tightness | all_indication_supportive | 7 | 1.14 (0.54-2.38) | 0.62 | Ineligible |
| T-DM1 | Urinary retention | all_indication_supportive | 7 | 0.88 (0.42-1.85) | 0.50 | Ineligible |
| T-DM1 | Vaginal haemorrhage | all_indication_supportive | 7 | 0.69 (0.33-1.46) | 0.41 | Ineligible |
| T-DM1 | Vascular device infection | all_indication_supportive | 7 | 5.32 (2.54-11.18) | 1.95 | Ineligible |
| T-DM1 | Viral infection | all_indication_supportive | 7 | 0.86 (0.41-1.80) | 0.49 | Ineligible |
| T-DM1 | Administration site extravasation | all_indication_supportive | 6 | 21.18 (9.49-47.30) | 9.00 | Ineligible |
| T-DM1 | Adverse drug reaction | all_indication_supportive | 6 | 0.24 (0.11-0.54) | 0.15 | Ineligible |
| T-DM1 | Ammonia increased | all_indication_supportive | 6 | 5.31 (2.38-11.84) | 1.78 | Ineligible |
| T-DM1 | Angioedema | all_indication_supportive | 6 | 0.56 (0.25-1.25) | 0.33 | Ineligible |
| T-DM1 | Arteriovenous malformation | all_indication_supportive | 6 | 29.55 (13.22-66.05) | 12.59 | Ineligible |
| T-DM1 | Ataxia | all_indication_supportive | 6 | 2.21 (0.99-4.91) | 0.99 | Ineligible |
| T-DM1 | Atrial fibrillation | all_indication_supportive | 6 | 0.26 (0.12-0.58) | 0.16 | Ineligible |
| T-DM1 | Blindness unilateral | all_indication_supportive | 6 | 1.87 (0.84-4.16) | 0.88 | Ineligible |
| T-DM1 | Blood test abnormal | all_indication_supportive | 6 | 1.58 (0.71-3.51) | 0.78 | Ineligible |
| T-DM1 | Breast operation | all_indication_supportive | 6 | 35.07 (15.68-78.45) | 14.64 | Ineligible |
| T-DM1 | Coma | all_indication_supportive | 6 | 0.57 (0.26-1.27) | 0.33 | Ineligible |
| T-DM1 | Deafness | all_indication_supportive | 6 | 0.92 (0.42-2.06) | 0.50 | Ineligible |
| T-DM1 | Deep vein thrombosis | all_indication_supportive | 6 | 0.44 (0.20-0.97) | 0.26 | Ineligible |
| T-DM1 | Dermatitis acneiform | all_indication_supportive | 6 | 4.16 (1.87-9.26) | 1.52 | Ineligible |
| T-DM1 | Dysphonia | all_indication_supportive | 6 | 0.41 (0.18-0.91) | 0.24 | Ineligible |
| T-DM1 | Full blood count abnormal | all_indication_supportive | 6 | 1.97 (0.88-4.38) | 0.92 | Ineligible |
| T-DM1 | Haemangioma | all_indication_supportive | 6 | 11.81 (5.30-26.34) | 3.74 | Ineligible |
| T-DM1 | Hallucination | all_indication_supportive | 6 | 0.34 (0.15-0.77) | 0.21 | Ineligible |
| T-DM1 | Hepatic neoplasm | all_indication_supportive | 6 | 7.82 (3.51-17.43) | 2.38 | Ineligible |
| T-DM1 | Hyperlipidaemia | all_indication_supportive | 6 | 2.83 (1.27-6.30) | 1.18 | Ineligible |
| T-DM1 | Hypertensive crisis | all_indication_supportive | 6 | 2.24 (1.00-4.98) | 1.00 | Ineligible |
| T-DM1 | Immune thrombocytopenic purpura | all_indication_supportive | 6 | 6.77 (3.04-15.09) | 2.12 | Ineligible |
| T-DM1 | Intestinal obstruction | all_indication_supportive | 6 | 0.64 (0.29-1.43) | 0.37 | Ineligible |
| T-DM1 | Left ventricular dysfunction | all_indication_supportive | 6 | 3.85 (1.73-8.57) | 1.44 | Ineligible |
| T-DM1 | Lethargy | all_indication_supportive | 6 | 0.46 (0.21-1.02) | 0.27 | Ineligible |
| T-DM1 | Lipase increased | all_indication_supportive | 6 | 3.65 (1.64-8.14) | 1.40 | Ineligible |
| T-DM1 | Lower gastrointestinal haemorrhage | all_indication_supportive | 6 | 3.07 (1.38-6.83) | 1.25 | Ineligible |
| T-DM1 | Mass | all_indication_supportive | 6 | 1.68 (0.75-3.74) | 0.82 | Ineligible |
| T-DM1 | Mobility decreased | all_indication_supportive | 6 | 0.32 (0.14-0.71) | 0.19 | Ineligible |
| T-DM1 | Myocardial infarction | all_indication_supportive | 6 | 0.18 (0.08-0.41) | 0.11 | Ineligible |
| T-DM1 | Necrosis | all_indication_supportive | 6 | 5.23 (2.35-11.65) | 1.76 | Ineligible |
| T-DM1 | Neurological symptom | all_indication_supportive | 6 | 4.65 (2.09-10.36) | 1.63 | Ineligible |
| T-DM1 | Neutrophil count increased | all_indication_supportive | 6 | 2.38 (1.07-5.30) | 1.05 | Ineligible |
| T-DM1 | Onychomadesis | all_indication_supportive | 6 | 5.72 (2.57-12.74) | 1.87 | Ineligible |
| T-DM1 | Periorbital oedema | all_indication_supportive | 6 | 5.16 (2.31-11.49) | 1.74 | Ineligible |
| T-DM1 | Pollakiuria | all_indication_supportive | 6 | 0.60 (0.27-1.33) | 0.34 | Ineligible |
| T-DM1 | Productive cough | all_indication_supportive | 6 | 0.48 (0.22-1.07) | 0.28 | Ineligible |
| T-DM1 | Pulmonary alveolar haemorrhage | all_indication_supportive | 6 | 4.33 (1.94-9.66) | 1.56 | Ineligible |
| T-DM1 | Respiratory disorder | all_indication_supportive | 6 | 0.84 (0.38-1.88) | 0.47 | Ineligible |
| T-DM1 | Skin discolouration | all_indication_supportive | 6 | 0.52 (0.23-1.16) | 0.30 | Ineligible |
| T-DM1 | Skin fissures | all_indication_supportive | 6 | 1.19 (0.53-2.64) | 0.62 | Ineligible |
| T-DM1 | Skin infection | all_indication_supportive | 6 | 2.06 (0.92-4.59) | 0.95 | Ineligible |
| T-DM1 | Skin lesion | all_indication_supportive | 6 | 0.90 (0.40-2.00) | 0.49 | Ineligible |
| T-DM1 | Therapeutic response decreased | all_indication_supportive | 6 | 0.41 (0.18-0.90) | 0.24 | Ineligible |
| T-DM1 | Tooth disorder | all_indication_supportive | 6 | 1.07 (0.48-2.37) | 0.57 | Ineligible |
| T-DM1 | Type 2 diabetes mellitus | all_indication_supportive | 6 | 0.72 (0.32-1.60) | 0.40 | Ineligible |
| T-DM1 | Urosepsis | all_indication_supportive | 6 | 2.69 (1.21-5.99) | 1.14 | Ineligible |
| T-DM1 | Acute coronary syndrome | all_indication_supportive | 5 | 2.71 (1.13-6.51) | 1.06 | Ineligible |
| T-DM1 | Altered state of consciousness | all_indication_supportive | 5 | 0.94 (0.39-2.25) | 0.49 | Ineligible |
| T-DM1 | Arthropathy | all_indication_supportive | 5 | 0.34 (0.14-0.82) | 0.20 | Ineligible |
| T-DM1 | Axillary pain | all_indication_supportive | 5 | 13.58 (5.64-32.70) | 3.68 | Ineligible |
| T-DM1 | Bacteraemia | all_indication_supportive | 5 | 1.73 (0.72-4.17) | 0.78 | Ineligible |
| T-DM1 | Blood bilirubin decreased | all_indication_supportive | 5 | 30.74 (12.74-74.20) | 11.67 | Ineligible |
| T-DM1 | Blood calcium decreased | all_indication_supportive | 5 | 1.90 (0.79-4.57) | 0.84 | Ineligible |
| T-DM1 | Blood chloride decreased | all_indication_supportive | 5 | 12.73 (5.29-30.66) | 3.38 | Ineligible |
| T-DM1 | Blood creatine phosphokinase increased | all_indication_supportive | 5 | 0.93 (0.39-2.24) | 0.49 | Ineligible |
| T-DM1 | Blood urea increased | all_indication_supportive | 5 | 1.75 (0.73-4.20) | 0.79 | Ineligible |
| T-DM1 | Body temperature decreased | all_indication_supportive | 5 | 2.03 (0.84-4.88) | 0.87 | Ineligible |
| T-DM1 | Brain fog | all_indication_supportive | 5 | 1.33 (0.55-3.19) | 0.64 | Ineligible |
| T-DM1 | Brain injury | all_indication_supportive | 5 | 1.87 (0.78-4.50) | 0.83 | Ineligible |
| T-DM1 | Brain operation | all_indication_supportive | 5 | 8.45 (3.51-20.33) | 2.21 | Ineligible |
| T-DM1 | Catheter site pain | all_indication_supportive | 5 | 7.49 (3.11-18.01) | 2.00 | Ineligible |
| T-DM1 | Cholangitis | all_indication_supportive | 5 | 3.40 (1.42-8.18) | 1.22 | Ineligible |
| T-DM1 | Coagulopathy | all_indication_supportive | 5 | 1.36 (0.56-3.26) | 0.65 | Ineligible |
| T-DM1 | Convulsion | all_indication_supportive | 5 | 1.17 (0.49-2.82) | 0.58 | Ineligible |
| T-DM1 | Diplopia | all_indication_supportive | 5 | 0.85 (0.35-2.05) | 0.45 | Ineligible |
| T-DM1 | Drug effect decreased | all_indication_supportive | 5 | 0.41 (0.17-0.99) | 0.24 | Ineligible |
| T-DM1 | Dysarthria | all_indication_supportive | 5 | 0.60 (0.25-1.45) | 0.33 | Ineligible |
| T-DM1 | Eczema | all_indication_supportive | 5 | 0.39 (0.16-0.93) | 0.22 | Ineligible |
| T-DM1 | Encephalitis | all_indication_supportive | 5 | 3.06 (1.27-7.35) | 1.14 | Ineligible |
| T-DM1 | Enteritis | all_indication_supportive | 5 | 3.02 (1.25-7.25) | 1.13 | Ineligible |
| T-DM1 | Eosinophil count increased | all_indication_supportive | 5 | 2.49 (1.04-6.00) | 1.00 | Ineligible |
| T-DM1 | Erysipelas | all_indication_supportive | 5 | 4.02 (1.67-9.66) | 1.34 | Ineligible |
| T-DM1 | Erythema multiforme | all_indication_supportive | 5 | 2.52 (1.05-6.05) | 1.01 | Ineligible |
| T-DM1 | Eye swelling | all_indication_supportive | 5 | 0.55 (0.23-1.32) | 0.31 | Ineligible |
| T-DM1 | Feeling hot | all_indication_supportive | 5 | 0.36 (0.15-0.86) | 0.21 | Ineligible |
| T-DM1 | Full blood count decreased | all_indication_supportive | 5 | 0.87 (0.36-2.08) | 0.46 | Ineligible |
| T-DM1 | Fungal infection | all_indication_supportive | 5 | 0.59 (0.25-1.42) | 0.33 | Ineligible |
| T-DM1 | Gastric disorder | all_indication_supportive | 5 | 0.56 (0.23-1.34) | 0.31 | Ineligible |
| T-DM1 | Gastric varices | all_indication_supportive | 5 | 45.98 (19.01-111.21) | 16.42 | Ineligible |
| T-DM1 | Haemolytic anaemia | all_indication_supportive | 5 | 2.62 (1.09-6.31) | 1.04 | Ineligible |
| T-DM1 | Heart rate decreased | all_indication_supportive | 5 | 0.57 (0.24-1.38) | 0.32 | Ineligible |
| T-DM1 | Heavy menstrual bleeding | all_indication_supportive | 5 | 2.35 (0.98-5.65) | 0.96 | Ineligible |
| T-DM1 | Hepatic cyst | all_indication_supportive | 5 | 6.60 (2.74-15.87) | 1.83 | Ineligible |
| T-DM1 | Hepatic lesion | all_indication_supportive | 5 | 4.79 (1.99-11.51) | 1.49 | Ineligible |
| T-DM1 | Hydrocephalus | all_indication_supportive | 5 | 4.11 (1.71-9.88) | 1.36 | Ineligible |
| T-DM1 | Hyperuricaemia | all_indication_supportive | 5 | 5.22 (2.17-12.56) | 1.57 | Ineligible |
| T-DM1 | Hypoacusis | all_indication_supportive | 5 | 0.41 (0.17-1.00) | 0.24 | Ineligible |
| T-DM1 | Hypophagia | all_indication_supportive | 5 | 0.79 (0.33-1.89) | 0.42 | Ineligible |
| T-DM1 | Incorrect dose administered | all_indication_supportive | 5 | 0.08 (0.03-0.20) | 0.05 | Ineligible |
| T-DM1 | Infusion site extravasation | all_indication_supportive | 5 | 2.97 (1.23-7.13) | 1.12 | Ineligible |
| T-DM1 | Injection site extravasation | all_indication_supportive | 5 | 1.60 (0.67-3.85) | 0.74 | Ineligible |
| T-DM1 | Injury | all_indication_supportive | 5 | 0.32 (0.13-0.77) | 0.19 | Ineligible |
| T-DM1 | Joint stiffness | all_indication_supportive | 5 | 0.75 (0.31-1.81) | 0.41 | Ineligible |
| T-DM1 | Kidney infection | all_indication_supportive | 5 | 0.99 (0.41-2.38) | 0.51 | Ineligible |
| T-DM1 | Lung neoplasm malignant | all_indication_supportive | 5 | 0.46 (0.19-1.09) | 0.26 | Ineligible |
| T-DM1 | Lymphoma | all_indication_supportive | 5 | 1.33 (0.55-3.21) | 0.64 | Ineligible |
| T-DM1 | Mental disorder | all_indication_supportive | 5 | 0.50 (0.21-1.20) | 0.28 | Ineligible |
| T-DM1 | Muscle twitching | all_indication_supportive | 5 | 0.95 (0.40-2.28) | 0.49 | Ineligible |
| T-DM1 | Myelodysplastic syndrome | all_indication_supportive | 5 | 1.53 (0.64-3.68) | 0.72 | Ineligible |
| T-DM1 | Nail disorder | all_indication_supportive | 5 | 2.28 (0.95-5.48) | 0.95 | Ineligible |
| T-DM1 | Nephrolithiasis | all_indication_supportive | 5 | 0.44 (0.18-1.05) | 0.25 | Ineligible |
| T-DM1 | Optic neuritis | all_indication_supportive | 5 | 2.50 (1.04-6.00) | 1.00 | Ineligible |
| T-DM1 | Oral candidiasis | all_indication_supportive | 5 | 1.62 (0.67-3.90) | 0.75 | Ineligible |
| T-DM1 | Osteonecrosis | all_indication_supportive | 5 | 0.79 (0.33-1.89) | 0.42 | Ineligible |
| T-DM1 | Ovarian cyst | all_indication_supportive | 5 | 2.22 (0.92-5.33) | 0.93 | Ineligible |
| T-DM1 | Paronychia | all_indication_supportive | 5 | 4.58 (1.90-11.01) | 1.45 | Ineligible |
| T-DM1 | Peripheral coldness | all_indication_supportive | 5 | 1.58 (0.66-3.79) | 0.73 | Ineligible |
| T-DM1 | Pneumonia bacterial | all_indication_supportive | 5 | 2.14 (0.89-5.15) | 0.91 | Ineligible |
| T-DM1 | Posterior reversible encephalopathy syndrome | all_indication_supportive | 5 | 1.91 (0.79-4.59) | 0.84 | Ineligible |
| T-DM1 | Proteinuria | all_indication_supportive | 5 | 1.09 (0.45-2.63) | 0.55 | Ineligible |
| T-DM1 | Rash maculo-papular | all_indication_supportive | 5 | 0.97 (0.40-2.32) | 0.50 | Ineligible |
| T-DM1 | Retching | all_indication_supportive | 5 | 0.98 (0.41-2.37) | 0.51 | Ineligible |
| T-DM1 | Rib fracture | all_indication_supportive | 5 | 0.98 (0.41-2.36) | 0.51 | Ineligible |
| T-DM1 | SARS-CoV-2 test positive | all_indication_supportive | 5 | 1.22 (0.51-2.94) | 0.60 | Ineligible |
| T-DM1 | Skin ulcer | all_indication_supportive | 5 | 0.79 (0.33-1.90) | 0.42 | Ineligible |
| T-DM1 | Staphylococcal infection | all_indication_supportive | 5 | 0.69 (0.29-1.67) | 0.38 | Ineligible |
| T-DM1 | Subarachnoid haemorrhage | all_indication_supportive | 5 | 2.05 (0.85-4.92) | 0.88 | Ineligible |
| T-DM1 | Surgery | all_indication_supportive | 5 | 0.34 (0.14-0.82) | 0.20 | Ineligible |
| T-DM1 | Swelling face | all_indication_supportive | 5 | 0.33 (0.14-0.79) | 0.19 | Ineligible |
| T-DM1 | Systemic lupus erythematosus | all_indication_supportive | 5 | 0.53 (0.22-1.28) | 0.30 | Ineligible |
| T-DM1 | Throat irritation | all_indication_supportive | 5 | 0.45 (0.19-1.09) | 0.26 | Ineligible |
| T-DM1 | Toothache | all_indication_supportive | 5 | 1.15 (0.48-2.75) | 0.57 | Ineligible |
| T-DM1 | Treatment failure | all_indication_supportive | 5 | 0.21 (0.09-0.50) | 0.12 | Ineligible |
| T-DM1 | Vascular rupture | all_indication_supportive | 5 | 13.65 (5.67-32.86) | 3.71 | Ineligible |
| T-DM1 | Abasia | all_indication_supportive | 4 | 0.93 (0.35-2.47) | 0.46 | Ineligible |
| T-DM1 | Acne | all_indication_supportive | 4 | 0.18 (0.07-0.49) | 0.11 | Ineligible |
| T-DM1 | Acute pulmonary oedema | all_indication_supportive | 4 | 3.46 (1.30-9.22) | 1.09 | Ineligible |
| T-DM1 | Adrenal insufficiency | all_indication_supportive | 4 | 1.28 (0.48-3.42) | 0.58 | Ineligible |
| T-DM1 | Ankle fracture | all_indication_supportive | 4 | 1.06 (0.40-2.82) | 0.51 | Ineligible |
| T-DM1 | Aortic valve incompetence | all_indication_supportive | 4 | 5.29 (1.98-14.10) | 1.38 | Ineligible |
| T-DM1 | Arthritis | all_indication_supportive | 4 | 0.20 (0.07-0.53) | 0.11 | Ineligible |
| T-DM1 | Asthenopia | all_indication_supportive | 4 | 2.90 (1.09-7.74) | 0.99 | Ineligible |
| T-DM1 | Astigmatism | all_indication_supportive | 4 | 10.70 (4.01-28.56) | 2.21 | Ineligible |
| T-DM1 | Bile duct obstruction | all_indication_supportive | 4 | 10.37 (3.89-27.70) | 2.15 | Ineligible |
| T-DM1 | Blood cholesterol increased | all_indication_supportive | 4 | 0.40 (0.15-1.06) | 0.22 | Ineligible |
| T-DM1 | Blood urine present | all_indication_supportive | 4 | 0.86 (0.32-2.29) | 0.43 | Ineligible |
| T-DM1 | Bone cancer | all_indication_supportive | 4 | 3.05 (1.15-8.14) | 1.02 | Ineligible |
| T-DM1 | Bone lesion | all_indication_supportive | 4 | 4.66 (1.75-12.44) | 1.29 | Ineligible |
| T-DM1 | Candida infection | all_indication_supportive | 4 | 0.81 (0.30-2.17) | 0.41 | Ineligible |
| T-DM1 | Carbohydrate antigen 125 increased | all_indication_supportive | 4 | 4.01 (1.50-10.68) | 1.18 | Ineligible |
| T-DM1 | Cardiac tamponade | all_indication_supportive | 4 | 3.59 (1.35-9.57) | 1.12 | Ineligible |
| T-DM1 | Catheter site erythema | all_indication_supportive | 4 | 7.22 (2.71-19.27) | 1.65 | Ineligible |
| T-DM1 | Central nervous system necrosis | all_indication_supportive | 4 | 63.18 (23.47-170.03) | 17.61 | Ineligible |
| T-DM1 | Cerebral infarction | all_indication_supportive | 4 | 0.71 (0.27-1.90) | 0.37 | Ineligible |
| T-DM1 | Cholelithiasis | all_indication_supportive | 4 | 0.61 (0.23-1.63) | 0.32 | Ineligible |
| T-DM1 | Chromaturia | all_indication_supportive | 4 | 0.76 (0.28-2.02) | 0.39 | Ineligible |
| T-DM1 | Chronic gastritis | all_indication_supportive | 4 | 6.13 (2.30-16.35) | 1.50 | Ineligible |
| T-DM1 | Clostridium difficile infection | all_indication_supportive | 4 | 0.63 (0.24-1.68) | 0.33 | Ineligible |
| T-DM1 | Corneal deposits | all_indication_supportive | 4 | 27.86 (10.41-74.59) | 8.46 | Ineligible |
| T-DM1 | Coronavirus infection | all_indication_supportive | 4 | 1.98 (0.74-5.29) | 0.79 | Ineligible |
| T-DM1 | Cystitis haemorrhagic | all_indication_supportive | 4 | 4.07 (1.53-10.87) | 1.20 | Ineligible |
| T-DM1 | Demyelination | all_indication_supportive | 4 | 4.53 (1.70-12.08) | 1.27 | Ineligible |
| T-DM1 | Depressed level of consciousness | all_indication_supportive | 4 | 0.48 (0.18-1.28) | 0.26 | Ineligible |
| T-DM1 | Dermatitis | all_indication_supportive | 4 | 0.76 (0.28-2.02) | 0.39 | Ineligible |
| T-DM1 | Disturbance in attention | all_indication_supportive | 4 | 0.31 (0.12-0.84) | 0.18 | Ineligible |
| T-DM1 | Drug administration error | all_indication_supportive | 4 | 0.57 (0.21-1.52) | 0.30 | Ineligible |
| T-DM1 | Duodenal ulcer | all_indication_supportive | 4 | 2.65 (0.99-7.06) | 0.94 | Ineligible |
| T-DM1 | Dysuria | all_indication_supportive | 4 | 0.46 (0.17-1.24) | 0.25 | Ineligible |
| T-DM1 | Electrocardiogram QT prolonged | all_indication_supportive | 4 | 0.45 (0.17-1.20) | 0.25 | Ineligible |
| T-DM1 | Eye haemorrhage | all_indication_supportive | 4 | 1.28 (0.48-3.42) | 0.58 | Ineligible |
| T-DM1 | Face oedema | all_indication_supportive | 4 | 1.11 (0.41-2.95) | 0.52 | Ineligible |
| T-DM1 | Faeces discoloured | all_indication_supportive | 4 | 0.80 (0.30-2.12) | 0.40 | Ineligible |
| T-DM1 | Flatulence | all_indication_supportive | 4 | 0.30 (0.11-0.80) | 0.17 | Ineligible |
| T-DM1 | Gait inability | all_indication_supportive | 4 | 0.41 (0.15-1.09) | 0.23 | Ineligible |
| T-DM1 | Generalised oedema | all_indication_supportive | 4 | 1.50 (0.56-3.99) | 0.65 | Ineligible |
| T-DM1 | Guillain-Barre syndrome | all_indication_supportive | 4 | 3.60 (1.35-9.60) | 1.12 | Ineligible |
| T-DM1 | HER-2 positive breast cancer | all_indication_supportive | 4 | 94.53 (34.96-255.63) | 22.54 | Ineligible |
| T-DM1 | Haematoma | all_indication_supportive | 4 | 0.66 (0.25-1.75) | 0.34 | Ineligible |
| T-DM1 | Haematotoxicity | all_indication_supportive | 4 | 1.65 (0.62-4.41) | 0.70 | Ineligible |
| T-DM1 | Henoch-Schonlein purpura | all_indication_supportive | 4 | 8.70 (3.26-23.23) | 1.87 | Ineligible |
| T-DM1 | Hip fracture | all_indication_supportive | 4 | 0.51 (0.19-1.37) | 0.28 | Ineligible |
| T-DM1 | Hyperammonaemia | all_indication_supportive | 4 | 3.40 (1.27-9.07) | 1.08 | Ineligible |
| T-DM1 | Hyperkalaemia | all_indication_supportive | 4 | 0.50 (0.19-1.34) | 0.27 | Ineligible |
| T-DM1 | Hypophosphataemia | all_indication_supportive | 4 | 2.20 (0.82-5.86) | 0.84 | Ineligible |
| T-DM1 | Hypoproteinaemia | all_indication_supportive | 4 | 10.52 (3.94-28.09) | 2.18 | Ineligible |
| T-DM1 | Intercepted product preparation error | all_indication_supportive | 4 | 1.10 (0.41-2.93) | 0.52 | Ineligible |
| T-DM1 | Intracranial mass | all_indication_supportive | 4 | 14.24 (5.33-38.05) | 2.95 | Ineligible |
| T-DM1 | Iron deficiency anaemia | all_indication_supportive | 4 | 1.82 (0.68-4.85) | 0.74 | Ineligible |
| T-DM1 | Ischaemic stroke | all_indication_supportive | 4 | 0.90 (0.34-2.40) | 0.45 | Ineligible |
| T-DM1 | Laryngeal oedema | all_indication_supportive | 4 | 3.01 (1.13-8.02) | 1.01 | Ineligible |
| T-DM1 | Laryngitis | all_indication_supportive | 4 | 1.48 (0.55-3.94) | 0.65 | Ineligible |
| T-DM1 | Leukocytosis | all_indication_supportive | 4 | 1.01 (0.38-2.68) | 0.49 | Ineligible |
| T-DM1 | Leukoencephalopathy | all_indication_supportive | 4 | 5.01 (1.88-13.36) | 1.34 | Ineligible |
| T-DM1 | Lymphadenopathy mediastinal | all_indication_supportive | 4 | 8.71 (3.26-23.25) | 1.88 | Ineligible |
| T-DM1 | Lymphangitis | all_indication_supportive | 4 | 22.16 (8.28-59.25) | 5.81 | Ineligible |
| T-DM1 | Malignant pleural effusion | all_indication_supportive | 4 | 10.23 (3.83-27.30) | 2.13 | Ineligible |
| T-DM1 | Mastitis | all_indication_supportive | 4 | 8.10 (3.03-21.61) | 1.78 | Ineligible |
| T-DM1 | Mean cell haemoglobin increased | all_indication_supportive | 4 | 9.06 (3.39-24.17) | 1.93 | Ineligible |
| T-DM1 | Menorrhagia | all_indication_supportive | 4 | 0.91 (0.34-2.42) | 0.45 | Ineligible |
| T-DM1 | Mental status changes | all_indication_supportive | 4 | 0.69 (0.26-1.85) | 0.36 | Ineligible |
| T-DM1 | Motor dysfunction | all_indication_supportive | 4 | 1.68 (0.63-4.48) | 0.71 | Ineligible |
| T-DM1 | Nasal congestion | all_indication_supportive | 4 | 0.26 (0.10-0.70) | 0.15 | Ineligible |
| T-DM1 | Nasal dryness | all_indication_supportive | 4 | 2.51 (0.94-6.69) | 0.91 | Ineligible |
| T-DM1 | Night sweats | all_indication_supportive | 4 | 0.50 (0.19-1.33) | 0.27 | Ineligible |
| T-DM1 | Nodule | all_indication_supportive | 4 | 1.17 (0.44-3.12) | 0.55 | Ineligible |
| T-DM1 | Oral herpes | all_indication_supportive | 4 | 0.78 (0.29-2.07) | 0.40 | Ineligible |
| T-DM1 | Organising pneumonia | all_indication_supportive | 4 | 3.12 (1.17-8.31) | 1.03 | Ineligible |
| T-DM1 | Overdose | all_indication_supportive | 4 | 0.07 (0.03-0.19) | 0.04 | Ineligible |
| T-DM1 | Pain of skin | all_indication_supportive | 4 | 0.53 (0.20-1.41) | 0.28 | Ineligible |
| T-DM1 | Paravenous drug administration | all_indication_supportive | 4 | 327.23 (116.89-916.02) | 36.96 | Ineligible |
| T-DM1 | Peripheral sensorimotor neuropathy | all_indication_supportive | 4 | 13.87 (5.19-37.05) | 2.86 | Ineligible |
| T-DM1 | Pharyngitis | all_indication_supportive | 4 | 1.32 (0.49-3.51) | 0.60 | Ineligible |
| T-DM1 | Photophobia | all_indication_supportive | 4 | 0.87 (0.33-2.33) | 0.44 | Ineligible |
| T-DM1 | Pneumonia viral | all_indication_supportive | 4 | 4.82 (1.81-12.86) | 1.31 | Ineligible |
| T-DM1 | Post procedural haemorrhage | all_indication_supportive | 4 | 1.58 (0.59-4.21) | 0.68 | Ineligible |
| T-DM1 | Product temperature excursion issue | all_indication_supportive | 4 | 3.00 (1.13-8.01) | 1.01 | Ineligible |
| T-DM1 | Prothrombin time prolonged | all_indication_supportive | 4 | 4.71 (1.77-12.57) | 1.29 | Ineligible |
| T-DM1 | Psychotic disorder | all_indication_supportive | 4 | 0.64 (0.24-1.70) | 0.34 | Ineligible |
| T-DM1 | Respiratory arrest | all_indication_supportive | 4 | 0.81 (0.30-2.15) | 0.41 | Ineligible |
| T-DM1 | Respiratory tract infection viral | all_indication_supportive | 4 | 6.51 (2.44-17.37) | 1.55 | Ineligible |
| T-DM1 | Sinusitis | all_indication_supportive | 4 | 0.15 (0.06-0.41) | 0.09 | Ineligible |
| T-DM1 | Skin necrosis | all_indication_supportive | 4 | 3.45 (1.29-9.19) | 1.09 | Ineligible |
| T-DM1 | Skin reaction | all_indication_supportive | 4 | 1.12 (0.42-2.98) | 0.53 | Ineligible |
| T-DM1 | Small intestinal obstruction | all_indication_supportive | 4 | 1.38 (0.52-3.68) | 0.62 | Ineligible |
| T-DM1 | Spinal pain | all_indication_supportive | 4 | 1.16 (0.44-3.10) | 0.54 | Ineligible |
| T-DM1 | Subcapsular hepatic haematoma | all_indication_supportive | 4 | 319.05 (114.11-892.05) | 36.72 | Ineligible |
| T-DM1 | Subdural haematoma | all_indication_supportive | 4 | 1.14 (0.43-3.03) | 0.53 | Ineligible |
| T-DM1 | Systemic inflammatory response syndrome | all_indication_supportive | 4 | 4.42 (1.66-11.78) | 1.25 | Ineligible |
| T-DM1 | Therapy interrupted | all_indication_supportive | 4 | 0.21 (0.08-0.55) | 0.12 | Ineligible |
| T-DM1 | Therapy non-responder | all_indication_supportive | 4 | 0.32 (0.12-0.86) | 0.18 | Ineligible |
| T-DM1 | Tinnitus | all_indication_supportive | 4 | 0.37 (0.14-0.97) | 0.20 | Ineligible |
| T-DM1 | Tooth fracture | all_indication_supportive | 4 | 1.46 (0.55-3.88) | 0.64 | Ineligible |
| T-DM1 | Ulcerative keratitis | all_indication_supportive | 4 | 6.20 (2.32-16.53) | 1.50 | Ineligible |
| T-DM1 | Upper-airway cough syndrome | all_indication_supportive | 4 | 1.77 (0.66-4.72) | 0.73 | Ineligible |
| T-DM1 | Urinary tract disorder | all_indication_supportive | 4 | 3.60 (1.35-9.61) | 1.12 | Ineligible |
| T-DM1 | Vasculitis | all_indication_supportive | 4 | 1.57 (0.59-4.18) | 0.67 | Ineligible |
| T-DM1 | Viral upper respiratory tract infection | all_indication_supportive | 4 | 2.95 (1.11-7.86) | 1.00 | Ineligible |
| T-DM1 | Weight fluctuation | all_indication_supportive | 4 | 1.41 (0.53-3.77) | 0.63 | Ineligible |
| T-DM1 | Wound | all_indication_supportive | 4 | 0.52 (0.20-1.39) | 0.28 | Ineligible |
| T-DM1 | Wound dehiscence | all_indication_supportive | 4 | 5.62 (2.11-14.98) | 1.42 | Ineligible |
| T-DM1 | Abortion spontaneous | all_indication_supportive | 3 | 0.33 (0.11-1.03) | 0.18 | Ineligible |
| T-DM1 | Acute respiratory failure | all_indication_supportive | 3 | 0.62 (0.20-1.92) | 0.31 | Ineligible |
| T-DM1 | Angina pectoris | all_indication_supportive | 3 | 0.50 (0.16-1.55) | 0.26 | Ineligible |
| T-DM1 | Aortic arteriosclerosis | all_indication_supportive | 3 | 5.23 (1.68-16.22) | 1.13 | Ineligible |
| T-DM1 | Aphthous ulcer | all_indication_supportive | 3 | 1.32 (0.43-4.09) | 0.54 | Ineligible |
| T-DM1 | Arthritis bacterial | all_indication_supportive | 3 | 3.10 (1.00-9.61) | 0.89 | Ineligible |
| T-DM1 | Arthritis infective | all_indication_supportive | 3 | 2.52 (0.81-7.83) | 0.80 | Ineligible |
| T-DM1 | Bacterial infection | all_indication_supportive | 3 | 0.68 (0.22-2.10) | 0.33 | Ineligible |
| T-DM1 | Bacterial sepsis | all_indication_supportive | 3 | 4.12 (1.33-12.78) | 1.01 | Ineligible |
| T-DM1 | Blood bilirubin unconjugated increased | all_indication_supportive | 3 | 22.46 (7.21-69.95) | 3.28 | Ineligible |
| T-DM1 | Blood calcium increased | all_indication_supportive | 3 | 1.43 (0.46-4.44) | 0.57 | Ineligible |
| T-DM1 | Blood disorder | all_indication_supportive | 3 | 1.58 (0.51-4.90) | 0.61 | Ineligible |
| T-DM1 | Blood phosphorus increased | all_indication_supportive | 3 | 4.76 (1.53-14.78) | 1.08 | Ineligible |
| T-DM1 | Blood thyroid stimulating hormone increased | all_indication_supportive | 3 | 1.29 (0.41-3.99) | 0.53 | Ineligible |
| T-DM1 | Blood urea decreased | all_indication_supportive | 3 | 13.00 (4.18-40.42) | 1.92 | Ineligible |
| T-DM1 | Blood uric acid decreased | all_indication_supportive | 3 | 27.34 (8.77-85.21) | 4.34 | Ineligible |
| T-DM1 | Bone neoplasm | all_indication_supportive | 3 | 11.14 (3.58-34.62) | 1.72 | Ineligible |
| T-DM1 | Breast cellulitis | all_indication_supportive | 3 | 43.70 (13.98-136.58) | 9.35 | Ineligible |
| T-DM1 | Breast haemorrhage | all_indication_supportive | 3 | 35.98 (11.53-112.29) | 7.11 | Ineligible |
| T-DM1 | Breast reconstruction | all_indication_supportive | 3 | 35.98 (11.53-112.29) | 7.11 | Ineligible |
| T-DM1 | Bronchospasm | all_indication_supportive | 3 | 0.96 (0.31-2.98) | 0.43 | Ineligible |
| T-DM1 | Cancer pain | all_indication_supportive | 3 | 4.30 (1.38-13.34) | 1.03 | Ineligible |
| T-DM1 | Catheter site extravasation | all_indication_supportive | 3 | 24.99 (8.02-77.84) | 3.78 | Ineligible |
| T-DM1 | Cell death | all_indication_supportive | 3 | 6.05 (1.95-18.79) | 1.21 | Ineligible |
| T-DM1 | Chronic hepatitis | all_indication_supportive | 3 | 18.87 (6.06-58.74) | 2.69 | Ineligible |
| T-DM1 | Chronic myeloid leukaemia | all_indication_supportive | 3 | 2.64 (0.85-8.19) | 0.82 | Ineligible |
| T-DM1 | Circulatory collapse | all_indication_supportive | 3 | 0.84 (0.27-2.61) | 0.39 | Ineligible |
| T-DM1 | Circumstance or information capable of leading to medication error | all_indication_supportive | 3 | 0.32 (0.10-0.98) | 0.17 | Ineligible |
| T-DM1 | Colitis ischaemic | all_indication_supportive | 3 | 2.09 (0.67-6.50) | 0.72 | Ineligible |
| T-DM1 | Conjunctival hyperaemia | all_indication_supportive | 3 | 3.57 (1.15-11.07) | 0.95 | Ineligible |
| T-DM1 | Cutaneous lupus erythematosus | all_indication_supportive | 3 | 8.18 (2.63-25.40) | 1.42 | Ineligible |
| T-DM1 | Dacryostenosis acquired | all_indication_supportive | 3 | 8.84 (2.84-27.45) | 1.48 | Ineligible |
| T-DM1 | Decreased immune responsiveness | all_indication_supportive | 3 | 1.08 (0.35-3.34) | 0.47 | Ineligible |
| T-DM1 | Delirium | all_indication_supportive | 3 | 0.37 (0.12-1.14) | 0.20 | Ineligible |
| T-DM1 | Dementia | all_indication_supportive | 3 | 0.43 (0.14-1.32) | 0.22 | Ineligible |
| T-DM1 | Dental caries | all_indication_supportive | 3 | 1.20 (0.39-3.73) | 0.51 | Ineligible |
| T-DM1 | Depressed mood | all_indication_supportive | 3 | 0.22 (0.07-0.69) | 0.12 | Ineligible |
| T-DM1 | Device related sepsis | all_indication_supportive | 3 | 5.53 (1.78-17.17) | 1.16 | Ineligible |
| T-DM1 | Diverticulitis | all_indication_supportive | 3 | 0.43 (0.14-1.34) | 0.23 | Ineligible |
| T-DM1 | Duodenitis | all_indication_supportive | 3 | 4.61 (1.48-14.31) | 1.07 | Ineligible |
| T-DM1 | Dyschezia | all_indication_supportive | 3 | 3.47 (1.12-10.78) | 0.94 | Ineligible |
| T-DM1 | Electrolyte imbalance | all_indication_supportive | 3 | 1.11 (0.36-3.43) | 0.48 | Ineligible |
| T-DM1 | Emotional distress | all_indication_supportive | 3 | 0.09 (0.03-0.28) | 0.05 | Ineligible |
| T-DM1 | Endocarditis | all_indication_supportive | 3 | 2.50 (0.81-7.76) | 0.79 | Ineligible |
| T-DM1 | Exercise tolerance decreased | all_indication_supportive | 3 | 2.09 (0.67-6.48) | 0.72 | Ineligible |
| T-DM1 | Extrasystoles | all_indication_supportive | 3 | 2.07 (0.67-6.43) | 0.72 | Ineligible |
| T-DM1 | Fear | all_indication_supportive | 3 | 0.59 (0.19-1.84) | 0.30 | Ineligible |
| T-DM1 | Febrile infection | all_indication_supportive | 3 | 10.36 (3.33-32.18) | 1.63 | Ineligible |
| T-DM1 | Feeling cold | all_indication_supportive | 3 | 0.45 (0.15-1.40) | 0.24 | Ineligible |
| T-DM1 | Fibrosis | all_indication_supportive | 3 | 4.75 (1.53-14.74) | 1.08 | Ineligible |
| T-DM1 | Flank pain | all_indication_supportive | 3 | 1.37 (0.44-4.25) | 0.56 | Ineligible |
| T-DM1 | Focal segmental glomerulosclerosis | all_indication_supportive | 3 | 7.50 (2.42-23.31) | 1.35 | Ineligible |
| T-DM1 | Foetal exposure during pregnancy | all_indication_supportive | 3 | 0.13 (0.04-0.40) | 0.07 | Ineligible |
| T-DM1 | Folliculitis | all_indication_supportive | 3 | 1.08 (0.35-3.35) | 0.47 | Ineligible |
| T-DM1 | Food poisoning | all_indication_supportive | 3 | 2.21 (0.71-6.86) | 0.74 | Ineligible |
| T-DM1 | Gastric antral vascular ectasia | all_indication_supportive | 3 | 24.54 (7.88-76.44) | 3.69 | Ineligible |
| T-DM1 | Gastric ulcer haemorrhage | all_indication_supportive | 3 | 2.53 (0.82-7.85) | 0.80 | Ineligible |
| T-DM1 | Gastroenteritis viral | all_indication_supportive | 3 | 0.64 (0.21-2.00) | 0.32 | Ineligible |
| T-DM1 | Gastrointestinal ulcer | all_indication_supportive | 3 | 6.21 (2.00-19.27) | 1.23 | Ineligible |
| T-DM1 | Haematochezia | all_indication_supportive | 3 | 0.21 (0.07-0.66) | 0.12 | Ineligible |
| T-DM1 | Haemoglobin increased | all_indication_supportive | 3 | 2.68 (0.86-8.30) | 0.82 | Ineligible |
| T-DM1 | Haemorrhagic stroke | all_indication_supportive | 3 | 1.54 (0.50-4.78) | 0.60 | Ineligible |
| T-DM1 | Hair growth abnormal | all_indication_supportive | 3 | 1.70 (0.55-5.26) | 0.64 | Ineligible |
| T-DM1 | Head discomfort | all_indication_supportive | 3 | 0.62 (0.20-1.91) | 0.31 | Ineligible |
| T-DM1 | Hepatic congestion | all_indication_supportive | 3 | 24.47 (7.86-76.24) | 3.67 | Ineligible |
| T-DM1 | Hepatocellular carcinoma | all_indication_supportive | 3 | 1.32 (0.43-4.11) | 0.54 | Ineligible |
| T-DM1 | Hyperchlorhydria | all_indication_supportive | 3 | 1.87 (0.60-5.81) | 0.68 | Ineligible |
| T-DM1 | Hypermetropia | all_indication_supportive | 3 | 13.71 (4.41-42.63) | 2.01 | Ineligible |
| T-DM1 | Hypersomnia | all_indication_supportive | 3 | 0.43 (0.14-1.33) | 0.23 | Ineligible |
| T-DM1 | Hypoaesthesia oral | all_indication_supportive | 3 | 0.87 (0.28-2.71) | 0.40 | Ineligible |
| T-DM1 | Hypomagnesaemia | all_indication_supportive | 3 | 0.88 (0.28-2.73) | 0.41 | Ineligible |
| T-DM1 | Hysterectomy | all_indication_supportive | 3 | 1.45 (0.47-4.50) | 0.58 | Ineligible |
| T-DM1 | Immune thrombocytopenia | all_indication_supportive | 3 | 2.34 (0.75-7.25) | 0.77 | Ineligible |
| T-DM1 | Immunosuppression | all_indication_supportive | 3 | 1.35 (0.44-4.19) | 0.55 | Ineligible |
| T-DM1 | Inappropriate schedule of product administration | all_indication_supportive | 3 | 0.06 (0.02-0.18) | 0.03 | Ineligible |
| T-DM1 | Infectious pleural effusion | all_indication_supportive | 3 | 7.12 (2.29-22.13) | 1.32 | Ineligible |
| T-DM1 | Joint injury | all_indication_supportive | 3 | 0.57 (0.18-1.77) | 0.29 | Ineligible |
| T-DM1 | Keratitis | all_indication_supportive | 3 | 4.23 (1.36-13.12) | 1.03 | Ineligible |
| T-DM1 | Localised infection | all_indication_supportive | 3 | 0.47 (0.15-1.46) | 0.24 | Ineligible |
| T-DM1 | Loss of personal independence in daily activities | all_indication_supportive | 3 | 0.21 (0.07-0.66) | 0.12 | Ineligible |
| T-DM1 | Lymphopenia | all_indication_supportive | 3 | 0.85 (0.27-2.64) | 0.40 | Ineligible |
| T-DM1 | Macular degeneration | all_indication_supportive | 3 | 1.05 (0.34-3.25) | 0.46 | Ineligible |
| T-DM1 | Maternal exposure before pregnancy | all_indication_supportive | 3 | 1.54 (0.50-4.78) | 0.60 | Ineligible |
| T-DM1 | Mean cell haemoglobin decreased | all_indication_supportive | 3 | 5.94 (1.91-18.43) | 1.20 | Ineligible |
| T-DM1 | Metastases to spinal cord | all_indication_supportive | 3 | 53.76 (17.17-168.34) | 11.52 | Ineligible |
| T-DM1 | Mitral valve incompetence | all_indication_supportive | 3 | 1.46 (0.47-4.53) | 0.58 | Ineligible |
| T-DM1 | Multi-organ failure | all_indication_supportive | 3 | 1.76 (0.57-5.47) | 0.65 | Ineligible |
| T-DM1 | Muscle atrophy | all_indication_supportive | 3 | 1.03 (0.33-3.19) | 0.46 | Ineligible |
| T-DM1 | Musculoskeletal chest pain | all_indication_supportive | 3 | 0.80 (0.26-2.48) | 0.38 | Ineligible |
| T-DM1 | Musculoskeletal disorder | all_indication_supportive | 3 | 0.59 (0.19-1.82) | 0.30 | Ineligible |
| T-DM1 | Myopathy | all_indication_supportive | 3 | 1.59 (0.51-4.93) | 0.61 | Ineligible |
| T-DM1 | Myopia | all_indication_supportive | 3 | 5.59 (1.80-17.34) | 1.17 | Ineligible |
| T-DM1 | Myositis | all_indication_supportive | 3 | 1.50 (0.48-4.66) | 0.59 | Ineligible |
| T-DM1 | Nephrotic syndrome | all_indication_supportive | 3 | 1.97 (0.64-6.12) | 0.70 | Ineligible |
| T-DM1 | Nerve injury | all_indication_supportive | 3 | 1.10 (0.36-3.43) | 0.48 | Ineligible |
| T-DM1 | No therapeutic response | all_indication_supportive | 3 | 1.37 (0.44-4.25) | 0.56 | Ineligible |
| T-DM1 | Oral mucosal blistering | all_indication_supportive | 3 | 1.72 (0.56-5.35) | 0.64 | Ineligible |
| T-DM1 | Osteoarthritis | all_indication_supportive | 3 | 0.28 (0.09-0.88) | 0.15 | Ineligible |
| T-DM1 | Otitis media | all_indication_supportive | 3 | 4.03 (1.30-12.50) | 1.00 | Ineligible |
| T-DM1 | Pallor | all_indication_supportive | 3 | 0.48 (0.15-1.48) | 0.25 | Ineligible |
| T-DM1 | Performance status decreased | all_indication_supportive | 3 | 2.92 (0.94-9.07) | 0.86 | Ineligible |
| T-DM1 | Platelet count abnormal | all_indication_supportive | 3 | 1.86 (0.60-5.76) | 0.67 | Ineligible |
| T-DM1 | Platelet count increased | all_indication_supportive | 3 | 0.97 (0.31-2.99) | 0.44 | Ineligible |
| T-DM1 | Pneumonia aspiration | all_indication_supportive | 3 | 0.50 (0.16-1.56) | 0.26 | Ineligible |
| T-DM1 | Postmastectomy lymphoedema syndrome | all_indication_supportive | 3 | 598.10 (174.22-2053.28) | 29.08 | Ineligible |
| T-DM1 | Premature delivery | all_indication_supportive | 3 | 0.64 (0.21-1.99) | 0.32 | Ineligible |
| T-DM1 | Product use in unapproved indication | all_indication_supportive | 3 | 0.04 (0.01-0.13) | 0.02 | Ineligible |
| T-DM1 | Pulmonary haemorrhage | all_indication_supportive | 3 | 1.76 (0.57-5.47) | 0.65 | Ineligible |
| T-DM1 | Purpura | all_indication_supportive | 3 | 1.66 (0.53-5.14) | 0.63 | Ineligible |
| T-DM1 | Radiation injury | all_indication_supportive | 3 | 13.53 (4.35-42.08) | 1.98 | Ineligible |
| T-DM1 | Rash pruritic | all_indication_supportive | 3 | 0.23 (0.07-0.71) | 0.13 | Ineligible |
| T-DM1 | Rash pustular | all_indication_supportive | 3 | 1.89 (0.61-5.85) | 0.68 | Ineligible |
| T-DM1 | Renal disorder | all_indication_supportive | 3 | 0.26 (0.08-0.81) | 0.14 | Ineligible |
| T-DM1 | Renal failure acute | all_indication_supportive | 3 | 0.83 (0.27-2.57) | 0.39 | Ineligible |
| T-DM1 | Rheumatoid arthritis | all_indication_supportive | 3 | 0.10 (0.03-0.32) | 0.06 | Ineligible |
| T-DM1 | Scab | all_indication_supportive | 3 | 1.03 (0.33-3.20) | 0.46 | Ineligible |
| T-DM1 | Scratch | all_indication_supportive | 3 | 1.12 (0.36-3.47) | 0.48 | Ineligible |
| T-DM1 | Sensory disturbance | all_indication_supportive | 3 | 0.82 (0.26-2.53) | 0.38 | Ineligible |
| T-DM1 | Serum ferritin decreased | all_indication_supportive | 3 | 7.02 (2.26-21.79) | 1.31 | Ineligible |
| T-DM1 | Sjogren's syndrome | all_indication_supportive | 3 | 2.30 (0.74-7.13) | 0.76 | Ineligible |
| T-DM1 | Skin burning sensation | all_indication_supportive | 3 | 0.15 (0.05-0.47) | 0.08 | Ineligible |
| T-DM1 | Skin irritation | all_indication_supportive | 3 | 0.23 (0.07-0.70) | 0.12 | Ineligible |
| T-DM1 | Skin mass | all_indication_supportive | 3 | 1.62 (0.52-5.03) | 0.62 | Ineligible |
| T-DM1 | Skin warm | all_indication_supportive | 3 | 2.13 (0.69-6.61) | 0.73 | Ineligible |
| T-DM1 | Subileus | all_indication_supportive | 3 | 6.66 (2.15-20.69) | 1.27 | Ineligible |
| T-DM1 | Sudden death | all_indication_supportive | 3 | 1.55 (0.50-4.82) | 0.60 | Ineligible |
| T-DM1 | Tachypnoea | all_indication_supportive | 3 | 0.95 (0.31-2.96) | 0.43 | Ineligible |
| T-DM1 | Therapeutic product effect decreased | all_indication_supportive | 3 | 0.23 (0.07-0.71) | 0.13 | Ineligible |
| T-DM1 | Therapy partial responder | all_indication_supportive | 3 | 0.71 (0.23-2.21) | 0.35 | Ineligible |
| T-DM1 | Thermal burn | all_indication_supportive | 3 | 1.88 (0.61-5.84) | 0.68 | Ineligible |
| T-DM1 | Thrombocytopenic purpura | all_indication_supportive | 3 | 12.10 (3.89-37.60) | 1.82 | Ineligible |
| T-DM1 | Thrombosis in device | all_indication_supportive | 3 | 4.60 (1.48-14.28) | 1.07 | Ineligible |
| T-DM1 | Tooth abscess | all_indication_supportive | 3 | 1.24 (0.40-3.84) | 0.52 | Ineligible |
| T-DM1 | Tooth loss | all_indication_supportive | 3 | 0.52 (0.17-1.61) | 0.27 | Ineligible |
| T-DM1 | Transient ischaemic attack | all_indication_supportive | 3 | 0.44 (0.14-1.35) | 0.23 | Ineligible |
| T-DM1 | Tubulointerstitial nephritis | all_indication_supportive | 3 | 0.60 (0.19-1.85) | 0.30 | Ineligible |
| T-DM1 | Ulcer | all_indication_supportive | 3 | 0.81 (0.26-2.52) | 0.38 | Ineligible |
| T-DM1 | Underdose | all_indication_supportive | 3 | 0.14 (0.05-0.44) | 0.08 | Ineligible |
| T-DM1 | Varicose vein | all_indication_supportive | 3 | 2.36 (0.76-7.31) | 0.77 | Ineligible |
| T-DM1 | Venous thrombosis | all_indication_supportive | 3 | 3.37 (1.09-10.47) | 0.92 | Ineligible |
| T-DM1 | Ventricular extrasystoles | all_indication_supportive | 3 | 1.53 (0.49-4.74) | 0.60 | Ineligible |
| T-DM1 | Ventricular fibrillation | all_indication_supportive | 3 | 1.36 (0.44-4.22) | 0.55 | Ineligible |
| T-DM1 | Ventricular tachycardia | all_indication_supportive | 3 | 0.88 (0.28-2.72) | 0.41 | Ineligible |
| T-DM1 | Vitamin D decreased | all_indication_supportive | 3 | 1.34 (0.43-4.16) | 0.55 | Ineligible |
| T-DM1 | Vitamin D deficiency | all_indication_supportive | 3 | 1.18 (0.38-3.67) | 0.50 | Ineligible |
| T-DXd | Death | all_indication_supportive | 1254 | 3.17 (2.99-3.36) | 2.76 | Eligible |
| T-DXd | Nausea | all_indication_supportive | 1190 | 3.50 (3.29-3.72) | 3.05 | Eligible |
| T-DXd | Interstitial lung disease | all_indication_supportive | 1003 | 50.29 (47.08-53.72) | 41.91 | Eligible |
| T-DXd | Disease progression | all_indication_supportive | 835 | 15.63 (14.56-16.78) | 13.52 | Eligible |
| T-DXd | Off label use | all_indication_supportive | 814 | 1.72 (1.60-1.85) | 1.57 | Eligible |
| T-DXd | Fatigue | all_indication_supportive | 798 | 2.12 (1.97-2.28) | 1.91 | Eligible |
| T-DXd | Myelosuppression | all_indication_supportive | 537 | 32.11 (29.41-35.05) | 27.79 | Eligible |
| T-DXd | Vomiting | all_indication_supportive | 525 | 2.50 (2.29-2.73) | 2.25 | Eligible |
| T-DXd | Diarrhoea | all_indication_supportive | 517 | 1.64 (1.50-1.79) | 1.49 | Eligible |
| T-DXd | Pneumonitis | all_indication_supportive | 426 | 33.64 (30.50-37.10) | 29.11 | Eligible |
| T-DXd | Decreased appetite | all_indication_supportive | 367 | 3.23 (2.91-3.59) | 2.87 | Eligible |
| T-DXd | Asthenia | all_indication_supportive | 350 | 2.00 (1.80-2.23) | 1.79 | Eligible |
| T-DXd | Alopecia | all_indication_supportive | 335 | 3.31 (2.97-3.69) | 2.93 | Eligible |
| T-DXd | No adverse event | all_indication_supportive | 325 | 3.52 (3.15-3.93) | 3.11 | Eligible |
| T-DXd | Dyspnoea | all_indication_supportive | 306 | 1.14 (1.02-1.27) | 1.03 | Eligible |
| T-DXd | Malaise | all_indication_supportive | 250 | 1.14 (1.01-1.29) | 1.02 | Eligible |
| T-DXd | Neutropenia | all_indication_supportive | 245 | 3.70 (3.26-4.20) | 3.23 | Eligible |
| T-DXd | Weight decreased | all_indication_supportive | 240 | 1.82 (1.60-2.07) | 1.61 | Eligible |
| T-DXd | Anaemia | all_indication_supportive | 235 | 2.68 (2.35-3.05) | 2.35 | Eligible |
| T-DXd | Intentional product use issue | all_indication_supportive | 227 | 4.51 (3.95-5.14) | 3.91 | Eligible |
| T-DXd | Malignant neoplasm progression | all_indication_supportive | 223 | 4.25 (3.72-4.86) | 3.69 | Eligible |
| T-DXd | Constipation | all_indication_supportive | 210 | 2.00 (1.74-2.29) | 1.75 | Eligible |
| T-DXd | Pneumonia | all_indication_supportive | 209 | 1.32 (1.15-1.52) | 1.17 | Eligible |
| T-DXd | Neutrophil count decreased | all_indication_supportive | 190 | 9.76 (8.46-11.28) | 8.45 | Eligible |
| T-DXd | Metastases to central nervous system | all_indication_supportive | 188 | 30.69 (26.53-35.50) | 26.00 | Eligible |
| T-DXd | Platelet count decreased | all_indication_supportive | 186 | 3.70 (3.20-4.28) | 3.19 | Eligible |
| T-DXd | Toxicity to various agents | all_indication_supportive | 185 | 2.04 (1.77-2.36) | 1.78 | Eligible |
| T-DXd | Pyrexia | all_indication_supportive | 165 | 1.03 (0.88-1.20) | 0.90 | Ineligible |
| T-DXd | Cough | all_indication_supportive | 163 | 1.16 (0.99-1.35) | 1.01 | Ineligible |
| T-DXd | Ascites | all_indication_supportive | 157 | 11.97 (10.22-14.02) | 10.24 | Eligible |
| T-DXd | Thrombocytopenia | all_indication_supportive | 153 | 3.03 (2.59-3.56) | 2.58 | Eligible |
| T-DXd | General physical health deterioration | all_indication_supportive | 143 | 2.60 (2.20-3.06) | 2.21 | Eligible |
| T-DXd | Drug ineffective | all_indication_supportive | 141 | 0.19 (0.17-0.23) | 0.18 | Ineligible |
| T-DXd | Underdose | all_indication_supportive | 141 | 3.45 (2.92-4.08) | 2.91 | Eligible |
| T-DXd | White blood cell count decreased | all_indication_supportive | 139 | 2.62 (2.22-3.10) | 2.23 | Eligible |
| T-DXd | Pleural effusion | all_indication_supportive | 132 | 4.90 (4.13-5.82) | 4.11 | Eligible |
| T-DXd | Febrile neutropenia | all_indication_supportive | 131 | 4.14 (3.48-4.92) | 3.47 | Eligible |
| T-DXd | Pain | all_indication_supportive | 130 | 0.41 (0.35-0.49) | 0.36 | Ineligible |
| T-DXd | COVID-19 | all_indication_supportive | 121 | 1.15 (0.96-1.38) | 0.98 | Ineligible |
| T-DXd | Metastases to liver | all_indication_supportive | 117 | 13.76 (11.46-16.52) | 11.52 | Eligible |
| T-DXd | Headache | all_indication_supportive | 113 | 0.37 (0.30-0.44) | 0.32 | Ineligible |
| T-DXd | Inappropriate schedule of product administration | all_indication_supportive | 106 | 1.06 (0.87-1.28) | 0.90 | Ineligible |
| T-DXd | Ejection fraction decreased | all_indication_supportive | 104 | 14.83 (12.22-18.01) | 12.30 | Eligible |
| T-DXd | Product dose omission issue | all_indication_supportive | 103 | 0.66 (0.55-0.81) | 0.57 | Ineligible |
| T-DXd | Dehydration | all_indication_supportive | 101 | 1.71 (1.41-2.08) | 1.43 | Eligible |
| T-DXd | Sepsis | all_indication_supportive | 101 | 1.96 (1.61-2.38) | 1.63 | Eligible |
| T-DXd | Dizziness | all_indication_supportive | 98 | 0.42 (0.34-0.51) | 0.36 | Ineligible |
| T-DXd | Neuropathy peripheral | all_indication_supportive | 96 | 2.01 (1.64-2.46) | 1.66 | Eligible |
| T-DXd | Pneumocystis jirovecii pneumonia | all_indication_supportive | 95 | 16.69 (13.62-20.45) | 13.70 | Eligible |
| T-DXd | Lung disorder | all_indication_supportive | 94 | 4.01 (3.27-4.91) | 3.25 | Eligible |
| T-DXd | Infection | all_indication_supportive | 88 | 1.21 (0.98-1.49) | 1.01 | Ineligible |
| T-DXd | Pancytopenia | all_indication_supportive | 88 | 3.52 (2.85-4.34) | 2.84 | Eligible |
| T-DXd | Respiratory failure | all_indication_supportive | 88 | 2.76 (2.24-3.41) | 2.24 | Eligible |
| T-DXd | Pulmonary embolism | all_indication_supportive | 87 | 2.21 (1.79-2.73) | 1.81 | Eligible |
| T-DXd | Pulmonary toxicity | all_indication_supportive | 85 | 24.89 (20.07-30.86) | 19.99 | Eligible |
| T-DXd | Abdominal pain | all_indication_supportive | 82 | 0.76 (0.61-0.94) | 0.63 | Ineligible |
| T-DXd | Haemoglobin decreased | all_indication_supportive | 81 | 1.75 (1.40-2.17) | 1.43 | Eligible |
| T-DXd | Fall | all_indication_supportive | 79 | 0.49 (0.40-0.62) | 0.41 | Ineligible |
| T-DXd | Metastases to bone | all_indication_supportive | 77 | 9.37 (7.49-11.74) | 7.61 | Eligible |
| T-DXd | Gastrointestinal disorder | all_indication_supportive | 72 | 1.64 (1.30-2.06) | 1.32 | Eligible |
| T-DXd | Incorrect dose administered | all_indication_supportive | 70 | 0.60 (0.47-0.75) | 0.49 | Ineligible |
| T-DXd | Therapy change | all_indication_supportive | 67 | 12.38 (9.73-15.76) | 9.89 | Eligible |
| T-DXd | Acute kidney injury | all_indication_supportive | 65 | 0.73 (0.57-0.93) | 0.60 | Ineligible |
| T-DXd | Drug intolerance | all_indication_supportive | 65 | 1.23 (0.97-1.57) | 0.99 | Ineligible |
| T-DXd | Hepatic failure | all_indication_supportive | 65 | 5.25 (4.11-6.70) | 4.08 | Eligible |
| T-DXd | Adverse drug reaction | all_indication_supportive | 62 | 1.29 (1.01-1.66) | 1.04 | Eligible |
| T-DXd | Hypotension | all_indication_supportive | 61 | 0.65 (0.51-0.84) | 0.53 | Ineligible |
| T-DXd | Weight increased | all_indication_supportive | 61 | 0.58 (0.45-0.75) | 0.47 | Ineligible |
| T-DXd | Feeling abnormal | all_indication_supportive | 60 | 0.52 (0.40-0.67) | 0.42 | Ineligible |
| T-DXd | Rash | all_indication_supportive | 60 | 0.28 (0.21-0.36) | 0.23 | Ineligible |
| T-DXd | Urinary tract infection | all_indication_supportive | 60 | 0.71 (0.55-0.91) | 0.57 | Ineligible |
| T-DXd | Hepatic function abnormal | all_indication_supportive | 58 | 3.51 (2.71-4.55) | 2.68 | Eligible |
| T-DXd | Hypokalaemia | all_indication_supportive | 57 | 2.74 (2.11-3.55) | 2.11 | Eligible |
| T-DXd | Back pain | all_indication_supportive | 56 | 0.50 (0.38-0.64) | 0.40 | Ineligible |
| T-DXd | Hepatotoxicity | all_indication_supportive | 53 | 5.04 (3.85-6.60) | 3.79 | Eligible |
| T-DXd | Pain in extremity | all_indication_supportive | 52 | 0.36 (0.28-0.47) | 0.29 | Ineligible |
| T-DXd | Product use issue | all_indication_supportive | 52 | 0.48 (0.36-0.63) | 0.38 | Ineligible |
| T-DXd | Septic shock | all_indication_supportive | 52 | 2.58 (1.97-3.39) | 1.97 | Eligible |
| T-DXd | Abdominal pain upper | all_indication_supportive | 51 | 0.52 (0.39-0.68) | 0.41 | Ineligible |
| T-DXd | Condition aggravated | all_indication_supportive | 51 | 0.33 (0.25-0.43) | 0.27 | Ineligible |
| T-DXd | Illness | all_indication_supportive | 51 | 0.92 (0.70-1.21) | 0.72 | Ineligible |
| T-DXd | Cardiac failure | all_indication_supportive | 50 | 1.34 (1.02-1.78) | 1.05 | Eligible |
| T-DXd | Oedema peripheral | all_indication_supportive | 50 | 1.17 (0.88-1.54) | 0.91 | Ineligible |
| T-DXd | Seizure | all_indication_supportive | 50 | 0.75 (0.57-1.00) | 0.60 | Ineligible |
| T-DXd | Vision blurred | all_indication_supportive | 50 | 0.80 (0.60-1.05) | 0.63 | Ineligible |
| T-DXd | Metastases to meninges | all_indication_supportive | 49 | 44.10 (33.18-58.61) | 31.95 | Eligible |
| T-DXd | Adverse event | all_indication_supportive | 48 | 1.05 (0.79-1.40) | 0.82 | Ineligible |
| T-DXd | Blood bilirubin increased | all_indication_supportive | 48 | 4.52 (3.40-6.00) | 3.33 | Eligible |
| T-DXd | Dyspepsia | all_indication_supportive | 48 | 1.06 (0.80-1.41) | 0.83 | Ineligible |
| T-DXd | Renal failure | all_indication_supportive | 48 | 0.80 (0.60-1.06) | 0.63 | Ineligible |
| T-DXd | Hepatic cytolysis | all_indication_supportive | 47 | 7.15 (5.37-9.53) | 5.41 | Eligible |
| T-DXd | Abdominal distension | all_indication_supportive | 45 | 0.91 (0.68-1.22) | 0.71 | Ineligible |
| T-DXd | Colitis | all_indication_supportive | 45 | 2.40 (1.79-3.22) | 1.79 | Eligible |
| T-DXd | Metastases to lung | all_indication_supportive | 45 | 7.86 (5.86-10.54) | 5.95 | Eligible |
| T-DXd | Hepatic enzyme increased | all_indication_supportive | 44 | 1.36 (1.01-1.83) | 1.04 | Eligible |
| T-DXd | Hyponatraemia | all_indication_supportive | 44 | 1.75 (1.30-2.35) | 1.32 | Eligible |
| T-DXd | Peripheral swelling | all_indication_supportive | 44 | 0.49 (0.36-0.66) | 0.39 | Ineligible |
| T-DXd | Breast cancer | all_indication_supportive | 43 | 0.96 (0.71-1.30) | 0.74 | Ineligible |
| T-DXd | Somnolence | all_indication_supportive | 43 | 0.46 (0.34-0.61) | 0.36 | Ineligible |
| T-DXd | Abdominal discomfort | all_indication_supportive | 42 | 0.48 (0.35-0.65) | 0.38 | Ineligible |
| T-DXd | Renal impairment | all_indication_supportive | 42 | 1.03 (0.76-1.39) | 0.79 | Ineligible |
| T-DXd | Drug-induced liver injury | all_indication_supportive | 41 | 2.38 (1.75-3.23) | 1.75 | Eligible |
| T-DXd | Lung opacity | all_indication_supportive | 41 | 29.33 (21.52-39.96) | 21.25 | Eligible |
| T-DXd | Alanine aminotransferase increased | all_indication_supportive | 40 | 1.71 (1.25-2.33) | 1.28 | Eligible |
| T-DXd | Chills | all_indication_supportive | 40 | 0.73 (0.54-1.00) | 0.56 | Ineligible |
| T-DXd | Liver disorder | all_indication_supportive | 40 | 2.02 (1.48-2.75) | 1.49 | Eligible |
| T-DXd | Stomatitis | all_indication_supportive | 40 | 1.31 (0.96-1.79) | 0.99 | Ineligible |
| T-DXd | Unevaluable event | all_indication_supportive | 40 | 1.07 (0.79-1.47) | 0.82 | Ineligible |
| T-DXd | Mucosal inflammation | all_indication_supportive | 39 | 3.24 (2.36-4.44) | 2.32 | Eligible |
| T-DXd | Pulmonary oedema | all_indication_supportive | 39 | 1.94 (1.42-2.66) | 1.43 | Eligible |
| T-DXd | Hospitalisation | all_indication_supportive | 38 | 0.45 (0.33-0.62) | 0.35 | Ineligible |
| T-DXd | Pulmonary fibrosis | all_indication_supportive | 38 | 4.30 (3.12-5.91) | 3.03 | Eligible |
| T-DXd | Anxiety | all_indication_supportive | 37 | 0.28 (0.20-0.38) | 0.22 | Ineligible |
| T-DXd | Infusion related reaction | all_indication_supportive | 37 | 1.14 (0.82-1.57) | 0.85 | Ineligible |
| T-DXd | Pneumonia bacterial | all_indication_supportive | 37 | 8.15 (5.90-11.27) | 5.99 | Eligible |
| T-DXd | Aspartate aminotransferase increased | all_indication_supportive | 36 | 1.85 (1.34-2.57) | 1.36 | Eligible |
| T-DXd | Gait disturbance | all_indication_supportive | 36 | 0.38 (0.27-0.52) | 0.29 | Ineligible |
| T-DXd | Thrombosis | all_indication_supportive | 36 | 0.97 (0.70-1.34) | 0.73 | Ineligible |
| T-DXd | Oxygen saturation decreased | all_indication_supportive | 34 | 1.23 (0.88-1.73) | 0.91 | Ineligible |
| T-DXd | Arthralgia | all_indication_supportive | 33 | 0.16 (0.11-0.22) | 0.12 | Ineligible |
| T-DXd | Chest pain | all_indication_supportive | 33 | 0.41 (0.29-0.58) | 0.32 | Ineligible |
| T-DXd | Pruritus | all_indication_supportive | 33 | 0.18 (0.12-0.25) | 0.14 | Ineligible |
| T-DXd | Cholestasis | all_indication_supportive | 32 | 3.73 (2.64-5.28) | 2.54 | Eligible |
| T-DXd | Gastrointestinal toxicity | all_indication_supportive | 32 | 13.69 (9.66-19.40) | 9.88 | Eligible |
| T-DXd | Haematotoxicity | all_indication_supportive | 32 | 6.81 (4.81-9.64) | 4.76 | Eligible |
| T-DXd | Cytopenia | all_indication_supportive | 31 | 4.62 (3.24-6.57) | 3.10 | Eligible |
| T-DXd | Feeding disorder | all_indication_supportive | 31 | 2.83 (1.99-4.03) | 1.95 | Eligible |
| T-DXd | Hepatic cirrhosis | all_indication_supportive | 31 | 3.53 (2.48-5.02) | 2.39 | Eligible |
| T-DXd | Loss of consciousness | all_indication_supportive | 31 | 0.56 (0.40-0.80) | 0.42 | Ineligible |
| T-DXd | Muscle spasms | all_indication_supportive | 31 | 0.35 (0.24-0.50) | 0.26 | Ineligible |
| T-DXd | Taste disorder | all_indication_supportive | 31 | 2.74 (1.93-3.90) | 1.90 | Eligible |
| T-DXd | Therapeutic response unexpected | all_indication_supportive | 31 | 1.29 (0.91-1.84) | 0.94 | Ineligible |
| T-DXd | Visual impairment | all_indication_supportive | 31 | 0.49 (0.34-0.69) | 0.37 | Ineligible |
| T-DXd | Gastrooesophageal reflux disease | all_indication_supportive | 30 | 0.82 (0.57-1.17) | 0.60 | Ineligible |
| T-DXd | Myalgia | all_indication_supportive | 30 | 0.39 (0.27-0.55) | 0.29 | Ineligible |
| T-DXd | Paraesthesia | all_indication_supportive | 30 | 0.41 (0.29-0.59) | 0.31 | Ineligible |
| T-DXd | Dry eye | all_indication_supportive | 29 | 1.18 (0.82-1.70) | 0.85 | Ineligible |
| T-DXd | Insomnia | all_indication_supportive | 29 | 0.23 (0.16-0.34) | 0.18 | Ineligible |
| T-DXd | Leukopenia | all_indication_supportive | 29 | 1.27 (0.88-1.83) | 0.92 | Ineligible |
| T-DXd | Lung infiltration | all_indication_supportive | 29 | 9.94 (6.90-14.33) | 7.07 | Eligible |
| T-DXd | Product administration error | all_indication_supportive | 29 | 2.55 (1.77-3.68) | 1.75 | Eligible |
| T-DXd | Quality of life decreased | all_indication_supportive | 29 | 4.94 (3.43-7.11) | 3.26 | Eligible |
| T-DXd | Therapy cessation | all_indication_supportive | 29 | 0.99 (0.69-1.42) | 0.72 | Ineligible |
| T-DXd | Chest discomfort | all_indication_supportive | 28 | 0.58 (0.40-0.85) | 0.43 | Ineligible |
| T-DXd | Erythema | all_indication_supportive | 27 | 0.26 (0.18-0.38) | 0.20 | Ineligible |
| T-DXd | Hypersomnia | all_indication_supportive | 27 | 1.98 (1.36-2.89) | 1.37 | Eligible |
| T-DXd | Pneumothorax | all_indication_supportive | 27 | 3.53 (2.42-5.15) | 2.32 | Eligible |
| T-DXd | Acute respiratory failure | all_indication_supportive | 26 | 2.76 (1.88-4.05) | 1.83 | Eligible |
| T-DXd | Cardiotoxicity | all_indication_supportive | 26 | 5.73 (3.89-8.42) | 3.71 | Eligible |
| T-DXd | Cataract | all_indication_supportive | 26 | 0.88 (0.60-1.30) | 0.63 | Ineligible |
| T-DXd | Metastases to lymph nodes | all_indication_supportive | 26 | 7.99 (5.43-11.75) | 5.44 | Eligible |
| T-DXd | Blood creatinine increased | all_indication_supportive | 25 | 0.89 (0.60-1.32) | 0.64 | Ineligible |
| T-DXd | Cardiac disorder | all_indication_supportive | 25 | 0.59 (0.40-0.88) | 0.43 | Ineligible |
| T-DXd | Epistaxis | all_indication_supportive | 25 | 0.68 (0.46-1.01) | 0.49 | Ineligible |
| T-DXd | Hypoxia | all_indication_supportive | 25 | 1.56 (1.05-2.31) | 1.08 | Eligible |
| T-DXd | Mobility decreased | all_indication_supportive | 25 | 0.68 (0.46-1.01) | 0.49 | Ineligible |
| T-DXd | Muscular weakness | all_indication_supportive | 25 | 0.47 (0.32-0.70) | 0.34 | Ineligible |
| T-DXd | Nasopharyngitis | all_indication_supportive | 25 | 0.26 (0.18-0.38) | 0.19 | Ineligible |
| T-DXd | Acute respiratory distress syndrome | all_indication_supportive | 24 | 3.20 (2.14-4.78) | 2.06 | Eligible |
| T-DXd | Blood pressure decreased | all_indication_supportive | 24 | 0.81 (0.54-1.21) | 0.58 | Ineligible |
| T-DXd | Brain fog | all_indication_supportive | 24 | 3.26 (2.19-4.87) | 2.09 | Eligible |
| T-DXd | Cell death | all_indication_supportive | 24 | 25.13 (16.78-37.62) | 16.58 | Eligible |
| T-DXd | Dysphagia | all_indication_supportive | 24 | 0.54 (0.36-0.81) | 0.39 | Ineligible |
| T-DXd | Hypophagia | all_indication_supportive | 24 | 1.94 (1.30-2.89) | 1.31 | Eligible |
| T-DXd | Lymphoedema | all_indication_supportive | 24 | 7.07 (4.74-10.57) | 4.62 | Eligible |
| T-DXd | Oropharyngeal pain | all_indication_supportive | 24 | 0.51 (0.34-0.76) | 0.37 | Ineligible |
| T-DXd | Tumour marker increased | all_indication_supportive | 24 | 9.33 (6.24-13.94) | 6.35 | Eligible |
| T-DXd | Blood potassium decreased | all_indication_supportive | 23 | 1.70 (1.13-2.56) | 1.15 | Eligible |
| T-DXd | Cardiac arrest | all_indication_supportive | 23 | 0.67 (0.44-1.01) | 0.48 | Ineligible |
| T-DXd | Confusional state | all_indication_supportive | 23 | 0.31 (0.21-0.47) | 0.23 | Ineligible |
| T-DXd | Hypoaesthesia | all_indication_supportive | 23 | 0.33 (0.22-0.49) | 0.24 | Ineligible |
| T-DXd | Intestinal obstruction | all_indication_supportive | 23 | 1.26 (0.84-1.90) | 0.87 | Ineligible |
| T-DXd | Neoplasm progression | all_indication_supportive | 23 | 1.21 (0.80-1.82) | 0.84 | Ineligible |
| T-DXd | Cellulitis | all_indication_supportive | 22 | 0.92 (0.61-1.40) | 0.64 | Ineligible |
| T-DXd | Jaundice | all_indication_supportive | 22 | 2.09 (1.37-3.17) | 1.37 | Eligible |
| T-DXd | Sleep disorder | all_indication_supportive | 22 | 0.63 (0.42-0.96) | 0.45 | Ineligible |
| T-DXd | Brain oedema | all_indication_supportive | 21 | 3.86 (2.51-5.93) | 2.35 | Eligible |
| T-DXd | Cardiac dysfunction | all_indication_supportive | 21 | 10.08 (6.56-15.49) | 6.69 | Eligible |
| T-DXd | Liver function test increased | all_indication_supportive | 21 | 1.71 (1.11-2.62) | 1.13 | Eligible |
| T-DXd | Respiratory disorder | all_indication_supportive | 21 | 1.51 (0.99-2.32) | 1.01 | Ineligible |
| T-DXd | Transaminases increased | all_indication_supportive | 21 | 2.02 (1.31-3.10) | 1.31 | Eligible |
| T-DXd | Dry skin | all_indication_supportive | 20 | 0.28 (0.18-0.43) | 0.20 | Ineligible |
| T-DXd | Gastrointestinal haemorrhage | all_indication_supportive | 20 | 0.49 (0.32-0.76) | 0.35 | Ineligible |
| T-DXd | Hypertension | all_indication_supportive | 20 | 0.20 (0.13-0.31) | 0.15 | Ineligible |
| T-DXd | Influenza | all_indication_supportive | 20 | 0.34 (0.22-0.53) | 0.24 | Ineligible |
| T-DXd | Oedema | all_indication_supportive | 20 | 0.80 (0.52-1.24) | 0.55 | Ineligible |
| T-DXd | Radiation necrosis | all_indication_supportive | 20 | 68.17 (43.56-106.67) | 37.09 | Eligible |
| T-DXd | Tachycardia | all_indication_supportive | 20 | 0.50 (0.32-0.78) | 0.35 | Ineligible |
| T-DXd | Bone pain | all_indication_supportive | 19 | 0.67 (0.43-1.06) | 0.46 | Ineligible |
| T-DXd | Cerebral haemorrhage | all_indication_supportive | 19 | 1.13 (0.72-1.78) | 0.76 | Ineligible |
| T-DXd | Dysarthria | all_indication_supportive | 19 | 1.17 (0.75-1.84) | 0.78 | Ineligible |
| T-DXd | Femur fracture | all_indication_supportive | 19 | 1.43 (0.91-2.24) | 0.94 | Ineligible |
| T-DXd | Flushing | all_indication_supportive | 19 | 0.46 (0.29-0.71) | 0.32 | Ineligible |
| T-DXd | Lymphangiosis carcinomatosa | all_indication_supportive | 19 | 41.14 (26.08-64.88) | 24.15 | Eligible |
| T-DXd | Memory impairment | all_indication_supportive | 19 | 0.27 (0.17-0.43) | 0.19 | Ineligible |
| T-DXd | Prescribed underdose | all_indication_supportive | 19 | 1.64 (1.05-2.57) | 1.07 | Eligible |
| T-DXd | Blood glucose increased | all_indication_supportive | 18 | 0.21 (0.13-0.33) | 0.15 | Ineligible |
| T-DXd | Depression | all_indication_supportive | 18 | 0.18 (0.11-0.29) | 0.13 | Ineligible |
| T-DXd | Pneumonia aspiration | all_indication_supportive | 18 | 1.54 (0.97-2.45) | 1.00 | Ineligible |
| T-DXd | Product use in unapproved indication | all_indication_supportive | 18 | 0.13 (0.08-0.20) | 0.09 | Ineligible |
| T-DXd | Syncope | all_indication_supportive | 18 | 0.40 (0.25-0.64) | 0.28 | Ineligible |
| T-DXd | Therapy interrupted | all_indication_supportive | 18 | 0.47 (0.30-0.75) | 0.33 | Ineligible |
| T-DXd | Therapy partial responder | all_indication_supportive | 18 | 2.19 (1.38-3.47) | 1.36 | Eligible |
| T-DXd | Wrong technique in product usage process | all_indication_supportive | 18 | 0.15 (0.09-0.24) | 0.11 | Ineligible |
| T-DXd | Bedridden | all_indication_supportive | 17 | 2.78 (1.72-4.47) | 1.65 | Eligible |
| T-DXd | Dysgeusia | all_indication_supportive | 17 | 0.51 (0.32-0.82) | 0.35 | Ineligible |
| T-DXd | Electrolyte imbalance | all_indication_supportive | 17 | 3.21 (2.00-5.17) | 1.87 | Eligible |
| T-DXd | Flatulence | all_indication_supportive | 17 | 0.66 (0.41-1.06) | 0.44 | Ineligible |
| T-DXd | Haemorrhage | all_indication_supportive | 17 | 0.33 (0.21-0.54) | 0.23 | Ineligible |
| T-DXd | Hospice care | all_indication_supportive | 17 | 3.05 (1.89-4.91) | 1.78 | Eligible |
| T-DXd | Hypersensitivity | all_indication_supportive | 17 | 0.18 (0.11-0.30) | 0.13 | Ineligible |
| T-DXd | Lethargy | all_indication_supportive | 17 | 0.66 (0.41-1.06) | 0.45 | Ineligible |
| T-DXd | Liver injury | all_indication_supportive | 17 | 1.41 (0.88-2.27) | 0.91 | Ineligible |
| T-DXd | Pericardial effusion | all_indication_supportive | 17 | 1.60 (1.00-2.58) | 1.02 | Ineligible |
| T-DXd | Balance disorder | all_indication_supportive | 16 | 0.39 (0.24-0.63) | 0.27 | Ineligible |
| T-DXd | Blood alkaline phosphatase increased | all_indication_supportive | 16 | 1.84 (1.13-3.00) | 1.13 | Eligible |
| T-DXd | Blood pressure increased | all_indication_supportive | 16 | 0.22 (0.13-0.36) | 0.15 | Ineligible |
| T-DXd | Breast cancer metastatic | all_indication_supportive | 16 | 3.74 (2.29-6.10) | 2.09 | Eligible |
| T-DXd | Clostridium difficile infection | all_indication_supportive | 16 | 1.29 (0.79-2.10) | 0.82 | Ineligible |
| T-DXd | Deep vein thrombosis | all_indication_supportive | 16 | 0.60 (0.37-0.97) | 0.40 | Ineligible |
| T-DXd | Disseminated intravascular coagulation | all_indication_supportive | 16 | 3.01 (1.84-4.92) | 1.73 | Eligible |
| T-DXd | Gamma-glutamyltransferase increased | all_indication_supportive | 16 | 1.98 (1.21-3.23) | 1.21 | Eligible |
| T-DXd | Immunodeficiency | all_indication_supportive | 16 | 1.99 (1.22-3.25) | 1.21 | Eligible |
| T-DXd | Metastases to skin | all_indication_supportive | 16 | 30.50 (18.60-50.04) | 17.74 | Eligible |
| T-DXd | Palmar-plantar erythrodysaesthesia syndrome | all_indication_supportive | 16 | 1.40 (0.86-2.29) | 0.89 | Ineligible |
| T-DXd | Palpitations | all_indication_supportive | 16 | 0.30 (0.18-0.49) | 0.21 | Ineligible |
| T-DXd | Red blood cell count decreased | all_indication_supportive | 16 | 1.16 (0.71-1.89) | 0.75 | Ineligible |
| T-DXd | Upper gastrointestinal haemorrhage | all_indication_supportive | 16 | 1.86 (1.14-3.04) | 1.14 | Eligible |
| T-DXd | Weight abnormal | all_indication_supportive | 16 | 12.30 (7.52-20.12) | 7.67 | Eligible |
| T-DXd | Blood sodium decreased | all_indication_supportive | 15 | 1.91 (1.15-3.18) | 1.15 | Eligible |
| T-DXd | Drug effective for unapproved indication | all_indication_supportive | 15 | 0.88 (0.53-1.46) | 0.57 | Ineligible |
| T-DXd | Dry mouth | all_indication_supportive | 15 | 0.40 (0.24-0.66) | 0.27 | Ineligible |
| T-DXd | Dyspnoea exertional | all_indication_supportive | 15 | 0.79 (0.48-1.32) | 0.52 | Ineligible |
| T-DXd | Full blood count decreased | all_indication_supportive | 15 | 1.33 (0.80-2.21) | 0.83 | Ineligible |
| T-DXd | Gait inability | all_indication_supportive | 15 | 0.79 (0.47-1.31) | 0.51 | Ineligible |
| T-DXd | Generalised oedema | all_indication_supportive | 15 | 2.88 (1.73-4.78) | 1.63 | Eligible |
| T-DXd | Herpes zoster | all_indication_supportive | 15 | 0.52 (0.31-0.86) | 0.35 | Ineligible |
| T-DXd | Hyperglycaemia | all_indication_supportive | 15 | 0.96 (0.58-1.60) | 0.62 | Ineligible |
| T-DXd | Hypoalbuminaemia | all_indication_supportive | 15 | 4.50 (2.71-7.47) | 2.40 | Eligible |
| T-DXd | Lower respiratory tract infection | all_indication_supportive | 15 | 0.63 (0.38-1.05) | 0.42 | Ineligible |
| T-DXd | Lymphocyte count decreased | all_indication_supportive | 15 | 1.61 (0.97-2.67) | 0.99 | Ineligible |
| T-DXd | Multiple organ dysfunction syndrome | all_indication_supportive | 15 | 0.95 (0.57-1.57) | 0.61 | Ineligible |
| T-DXd | Myocardial infarction | all_indication_supportive | 15 | 0.23 (0.14-0.39) | 0.16 | Ineligible |
| T-DXd | Neoplasm | all_indication_supportive | 15 | 2.56 (1.54-4.25) | 1.48 | Eligible |
| T-DXd | Osteonecrosis of jaw | all_indication_supportive | 15 | 1.23 (0.74-2.04) | 0.78 | Ineligible |
| T-DXd | Pulmonary mass | all_indication_supportive | 15 | 1.84 (1.11-3.05) | 1.11 | Eligible |
| T-DXd | Radiation pneumonitis | all_indication_supportive | 15 | 13.87 (8.34-23.07) | 8.50 | Eligible |
| T-DXd | Agranulocytosis | all_indication_supportive | 14 | 1.65 (0.98-2.78) | 0.99 | Ineligible |
| T-DXd | COVID-19 pneumonia | all_indication_supportive | 14 | 2.00 (1.18-3.38) | 1.17 | Eligible |
| T-DXd | Chromaturia | all_indication_supportive | 14 | 1.36 (0.80-2.29) | 0.83 | Ineligible |
| T-DXd | Discomfort | all_indication_supportive | 14 | 0.44 (0.26-0.74) | 0.29 | Ineligible |
| T-DXd | Epilepsy | all_indication_supportive | 14 | 0.97 (0.57-1.64) | 0.62 | Ineligible |
| T-DXd | Haematological infection | all_indication_supportive | 14 | 11.82 (6.98-20.00) | 7.07 | Eligible |
| T-DXd | Heart rate increased | all_indication_supportive | 14 | 0.31 (0.19-0.53) | 0.21 | Ineligible |
| T-DXd | Hepatitis | all_indication_supportive | 14 | 1.30 (0.77-2.19) | 0.80 | Ineligible |
| T-DXd | Hypoglycaemia | all_indication_supportive | 14 | 0.68 (0.40-1.15) | 0.44 | Ineligible |
| T-DXd | Loss of personal independence in daily activities | all_indication_supportive | 14 | 0.51 (0.30-0.86) | 0.34 | Ineligible |
| T-DXd | Product prescribing error | all_indication_supportive | 14 | 1.03 (0.61-1.75) | 0.65 | Ineligible |
| T-DXd | Respiratory distress | all_indication_supportive | 14 | 1.18 (0.70-1.99) | 0.74 | Ineligible |
| T-DXd | Tremor | all_indication_supportive | 14 | 0.19 (0.11-0.32) | 0.13 | Ineligible |
| T-DXd | Ageusia | all_indication_supportive | 13 | 1.19 (0.69-2.05) | 0.73 | Ineligible |
| T-DXd | Bacterial infection | all_indication_supportive | 13 | 1.50 (0.87-2.58) | 0.89 | Ineligible |
| T-DXd | C-reactive protein increased | all_indication_supportive | 13 | 0.75 (0.44-1.30) | 0.48 | Ineligible |
| T-DXd | Clostridium difficile colitis | all_indication_supportive | 13 | 2.61 (1.51-4.49) | 1.43 | Eligible |
| T-DXd | Device related infection | all_indication_supportive | 13 | 1.65 (0.96-2.85) | 0.97 | Ineligible |
| T-DXd | Diabetes mellitus | all_indication_supportive | 13 | 0.41 (0.24-0.70) | 0.27 | Ineligible |
| T-DXd | Eating disorder | all_indication_supportive | 13 | 1.26 (0.73-2.17) | 0.76 | Ineligible |
| T-DXd | Enterocolitis | all_indication_supportive | 13 | 4.62 (2.68-7.96) | 2.32 | Eligible |
| T-DXd | Gastritis | all_indication_supportive | 13 | 1.15 (0.67-1.97) | 0.70 | Ineligible |
| T-DXd | Haematemesis | all_indication_supportive | 13 | 1.17 (0.68-2.02) | 0.72 | Ineligible |
| T-DXd | Hypocalcaemia | all_indication_supportive | 13 | 1.48 (0.86-2.55) | 0.88 | Ineligible |
| T-DXd | Hypomagnesaemia | all_indication_supportive | 13 | 1.95 (1.13-3.36) | 1.12 | Eligible |
| T-DXd | Impaired quality of life | all_indication_supportive | 13 | 1.53 (0.89-2.64) | 0.91 | Ineligible |
| T-DXd | Inflammation | all_indication_supportive | 13 | 0.51 (0.29-0.87) | 0.33 | Ineligible |
| T-DXd | Laboratory test abnormal | all_indication_supportive | 13 | 0.83 (0.48-1.43) | 0.52 | Ineligible |
| T-DXd | Neoplasm malignant | all_indication_supportive | 13 | 0.39 (0.23-0.67) | 0.26 | Ineligible |
| T-DXd | Product preparation error | all_indication_supportive | 13 | 1.06 (0.62-1.83) | 0.66 | Ineligible |
| T-DXd | Skin discolouration | all_indication_supportive | 13 | 0.58 (0.34-1.00) | 0.37 | Ineligible |
| T-DXd | Drug resistance | all_indication_supportive | 12 | 0.86 (0.49-1.51) | 0.53 | Ineligible |
| T-DXd | Dysphonia | all_indication_supportive | 12 | 0.42 (0.24-0.73) | 0.27 | Ineligible |
| T-DXd | Eastern Cooperative Oncology Group performance status worsened | all_indication_supportive | 12 | 17.38 (9.84-30.71) | 9.85 | Eligible |
| T-DXd | Failure to thrive | all_indication_supportive | 12 | 5.72 (3.24-10.08) | 2.74 | Eligible |
| T-DXd | Haemoptysis | all_indication_supportive | 12 | 0.92 (0.52-1.62) | 0.57 | Ineligible |
| T-DXd | Hepatic lesion | all_indication_supportive | 12 | 5.89 (3.34-10.38) | 2.82 | Eligible |
| T-DXd | Liver function test abnormal | all_indication_supportive | 12 | 1.14 (0.65-2.01) | 0.69 | Ineligible |
| T-DXd | Mental impairment | all_indication_supportive | 12 | 1.07 (0.61-1.88) | 0.65 | Ineligible |
| T-DXd | Metastases to spine | all_indication_supportive | 12 | 7.32 (4.15-12.91) | 3.63 | Eligible |
| T-DXd | Nervous system disorder | all_indication_supportive | 12 | 1.17 (0.66-2.06) | 0.70 | Ineligible |
| T-DXd | Ocular toxicity | all_indication_supportive | 12 | 23.73 (13.42-41.97) | 13.01 | Eligible |
| T-DXd | Pulmonary thrombosis | all_indication_supportive | 12 | 2.17 (1.23-3.82) | 1.20 | Eligible |
| T-DXd | Splenomegaly | all_indication_supportive | 12 | 2.23 (1.26-3.93) | 1.22 | Eligible |
| T-DXd | Tumour lysis syndrome | all_indication_supportive | 12 | 2.78 (1.58-4.90) | 1.47 | Eligible |
| T-DXd | Visual acuity reduced | all_indication_supportive | 12 | 0.93 (0.53-1.63) | 0.57 | Ineligible |
| T-DXd | Altered state of consciousness | all_indication_supportive | 11 | 1.06 (0.58-1.91) | 0.63 | Ineligible |
| T-DXd | Amnesia | all_indication_supportive | 11 | 0.39 (0.22-0.71) | 0.25 | Ineligible |
| T-DXd | Atrial fibrillation | all_indication_supportive | 11 | 0.24 (0.13-0.44) | 0.16 | Ineligible |
| T-DXd | Blood albumin decreased | all_indication_supportive | 11 | 3.78 (2.09-6.84) | 1.82 | Eligible |
| T-DXd | Blood lactate dehydrogenase increased | all_indication_supportive | 11 | 1.90 (1.05-3.44) | 1.04 | Eligible |
| T-DXd | Blood magnesium decreased | all_indication_supportive | 11 | 2.59 (1.44-4.69) | 1.34 | Eligible |
| T-DXd | Cancer pain | all_indication_supportive | 11 | 8.09 (4.47-14.63) | 3.92 | Eligible |
| T-DXd | Cardiac failure congestive | all_indication_supportive | 11 | 0.33 (0.18-0.60) | 0.21 | Ineligible |
| T-DXd | Cardiomyopathy | all_indication_supportive | 11 | 1.78 (0.99-3.22) | 0.99 | Ineligible |
| T-DXd | Cholangitis | all_indication_supportive | 11 | 3.83 (2.12-6.93) | 1.84 | Eligible |
| T-DXd | Coronavirus infection | all_indication_supportive | 11 | 2.79 (1.54-5.05) | 1.43 | Eligible |
| T-DXd | Delirium | all_indication_supportive | 11 | 0.69 (0.38-1.25) | 0.43 | Ineligible |
| T-DXd | Disease complication | all_indication_supportive | 11 | 4.64 (2.57-8.39) | 2.16 | Eligible |
| T-DXd | Drug hypersensitivity | all_indication_supportive | 11 | 0.10 (0.06-0.18) | 0.07 | Ineligible |
| T-DXd | Fracture | all_indication_supportive | 11 | 1.15 (0.63-2.07) | 0.67 | Ineligible |
| T-DXd | Haemolytic anaemia | all_indication_supportive | 11 | 2.95 (1.63-5.34) | 1.49 | Eligible |
| T-DXd | Hip fracture | all_indication_supportive | 11 | 0.72 (0.40-1.30) | 0.44 | Ineligible |
| T-DXd | Hyperbilirubinaemia | all_indication_supportive | 11 | 2.30 (1.27-4.15) | 1.22 | Eligible |
| T-DXd | Ileus | all_indication_supportive | 11 | 2.06 (1.14-3.72) | 1.11 | Eligible |
| T-DXd | Keratitis | all_indication_supportive | 11 | 7.96 (4.40-14.39) | 3.84 | Eligible |
| T-DXd | Mental disorder | all_indication_supportive | 11 | 0.56 (0.31-1.02) | 0.35 | Ineligible |
| T-DXd | Metastases to peritoneum | all_indication_supportive | 11 | 8.76 (4.84-15.85) | 4.36 | Eligible |
| T-DXd | Metastasis | all_indication_supportive | 11 | 3.17 (1.75-5.73) | 1.58 | Eligible |
| T-DXd | Organising pneumonia | all_indication_supportive | 11 | 4.39 (2.43-7.93) | 2.06 | Eligible |
| T-DXd | Pneumocystis jirovecii infection | all_indication_supportive | 11 | 17.20 (9.49-31.17) | 9.48 | Eligible |
| T-DXd | Portal hypertension | all_indication_supportive | 11 | 8.20 (4.53-14.83) | 3.99 | Eligible |
| T-DXd | Productive cough | all_indication_supportive | 11 | 0.45 (0.25-0.81) | 0.29 | Ineligible |
| T-DXd | Pulmonary hypertension | all_indication_supportive | 11 | 1.18 (0.65-2.12) | 0.69 | Ineligible |
| T-DXd | Rhinorrhoea | all_indication_supportive | 11 | 0.33 (0.18-0.60) | 0.21 | Ineligible |
| T-DXd | Swelling | all_indication_supportive | 11 | 0.23 (0.13-0.42) | 0.15 | Ineligible |
| T-DXd | Treatment noncompliance | all_indication_supportive | 11 | 0.42 (0.23-0.76) | 0.27 | Ineligible |
| T-DXd | Wheezing | all_indication_supportive | 11 | 0.37 (0.21-0.67) | 0.24 | Ineligible |
| T-DXd | Blindness | all_indication_supportive | 10 | 0.52 (0.28-0.96) | 0.32 | Ineligible |
| T-DXd | Blood test abnormal | all_indication_supportive | 10 | 1.34 (0.72-2.50) | 0.75 | Ineligible |
| T-DXd | Brain neoplasm | all_indication_supportive | 10 | 2.22 (1.20-4.13) | 1.15 | Eligible |
| T-DXd | Cerebrovascular accident | all_indication_supportive | 10 | 0.14 (0.08-0.26) | 0.09 | Ineligible |
| T-DXd | Cognitive disorder | all_indication_supportive | 10 | 0.44 (0.23-0.81) | 0.27 | Ineligible |
| T-DXd | Computerised tomogram abnormal | all_indication_supportive | 10 | 14.36 (7.70-26.76) | 7.67 | Eligible |
| T-DXd | Full blood count abnormal | all_indication_supportive | 10 | 1.68 (0.90-3.12) | 0.91 | Ineligible |
| T-DXd | Gastric cancer | all_indication_supportive | 10 | 0.69 (0.37-1.27) | 0.41 | Ineligible |
| T-DXd | Hair growth abnormal | all_indication_supportive | 10 | 2.89 (1.56-5.38) | 1.41 | Eligible |
| T-DXd | Hyperkalaemia | all_indication_supportive | 10 | 0.65 (0.35-1.20) | 0.39 | Ineligible |
| T-DXd | Hypertransaminasaemia | all_indication_supportive | 10 | 2.73 (1.47-5.09) | 1.35 | Eligible |
| T-DXd | Immune system disorder | all_indication_supportive | 10 | 1.49 (0.80-2.78) | 0.83 | Ineligible |
| T-DXd | Limb discomfort | all_indication_supportive | 10 | 0.59 (0.32-1.10) | 0.36 | Ineligible |
| T-DXd | Mouth ulceration | all_indication_supportive | 10 | 1.02 (0.55-1.89) | 0.59 | Ineligible |
| T-DXd | Osteomyelitis | all_indication_supportive | 10 | 1.24 (0.67-2.30) | 0.70 | Ineligible |
| T-DXd | Parosmia | all_indication_supportive | 10 | 2.99 (1.61-5.56) | 1.45 | Eligible |
| T-DXd | Pseudocirrhosis | all_indication_supportive | 10 | 57.03 (30.35-107.17) | 24.11 | Eligible |
| T-DXd | Rash pruritic | all_indication_supportive | 10 | 0.39 (0.21-0.72) | 0.24 | Ineligible |
| T-DXd | Urinary retention | all_indication_supportive | 10 | 0.64 (0.35-1.20) | 0.39 | Ineligible |
| T-DXd | Adverse reaction | all_indication_supportive | 9 | 1.24 (0.65-2.39) | 0.68 | Ineligible |
| T-DXd | Aphasia | all_indication_supportive | 9 | 0.65 (0.34-1.26) | 0.39 | Ineligible |
| T-DXd | Asthma | all_indication_supportive | 9 | 0.16 (0.08-0.31) | 0.10 | Ineligible |
| T-DXd | Atelectasis | all_indication_supportive | 9 | 2.39 (1.24-4.59) | 1.17 | Ineligible |
| T-DXd | Bacteraemia | all_indication_supportive | 9 | 1.60 (0.83-3.07) | 0.84 | Ineligible |
| T-DXd | Blood calcium decreased | all_indication_supportive | 9 | 1.75 (0.91-3.36) | 0.91 | Ineligible |
| T-DXd | Blood glucose abnormal | all_indication_supportive | 9 | 0.94 (0.49-1.80) | 0.53 | Ineligible |
| T-DXd | Cerebral infarction | all_indication_supportive | 9 | 0.82 (0.43-1.58) | 0.48 | Ineligible |
| T-DXd | Coma | all_indication_supportive | 9 | 0.44 (0.23-0.84) | 0.27 | Ineligible |
| T-DXd | Cystitis | all_indication_supportive | 9 | 0.58 (0.30-1.12) | 0.35 | Ineligible |
| T-DXd | Depressed mood | all_indication_supportive | 9 | 0.34 (0.18-0.66) | 0.21 | Ineligible |
| T-DXd | Enteritis | all_indication_supportive | 9 | 2.78 (1.44-5.34) | 1.31 | Ineligible |
| T-DXd | General physical condition abnormal | all_indication_supportive | 9 | 2.66 (1.38-5.12) | 1.27 | Ineligible |
| T-DXd | Haematochezia | all_indication_supportive | 9 | 0.33 (0.17-0.63) | 0.20 | Ineligible |
| T-DXd | Hepatic encephalopathy | all_indication_supportive | 9 | 2.13 (1.11-4.09) | 1.06 | Ineligible |
| T-DXd | Hypercalcaemia | all_indication_supportive | 9 | 1.58 (0.82-3.03) | 0.84 | Ineligible |
| T-DXd | Immune thrombocytopenia | all_indication_supportive | 9 | 3.59 (1.87-6.90) | 1.59 | Ineligible |
| T-DXd | Immunosuppression | all_indication_supportive | 9 | 2.07 (1.08-3.99) | 1.04 | Ineligible |
| T-DXd | Infusion site extravasation | all_indication_supportive | 9 | 2.73 (1.42-5.25) | 1.29 | Ineligible |
| T-DXd | Intentional dose omission | all_indication_supportive | 9 | 0.60 (0.31-1.16) | 0.36 | Ineligible |
| T-DXd | Intentional product misuse | all_indication_supportive | 9 | 0.18 (0.09-0.34) | 0.11 | Ineligible |
| T-DXd | Intestinal ischaemia | all_indication_supportive | 9 | 3.79 (1.97-7.30) | 1.66 | Ineligible |
| T-DXd | Intra-abdominal fluid collection | all_indication_supportive | 9 | 8.79 (4.56-16.93) | 3.83 | Ineligible |
| T-DXd | KL-6 increased | all_indication_supportive | 9 | 82.93 (42.43-162.06) | 28.89 | Ineligible |
| T-DXd | Lymphopenia | all_indication_supportive | 9 | 1.31 (0.68-2.51) | 0.71 | Ineligible |
| T-DXd | Melaena | all_indication_supportive | 9 | 0.89 (0.46-1.72) | 0.51 | Ineligible |
| T-DXd | Skin hyperpigmentation | all_indication_supportive | 9 | 2.15 (1.12-4.13) | 1.07 | Ineligible |
| T-DXd | Skin lesion | all_indication_supportive | 9 | 0.69 (0.36-1.32) | 0.40 | Ineligible |
| T-DXd | Speech disorder | all_indication_supportive | 9 | 0.38 (0.20-0.73) | 0.23 | Ineligible |
| T-DXd | Transient ischaemic attack | all_indication_supportive | 9 | 0.67 (0.35-1.29) | 0.39 | Ineligible |
| T-DXd | Urosepsis | all_indication_supportive | 9 | 2.06 (1.07-3.97) | 1.04 | Ineligible |
| T-DXd | Viral infection | all_indication_supportive | 9 | 0.56 (0.29-1.08) | 0.34 | Ineligible |
| T-DXd | Arrhythmia | all_indication_supportive | 8 | 0.40 (0.20-0.80) | 0.24 | Ineligible |
| T-DXd | Blister | all_indication_supportive | 8 | 0.29 (0.15-0.58) | 0.18 | Ineligible |
| T-DXd | Bradycardia | all_indication_supportive | 8 | 0.34 (0.17-0.68) | 0.20 | Ineligible |
| T-DXd | Depressed level of consciousness | all_indication_supportive | 8 | 0.49 (0.25-0.98) | 0.29 | Ineligible |
| T-DXd | Drug interaction | all_indication_supportive | 8 | 0.11 (0.06-0.22) | 0.07 | Ineligible |
| T-DXd | Feeling hot | all_indication_supportive | 8 | 0.29 (0.15-0.58) | 0.18 | Ineligible |
| T-DXd | Femoral neck fracture | all_indication_supportive | 8 | 3.36 (1.68-6.72) | 1.43 | Ineligible |
| T-DXd | Food aversion | all_indication_supportive | 8 | 10.43 (5.20-20.90) | 4.46 | Ineligible |
| T-DXd | Hepatic steatosis | all_indication_supportive | 8 | 1.01 (0.50-2.02) | 0.55 | Ineligible |
| T-DXd | Joint swelling | all_indication_supportive | 8 | 0.13 (0.06-0.26) | 0.08 | Ineligible |
| T-DXd | Lung neoplasm | all_indication_supportive | 8 | 6.95 (3.47-13.92) | 2.64 | Ineligible |
| T-DXd | Lung neoplasm malignant | all_indication_supportive | 8 | 0.37 (0.19-0.74) | 0.22 | Ineligible |
| T-DXd | Malignant pleural effusion | all_indication_supportive | 8 | 10.49 (5.23-21.02) | 4.50 | Ineligible |
| T-DXd | Metabolic acidosis | all_indication_supportive | 8 | 0.54 (0.27-1.08) | 0.32 | Ineligible |
| T-DXd | Migraine | all_indication_supportive | 8 | 0.17 (0.08-0.34) | 0.10 | Ineligible |
| T-DXd | Neurotoxicity | all_indication_supportive | 8 | 0.91 (0.46-1.83) | 0.51 | Ineligible |
| T-DXd | Overdose | all_indication_supportive | 8 | 0.07 (0.04-0.14) | 0.05 | Ineligible |
| T-DXd | Pancreatitis | all_indication_supportive | 8 | 0.37 (0.19-0.75) | 0.22 | Ineligible |
| T-DXd | Pathological fracture | all_indication_supportive | 8 | 3.50 (1.75-7.00) | 1.47 | Ineligible |
| T-DXd | Pneumonia cytomegaloviral | all_indication_supportive | 8 | 9.12 (4.55-18.28) | 3.68 | Ineligible |
| T-DXd | Product storage error | all_indication_supportive | 8 | 0.16 (0.08-0.32) | 0.10 | Ineligible |
| T-DXd | Product use complaint | all_indication_supportive | 8 | 0.91 (0.46-1.83) | 0.51 | Ineligible |
| T-DXd | Rectal haemorrhage | all_indication_supportive | 8 | 0.40 (0.20-0.80) | 0.24 | Ineligible |
| T-DXd | Respiratory syncytial virus infection | all_indication_supportive | 8 | 1.82 (0.91-3.64) | 0.90 | Ineligible |
| T-DXd | Respiratory tract infection | all_indication_supportive | 8 | 0.61 (0.30-1.22) | 0.35 | Ineligible |
| T-DXd | SARS-CoV-2 test positive | all_indication_supportive | 8 | 1.00 (0.50-2.00) | 0.55 | Ineligible |
| T-DXd | Skin exfoliation | all_indication_supportive | 8 | 0.18 (0.09-0.37) | 0.11 | Ineligible |
| T-DXd | Swelling face | all_indication_supportive | 8 | 0.27 (0.13-0.54) | 0.16 | Ineligible |
| T-DXd | Ulcer haemorrhage | all_indication_supportive | 8 | 2.47 (1.23-4.94) | 1.14 | Ineligible |
| T-DXd | Unresponsive to stimuli | all_indication_supportive | 8 | 0.71 (0.35-1.41) | 0.40 | Ineligible |
| T-DXd | Vertigo | all_indication_supportive | 8 | 0.27 (0.14-0.55) | 0.17 | Ineligible |
| T-DXd | White blood cell count increased | all_indication_supportive | 8 | 0.48 (0.24-0.96) | 0.28 | Ineligible |
| T-DXd | Abdominal cavity drainage | all_indication_supportive | 7 | 33.77 (15.97-71.39) | 13.79 | Ineligible |
| T-DXd | Bone lesion | all_indication_supportive | 7 | 4.18 (1.99-8.77) | 1.56 | Ineligible |
| T-DXd | Brain injury | all_indication_supportive | 7 | 1.34 (0.64-2.81) | 0.67 | Ineligible |
| T-DXd | Breast haemorrhage | all_indication_supportive | 7 | 43.56 (20.56-92.31) | 16.53 | Ineligible |
| T-DXd | Bronchitis | all_indication_supportive | 7 | 0.19 (0.09-0.40) | 0.12 | Ineligible |
| T-DXd | Burning sensation | all_indication_supportive | 7 | 0.21 (0.10-0.45) | 0.13 | Ineligible |
| T-DXd | Cachexia | all_indication_supportive | 7 | 3.06 (1.46-6.43) | 1.25 | Ineligible |
| T-DXd | Cerebellar infarction | all_indication_supportive | 7 | 9.99 (4.75-21.02) | 3.72 | Ineligible |
| T-DXd | Cerebral disorder | all_indication_supportive | 7 | 1.81 (0.86-3.81) | 0.85 | Ineligible |
| T-DXd | Chronic obstructive pulmonary disease | all_indication_supportive | 7 | 0.28 (0.14-0.60) | 0.17 | Ineligible |
| T-DXd | Contusion | all_indication_supportive | 7 | 0.15 (0.07-0.31) | 0.09 | Ineligible |
| T-DXd | Cystitis haemorrhagic | all_indication_supportive | 7 | 3.65 (1.74-7.66) | 1.42 | Ineligible |
| T-DXd | Disorientation | all_indication_supportive | 7 | 0.43 (0.20-0.90) | 0.25 | Ineligible |
| T-DXd | Emotional distress | all_indication_supportive | 7 | 0.11 (0.05-0.23) | 0.07 | Ineligible |
| T-DXd | Extravasation | all_indication_supportive | 7 | 3.97 (1.89-8.33) | 1.51 | Ineligible |
| T-DXd | Fluid retention | all_indication_supportive | 7 | 0.27 (0.13-0.57) | 0.16 | Ineligible |
| T-DXd | Gastroenteritis | all_indication_supportive | 7 | 1.07 (0.51-2.25) | 0.56 | Ineligible |
| T-DXd | Haematocrit decreased | all_indication_supportive | 7 | 0.93 (0.44-1.96) | 0.50 | Ineligible |
| T-DXd | Hepatic cyst | all_indication_supportive | 7 | 4.72 (2.25-9.92) | 1.72 | Ineligible |
| T-DXd | Hepatomegaly | all_indication_supportive | 7 | 1.82 (0.86-3.81) | 0.85 | Ineligible |
| T-DXd | Ill-defined disorder | all_indication_supportive | 7 | 0.22 (0.11-0.47) | 0.13 | Ineligible |
| T-DXd | Impaired healing | all_indication_supportive | 7 | 0.46 (0.22-0.97) | 0.27 | Ineligible |
| T-DXd | Incorrect product administration duration | all_indication_supportive | 7 | 0.59 (0.28-1.24) | 0.33 | Ineligible |
| T-DXd | Left ventricular dysfunction | all_indication_supportive | 7 | 2.29 (1.09-4.81) | 1.02 | Ineligible |
| T-DXd | Lower limb fracture | all_indication_supportive | 7 | 0.75 (0.36-1.57) | 0.41 | Ineligible |
| T-DXd | Lymphadenopathy | all_indication_supportive | 7 | 0.43 (0.21-0.91) | 0.25 | Ineligible |
| T-DXd | Malnutrition | all_indication_supportive | 7 | 1.41 (0.67-2.97) | 0.70 | Ineligible |
| T-DXd | Mental status changes | all_indication_supportive | 7 | 0.62 (0.30-1.30) | 0.35 | Ineligible |
| T-DXd | Neuralgia | all_indication_supportive | 7 | 0.57 (0.27-1.19) | 0.32 | Ineligible |
| T-DXd | Ocular icterus | all_indication_supportive | 7 | 3.93 (1.87-8.26) | 1.50 | Ineligible |
| T-DXd | Oncologic complication | all_indication_supportive | 7 | 13.65 (6.49-28.73) | 5.88 | Ineligible |
| T-DXd | Ovarian cancer | all_indication_supportive | 7 | 1.59 (0.76-3.34) | 0.77 | Ineligible |
| T-DXd | Product preparation issue | all_indication_supportive | 7 | 2.30 (1.10-4.83) | 1.02 | Ineligible |
| T-DXd | Pyelonephritis | all_indication_supportive | 7 | 1.72 (0.82-3.62) | 0.82 | Ineligible |
| T-DXd | Respiratory symptom | all_indication_supportive | 7 | 4.42 (2.10-9.28) | 1.63 | Ineligible |
| T-DXd | Restlessness | all_indication_supportive | 7 | 0.43 (0.20-0.90) | 0.25 | Ineligible |
| T-DXd | Skin fissures | all_indication_supportive | 7 | 0.71 (0.34-1.48) | 0.39 | Ineligible |
| T-DXd | Skin toxicity | all_indication_supportive | 7 | 2.50 (1.19-5.26) | 1.09 | Ineligible |
| T-DXd | Spinal compression fracture | all_indication_supportive | 7 | 1.70 (0.81-3.57) | 0.81 | Ineligible |
| T-DXd | Terminal state | all_indication_supportive | 7 | 1.97 (0.94-4.14) | 0.91 | Ineligible |
| T-DXd | Weight fluctuation | all_indication_supportive | 7 | 1.27 (0.60-2.66) | 0.64 | Ineligible |
| T-DXd | Acute myocardial infarction | all_indication_supportive | 6 | 0.48 (0.21-1.06) | 0.26 | Ineligible |
| T-DXd | Agitation | all_indication_supportive | 6 | 0.19 (0.09-0.43) | 0.11 | Ineligible |
| T-DXd | Appetite disorder | all_indication_supportive | 6 | 1.80 (0.81-4.01) | 0.80 | Ineligible |
| T-DXd | Blood alkaline phosphatase abnormal | all_indication_supportive | 6 | 13.78 (6.17-30.78) | 5.14 | Ineligible |
| T-DXd | Bone marrow failure | all_indication_supportive | 6 | 0.63 (0.28-1.40) | 0.34 | Ineligible |
| T-DXd | Carcinoembryonic antigen increased | all_indication_supportive | 6 | 6.26 (2.81-13.95) | 1.94 | Ineligible |
| T-DXd | Central nervous system lesion | all_indication_supportive | 6 | 0.85 (0.38-1.90) | 0.44 | Ineligible |
| T-DXd | Deafness | all_indication_supportive | 6 | 0.47 (0.21-1.05) | 0.26 | Ineligible |
| T-DXd | Device occlusion | all_indication_supportive | 6 | 0.78 (0.35-1.74) | 0.41 | Ineligible |
| T-DXd | Diplopia | all_indication_supportive | 6 | 0.52 (0.23-1.16) | 0.29 | Ineligible |
| T-DXd | Drug effect less than expected | all_indication_supportive | 6 | 0.81 (0.36-1.81) | 0.42 | Ineligible |
| T-DXd | Dysstasia | all_indication_supportive | 6 | 0.43 (0.19-0.96) | 0.24 | Ineligible |
| T-DXd | Ear discomfort | all_indication_supportive | 6 | 1.19 (0.53-2.64) | 0.58 | Ineligible |
| T-DXd | Eye haematoma | all_indication_supportive | 6 | 40.76 (18.13-91.66) | 14.43 | Ineligible |
| T-DXd | Faeces discoloured | all_indication_supportive | 6 | 0.61 (0.27-1.36) | 0.33 | Ineligible |
| T-DXd | Fluid intake reduced | all_indication_supportive | 6 | 2.71 (1.22-6.05) | 1.07 | Ineligible |
| T-DXd | Fungal infection | all_indication_supportive | 6 | 0.36 (0.16-0.80) | 0.20 | Ineligible |
| T-DXd | Gastroenteritis listeria | all_indication_supportive | 6 | 489.13 (196.39-1218.23) | 36.98 | Ineligible |
| T-DXd | Glomerular filtration rate decreased | all_indication_supportive | 6 | 0.96 (0.43-2.15) | 0.49 | Ineligible |
| T-DXd | Granulocyte count decreased | all_indication_supportive | 6 | 7.25 (3.25-16.17) | 2.22 | Ineligible |
| T-DXd | Hyperhidrosis | all_indication_supportive | 6 | 0.10 (0.05-0.23) | 0.06 | Ineligible |
| T-DXd | Hypoacusis | all_indication_supportive | 6 | 0.25 (0.11-0.57) | 0.15 | Ineligible |
| T-DXd | Hypokinesia | all_indication_supportive | 6 | 0.84 (0.38-1.87) | 0.43 | Ineligible |
| T-DXd | Hypothyroidism | all_indication_supportive | 6 | 0.40 (0.18-0.88) | 0.22 | Ineligible |
| T-DXd | Inappropriate antidiuretic hormone secretion | all_indication_supportive | 6 | 1.34 (0.60-2.99) | 0.64 | Ineligible |
| T-DXd | Intestinal perforation | all_indication_supportive | 6 | 1.16 (0.52-2.58) | 0.57 | Ineligible |
| T-DXd | Jaundice cholestatic | all_indication_supportive | 6 | 4.15 (1.86-9.26) | 1.42 | Ineligible |
| T-DXd | Klebsiella infection | all_indication_supportive | 6 | 2.54 (1.14-5.65) | 1.02 | Ineligible |
| T-DXd | Liver abscess | all_indication_supportive | 6 | 3.87 (1.74-8.63) | 1.36 | Ineligible |
| T-DXd | Localised oedema | all_indication_supportive | 6 | 2.83 (1.27-6.30) | 1.10 | Ineligible |
| T-DXd | Lung consolidation | all_indication_supportive | 6 | 5.34 (2.39-11.90) | 1.71 | Ineligible |
| T-DXd | Mass | all_indication_supportive | 6 | 0.86 (0.39-1.91) | 0.44 | Ineligible |
| T-DXd | Medication error | all_indication_supportive | 6 | 0.29 (0.13-0.65) | 0.17 | Ineligible |
| T-DXd | Necrotising fasciitis | all_indication_supportive | 6 | 3.63 (1.63-8.10) | 1.30 | Ineligible |
| T-DXd | Neurological symptom | all_indication_supportive | 6 | 2.37 (1.07-5.29) | 0.97 | Ineligible |
| T-DXd | Neutropenic sepsis | all_indication_supportive | 6 | 1.85 (0.83-4.12) | 0.81 | Ineligible |
| T-DXd | Onychoclasis | all_indication_supportive | 6 | 1.66 (0.74-3.69) | 0.75 | Ineligible |
| T-DXd | Peritonitis | all_indication_supportive | 6 | 0.61 (0.27-1.35) | 0.33 | Ineligible |
| T-DXd | Pneumomediastinum | all_indication_supportive | 6 | 7.05 (3.16-15.73) | 2.16 | Ineligible |
| T-DXd | Polyneuropathy | all_indication_supportive | 6 | 1.03 (0.46-2.30) | 0.52 | Ineligible |
| T-DXd | Prescribed overdose | all_indication_supportive | 6 | 0.60 (0.27-1.34) | 0.32 | Ineligible |
| T-DXd | Protein total decreased | all_indication_supportive | 6 | 4.01 (1.80-8.93) | 1.39 | Ineligible |
| T-DXd | Rash erythematous | all_indication_supportive | 6 | 0.28 (0.12-0.61) | 0.16 | Ineligible |
| T-DXd | Rash vesicular | all_indication_supportive | 6 | 2.61 (1.17-5.81) | 1.04 | Ineligible |
| T-DXd | Red cell distribution width increased | all_indication_supportive | 6 | 2.71 (1.22-6.04) | 1.07 | Ineligible |
| T-DXd | Retching | all_indication_supportive | 6 | 0.60 (0.27-1.34) | 0.33 | Ineligible |
| T-DXd | Skin haemorrhage | all_indication_supportive | 6 | 0.80 (0.36-1.79) | 0.42 | Ineligible |
| T-DXd | Staphylococcal bacteraemia | all_indication_supportive | 6 | 3.06 (1.37-6.82) | 1.16 | Ineligible |
| T-DXd | Suicidal ideation | all_indication_supportive | 6 | 0.15 (0.07-0.34) | 0.09 | Ineligible |
| T-DXd | Therapeutic product effect incomplete | all_indication_supportive | 6 | 0.14 (0.07-0.32) | 0.09 | Ineligible |
| T-DXd | Tumour haemorrhage | all_indication_supportive | 6 | 4.36 (1.96-9.72) | 1.47 | Ineligible |
| T-DXd | Wound | all_indication_supportive | 6 | 0.40 (0.18-0.89) | 0.22 | Ineligible |
| T-DXd | Angina pectoris | all_indication_supportive | 5 | 0.43 (0.18-1.03) | 0.23 | Ineligible |
| T-DXd | Ankle fracture | all_indication_supportive | 5 | 0.68 (0.28-1.63) | 0.34 | Ineligible |
| T-DXd | Anticoagulation drug level below therapeutic | all_indication_supportive | 5 | 12.44 (5.16-30.00) | 3.50 | Ineligible |
| T-DXd | Arteriosclerosis coronary artery | all_indication_supportive | 5 | 1.94 (0.81-4.68) | 0.78 | Ineligible |
| T-DXd | Asphyxia | all_indication_supportive | 5 | 1.22 (0.51-2.94) | 0.55 | Ineligible |
| T-DXd | Aspiration | all_indication_supportive | 5 | 1.08 (0.45-2.59) | 0.50 | Ineligible |
| T-DXd | Blindness unilateral | all_indication_supportive | 5 | 0.80 (0.33-1.91) | 0.39 | Ineligible |
| T-DXd | Cardio-respiratory arrest | all_indication_supportive | 5 | 0.30 (0.12-0.71) | 0.16 | Ineligible |
| T-DXd | Cholelithiasis | all_indication_supportive | 5 | 0.39 (0.16-0.94) | 0.21 | Ineligible |
| T-DXd | Complication associated with device | all_indication_supportive | 5 | 0.65 (0.27-1.55) | 0.33 | Ineligible |
| T-DXd | Conjunctivitis | all_indication_supportive | 5 | 0.54 (0.23-1.30) | 0.28 | Ineligible |
| T-DXd | Crepitations | all_indication_supportive | 5 | 3.85 (1.60-9.26) | 1.21 | Ineligible |
| T-DXd | Device defective | all_indication_supportive | 5 | 0.59 (0.25-1.43) | 0.31 | Ineligible |
| T-DXd | Diverticulitis | all_indication_supportive | 5 | 0.37 (0.15-0.89) | 0.20 | Ineligible |
| T-DXd | Electrocardiogram QT prolonged | all_indication_supportive | 5 | 0.29 (0.12-0.69) | 0.16 | Ineligible |
| T-DXd | Encephalopathy | all_indication_supportive | 5 | 0.45 (0.19-1.09) | 0.24 | Ineligible |
| T-DXd | Eructation | all_indication_supportive | 5 | 0.60 (0.25-1.44) | 0.31 | Ineligible |
| T-DXd | Gastric haemorrhage | all_indication_supportive | 5 | 0.97 (0.41-2.34) | 0.46 | Ineligible |
| T-DXd | Gastric perforation | all_indication_supportive | 5 | 3.87 (1.61-9.31) | 1.22 | Ineligible |
| T-DXd | Generalised tonic-clonic seizure | all_indication_supportive | 5 | 0.53 (0.22-1.27) | 0.28 | Ineligible |
| T-DXd | Haematuria | all_indication_supportive | 5 | 0.31 (0.13-0.75) | 0.17 | Ineligible |
| T-DXd | Haemorrhoids | all_indication_supportive | 5 | 0.56 (0.23-1.34) | 0.29 | Ineligible |
| T-DXd | Hemiparesis | all_indication_supportive | 5 | 0.68 (0.28-1.63) | 0.34 | Ineligible |
| T-DXd | Hot flush | all_indication_supportive | 5 | 0.13 (0.05-0.32) | 0.08 | Ineligible |
| T-DXd | Hunger | all_indication_supportive | 5 | 0.83 (0.34-1.99) | 0.41 | Ineligible |
| T-DXd | Hydronephrosis | all_indication_supportive | 5 | 1.49 (0.62-3.58) | 0.64 | Ineligible |
| T-DXd | Hyperammonaemia | all_indication_supportive | 5 | 2.17 (0.90-5.22) | 0.84 | Ineligible |
| T-DXd | Hypervolaemia | all_indication_supportive | 5 | 1.96 (0.82-4.71) | 0.78 | Ineligible |
| T-DXd | Hypovolaemia | all_indication_supportive | 5 | 2.19 (0.91-5.26) | 0.84 | Ineligible |
| T-DXd | Impaired gastric emptying | all_indication_supportive | 5 | 0.85 (0.35-2.03) | 0.41 | Ineligible |
| T-DXd | Influenza like illness | all_indication_supportive | 5 | 0.13 (0.06-0.32) | 0.08 | Ineligible |
| T-DXd | Intracranial pressure increased | all_indication_supportive | 5 | 1.99 (0.83-4.78) | 0.79 | Ineligible |
| T-DXd | Leukocytosis | all_indication_supportive | 5 | 0.64 (0.27-1.54) | 0.33 | Ineligible |
| T-DXd | Maculopathy | all_indication_supportive | 5 | 2.81 (1.17-6.76) | 0.99 | Ineligible |
| T-DXd | Mitral valve incompetence | all_indication_supportive | 5 | 1.24 (0.52-2.99) | 0.56 | Ineligible |
| T-DXd | Mitral valve replacement | all_indication_supportive | 5 | 32.35 (13.34-78.41) | 10.89 | Ineligible |
| T-DXd | Nasal obstruction | all_indication_supportive | 5 | 5.05 (2.10-12.14) | 1.45 | Ineligible |
| T-DXd | Near death experience | all_indication_supportive | 5 | 2.67 (1.11-6.43) | 0.96 | Ineligible |
| T-DXd | Nephrolithiasis | all_indication_supportive | 5 | 0.22 (0.09-0.54) | 0.13 | Ineligible |
| T-DXd | Nervousness | all_indication_supportive | 5 | 0.22 (0.09-0.53) | 0.12 | Ineligible |
| T-DXd | Oesophageal varices haemorrhage | all_indication_supportive | 5 | 4.93 (2.05-11.86) | 1.42 | Ineligible |
| T-DXd | Pneumonia fungal | all_indication_supportive | 5 | 2.62 (1.09-6.31) | 0.95 | Ineligible |
| T-DXd | Pneumonitis chemical | all_indication_supportive | 5 | 28.50 (11.77-69.01) | 9.88 | Ineligible |
| T-DXd | Product prescribing issue | all_indication_supportive | 5 | 0.48 (0.20-1.16) | 0.25 | Ineligible |
| T-DXd | Pulmonary alveolar haemorrhage | all_indication_supportive | 5 | 1.84 (0.77-4.43) | 0.75 | Ineligible |
| T-DXd | Pulmonary arterial hypertension | all_indication_supportive | 5 | 0.64 (0.27-1.54) | 0.33 | Ineligible |
| T-DXd | Refusal of treatment by patient | all_indication_supportive | 5 | 2.33 (0.97-5.60) | 0.88 | Ineligible |
| T-DXd | Renal disorder | all_indication_supportive | 5 | 0.22 (0.09-0.53) | 0.12 | Ineligible |
| T-DXd | Right ventricular failure | all_indication_supportive | 5 | 1.50 (0.62-3.61) | 0.65 | Ineligible |
| T-DXd | Scab | all_indication_supportive | 5 | 0.88 (0.37-2.11) | 0.43 | Ineligible |
| T-DXd | Sinus tachycardia | all_indication_supportive | 5 | 0.86 (0.36-2.07) | 0.42 | Ineligible |
| T-DXd | Small intestinal obstruction | all_indication_supportive | 5 | 0.88 (0.37-2.12) | 0.43 | Ineligible |
| T-DXd | Tumour marker decreased | all_indication_supportive | 5 | 49.70 (20.41-121.03) | 14.47 | Ineligible |
| T-DXd | Vascular device infection | all_indication_supportive | 5 | 1.94 (0.81-4.67) | 0.78 | Ineligible |
| T-DXd | Accidental exposure to product | all_indication_supportive | 4 | 0.07 (0.03-0.19) | 0.04 | Ineligible |
| T-DXd | Adrenal insufficiency | all_indication_supportive | 4 | 0.65 (0.25-1.75) | 0.31 | Ineligible |
| T-DXd | Aortic arteriosclerosis | all_indication_supportive | 4 | 3.56 (1.34-9.50) | 1.01 | Ineligible |
| T-DXd | Aphthous ulcer | all_indication_supportive | 4 | 0.90 (0.34-2.40) | 0.40 | Ineligible |
| T-DXd | Autoimmune haemolytic anaemia | all_indication_supportive | 4 | 2.05 (0.77-5.47) | 0.72 | Ineligible |
| T-DXd | Bartholin's cyst removal | all_indication_supportive | 4 | 1630.11 (407.62-6518.92) | 25.06 | Ineligible |
| T-DXd | Blood chloride increased | all_indication_supportive | 4 | 6.13 (2.30-16.36) | 1.40 | Ineligible |
| T-DXd | Breast discomfort | all_indication_supportive | 4 | 8.69 (3.25-23.23) | 1.82 | Ineligible |
| T-DXd | Candida infection | all_indication_supportive | 4 | 0.42 (0.16-1.11) | 0.21 | Ineligible |
| T-DXd | Carbohydrate antigen 125 increased | all_indication_supportive | 4 | 2.05 (0.77-5.46) | 0.72 | Ineligible |
| T-DXd | Cardiac failure acute | all_indication_supportive | 4 | 1.22 (0.46-3.25) | 0.51 | Ineligible |
| T-DXd | Cardiac failure chronic | all_indication_supportive | 4 | 1.75 (0.66-4.66) | 0.65 | Ineligible |
| T-DXd | Cardiac ventricular thrombosis | all_indication_supportive | 4 | 5.79 (2.17-15.46) | 1.34 | Ineligible |
| T-DXd | Cholecystitis | all_indication_supportive | 4 | 0.82 (0.31-2.19) | 0.38 | Ineligible |
| T-DXd | Colitis ulcerative | all_indication_supportive | 4 | 0.17 (0.06-0.46) | 0.09 | Ineligible |
| T-DXd | Colorectal cancer | all_indication_supportive | 4 | 0.14 (0.05-0.37) | 0.08 | Ineligible |
| T-DXd | Coronavirus pneumonia | all_indication_supportive | 4 | 45.28 (16.76-122.31) | 11.41 | Ineligible |
| T-DXd | Creatinine renal clearance increased | all_indication_supportive | 4 | 12.19 (4.56-32.60) | 2.54 | Ineligible |
| T-DXd | Crying | all_indication_supportive | 4 | 0.26 (0.10-0.68) | 0.14 | Ineligible |
| T-DXd | Dental caries | all_indication_supportive | 4 | 0.82 (0.31-2.18) | 0.37 | Ineligible |
| T-DXd | Dermatitis | all_indication_supportive | 4 | 0.39 (0.15-1.03) | 0.20 | Ineligible |
| T-DXd | Dermatitis bullous | all_indication_supportive | 4 | 1.26 (0.47-3.36) | 0.52 | Ineligible |
| T-DXd | Disturbance in attention | all_indication_supportive | 4 | 0.16 (0.06-0.43) | 0.09 | Ineligible |
| T-DXd | Dyskinesia | all_indication_supportive | 4 | 0.24 (0.09-0.63) | 0.13 | Ineligible |
| T-DXd | Dysuria | all_indication_supportive | 4 | 0.24 (0.09-0.63) | 0.13 | Ineligible |
| T-DXd | Echocardiogram abnormal | all_indication_supportive | 4 | 7.74 (2.90-20.68) | 1.65 | Ineligible |
| T-DXd | Eczema | all_indication_supportive | 4 | 0.16 (0.06-0.42) | 0.09 | Ineligible |
| T-DXd | Ejection fraction abnormal | all_indication_supportive | 4 | 6.09 (2.28-16.27) | 1.39 | Ineligible |
| T-DXd | Embolism venous | all_indication_supportive | 4 | 2.55 (0.96-6.80) | 0.83 | Ineligible |
| T-DXd | Emergency care | all_indication_supportive | 4 | 2.38 (0.89-6.35) | 0.79 | Ineligible |
| T-DXd | Encephalitis | all_indication_supportive | 4 | 1.25 (0.47-3.33) | 0.52 | Ineligible |
| T-DXd | Escherichia infection | all_indication_supportive | 4 | 1.07 (0.40-2.86) | 0.46 | Ineligible |
| T-DXd | Eyelid ptosis | all_indication_supportive | 4 | 0.86 (0.32-2.29) | 0.39 | Ineligible |
| T-DXd | Face oedema | all_indication_supportive | 4 | 0.56 (0.21-1.51) | 0.28 | Ineligible |
| T-DXd | Facial paralysis | all_indication_supportive | 4 | 0.82 (0.31-2.18) | 0.37 | Ineligible |
| T-DXd | Febrile bone marrow aplasia | all_indication_supportive | 4 | 1.93 (0.72-5.14) | 0.70 | Ineligible |
| T-DXd | Fluid replacement | all_indication_supportive | 4 | 23.37 (8.71-62.72) | 6.40 | Ineligible |
| T-DXd | Focal nodular hyperplasia | all_indication_supportive | 4 | 19.18 (7.15-51.40) | 4.77 | Ineligible |
| T-DXd | Frustration tolerance decreased | all_indication_supportive | 4 | 0.86 (0.32-2.29) | 0.39 | Ineligible |
| T-DXd | Gastrointestinal infection | all_indication_supportive | 4 | 0.89 (0.34-2.38) | 0.40 | Ineligible |
| T-DXd | Gastrointestinal oedema | all_indication_supportive | 4 | 4.77 (1.79-12.72) | 1.19 | Ineligible |
| T-DXd | Granulocytopenia | all_indication_supportive | 4 | 1.90 (0.71-5.06) | 0.69 | Ineligible |
| T-DXd | Heart rate decreased | all_indication_supportive | 4 | 0.23 (0.09-0.62) | 0.13 | Ineligible |
| T-DXd | Hemiplegia | all_indication_supportive | 4 | 1.10 (0.41-2.95) | 0.47 | Ineligible |
| T-DXd | Hepatic pain | all_indication_supportive | 4 | 1.94 (0.73-5.18) | 0.70 | Ineligible |
| T-DXd | Hydrocephalus | all_indication_supportive | 4 | 1.68 (0.63-4.48) | 0.64 | Ineligible |
| T-DXd | Hyperchlorhydria | all_indication_supportive | 4 | 1.28 (0.48-3.40) | 0.52 | Ineligible |
| T-DXd | Hyperpyrexia | all_indication_supportive | 4 | 1.95 (0.73-5.20) | 0.70 | Ineligible |
| T-DXd | Impaired work ability | all_indication_supportive | 4 | 0.42 (0.16-1.13) | 0.22 | Ineligible |
| T-DXd | Incontinence | all_indication_supportive | 4 | 0.84 (0.31-2.23) | 0.38 | Ineligible |
| T-DXd | Incorrect drug administration rate | all_indication_supportive | 4 | 1.87 (0.70-4.98) | 0.68 | Ineligible |
| T-DXd | Interstitial lung abnormality | all_indication_supportive | 4 | 83.59 (30.60-228.37) | 15.55 | Ineligible |
| T-DXd | Leukoencephalopathy | all_indication_supportive | 4 | 2.56 (0.96-6.83) | 0.83 | Ineligible |
| T-DXd | Lip swelling | all_indication_supportive | 4 | 0.25 (0.10-0.68) | 0.14 | Ineligible |
| T-DXd | Madarosis | all_indication_supportive | 4 | 0.62 (0.23-1.64) | 0.30 | Ineligible |
| T-DXd | Malignant ascites | all_indication_supportive | 4 | 11.62 (4.35-31.08) | 2.41 | Ineligible |
| T-DXd | Metastases to bone marrow | all_indication_supportive | 4 | 17.16 (6.41-45.96) | 4.00 | Ineligible |
| T-DXd | Metastases to pleura | all_indication_supportive | 4 | 7.77 (2.91-20.76) | 1.66 | Ineligible |
| T-DXd | Musculoskeletal chest pain | all_indication_supportive | 4 | 0.54 (0.20-1.45) | 0.27 | Ineligible |
| T-DXd | Musculoskeletal stiffness | all_indication_supportive | 4 | 0.09 (0.03-0.24) | 0.05 | Ineligible |
| T-DXd | Myelodysplastic syndrome | all_indication_supportive | 4 | 0.63 (0.23-1.67) | 0.30 | Ineligible |
| T-DXd | Nail disorder | all_indication_supportive | 4 | 0.93 (0.35-2.48) | 0.41 | Ineligible |
| T-DXd | Nasal congestion | all_indication_supportive | 4 | 0.13 (0.05-0.36) | 0.07 | Ineligible |
| T-DXd | Neck pain | all_indication_supportive | 4 | 0.15 (0.06-0.40) | 0.08 | Ineligible |
| T-DXd | Necrosis | all_indication_supportive | 4 | 1.78 (0.67-4.75) | 0.66 | Ineligible |
| T-DXd | Nodular regenerative hyperplasia | all_indication_supportive | 4 | 12.64 (4.72-33.80) | 2.65 | Ineligible |
| T-DXd | Oesophageal ulcer | all_indication_supportive | 4 | 2.58 (0.97-6.88) | 0.83 | Ineligible |
| T-DXd | Opportunistic infection | all_indication_supportive | 4 | 4.89 (1.83-13.05) | 1.21 | Ineligible |
| T-DXd | Oral candidiasis | all_indication_supportive | 4 | 0.66 (0.25-1.77) | 0.32 | Ineligible |
| T-DXd | Oral herpes | all_indication_supportive | 4 | 0.40 (0.15-1.06) | 0.20 | Ineligible |
| T-DXd | Oral pain | all_indication_supportive | 4 | 0.37 (0.14-0.98) | 0.19 | Ineligible |
| T-DXd | Pain in jaw | all_indication_supportive | 4 | 0.29 (0.11-0.78) | 0.15 | Ineligible |
| T-DXd | Palliative care | all_indication_supportive | 4 | 4.73 (1.77-12.63) | 1.19 | Ineligible |
| T-DXd | Pancreatitis acute | all_indication_supportive | 4 | 0.41 (0.15-1.09) | 0.21 | Ineligible |
| T-DXd | Paralysis | all_indication_supportive | 4 | 0.63 (0.24-1.67) | 0.30 | Ineligible |
| T-DXd | Paranoia | all_indication_supportive | 4 | 0.58 (0.22-1.54) | 0.28 | Ineligible |
| T-DXd | Paronychia | all_indication_supportive | 4 | 1.87 (0.70-4.99) | 0.68 | Ineligible |
| T-DXd | Performance status decreased | all_indication_supportive | 4 | 1.99 (0.75-5.31) | 0.71 | Ineligible |
| T-DXd | Photophobia | all_indication_supportive | 4 | 0.45 (0.17-1.19) | 0.23 | Ineligible |
| T-DXd | Presyncope | all_indication_supportive | 4 | 0.34 (0.13-0.91) | 0.18 | Ineligible |
| T-DXd | Pseudomonas infection | all_indication_supportive | 4 | 1.05 (0.39-2.79) | 0.45 | Ineligible |
| T-DXd | Radiation injury | all_indication_supportive | 4 | 9.24 (3.46-24.68) | 1.92 | Ineligible |
| T-DXd | Rales | all_indication_supportive | 4 | 1.56 (0.58-4.16) | 0.60 | Ineligible |
| T-DXd | Salivary hypersecretion | all_indication_supportive | 4 | 0.88 (0.33-2.33) | 0.40 | Ineligible |
| T-DXd | Scar | all_indication_supportive | 4 | 0.52 (0.19-1.38) | 0.26 | Ineligible |
| T-DXd | Shock haemorrhagic | all_indication_supportive | 4 | 0.99 (0.37-2.65) | 0.44 | Ineligible |
| T-DXd | Skin abrasion | all_indication_supportive | 4 | 1.48 (0.55-3.94) | 0.58 | Ineligible |
| T-DXd | Spinal fracture | all_indication_supportive | 4 | 0.40 (0.15-1.08) | 0.21 | Ineligible |
| T-DXd | Starvation | all_indication_supportive | 4 | 10.80 (4.04-28.86) | 2.23 | Ineligible |
| T-DXd | Stress | all_indication_supportive | 4 | 0.11 (0.04-0.30) | 0.06 | Ineligible |
| T-DXd | Surgery | all_indication_supportive | 4 | 0.14 (0.05-0.37) | 0.08 | Ineligible |
| T-DXd | Symptom recurrence | all_indication_supportive | 4 | 1.21 (0.45-3.22) | 0.50 | Ineligible |
| T-DXd | Tachypnoea | all_indication_supportive | 4 | 0.65 (0.24-1.73) | 0.31 | Ineligible |
| T-DXd | Therapy non-responder | all_indication_supportive | 4 | 0.17 (0.06-0.44) | 0.09 | Ineligible |
| T-DXd | Tinnitus | all_indication_supportive | 4 | 0.19 (0.07-0.50) | 0.10 | Ineligible |
| T-DXd | Tooth disorder | all_indication_supportive | 4 | 0.36 (0.14-0.97) | 0.19 | Ineligible |
| T-DXd | Toxic skin eruption | all_indication_supportive | 4 | 0.91 (0.34-2.43) | 0.41 | Ineligible |
| T-DXd | Traumatic lung injury | all_indication_supportive | 4 | 4.21 (1.58-11.25) | 1.11 | Ineligible |
| T-DXd | Treatment delayed | all_indication_supportive | 4 | 4.90 (1.84-13.07) | 1.21 | Ineligible |
| T-DXd | Tumour marker abnormal | all_indication_supportive | 4 | 20.38 (7.60-54.63) | 5.25 | Ineligible |
| T-DXd | Type 2 diabetes mellitus | all_indication_supportive | 4 | 0.24 (0.09-0.65) | 0.13 | Ineligible |
| T-DXd | Ulcerative keratitis | all_indication_supportive | 4 | 3.17 (1.19-8.45) | 0.94 | Ineligible |
| T-DXd | Urinary tract stoma complication | all_indication_supportive | 4 | 80.50 (29.49-219.72) | 15.31 | Ineligible |
| T-DXd | Varices oesophageal | all_indication_supportive | 4 | 2.97 (1.11-7.92) | 0.91 | Ineligible |
| T-DXd | Varicose vein | all_indication_supportive | 4 | 1.61 (0.60-4.28) | 0.62 | Ineligible |
| T-DXd | Ventricular dysfunction | all_indication_supportive | 4 | 5.22 (1.96-13.94) | 1.26 | Ineligible |
| T-DXd | Vocal cord paralysis | all_indication_supportive | 4 | 5.05 (1.89-13.47) | 1.23 | Ineligible |
| T-DXd | White blood cell count abnormal | all_indication_supportive | 4 | 1.19 (0.45-3.18) | 0.50 | Ineligible |
| T-DXd | Withdrawal syndrome | all_indication_supportive | 4 | 0.19 (0.07-0.50) | 0.10 | Ineligible |
| T-DXd | Abdominal tenderness | all_indication_supportive | 3 | 1.42 (0.46-4.39) | 0.50 | Ineligible |
| T-DXd | Acute interstitial pneumonitis | all_indication_supportive | 3 | 10.87 (3.49-33.82) | 1.60 | Ineligible |
| T-DXd | Administration site bruise | all_indication_supportive | 3 | 15.72 (5.04-49.02) | 2.20 | Ineligible |
| T-DXd | Administration site extravasation | all_indication_supportive | 3 | 5.39 (1.74-16.75) | 1.03 | Ineligible |
| T-DXd | Affective disorder | all_indication_supportive | 3 | 0.80 (0.26-2.49) | 0.34 | Ineligible |
| T-DXd | Akathisia | all_indication_supportive | 3 | 0.54 (0.17-1.68) | 0.25 | Ineligible |
| T-DXd | Anaphylactic reaction | all_indication_supportive | 3 | 0.12 (0.04-0.36) | 0.06 | Ineligible |
| T-DXd | Anaphylactic shock | all_indication_supportive | 3 | 0.26 (0.09-0.82) | 0.13 | Ineligible |
| T-DXd | Anger | all_indication_supportive | 3 | 0.21 (0.07-0.64) | 0.11 | Ineligible |
| T-DXd | Aplasia | all_indication_supportive | 3 | 3.39 (1.09-10.53) | 0.81 | Ineligible |
| T-DXd | Arthritis bacterial | all_indication_supportive | 3 | 1.58 (0.51-4.91) | 0.53 | Ineligible |
| T-DXd | Arthropathy | all_indication_supportive | 3 | 0.10 (0.03-0.32) | 0.06 | Ineligible |
| T-DXd | Aspergillus infection | all_indication_supportive | 3 | 0.80 (0.26-2.47) | 0.33 | Ineligible |
| T-DXd | Biliary obstruction | all_indication_supportive | 3 | 4.47 (1.44-13.89) | 0.93 | Ineligible |
| T-DXd | Blood bicarbonate decreased | all_indication_supportive | 3 | 4.88 (1.57-15.16) | 0.98 | Ineligible |
| T-DXd | Blood disorder | all_indication_supportive | 3 | 0.81 (0.26-2.50) | 0.34 | Ineligible |
| T-DXd | Blood glucose decreased | all_indication_supportive | 3 | 0.15 (0.05-0.45) | 0.08 | Ineligible |
| T-DXd | Blood lactic acid abnormal | all_indication_supportive | 3 | 53.73 (17.01-169.75) | 8.87 | Ineligible |
| T-DXd | Blood lactic acid increased | all_indication_supportive | 3 | 1.21 (0.39-3.77) | 0.45 | Ineligible |
| T-DXd | Bone disorder | all_indication_supportive | 3 | 0.39 (0.13-1.22) | 0.19 | Ineligible |
| T-DXd | Breast mass | all_indication_supportive | 3 | 1.02 (0.33-3.16) | 0.40 | Ineligible |
| T-DXd | Bronchiectasis | all_indication_supportive | 3 | 0.89 (0.29-2.77) | 0.36 | Ineligible |
| T-DXd | Bronchiolitis | all_indication_supportive | 3 | 1.86 (0.60-5.76) | 0.59 | Ineligible |
| T-DXd | Bronchopulmonary aspergillosis | all_indication_supportive | 3 | 0.80 (0.26-2.48) | 0.33 | Ineligible |
| T-DXd | Cardiogenic shock | all_indication_supportive | 3 | 0.45 (0.15-1.40) | 0.21 | Ineligible |
| T-DXd | Cardiomegaly | all_indication_supportive | 3 | 0.69 (0.22-2.15) | 0.30 | Ineligible |
| T-DXd | Cardiovascular disorder | all_indication_supportive | 3 | 0.36 (0.12-1.12) | 0.17 | Ineligible |
| T-DXd | Catheter site infection | all_indication_supportive | 3 | 1.59 (0.51-4.94) | 0.54 | Ineligible |
| T-DXd | Cell marker increased | all_indication_supportive | 3 | 13.32 (4.28-41.51) | 1.89 | Ineligible |
| T-DXd | Choking | all_indication_supportive | 3 | 0.32 (0.10-0.98) | 0.16 | Ineligible |
| T-DXd | Chronic kidney disease | all_indication_supportive | 3 | 0.07 (0.02-0.20) | 0.04 | Ineligible |
| T-DXd | Colitis ischaemic | all_indication_supportive | 3 | 1.07 (0.34-3.32) | 0.41 | Ineligible |
| T-DXd | Colorectal cancer stage IV | all_indication_supportive | 3 | 1.06 (0.34-3.29) | 0.41 | Ineligible |
| T-DXd | Corneal disorder | all_indication_supportive | 3 | 3.10 (1.00-9.63) | 0.78 | Ineligible |
| T-DXd | Cyst | all_indication_supportive | 3 | 0.50 (0.16-1.54) | 0.23 | Ineligible |
| T-DXd | Device malfunction | all_indication_supportive | 3 | 0.09 (0.03-0.27) | 0.05 | Ineligible |
| T-DXd | Diabetes mellitus inadequate control | all_indication_supportive | 3 | 0.39 (0.12-1.20) | 0.19 | Ineligible |
| T-DXd | Diabetic metabolic decompensation | all_indication_supportive | 3 | 3.70 (1.19-11.50) | 0.85 | Ineligible |
| T-DXd | Diastolic dysfunction | all_indication_supportive | 3 | 2.27 (0.73-7.05) | 0.66 | Ineligible |
| T-DXd | Dilated cardiomyopathy | all_indication_supportive | 3 | 5.81 (1.87-18.07) | 1.07 | Ineligible |
| T-DXd | Diverticular perforation | all_indication_supportive | 3 | 3.40 (1.10-10.56) | 0.81 | Ineligible |
| T-DXd | Duodenal ulcer | all_indication_supportive | 3 | 1.01 (0.33-3.15) | 0.40 | Ineligible |
| T-DXd | Ecchymosis | all_indication_supportive | 3 | 1.05 (0.34-3.25) | 0.41 | Ineligible |
| T-DXd | Embolism | all_indication_supportive | 3 | 0.82 (0.27-2.56) | 0.34 | Ineligible |
| T-DXd | Emotional disorder | all_indication_supportive | 3 | 0.21 (0.07-0.65) | 0.11 | Ineligible |
| T-DXd | Endocarditis | all_indication_supportive | 3 | 1.28 (0.41-3.96) | 0.47 | Ineligible |
| T-DXd | Enterococcal infection | all_indication_supportive | 3 | 1.42 (0.46-4.42) | 0.50 | Ineligible |
| T-DXd | Erysipelas | all_indication_supportive | 3 | 1.23 (0.40-3.82) | 0.45 | Ineligible |
| T-DXd | Escherichia sepsis | all_indication_supportive | 3 | 2.37 (0.76-7.34) | 0.67 | Ineligible |
| T-DXd | Euphoric mood | all_indication_supportive | 3 | 0.66 (0.21-2.05) | 0.29 | Ineligible |
| T-DXd | Eye disorder | all_indication_supportive | 3 | 0.19 (0.06-0.58) | 0.10 | Ineligible |
| T-DXd | Eye haemorrhage | all_indication_supportive | 3 | 0.49 (0.16-1.52) | 0.23 | Ineligible |
| T-DXd | Facial discomfort | all_indication_supportive | 3 | 8.62 (2.77-26.82) | 1.35 | Ineligible |
| T-DXd | Faeces soft | all_indication_supportive | 3 | 0.72 (0.23-2.23) | 0.31 | Ineligible |
| T-DXd | Feeling cold | all_indication_supportive | 3 | 0.23 (0.07-0.71) | 0.12 | Ineligible |
| T-DXd | Feeling jittery | all_indication_supportive | 3 | 0.37 (0.12-1.14) | 0.18 | Ineligible |
| T-DXd | Fluid imbalance | all_indication_supportive | 3 | 8.39 (2.70-26.09) | 1.33 | Ineligible |
| T-DXd | Food craving | all_indication_supportive | 3 | 1.47 (0.47-4.57) | 0.51 | Ineligible |
| T-DXd | Furuncle | all_indication_supportive | 3 | 0.78 (0.25-2.42) | 0.33 | Ineligible |
| T-DXd | Gastric dilatation | all_indication_supportive | 3 | 3.58 (1.15-11.11) | 0.83 | Ineligible |
| T-DXd | Gastric varices | all_indication_supportive | 3 | 14.01 (4.50-43.66) | 1.97 | Ineligible |
| T-DXd | Gingival erythema | all_indication_supportive | 3 | 7.91 (2.54-24.60) | 1.28 | Ineligible |
| T-DXd | Haemoglobin abnormal | all_indication_supportive | 3 | 0.90 (0.29-2.80) | 0.37 | Ineligible |
| T-DXd | Haemolysis | all_indication_supportive | 3 | 0.85 (0.27-2.64) | 0.35 | Ineligible |
| T-DXd | Haemorrhoidal haemorrhage | all_indication_supportive | 3 | 1.26 (0.41-3.90) | 0.46 | Ineligible |
| T-DXd | Hallucination | all_indication_supportive | 3 | 0.09 (0.03-0.27) | 0.05 | Ineligible |
| T-DXd | Head and neck cancer | all_indication_supportive | 3 | 9.35 (3.01-29.09) | 1.43 | Ineligible |
| T-DXd | Head discomfort | all_indication_supportive | 3 | 0.31 (0.10-0.97) | 0.15 | Ineligible |
| T-DXd | Heart failure with reduced ejection fraction | all_indication_supportive | 3 | 14.38 (4.61-44.82) | 2.02 | Ineligible |
| T-DXd | Heart rate irregular | all_indication_supportive | 3 | 0.31 (0.10-0.96) | 0.15 | Ineligible |
| T-DXd | Hemianopia | all_indication_supportive | 3 | 7.51 (2.42-23.35) | 1.24 | Ineligible |
| T-DXd | Hepatic fibrosis | all_indication_supportive | 3 | 2.11 (0.68-6.54) | 0.63 | Ineligible |
| T-DXd | Hepatocellular injury | all_indication_supportive | 3 | 0.39 (0.12-1.20) | 0.19 | Ineligible |
| T-DXd | Hiccups | all_indication_supportive | 3 | 0.83 (0.27-2.57) | 0.34 | Ineligible |
| T-DXd | Hip arthroplasty | all_indication_supportive | 3 | 0.40 (0.13-1.25) | 0.19 | Ineligible |
| T-DXd | Hypersensitivity pneumonitis | all_indication_supportive | 3 | 4.76 (1.53-14.77) | 0.96 | Ineligible |
| T-DXd | Hyperthyroidism | all_indication_supportive | 3 | 0.43 (0.14-1.35) | 0.21 | Ineligible |
| T-DXd | Hypovolaemic shock | all_indication_supportive | 3 | 1.34 (0.43-4.14) | 0.48 | Ineligible |
| T-DXd | Ileus paralytic | all_indication_supportive | 3 | 1.70 (0.55-5.29) | 0.56 | Ineligible |
| T-DXd | Impaired driving ability | all_indication_supportive | 3 | 0.64 (0.21-1.99) | 0.28 | Ineligible |
| T-DXd | Incorrect dosage administered | all_indication_supportive | 3 | 1.04 (0.34-3.23) | 0.41 | Ineligible |
| T-DXd | Incorrect route of product administration | all_indication_supportive | 3 | 0.31 (0.10-0.97) | 0.15 | Ineligible |
| T-DXd | Insurance issue | all_indication_supportive | 3 | 0.38 (0.12-1.18) | 0.18 | Ineligible |
| T-DXd | Intentional underdose | all_indication_supportive | 3 | 0.71 (0.23-2.21) | 0.31 | Ineligible |
| T-DXd | Internal haemorrhage | all_indication_supportive | 3 | 0.44 (0.14-1.38) | 0.21 | Ineligible |
| T-DXd | Intracardiac thrombus | all_indication_supportive | 3 | 2.41 (0.78-7.49) | 0.68 | Ineligible |
| T-DXd | Jugular vein thrombosis | all_indication_supportive | 3 | 3.91 (1.26-12.15) | 0.87 | Ineligible |
| T-DXd | Klebsiella test positive | all_indication_supportive | 3 | 9.53 (3.06-29.66) | 1.45 | Ineligible |
| T-DXd | Lacrimation increased | all_indication_supportive | 3 | 0.20 (0.06-0.62) | 0.10 | Ineligible |
| T-DXd | Language disorder | all_indication_supportive | 3 | 2.39 (0.77-7.42) | 0.68 | Ineligible |
| T-DXd | Large intestine perforation | all_indication_supportive | 3 | 0.96 (0.31-2.97) | 0.38 | Ineligible |
| T-DXd | Limbal stem cell deficiency | all_indication_supportive | 3 | 67.91 (21.39-215.58) | 10.17 | Ineligible |
| T-DXd | Mental fatigue | all_indication_supportive | 3 | 3.60 (1.16-11.18) | 0.84 | Ineligible |
| T-DXd | Metastatic neoplasm | all_indication_supportive | 3 | 1.58 (0.51-4.91) | 0.53 | Ineligible |
| T-DXd | Monoparesis | all_indication_supportive | 3 | 3.68 (1.19-11.44) | 0.85 | Ineligible |
| T-DXd | Motor dysfunction | all_indication_supportive | 3 | 0.64 (0.21-2.00) | 0.28 | Ineligible |
| T-DXd | Movement disorder | all_indication_supportive | 3 | 0.22 (0.07-0.67) | 0.11 | Ineligible |
| T-DXd | Muscle atrophy | all_indication_supportive | 3 | 0.53 (0.17-1.63) | 0.24 | Ineligible |
| T-DXd | Musculoskeletal discomfort | all_indication_supportive | 3 | 0.35 (0.11-1.08) | 0.17 | Ineligible |
| T-DXd | Musculoskeletal pain | all_indication_supportive | 3 | 0.12 (0.04-0.37) | 0.06 | Ineligible |
| T-DXd | Myocarditis | all_indication_supportive | 3 | 0.53 (0.17-1.63) | 0.24 | Ineligible |
| T-DXd | Nail bed inflammation | all_indication_supportive | 3 | 19.03 (6.09-59.39) | 2.67 | Ineligible |
| T-DXd | Nasal mucosal disorder | all_indication_supportive | 3 | 12.54 (4.03-39.05) | 1.79 | Ineligible |
| T-DXd | Neurological decompensation | all_indication_supportive | 3 | 2.72 (0.88-8.43) | 0.73 | Ineligible |
| T-DXd | Obstruction gastric | all_indication_supportive | 3 | 4.97 (1.60-15.44) | 0.98 | Ineligible |
| T-DXd | Ocular discomfort | all_indication_supportive | 3 | 0.51 (0.16-1.58) | 0.23 | Ineligible |
| T-DXd | Oesophageal carcinoma | all_indication_supportive | 3 | 0.17 (0.05-0.53) | 0.09 | Ineligible |
| T-DXd | Oesophageal stenosis | all_indication_supportive | 3 | 2.46 (0.79-7.63) | 0.69 | Ineligible |
| T-DXd | Optic neuritis | all_indication_supportive | 3 | 0.76 (0.25-2.37) | 0.32 | Ineligible |
| T-DXd | Oral disorder | all_indication_supportive | 3 | 0.86 (0.28-2.67) | 0.35 | Ineligible |
| T-DXd | Orthostatic hypotension | all_indication_supportive | 3 | 0.38 (0.12-1.19) | 0.18 | Ineligible |
| T-DXd | Orthostatic intolerance | all_indication_supportive | 3 | 8.07 (2.59-25.09) | 1.30 | Ineligible |
| T-DXd | Ovarian cyst | all_indication_supportive | 3 | 0.68 (0.22-2.11) | 0.29 | Ineligible |
| T-DXd | Pain of skin | all_indication_supportive | 3 | 0.20 (0.07-0.63) | 0.10 | Ineligible |
| T-DXd | Paraesthesia oral | all_indication_supportive | 3 | 0.44 (0.14-1.38) | 0.21 | Ineligible |
| T-DXd | Paresis | all_indication_supportive | 3 | 3.22 (1.04-9.99) | 0.79 | Ineligible |
| T-DXd | Pericarditis | all_indication_supportive | 3 | 0.36 (0.11-1.10) | 0.17 | Ineligible |
| T-DXd | Peritoneal disorder | all_indication_supportive | 3 | 10.02 (3.22-31.18) | 1.50 | Ineligible |
| T-DXd | Pigmentation disorder | all_indication_supportive | 3 | 1.00 (0.32-3.11) | 0.40 | Ineligible |
| T-DXd | Poor venous access | all_indication_supportive | 3 | 0.57 (0.18-1.75) | 0.26 | Ineligible |
| T-DXd | Portal vein thrombosis | all_indication_supportive | 3 | 2.08 (0.67-6.47) | 0.63 | Ineligible |
| T-DXd | Post procedural complication | all_indication_supportive | 3 | 0.40 (0.13-1.25) | 0.19 | Ineligible |
| T-DXd | Post procedural infection | all_indication_supportive | 3 | 0.71 (0.23-2.20) | 0.30 | Ineligible |
| T-DXd | Proctitis | all_indication_supportive | 3 | 2.19 (0.70-6.79) | 0.65 | Ineligible |
| T-DXd | Product administration interrupted | all_indication_supportive | 3 | 0.68 (0.22-2.11) | 0.30 | Ineligible |
| T-DXd | Product distribution issue | all_indication_supportive | 3 | 0.65 (0.21-2.02) | 0.29 | Ineligible |
| T-DXd | Product leakage | all_indication_supportive | 3 | 1.02 (0.33-3.17) | 0.40 | Ineligible |
| T-DXd | Psychiatric symptom | all_indication_supportive | 3 | 0.86 (0.28-2.68) | 0.35 | Ineligible |
| T-DXd | Pulmonary congestion | all_indication_supportive | 3 | 0.51 (0.17-1.60) | 0.24 | Ineligible |
| T-DXd | Pyelonephritis acute | all_indication_supportive | 3 | 3.26 (1.05-10.13) | 0.80 | Ineligible |
| T-DXd | Rash maculo-papular | all_indication_supportive | 3 | 0.30 (0.10-0.92) | 0.15 | Ineligible |
| T-DXd | Recurrent cancer | all_indication_supportive | 3 | 1.43 (0.46-4.44) | 0.50 | Ineligible |
| T-DXd | Renal tubular necrosis | all_indication_supportive | 3 | 0.70 (0.22-2.16) | 0.30 | Ineligible |
| T-DXd | Respiratory rate increased | all_indication_supportive | 3 | 0.84 (0.27-2.59) | 0.35 | Ineligible |
| T-DXd | Rhabdomyolysis | all_indication_supportive | 3 | 0.18 (0.06-0.56) | 0.09 | Ineligible |
| T-DXd | Rheumatoid arthritis | all_indication_supportive | 3 | 0.05 (0.02-0.16) | 0.03 | Ineligible |
| T-DXd | Rhinitis | all_indication_supportive | 3 | 0.77 (0.25-2.40) | 0.33 | Ineligible |
| T-DXd | Right ventricular enlargement | all_indication_supportive | 3 | 16.86 (5.41-52.60) | 2.35 | Ineligible |
| T-DXd | Scan abnormal | all_indication_supportive | 3 | 15.47 (4.96-48.24) | 2.16 | Ineligible |
| T-DXd | Shock | all_indication_supportive | 3 | 0.31 (0.10-0.96) | 0.15 | Ineligible |
| T-DXd | Skin infection | all_indication_supportive | 3 | 0.53 (0.17-1.63) | 0.24 | Ineligible |
| T-DXd | Skin irritation | all_indication_supportive | 3 | 0.12 (0.04-0.36) | 0.06 | Ineligible |
| T-DXd | Skin mass | all_indication_supportive | 3 | 0.83 (0.27-2.57) | 0.34 | Ineligible |
| T-DXd | Sluggishness | all_indication_supportive | 3 | 0.61 (0.20-1.89) | 0.27 | Ineligible |
| T-DXd | Splenic embolism | all_indication_supportive | 3 | 52.58 (16.65-166.03) | 8.74 | Ineligible |
| T-DXd | Staphylococcal infection | all_indication_supportive | 3 | 0.21 (0.07-0.66) | 0.11 | Ineligible |
| T-DXd | Status epilepticus | all_indication_supportive | 3 | 0.58 (0.19-1.81) | 0.26 | Ineligible |
| T-DXd | Streptococcal sepsis | all_indication_supportive | 3 | 5.88 (1.89-18.26) | 1.08 | Ineligible |
| T-DXd | Stress cardiomyopathy | all_indication_supportive | 3 | 1.00 (0.32-3.10) | 0.39 | Ineligible |
| T-DXd | Thrombophlebitis migrans | all_indication_supportive | 3 | 16.80 (5.39-52.41) | 2.34 | Ineligible |
| T-DXd | Treatment failure | all_indication_supportive | 3 | 0.06 (0.02-0.20) | 0.03 | Ineligible |
| T-DXd | Tuberculosis | all_indication_supportive | 3 | 0.49 (0.16-1.53) | 0.23 | Ineligible |
| T-DXd | Urinary bladder haemorrhage | all_indication_supportive | 3 | 2.92 (0.94-9.07) | 0.75 | Ineligible |
| T-DXd | Urinary incontinence | all_indication_supportive | 3 | 0.23 (0.07-0.70) | 0.12 | Ineligible |
| T-DXd | Urticaria | all_indication_supportive | 3 | 0.04 (0.01-0.12) | 0.02 | Ineligible |
| T-DXd | Vascular device occlusion | all_indication_supportive | 3 | 16.25 (5.21-50.66) | 2.27 | Ineligible |
| T-DXd | Venoocclusive liver disease | all_indication_supportive | 3 | 1.29 (0.41-3.99) | 0.47 | Ineligible |
| T-DXd | Venous thrombosis limb | all_indication_supportive | 3 | 3.17 (1.02-9.85) | 0.79 | Ineligible |
| T-DXd | Vertebral lesion | all_indication_supportive | 3 | 10.10 (3.25-31.44) | 1.51 | Ineligible |
| T-DXd | Volvulus | all_indication_supportive | 3 | 2.60 (0.84-8.06) | 0.71 | Ineligible |
| T-DXd | Vomiting projectile | all_indication_supportive | 3 | 2.24 (0.72-6.94) | 0.65 | Ineligible |
| T-DXd | White coat hypertension | all_indication_supportive | 3 | 9.72 (3.12-30.25) | 1.47 | Ineligible |
| SG | Disease progression | breast_cancer_primary | 920 | 14.08 (13.00-15.26) | 8.75 | Eligible |
| SG | Diarrhoea | breast_cancer_primary | 451 | 1.91 (1.73-2.12) | 1.62 | Eligible |
| SG | Death | breast_cancer_primary | 400 | 2.19 (1.97-2.43) | 1.84 | Eligible |
| SG | Neutropenia | breast_cancer_primary | 381 | 2.82 (2.53-3.14) | 2.32 | Eligible |
| SG | Inappropriate schedule of product administration | breast_cancer_primary | 306 | 23.22 (20.38-26.45) | 15.49 | Eligible |
| SG | Fatigue | breast_cancer_primary | 223 | 0.78 (0.68-0.90) | 0.72 | Ineligible |
| SG | Fatigue | breast_cancer_primary | 223 | 0.78 (0.68-0.90) | 0.72 | Ineligible |
| SG | Off label use | breast_cancer_primary | 208 | 1.60 (1.39-1.85) | 1.37 | Eligible |
| SG | Nausea | breast_cancer_primary | 205 | 0.85 (0.74-0.98) | 0.77 | Ineligible |
| SG | Nausea | breast_cancer_primary | 205 | 0.85 (0.74-0.98) | 0.77 | Ineligible |
| SG | Asthenia | breast_cancer_primary | 193 | 1.69 (1.46-1.95) | 1.43 | Eligible |
| SG | Weight fluctuation | breast_cancer_primary | 161 | 91.76 (73.49-114.59) | 37.47 | Eligible |
| SG | Triple negative breast cancer | breast_cancer_primary | 150 | 107.50 (84.60-136.60) | 40.12 | Eligible |
| SG | Alopecia | breast_cancer_primary | 147 | 0.45 (0.38-0.53) | 0.42 | Ineligible |
| SG | Alopecia | breast_cancer_primary | 147 | 0.45 (0.38-0.53) | 0.42 | Ineligible |
| SG | Neutrophil count decreased | breast_cancer_primary | 137 | 2.95 (2.48-3.51) | 2.36 | Eligible |
| SG | Febrile neutropenia | breast_cancer_primary | 136 | 3.00 (2.52-3.58) | 2.40 | Eligible |
| SG | Weight decreased | breast_cancer_primary | 134 | 2.17 (1.82-2.58) | 1.77 | Eligible |
| SG | Anaemia | breast_cancer_primary | 123 | 1.45 (1.21-1.74) | 1.22 | Eligible |
| SG | Vomiting | breast_cancer_primary | 117 | 0.89 (0.74-1.07) | 0.77 | Ineligible |
| SG | Vomiting | breast_cancer_primary | 117 | 0.89 (0.74-1.07) | 0.77 | Ineligible |
| SG | Malaise | breast_cancer_primary | 107 | 1.29 (1.07-1.57) | 1.08 | Eligible |
| SG | Myelosuppression | breast_cancer_primary | 96 | 1.51 (1.23-1.85) | 1.23 | Eligible |
| SG | Constipation | breast_cancer_primary | 95 | 1.39 (1.13-1.70) | 1.14 | Eligible |
| SG | Thrombocytopenia | breast_cancer_primary | 95 | 1.84 (1.50-2.26) | 1.48 | Eligible |
| SG | Pyrexia | breast_cancer_primary | 91 | 1.02 (0.83-1.26) | 0.86 | Ineligible |
| SG | Pyrexia | breast_cancer_primary | 91 | 1.02 (0.83-1.26) | 0.86 | Ineligible |
| SG | Weight increased | breast_cancer_primary | 91 | 3.69 (2.98-4.56) | 2.82 | Eligible |
| SG | White blood cell count decreased | breast_cancer_primary | 84 | 0.58 (0.46-0.72) | 0.50 | Ineligible |
| SG | White blood cell count decreased | breast_cancer_primary | 84 | 0.58 (0.46-0.72) | 0.50 | Ineligible |
| SG | Dyspnoea | breast_cancer_primary | 81 | 0.66 (0.53-0.82) | 0.56 | Ineligible |
| SG | Dyspnoea | breast_cancer_primary | 81 | 0.66 (0.53-0.82) | 0.56 | Ineligible |
| SG | Abdominal pain | breast_cancer_primary | 72 | 1.68 (1.33-2.13) | 1.32 | Eligible |
| SG | Decreased appetite | breast_cancer_primary | 70 | 0.71 (0.56-0.90) | 0.59 | Ineligible |
| SG | Decreased appetite | breast_cancer_primary | 70 | 0.71 (0.56-0.90) | 0.59 | Ineligible |
| SG | Drug ineffective | breast_cancer_primary | 69 | 1.02 (0.80-1.29) | 0.83 | Ineligible |
| SG | Drug ineffective | breast_cancer_primary | 69 | 1.02 (0.80-1.29) | 0.83 | Ineligible |
| SG | Heart rate increased | breast_cancer_primary | 68 | 6.20 (4.84-7.96) | 4.68 | Eligible |
| SG | Pain | breast_cancer_primary | 65 | 0.47 (0.37-0.60) | 0.40 | Ineligible |
| SG | Pain | breast_cancer_primary | 65 | 0.47 (0.37-0.60) | 0.40 | Ineligible |
| SG | COVID-19 | breast_cancer_primary | 61 | 2.16 (1.67-2.79) | 1.63 | Eligible |
| SG | Metastases to central nervous system | breast_cancer_primary | 60 | 2.25 (1.74-2.92) | 1.68 | Eligible |
| SG | General physical health deterioration | breast_cancer_primary | 56 | 1.53 (1.18-2.00) | 1.18 | Eligible |
| SG | Hypertension | breast_cancer_primary | 56 | 1.75 (1.34-2.29) | 1.33 | Eligible |
| SG | Hypotension | breast_cancer_primary | 56 | 2.35 (1.80-3.07) | 1.73 | Eligible |
| SG | Cough | breast_cancer_primary | 52 | 0.75 (0.57-0.99) | 0.61 | Ineligible |
| SG | Cough | breast_cancer_primary | 52 | 0.75 (0.57-0.99) | 0.61 | Ineligible |
| SG | Metastases to liver | breast_cancer_primary | 48 | 1.20 (0.90-1.60) | 0.93 | Ineligible |
| SG | Metastases to liver | breast_cancer_primary | 48 | 1.20 (0.90-1.60) | 0.93 | Ineligible |
| SG | Headache | breast_cancer_primary | 46 | 0.55 (0.41-0.74) | 0.45 | Ineligible |
| SG | Headache | breast_cancer_primary | 46 | 0.55 (0.41-0.74) | 0.45 | Ineligible |
| SG | Dizziness | breast_cancer_primary | 45 | 0.65 (0.48-0.87) | 0.52 | Ineligible |
| SG | Dizziness | breast_cancer_primary | 45 | 0.65 (0.48-0.87) | 0.52 | Ineligible |
| SG | Colitis | breast_cancer_primary | 44 | 3.61 (2.66-4.89) | 2.45 | Eligible |
| SG | Neutropenic colitis | breast_cancer_primary | 43 | 28.61 (20.23-40.45) | 16.04 | Eligible |
| SG | Rash | breast_cancer_primary | 43 | 0.49 (0.36-0.67) | 0.40 | Ineligible |
| SG | Rash | breast_cancer_primary | 43 | 0.49 (0.36-0.67) | 0.40 | Ineligible |
| SG | Septic shock | breast_cancer_primary | 42 | 5.75 (4.20-7.88) | 4.09 | Eligible |
| SG | Sepsis | breast_cancer_primary | 41 | 2.08 (1.52-2.84) | 1.47 | Eligible |
| SG | Pleural effusion | breast_cancer_primary | 37 | 1.00 (0.72-1.38) | 0.76 | Ineligible |
| SG | Pleural effusion | breast_cancer_primary | 37 | 1.00 (0.72-1.38) | 0.76 | Ineligible |
| SG | Pneumonia | breast_cancer_primary | 37 | 0.69 (0.50-0.96) | 0.54 | Ineligible |
| SG | Pneumonia | breast_cancer_primary | 37 | 0.69 (0.50-0.96) | 0.54 | Ineligible |
| SG | Platelet count decreased | breast_cancer_primary | 36 | 0.70 (0.51-0.98) | 0.55 | Ineligible |
| SG | Platelet count decreased | breast_cancer_primary | 36 | 0.70 (0.51-0.98) | 0.55 | Ineligible |
| SG | Dehydration | breast_cancer_primary | 35 | 0.93 (0.66-1.29) | 0.70 | Ineligible |
| SG | Dehydration | breast_cancer_primary | 35 | 0.93 (0.66-1.29) | 0.70 | Ineligible |
| SG | Pancytopenia | breast_cancer_primary | 34 | 1.73 (1.23-2.43) | 1.21 | Eligible |
| SG | Abdominal pain upper | breast_cancer_primary | 33 | 0.89 (0.63-1.25) | 0.67 | Ineligible |
| SG | Abdominal pain upper | breast_cancer_primary | 33 | 0.89 (0.63-1.25) | 0.67 | Ineligible |
| SG | Leukopenia | breast_cancer_primary | 33 | 0.67 (0.47-0.94) | 0.52 | Ineligible |
| SG | Leukopenia | breast_cancer_primary | 33 | 0.67 (0.47-0.94) | 0.52 | Ineligible |
| SG | Infection | breast_cancer_primary | 32 | 1.37 (0.96-1.94) | 0.98 | Ineligible |
| SG | Infection | breast_cancer_primary | 32 | 1.37 (0.96-1.94) | 0.98 | Ineligible |
| SG | Febrile bone marrow aplasia | breast_cancer_primary | 31 | 11.02 (7.57-16.05) | 7.15 | Eligible |
| SG | Pneumonitis | breast_cancer_primary | 31 | 1.14 (0.80-1.63) | 0.83 | Ineligible |
| SG | Pneumonitis | breast_cancer_primary | 31 | 1.14 (0.80-1.63) | 0.83 | Ineligible |
| SG | Ill-defined disorder | breast_cancer_primary | 30 | 3.56 (2.47-5.14) | 2.19 | Eligible |
| SG | Urinary tract infection | breast_cancer_primary | 30 | 0.85 (0.60-1.23) | 0.64 | Ineligible |
| SG | Urinary tract infection | breast_cancer_primary | 30 | 0.85 (0.60-1.23) | 0.64 | Ineligible |
| SG | Metastases to bone | breast_cancer_primary | 29 | 0.55 (0.38-0.80) | 0.43 | Ineligible |
| SG | Metastases to bone | breast_cancer_primary | 29 | 0.55 (0.38-0.80) | 0.43 | Ineligible |
| SG | Product dose omission issue | breast_cancer_primary | 29 | 0.98 (0.68-1.41) | 0.72 | Ineligible |
| SG | Product dose omission issue | breast_cancer_primary | 29 | 0.98 (0.68-1.41) | 0.72 | Ineligible |
| SG | General physical condition abnormal | breast_cancer_primary | 28 | 18.97 (12.59-28.60) | 10.97 | Eligible |
| SG | Mucosal inflammation | breast_cancer_primary | 28 | 1.07 (0.73-1.55) | 0.77 | Ineligible |
| SG | Mucosal inflammation | breast_cancer_primary | 28 | 1.07 (0.73-1.55) | 0.77 | Ineligible |
| SG | Nasopharyngitis | breast_cancer_primary | 28 | 1.00 (0.69-1.46) | 0.73 | Ineligible |
| SG | Nasopharyngitis | breast_cancer_primary | 28 | 1.00 (0.69-1.46) | 0.73 | Ineligible |
| SG | Neuropathy peripheral | breast_cancer_primary | 28 | 0.47 (0.32-0.68) | 0.36 | Ineligible |
| SG | Neuropathy peripheral | breast_cancer_primary | 28 | 0.47 (0.32-0.68) | 0.36 | Ineligible |
| SG | Hypokalaemia | breast_cancer_primary | 27 | 1.46 (1.00-2.14) | 1.01 | Ineligible |
| SG | Hypokalaemia | breast_cancer_primary | 27 | 1.46 (1.00-2.14) | 1.01 | Ineligible |
| SG | Intentional dose omission | breast_cancer_primary | 26 | 11.86 (7.86-17.89) | 7.39 | Eligible |
| SG | Neutrophil count abnormal | breast_cancer_primary | 26 | 9.50 (6.33-14.27) | 6.11 | Eligible |
| SG | Hepatic failure | breast_cancer_primary | 25 | 2.06 (1.39-3.07) | 1.32 | Eligible |
| SG | Pain in extremity | breast_cancer_primary | 25 | 0.42 (0.29-0.63) | 0.33 | Ineligible |
| SG | Pain in extremity | breast_cancer_primary | 25 | 0.42 (0.29-0.63) | 0.33 | Ineligible |
| SG | Oxygen saturation decreased | breast_cancer_primary | 24 | 2.41 (1.60-3.61) | 1.48 | Eligible |
| SG | Stomatitis | breast_cancer_primary | 24 | 0.41 (0.27-0.61) | 0.32 | Ineligible |
| SG | Stomatitis | breast_cancer_primary | 24 | 0.41 (0.27-0.61) | 0.32 | Ineligible |
| SG | Haemoglobin decreased | breast_cancer_primary | 23 | 0.59 (0.39-0.90) | 0.44 | Ineligible |
| SG | Haemoglobin decreased | breast_cancer_primary | 23 | 0.59 (0.39-0.90) | 0.44 | Ineligible |
| SG | Metastases to lung | breast_cancer_primary | 23 | 0.91 (0.60-1.38) | 0.65 | Ineligible |
| SG | Metastases to lung | breast_cancer_primary | 23 | 0.91 (0.60-1.38) | 0.65 | Ineligible |
| SG | Back pain | breast_cancer_primary | 22 | 0.38 (0.25-0.58) | 0.29 | Ineligible |
| SG | Back pain | breast_cancer_primary | 22 | 0.38 (0.25-0.58) | 0.29 | Ineligible |
| SG | Interstitial lung disease | breast_cancer_primary | 22 | 0.63 (0.41-0.96) | 0.46 | Ineligible |
| SG | Interstitial lung disease | breast_cancer_primary | 22 | 0.63 (0.41-0.96) | 0.46 | Ineligible |
| SG | Liver disorder | breast_cancer_primary | 22 | 1.77 (1.16-2.71) | 1.13 | Eligible |
| SG | Agranulocytosis | breast_cancer_primary | 21 | 1.67 (1.08-2.58) | 1.07 | Eligible |
| SG | Arthralgia | breast_cancer_primary | 21 | 0.24 (0.15-0.36) | 0.19 | Ineligible |
| SG | Arthralgia | breast_cancer_primary | 21 | 0.24 (0.15-0.36) | 0.19 | Ineligible |
| SG | Ascites | breast_cancer_primary | 21 | 1.26 (0.82-1.95) | 0.85 | Ineligible |
| SG | Ascites | breast_cancer_primary | 21 | 1.26 (0.82-1.95) | 0.85 | Ineligible |
| SG | Cholestasis | breast_cancer_primary | 21 | 3.52 (2.27-5.46) | 1.94 | Eligible |
| SG | Pruritus | breast_cancer_primary | 21 | 0.37 (0.24-0.56) | 0.28 | Ineligible |
| SG | Pruritus | breast_cancer_primary | 21 | 0.37 (0.24-0.56) | 0.28 | Ineligible |
| SG | Acute kidney injury | breast_cancer_primary | 20 | 0.85 (0.55-1.32) | 0.60 | Ineligible |
| SG | Acute kidney injury | breast_cancer_primary | 20 | 0.85 (0.55-1.32) | 0.60 | Ineligible |
| SG | Chills | breast_cancer_primary | 20 | 0.70 (0.45-1.09) | 0.51 | Ineligible |
| SG | Chills | breast_cancer_primary | 20 | 0.70 (0.45-1.09) | 0.51 | Ineligible |
| SG | Product use issue | breast_cancer_primary | 20 | 0.90 (0.58-1.40) | 0.63 | Ineligible |
| SG | Product use issue | breast_cancer_primary | 20 | 0.90 (0.58-1.40) | 0.63 | Ineligible |
| SG | Somnolence | breast_cancer_primary | 20 | 1.04 (0.67-1.62) | 0.71 | Ineligible |
| SG | Somnolence | breast_cancer_primary | 20 | 1.04 (0.67-1.62) | 0.71 | Ineligible |
| SG | Bone pain | breast_cancer_primary | 19 | 0.43 (0.27-0.68) | 0.32 | Ineligible |
| SG | Bone pain | breast_cancer_primary | 19 | 0.43 (0.27-0.68) | 0.32 | Ineligible |
| SG | Haematotoxicity | breast_cancer_primary | 19 | 2.43 (1.54-3.83) | 1.39 | Eligible |
| SG | Hyponatraemia | breast_cancer_primary | 19 | 1.80 (1.14-2.83) | 1.10 | Eligible |
| SG | Infusion related reaction | breast_cancer_primary | 19 | 1.30 (0.82-2.04) | 0.85 | Ineligible |
| SG | Infusion related reaction | breast_cancer_primary | 19 | 1.30 (0.82-2.04) | 0.85 | Ineligible |
| SG | Breast cancer metastatic | breast_cancer_primary | 18 | 0.31 (0.19-0.49) | 0.24 | Ineligible |
| SG | Breast cancer metastatic | breast_cancer_primary | 18 | 0.31 (0.19-0.49) | 0.24 | Ineligible |
| SG | Cytopenia | breast_cancer_primary | 18 | 2.81 (1.76-4.51) | 1.53 | Eligible |
| SG | Fall | breast_cancer_primary | 18 | 0.52 (0.32-0.82) | 0.38 | Ineligible |
| SG | Fall | breast_cancer_primary | 18 | 0.52 (0.32-0.82) | 0.38 | Ineligible |
| SG | Condition aggravated | breast_cancer_primary | 17 | 0.66 (0.41-1.07) | 0.47 | Ineligible |
| SG | Condition aggravated | breast_cancer_primary | 17 | 0.66 (0.41-1.07) | 0.47 | Ineligible |
| SG | Erythema | breast_cancer_primary | 17 | 0.43 (0.27-0.70) | 0.32 | Ineligible |
| SG | Erythema | breast_cancer_primary | 17 | 0.43 (0.27-0.70) | 0.32 | Ineligible |
| SG | Hepatic cytolysis | breast_cancer_primary | 17 | 1.72 (1.07-2.79) | 1.04 | Eligible |
| SG | Metastases to lymph nodes | breast_cancer_primary | 17 | 1.26 (0.78-2.03) | 0.81 | Ineligible |
| SG | Metastases to lymph nodes | breast_cancer_primary | 17 | 1.26 (0.78-2.03) | 0.81 | Ineligible |
| SG | SARS-CoV-2 test positive | breast_cancer_primary | 17 | 6.99 (4.26-11.48) | 4.08 | Eligible |
| SG | Chest pain | breast_cancer_primary | 16 | 0.59 (0.36-0.97) | 0.42 | Ineligible |
| SG | Chest pain | breast_cancer_primary | 16 | 0.59 (0.36-0.97) | 0.42 | Ineligible |
| SG | Neoplasm progression | breast_cancer_primary | 16 | 0.16 (0.10-0.26) | 0.13 | Ineligible |
| SG | Neoplasm progression | breast_cancer_primary | 16 | 0.16 (0.10-0.26) | 0.13 | Ineligible |
| SG | Neutropenic sepsis | breast_cancer_primary | 16 | 3.40 (2.06-5.61) | 1.69 | Eligible |
| SG | Full blood count decreased | breast_cancer_primary | 15 | 0.80 (0.48-1.33) | 0.54 | Ineligible |
| SG | Full blood count decreased | breast_cancer_primary | 15 | 0.80 (0.48-1.33) | 0.54 | Ineligible |
| SG | Hypoaesthesia | breast_cancer_primary | 15 | 0.49 (0.29-0.81) | 0.35 | Ineligible |
| SG | Hypoaesthesia | breast_cancer_primary | 15 | 0.49 (0.29-0.81) | 0.35 | Ineligible |
| SG | Illness | breast_cancer_primary | 15 | 0.74 (0.44-1.23) | 0.50 | Ineligible |
| SG | Illness | breast_cancer_primary | 15 | 0.74 (0.44-1.23) | 0.50 | Ineligible |
| SG | Oropharyngeal pain | breast_cancer_primary | 15 | 0.86 (0.51-1.43) | 0.57 | Ineligible |
| SG | Oropharyngeal pain | breast_cancer_primary | 15 | 0.86 (0.51-1.43) | 0.57 | Ineligible |
| SG | Poor venous access | breast_cancer_primary | 15 | 13.36 (7.75-23.03) | 7.25 | Eligible |
| SG | Productive cough | breast_cancer_primary | 15 | 1.97 (1.18-3.30) | 1.11 | Eligible |
| SG | Toxicity to various agents | breast_cancer_primary | 15 | 0.93 (0.56-1.55) | 0.61 | Ineligible |
| SG | Toxicity to various agents | breast_cancer_primary | 15 | 0.93 (0.56-1.55) | 0.61 | Ineligible |
| SG | Anxiety | breast_cancer_primary | 14 | 0.18 (0.11-0.30) | 0.14 | Ineligible |
| SG | Anxiety | breast_cancer_primary | 14 | 0.18 (0.11-0.30) | 0.14 | Ineligible |
| SG | Blood bilirubin increased | breast_cancer_primary | 14 | 1.26 (0.74-2.13) | 0.77 | Ineligible |
| SG | Blood bilirubin increased | breast_cancer_primary | 14 | 1.26 (0.74-2.13) | 0.77 | Ineligible |
| SG | Breast cancer | breast_cancer_primary | 14 | 0.28 (0.16-0.47) | 0.21 | Ineligible |
| SG | Breast cancer | breast_cancer_primary | 14 | 0.28 (0.16-0.47) | 0.21 | Ineligible |
| SG | Cardiac failure | breast_cancer_primary | 14 | 0.58 (0.34-0.98) | 0.40 | Ineligible |
| SG | Cardiac failure | breast_cancer_primary | 14 | 0.58 (0.34-0.98) | 0.40 | Ineligible |
| SG | Enteritis | breast_cancer_primary | 14 | 9.52 (5.47-16.55) | 5.32 | Eligible |
| SG | Feeling abnormal | breast_cancer_primary | 14 | 0.49 (0.29-0.83) | 0.35 | Ineligible |
| SG | Feeling abnormal | breast_cancer_primary | 14 | 0.49 (0.29-0.83) | 0.35 | Ineligible |
| SG | Hypersensitivity | breast_cancer_primary | 14 | 0.52 (0.31-0.88) | 0.37 | Ineligible |
| SG | Hypersensitivity | breast_cancer_primary | 14 | 0.52 (0.31-0.88) | 0.37 | Ineligible |
| SG | Intentional product use issue | breast_cancer_primary | 14 | 1.34 (0.79-2.28) | 0.82 | Ineligible |
| SG | Intentional product use issue | breast_cancer_primary | 14 | 1.34 (0.79-2.28) | 0.82 | Ineligible |
| SG | Malignant neoplasm progression | breast_cancer_primary | 14 | 0.10 (0.06-0.18) | 0.08 | Ineligible |
| SG | Malignant neoplasm progression | breast_cancer_primary | 14 | 0.10 (0.06-0.18) | 0.08 | Ineligible |
| SG | Metastases to meninges | breast_cancer_primary | 14 | 3.76 (2.20-6.43) | 1.74 | Eligible |
| SG | Paraesthesia | breast_cancer_primary | 14 | 0.44 (0.26-0.75) | 0.32 | Ineligible |
| SG | Paraesthesia | breast_cancer_primary | 14 | 0.44 (0.26-0.75) | 0.32 | Ineligible |
| SG | Pulmonary embolism | breast_cancer_primary | 14 | 0.58 (0.34-0.98) | 0.40 | Ineligible |
| SG | Pulmonary embolism | breast_cancer_primary | 14 | 0.58 (0.34-0.98) | 0.40 | Ineligible |
| SG | Blood test abnormal | breast_cancer_primary | 13 | 1.89 (1.09-3.27) | 1.03 | Eligible |
| SG | Chest discomfort | breast_cancer_primary | 13 | 0.62 (0.36-1.07) | 0.42 | Ineligible |
| SG | Chest discomfort | breast_cancer_primary | 13 | 0.62 (0.36-1.07) | 0.42 | Ineligible |
| SG | Dysphagia | breast_cancer_primary | 13 | 0.63 (0.37-1.09) | 0.43 | Ineligible |
| SG | Dysphagia | breast_cancer_primary | 13 | 0.63 (0.37-1.09) | 0.43 | Ineligible |
| SG | Gamma-glutamyltransferase increased | breast_cancer_primary | 13 | 1.16 (0.67-2.01) | 0.71 | Ineligible |
| SG | Gamma-glutamyltransferase increased | breast_cancer_primary | 13 | 1.16 (0.67-2.01) | 0.71 | Ineligible |
| SG | Hepatic function abnormal | breast_cancer_primary | 13 | 0.65 (0.38-1.12) | 0.44 | Ineligible |
| SG | Hepatic function abnormal | breast_cancer_primary | 13 | 0.65 (0.38-1.12) | 0.44 | Ineligible |
| SG | Product preparation error | breast_cancer_primary | 13 | 20.41 (11.14-37.39) | 9.62 | Eligible |
| SG | Red blood cell count decreased | breast_cancer_primary | 13 | 0.48 (0.28-0.84) | 0.34 | Ineligible |
| SG | Red blood cell count decreased | breast_cancer_primary | 13 | 0.48 (0.28-0.84) | 0.34 | Ineligible |
| SG | Respiratory failure | breast_cancer_primary | 13 | 0.92 (0.53-1.59) | 0.59 | Ineligible |
| SG | Respiratory failure | breast_cancer_primary | 13 | 0.92 (0.53-1.59) | 0.59 | Ineligible |
| SG | Tachycardia | breast_cancer_primary | 13 | 0.87 (0.50-1.50) | 0.57 | Ineligible |
| SG | Tachycardia | breast_cancer_primary | 13 | 0.87 (0.50-1.50) | 0.57 | Ineligible |
| SG | Abdominal discomfort | breast_cancer_primary | 12 | 0.40 (0.23-0.71) | 0.29 | Ineligible |
| SG | Abdominal discomfort | breast_cancer_primary | 12 | 0.40 (0.23-0.71) | 0.29 | Ineligible |
| SG | Alanine aminotransferase increased | breast_cancer_primary | 12 | 0.46 (0.26-0.80) | 0.32 | Ineligible |
| SG | Alanine aminotransferase increased | breast_cancer_primary | 12 | 0.46 (0.26-0.80) | 0.32 | Ineligible |
| SG | Cell death | breast_cancer_primary | 12 | 7.45 (4.13-13.45) | 3.74 | Eligible |
| SG | Complication associated with device | breast_cancer_primary | 12 | 22.52 (11.92-42.54) | 10.05 | Eligible |
| SG | Drug-induced liver injury | breast_cancer_primary | 12 | 1.18 (0.67-2.09) | 0.71 | Ineligible |
| SG | Drug-induced liver injury | breast_cancer_primary | 12 | 1.18 (0.67-2.09) | 0.71 | Ineligible |
| SG | Dry skin | breast_cancer_primary | 12 | 0.36 (0.20-0.63) | 0.26 | Ineligible |
| SG | Dry skin | breast_cancer_primary | 12 | 0.36 (0.20-0.63) | 0.26 | Ineligible |
| SG | Enterocolitis | breast_cancer_primary | 12 | 5.18 (2.89-9.28) | 2.18 | Eligible |
| SG | Gastrooesophageal reflux disease | breast_cancer_primary | 12 | 0.91 (0.51-1.60) | 0.58 | Ineligible |
| SG | Gastrooesophageal reflux disease | breast_cancer_primary | 12 | 0.91 (0.51-1.60) | 0.58 | Ineligible |
| SG | Hepatotoxicity | breast_cancer_primary | 12 | 0.85 (0.48-1.51) | 0.55 | Ineligible |
| SG | Hepatotoxicity | breast_cancer_primary | 12 | 0.85 (0.48-1.51) | 0.55 | Ineligible |
| SG | Herpes zoster | breast_cancer_primary | 12 | 1.52 (0.86-2.70) | 0.86 | Ineligible |
| SG | Herpes zoster | breast_cancer_primary | 12 | 1.52 (0.86-2.70) | 0.86 | Ineligible |
| SG | Renal failure | breast_cancer_primary | 12 | 0.72 (0.41-1.28) | 0.48 | Ineligible |
| SG | Renal failure | breast_cancer_primary | 12 | 0.72 (0.41-1.28) | 0.48 | Ineligible |
| SG | Therapeutic response decreased | breast_cancer_primary | 12 | 3.19 (1.79-5.69) | 1.43 | Eligible |
| SG | Thrombosis | breast_cancer_primary | 12 | 0.71 (0.40-1.25) | 0.47 | Ineligible |
| SG | Thrombosis | breast_cancer_primary | 12 | 0.71 (0.40-1.25) | 0.47 | Ineligible |
| SG | Aspartate aminotransferase increased | breast_cancer_primary | 11 | 0.45 (0.25-0.81) | 0.31 | Ineligible |
| SG | Aspartate aminotransferase increased | breast_cancer_primary | 11 | 0.45 (0.25-0.81) | 0.31 | Ineligible |
| SG | Depression | breast_cancer_primary | 11 | 0.33 (0.18-0.60) | 0.24 | Ineligible |
| SG | Depression | breast_cancer_primary | 11 | 0.33 (0.18-0.60) | 0.24 | Ineligible |
| SG | Gastrointestinal disorder | breast_cancer_primary | 11 | 0.64 (0.35-1.16) | 0.42 | Ineligible |
| SG | Gastrointestinal disorder | breast_cancer_primary | 11 | 0.64 (0.35-1.16) | 0.42 | Ineligible |
| SG | Hepatic enzyme increased | breast_cancer_primary | 11 | 0.63 (0.35-1.15) | 0.42 | Ineligible |
| SG | Hepatic enzyme increased | breast_cancer_primary | 11 | 0.63 (0.35-1.15) | 0.42 | Ineligible |
| SG | Incorrect dose administered | breast_cancer_primary | 11 | 1.56 (0.86-2.83) | 0.85 | Ineligible |
| SG | Incorrect dose administered | breast_cancer_primary | 11 | 1.56 (0.86-2.83) | 0.85 | Ineligible |
| SG | Insomnia | breast_cancer_primary | 11 | 0.28 (0.15-0.50) | 0.20 | Ineligible |
| SG | Insomnia | breast_cancer_primary | 11 | 0.28 (0.15-0.50) | 0.20 | Ineligible |
| SG | Memory impairment | breast_cancer_primary | 11 | 0.44 (0.25-0.81) | 0.31 | Ineligible |
| SG | Memory impairment | breast_cancer_primary | 11 | 0.44 (0.25-0.81) | 0.31 | Ineligible |
| SG | Multiple organ dysfunction syndrome | breast_cancer_primary | 11 | 1.95 (1.07-3.55) | 1.00 | Eligible |
| SG | Product storage error | breast_cancer_primary | 11 | 6.16 (3.34-11.37) | 2.58 | Eligible |
| SG | Therapy partial responder | breast_cancer_primary | 11 | 1.58 (0.87-2.87) | 0.86 | Ineligible |
| SG | Therapy partial responder | breast_cancer_primary | 11 | 1.58 (0.87-2.87) | 0.86 | Ineligible |
| SG | Dry mouth | breast_cancer_primary | 10 | 0.56 (0.30-1.04) | 0.37 | Ineligible |
| SG | Dry mouth | breast_cancer_primary | 10 | 0.56 (0.30-1.04) | 0.37 | Ineligible |
| SG | Dyspepsia | breast_cancer_primary | 10 | 0.42 (0.23-0.79) | 0.29 | Ineligible |
| SG | Dyspepsia | breast_cancer_primary | 10 | 0.42 (0.23-0.79) | 0.29 | Ineligible |
| SG | Flushing | breast_cancer_primary | 10 | 0.71 (0.38-1.32) | 0.46 | Ineligible |
| SG | Flushing | breast_cancer_primary | 10 | 0.71 (0.38-1.32) | 0.46 | Ineligible |
| SG | Hypophagia | breast_cancer_primary | 10 | 1.68 (0.90-3.15) | 0.87 | Ineligible |
| SG | Hypophagia | breast_cancer_primary | 10 | 1.68 (0.90-3.15) | 0.87 | Ineligible |
| SG | Laboratory test abnormal | breast_cancer_primary | 10 | 1.35 (0.72-2.53) | 0.75 | Ineligible |
| SG | Laboratory test abnormal | breast_cancer_primary | 10 | 1.35 (0.72-2.53) | 0.75 | Ineligible |
| SG | Lung disorder | breast_cancer_primary | 10 | 0.82 (0.44-1.53) | 0.51 | Ineligible |
| SG | Lung disorder | breast_cancer_primary | 10 | 0.82 (0.44-1.53) | 0.51 | Ineligible |
| SG | Myalgia | breast_cancer_primary | 10 | 0.29 (0.15-0.54) | 0.21 | Ineligible |
| SG | Myalgia | breast_cancer_primary | 10 | 0.29 (0.15-0.54) | 0.21 | Ineligible |
| SG | Seizure | breast_cancer_primary | 10 | 0.77 (0.41-1.44) | 0.49 | Ineligible |
| SG | Seizure | breast_cancer_primary | 10 | 0.77 (0.41-1.44) | 0.49 | Ineligible |
| SG | Syncope | breast_cancer_primary | 10 | 0.73 (0.39-1.37) | 0.47 | Ineligible |
| SG | Syncope | breast_cancer_primary | 10 | 0.73 (0.39-1.37) | 0.47 | Ineligible |
| SG | Abdominal distension | breast_cancer_primary | 9 | 0.53 (0.28-1.03) | 0.35 | Ineligible |
| SG | Abdominal distension | breast_cancer_primary | 9 | 0.53 (0.28-1.03) | 0.35 | Ineligible |
| SG | Cerebrovascular accident | breast_cancer_primary | 9 | 0.64 (0.33-1.23) | 0.41 | Ineligible |
| SG | Cerebrovascular accident | breast_cancer_primary | 9 | 0.64 (0.33-1.23) | 0.41 | Ineligible |
| SG | Electrolyte imbalance | breast_cancer_primary | 9 | 2.86 (1.47-5.56) | 1.17 | Ineligible |
| SG | Electrolyte imbalance | breast_cancer_primary | 9 | 2.86 (1.47-5.56) | 1.17 | Ineligible |
| SG | Gait disturbance | breast_cancer_primary | 9 | 0.30 (0.16-0.58) | 0.22 | Ineligible |
| SG | Gait disturbance | breast_cancer_primary | 9 | 0.30 (0.16-0.58) | 0.22 | Ineligible |
| SG | Gastrointestinal toxicity | breast_cancer_primary | 9 | 2.66 (1.37-5.16) | 1.12 | Ineligible |
| SG | Gastrointestinal toxicity | breast_cancer_primary | 9 | 2.66 (1.37-5.16) | 1.12 | Ineligible |
| SG | Hyperthermia | breast_cancer_primary | 9 | 4.22 (2.16-8.24) | 1.51 | Ineligible |
| SG | Hyperthermia | breast_cancer_primary | 9 | 4.22 (2.16-8.24) | 1.51 | Ineligible |
| SG | Liver function test abnormal | breast_cancer_primary | 9 | 1.65 (0.85-3.20) | 0.84 | Ineligible |
| SG | Liver function test abnormal | breast_cancer_primary | 9 | 1.65 (0.85-3.20) | 0.84 | Ineligible |
| SG | Lymphopenia | breast_cancer_primary | 9 | 1.82 (0.94-3.52) | 0.89 | Ineligible |
| SG | Lymphopenia | breast_cancer_primary | 9 | 1.82 (0.94-3.52) | 0.89 | Ineligible |
| SG | Metastasis | breast_cancer_primary | 9 | 0.74 (0.38-1.43) | 0.46 | Ineligible |
| SG | Metastasis | breast_cancer_primary | 9 | 0.74 (0.38-1.43) | 0.46 | Ineligible |
| SG | Product preparation issue | breast_cancer_primary | 9 | 43.12 (19.36-96.05) | 13.23 | Ineligible |
| SG | Product preparation issue | breast_cancer_primary | 9 | 43.12 (19.36-96.05) | 13.23 | Ineligible |
| SG | Respiratory tract infection | breast_cancer_primary | 9 | 2.46 (1.27-4.78) | 1.07 | Ineligible |
| SG | Respiratory tract infection | breast_cancer_primary | 9 | 2.46 (1.27-4.78) | 1.07 | Ineligible |
| SG | Rhinorrhoea | breast_cancer_primary | 9 | 0.76 (0.39-1.46) | 0.47 | Ineligible |
| SG | Rhinorrhoea | breast_cancer_primary | 9 | 0.76 (0.39-1.46) | 0.47 | Ineligible |
| SG | Sleep disorder | breast_cancer_primary | 9 | 0.81 (0.42-1.56) | 0.50 | Ineligible |
| SG | Sleep disorder | breast_cancer_primary | 9 | 0.81 (0.42-1.56) | 0.50 | Ineligible |
| SG | Unevaluable event | breast_cancer_primary | 9 | 1.92 (0.99-3.72) | 0.92 | Ineligible |
| SG | Unevaluable event | breast_cancer_primary | 9 | 1.92 (0.99-3.72) | 0.92 | Ineligible |
| SG | Vision blurred | breast_cancer_primary | 9 | 0.57 (0.30-1.10) | 0.38 | Ineligible |
| SG | Vision blurred | breast_cancer_primary | 9 | 0.57 (0.30-1.10) | 0.38 | Ineligible |
| SG | White blood cell count abnormal | breast_cancer_primary | 9 | 1.61 (0.83-3.12) | 0.82 | Ineligible |
| SG | White blood cell count abnormal | breast_cancer_primary | 9 | 1.61 (0.83-3.12) | 0.82 | Ineligible |
| SG | Aplasia | breast_cancer_primary | 8 | 13.52 (6.41-28.51) | 5.93 | Ineligible |
| SG | Aplasia | breast_cancer_primary | 8 | 13.52 (6.41-28.51) | 5.93 | Ineligible |
| SG | Atrial fibrillation | breast_cancer_primary | 8 | 0.56 (0.28-1.13) | 0.37 | Ineligible |
| SG | Atrial fibrillation | breast_cancer_primary | 8 | 0.56 (0.28-1.13) | 0.37 | Ineligible |
| SG | Bone marrow failure | breast_cancer_primary | 8 | 0.39 (0.19-0.78) | 0.27 | Ineligible |
| SG | Bone marrow failure | breast_cancer_primary | 8 | 0.39 (0.19-0.78) | 0.27 | Ineligible |
| SG | Breast cancer recurrent | breast_cancer_primary | 8 | 0.54 (0.27-1.08) | 0.35 | Ineligible |
| SG | Breast cancer recurrent | breast_cancer_primary | 8 | 0.54 (0.27-1.08) | 0.35 | Ineligible |
| SG | Cholinergic syndrome | breast_cancer_primary | 8 | 689.70 (86.23-5516.25) | 22.95 | Ineligible |
| SG | Cholinergic syndrome | breast_cancer_primary | 8 | 689.70 (86.23-5516.25) | 22.95 | Ineligible |
| SG | Confusional state | breast_cancer_primary | 8 | 0.48 (0.24-0.96) | 0.32 | Ineligible |
| SG | Confusional state | breast_cancer_primary | 8 | 0.48 (0.24-0.96) | 0.32 | Ineligible |
| SG | Dyspnoea exertional | breast_cancer_primary | 8 | 0.74 (0.37-1.48) | 0.45 | Ineligible |
| SG | Dyspnoea exertional | breast_cancer_primary | 8 | 0.74 (0.37-1.48) | 0.45 | Ineligible |
| SG | Epistaxis | breast_cancer_primary | 8 | 0.25 (0.13-0.51) | 0.18 | Ineligible |
| SG | Epistaxis | breast_cancer_primary | 8 | 0.25 (0.13-0.51) | 0.18 | Ineligible |
| SG | Hot flush | breast_cancer_primary | 8 | 0.21 (0.10-0.41) | 0.15 | Ineligible |
| SG | Hot flush | breast_cancer_primary | 8 | 0.21 (0.10-0.41) | 0.15 | Ineligible |
| SG | Hypoxia | breast_cancer_primary | 8 | 1.03 (0.51-2.06) | 0.58 | Ineligible |
| SG | Hypoxia | breast_cancer_primary | 8 | 1.03 (0.51-2.06) | 0.58 | Ineligible |
| SG | Intestinal obstruction | breast_cancer_primary | 8 | 1.94 (0.96-3.91) | 0.89 | Ineligible |
| SG | Intestinal obstruction | breast_cancer_primary | 8 | 1.94 (0.96-3.91) | 0.89 | Ineligible |
| SG | Liver function test increased | breast_cancer_primary | 8 | 0.80 (0.40-1.61) | 0.48 | Ineligible |
| SG | Liver function test increased | breast_cancer_primary | 8 | 0.80 (0.40-1.61) | 0.48 | Ineligible |
| SG | Lower respiratory tract infection | breast_cancer_primary | 8 | 1.40 (0.70-2.82) | 0.72 | Ineligible |
| SG | Lower respiratory tract infection | breast_cancer_primary | 8 | 1.40 (0.70-2.82) | 0.72 | Ineligible |
| SG | Lymphocyte count decreased | breast_cancer_primary | 8 | 1.04 (0.52-2.10) | 0.59 | Ineligible |
| SG | Lymphocyte count decreased | breast_cancer_primary | 8 | 1.04 (0.52-2.10) | 0.59 | Ineligible |
| SG | Metastases to spine | breast_cancer_primary | 8 | 0.96 (0.48-1.92) | 0.55 | Ineligible |
| SG | Metastases to spine | breast_cancer_primary | 8 | 0.96 (0.48-1.92) | 0.55 | Ineligible |
| SG | Neoplasm | breast_cancer_primary | 8 | 1.28 (0.64-2.58) | 0.68 | Ineligible |
| SG | Neoplasm | breast_cancer_primary | 8 | 1.28 (0.64-2.58) | 0.68 | Ineligible |
| SG | Nervous system disorder | breast_cancer_primary | 8 | 2.14 (1.06-4.32) | 0.94 | Ineligible |
| SG | Nervous system disorder | breast_cancer_primary | 8 | 2.14 (1.06-4.32) | 0.94 | Ineligible |
| SG | Peripheral swelling | breast_cancer_primary | 8 | 0.25 (0.12-0.50) | 0.18 | Ineligible |
| SG | Peripheral swelling | breast_cancer_primary | 8 | 0.25 (0.12-0.50) | 0.18 | Ineligible |
| SG | Product administration error | breast_cancer_primary | 8 | 6.76 (3.29-13.89) | 2.22 | Ineligible |
| SG | Product administration error | breast_cancer_primary | 8 | 6.76 (3.29-13.89) | 2.22 | Ineligible |
| SG | Rash pruritic | breast_cancer_primary | 8 | 0.94 (0.47-1.90) | 0.55 | Ineligible |
| SG | Rash pruritic | breast_cancer_primary | 8 | 0.94 (0.47-1.90) | 0.55 | Ineligible |
| SG | Swelling | breast_cancer_primary | 8 | 0.69 (0.34-1.39) | 0.43 | Ineligible |
| SG | Swelling | breast_cancer_primary | 8 | 0.69 (0.34-1.39) | 0.43 | Ineligible |
| SG | Therapy non-responder | breast_cancer_primary | 8 | 1.92 (0.95-3.88) | 0.88 | Ineligible |
| SG | Therapy non-responder | breast_cancer_primary | 8 | 1.92 (0.95-3.88) | 0.88 | Ineligible |
| SG | Anaphylactic reaction | breast_cancer_primary | 7 | 1.10 (0.52-2.33) | 0.59 | Ineligible |
| SG | Anaphylactic reaction | breast_cancer_primary | 7 | 1.10 (0.52-2.33) | 0.59 | Ineligible |
| SG | Balance disorder | breast_cancer_primary | 7 | 0.67 (0.32-1.40) | 0.41 | Ineligible |
| SG | Balance disorder | breast_cancer_primary | 7 | 0.67 (0.32-1.40) | 0.41 | Ineligible |
| SG | Blood alkaline phosphatase increased | breast_cancer_primary | 7 | 0.67 (0.32-1.41) | 0.41 | Ineligible |
| SG | Blood alkaline phosphatase increased | breast_cancer_primary | 7 | 0.67 (0.32-1.41) | 0.41 | Ineligible |
| SG | Blood glucose increased | breast_cancer_primary | 7 | 0.36 (0.17-0.76) | 0.25 | Ineligible |
| SG | Blood glucose increased | breast_cancer_primary | 7 | 0.36 (0.17-0.76) | 0.25 | Ineligible |
| SG | Circumstance or information capable of leading to medication error | breast_cancer_primary | 7 | 8.15 (3.75-17.71) | 2.51 | Ineligible |
| SG | Circumstance or information capable of leading to medication error | breast_cancer_primary | 7 | 8.15 (3.75-17.71) | 2.51 | Ineligible |
| SG | Clostridium difficile infection | breast_cancer_primary | 7 | 2.09 (0.99-4.43) | 0.88 | Ineligible |
| SG | Clostridium difficile infection | breast_cancer_primary | 7 | 2.09 (0.99-4.43) | 0.88 | Ineligible |
| SG | Cystitis | breast_cancer_primary | 7 | 0.92 (0.44-1.95) | 0.52 | Ineligible |
| SG | Cystitis | breast_cancer_primary | 7 | 0.92 (0.44-1.95) | 0.52 | Ineligible |
| SG | Device related infection | breast_cancer_primary | 7 | 1.54 (0.73-3.25) | 0.73 | Ineligible |
| SG | Device related infection | breast_cancer_primary | 7 | 1.54 (0.73-3.25) | 0.73 | Ineligible |
| SG | Discomfort | breast_cancer_primary | 7 | 0.48 (0.23-1.02) | 0.32 | Ineligible |
| SG | Discomfort | breast_cancer_primary | 7 | 0.48 (0.23-1.02) | 0.32 | Ineligible |
| SG | Hypercalcaemia | breast_cancer_primary | 7 | 1.65 (0.78-3.48) | 0.77 | Ineligible |
| SG | Hypercalcaemia | breast_cancer_primary | 7 | 1.65 (0.78-3.48) | 0.77 | Ineligible |
| SG | Hyperglycaemia | breast_cancer_primary | 7 | 0.31 (0.15-0.65) | 0.22 | Ineligible |
| SG | Hyperglycaemia | breast_cancer_primary | 7 | 0.31 (0.15-0.65) | 0.22 | Ineligible |
| SG | Hypocalcaemia | breast_cancer_primary | 7 | 1.03 (0.49-2.18) | 0.57 | Ineligible |
| SG | Hypocalcaemia | breast_cancer_primary | 7 | 1.03 (0.49-2.18) | 0.57 | Ineligible |
| SG | Inflammation | breast_cancer_primary | 7 | 0.74 (0.35-1.56) | 0.44 | Ineligible |
| SG | Inflammation | breast_cancer_primary | 7 | 0.74 (0.35-1.56) | 0.44 | Ineligible |
| SG | Lymphadenopathy | breast_cancer_primary | 7 | 0.59 (0.28-1.24) | 0.37 | Ineligible |
| SG | Lymphadenopathy | breast_cancer_primary | 7 | 0.59 (0.28-1.24) | 0.37 | Ineligible |
| SG | Lymphoedema | breast_cancer_primary | 7 | 0.60 (0.28-1.26) | 0.38 | Ineligible |
| SG | Lymphoedema | breast_cancer_primary | 7 | 0.60 (0.28-1.26) | 0.38 | Ineligible |
| SG | Muscle spasms | breast_cancer_primary | 7 | 0.27 (0.13-0.57) | 0.19 | Ineligible |
| SG | Muscle spasms | breast_cancer_primary | 7 | 0.27 (0.13-0.57) | 0.19 | Ineligible |
| SG | Muscular weakness | breast_cancer_primary | 7 | 0.46 (0.22-0.97) | 0.30 | Ineligible |
| SG | Muscular weakness | breast_cancer_primary | 7 | 0.46 (0.22-0.97) | 0.30 | Ineligible |
| SG | Respiratory distress | breast_cancer_primary | 7 | 1.93 (0.91-4.08) | 0.84 | Ineligible |
| SG | Respiratory distress | breast_cancer_primary | 7 | 1.93 (0.91-4.08) | 0.84 | Ineligible |
| SG | Skin lesion | breast_cancer_primary | 7 | 1.08 (0.51-2.27) | 0.58 | Ineligible |
| SG | Skin lesion | breast_cancer_primary | 7 | 1.08 (0.51-2.27) | 0.58 | Ineligible |
| SG | Stress | breast_cancer_primary | 7 | 0.74 (0.35-1.56) | 0.44 | Ineligible |
| SG | Stress | breast_cancer_primary | 7 | 0.74 (0.35-1.56) | 0.44 | Ineligible |
| SG | Surgery | breast_cancer_primary | 7 | 1.42 (0.67-3.00) | 0.70 | Ineligible |
| SG | Surgery | breast_cancer_primary | 7 | 1.42 (0.67-3.00) | 0.70 | Ineligible |
| SG | Therapy change | breast_cancer_primary | 7 | 1.90 (0.90-4.02) | 0.83 | Ineligible |
| SG | Therapy change | breast_cancer_primary | 7 | 1.90 (0.90-4.02) | 0.83 | Ineligible |
| SG | Throat irritation | breast_cancer_primary | 7 | 1.17 (0.56-2.48) | 0.62 | Ineligible |
| SG | Throat irritation | breast_cancer_primary | 7 | 1.17 (0.56-2.48) | 0.62 | Ineligible |
| SG | Transaminases increased | breast_cancer_primary | 7 | 0.57 (0.27-1.20) | 0.36 | Ineligible |
| SG | Transaminases increased | breast_cancer_primary | 7 | 0.57 (0.27-1.20) | 0.36 | Ineligible |
| SG | Transfusion | breast_cancer_primary | 7 | 6.63 (3.07-14.31) | 1.91 | Ineligible |
| SG | Transfusion | breast_cancer_primary | 7 | 6.63 (3.07-14.31) | 1.91 | Ineligible |
| SG | Tremor | breast_cancer_primary | 7 | 0.43 (0.21-0.91) | 0.29 | Ineligible |
| SG | Tremor | breast_cancer_primary | 7 | 0.43 (0.21-0.91) | 0.29 | Ineligible |
| SG | Bacteraemia | breast_cancer_primary | 6 | 3.08 (1.36-6.95) | 1.00 | Ineligible |
| SG | Bacteraemia | breast_cancer_primary | 6 | 3.08 (1.36-6.95) | 1.00 | Ineligible |
| SG | Blood creatinine increased | breast_cancer_primary | 6 | 0.28 (0.12-0.62) | 0.19 | Ineligible |
| SG | Blood creatinine increased | breast_cancer_primary | 6 | 0.28 (0.12-0.62) | 0.19 | Ineligible |
| SG | Blood disorder | breast_cancer_primary | 6 | 3.02 (1.34-6.82) | 0.99 | Ineligible |
| SG | Blood disorder | breast_cancer_primary | 6 | 3.02 (1.34-6.82) | 0.99 | Ineligible |
| SG | Blood magnesium decreased | breast_cancer_primary | 6 | 1.72 (0.77-3.86) | 0.74 | Ineligible |
| SG | Blood magnesium decreased | breast_cancer_primary | 6 | 1.72 (0.77-3.86) | 0.74 | Ineligible |
| SG | Blood pressure decreased | breast_cancer_primary | 6 | 0.75 (0.34-1.68) | 0.44 | Ineligible |
| SG | Blood pressure decreased | breast_cancer_primary | 6 | 0.75 (0.34-1.68) | 0.44 | Ineligible |
| SG | C-reactive protein increased | breast_cancer_primary | 6 | 0.70 (0.31-1.56) | 0.41 | Ineligible |
| SG | C-reactive protein increased | breast_cancer_primary | 6 | 0.70 (0.31-1.56) | 0.41 | Ineligible |
| SG | Candida infection | breast_cancer_primary | 6 | 1.57 (0.70-3.53) | 0.71 | Ineligible |
| SG | Candida infection | breast_cancer_primary | 6 | 1.57 (0.70-3.53) | 0.71 | Ineligible |
| SG | Cerebral haemorrhage | breast_cancer_primary | 6 | 1.61 (0.72-3.61) | 0.72 | Ineligible |
| SG | Cerebral haemorrhage | breast_cancer_primary | 6 | 1.61 (0.72-3.61) | 0.72 | Ineligible |
| SG | Depressed mood | breast_cancer_primary | 6 | 0.54 (0.24-1.21) | 0.34 | Ineligible |
| SG | Depressed mood | breast_cancer_primary | 6 | 0.54 (0.24-1.21) | 0.34 | Ineligible |
| SG | Diarrhoea haemorrhagic | breast_cancer_primary | 6 | 4.31 (1.90-9.78) | 1.20 | Ineligible |
| SG | Diarrhoea haemorrhagic | breast_cancer_primary | 6 | 4.31 (1.90-9.78) | 1.20 | Ineligible |
| SG | Drug intolerance | breast_cancer_primary | 6 | 0.30 (0.13-0.66) | 0.21 | Ineligible |
| SG | Drug intolerance | breast_cancer_primary | 6 | 0.30 (0.13-0.66) | 0.21 | Ineligible |
| SG | Dysgeusia | breast_cancer_primary | 6 | 0.32 (0.14-0.71) | 0.22 | Ineligible |
| SG | Dysgeusia | breast_cancer_primary | 6 | 0.32 (0.14-0.71) | 0.22 | Ineligible |
| SG | Dysphonia | breast_cancer_primary | 6 | 0.58 (0.26-1.31) | 0.36 | Ineligible |
| SG | Dysphonia | breast_cancer_primary | 6 | 0.58 (0.26-1.31) | 0.36 | Ineligible |
| SG | Epilepsy | breast_cancer_primary | 6 | 1.58 (0.71-3.55) | 0.71 | Ineligible |
| SG | Epilepsy | breast_cancer_primary | 6 | 1.58 (0.71-3.55) | 0.71 | Ineligible |
| SG | Faeces soft | breast_cancer_primary | 6 | 2.73 (1.21-6.17) | 0.95 | Ineligible |
| SG | Faeces soft | breast_cancer_primary | 6 | 2.73 (1.21-6.17) | 0.95 | Ineligible |
| SG | Femur fracture | breast_cancer_primary | 6 | 1.08 (0.48-2.41) | 0.56 | Ineligible |
| SG | Femur fracture | breast_cancer_primary | 6 | 1.08 (0.48-2.41) | 0.56 | Ineligible |
| SG | Gait inability | breast_cancer_primary | 6 | 0.91 (0.41-2.04) | 0.50 | Ineligible |
| SG | Gait inability | breast_cancer_primary | 6 | 0.91 (0.41-2.04) | 0.50 | Ineligible |
| SG | Granulocyte count decreased | breast_cancer_primary | 6 | 2.37 (1.05-5.34) | 0.88 | Ineligible |
| SG | Granulocyte count decreased | breast_cancer_primary | 6 | 2.37 (1.05-5.34) | 0.88 | Ineligible |
| SG | Haemoglobin abnormal | breast_cancer_primary | 6 | 2.58 (1.15-5.82) | 0.92 | Ineligible |
| SG | Haemoglobin abnormal | breast_cancer_primary | 6 | 2.58 (1.15-5.82) | 0.92 | Ineligible |
| SG | Hyperbilirubinaemia | breast_cancer_primary | 6 | 1.30 (0.58-2.92) | 0.63 | Ineligible |
| SG | Hyperbilirubinaemia | breast_cancer_primary | 6 | 1.30 (0.58-2.92) | 0.63 | Ineligible |
| SG | Hyperhidrosis | breast_cancer_primary | 6 | 0.43 (0.19-0.96) | 0.28 | Ineligible |
| SG | Hyperhidrosis | breast_cancer_primary | 6 | 0.43 (0.19-0.96) | 0.28 | Ineligible |
| SG | Hypersomnia | breast_cancer_primary | 6 | 1.43 (0.64-3.22) | 0.67 | Ineligible |
| SG | Hypersomnia | breast_cancer_primary | 6 | 1.43 (0.64-3.22) | 0.67 | Ineligible |
| SG | Hypothyroidism | breast_cancer_primary | 6 | 0.71 (0.32-1.58) | 0.42 | Ineligible |
| SG | Hypothyroidism | breast_cancer_primary | 6 | 0.71 (0.32-1.58) | 0.42 | Ineligible |
| SG | Influenza | breast_cancer_primary | 6 | 0.41 (0.18-0.92) | 0.27 | Ineligible |
| SG | Influenza | breast_cancer_primary | 6 | 0.41 (0.18-0.92) | 0.27 | Ineligible |
| SG | Lethargy | breast_cancer_primary | 6 | 0.89 (0.40-2.00) | 0.49 | Ineligible |
| SG | Lethargy | breast_cancer_primary | 6 | 0.89 (0.40-2.00) | 0.49 | Ineligible |
| SG | Metastases to pleura | breast_cancer_primary | 6 | 1.67 (0.74-3.74) | 0.73 | Ineligible |
| SG | Metastases to pleura | breast_cancer_primary | 6 | 1.67 (0.74-3.74) | 0.73 | Ineligible |
| SG | Metastases to skin | breast_cancer_primary | 6 | 1.39 (0.62-3.11) | 0.66 | Ineligible |
| SG | Metastases to skin | breast_cancer_primary | 6 | 1.39 (0.62-3.11) | 0.66 | Ineligible |
| SG | Musculoskeletal chest pain | breast_cancer_primary | 6 | 0.75 (0.34-1.69) | 0.44 | Ineligible |
| SG | Musculoskeletal chest pain | breast_cancer_primary | 6 | 0.75 (0.34-1.69) | 0.44 | Ineligible |
| SG | Pulmonary mass | breast_cancer_primary | 6 | 0.74 (0.33-1.65) | 0.43 | Ineligible |
| SG | Pulmonary mass | breast_cancer_primary | 6 | 0.74 (0.33-1.65) | 0.43 | Ineligible |
| SG | Red cell distribution width increased | breast_cancer_primary | 6 | 1.28 (0.57-2.87) | 0.63 | Ineligible |
| SG | Red cell distribution width increased | breast_cancer_primary | 6 | 1.28 (0.57-2.87) | 0.63 | Ineligible |
| SG | Urticaria | breast_cancer_primary | 6 | 0.44 (0.20-0.98) | 0.29 | Ineligible |
| SG | Urticaria | breast_cancer_primary | 6 | 0.44 (0.20-0.98) | 0.29 | Ineligible |
| SG | Vertigo | breast_cancer_primary | 6 | 0.59 (0.26-1.32) | 0.36 | Ineligible |
| SG | Vertigo | breast_cancer_primary | 6 | 0.59 (0.26-1.32) | 0.36 | Ineligible |
| SG | Viral infection | breast_cancer_primary | 6 | 1.35 (0.60-3.03) | 0.65 | Ineligible |
| SG | Viral infection | breast_cancer_primary | 6 | 1.35 (0.60-3.03) | 0.65 | Ineligible |
| SG | White blood cell count increased | breast_cancer_primary | 6 | 1.06 (0.47-2.37) | 0.55 | Ineligible |
| SG | White blood cell count increased | breast_cancer_primary | 6 | 1.06 (0.47-2.37) | 0.55 | Ineligible |
| SG | Accidental overdose | breast_cancer_primary | 5 | 4.30 (1.75-10.58) | 1.07 | Ineligible |
| SG | Accidental overdose | breast_cancer_primary | 5 | 4.30 (1.75-10.58) | 1.07 | Ineligible |
| SG | Acute respiratory failure | breast_cancer_primary | 5 | 1.48 (0.61-3.59) | 0.64 | Ineligible |
| SG | Acute respiratory failure | breast_cancer_primary | 5 | 1.48 (0.61-3.59) | 0.64 | Ineligible |
| SG | Anaphylactic shock | breast_cancer_primary | 5 | 1.32 (0.55-3.20) | 0.60 | Ineligible |
| SG | Anaphylactic shock | breast_cancer_primary | 5 | 1.32 (0.55-3.20) | 0.60 | Ineligible |
| SG | Blood lactate dehydrogenase increased | breast_cancer_primary | 5 | 0.83 (0.34-2.01) | 0.45 | Ineligible |
| SG | Blood lactate dehydrogenase increased | breast_cancer_primary | 5 | 0.83 (0.34-2.01) | 0.45 | Ineligible |
| SG | Brain oedema | breast_cancer_primary | 5 | 1.43 (0.59-3.47) | 0.63 | Ineligible |
| SG | Brain oedema | breast_cancer_primary | 5 | 1.43 (0.59-3.47) | 0.63 | Ineligible |
| SG | Breast mass | breast_cancer_primary | 5 | 1.09 (0.45-2.65) | 0.54 | Ineligible |
| SG | Breast mass | breast_cancer_primary | 5 | 1.09 (0.45-2.65) | 0.54 | Ineligible |
| SG | Bronchitis | breast_cancer_primary | 5 | 0.60 (0.25-1.46) | 0.36 | Ineligible |
| SG | Bronchitis | breast_cancer_primary | 5 | 0.60 (0.25-1.46) | 0.36 | Ineligible |
| SG | Cellulitis | breast_cancer_primary | 5 | 0.40 (0.16-0.96) | 0.26 | Ineligible |
| SG | Cellulitis | breast_cancer_primary | 5 | 0.40 (0.16-0.96) | 0.26 | Ineligible |
| SG | Central nervous system lesion | breast_cancer_primary | 5 | 1.70 (0.70-4.12) | 0.69 | Ineligible |
| SG | Central nervous system lesion | breast_cancer_primary | 5 | 1.70 (0.70-4.12) | 0.69 | Ineligible |
| SG | Clostridium difficile colitis | breast_cancer_primary | 5 | 2.67 (1.10-6.51) | 0.86 | Ineligible |
| SG | Clostridium difficile colitis | breast_cancer_primary | 5 | 2.67 (1.10-6.51) | 0.86 | Ineligible |
| SG | Device issue | breast_cancer_primary | 5 | 3.74 (1.53-9.17) | 1.00 | Ineligible |
| SG | Device issue | breast_cancer_primary | 5 | 3.74 (1.53-9.17) | 1.00 | Ineligible |
| SG | Device malfunction | breast_cancer_primary | 5 | 2.69 (1.10-6.56) | 0.86 | Ineligible |
| SG | Device malfunction | breast_cancer_primary | 5 | 2.69 (1.10-6.56) | 0.86 | Ineligible |
| SG | Device occlusion | breast_cancer_primary | 5 | 3.34 (1.36-8.16) | 0.95 | Ineligible |
| SG | Device occlusion | breast_cancer_primary | 5 | 3.34 (1.36-8.16) | 0.95 | Ineligible |
| SG | Disseminated intravascular coagulation | breast_cancer_primary | 5 | 1.92 (0.79-4.66) | 0.74 | Ineligible |
| SG | Disseminated intravascular coagulation | breast_cancer_primary | 5 | 1.92 (0.79-4.66) | 0.74 | Ineligible |
| SG | Diverticulitis | breast_cancer_primary | 5 | 1.21 (0.50-2.93) | 0.57 | Ineligible |
| SG | Diverticulitis | breast_cancer_primary | 5 | 1.21 (0.50-2.93) | 0.57 | Ineligible |
| SG | Dry eye | breast_cancer_primary | 5 | 0.46 (0.19-1.11) | 0.29 | Ineligible |
| SG | Dry eye | breast_cancer_primary | 5 | 0.46 (0.19-1.11) | 0.29 | Ineligible |
| SG | Eastern Cooperative Oncology Group performance status worsened | breast_cancer_primary | 5 | 2.23 (0.92-5.42) | 0.79 | Ineligible |
| SG | Eastern Cooperative Oncology Group performance status worsened | breast_cancer_primary | 5 | 2.23 (0.92-5.42) | 0.79 | Ineligible |
| SG | Eating disorder | breast_cancer_primary | 5 | 0.58 (0.24-1.40) | 0.35 | Ineligible |
| SG | Eating disorder | breast_cancer_primary | 5 | 0.58 (0.24-1.40) | 0.35 | Ineligible |
| SG | Feeling hot | breast_cancer_primary | 5 | 0.50 (0.21-1.21) | 0.31 | Ineligible |
| SG | Feeling hot | breast_cancer_primary | 5 | 0.50 (0.21-1.21) | 0.31 | Ineligible |
| SG | Full blood count abnormal | breast_cancer_primary | 5 | 0.55 (0.23-1.33) | 0.34 | Ineligible |
| SG | Full blood count abnormal | breast_cancer_primary | 5 | 0.55 (0.23-1.33) | 0.34 | Ineligible |
| SG | Haemorrhoids | breast_cancer_primary | 5 | 1.01 (0.42-2.44) | 0.51 | Ineligible |
| SG | Haemorrhoids | breast_cancer_primary | 5 | 1.01 (0.42-2.44) | 0.51 | Ineligible |
| SG | Hair growth abnormal | breast_cancer_primary | 5 | 1.54 (0.64-3.74) | 0.66 | Ineligible |
| SG | Hair growth abnormal | breast_cancer_primary | 5 | 1.54 (0.64-3.74) | 0.66 | Ineligible |
| SG | Hepatic lesion | breast_cancer_primary | 5 | 0.96 (0.40-2.31) | 0.50 | Ineligible |
| SG | Hepatic lesion | breast_cancer_primary | 5 | 0.96 (0.40-2.31) | 0.50 | Ineligible |
| SG | Hepatic neoplasm | breast_cancer_primary | 5 | 3.42 (1.40-8.36) | 0.96 | Ineligible |
| SG | Hepatic neoplasm | breast_cancer_primary | 5 | 3.42 (1.40-8.36) | 0.96 | Ineligible |
| SG | Hyperkalaemia | breast_cancer_primary | 5 | 1.54 (0.64-3.74) | 0.66 | Ineligible |
| SG | Hyperkalaemia | breast_cancer_primary | 5 | 1.54 (0.64-3.74) | 0.66 | Ineligible |
| SG | Hypertransaminasaemia | breast_cancer_primary | 5 | 0.89 (0.37-2.15) | 0.47 | Ineligible |
| SG | Hypertransaminasaemia | breast_cancer_primary | 5 | 0.89 (0.37-2.15) | 0.47 | Ineligible |
| SG | Large intestine perforation | breast_cancer_primary | 5 | 3.36 (1.38-8.22) | 0.95 | Ineligible |
| SG | Large intestine perforation | breast_cancer_primary | 5 | 3.36 (1.38-8.22) | 0.95 | Ineligible |
| SG | Loss of personal independence in daily activities | breast_cancer_primary | 5 | 1.21 (0.50-2.93) | 0.57 | Ineligible |
| SG | Loss of personal independence in daily activities | breast_cancer_primary | 5 | 1.21 (0.50-2.93) | 0.57 | Ineligible |
| SG | Malignant pleural effusion | breast_cancer_primary | 5 | 1.77 (0.73-4.30) | 0.71 | Ineligible |
| SG | Malignant pleural effusion | breast_cancer_primary | 5 | 1.77 (0.73-4.30) | 0.71 | Ineligible |
| SG | Mass | breast_cancer_primary | 5 | 1.29 (0.53-3.13) | 0.60 | Ineligible |
| SG | Mass | breast_cancer_primary | 5 | 1.29 (0.53-3.13) | 0.60 | Ineligible |
| SG | Nasal congestion | breast_cancer_primary | 5 | 0.93 (0.38-2.24) | 0.49 | Ineligible |
| SG | Nasal congestion | breast_cancer_primary | 5 | 0.93 (0.38-2.24) | 0.49 | Ineligible |
| SG | Neoplasm malignant | breast_cancer_primary | 5 | 0.65 (0.27-1.58) | 0.38 | Ineligible |
| SG | Neoplasm malignant | breast_cancer_primary | 5 | 0.65 (0.27-1.58) | 0.38 | Ineligible |
| SG | Neuralgia | breast_cancer_primary | 5 | 0.91 (0.38-2.21) | 0.48 | Ineligible |
| SG | Neuralgia | breast_cancer_primary | 5 | 0.91 (0.38-2.21) | 0.48 | Ineligible |
| SG | Neurotoxicity | breast_cancer_primary | 5 | 0.85 (0.35-2.05) | 0.46 | Ineligible |
| SG | Neurotoxicity | breast_cancer_primary | 5 | 0.85 (0.35-2.05) | 0.46 | Ineligible |
| SG | Nutritional condition abnormal | breast_cancer_primary | 5 | 19.57 (7.41-51.72) | 5.96 | Ineligible |
| SG | Nutritional condition abnormal | breast_cancer_primary | 5 | 19.57 (7.41-51.72) | 5.96 | Ineligible |
| SG | Oedema peripheral | breast_cancer_primary | 5 | 0.21 (0.09-0.51) | 0.15 | Ineligible |
| SG | Oedema peripheral | breast_cancer_primary | 5 | 0.21 (0.09-0.51) | 0.15 | Ineligible |
| SG | Overdose | breast_cancer_primary | 5 | 0.98 (0.41-2.37) | 0.50 | Ineligible |
| SG | Overdose | breast_cancer_primary | 5 | 0.98 (0.41-2.37) | 0.50 | Ineligible |
| SG | Palliative care | breast_cancer_primary | 5 | 39.15 (13.59-112.74) | 9.15 | Ineligible |
| SG | Palliative care | breast_cancer_primary | 5 | 39.15 (13.59-112.74) | 9.15 | Ineligible |
| SG | Pericardial effusion | breast_cancer_primary | 5 | 0.56 (0.23-1.36) | 0.34 | Ineligible |
| SG | Pericardial effusion | breast_cancer_primary | 5 | 0.56 (0.23-1.36) | 0.34 | Ineligible |
| SG | Product dispensing error | breast_cancer_primary | 5 | 1.69 (0.70-4.11) | 0.69 | Ineligible |
| SG | Product dispensing error | breast_cancer_primary | 5 | 1.69 (0.70-4.11) | 0.69 | Ineligible |
| SG | Radiotherapy | breast_cancer_primary | 5 | 2.93 (1.20-7.14) | 0.89 | Ineligible |
| SG | Radiotherapy | breast_cancer_primary | 5 | 2.93 (1.20-7.14) | 0.89 | Ineligible |
| SG | Renal impairment | breast_cancer_primary | 5 | 0.29 (0.12-0.70) | 0.20 | Ineligible |
| SG | Renal impairment | breast_cancer_primary | 5 | 0.29 (0.12-0.70) | 0.20 | Ineligible |
| SG | Retching | breast_cancer_primary | 5 | 1.28 (0.53-3.09) | 0.59 | Ineligible |
| SG | Retching | breast_cancer_primary | 5 | 1.28 (0.53-3.09) | 0.59 | Ineligible |
| SG | Sinus tachycardia | breast_cancer_primary | 5 | 1.69 (0.70-4.11) | 0.69 | Ineligible |
| SG | Sinus tachycardia | breast_cancer_primary | 5 | 1.69 (0.70-4.11) | 0.69 | Ineligible |
| SG | Sinusitis | breast_cancer_primary | 5 | 0.59 (0.25-1.43) | 0.36 | Ineligible |
| SG | Sinusitis | breast_cancer_primary | 5 | 0.59 (0.25-1.43) | 0.36 | Ineligible |
| SG | Therapy interrupted | breast_cancer_primary | 5 | 0.53 (0.22-1.28) | 0.33 | Ineligible |
| SG | Therapy interrupted | breast_cancer_primary | 5 | 0.53 (0.22-1.28) | 0.33 | Ineligible |
| SG | Upper respiratory tract infection | breast_cancer_primary | 5 | 0.76 (0.32-1.84) | 0.43 | Ineligible |
| SG | Upper respiratory tract infection | breast_cancer_primary | 5 | 0.76 (0.32-1.84) | 0.43 | Ineligible |
| SG | Vein collapse | breast_cancer_primary | 5 | 35.89 (12.63-101.92) | 8.78 | Ineligible |
| SG | Vein collapse | breast_cancer_primary | 5 | 35.89 (12.63-101.92) | 8.78 | Ineligible |
| SG | Visual impairment | breast_cancer_primary | 5 | 0.28 (0.12-0.68) | 0.20 | Ineligible |
| SG | Visual impairment | breast_cancer_primary | 5 | 0.28 (0.12-0.68) | 0.20 | Ineligible |
| SG | Wound | breast_cancer_primary | 5 | 0.95 (0.39-2.29) | 0.49 | Ineligible |
| SG | Wound | breast_cancer_primary | 5 | 0.95 (0.39-2.29) | 0.49 | Ineligible |
| SG | Bedridden | breast_cancer_primary | 4 | 3.34 (1.23-9.08) | 0.84 | Ineligible |
| SG | Bedridden | breast_cancer_primary | 4 | 3.34 (1.23-9.08) | 0.84 | Ineligible |
| SG | Blood calcium decreased | breast_cancer_primary | 4 | 0.86 (0.32-2.31) | 0.44 | Ineligible |
| SG | Blood calcium decreased | breast_cancer_primary | 4 | 0.86 (0.32-2.31) | 0.44 | Ineligible |
| SG | Body temperature increased | breast_cancer_primary | 4 | 1.05 (0.39-2.81) | 0.50 | Ineligible |
| SG | Body temperature increased | breast_cancer_primary | 4 | 1.05 (0.39-2.81) | 0.50 | Ineligible |
| SG | Brain neoplasm malignant | breast_cancer_primary | 4 | 4.25 (1.56-11.61) | 0.93 | Ineligible |
| SG | Brain neoplasm malignant | breast_cancer_primary | 4 | 4.25 (1.56-11.61) | 0.93 | Ineligible |
| SG | Cardio-respiratory arrest | breast_cancer_primary | 4 | 1.07 (0.40-2.88) | 0.50 | Ineligible |
| SG | Cardio-respiratory arrest | breast_cancer_primary | 4 | 1.07 (0.40-2.88) | 0.50 | Ineligible |
| SG | Cataract | breast_cancer_primary | 4 | 0.38 (0.14-1.01) | 0.25 | Ineligible |
| SG | Cataract | breast_cancer_primary | 4 | 0.38 (0.14-1.01) | 0.25 | Ineligible |
| SG | Cerebral disorder | breast_cancer_primary | 4 | 1.76 (0.65-4.73) | 0.65 | Ineligible |
| SG | Cerebral disorder | breast_cancer_primary | 4 | 1.76 (0.65-4.73) | 0.65 | Ineligible |
| SG | Colitis ischaemic | breast_cancer_primary | 4 | 2.73 (1.01-7.40) | 0.78 | Ineligible |
| SG | Colitis ischaemic | breast_cancer_primary | 4 | 2.73 (1.01-7.40) | 0.78 | Ineligible |
| SG | Decreased activity | breast_cancer_primary | 4 | 3.78 (1.39-10.30) | 0.88 | Ineligible |
| SG | Decreased activity | breast_cancer_primary | 4 | 3.78 (1.39-10.30) | 0.88 | Ineligible |
| SG | Deep vein thrombosis | breast_cancer_primary | 4 | 0.37 (0.14-0.99) | 0.24 | Ineligible |
| SG | Deep vein thrombosis | breast_cancer_primary | 4 | 0.37 (0.14-0.99) | 0.24 | Ineligible |
| SG | Dermatitis contact | breast_cancer_primary | 4 | 9.06 (3.23-25.41) | 1.44 | Ineligible |
| SG | Dermatitis contact | breast_cancer_primary | 4 | 9.06 (3.23-25.41) | 1.44 | Ineligible |
| SG | Device infusion issue | breast_cancer_primary | 4 | 38.27 (11.78-124.33) | 7.54 | Ineligible |
| SG | Device infusion issue | breast_cancer_primary | 4 | 38.27 (11.78-124.33) | 7.54 | Ineligible |
| SG | Disease recurrence | breast_cancer_primary | 4 | 0.39 (0.15-1.05) | 0.26 | Ineligible |
| SG | Disease recurrence | breast_cancer_primary | 4 | 0.39 (0.15-1.05) | 0.26 | Ineligible |
| SG | Dose calculation error | breast_cancer_primary | 4 | 172.20 (31.53-940.54) | 11.57 | Ineligible |
| SG | Dose calculation error | breast_cancer_primary | 4 | 172.20 (31.53-940.54) | 11.57 | Ineligible |
| SG | Drug eruption | breast_cancer_primary | 4 | 1.41 (0.52-3.79) | 0.58 | Ineligible |
| SG | Drug eruption | breast_cancer_primary | 4 | 1.41 (0.52-3.79) | 0.58 | Ineligible |
| SG | Eastern Cooperative Oncology Group performance status abnormal | breast_cancer_primary | 4 | 9.57 (3.40-26.89) | 1.51 | Ineligible |
| SG | Eastern Cooperative Oncology Group performance status abnormal | breast_cancer_primary | 4 | 9.57 (3.40-26.89) | 1.51 | Ineligible |
| SG | Enterocolitis infectious | breast_cancer_primary | 4 | 7.65 (2.75-21.29) | 1.27 | Ineligible |
| SG | Enterocolitis infectious | breast_cancer_primary | 4 | 7.65 (2.75-21.29) | 1.27 | Ineligible |
| SG | Escherichia infection | breast_cancer_primary | 4 | 3.44 (1.27-9.36) | 0.85 | Ineligible |
| SG | Escherichia infection | breast_cancer_primary | 4 | 3.44 (1.27-9.36) | 0.85 | Ineligible |
| SG | Escherichia urinary tract infection | breast_cancer_primary | 4 | 4.30 (1.58-11.76) | 0.93 | Ineligible |
| SG | Escherichia urinary tract infection | breast_cancer_primary | 4 | 4.30 (1.58-11.76) | 0.93 | Ineligible |
| SG | Feeding disorder | breast_cancer_primary | 4 | 0.45 (0.17-1.21) | 0.28 | Ineligible |
| SG | Feeding disorder | breast_cancer_primary | 4 | 0.45 (0.17-1.21) | 0.28 | Ineligible |
| SG | Feeling cold | breast_cancer_primary | 4 | 0.63 (0.24-1.69) | 0.36 | Ineligible |
| SG | Feeling cold | breast_cancer_primary | 4 | 0.63 (0.24-1.69) | 0.36 | Ineligible |
| SG | Feeling jittery | breast_cancer_primary | 4 | 5.22 (1.90-14.32) | 1.02 | Ineligible |
| SG | Feeling jittery | breast_cancer_primary | 4 | 5.22 (1.90-14.32) | 1.02 | Ineligible |
| SG | Flatulence | breast_cancer_primary | 4 | 0.52 (0.19-1.39) | 0.31 | Ineligible |
| SG | Flatulence | breast_cancer_primary | 4 | 0.52 (0.19-1.39) | 0.31 | Ineligible |
| SG | Foot fracture | breast_cancer_primary | 4 | 1.93 (0.72-5.21) | 0.67 | Ineligible |
| SG | Foot fracture | breast_cancer_primary | 4 | 1.93 (0.72-5.21) | 0.67 | Ineligible |
| SG | Fracture | breast_cancer_primary | 4 | 0.94 (0.35-2.53) | 0.47 | Ineligible |
| SG | Fracture | breast_cancer_primary | 4 | 0.94 (0.35-2.53) | 0.47 | Ineligible |
| SG | Gastroenteritis | breast_cancer_primary | 4 | 1.35 (0.50-3.64) | 0.57 | Ineligible |
| SG | Gastroenteritis | breast_cancer_primary | 4 | 1.35 (0.50-3.64) | 0.57 | Ineligible |
| SG | Gastrointestinal inflammation | breast_cancer_primary | 4 | 4.47 (1.64-12.23) | 0.95 | Ineligible |
| SG | Gastrointestinal inflammation | breast_cancer_primary | 4 | 4.47 (1.64-12.23) | 0.95 | Ineligible |
| SG | Generalised tonic-clonic seizure | breast_cancer_primary | 4 | 4.00 (1.47-10.92) | 0.90 | Ineligible |
| SG | Generalised tonic-clonic seizure | breast_cancer_primary | 4 | 4.00 (1.47-10.92) | 0.90 | Ineligible |
| SG | Gingival pain | breast_cancer_primary | 4 | 1.18 (0.44-3.16) | 0.53 | Ineligible |
| SG | Gingival pain | breast_cancer_primary | 4 | 1.18 (0.44-3.16) | 0.53 | Ineligible |
| SG | Glossodynia | breast_cancer_primary | 4 | 0.90 (0.33-2.40) | 0.45 | Ineligible |
| SG | Glossodynia | breast_cancer_primary | 4 | 0.90 (0.33-2.40) | 0.45 | Ineligible |
| SG | Haematoma | breast_cancer_primary | 4 | 1.11 (0.41-2.99) | 0.51 | Ineligible |
| SG | Haematoma | breast_cancer_primary | 4 | 1.11 (0.41-2.99) | 0.51 | Ineligible |
| SG | Hallucination | breast_cancer_primary | 4 | 1.42 (0.53-3.81) | 0.58 | Ineligible |
| SG | Hallucination | breast_cancer_primary | 4 | 1.42 (0.53-3.81) | 0.58 | Ineligible |
| SG | Hepatic cirrhosis | breast_cancer_primary | 4 | 0.83 (0.31-2.23) | 0.43 | Ineligible |
| SG | Hepatic cirrhosis | breast_cancer_primary | 4 | 0.83 (0.31-2.23) | 0.43 | Ineligible |
| SG | Hospice care | breast_cancer_primary | 4 | 0.94 (0.35-2.52) | 0.47 | Ineligible |
| SG | Hospice care | breast_cancer_primary | 4 | 0.94 (0.35-2.52) | 0.47 | Ineligible |
| SG | Infusion related hypersensitivity reaction | breast_cancer_primary | 4 | 11.11 (3.92-31.49) | 1.73 | Ineligible |
| SG | Infusion related hypersensitivity reaction | breast_cancer_primary | 4 | 11.11 (3.92-31.49) | 1.73 | Ineligible |
| SG | Intestinal ischaemia | breast_cancer_primary | 4 | 3.59 (1.32-9.76) | 0.86 | Ineligible |
| SG | Intestinal ischaemia | breast_cancer_primary | 4 | 3.59 (1.32-9.76) | 0.86 | Ineligible |
| SG | Localised infection | breast_cancer_primary | 4 | 1.23 (0.46-3.30) | 0.54 | Ineligible |
| SG | Localised infection | breast_cancer_primary | 4 | 1.23 (0.46-3.30) | 0.54 | Ineligible |
| SG | Loss of consciousness | breast_cancer_primary | 4 | 0.34 (0.13-0.90) | 0.22 | Ineligible |
| SG | Loss of consciousness | breast_cancer_primary | 4 | 0.34 (0.13-0.90) | 0.22 | Ineligible |
| SG | Meningitis | breast_cancer_primary | 4 | 5.38 (1.96-14.78) | 1.03 | Ineligible |
| SG | Meningitis | breast_cancer_primary | 4 | 5.38 (1.96-14.78) | 1.03 | Ineligible |
| SG | Metabolic acidosis | breast_cancer_primary | 4 | 1.87 (0.69-5.04) | 0.66 | Ineligible |
| SG | Metabolic acidosis | breast_cancer_primary | 4 | 1.87 (0.69-5.04) | 0.66 | Ineligible |
| SG | Mouth ulceration | breast_cancer_primary | 4 | 0.45 (0.17-1.21) | 0.28 | Ineligible |
| SG | Mouth ulceration | breast_cancer_primary | 4 | 0.45 (0.17-1.21) | 0.28 | Ineligible |
| SG | Neck pain | breast_cancer_primary | 4 | 0.50 (0.19-1.33) | 0.30 | Ineligible |
| SG | Neck pain | breast_cancer_primary | 4 | 0.50 (0.19-1.33) | 0.30 | Ineligible |
| SG | Oesophageal stenosis | breast_cancer_primary | 4 | 4.72 (1.72-12.91) | 0.97 | Ineligible |
| SG | Oesophageal stenosis | breast_cancer_primary | 4 | 4.72 (1.72-12.91) | 0.97 | Ineligible |
| SG | Pain of skin | breast_cancer_primary | 4 | 1.14 (0.43-3.07) | 0.52 | Ineligible |
| SG | Pain of skin | breast_cancer_primary | 4 | 1.14 (0.43-3.07) | 0.52 | Ineligible |
| SG | Palpitations | breast_cancer_primary | 4 | 0.27 (0.10-0.72) | 0.19 | Ineligible |
| SG | Palpitations | breast_cancer_primary | 4 | 0.27 (0.10-0.72) | 0.19 | Ineligible |
| SG | Prerenal failure | breast_cancer_primary | 4 | 12.30 (4.31-35.09) | 1.92 | Ineligible |
| SG | Prerenal failure | breast_cancer_primary | 4 | 12.30 (4.31-35.09) | 1.92 | Ineligible |
| SG | Presyncope | breast_cancer_primary | 4 | 1.40 (0.52-3.76) | 0.58 | Ineligible |
| SG | Presyncope | breast_cancer_primary | 4 | 1.40 (0.52-3.76) | 0.58 | Ineligible |
| SG | Product use in unapproved indication | breast_cancer_primary | 4 | 0.23 (0.09-0.63) | 0.17 | Ineligible |
| SG | Product use in unapproved indication | breast_cancer_primary | 4 | 0.23 (0.09-0.63) | 0.17 | Ineligible |
| SG | Rash erythematous | breast_cancer_primary | 4 | 0.68 (0.25-1.82) | 0.38 | Ineligible |
| SG | Rash erythematous | breast_cancer_primary | 4 | 0.68 (0.25-1.82) | 0.38 | Ineligible |
| SG | Rash macular | breast_cancer_primary | 4 | 0.98 (0.37-2.63) | 0.48 | Ineligible |
| SG | Rash macular | breast_cancer_primary | 4 | 0.98 (0.37-2.63) | 0.48 | Ineligible |
| SG | Shock | breast_cancer_primary | 4 | 1.94 (0.72-5.24) | 0.68 | Ineligible |
| SG | Shock | breast_cancer_primary | 4 | 1.94 (0.72-5.24) | 0.68 | Ineligible |
| SG | Skin infection | breast_cancer_primary | 4 | 1.82 (0.68-4.91) | 0.66 | Ineligible |
| SG | Skin infection | breast_cancer_primary | 4 | 1.82 (0.68-4.91) | 0.66 | Ineligible |
| SG | Skin ulcer | breast_cancer_primary | 4 | 0.84 (0.31-2.24) | 0.43 | Ineligible |
| SG | Skin ulcer | breast_cancer_primary | 4 | 0.84 (0.31-2.24) | 0.43 | Ineligible |
| SG | Speech disorder | breast_cancer_primary | 4 | 0.55 (0.21-1.48) | 0.33 | Ineligible |
| SG | Speech disorder | breast_cancer_primary | 4 | 0.55 (0.21-1.48) | 0.33 | Ineligible |
| SG | Staphylococcal sepsis | breast_cancer_primary | 4 | 8.40 (3.01-23.46) | 1.36 | Ineligible |
| SG | Staphylococcal sepsis | breast_cancer_primary | 4 | 8.40 (3.01-23.46) | 1.36 | Ineligible |
| SG | Swelling face | breast_cancer_primary | 4 | 0.58 (0.22-1.55) | 0.34 | Ineligible |
| SG | Swelling face | breast_cancer_primary | 4 | 0.58 (0.22-1.55) | 0.34 | Ineligible |
| SG | Tooth extraction | breast_cancer_primary | 4 | 1.62 (0.60-4.37) | 0.62 | Ineligible |
| SG | Tooth extraction | breast_cancer_primary | 4 | 1.62 (0.60-4.37) | 0.62 | Ineligible |
| SG | Tubulointerstitial nephritis | breast_cancer_primary | 4 | 1.59 (0.59-4.29) | 0.62 | Ineligible |
| SG | Tubulointerstitial nephritis | breast_cancer_primary | 4 | 1.59 (0.59-4.29) | 0.62 | Ineligible |
| SG | Tumour marker increased | breast_cancer_primary | 4 | 0.24 (0.09-0.63) | 0.17 | Ineligible |
| SG | Tumour marker increased | breast_cancer_primary | 4 | 0.24 (0.09-0.63) | 0.17 | Ineligible |
| SG | Wound infection | breast_cancer_primary | 4 | 2.67 (0.99-7.22) | 0.77 | Ineligible |
| SG | Wound infection | breast_cancer_primary | 4 | 2.67 (0.99-7.22) | 0.77 | Ineligible |
| SG | Abscess | breast_cancer_primary | 3 | 1.25 (0.40-3.92) | 0.50 | Ineligible |
| SG | Abscess | breast_cancer_primary | 3 | 1.25 (0.40-3.92) | 0.50 | Ineligible |
| SG | Acne | breast_cancer_primary | 3 | 0.54 (0.17-1.67) | 0.31 | Ineligible |
| SG | Acne | breast_cancer_primary | 3 | 0.54 (0.17-1.67) | 0.31 | Ineligible |
| SG | Aphasia | breast_cancer_primary | 3 | 0.83 (0.27-2.60) | 0.41 | Ineligible |
| SG | Aphasia | breast_cancer_primary | 3 | 0.83 (0.27-2.60) | 0.41 | Ineligible |
| SG | Blood creatinine decreased | breast_cancer_primary | 3 | 1.37 (0.44-4.27) | 0.52 | Ineligible |
| SG | Blood creatinine decreased | breast_cancer_primary | 3 | 1.37 (0.44-4.27) | 0.52 | Ineligible |
| SG | Blood potassium decreased | breast_cancer_primary | 3 | 0.35 (0.11-1.07) | 0.23 | Ineligible |
| SG | Blood potassium decreased | breast_cancer_primary | 3 | 0.35 (0.11-1.07) | 0.23 | Ineligible |
| SG | Blood pressure fluctuation | breast_cancer_primary | 3 | 1.39 (0.45-4.37) | 0.53 | Ineligible |
| SG | Blood pressure fluctuation | breast_cancer_primary | 3 | 1.39 (0.45-4.37) | 0.53 | Ineligible |
| SG | Blood urea increased | breast_cancer_primary | 3 | 0.83 (0.27-2.58) | 0.41 | Ineligible |
| SG | Blood urea increased | breast_cancer_primary | 3 | 0.83 (0.27-2.58) | 0.41 | Ineligible |
| SG | Breast discharge | breast_cancer_primary | 3 | 3.31 (1.04-10.49) | 0.72 | Ineligible |
| SG | Breast discharge | breast_cancer_primary | 3 | 3.31 (1.04-10.49) | 0.72 | Ineligible |
| SG | Breast pain | breast_cancer_primary | 3 | 0.44 (0.14-1.38) | 0.27 | Ineligible |
| SG | Breast pain | breast_cancer_primary | 3 | 0.44 (0.14-1.38) | 0.27 | Ineligible |
| SG | Burning sensation | breast_cancer_primary | 3 | 0.29 (0.09-0.89) | 0.20 | Ineligible |
| SG | Burning sensation | breast_cancer_primary | 3 | 0.29 (0.09-0.89) | 0.20 | Ineligible |
| SG | Cancer pain | breast_cancer_primary | 3 | 0.98 (0.31-3.05) | 0.45 | Ineligible |
| SG | Cancer pain | breast_cancer_primary | 3 | 0.98 (0.31-3.05) | 0.45 | Ineligible |
| SG | Cardiac arrest | breast_cancer_primary | 3 | 0.43 (0.14-1.33) | 0.27 | Ineligible |
| SG | Cardiac arrest | breast_cancer_primary | 3 | 0.43 (0.14-1.33) | 0.27 | Ineligible |
| SG | Catheter site infection | breast_cancer_primary | 3 | 3.31 (1.04-10.49) | 0.72 | Ineligible |
| SG | Catheter site infection | breast_cancer_primary | 3 | 3.31 (1.04-10.49) | 0.72 | Ineligible |
| SG | Central venous catheterisation | breast_cancer_primary | 3 | 6.15 (1.90-19.84) | 0.90 | Ineligible |
| SG | Central venous catheterisation | breast_cancer_primary | 3 | 6.15 (1.90-19.84) | 0.90 | Ineligible |
| SG | Cholangiocarcinoma | breast_cancer_primary | 3 | 36.89 (9.53-142.72) | 3.98 | Ineligible |
| SG | Cholangiocarcinoma | breast_cancer_primary | 3 | 36.89 (9.53-142.72) | 3.98 | Ineligible |
| SG | Cholangitis | breast_cancer_primary | 3 | 2.72 (0.86-8.58) | 0.68 | Ineligible |
| SG | Cholangitis | breast_cancer_primary | 3 | 2.72 (0.86-8.58) | 0.68 | Ineligible |
| SG | Cholecystitis | breast_cancer_primary | 3 | 1.34 (0.43-4.21) | 0.52 | Ineligible |
| SG | Cholecystitis | breast_cancer_primary | 3 | 1.34 (0.43-4.21) | 0.52 | Ineligible |
| SG | Cholecystitis acute | breast_cancer_primary | 3 | 3.27 (1.03-10.36) | 0.72 | Ineligible |
| SG | Cholecystitis acute | breast_cancer_primary | 3 | 3.27 (1.03-10.36) | 0.72 | Ineligible |
| SG | Cognitive disorder | breast_cancer_primary | 3 | 0.41 (0.13-1.29) | 0.26 | Ineligible |
| SG | Cognitive disorder | breast_cancer_primary | 3 | 0.41 (0.13-1.29) | 0.26 | Ineligible |
| SG | Cold sweat | breast_cancer_primary | 3 | 1.86 (0.59-5.83) | 0.59 | Ineligible |
| SG | Cold sweat | breast_cancer_primary | 3 | 1.86 (0.59-5.83) | 0.59 | Ineligible |
| SG | Congenital aplasia | breast_cancer_primary | 3 | 11.23 (3.37-37.41) | 1.21 | Ineligible |
| SG | Congenital aplasia | breast_cancer_primary | 3 | 11.23 (3.37-37.41) | 1.21 | Ineligible |
| SG | Dermatitis | breast_cancer_primary | 3 | 0.61 (0.20-1.91) | 0.34 | Ineligible |
| SG | Dermatitis | breast_cancer_primary | 3 | 0.61 (0.20-1.91) | 0.34 | Ineligible |
| SG | Diabetes mellitus | breast_cancer_primary | 3 | 0.23 (0.07-0.71) | 0.17 | Ineligible |
| SG | Diabetes mellitus | breast_cancer_primary | 3 | 0.23 (0.07-0.71) | 0.17 | Ineligible |
| SG | Diplopia | breast_cancer_primary | 3 | 0.84 (0.27-2.63) | 0.41 | Ineligible |
| SG | Diplopia | breast_cancer_primary | 3 | 0.84 (0.27-2.63) | 0.41 | Ineligible |
| SG | Dizziness postural | breast_cancer_primary | 3 | 3.00 (0.95-9.50) | 0.70 | Ineligible |
| SG | Dizziness postural | breast_cancer_primary | 3 | 3.00 (0.95-9.50) | 0.70 | Ineligible |
| SG | Dysuria | breast_cancer_primary | 3 | 0.42 (0.14-1.32) | 0.27 | Ineligible |
| SG | Dysuria | breast_cancer_primary | 3 | 0.42 (0.14-1.32) | 0.27 | Ineligible |
| SG | Ejection fraction decreased | breast_cancer_primary | 3 | 0.12 (0.04-0.38) | 0.09 | Ineligible |
| SG | Ejection fraction decreased | breast_cancer_primary | 3 | 0.12 (0.04-0.38) | 0.09 | Ineligible |
| SG | Electrocardiogram T wave abnormal | breast_cancer_primary | 3 | 5.74 (1.78-18.47) | 0.87 | Ineligible |
| SG | Electrocardiogram T wave abnormal | breast_cancer_primary | 3 | 5.74 (1.78-18.47) | 0.87 | Ineligible |
| SG | Emotional disorder | breast_cancer_primary | 3 | 0.70 (0.23-2.19) | 0.37 | Ineligible |
| SG | Emotional disorder | breast_cancer_primary | 3 | 0.70 (0.23-2.19) | 0.37 | Ineligible |
| SG | Encephalopathy | breast_cancer_primary | 3 | 1.49 (0.48-4.67) | 0.54 | Ineligible |
| SG | Encephalopathy | breast_cancer_primary | 3 | 1.49 (0.48-4.67) | 0.54 | Ineligible |
| SG | Energy increased | breast_cancer_primary | 3 | 4.10 (1.29-13.06) | 0.77 | Ineligible |
| SG | Energy increased | breast_cancer_primary | 3 | 4.10 (1.29-13.06) | 0.77 | Ineligible |
| SG | Erysipelas | breast_cancer_primary | 3 | 0.99 (0.32-3.10) | 0.45 | Ineligible |
| SG | Erysipelas | breast_cancer_primary | 3 | 0.99 (0.32-3.10) | 0.45 | Ineligible |
| SG | Flashback | breast_cancer_primary | 3 | 51.64 (12.34-216.20) | 5.62 | Ineligible |
| SG | Flashback | breast_cancer_primary | 3 | 51.64 (12.34-216.20) | 5.62 | Ineligible |
| SG | Formication | breast_cancer_primary | 3 | 5.49 (1.71-17.66) | 0.86 | Ineligible |
| SG | Formication | breast_cancer_primary | 3 | 5.49 (1.71-17.66) | 0.86 | Ineligible |
| SG | Frequent bowel movements | breast_cancer_primary | 3 | 1.47 (0.47-4.62) | 0.54 | Ineligible |
| SG | Frequent bowel movements | breast_cancer_primary | 3 | 1.47 (0.47-4.62) | 0.54 | Ineligible |
| SG | Gastrointestinal haemorrhage | breast_cancer_primary | 3 | 0.63 (0.20-1.95) | 0.35 | Ineligible |
| SG | Gastrointestinal haemorrhage | breast_cancer_primary | 3 | 0.63 (0.20-1.95) | 0.35 | Ineligible |
| SG | Gastrointestinal pain | breast_cancer_primary | 3 | 1.58 (0.51-4.96) | 0.56 | Ineligible |
| SG | Gastrointestinal pain | breast_cancer_primary | 3 | 1.58 (0.51-4.96) | 0.56 | Ineligible |
| SG | Generalised oedema | breast_cancer_primary | 3 | 1.11 (0.35-3.46) | 0.47 | Ineligible |
| SG | Generalised oedema | breast_cancer_primary | 3 | 1.11 (0.35-3.46) | 0.47 | Ineligible |
| SG | Haematocrit decreased | breast_cancer_primary | 3 | 0.36 (0.12-1.13) | 0.24 | Ineligible |
| SG | Haematocrit decreased | breast_cancer_primary | 3 | 0.36 (0.12-1.13) | 0.24 | Ineligible |
| SG | Haematuria | breast_cancer_primary | 3 | 1.05 (0.34-3.28) | 0.46 | Ineligible |
| SG | Haematuria | breast_cancer_primary | 3 | 1.05 (0.34-3.28) | 0.46 | Ineligible |
| SG | Head discomfort | breast_cancer_primary | 3 | 1.02 (0.33-3.17) | 0.45 | Ineligible |
| SG | Head discomfort | breast_cancer_primary | 3 | 1.02 (0.33-3.17) | 0.45 | Ineligible |
| SG | Hemiparesis | breast_cancer_primary | 3 | 1.68 (0.53-5.26) | 0.57 | Ineligible |
| SG | Hemiparesis | breast_cancer_primary | 3 | 1.68 (0.53-5.26) | 0.57 | Ineligible |
| SG | Hepatitis | breast_cancer_primary | 3 | 0.43 (0.14-1.33) | 0.27 | Ineligible |
| SG | Hepatitis | breast_cancer_primary | 3 | 0.43 (0.14-1.33) | 0.27 | Ineligible |
| SG | Hepatitis acute | breast_cancer_primary | 3 | 2.05 (0.65-6.44) | 0.61 | Ineligible |
| SG | Hepatitis acute | breast_cancer_primary | 3 | 2.05 (0.65-6.44) | 0.61 | Ineligible |
| SG | Hepatocellular injury | breast_cancer_primary | 3 | 0.60 (0.19-1.86) | 0.34 | Ineligible |
| SG | Hepatocellular injury | breast_cancer_primary | 3 | 0.60 (0.19-1.86) | 0.34 | Ineligible |
| SG | Hepatomegaly | breast_cancer_primary | 3 | 1.04 (0.33-3.25) | 0.46 | Ineligible |
| SG | Hepatomegaly | breast_cancer_primary | 3 | 1.04 (0.33-3.25) | 0.46 | Ineligible |
| SG | Hypokinesia | breast_cancer_primary | 3 | 0.97 (0.31-3.03) | 0.44 | Ineligible |
| SG | Hypokinesia | breast_cancer_primary | 3 | 0.97 (0.31-3.03) | 0.44 | Ineligible |
| SG | Hypomagnesaemia | breast_cancer_primary | 3 | 0.96 (0.31-3.01) | 0.44 | Ineligible |
| SG | Hypomagnesaemia | breast_cancer_primary | 3 | 0.96 (0.31-3.01) | 0.44 | Ineligible |
| SG | Hypoproteinaemia | breast_cancer_primary | 3 | 6.15 (1.90-19.84) | 0.90 | Ineligible |
| SG | Hypoproteinaemia | breast_cancer_primary | 3 | 6.15 (1.90-19.84) | 0.90 | Ineligible |
| SG | Hypovolaemic shock | breast_cancer_primary | 3 | 3.15 (0.99-9.97) | 0.71 | Ineligible |
| SG | Hypovolaemic shock | breast_cancer_primary | 3 | 3.15 (0.99-9.97) | 0.71 | Ineligible |
| SG | Ileus | breast_cancer_primary | 3 | 1.56 (0.50-4.90) | 0.55 | Ineligible |
| SG | Ileus | breast_cancer_primary | 3 | 1.56 (0.50-4.90) | 0.55 | Ineligible |
| SG | Ileus paralytic | breast_cancer_primary | 3 | 5.87 (1.82-18.91) | 0.88 | Ineligible |
| SG | Ileus paralytic | breast_cancer_primary | 3 | 5.87 (1.82-18.91) | 0.88 | Ineligible |
| SG | Immune system disorder | breast_cancer_primary | 3 | 0.71 (0.23-2.20) | 0.37 | Ineligible |
| SG | Immune system disorder | breast_cancer_primary | 3 | 0.71 (0.23-2.20) | 0.37 | Ineligible |
| SG | Immunodeficiency | breast_cancer_primary | 3 | 0.72 (0.23-2.25) | 0.38 | Ineligible |
| SG | Immunodeficiency | breast_cancer_primary | 3 | 0.72 (0.23-2.25) | 0.38 | Ineligible |
| SG | Incorrect dosage administered | breast_cancer_primary | 3 | 11.74 (3.51-39.23) | 1.25 | Ineligible |
| SG | Incorrect dosage administered | breast_cancer_primary | 3 | 11.74 (3.51-39.23) | 1.25 | Ineligible |
| SG | Ischaemic stroke | breast_cancer_primary | 3 | 0.56 (0.18-1.74) | 0.32 | Ineligible |
| SG | Ischaemic stroke | breast_cancer_primary | 3 | 0.56 (0.18-1.74) | 0.32 | Ineligible |
| SG | Jaundice | breast_cancer_primary | 3 | 0.41 (0.13-1.28) | 0.26 | Ineligible |
| SG | Jaundice | breast_cancer_primary | 3 | 0.41 (0.13-1.28) | 0.26 | Ineligible |
| SG | Joint swelling | breast_cancer_primary | 3 | 0.30 (0.10-0.94) | 0.21 | Ineligible |
| SG | Joint swelling | breast_cancer_primary | 3 | 0.30 (0.10-0.94) | 0.21 | Ineligible |
| SG | Maternal exposure during pregnancy | breast_cancer_primary | 3 | 0.73 (0.24-2.28) | 0.38 | Ineligible |
| SG | Maternal exposure during pregnancy | breast_cancer_primary | 3 | 0.73 (0.24-2.28) | 0.38 | Ineligible |
| SG | Medication error | breast_cancer_primary | 3 | 1.61 (0.51-5.06) | 0.56 | Ineligible |
| SG | Medication error | breast_cancer_primary | 3 | 1.61 (0.51-5.06) | 0.56 | Ineligible |
| SG | Migraine | breast_cancer_primary | 3 | 0.42 (0.14-1.32) | 0.27 | Ineligible |
| SG | Migraine | breast_cancer_primary | 3 | 0.42 (0.14-1.32) | 0.27 | Ineligible |
| SG | Neutrophil count increased | breast_cancer_primary | 3 | 0.89 (0.29-2.78) | 0.42 | Ineligible |
| SG | Neutrophil count increased | breast_cancer_primary | 3 | 0.89 (0.29-2.78) | 0.42 | Ineligible |
| SG | Oral pain | breast_cancer_primary | 3 | 0.21 (0.07-0.66) | 0.15 | Ineligible |
| SG | Oral pain | breast_cancer_primary | 3 | 0.21 (0.07-0.66) | 0.15 | Ineligible |
| SG | Palmar-plantar erythrodysaesthesia syndrome | breast_cancer_primary | 3 | 0.09 (0.03-0.27) | 0.07 | Ineligible |
| SG | Palmar-plantar erythrodysaesthesia syndrome | breast_cancer_primary | 3 | 0.09 (0.03-0.27) | 0.07 | Ineligible |
| SG | Pancreatitis | breast_cancer_primary | 3 | 0.78 (0.25-2.42) | 0.39 | Ineligible |
| SG | Pancreatitis | breast_cancer_primary | 3 | 0.78 (0.25-2.42) | 0.39 | Ineligible |
| SG | Pharyngitis | breast_cancer_primary | 3 | 1.33 (0.43-4.16) | 0.52 | Ineligible |
| SG | Pharyngitis | breast_cancer_primary | 3 | 1.33 (0.43-4.16) | 0.52 | Ineligible |
| SG | Pneumocystis jirovecii pneumonia | breast_cancer_primary | 3 | 0.49 (0.16-1.52) | 0.29 | Ineligible |
| SG | Pneumocystis jirovecii pneumonia | breast_cancer_primary | 3 | 0.49 (0.16-1.52) | 0.29 | Ineligible |
| SG | Pneumonia aspiration | breast_cancer_primary | 3 | 2.13 (0.68-6.71) | 0.62 | Ineligible |
| SG | Pneumonia aspiration | breast_cancer_primary | 3 | 2.13 (0.68-6.71) | 0.62 | Ineligible |
| SG | Pollakiuria | breast_cancer_primary | 3 | 0.57 (0.18-1.78) | 0.33 | Ineligible |
| SG | Pollakiuria | breast_cancer_primary | 3 | 0.57 (0.18-1.78) | 0.33 | Ineligible |
| SG | Pulmonary toxicity | breast_cancer_primary | 3 | 0.75 (0.24-2.34) | 0.39 | Ineligible |
| SG | Pulmonary toxicity | breast_cancer_primary | 3 | 0.75 (0.24-2.34) | 0.39 | Ineligible |
| SG | Pyelonephritis | breast_cancer_primary | 3 | 1.56 (0.50-4.90) | 0.55 | Ineligible |
| SG | Pyelonephritis | breast_cancer_primary | 3 | 1.56 (0.50-4.90) | 0.55 | Ineligible |
| SG | Sedation | breast_cancer_primary | 3 | 4.69 (1.47-15.01) | 0.81 | Ineligible |
| SG | Sedation | breast_cancer_primary | 3 | 4.69 (1.47-15.01) | 0.81 | Ineligible |
| SG | Skin cancer | breast_cancer_primary | 3 | 2.61 (0.83-8.23) | 0.67 | Ineligible |
| SG | Skin cancer | breast_cancer_primary | 3 | 2.61 (0.83-8.23) | 0.67 | Ineligible |
| SG | Skin hyperpigmentation | breast_cancer_primary | 3 | 1.62 (0.52-5.09) | 0.56 | Ineligible |
| SG | Skin hyperpigmentation | breast_cancer_primary | 3 | 1.62 (0.52-5.09) | 0.56 | Ineligible |
| SG | Skin tightness | breast_cancer_primary | 3 | 5.49 (1.71-17.66) | 0.86 | Ineligible |
| SG | Skin tightness | breast_cancer_primary | 3 | 5.49 (1.71-17.66) | 0.86 | Ineligible |
| SG | Staphylococcal infection | breast_cancer_primary | 3 | 1.09 (0.35-3.40) | 0.47 | Ineligible |
| SG | Staphylococcal infection | breast_cancer_primary | 3 | 1.09 (0.35-3.40) | 0.47 | Ineligible |
| SG | Tachypnoea | breast_cancer_primary | 3 | 1.19 (0.38-3.72) | 0.49 | Ineligible |
| SG | Tachypnoea | breast_cancer_primary | 3 | 1.19 (0.38-3.72) | 0.49 | Ineligible |
| SG | Therapeutic product effect decreased | breast_cancer_primary | 3 | 3.35 (1.06-10.63) | 0.72 | Ineligible |
| SG | Therapeutic product effect decreased | breast_cancer_primary | 3 | 3.35 (1.06-10.63) | 0.72 | Ineligible |
| SG | Tongue coated | breast_cancer_primary | 3 | 5.61 (1.74-18.06) | 0.86 | Ineligible |
| SG | Tongue coated | breast_cancer_primary | 3 | 5.61 (1.74-18.06) | 0.86 | Ineligible |
| SG | Treatment failure | breast_cancer_primary | 3 | 0.49 (0.16-1.54) | 0.30 | Ineligible |
| SG | Treatment failure | breast_cancer_primary | 3 | 0.49 (0.16-1.54) | 0.30 | Ineligible |
| SG | Type 2 diabetes mellitus | breast_cancer_primary | 3 | 1.05 (0.34-3.28) | 0.46 | Ineligible |
| SG | Type 2 diabetes mellitus | breast_cancer_primary | 3 | 1.05 (0.34-3.28) | 0.46 | Ineligible |
| SG | Underdose | breast_cancer_primary | 3 | 0.63 (0.20-1.96) | 0.35 | Ineligible |
| SG | Underdose | breast_cancer_primary | 3 | 0.63 (0.20-1.96) | 0.35 | Ineligible |
| SG | Vascular device infection | breast_cancer_primary | 3 | 1.32 (0.42-4.12) | 0.51 | Ineligible |
| SG | Vascular device infection | breast_cancer_primary | 3 | 1.32 (0.42-4.12) | 0.51 | Ineligible |
| SG | Vascular device occlusion | breast_cancer_primary | 3 | 28.69 (7.76-106.03) | 2.67 | Ineligible |
| SG | Vascular device occlusion | breast_cancer_primary | 3 | 28.69 (7.76-106.03) | 2.67 | Ineligible |
| T-DM1 | Disease progression | breast_cancer_primary | 260 | 2.16 (1.90-2.46) | 1.84 | Eligible |
| T-DM1 | Platelet count decreased | breast_cancer_primary | 252 | 4.30 (3.77-4.90) | 3.47 | Eligible |
| T-DM1 | Death | breast_cancer_primary | 250 | 0.99 (0.87-1.12) | 0.89 | Ineligible |
| T-DM1 | Death | breast_cancer_primary | 250 | 0.99 (0.87-1.12) | 0.89 | Ineligible |
| T-DM1 | Fatigue | breast_cancer_primary | 230 | 0.62 (0.55-0.71) | 0.58 | Ineligible |
| T-DM1 | Fatigue | breast_cancer_primary | 230 | 0.62 (0.55-0.71) | 0.58 | Ineligible |
| T-DM1 | Thrombocytopenia | breast_cancer_primary | 225 | 3.62 (3.15-4.15) | 2.95 | Eligible |
| T-DM1 | Nausea | breast_cancer_primary | 187 | 0.60 (0.52-0.69) | 0.55 | Ineligible |
| T-DM1 | Nausea | breast_cancer_primary | 187 | 0.60 (0.52-0.69) | 0.55 | Ineligible |
| T-DM1 | Pyrexia | breast_cancer_primary | 160 | 1.44 (1.23-1.69) | 1.23 | Eligible |
| T-DM1 | Myelosuppression | breast_cancer_primary | 158 | 1.99 (1.69-2.34) | 1.67 | Eligible |
| T-DM1 | Drug ineffective | breast_cancer_primary | 140 | 1.66 (1.40-1.97) | 1.40 | Eligible |
| T-DM1 | Neuropathy peripheral | breast_cancer_primary | 135 | 1.85 (1.55-2.20) | 1.54 | Eligible |
| T-DM1 | Epistaxis | breast_cancer_primary | 131 | 3.51 (2.94-4.20) | 2.78 | Eligible |
| T-DM1 | Metastases to central nervous system | breast_cancer_primary | 120 | 3.67 (3.05-4.42) | 2.88 | Eligible |
| T-DM1 | Pneumonia | breast_cancer_primary | 119 | 1.80 (1.50-2.17) | 1.49 | Eligible |
| T-DM1 | Pain | breast_cancer_primary | 114 | 0.65 (0.54-0.79) | 0.58 | Ineligible |
| T-DM1 | Pain | breast_cancer_primary | 114 | 0.65 (0.54-0.79) | 0.58 | Ineligible |
| T-DM1 | Headache | breast_cancer_primary | 113 | 1.09 (0.90-1.32) | 0.93 | Ineligible |
| T-DM1 | Headache | breast_cancer_primary | 113 | 1.09 (0.90-1.32) | 0.93 | Ineligible |
| T-DM1 | Dyspnoea | breast_cancer_primary | 108 | 0.69 (0.57-0.84) | 0.60 | Ineligible |
| T-DM1 | Dyspnoea | breast_cancer_primary | 108 | 0.69 (0.57-0.84) | 0.60 | Ineligible |
| T-DM1 | Vomiting | breast_cancer_primary | 103 | 0.60 (0.50-0.74) | 0.53 | Ineligible |
| T-DM1 | Vomiting | breast_cancer_primary | 103 | 0.60 (0.50-0.74) | 0.53 | Ineligible |
| T-DM1 | Aspartate aminotransferase increased | breast_cancer_primary | 102 | 3.48 (2.85-4.26) | 2.70 | Eligible |
| T-DM1 | Asthenia | breast_cancer_primary | 102 | 0.67 (0.55-0.81) | 0.58 | Ineligible |
| T-DM1 | Asthenia | breast_cancer_primary | 102 | 0.67 (0.55-0.81) | 0.58 | Ineligible |
| T-DM1 | Diarrhoea | breast_cancer_primary | 97 | 0.28 (0.23-0.34) | 0.26 | Ineligible |
| T-DM1 | Diarrhoea | breast_cancer_primary | 97 | 0.28 (0.23-0.34) | 0.26 | Ineligible |
| T-DM1 | Anaemia | breast_cancer_primary | 93 | 0.84 (0.69-1.04) | 0.72 | Ineligible |
| T-DM1 | Anaemia | breast_cancer_primary | 93 | 0.84 (0.69-1.04) | 0.72 | Ineligible |
| T-DM1 | Alanine aminotransferase increased | breast_cancer_primary | 82 | 2.56 (2.05-3.20) | 1.99 | Eligible |
| T-DM1 | Weight decreased | breast_cancer_primary | 81 | 0.99 (0.80-1.24) | 0.83 | Ineligible |
| T-DM1 | Weight decreased | breast_cancer_primary | 81 | 0.99 (0.80-1.24) | 0.83 | Ineligible |
| T-DM1 | Decreased appetite | breast_cancer_primary | 78 | 0.62 (0.49-0.77) | 0.53 | Ineligible |
| T-DM1 | Decreased appetite | breast_cancer_primary | 78 | 0.62 (0.49-0.77) | 0.53 | Ineligible |
| T-DM1 | Cough | breast_cancer_primary | 73 | 0.83 (0.66-1.05) | 0.69 | Ineligible |
| T-DM1 | Cough | breast_cancer_primary | 73 | 0.83 (0.66-1.05) | 0.69 | Ineligible |
| T-DM1 | Rash | breast_cancer_primary | 73 | 0.66 (0.52-0.83) | 0.56 | Ineligible |
| T-DM1 | Rash | breast_cancer_primary | 73 | 0.66 (0.52-0.83) | 0.56 | Ineligible |
| T-DM1 | Off label use | breast_cancer_primary | 72 | 0.41 (0.32-0.52) | 0.35 | Ineligible |
| T-DM1 | Off label use | breast_cancer_primary | 72 | 0.41 (0.32-0.52) | 0.35 | Ineligible |
| T-DM1 | Constipation | breast_cancer_primary | 69 | 0.77 (0.61-0.98) | 0.65 | Ineligible |
| T-DM1 | Constipation | breast_cancer_primary | 69 | 0.77 (0.61-0.98) | 0.65 | Ineligible |
| T-DM1 | Malaise | breast_cancer_primary | 62 | 0.57 (0.45-0.74) | 0.48 | Ineligible |
| T-DM1 | Malaise | breast_cancer_primary | 62 | 0.57 (0.45-0.74) | 0.48 | Ineligible |
| T-DM1 | Myalgia | breast_cancer_primary | 62 | 1.44 (1.12-1.86) | 1.14 | Eligible |
| T-DM1 | Arthralgia | breast_cancer_primary | 61 | 0.55 (0.42-0.70) | 0.46 | Ineligible |
| T-DM1 | Arthralgia | breast_cancer_primary | 61 | 0.55 (0.42-0.70) | 0.46 | Ineligible |
| T-DM1 | Dizziness | breast_cancer_primary | 61 | 0.69 (0.54-0.89) | 0.57 | Ineligible |
| T-DM1 | Dizziness | breast_cancer_primary | 61 | 0.69 (0.54-0.89) | 0.57 | Ineligible |
| T-DM1 | Interstitial lung disease | breast_cancer_primary | 61 | 1.39 (1.08-1.80) | 1.10 | Eligible |
| T-DM1 | Hypoaesthesia | breast_cancer_primary | 59 | 1.54 (1.19-1.99) | 1.21 | Eligible |
| T-DM1 | Hepatic function abnormal | breast_cancer_primary | 57 | 2.31 (1.77-3.02) | 1.74 | Eligible |
| T-DM1 | Chills | breast_cancer_primary | 56 | 1.58 (1.21-2.06) | 1.23 | Eligible |
| T-DM1 | Hepatic cirrhosis | breast_cancer_primary | 55 | 10.37 (7.79-13.79) | 7.14 | Eligible |
| T-DM1 | Pneumonitis | breast_cancer_primary | 52 | 1.52 (1.15-2.01) | 1.18 | Eligible |
| T-DM1 | Blood bilirubin increased | breast_cancer_primary | 48 | 3.52 (2.63-4.72) | 2.45 | Eligible |
| T-DM1 | Ejection fraction decreased | breast_cancer_primary | 48 | 1.58 (1.19-2.11) | 1.21 | Eligible |
| T-DM1 | Neutropenia | breast_cancer_primary | 48 | 0.24 (0.18-0.32) | 0.21 | Ineligible |
| T-DM1 | Neutropenia | breast_cancer_primary | 48 | 0.24 (0.18-0.32) | 0.21 | Ineligible |
| T-DM1 | Nasopharyngitis | breast_cancer_primary | 46 | 1.31 (0.97-1.75) | 1.01 | Ineligible |
| T-DM1 | Nasopharyngitis | breast_cancer_primary | 46 | 1.31 (0.97-1.75) | 1.01 | Ineligible |
| T-DM1 | Pain in extremity | breast_cancer_primary | 46 | 0.62 (0.46-0.82) | 0.50 | Ineligible |
| T-DM1 | Pain in extremity | breast_cancer_primary | 46 | 0.62 (0.46-0.82) | 0.50 | Ineligible |
| T-DM1 | Paraesthesia | breast_cancer_primary | 46 | 1.16 (0.87-1.56) | 0.91 | Ineligible |
| T-DM1 | Paraesthesia | breast_cancer_primary | 46 | 1.16 (0.87-1.56) | 0.91 | Ineligible |
| T-DM1 | Pleural effusion | breast_cancer_primary | 46 | 0.97 (0.73-1.30) | 0.77 | Ineligible |
| T-DM1 | Pleural effusion | breast_cancer_primary | 46 | 0.97 (0.73-1.30) | 0.77 | Ineligible |
| T-DM1 | Abdominal pain | breast_cancer_primary | 45 | 0.81 (0.60-1.09) | 0.64 | Ineligible |
| T-DM1 | Abdominal pain | breast_cancer_primary | 45 | 0.81 (0.60-1.09) | 0.64 | Ineligible |
| T-DM1 | Blood alkaline phosphatase increased | breast_cancer_primary | 45 | 3.56 (2.63-4.81) | 2.44 | Eligible |
| T-DM1 | Cardiac failure | breast_cancer_primary | 45 | 1.48 (1.10-2.00) | 1.13 | Eligible |
| T-DM1 | Hypokalaemia | breast_cancer_primary | 45 | 1.93 (1.43-2.60) | 1.43 | Eligible |
| T-DM1 | White blood cell count decreased | breast_cancer_primary | 43 | 0.23 (0.17-0.31) | 0.19 | Ineligible |
| T-DM1 | White blood cell count decreased | breast_cancer_primary | 43 | 0.23 (0.17-0.31) | 0.19 | Ineligible |
| T-DM1 | Hepatic enzyme increased | breast_cancer_primary | 42 | 1.95 (1.43-2.65) | 1.43 | Eligible |
| T-DM1 | Hypertension | breast_cancer_primary | 41 | 0.99 (0.73-1.35) | 0.77 | Ineligible |
| T-DM1 | Hypertension | breast_cancer_primary | 41 | 0.99 (0.73-1.35) | 0.77 | Ineligible |
| T-DM1 | Portal hypertension | breast_cancer_primary | 41 | 19.93 (14.05-28.27) | 11.56 | Eligible |
| T-DM1 | Erythema | breast_cancer_primary | 40 | 0.81 (0.59-1.10) | 0.63 | Ineligible |
| T-DM1 | Erythema | breast_cancer_primary | 40 | 0.81 (0.59-1.10) | 0.63 | Ineligible |
| T-DM1 | Liver disorder | breast_cancer_primary | 40 | 2.58 (1.88-3.54) | 1.81 | Eligible |
| T-DM1 | Vision blurred | breast_cancer_primary | 40 | 2.05 (1.50-2.82) | 1.48 | Eligible |
| T-DM1 | Back pain | breast_cancer_primary | 38 | 0.52 (0.38-0.71) | 0.41 | Ineligible |
| T-DM1 | Back pain | breast_cancer_primary | 38 | 0.52 (0.38-0.71) | 0.41 | Ineligible |
| T-DM1 | Hepatotoxicity | breast_cancer_primary | 38 | 2.18 (1.58-3.02) | 1.55 | Eligible |
| T-DM1 | Infusion related reaction | breast_cancer_primary | 38 | 2.07 (1.50-2.87) | 1.48 | Eligible |
| T-DM1 | Seizure | breast_cancer_primary | 38 | 2.38 (1.72-3.29) | 1.67 | Eligible |
| T-DM1 | Breast cancer | breast_cancer_primary | 36 | 0.56 (0.41-0.78) | 0.45 | Ineligible |
| T-DM1 | Breast cancer | breast_cancer_primary | 36 | 0.56 (0.41-0.78) | 0.45 | Ineligible |
| T-DM1 | Nodular regenerative hyperplasia | breast_cancer_primary | 36 | 58.29 (37.30-91.07) | 21.81 | Eligible |
| T-DM1 | Urinary tract infection | breast_cancer_primary | 36 | 0.81 (0.58-1.12) | 0.62 | Ineligible |
| T-DM1 | Urinary tract infection | breast_cancer_primary | 36 | 0.81 (0.58-1.12) | 0.62 | Ineligible |
| T-DM1 | Abdominal pain upper | breast_cancer_primary | 35 | 0.74 (0.53-1.03) | 0.57 | Ineligible |
| T-DM1 | Abdominal pain upper | breast_cancer_primary | 35 | 0.74 (0.53-1.03) | 0.57 | Ineligible |
| T-DM1 | Alopecia | breast_cancer_primary | 35 | 0.08 (0.06-0.11) | 0.07 | Ineligible |
| T-DM1 | Alopecia | breast_cancer_primary | 35 | 0.08 (0.06-0.11) | 0.07 | Ineligible |
| T-DM1 | Malignant neoplasm progression | breast_cancer_primary | 35 | 0.21 (0.15-0.29) | 0.17 | Ineligible |
| T-DM1 | Malignant neoplasm progression | breast_cancer_primary | 35 | 0.21 (0.15-0.29) | 0.17 | Ineligible |
| T-DM1 | Metastases to liver | breast_cancer_primary | 35 | 0.68 (0.49-0.95) | 0.53 | Ineligible |
| T-DM1 | Metastases to liver | breast_cancer_primary | 35 | 0.68 (0.49-0.95) | 0.53 | Ineligible |
| T-DM1 | Peripheral swelling | breast_cancer_primary | 34 | 0.84 (0.60-1.18) | 0.64 | Ineligible |
| T-DM1 | Peripheral swelling | breast_cancer_primary | 34 | 0.84 (0.60-1.18) | 0.64 | Ineligible |
| T-DM1 | Pruritus | breast_cancer_primary | 34 | 0.47 (0.33-0.66) | 0.37 | Ineligible |
| T-DM1 | Pruritus | breast_cancer_primary | 34 | 0.47 (0.33-0.66) | 0.37 | Ineligible |
| T-DM1 | Dry mouth | breast_cancer_primary | 32 | 1.43 (1.01-2.03) | 1.04 | Eligible |
| T-DM1 | General physical health deterioration | breast_cancer_primary | 32 | 0.68 (0.48-0.96) | 0.52 | Ineligible |
| T-DM1 | General physical health deterioration | breast_cancer_primary | 32 | 0.68 (0.48-0.96) | 0.52 | Ineligible |
| T-DM1 | Gingival bleeding | breast_cancer_primary | 32 | 7.41 (5.14-10.69) | 4.83 | Eligible |
| T-DM1 | Haemoglobin decreased | breast_cancer_primary | 32 | 0.65 (0.46-0.92) | 0.50 | Ineligible |
| T-DM1 | Haemoglobin decreased | breast_cancer_primary | 32 | 0.65 (0.46-0.92) | 0.50 | Ineligible |
| T-DM1 | Transaminases increased | breast_cancer_primary | 32 | 2.11 (1.48-3.01) | 1.46 | Eligible |
| T-DM1 | Fall | breast_cancer_primary | 31 | 0.70 (0.49-1.00) | 0.54 | Ineligible |
| T-DM1 | Fall | breast_cancer_primary | 31 | 0.70 (0.49-1.00) | 0.54 | Ineligible |
| T-DM1 | Metastases to lung | breast_cancer_primary | 31 | 0.97 (0.68-1.38) | 0.73 | Ineligible |
| T-DM1 | Metastases to lung | breast_cancer_primary | 31 | 0.97 (0.68-1.38) | 0.73 | Ineligible |
| T-DM1 | Radiation necrosis | breast_cancer_primary | 31 | 25.67 (16.96-38.85) | 13.16 | Eligible |
| T-DM1 | Cardiotoxicity | breast_cancer_primary | 29 | 1.53 (1.06-2.21) | 1.08 | Eligible |
| T-DM1 | Cerebral haemorrhage | breast_cancer_primary | 29 | 6.60 (4.50-9.68) | 4.13 | Eligible |
| T-DM1 | Ill-defined disorder | breast_cancer_primary | 29 | 2.69 (1.85-3.90) | 1.75 | Eligible |
| T-DM1 | Respiratory failure | breast_cancer_primary | 29 | 1.63 (1.13-2.36) | 1.15 | Eligible |
| T-DM1 | Sepsis | breast_cancer_primary | 29 | 1.14 (0.79-1.64) | 0.83 | Ineligible |
| T-DM1 | Sepsis | breast_cancer_primary | 29 | 1.14 (0.79-1.64) | 0.83 | Ineligible |
| T-DM1 | Insomnia | breast_cancer_primary | 28 | 0.56 (0.38-0.81) | 0.43 | Ineligible |
| T-DM1 | Insomnia | breast_cancer_primary | 28 | 0.56 (0.38-0.81) | 0.43 | Ineligible |
| T-DM1 | Neoplasm malignant | breast_cancer_primary | 28 | 3.00 (2.05-4.38) | 1.90 | Eligible |
| T-DM1 | No adverse event | breast_cancer_primary | 28 | 1.40 (0.96-2.04) | 1.00 | Ineligible |
| T-DM1 | No adverse event | breast_cancer_primary | 28 | 1.40 (0.96-2.04) | 1.00 | Ineligible |
| T-DM1 | Ascites | breast_cancer_primary | 27 | 1.28 (0.87-1.88) | 0.92 | Ineligible |
| T-DM1 | Ascites | breast_cancer_primary | 27 | 1.28 (0.87-1.88) | 0.92 | Ineligible |
| T-DM1 | Dehydration | breast_cancer_primary | 27 | 0.56 (0.38-0.81) | 0.43 | Ineligible |
| T-DM1 | Dehydration | breast_cancer_primary | 27 | 0.56 (0.38-0.81) | 0.43 | Ineligible |
| T-DM1 | Gamma-glutamyltransferase increased | breast_cancer_primary | 27 | 1.92 (1.31-2.82) | 1.30 | Eligible |
| T-DM1 | Hyponatraemia | breast_cancer_primary | 27 | 2.02 (1.38-2.97) | 1.36 | Eligible |
| T-DM1 | Neutrophil count decreased | breast_cancer_primary | 27 | 0.43 (0.29-0.62) | 0.33 | Ineligible |
| T-DM1 | Neutrophil count decreased | breast_cancer_primary | 27 | 0.43 (0.29-0.62) | 0.33 | Ineligible |
| T-DM1 | Chest pain | breast_cancer_primary | 26 | 0.76 (0.51-1.11) | 0.56 | Ineligible |
| T-DM1 | Chest pain | breast_cancer_primary | 26 | 0.76 (0.51-1.11) | 0.56 | Ineligible |
| T-DM1 | Drug-induced liver injury | breast_cancer_primary | 26 | 2.04 (1.38-3.02) | 1.36 | Eligible |
| T-DM1 | Haemorrhage | breast_cancer_primary | 26 | 1.94 (1.32-2.88) | 1.31 | Eligible |
| T-DM1 | Hepatic failure | breast_cancer_primary | 26 | 1.68 (1.14-2.48) | 1.15 | Eligible |
| T-DM1 | Blood pressure increased | breast_cancer_primary | 25 | 1.20 (0.81-1.78) | 0.85 | Ineligible |
| T-DM1 | Blood pressure increased | breast_cancer_primary | 25 | 1.20 (0.81-1.78) | 0.85 | Ineligible |
| T-DM1 | Bone pain | breast_cancer_primary | 25 | 0.44 (0.30-0.66) | 0.34 | Ineligible |
| T-DM1 | Bone pain | breast_cancer_primary | 25 | 0.44 (0.30-0.66) | 0.34 | Ineligible |
| T-DM1 | Splenomegaly | breast_cancer_primary | 25 | 16.14 (10.42-25.00) | 9.05 | Eligible |
| T-DM1 | Stomatitis | breast_cancer_primary | 25 | 0.33 (0.23-0.50) | 0.26 | Ineligible |
| T-DM1 | Stomatitis | breast_cancer_primary | 25 | 0.33 (0.23-0.50) | 0.26 | Ineligible |
| T-DM1 | Muscular weakness | breast_cancer_primary | 24 | 1.26 (0.84-1.89) | 0.89 | Ineligible |
| T-DM1 | Muscular weakness | breast_cancer_primary | 24 | 1.26 (0.84-1.89) | 0.89 | Ineligible |
| T-DM1 | Pulmonary oedema | breast_cancer_primary | 24 | 1.35 (0.90-2.02) | 0.94 | Ineligible |
| T-DM1 | Pulmonary oedema | breast_cancer_primary | 24 | 1.35 (0.90-2.02) | 0.94 | Ineligible |
| T-DM1 | Red blood cell count decreased | breast_cancer_primary | 24 | 0.71 (0.47-1.06) | 0.53 | Ineligible |
| T-DM1 | Red blood cell count decreased | breast_cancer_primary | 24 | 0.71 (0.47-1.06) | 0.53 | Ineligible |
| T-DM1 | Breast cancer metastatic | breast_cancer_primary | 23 | 0.31 (0.21-0.47) | 0.24 | Ineligible |
| T-DM1 | Breast cancer metastatic | breast_cancer_primary | 23 | 0.31 (0.21-0.47) | 0.24 | Ineligible |
| T-DM1 | COVID-19 | breast_cancer_primary | 23 | 0.62 (0.41-0.94) | 0.46 | Ineligible |
| T-DM1 | COVID-19 | breast_cancer_primary | 23 | 0.62 (0.41-0.94) | 0.46 | Ineligible |
| T-DM1 | Hypersensitivity | breast_cancer_primary | 23 | 0.67 (0.44-1.01) | 0.50 | Ineligible |
| T-DM1 | Hypersensitivity | breast_cancer_primary | 23 | 0.67 (0.44-1.01) | 0.50 | Ineligible |
| T-DM1 | Lung disorder | breast_cancer_primary | 23 | 1.50 (0.99-2.27) | 1.02 | Ineligible |
| T-DM1 | Lung disorder | breast_cancer_primary | 23 | 1.50 (0.99-2.27) | 1.02 | Ineligible |
| T-DM1 | Metastases to bone | breast_cancer_primary | 23 | 0.34 (0.23-0.52) | 0.26 | Ineligible |
| T-DM1 | Metastases to bone | breast_cancer_primary | 23 | 0.34 (0.23-0.52) | 0.26 | Ineligible |
| T-DM1 | Visual impairment | breast_cancer_primary | 23 | 1.04 (0.69-1.57) | 0.74 | Ineligible |
| T-DM1 | Visual impairment | breast_cancer_primary | 23 | 1.04 (0.69-1.57) | 0.74 | Ineligible |
| T-DM1 | Contusion | breast_cancer_primary | 22 | 1.56 (1.02-2.38) | 1.05 | Eligible |
| T-DM1 | Hypotension | breast_cancer_primary | 22 | 0.70 (0.46-1.07) | 0.52 | Ineligible |
| T-DM1 | Hypotension | breast_cancer_primary | 22 | 0.70 (0.46-1.07) | 0.52 | Ineligible |
| T-DM1 | Pericardial effusion | breast_cancer_primary | 22 | 1.99 (1.30-3.05) | 1.29 | Eligible |
| T-DM1 | Blood lactate dehydrogenase increased | breast_cancer_primary | 21 | 2.84 (1.83-4.40) | 1.69 | Eligible |
| T-DM1 | Cellulitis | breast_cancer_primary | 21 | 1.33 (0.86-2.05) | 0.91 | Ineligible |
| T-DM1 | Cellulitis | breast_cancer_primary | 21 | 1.33 (0.86-2.05) | 0.91 | Ineligible |
| T-DM1 | Cerebrovascular accident | breast_cancer_primary | 21 | 1.18 (0.76-1.82) | 0.82 | Ineligible |
| T-DM1 | Cerebrovascular accident | breast_cancer_primary | 21 | 1.18 (0.76-1.82) | 0.82 | Ineligible |
| T-DM1 | Infection | breast_cancer_primary | 21 | 0.70 (0.45-1.07) | 0.51 | Ineligible |
| T-DM1 | Infection | breast_cancer_primary | 21 | 0.70 (0.45-1.07) | 0.51 | Ineligible |
| T-DM1 | Leukopenia | breast_cancer_primary | 21 | 0.33 (0.21-0.51) | 0.25 | Ineligible |
| T-DM1 | Leukopenia | breast_cancer_primary | 21 | 0.33 (0.21-0.51) | 0.25 | Ineligible |
| T-DM1 | Liver function test abnormal | breast_cancer_primary | 21 | 3.11 (2.00-4.82) | 1.81 | Eligible |
| T-DM1 | Muscle spasms | breast_cancer_primary | 21 | 0.65 (0.42-1.00) | 0.48 | Ineligible |
| T-DM1 | Muscle spasms | breast_cancer_primary | 21 | 0.65 (0.42-1.00) | 0.48 | Ineligible |
| T-DM1 | Neoplasm progression | breast_cancer_primary | 21 | 0.17 (0.11-0.25) | 0.13 | Ineligible |
| T-DM1 | Neoplasm progression | breast_cancer_primary | 21 | 0.17 (0.11-0.25) | 0.13 | Ineligible |
| T-DM1 | Dry eye | breast_cancer_primary | 20 | 1.47 (0.95-2.30) | 0.98 | Ineligible |
| T-DM1 | Dry eye | breast_cancer_primary | 20 | 1.47 (0.95-2.30) | 0.98 | Ineligible |
| T-DM1 | Febrile neutropenia | breast_cancer_primary | 20 | 0.32 (0.21-0.50) | 0.25 | Ineligible |
| T-DM1 | Febrile neutropenia | breast_cancer_primary | 20 | 0.32 (0.21-0.50) | 0.25 | Ineligible |
| T-DM1 | Gait disturbance | breast_cancer_primary | 20 | 0.53 (0.34-0.83) | 0.40 | Ineligible |
| T-DM1 | Gait disturbance | breast_cancer_primary | 20 | 0.53 (0.34-0.83) | 0.40 | Ineligible |
| T-DM1 | Gastrointestinal haemorrhage | breast_cancer_primary | 20 | 3.43 (2.19-5.39) | 1.93 | Eligible |
| T-DM1 | Hyperbilirubinaemia | breast_cancer_primary | 20 | 3.54 (2.26-5.56) | 1.98 | Eligible |
| T-DM1 | Lymphocyte count decreased | breast_cancer_primary | 20 | 2.09 (1.34-3.26) | 1.31 | Eligible |
| T-DM1 | Swelling | breast_cancer_primary | 20 | 1.38 (0.88-2.14) | 0.93 | Ineligible |
| T-DM1 | Swelling | breast_cancer_primary | 20 | 1.38 (0.88-2.14) | 0.93 | Ineligible |
| T-DM1 | Drug intolerance | breast_cancer_primary | 19 | 0.75 (0.47-1.18) | 0.54 | Ineligible |
| T-DM1 | Drug intolerance | breast_cancer_primary | 19 | 0.75 (0.47-1.18) | 0.54 | Ineligible |
| T-DM1 | Lacrimation increased | breast_cancer_primary | 19 | 1.12 (0.71-1.77) | 0.77 | Ineligible |
| T-DM1 | Lacrimation increased | breast_cancer_primary | 19 | 1.12 (0.71-1.77) | 0.77 | Ineligible |
| T-DM1 | Liver function test increased | breast_cancer_primary | 19 | 1.51 (0.96-2.39) | 0.99 | Ineligible |
| T-DM1 | Liver function test increased | breast_cancer_primary | 19 | 1.51 (0.96-2.39) | 0.99 | Ineligible |
| T-DM1 | Oxygen saturation decreased | breast_cancer_primary | 19 | 1.48 (0.94-2.33) | 0.97 | Ineligible |
| T-DM1 | Oxygen saturation decreased | breast_cancer_primary | 19 | 1.48 (0.94-2.33) | 0.97 | Ineligible |
| T-DM1 | Weight increased | breast_cancer_primary | 19 | 0.57 (0.36-0.90) | 0.42 | Ineligible |
| T-DM1 | Weight increased | breast_cancer_primary | 19 | 0.57 (0.36-0.90) | 0.42 | Ineligible |
| T-DM1 | Blood potassium decreased | breast_cancer_primary | 18 | 1.66 (1.04-2.66) | 1.06 | Eligible |
| T-DM1 | Brain oedema | breast_cancer_primary | 18 | 4.24 (2.63-6.84) | 2.20 | Eligible |
| T-DM1 | Cardiac disorder | breast_cancer_primary | 18 | 1.30 (0.81-2.08) | 0.86 | Ineligible |
| T-DM1 | Cardiac disorder | breast_cancer_primary | 18 | 1.30 (0.81-2.08) | 0.86 | Ineligible |
| T-DM1 | Chest discomfort | breast_cancer_primary | 18 | 0.67 (0.42-1.07) | 0.48 | Ineligible |
| T-DM1 | Chest discomfort | breast_cancer_primary | 18 | 0.67 (0.42-1.07) | 0.48 | Ineligible |
| T-DM1 | Jaundice | breast_cancer_primary | 18 | 1.98 (1.24-3.17) | 1.22 | Eligible |
| T-DM1 | Musculoskeletal pain | breast_cancer_primary | 18 | 1.45 (0.91-2.31) | 0.95 | Ineligible |
| T-DM1 | Musculoskeletal pain | breast_cancer_primary | 18 | 1.45 (0.91-2.31) | 0.95 | Ineligible |
| T-DM1 | Renal failure | breast_cancer_primary | 18 | 0.85 (0.54-1.36) | 0.60 | Ineligible |
| T-DM1 | Renal failure | breast_cancer_primary | 18 | 0.85 (0.54-1.36) | 0.60 | Ineligible |
| T-DM1 | Varices oesophageal | breast_cancer_primary | 18 | 19.65 (11.62-33.24) | 9.69 | Eligible |
| T-DM1 | Dyspepsia | breast_cancer_primary | 17 | 0.57 (0.35-0.92) | 0.41 | Ineligible |
| T-DM1 | Dyspepsia | breast_cancer_primary | 17 | 0.57 (0.35-0.92) | 0.41 | Ineligible |
| T-DM1 | Hepatitis | breast_cancer_primary | 17 | 1.95 (1.20-3.15) | 1.19 | Eligible |
| T-DM1 | Recurrent cancer | breast_cancer_primary | 17 | 5.04 (3.08-8.26) | 2.51 | Eligible |
| T-DM1 | Somnolence | breast_cancer_primary | 17 | 0.69 (0.43-1.12) | 0.49 | Ineligible |
| T-DM1 | Somnolence | breast_cancer_primary | 17 | 0.69 (0.43-1.12) | 0.49 | Ineligible |
| T-DM1 | Tachycardia | breast_cancer_primary | 17 | 0.89 (0.55-1.44) | 0.62 | Ineligible |
| T-DM1 | Tachycardia | breast_cancer_primary | 17 | 0.89 (0.55-1.44) | 0.62 | Ineligible |
| T-DM1 | Breast cancer recurrent | breast_cancer_primary | 16 | 0.85 (0.52-1.39) | 0.58 | Ineligible |
| T-DM1 | Breast cancer recurrent | breast_cancer_primary | 16 | 0.85 (0.52-1.39) | 0.58 | Ineligible |
| T-DM1 | Depression | breast_cancer_primary | 16 | 0.38 (0.23-0.62) | 0.28 | Ineligible |
| T-DM1 | Depression | breast_cancer_primary | 16 | 0.38 (0.23-0.62) | 0.28 | Ineligible |
| T-DM1 | Metastasis | breast_cancer_primary | 16 | 1.04 (0.63-1.70) | 0.70 | Ineligible |
| T-DM1 | Metastasis | breast_cancer_primary | 16 | 1.04 (0.63-1.70) | 0.70 | Ineligible |
| T-DM1 | Oedema peripheral | breast_cancer_primary | 16 | 0.53 (0.33-0.87) | 0.38 | Ineligible |
| T-DM1 | Oedema peripheral | breast_cancer_primary | 16 | 0.53 (0.33-0.87) | 0.38 | Ineligible |
| T-DM1 | Pulmonary arterial hypertension | breast_cancer_primary | 16 | 8.39 (4.99-14.12) | 4.46 | Eligible |
| T-DM1 | Pulmonary embolism | breast_cancer_primary | 16 | 0.52 (0.32-0.84) | 0.37 | Ineligible |
| T-DM1 | Pulmonary embolism | breast_cancer_primary | 16 | 0.52 (0.32-0.84) | 0.37 | Ineligible |
| T-DM1 | Tumour haemorrhage | breast_cancer_primary | 16 | 11.27 (6.63-19.16) | 6.12 | Eligible |
| T-DM1 | Acute kidney injury | breast_cancer_primary | 15 | 0.50 (0.30-0.82) | 0.36 | Ineligible |
| T-DM1 | Acute kidney injury | breast_cancer_primary | 15 | 0.50 (0.30-0.82) | 0.36 | Ineligible |
| T-DM1 | Anxiety | breast_cancer_primary | 15 | 0.15 (0.09-0.25) | 0.12 | Ineligible |
| T-DM1 | Anxiety | breast_cancer_primary | 15 | 0.15 (0.09-0.25) | 0.12 | Ineligible |
| T-DM1 | Device related infection | breast_cancer_primary | 15 | 2.64 (1.57-4.42) | 1.44 | Eligible |
| T-DM1 | Dry skin | breast_cancer_primary | 15 | 0.35 (0.21-0.58) | 0.26 | Ineligible |
| T-DM1 | Dry skin | breast_cancer_primary | 15 | 0.35 (0.21-0.58) | 0.26 | Ineligible |
| T-DM1 | Heart rate increased | breast_cancer_primary | 15 | 1.00 (0.60-1.66) | 0.67 | Ineligible |
| T-DM1 | Heart rate increased | breast_cancer_primary | 15 | 1.00 (0.60-1.66) | 0.67 | Ineligible |
| T-DM1 | Hepatopulmonary syndrome | breast_cancer_primary | 15 | 126.84 (53.75-299.34) | 21.73 | Eligible |
| T-DM1 | Memory impairment | breast_cancer_primary | 15 | 0.48 (0.29-0.79) | 0.34 | Ineligible |
| T-DM1 | Memory impairment | breast_cancer_primary | 15 | 0.48 (0.29-0.79) | 0.34 | Ineligible |
| T-DM1 | Spider naevus | breast_cancer_primary | 15 | 338.25 (97.88-1168.87) | 25.73 | Eligible |
| T-DM1 | Abdominal distension | breast_cancer_primary | 14 | 0.65 (0.39-1.11) | 0.45 | Ineligible |
| T-DM1 | Abdominal distension | breast_cancer_primary | 14 | 0.65 (0.39-1.11) | 0.45 | Ineligible |
| T-DM1 | Cholestasis | breast_cancer_primary | 14 | 1.81 (1.06-3.08) | 1.07 | Eligible |
| T-DM1 | Dysgeusia | breast_cancer_primary | 14 | 0.59 (0.35-0.99) | 0.41 | Ineligible |
| T-DM1 | Dysgeusia | breast_cancer_primary | 14 | 0.59 (0.35-0.99) | 0.41 | Ineligible |
| T-DM1 | Dysphagia | breast_cancer_primary | 14 | 0.53 (0.31-0.90) | 0.38 | Ineligible |
| T-DM1 | Dysphagia | breast_cancer_primary | 14 | 0.53 (0.31-0.90) | 0.38 | Ineligible |
| T-DM1 | Inflammation | breast_cancer_primary | 14 | 1.17 (0.69-1.99) | 0.75 | Ineligible |
| T-DM1 | Inflammation | breast_cancer_primary | 14 | 1.17 (0.69-1.99) | 0.75 | Ineligible |
| T-DM1 | Intentional product use issue | breast_cancer_primary | 14 | 1.05 (0.62-1.78) | 0.69 | Ineligible |
| T-DM1 | Intentional product use issue | breast_cancer_primary | 14 | 1.05 (0.62-1.78) | 0.69 | Ineligible |
| T-DM1 | Loss of consciousness | breast_cancer_primary | 14 | 0.93 (0.55-1.58) | 0.62 | Ineligible |
| T-DM1 | Loss of consciousness | breast_cancer_primary | 14 | 0.93 (0.55-1.58) | 0.62 | Ineligible |
| T-DM1 | Mucosal inflammation | breast_cancer_primary | 14 | 0.41 (0.24-0.70) | 0.30 | Ineligible |
| T-DM1 | Mucosal inflammation | breast_cancer_primary | 14 | 0.41 (0.24-0.70) | 0.30 | Ineligible |
| T-DM1 | Neuralgia | breast_cancer_primary | 14 | 2.05 (1.20-3.49) | 1.17 | Eligible |
| T-DM1 | Palmar-plantar erythrodysaesthesia syndrome | breast_cancer_primary | 14 | 0.33 (0.19-0.55) | 0.24 | Ineligible |
| T-DM1 | Palmar-plantar erythrodysaesthesia syndrome | breast_cancer_primary | 14 | 0.33 (0.19-0.55) | 0.24 | Ineligible |
| T-DM1 | Pancreatitis | breast_cancer_primary | 14 | 2.95 (1.72-5.04) | 1.53 | Eligible |
| T-DM1 | Pulmonary fibrosis | breast_cancer_primary | 14 | 2.72 (1.59-4.64) | 1.44 | Eligible |
| T-DM1 | Telangiectasia | breast_cancer_primary | 14 | 26.30 (14.17-48.80) | 10.91 | Eligible |
| T-DM1 | Tremor | breast_cancer_primary | 14 | 0.68 (0.40-1.15) | 0.47 | Ineligible |
| T-DM1 | Tremor | breast_cancer_primary | 14 | 0.68 (0.40-1.15) | 0.47 | Ineligible |
| T-DM1 | Urticaria | breast_cancer_primary | 14 | 0.81 (0.48-1.37) | 0.55 | Ineligible |
| T-DM1 | Urticaria | breast_cancer_primary | 14 | 0.81 (0.48-1.37) | 0.55 | Ineligible |
| T-DM1 | Venoocclusive liver disease | breast_cancer_primary | 14 | 25.59 (13.82-47.37) | 10.73 | Eligible |
| T-DM1 | Acute respiratory distress syndrome | breast_cancer_primary | 13 | 2.73 (1.56-4.75) | 1.41 | Eligible |
| T-DM1 | Blood glucose increased | breast_cancer_primary | 13 | 0.53 (0.31-0.91) | 0.37 | Ineligible |
| T-DM1 | Blood glucose increased | breast_cancer_primary | 13 | 0.53 (0.31-0.91) | 0.37 | Ineligible |
| T-DM1 | Cerebral disorder | breast_cancer_primary | 13 | 4.70 (2.67-8.25) | 2.09 | Eligible |
| T-DM1 | Condition aggravated | breast_cancer_primary | 13 | 0.39 (0.23-0.68) | 0.28 | Ineligible |
| T-DM1 | Condition aggravated | breast_cancer_primary | 13 | 0.39 (0.23-0.68) | 0.28 | Ineligible |
| T-DM1 | Confusional state | breast_cancer_primary | 13 | 0.61 (0.36-1.06) | 0.43 | Ineligible |
| T-DM1 | Confusional state | breast_cancer_primary | 13 | 0.61 (0.36-1.06) | 0.43 | Ineligible |
| T-DM1 | Extravasation | breast_cancer_primary | 13 | 10.10 (5.63-18.11) | 5.07 | Eligible |
| T-DM1 | Feeling abnormal | breast_cancer_primary | 13 | 0.36 (0.21-0.62) | 0.26 | Ineligible |
| T-DM1 | Feeling abnormal | breast_cancer_primary | 13 | 0.36 (0.21-0.62) | 0.26 | Ineligible |
| T-DM1 | Hypercalcaemia | breast_cancer_primary | 13 | 2.44 (1.40-4.24) | 1.30 | Eligible |
| T-DM1 | Metastases to lymph nodes | breast_cancer_primary | 13 | 0.75 (0.43-1.30) | 0.51 | Ineligible |
| T-DM1 | Metastases to lymph nodes | breast_cancer_primary | 13 | 0.75 (0.43-1.30) | 0.51 | Ineligible |
| T-DM1 | Neoplasm | breast_cancer_primary | 13 | 1.65 (0.95-2.87) | 0.98 | Ineligible |
| T-DM1 | Neoplasm | breast_cancer_primary | 13 | 1.65 (0.95-2.87) | 0.98 | Ineligible |
| T-DM1 | Non-cirrhotic portal hypertension | breast_cancer_primary | 13 | 46.26 (22.83-93.73) | 14.37 | Eligible |
| T-DM1 | Palpitations | breast_cancer_primary | 13 | 0.69 (0.40-1.19) | 0.47 | Ineligible |
| T-DM1 | Palpitations | breast_cancer_primary | 13 | 0.69 (0.40-1.19) | 0.47 | Ineligible |
| T-DM1 | Radiation pneumonitis | breast_cancer_primary | 13 | 5.60 (3.18-9.86) | 2.43 | Eligible |
| T-DM1 | Rectal haemorrhage | breast_cancer_primary | 13 | 1.95 (1.12-3.39) | 1.11 | Eligible |
| T-DM1 | Renal impairment | breast_cancer_primary | 13 | 0.59 (0.34-1.03) | 0.41 | Ineligible |
| T-DM1 | Renal impairment | breast_cancer_primary | 13 | 0.59 (0.34-1.03) | 0.41 | Ineligible |
| T-DM1 | Balance disorder | breast_cancer_primary | 12 | 0.90 (0.51-1.59) | 0.59 | Ineligible |
| T-DM1 | Balance disorder | breast_cancer_primary | 12 | 0.90 (0.51-1.59) | 0.59 | Ineligible |
| T-DM1 | Brain neoplasm | breast_cancer_primary | 12 | 2.72 (1.53-4.85) | 1.37 | Eligible |
| T-DM1 | Cholecystitis | breast_cancer_primary | 12 | 4.43 (2.47-7.95) | 1.92 | Eligible |
| T-DM1 | Drug resistance | breast_cancer_primary | 12 | 2.05 (1.15-3.64) | 1.12 | Eligible |
| T-DM1 | Flushing | breast_cancer_primary | 12 | 0.67 (0.38-1.18) | 0.45 | Ineligible |
| T-DM1 | Flushing | breast_cancer_primary | 12 | 0.67 (0.38-1.18) | 0.45 | Ineligible |
| T-DM1 | Haematocrit decreased | breast_cancer_primary | 12 | 1.15 (0.65-2.04) | 0.72 | Ineligible |
| T-DM1 | Haematocrit decreased | breast_cancer_primary | 12 | 1.15 (0.65-2.04) | 0.72 | Ineligible |
| T-DM1 | Liver injury | breast_cancer_primary | 12 | 1.22 (0.69-2.17) | 0.76 | Ineligible |
| T-DM1 | Liver injury | breast_cancer_primary | 12 | 1.22 (0.69-2.17) | 0.76 | Ineligible |
| T-DM1 | Lymphadenopathy | breast_cancer_primary | 12 | 0.80 (0.45-1.41) | 0.53 | Ineligible |
| T-DM1 | Lymphadenopathy | breast_cancer_primary | 12 | 0.80 (0.45-1.41) | 0.53 | Ineligible |
| T-DM1 | Monocyte count increased | breast_cancer_primary | 12 | 6.43 (3.56-11.64) | 2.68 | Eligible |
| T-DM1 | Red cell distribution width increased | breast_cancer_primary | 12 | 2.04 (1.15-3.63) | 1.12 | Eligible |
| T-DM1 | Skin toxicity | breast_cancer_primary | 12 | 1.53 (0.86-2.72) | 0.90 | Ineligible |
| T-DM1 | Skin toxicity | breast_cancer_primary | 12 | 1.53 (0.86-2.72) | 0.90 | Ineligible |
| T-DM1 | Vertigo | breast_cancer_primary | 12 | 0.93 (0.53-1.65) | 0.60 | Ineligible |
| T-DM1 | Vertigo | breast_cancer_primary | 12 | 0.93 (0.53-1.65) | 0.60 | Ineligible |
| T-DM1 | Blood creatinine increased | breast_cancer_primary | 11 | 0.40 (0.22-0.73) | 0.28 | Ineligible |
| T-DM1 | Blood creatinine increased | breast_cancer_primary | 11 | 0.40 (0.22-0.73) | 0.28 | Ineligible |
| T-DM1 | Cardiac dysfunction | breast_cancer_primary | 11 | 1.73 (0.95-3.15) | 0.97 | Ineligible |
| T-DM1 | Cardiac dysfunction | breast_cancer_primary | 11 | 1.73 (0.95-3.15) | 0.97 | Ineligible |
| T-DM1 | Cardiac failure congestive | breast_cancer_primary | 11 | 1.04 (0.57-1.89) | 0.65 | Ineligible |
| T-DM1 | Cardiac failure congestive | breast_cancer_primary | 11 | 1.04 (0.57-1.89) | 0.65 | Ineligible |
| T-DM1 | Colitis | breast_cancer_primary | 11 | 0.68 (0.37-1.23) | 0.45 | Ineligible |
| T-DM1 | Colitis | breast_cancer_primary | 11 | 0.68 (0.37-1.23) | 0.45 | Ineligible |
| T-DM1 | Disease recurrence | breast_cancer_primary | 11 | 0.86 (0.47-1.56) | 0.55 | Ineligible |
| T-DM1 | Disease recurrence | breast_cancer_primary | 11 | 0.86 (0.47-1.56) | 0.55 | Ineligible |
| T-DM1 | Encephalopathy | breast_cancer_primary | 11 | 4.50 (2.44-8.30) | 1.86 | Eligible |
| T-DM1 | Epilepsy | breast_cancer_primary | 11 | 2.31 (1.27-4.22) | 1.19 | Eligible |
| T-DM1 | Gastritis | breast_cancer_primary | 11 | 1.16 (0.64-2.10) | 0.71 | Ineligible |
| T-DM1 | Gastritis | breast_cancer_primary | 11 | 1.16 (0.64-2.10) | 0.71 | Ineligible |
| T-DM1 | Gastrointestinal disorder | breast_cancer_primary | 11 | 0.50 (0.28-0.90) | 0.35 | Ineligible |
| T-DM1 | Gastrointestinal disorder | breast_cancer_primary | 11 | 0.50 (0.28-0.90) | 0.35 | Ineligible |
| T-DM1 | Haemoptysis | breast_cancer_primary | 11 | 3.33 (1.82-6.11) | 1.52 | Eligible |
| T-DM1 | Hepatic cytolysis | breast_cancer_primary | 11 | 0.87 (0.48-1.57) | 0.56 | Ineligible |
| T-DM1 | Hepatic cytolysis | breast_cancer_primary | 11 | 0.87 (0.48-1.57) | 0.56 | Ineligible |
| T-DM1 | Hepatic encephalopathy | breast_cancer_primary | 11 | 6.19 (3.34-11.49) | 2.44 | Eligible |
| T-DM1 | Herpes zoster | breast_cancer_primary | 11 | 1.09 (0.60-1.98) | 0.68 | Ineligible |
| T-DM1 | Herpes zoster | breast_cancer_primary | 11 | 1.09 (0.60-1.98) | 0.68 | Ineligible |
| T-DM1 | Hot flush | breast_cancer_primary | 11 | 0.22 (0.12-0.40) | 0.16 | Ineligible |
| T-DM1 | Hot flush | breast_cancer_primary | 11 | 0.22 (0.12-0.40) | 0.16 | Ineligible |
| T-DM1 | Hyperglycaemia | breast_cancer_primary | 11 | 0.38 (0.21-0.69) | 0.27 | Ineligible |
| T-DM1 | Hyperglycaemia | breast_cancer_primary | 11 | 0.38 (0.21-0.69) | 0.27 | Ineligible |
| T-DM1 | Hypoxia | breast_cancer_primary | 11 | 1.11 (0.61-2.02) | 0.69 | Ineligible |
| T-DM1 | Hypoxia | breast_cancer_primary | 11 | 1.11 (0.61-2.02) | 0.69 | Ineligible |
| T-DM1 | Influenza | breast_cancer_primary | 11 | 0.59 (0.33-1.07) | 0.40 | Ineligible |
| T-DM1 | Influenza | breast_cancer_primary | 11 | 0.59 (0.33-1.07) | 0.40 | Ineligible |
| T-DM1 | Influenza like illness | breast_cancer_primary | 11 | 1.04 (0.57-1.89) | 0.65 | Ineligible |
| T-DM1 | Influenza like illness | breast_cancer_primary | 11 | 1.04 (0.57-1.89) | 0.65 | Ineligible |
| T-DM1 | Maternal exposure during pregnancy | breast_cancer_primary | 11 | 2.16 (1.18-3.94) | 1.13 | Eligible |
| T-DM1 | Pulmonary toxicity | breast_cancer_primary | 11 | 2.22 (1.21-4.04) | 1.15 | Eligible |
| T-DM1 | Amnesia | breast_cancer_primary | 10 | 1.05 (0.56-1.96) | 0.64 | Ineligible |
| T-DM1 | Amnesia | breast_cancer_primary | 10 | 1.05 (0.56-1.96) | 0.64 | Ineligible |
| T-DM1 | Anaphylactic reaction | breast_cancer_primary | 10 | 1.24 (0.66-2.32) | 0.73 | Ineligible |
| T-DM1 | Anaphylactic reaction | breast_cancer_primary | 10 | 1.24 (0.66-2.32) | 0.73 | Ineligible |
| T-DM1 | Anaphylactic shock | breast_cancer_primary | 10 | 2.11 (1.12-3.96) | 1.08 | Eligible |
| T-DM1 | Blister | breast_cancer_primary | 10 | 0.69 (0.37-1.29) | 0.46 | Ineligible |
| T-DM1 | Blister | breast_cancer_primary | 10 | 0.69 (0.37-1.29) | 0.46 | Ineligible |
| T-DM1 | Blood albumin decreased | breast_cancer_primary | 10 | 3.84 (2.03-7.26) | 1.60 | Eligible |
| T-DM1 | Blood sodium decreased | breast_cancer_primary | 10 | 1.82 (0.97-3.42) | 0.98 | Ineligible |
| T-DM1 | Blood sodium decreased | breast_cancer_primary | 10 | 1.82 (0.97-3.42) | 0.98 | Ineligible |
| T-DM1 | Body temperature increased | breast_cancer_primary | 10 | 2.10 (1.12-3.94) | 1.08 | Eligible |
| T-DM1 | Corneal disorder | breast_cancer_primary | 10 | 14.69 (7.41-29.12) | 6.50 | Eligible |
| T-DM1 | Haematuria | breast_cancer_primary | 10 | 2.82 (1.50-5.32) | 1.31 | Eligible |
| T-DM1 | Hepatic steatosis | breast_cancer_primary | 10 | 1.27 (0.68-2.38) | 0.75 | Ineligible |
| T-DM1 | Hepatic steatosis | breast_cancer_primary | 10 | 1.27 (0.68-2.38) | 0.75 | Ineligible |
| T-DM1 | Hypertransaminasaemia | breast_cancer_primary | 10 | 1.41 (0.75-2.64) | 0.81 | Ineligible |
| T-DM1 | Hypertransaminasaemia | breast_cancer_primary | 10 | 1.41 (0.75-2.64) | 0.81 | Ineligible |
| T-DM1 | Metastases to spine | breast_cancer_primary | 10 | 0.94 (0.50-1.76) | 0.59 | Ineligible |
| T-DM1 | Metastases to spine | breast_cancer_primary | 10 | 0.94 (0.50-1.76) | 0.59 | Ineligible |
| T-DM1 | Mouth haemorrhage | breast_cancer_primary | 10 | 4.63 (2.44-8.78) | 1.81 | Eligible |
| T-DM1 | Nervous system disorder | breast_cancer_primary | 10 | 2.11 (1.12-3.96) | 1.08 | Eligible |
| T-DM1 | Oropharyngeal pain | breast_cancer_primary | 10 | 0.44 (0.24-0.83) | 0.31 | Ineligible |
| T-DM1 | Oropharyngeal pain | breast_cancer_primary | 10 | 0.44 (0.24-0.83) | 0.31 | Ineligible |
| T-DM1 | Pulmonary hypertension | breast_cancer_primary | 10 | 2.69 (1.43-5.06) | 1.27 | Eligible |
| T-DM1 | Rhinorrhoea | breast_cancer_primary | 10 | 0.66 (0.35-1.23) | 0.44 | Ineligible |
| T-DM1 | Rhinorrhoea | breast_cancer_primary | 10 | 0.66 (0.35-1.23) | 0.44 | Ineligible |
| T-DM1 | Thrombosis | breast_cancer_primary | 10 | 0.46 (0.25-0.86) | 0.32 | Ineligible |
| T-DM1 | Thrombosis | breast_cancer_primary | 10 | 0.46 (0.25-0.86) | 0.32 | Ineligible |
| T-DM1 | White blood cell count increased | breast_cancer_primary | 10 | 1.39 (0.74-2.61) | 0.80 | Ineligible |
| T-DM1 | White blood cell count increased | breast_cancer_primary | 10 | 1.39 (0.74-2.61) | 0.80 | Ineligible |
| T-DM1 | Abdominal discomfort | breast_cancer_primary | 9 | 0.24 (0.12-0.45) | 0.17 | Ineligible |
| T-DM1 | Abdominal discomfort | breast_cancer_primary | 9 | 0.24 (0.12-0.45) | 0.17 | Ineligible |
| T-DM1 | Breast disorder | breast_cancer_primary | 9 | 3.38 (1.73-6.60) | 1.40 | Ineligible |
| T-DM1 | Breast disorder | breast_cancer_primary | 9 | 3.38 (1.73-6.60) | 1.40 | Ineligible |
| T-DM1 | Burning sensation | breast_cancer_primary | 9 | 0.68 (0.35-1.31) | 0.44 | Ineligible |
| T-DM1 | Burning sensation | breast_cancer_primary | 9 | 0.68 (0.35-1.31) | 0.44 | Ineligible |
| T-DM1 | C-reactive protein increased | breast_cancer_primary | 9 | 0.83 (0.43-1.60) | 0.52 | Ineligible |
| T-DM1 | C-reactive protein increased | breast_cancer_primary | 9 | 0.83 (0.43-1.60) | 0.52 | Ineligible |
| T-DM1 | Cataract | breast_cancer_primary | 9 | 0.67 (0.35-1.30) | 0.44 | Ineligible |
| T-DM1 | Cataract | breast_cancer_primary | 9 | 0.67 (0.35-1.30) | 0.44 | Ineligible |
| T-DM1 | Cytopenia | breast_cancer_primary | 9 | 1.08 (0.56-2.09) | 0.64 | Ineligible |
| T-DM1 | Cytopenia | breast_cancer_primary | 9 | 1.08 (0.56-2.09) | 0.64 | Ineligible |
| T-DM1 | Discomfort | breast_cancer_primary | 9 | 0.49 (0.25-0.94) | 0.33 | Ineligible |
| T-DM1 | Discomfort | breast_cancer_primary | 9 | 0.49 (0.25-0.94) | 0.33 | Ineligible |
| T-DM1 | Drug hypersensitivity | breast_cancer_primary | 9 | 0.69 (0.36-1.32) | 0.44 | Ineligible |
| T-DM1 | Drug hypersensitivity | breast_cancer_primary | 9 | 0.69 (0.36-1.32) | 0.44 | Ineligible |
| T-DM1 | Haemorrhage intracranial | breast_cancer_primary | 9 | 14.47 (7.04-29.75) | 6.09 | Ineligible |
| T-DM1 | Haemorrhage intracranial | breast_cancer_primary | 9 | 14.47 (7.04-29.75) | 6.09 | Ineligible |
| T-DM1 | Lower respiratory tract infection | breast_cancer_primary | 9 | 1.24 (0.64-2.39) | 0.71 | Ineligible |
| T-DM1 | Lower respiratory tract infection | breast_cancer_primary | 9 | 1.24 (0.64-2.39) | 0.71 | Ineligible |
| T-DM1 | Lung infiltration | breast_cancer_primary | 9 | 1.98 (1.02-3.85) | 1.00 | Ineligible |
| T-DM1 | Lung infiltration | breast_cancer_primary | 9 | 1.98 (1.02-3.85) | 1.00 | Ineligible |
| T-DM1 | Lymphoedema | breast_cancer_primary | 9 | 0.61 (0.31-1.17) | 0.40 | Ineligible |
| T-DM1 | Lymphoedema | breast_cancer_primary | 9 | 0.61 (0.31-1.17) | 0.40 | Ineligible |
| T-DM1 | Mean platelet volume decreased | breast_cancer_primary | 9 | 8.21 (4.11-16.42) | 2.90 | Ineligible |
| T-DM1 | Mean platelet volume decreased | breast_cancer_primary | 9 | 8.21 (4.11-16.42) | 2.90 | Ineligible |
| T-DM1 | Ocular hyperaemia | breast_cancer_primary | 9 | 2.27 (1.17-4.42) | 1.10 | Ineligible |
| T-DM1 | Ocular hyperaemia | breast_cancer_primary | 9 | 2.27 (1.17-4.42) | 1.10 | Ineligible |
| T-DM1 | Polyneuropathy | breast_cancer_primary | 9 | 0.58 (0.30-1.12) | 0.39 | Ineligible |
| T-DM1 | Polyneuropathy | breast_cancer_primary | 9 | 0.58 (0.30-1.12) | 0.39 | Ineligible |
| T-DM1 | Radiation skin injury | breast_cancer_primary | 9 | 4.08 (2.08-8.00) | 1.58 | Ineligible |
| T-DM1 | Radiation skin injury | breast_cancer_primary | 9 | 4.08 (2.08-8.00) | 1.58 | Ineligible |
| T-DM1 | Septic shock | breast_cancer_primary | 9 | 0.91 (0.47-1.75) | 0.56 | Ineligible |
| T-DM1 | Septic shock | breast_cancer_primary | 9 | 0.91 (0.47-1.75) | 0.56 | Ineligible |
| T-DM1 | Speech disorder | breast_cancer_primary | 9 | 0.99 (0.51-1.91) | 0.60 | Ineligible |
| T-DM1 | Speech disorder | breast_cancer_primary | 9 | 0.99 (0.51-1.91) | 0.60 | Ineligible |
| T-DM1 | Stress | breast_cancer_primary | 9 | 0.75 (0.39-1.44) | 0.48 | Ineligible |
| T-DM1 | Stress | breast_cancer_primary | 9 | 0.75 (0.39-1.44) | 0.48 | Ineligible |
| T-DM1 | Syncope | breast_cancer_primary | 9 | 0.52 (0.27-1.00) | 0.35 | Ineligible |
| T-DM1 | Syncope | breast_cancer_primary | 9 | 0.52 (0.27-1.00) | 0.35 | Ineligible |
| T-DM1 | Toxicity to various agents | breast_cancer_primary | 9 | 0.43 (0.23-0.84) | 0.30 | Ineligible |
| T-DM1 | Toxicity to various agents | breast_cancer_primary | 9 | 0.43 (0.23-0.84) | 0.30 | Ineligible |
| T-DM1 | Upper respiratory tract infection | breast_cancer_primary | 9 | 1.08 (0.56-2.09) | 0.64 | Ineligible |
| T-DM1 | Upper respiratory tract infection | breast_cancer_primary | 9 | 1.08 (0.56-2.09) | 0.64 | Ineligible |
| T-DM1 | Arrhythmia | breast_cancer_primary | 8 | 0.83 (0.41-1.67) | 0.51 | Ineligible |
| T-DM1 | Arrhythmia | breast_cancer_primary | 8 | 0.83 (0.41-1.67) | 0.51 | Ineligible |
| T-DM1 | Blindness | breast_cancer_primary | 8 | 1.36 (0.67-2.74) | 0.74 | Ineligible |
| T-DM1 | Blindness | breast_cancer_primary | 8 | 1.36 (0.67-2.74) | 0.74 | Ineligible |
| T-DM1 | Blood creatinine decreased | breast_cancer_primary | 8 | 2.93 (1.44-5.96) | 1.23 | Ineligible |
| T-DM1 | Blood creatinine decreased | breast_cancer_primary | 8 | 2.93 (1.44-5.96) | 1.23 | Ineligible |
| T-DM1 | Blood magnesium decreased | breast_cancer_primary | 8 | 1.81 (0.90-3.66) | 0.91 | Ineligible |
| T-DM1 | Blood magnesium decreased | breast_cancer_primary | 8 | 1.81 (0.90-3.66) | 0.91 | Ineligible |
| T-DM1 | Blood pressure decreased | breast_cancer_primary | 8 | 0.79 (0.39-1.58) | 0.49 | Ineligible |
| T-DM1 | Blood pressure decreased | breast_cancer_primary | 8 | 0.79 (0.39-1.58) | 0.49 | Ineligible |
| T-DM1 | Blood pressure diastolic decreased | breast_cancer_primary | 8 | 4.06 (1.99-8.29) | 1.48 | Ineligible |
| T-DM1 | Blood pressure diastolic decreased | breast_cancer_primary | 8 | 4.06 (1.99-8.29) | 1.48 | Ineligible |
| T-DM1 | Brain neoplasm malignant | breast_cancer_primary | 8 | 7.01 (3.38-14.54) | 2.22 | Ineligible |
| T-DM1 | Brain neoplasm malignant | breast_cancer_primary | 8 | 7.01 (3.38-14.54) | 2.22 | Ineligible |
| T-DM1 | Cardiac arrest | breast_cancer_primary | 8 | 0.90 (0.45-1.81) | 0.54 | Ineligible |
| T-DM1 | Cardiac arrest | breast_cancer_primary | 8 | 0.90 (0.45-1.81) | 0.54 | Ineligible |
| T-DM1 | Cardiomyopathy | breast_cancer_primary | 8 | 0.89 (0.44-1.79) | 0.54 | Ineligible |
| T-DM1 | Cardiomyopathy | breast_cancer_primary | 8 | 0.89 (0.44-1.79) | 0.54 | Ineligible |
| T-DM1 | Dyspnoea exertional | breast_cancer_primary | 8 | 0.58 (0.29-1.16) | 0.38 | Ineligible |
| T-DM1 | Dyspnoea exertional | breast_cancer_primary | 8 | 0.58 (0.29-1.16) | 0.38 | Ineligible |
| T-DM1 | Femur fracture | breast_cancer_primary | 8 | 1.13 (0.56-2.27) | 0.65 | Ineligible |
| T-DM1 | Femur fracture | breast_cancer_primary | 8 | 1.13 (0.56-2.27) | 0.65 | Ineligible |
| T-DM1 | Gastric ulcer | breast_cancer_primary | 8 | 2.50 (1.23-5.06) | 1.11 | Ineligible |
| T-DM1 | Gastric ulcer | breast_cancer_primary | 8 | 2.50 (1.23-5.06) | 1.11 | Ineligible |
| T-DM1 | Gastrooesophageal reflux disease | breast_cancer_primary | 8 | 0.47 (0.23-0.94) | 0.32 | Ineligible |
| T-DM1 | Gastrooesophageal reflux disease | breast_cancer_primary | 8 | 0.47 (0.23-0.94) | 0.32 | Ineligible |
| T-DM1 | Hyperhidrosis | breast_cancer_primary | 8 | 0.45 (0.22-0.90) | 0.30 | Ineligible |
| T-DM1 | Hyperhidrosis | breast_cancer_primary | 8 | 0.45 (0.22-0.90) | 0.30 | Ineligible |
| T-DM1 | Hypocalcaemia | breast_cancer_primary | 8 | 0.93 (0.46-1.87) | 0.56 | Ineligible |
| T-DM1 | Hypocalcaemia | breast_cancer_primary | 8 | 0.93 (0.46-1.87) | 0.56 | Ineligible |
| T-DM1 | Illness | breast_cancer_primary | 8 | 0.31 (0.15-0.61) | 0.21 | Ineligible |
| T-DM1 | Illness | breast_cancer_primary | 8 | 0.31 (0.15-0.61) | 0.21 | Ineligible |
| T-DM1 | Lung infection | breast_cancer_primary | 8 | 3.14 (1.54-6.38) | 1.28 | Ineligible |
| T-DM1 | Lung infection | breast_cancer_primary | 8 | 3.14 (1.54-6.38) | 1.28 | Ineligible |
| T-DM1 | Lymphangiosis carcinomatosa | breast_cancer_primary | 8 | 2.81 (1.39-5.71) | 1.20 | Ineligible |
| T-DM1 | Lymphangiosis carcinomatosa | breast_cancer_primary | 8 | 2.81 (1.39-5.71) | 1.20 | Ineligible |
| T-DM1 | Metastatic neoplasm | breast_cancer_primary | 8 | 2.13 (1.05-4.32) | 1.01 | Ineligible |
| T-DM1 | Metastatic neoplasm | breast_cancer_primary | 8 | 2.13 (1.05-4.32) | 1.01 | Ineligible |
| T-DM1 | Migraine | breast_cancer_primary | 8 | 0.90 (0.45-1.80) | 0.54 | Ineligible |
| T-DM1 | Migraine | breast_cancer_primary | 8 | 0.90 (0.45-1.80) | 0.54 | Ineligible |
| T-DM1 | Neurotoxicity | breast_cancer_primary | 8 | 1.07 (0.53-2.16) | 0.62 | Ineligible |
| T-DM1 | Neurotoxicity | breast_cancer_primary | 8 | 1.07 (0.53-2.16) | 0.62 | Ineligible |
| T-DM1 | Peripheral sensory neuropathy | breast_cancer_primary | 8 | 1.16 (0.58-2.34) | 0.66 | Ineligible |
| T-DM1 | Peripheral sensory neuropathy | breast_cancer_primary | 8 | 1.16 (0.58-2.34) | 0.66 | Ineligible |
| T-DM1 | Petechiae | breast_cancer_primary | 8 | 5.57 (2.71-11.46) | 1.83 | Ineligible |
| T-DM1 | Petechiae | breast_cancer_primary | 8 | 5.57 (2.71-11.46) | 1.83 | Ineligible |
| T-DM1 | Tumour lysis syndrome | breast_cancer_primary | 8 | 3.07 (1.51-6.23) | 1.26 | Ineligible |
| T-DM1 | Tumour lysis syndrome | breast_cancer_primary | 8 | 3.07 (1.51-6.23) | 1.26 | Ineligible |
| T-DM1 | Visual acuity reduced | breast_cancer_primary | 8 | 1.80 (0.89-3.63) | 0.90 | Ineligible |
| T-DM1 | Visual acuity reduced | breast_cancer_primary | 8 | 1.80 (0.89-3.63) | 0.90 | Ineligible |
| T-DM1 | Acute hepatic failure | breast_cancer_primary | 7 | 1.54 (0.73-3.25) | 0.78 | Ineligible |
| T-DM1 | Acute hepatic failure | breast_cancer_primary | 7 | 1.54 (0.73-3.25) | 0.78 | Ineligible |
| T-DM1 | Aphasia | breast_cancer_primary | 7 | 1.54 (0.73-3.27) | 0.78 | Ineligible |
| T-DM1 | Aphasia | breast_cancer_primary | 7 | 1.54 (0.73-3.27) | 0.78 | Ineligible |
| T-DM1 | Bone marrow failure | breast_cancer_primary | 7 | 0.27 (0.13-0.56) | 0.19 | Ineligible |
| T-DM1 | Bone marrow failure | breast_cancer_primary | 7 | 0.27 (0.13-0.56) | 0.19 | Ineligible |
| T-DM1 | Bronchitis | breast_cancer_primary | 7 | 0.67 (0.32-1.40) | 0.42 | Ineligible |
| T-DM1 | Bronchitis | breast_cancer_primary | 7 | 0.67 (0.32-1.40) | 0.42 | Ineligible |
| T-DM1 | Carcinoembryonic antigen increased | breast_cancer_primary | 7 | 1.34 (0.63-2.83) | 0.71 | Ineligible |
| T-DM1 | Carcinoembryonic antigen increased | breast_cancer_primary | 7 | 1.34 (0.63-2.83) | 0.71 | Ineligible |
| T-DM1 | Central nervous system lesion | breast_cancer_primary | 7 | 1.88 (0.89-3.99) | 0.89 | Ineligible |
| T-DM1 | Central nervous system lesion | breast_cancer_primary | 7 | 1.88 (0.89-3.99) | 0.89 | Ineligible |
| T-DM1 | Cognitive disorder | breast_cancer_primary | 7 | 0.76 (0.36-1.61) | 0.47 | Ineligible |
| T-DM1 | Cognitive disorder | breast_cancer_primary | 7 | 0.76 (0.36-1.61) | 0.47 | Ineligible |
| T-DM1 | Conjunctivitis | breast_cancer_primary | 7 | 1.79 (0.84-3.79) | 0.86 | Ineligible |
| T-DM1 | Conjunctivitis | breast_cancer_primary | 7 | 1.79 (0.84-3.79) | 0.86 | Ineligible |
| T-DM1 | Diabetes mellitus | breast_cancer_primary | 7 | 0.42 (0.20-0.89) | 0.28 | Ineligible |
| T-DM1 | Diabetes mellitus | breast_cancer_primary | 7 | 0.42 (0.20-0.89) | 0.28 | Ineligible |
| T-DM1 | Disseminated intravascular coagulation | breast_cancer_primary | 7 | 2.13 (1.00-4.52) | 0.96 | Ineligible |
| T-DM1 | Disseminated intravascular coagulation | breast_cancer_primary | 7 | 2.13 (1.00-4.52) | 0.96 | Ineligible |
| T-DM1 | Drug interaction | breast_cancer_primary | 7 | 0.36 (0.17-0.76) | 0.25 | Ineligible |
| T-DM1 | Drug interaction | breast_cancer_primary | 7 | 0.36 (0.17-0.76) | 0.25 | Ineligible |
| T-DM1 | Eye disorder | breast_cancer_primary | 7 | 1.39 (0.66-2.94) | 0.73 | Ineligible |
| T-DM1 | Eye disorder | breast_cancer_primary | 7 | 1.39 (0.66-2.94) | 0.73 | Ineligible |
| T-DM1 | Eye irritation | breast_cancer_primary | 7 | 1.97 (0.93-4.17) | 0.92 | Ineligible |
| T-DM1 | Eye irritation | breast_cancer_primary | 7 | 1.97 (0.93-4.17) | 0.92 | Ineligible |
| T-DM1 | Eye pruritus | breast_cancer_primary | 7 | 1.84 (0.87-3.89) | 0.88 | Ineligible |
| T-DM1 | Eye pruritus | breast_cancer_primary | 7 | 1.84 (0.87-3.89) | 0.88 | Ineligible |
| T-DM1 | Focal nodular hyperplasia | breast_cancer_primary | 7 | 24.87 (10.45-59.20) | 7.94 | Ineligible |
| T-DM1 | Focal nodular hyperplasia | breast_cancer_primary | 7 | 24.87 (10.45-59.20) | 7.94 | Ineligible |
| T-DM1 | Gastroenteritis | breast_cancer_primary | 7 | 1.88 (0.89-3.99) | 0.89 | Ineligible |
| T-DM1 | Gastroenteritis | breast_cancer_primary | 7 | 1.88 (0.89-3.99) | 0.89 | Ineligible |
| T-DM1 | Haematemesis | breast_cancer_primary | 7 | 1.52 (0.72-3.21) | 0.77 | Ineligible |
| T-DM1 | Haematemesis | breast_cancer_primary | 7 | 1.52 (0.72-3.21) | 0.77 | Ineligible |
| T-DM1 | Hemiplegia | breast_cancer_primary | 7 | 5.76 (2.66-12.47) | 1.72 | Ineligible |
| T-DM1 | Hemiplegia | breast_cancer_primary | 7 | 5.76 (2.66-12.47) | 1.72 | Ineligible |
| T-DM1 | Hepatic fibrosis | breast_cancer_primary | 7 | 14.32 (6.33-32.39) | 4.92 | Ineligible |
| T-DM1 | Hepatic fibrosis | breast_cancer_primary | 7 | 14.32 (6.33-32.39) | 4.92 | Ineligible |
| T-DM1 | Hepatic pain | breast_cancer_primary | 7 | 3.07 (1.44-6.55) | 1.19 | Ineligible |
| T-DM1 | Hepatic pain | breast_cancer_primary | 7 | 3.07 (1.44-6.55) | 1.19 | Ineligible |
| T-DM1 | Intracranial tumour haemorrhage | breast_cancer_primary | 7 | 33.76 (13.62-83.68) | 9.36 | Ineligible |
| T-DM1 | Intracranial tumour haemorrhage | breast_cancer_primary | 7 | 33.76 (13.62-83.68) | 9.36 | Ineligible |
| T-DM1 | Joint swelling | breast_cancer_primary | 7 | 0.55 (0.26-1.17) | 0.36 | Ineligible |
| T-DM1 | Joint swelling | breast_cancer_primary | 7 | 0.55 (0.26-1.17) | 0.36 | Ineligible |
| T-DM1 | Mean cell haemoglobin concentration decreased | breast_cancer_primary | 7 | 5.56 (2.57-12.02) | 1.68 | Ineligible |
| T-DM1 | Mean cell haemoglobin concentration decreased | breast_cancer_primary | 7 | 5.56 (2.57-12.02) | 1.68 | Ineligible |
| T-DM1 | Metastases to skin | breast_cancer_primary | 7 | 1.27 (0.60-2.69) | 0.69 | Ineligible |
| T-DM1 | Metastases to skin | breast_cancer_primary | 7 | 1.27 (0.60-2.69) | 0.69 | Ineligible |
| T-DM1 | Multiple organ dysfunction syndrome | breast_cancer_primary | 7 | 0.96 (0.46-2.03) | 0.56 | Ineligible |
| T-DM1 | Multiple organ dysfunction syndrome | breast_cancer_primary | 7 | 0.96 (0.46-2.03) | 0.56 | Ineligible |
| T-DM1 | Musculoskeletal stiffness | breast_cancer_primary | 7 | 0.58 (0.27-1.21) | 0.37 | Ineligible |
| T-DM1 | Musculoskeletal stiffness | breast_cancer_primary | 7 | 0.58 (0.27-1.21) | 0.37 | Ineligible |
| T-DM1 | Osteonecrosis of jaw | breast_cancer_primary | 7 | 0.16 (0.08-0.34) | 0.12 | Ineligible |
| T-DM1 | Osteonecrosis of jaw | breast_cancer_primary | 7 | 0.16 (0.08-0.34) | 0.12 | Ineligible |
| T-DM1 | Osteoporosis | breast_cancer_primary | 7 | 0.75 (0.36-1.59) | 0.46 | Ineligible |
| T-DM1 | Osteoporosis | breast_cancer_primary | 7 | 0.75 (0.36-1.59) | 0.46 | Ineligible |
| T-DM1 | Pancytopenia | breast_cancer_primary | 7 | 0.27 (0.13-0.57) | 0.19 | Ineligible |
| T-DM1 | Pancytopenia | breast_cancer_primary | 7 | 0.27 (0.13-0.57) | 0.19 | Ineligible |
| T-DM1 | Pneumocystis jirovecii pneumonia | breast_cancer_primary | 7 | 0.90 (0.43-1.90) | 0.53 | Ineligible |
| T-DM1 | Pneumocystis jirovecii pneumonia | breast_cancer_primary | 7 | 0.90 (0.43-1.90) | 0.53 | Ineligible |
| T-DM1 | Pulmonary mass | breast_cancer_primary | 7 | 0.68 (0.32-1.43) | 0.42 | Ineligible |
| T-DM1 | Pulmonary mass | breast_cancer_primary | 7 | 0.68 (0.32-1.43) | 0.42 | Ineligible |
| T-DM1 | Respiratory tract infection | breast_cancer_primary | 7 | 1.49 (0.70-3.15) | 0.76 | Ineligible |
| T-DM1 | Respiratory tract infection | breast_cancer_primary | 7 | 1.49 (0.70-3.15) | 0.76 | Ineligible |
| T-DM1 | Retinal detachment | breast_cancer_primary | 7 | 3.05 (1.43-6.50) | 1.18 | Ineligible |
| T-DM1 | Retinal detachment | breast_cancer_primary | 7 | 3.05 (1.43-6.50) | 1.18 | Ineligible |
| T-DM1 | Skin disorder | breast_cancer_primary | 7 | 0.69 (0.33-1.46) | 0.43 | Ineligible |
| T-DM1 | Skin disorder | breast_cancer_primary | 7 | 0.69 (0.33-1.46) | 0.43 | Ineligible |
| T-DM1 | Skin exfoliation | breast_cancer_primary | 7 | 0.31 (0.15-0.64) | 0.21 | Ineligible |
| T-DM1 | Skin exfoliation | breast_cancer_primary | 7 | 0.31 (0.15-0.64) | 0.21 | Ineligible |
| T-DM1 | Sleep disorder | breast_cancer_primary | 7 | 0.49 (0.23-1.03) | 0.32 | Ineligible |
| T-DM1 | Sleep disorder | breast_cancer_primary | 7 | 0.49 (0.23-1.03) | 0.32 | Ineligible |
| T-DM1 | Suspected counterfeit product | breast_cancer_primary | 7 | 22.50 (9.56-52.96) | 7.45 | Ineligible |
| T-DM1 | Suspected counterfeit product | breast_cancer_primary | 7 | 22.50 (9.56-52.96) | 7.45 | Ineligible |
| T-DM1 | Tumour marker increased | breast_cancer_primary | 7 | 0.33 (0.16-0.69) | 0.22 | Ineligible |
| T-DM1 | Tumour marker increased | breast_cancer_primary | 7 | 0.33 (0.16-0.69) | 0.22 | Ineligible |
| T-DM1 | Upper gastrointestinal haemorrhage | breast_cancer_primary | 7 | 4.68 (2.17-10.07) | 1.50 | Ineligible |
| T-DM1 | Upper gastrointestinal haemorrhage | breast_cancer_primary | 7 | 4.68 (2.17-10.07) | 1.50 | Ineligible |
| T-DM1 | Urinary retention | breast_cancer_primary | 7 | 1.80 (0.85-3.81) | 0.86 | Ineligible |
| T-DM1 | Urinary retention | breast_cancer_primary | 7 | 1.80 (0.85-3.81) | 0.86 | Ineligible |
| T-DM1 | Viral infection | breast_cancer_primary | 7 | 1.24 (0.59-2.62) | 0.67 | Ineligible |
| T-DM1 | Viral infection | breast_cancer_primary | 7 | 1.24 (0.59-2.62) | 0.67 | Ineligible |
| T-DM1 | Arteriovenous malformation | breast_cancer_primary | 6 | 17.61 (7.17-43.26) | 5.31 | Ineligible |
| T-DM1 | Arteriovenous malformation | breast_cancer_primary | 6 | 17.61 (7.17-43.26) | 5.31 | Ineligible |
| T-DM1 | Asthma | breast_cancer_primary | 6 | 0.98 (0.44-2.20) | 0.55 | Ineligible |
| T-DM1 | Asthma | breast_cancer_primary | 6 | 0.98 (0.44-2.20) | 0.55 | Ineligible |
| T-DM1 | Ataxia | breast_cancer_primary | 6 | 3.32 (1.46-7.54) | 1.15 | Ineligible |
| T-DM1 | Ataxia | breast_cancer_primary | 6 | 3.32 (1.46-7.54) | 1.15 | Ineligible |
| T-DM1 | Bone disorder | breast_cancer_primary | 6 | 0.65 (0.29-1.46) | 0.40 | Ineligible |
| T-DM1 | Bone disorder | breast_cancer_primary | 6 | 0.65 (0.29-1.46) | 0.40 | Ineligible |
| T-DM1 | Breast neoplasm | breast_cancer_primary | 6 | 4.31 (1.89-9.84) | 1.32 | Ineligible |
| T-DM1 | Breast neoplasm | breast_cancer_primary | 6 | 4.31 (1.89-9.84) | 1.32 | Ineligible |
| T-DM1 | Breast operation | breast_cancer_primary | 6 | 4.13 (1.81-9.43) | 1.29 | Ineligible |
| T-DM1 | Breast operation | breast_cancer_primary | 6 | 4.13 (1.81-9.43) | 1.29 | Ineligible |
| T-DM1 | Cardio-respiratory arrest | breast_cancer_primary | 6 | 1.27 (0.57-2.85) | 0.66 | Ineligible |
| T-DM1 | Cardio-respiratory arrest | breast_cancer_primary | 6 | 1.27 (0.57-2.85) | 0.66 | Ineligible |
| T-DM1 | Cystitis | breast_cancer_primary | 6 | 0.62 (0.28-1.39) | 0.38 | Ineligible |
| T-DM1 | Cystitis | breast_cancer_primary | 6 | 0.62 (0.28-1.39) | 0.38 | Ineligible |
| T-DM1 | Deep vein thrombosis | breast_cancer_primary | 6 | 0.44 (0.19-0.97) | 0.29 | Ineligible |
| T-DM1 | Deep vein thrombosis | breast_cancer_primary | 6 | 0.44 (0.19-0.97) | 0.29 | Ineligible |
| T-DM1 | Ear pain | breast_cancer_primary | 6 | 1.49 (0.66-3.34) | 0.73 | Ineligible |
| T-DM1 | Ear pain | breast_cancer_primary | 6 | 1.49 (0.66-3.34) | 0.73 | Ineligible |
| T-DM1 | Eye pain | breast_cancer_primary | 6 | 1.28 (0.57-2.88) | 0.66 | Ineligible |
| T-DM1 | Eye pain | breast_cancer_primary | 6 | 1.28 (0.57-2.88) | 0.66 | Ineligible |
| T-DM1 | Feeding disorder | breast_cancer_primary | 6 | 0.53 (0.24-1.19) | 0.34 | Ineligible |
| T-DM1 | Feeding disorder | breast_cancer_primary | 6 | 0.53 (0.24-1.19) | 0.34 | Ineligible |
| T-DM1 | Gastric haemorrhage | breast_cancer_primary | 6 | 4.76 (2.08-10.91) | 1.39 | Ineligible |
| T-DM1 | Gastric haemorrhage | breast_cancer_primary | 6 | 4.76 (2.08-10.91) | 1.39 | Ineligible |
| T-DM1 | Haemolysis | breast_cancer_primary | 6 | 5.70 (2.48-13.13) | 1.54 | Ineligible |
| T-DM1 | Haemolysis | breast_cancer_primary | 6 | 5.70 (2.48-13.13) | 1.54 | Ineligible |
| T-DM1 | Hyperaesthesia | breast_cancer_primary | 6 | 1.90 (0.84-4.28) | 0.85 | Ineligible |
| T-DM1 | Hyperaesthesia | breast_cancer_primary | 6 | 1.90 (0.84-4.28) | 0.85 | Ineligible |
| T-DM1 | Hyperlipidaemia | breast_cancer_primary | 6 | 2.41 (1.07-5.44) | 0.97 | Ineligible |
| T-DM1 | Hyperlipidaemia | breast_cancer_primary | 6 | 2.41 (1.07-5.44) | 0.97 | Ineligible |
| T-DM1 | Hyperpyrexia | breast_cancer_primary | 6 | 1.56 (0.69-3.51) | 0.75 | Ineligible |
| T-DM1 | Hyperpyrexia | breast_cancer_primary | 6 | 1.56 (0.69-3.51) | 0.75 | Ineligible |
| T-DM1 | Left ventricular dysfunction | breast_cancer_primary | 6 | 0.72 (0.32-1.61) | 0.43 | Ineligible |
| T-DM1 | Left ventricular dysfunction | breast_cancer_primary | 6 | 0.72 (0.32-1.61) | 0.43 | Ineligible |
| T-DM1 | Lethargy | breast_cancer_primary | 6 | 0.70 (0.31-1.56) | 0.42 | Ineligible |
| T-DM1 | Lethargy | breast_cancer_primary | 6 | 0.70 (0.31-1.56) | 0.42 | Ineligible |
| T-DM1 | Mastectomy | breast_cancer_primary | 6 | 1.70 (0.76-3.82) | 0.79 | Ineligible |
| T-DM1 | Mastectomy | breast_cancer_primary | 6 | 1.70 (0.76-3.82) | 0.79 | Ineligible |
| T-DM1 | Melaena | breast_cancer_primary | 6 | 2.20 (0.97-4.96) | 0.92 | Ineligible |
| T-DM1 | Melaena | breast_cancer_primary | 6 | 2.20 (0.97-4.96) | 0.92 | Ineligible |
| T-DM1 | Neck pain | breast_cancer_primary | 6 | 0.59 (0.26-1.31) | 0.37 | Ineligible |
| T-DM1 | Neck pain | breast_cancer_primary | 6 | 0.59 (0.26-1.31) | 0.37 | Ineligible |
| T-DM1 | Necrosis | breast_cancer_primary | 6 | 3.26 (1.44-7.41) | 1.14 | Ineligible |
| T-DM1 | Necrosis | breast_cancer_primary | 6 | 3.26 (1.44-7.41) | 1.14 | Ineligible |
| T-DM1 | Neutrophil count increased | breast_cancer_primary | 6 | 1.41 (0.63-3.17) | 0.70 | Ineligible |
| T-DM1 | Neutrophil count increased | breast_cancer_primary | 6 | 1.41 (0.63-3.17) | 0.70 | Ineligible |
| T-DM1 | Oedema | breast_cancer_primary | 6 | 0.48 (0.22-1.07) | 0.31 | Ineligible |
| T-DM1 | Oedema | breast_cancer_primary | 6 | 0.48 (0.22-1.07) | 0.31 | Ineligible |
| T-DM1 | Oesophageal varices haemorrhage | breast_cancer_primary | 6 | 9.20 (3.92-21.61) | 2.24 | Ineligible |
| T-DM1 | Oesophageal varices haemorrhage | breast_cancer_primary | 6 | 9.20 (3.92-21.61) | 2.24 | Ineligible |
| T-DM1 | Pancreatitis acute | breast_cancer_primary | 6 | 1.66 (0.74-3.73) | 0.78 | Ineligible |
| T-DM1 | Pancreatitis acute | breast_cancer_primary | 6 | 1.66 (0.74-3.73) | 0.78 | Ineligible |
| T-DM1 | Pollakiuria | breast_cancer_primary | 6 | 0.90 (0.40-2.03) | 0.52 | Ineligible |
| T-DM1 | Pollakiuria | breast_cancer_primary | 6 | 0.90 (0.40-2.03) | 0.52 | Ineligible |
| T-DM1 | Productive cough | breast_cancer_primary | 6 | 0.61 (0.27-1.36) | 0.38 | Ineligible |
| T-DM1 | Productive cough | breast_cancer_primary | 6 | 0.61 (0.27-1.36) | 0.38 | Ineligible |
| T-DM1 | Skin fissures | breast_cancer_primary | 6 | 0.76 (0.34-1.69) | 0.45 | Ineligible |
| T-DM1 | Skin fissures | breast_cancer_primary | 6 | 0.76 (0.34-1.69) | 0.45 | Ineligible |
| T-DM1 | Tenderness | breast_cancer_primary | 6 | 2.08 (0.92-4.68) | 0.89 | Ineligible |
| T-DM1 | Tenderness | breast_cancer_primary | 6 | 2.08 (0.92-4.68) | 0.89 | Ineligible |
| T-DM1 | Throat tightness | breast_cancer_primary | 6 | 1.16 (0.52-2.60) | 0.62 | Ineligible |
| T-DM1 | Throat tightness | breast_cancer_primary | 6 | 1.16 (0.52-2.60) | 0.62 | Ineligible |
| T-DM1 | Tooth disorder | breast_cancer_primary | 6 | 0.84 (0.37-1.87) | 0.49 | Ineligible |
| T-DM1 | Tooth disorder | breast_cancer_primary | 6 | 0.84 (0.37-1.87) | 0.49 | Ineligible |
| T-DM1 | Type 2 diabetes mellitus | breast_cancer_primary | 6 | 1.67 (0.74-3.75) | 0.78 | Ineligible |
| T-DM1 | Type 2 diabetes mellitus | breast_cancer_primary | 6 | 1.67 (0.74-3.75) | 0.78 | Ineligible |
| T-DM1 | Urosepsis | breast_cancer_primary | 6 | 2.13 (0.94-4.80) | 0.91 | Ineligible |
| T-DM1 | Urosepsis | breast_cancer_primary | 6 | 2.13 (0.94-4.80) | 0.91 | Ineligible |
| T-DM1 | Acute coronary syndrome | breast_cancer_primary | 5 | 2.50 (1.02-6.10) | 0.92 | Ineligible |
| T-DM1 | Acute coronary syndrome | breast_cancer_primary | 5 | 2.50 (1.02-6.10) | 0.92 | Ineligible |
| T-DM1 | Administration site extravasation | breast_cancer_primary | 5 | 5.72 (2.29-14.25) | 1.37 | Ineligible |
| T-DM1 | Administration site extravasation | breast_cancer_primary | 5 | 5.72 (2.29-14.25) | 1.37 | Ineligible |
| T-DM1 | Altered state of consciousness | breast_cancer_primary | 5 | 1.86 (0.77-4.53) | 0.79 | Ineligible |
| T-DM1 | Altered state of consciousness | breast_cancer_primary | 5 | 1.86 (0.77-4.53) | 0.79 | Ineligible |
| T-DM1 | Arthropathy | breast_cancer_primary | 5 | 0.68 (0.28-1.65) | 0.40 | Ineligible |
| T-DM1 | Arthropathy | breast_cancer_primary | 5 | 0.68 (0.28-1.65) | 0.40 | Ineligible |
| T-DM1 | Blood calcium decreased | breast_cancer_primary | 5 | 0.85 (0.35-2.05) | 0.48 | Ineligible |
| T-DM1 | Blood calcium decreased | breast_cancer_primary | 5 | 0.85 (0.35-2.05) | 0.48 | Ineligible |
| T-DM1 | Blood chloride decreased | breast_cancer_primary | 5 | 5.19 (2.09-12.89) | 1.30 | Ineligible |
| T-DM1 | Blood chloride decreased | breast_cancer_primary | 5 | 5.19 (2.09-12.89) | 1.30 | Ineligible |
| T-DM1 | Blood test abnormal | breast_cancer_primary | 5 | 0.56 (0.23-1.35) | 0.35 | Ineligible |
| T-DM1 | Blood test abnormal | breast_cancer_primary | 5 | 0.56 (0.23-1.35) | 0.35 | Ineligible |
| T-DM1 | Blood urea increased | breast_cancer_primary | 5 | 1.09 (0.45-2.63) | 0.57 | Ineligible |
| T-DM1 | Blood urea increased | breast_cancer_primary | 5 | 1.09 (0.45-2.63) | 0.57 | Ineligible |
| T-DM1 | Body temperature decreased | breast_cancer_primary | 5 | 2.41 (0.99-5.88) | 0.90 | Ineligible |
| T-DM1 | Body temperature decreased | breast_cancer_primary | 5 | 2.41 (0.99-5.88) | 0.90 | Ineligible |
| T-DM1 | Brain injury | breast_cancer_primary | 5 | 4.68 (1.89-11.60) | 1.24 | Ineligible |
| T-DM1 | Brain injury | breast_cancer_primary | 5 | 4.68 (1.89-11.60) | 1.24 | Ineligible |
| T-DM1 | Brain operation | breast_cancer_primary | 5 | 7.50 (2.97-18.90) | 1.61 | Ineligible |
| T-DM1 | Brain operation | breast_cancer_primary | 5 | 7.50 (2.97-18.90) | 1.61 | Ineligible |
| T-DM1 | Breast mass | breast_cancer_primary | 5 | 0.86 (0.35-2.07) | 0.48 | Ineligible |
| T-DM1 | Breast mass | breast_cancer_primary | 5 | 0.86 (0.35-2.07) | 0.48 | Ineligible |
| T-DM1 | Breast pain | breast_cancer_primary | 5 | 0.58 (0.24-1.40) | 0.36 | Ineligible |
| T-DM1 | Breast pain | breast_cancer_primary | 5 | 0.58 (0.24-1.40) | 0.36 | Ineligible |
| T-DM1 | Catheter site pain | breast_cancer_primary | 5 | 6.25 (2.50-15.62) | 1.44 | Ineligible |
| T-DM1 | Catheter site pain | breast_cancer_primary | 5 | 6.25 (2.50-15.62) | 1.44 | Ineligible |
| T-DM1 | Coma | breast_cancer_primary | 5 | 1.52 (0.63-3.69) | 0.70 | Ineligible |
| T-DM1 | Coma | breast_cancer_primary | 5 | 1.52 (0.63-3.69) | 0.70 | Ineligible |
| T-DM1 | Convulsion | breast_cancer_primary | 5 | 4.16 (1.69-10.28) | 1.17 | Ineligible |
| T-DM1 | Convulsion | breast_cancer_primary | 5 | 4.16 (1.69-10.28) | 1.17 | Ineligible |
| T-DM1 | Deafness | breast_cancer_primary | 5 | 0.96 (0.40-2.32) | 0.52 | Ineligible |
| T-DM1 | Deafness | breast_cancer_primary | 5 | 0.96 (0.40-2.32) | 0.52 | Ineligible |
| T-DM1 | Dermatitis acneiform | breast_cancer_primary | 5 | 1.70 (0.70-4.14) | 0.75 | Ineligible |
| T-DM1 | Dermatitis acneiform | breast_cancer_primary | 5 | 1.70 (0.70-4.14) | 0.75 | Ineligible |
| T-DM1 | Diplopia | breast_cancer_primary | 5 | 1.11 (0.46-2.68) | 0.57 | Ineligible |
| T-DM1 | Diplopia | breast_cancer_primary | 5 | 1.11 (0.46-2.68) | 0.57 | Ineligible |
| T-DM1 | Drug effect decreased | breast_cancer_primary | 5 | 6.02 (2.41-15.05) | 1.41 | Ineligible |
| T-DM1 | Drug effect decreased | breast_cancer_primary | 5 | 6.02 (2.41-15.05) | 1.41 | Ineligible |
| T-DM1 | Dysarthria | breast_cancer_primary | 5 | 1.42 (0.59-3.45) | 0.67 | Ineligible |
| T-DM1 | Dysarthria | breast_cancer_primary | 5 | 1.42 (0.59-3.45) | 0.67 | Ineligible |
| T-DM1 | Eating disorder | breast_cancer_primary | 5 | 0.45 (0.19-1.09) | 0.29 | Ineligible |
| T-DM1 | Eating disorder | breast_cancer_primary | 5 | 0.45 (0.19-1.09) | 0.29 | Ineligible |
| T-DM1 | Feeling hot | breast_cancer_primary | 5 | 0.39 (0.16-0.95) | 0.26 | Ineligible |
| T-DM1 | Feeling hot | breast_cancer_primary | 5 | 0.39 (0.16-0.95) | 0.26 | Ineligible |
| T-DM1 | Fracture | breast_cancer_primary | 5 | 0.93 (0.38-2.24) | 0.51 | Ineligible |
| T-DM1 | Fracture | breast_cancer_primary | 5 | 0.93 (0.38-2.24) | 0.51 | Ineligible |
| T-DM1 | Full blood count decreased | breast_cancer_primary | 5 | 0.21 (0.09-0.50) | 0.15 | Ineligible |
| T-DM1 | Full blood count decreased | breast_cancer_primary | 5 | 0.21 (0.09-0.50) | 0.15 | Ineligible |
| T-DM1 | Fungal infection | breast_cancer_primary | 5 | 1.18 (0.49-2.86) | 0.60 | Ineligible |
| T-DM1 | Fungal infection | breast_cancer_primary | 5 | 1.18 (0.49-2.86) | 0.60 | Ineligible |
| T-DM1 | Gastric varices | breast_cancer_primary | 5 | 28.12 (9.90-79.84) | 6.59 | Ineligible |
| T-DM1 | Gastric varices | breast_cancer_primary | 5 | 28.12 (9.90-79.84) | 6.59 | Ineligible |
| T-DM1 | Gastrointestinal toxicity | breast_cancer_primary | 5 | 1.14 (0.47-2.76) | 0.58 | Ineligible |
| T-DM1 | Gastrointestinal toxicity | breast_cancer_primary | 5 | 1.14 (0.47-2.76) | 0.58 | Ineligible |
| T-DM1 | Haemangioma | breast_cancer_primary | 5 | 6.75 (2.69-16.93) | 1.51 | Ineligible |
| T-DM1 | Haemangioma | breast_cancer_primary | 5 | 6.75 (2.69-16.93) | 1.51 | Ineligible |
| T-DM1 | Hallucination | breast_cancer_primary | 5 | 1.39 (0.57-3.38) | 0.66 | Ineligible |
| T-DM1 | Hallucination | breast_cancer_primary | 5 | 1.39 (0.57-3.38) | 0.66 | Ineligible |
| T-DM1 | Heart rate decreased | breast_cancer_primary | 5 | 1.43 (0.59-3.47) | 0.67 | Ineligible |
| T-DM1 | Heart rate decreased | breast_cancer_primary | 5 | 1.43 (0.59-3.47) | 0.67 | Ineligible |
| T-DM1 | Hepatic cyst | breast_cancer_primary | 5 | 2.07 (0.85-5.04) | 0.83 | Ineligible |
| T-DM1 | Hepatic cyst | breast_cancer_primary | 5 | 2.07 (0.85-5.04) | 0.83 | Ineligible |
| T-DM1 | Hepatic lesion | breast_cancer_primary | 5 | 0.75 (0.31-1.81) | 0.43 | Ineligible |
| T-DM1 | Hepatic lesion | breast_cancer_primary | 5 | 0.75 (0.31-1.81) | 0.43 | Ineligible |
| T-DM1 | Hepatocellular injury | breast_cancer_primary | 5 | 0.79 (0.32-1.90) | 0.45 | Ineligible |
| T-DM1 | Hepatocellular injury | breast_cancer_primary | 5 | 0.79 (0.32-1.90) | 0.45 | Ineligible |
| T-DM1 | Hepatomegaly | breast_cancer_primary | 5 | 1.37 (0.56-3.32) | 0.66 | Ineligible |
| T-DM1 | Hepatomegaly | breast_cancer_primary | 5 | 1.37 (0.56-3.32) | 0.66 | Ineligible |
| T-DM1 | Hyperuricaemia | breast_cancer_primary | 5 | 4.38 (1.77-10.83) | 1.20 | Ineligible |
| T-DM1 | Hyperuricaemia | breast_cancer_primary | 5 | 4.38 (1.77-10.83) | 1.20 | Ineligible |
| T-DM1 | Hypoacusis | breast_cancer_primary | 5 | 0.47 (0.19-1.12) | 0.30 | Ineligible |
| T-DM1 | Hypoacusis | breast_cancer_primary | 5 | 0.47 (0.19-1.12) | 0.30 | Ineligible |
| T-DM1 | Hypoalbuminaemia | breast_cancer_primary | 5 | 1.45 (0.60-3.51) | 0.68 | Ineligible |
| T-DM1 | Hypoalbuminaemia | breast_cancer_primary | 5 | 1.45 (0.60-3.51) | 0.68 | Ineligible |
| T-DM1 | Immune thrombocytopenic purpura | breast_cancer_primary | 5 | 14.67 (5.57-38.60) | 2.97 | Ineligible |
| T-DM1 | Immune thrombocytopenic purpura | breast_cancer_primary | 5 | 14.67 (5.57-38.60) | 2.97 | Ineligible |
| T-DM1 | Impaired healing | breast_cancer_primary | 5 | 0.59 (0.25-1.43) | 0.36 | Ineligible |
| T-DM1 | Impaired healing | breast_cancer_primary | 5 | 0.59 (0.25-1.43) | 0.36 | Ineligible |
| T-DM1 | Injection site extravasation | breast_cancer_primary | 5 | 4.16 (1.69-10.28) | 1.17 | Ineligible |
| T-DM1 | Injection site extravasation | breast_cancer_primary | 5 | 4.16 (1.69-10.28) | 1.17 | Ineligible |
| T-DM1 | Lipase increased | breast_cancer_primary | 5 | 4.06 (1.65-10.03) | 1.16 | Ineligible |
| T-DM1 | Lipase increased | breast_cancer_primary | 5 | 4.06 (1.65-10.03) | 1.16 | Ineligible |
| T-DM1 | Lung neoplasm | breast_cancer_primary | 5 | 3.71 (1.51-9.12) | 1.11 | Ineligible |
| T-DM1 | Lung neoplasm | breast_cancer_primary | 5 | 3.71 (1.51-9.12) | 1.11 | Ineligible |
| T-DM1 | Lymphoma | breast_cancer_primary | 5 | 3.31 (1.35-8.12) | 1.05 | Ineligible |
| T-DM1 | Lymphoma | breast_cancer_primary | 5 | 3.31 (1.35-8.12) | 1.05 | Ineligible |
| T-DM1 | Medication error | breast_cancer_primary | 5 | 2.13 (0.88-5.20) | 0.85 | Ineligible |
| T-DM1 | Medication error | breast_cancer_primary | 5 | 2.13 (0.88-5.20) | 0.85 | Ineligible |
| T-DM1 | Metastases to meninges | breast_cancer_primary | 5 | 1.02 (0.42-2.47) | 0.54 | Ineligible |
| T-DM1 | Metastases to meninges | breast_cancer_primary | 5 | 1.02 (0.42-2.47) | 0.54 | Ineligible |
| T-DM1 | Mobility decreased | breast_cancer_primary | 5 | 0.49 (0.20-1.18) | 0.31 | Ineligible |
| T-DM1 | Mobility decreased | breast_cancer_primary | 5 | 0.49 (0.20-1.18) | 0.31 | Ineligible |
| T-DM1 | Myocardial infarction | breast_cancer_primary | 5 | 0.43 (0.18-1.03) | 0.28 | Ineligible |
| T-DM1 | Myocardial infarction | breast_cancer_primary | 5 | 0.43 (0.18-1.03) | 0.28 | Ineligible |
| T-DM1 | Nephrolithiasis | breast_cancer_primary | 5 | 1.03 (0.43-2.49) | 0.55 | Ineligible |
| T-DM1 | Nephrolithiasis | breast_cancer_primary | 5 | 1.03 (0.43-2.49) | 0.55 | Ineligible |
| T-DM1 | Onychoclasis | breast_cancer_primary | 5 | 0.55 (0.23-1.33) | 0.34 | Ineligible |
| T-DM1 | Onychoclasis | breast_cancer_primary | 5 | 0.55 (0.23-1.33) | 0.34 | Ineligible |
| T-DM1 | Oral candidiasis | breast_cancer_primary | 5 | 1.33 (0.55-3.22) | 0.64 | Ineligible |
| T-DM1 | Oral candidiasis | breast_cancer_primary | 5 | 1.33 (0.55-3.22) | 0.64 | Ineligible |
| T-DM1 | Osteonecrosis | breast_cancer_primary | 5 | 0.90 (0.37-2.18) | 0.50 | Ineligible |
| T-DM1 | Osteonecrosis | breast_cancer_primary | 5 | 0.90 (0.37-2.18) | 0.50 | Ineligible |
| T-DM1 | Ovarian cyst | breast_cancer_primary | 5 | 3.07 (1.25-7.52) | 1.01 | Ineligible |
| T-DM1 | Ovarian cyst | breast_cancer_primary | 5 | 3.07 (1.25-7.52) | 1.01 | Ineligible |
| T-DM1 | Peripheral coldness | breast_cancer_primary | 5 | 2.16 (0.89-5.27) | 0.85 | Ineligible |
| T-DM1 | Peripheral coldness | breast_cancer_primary | 5 | 2.16 (0.89-5.27) | 0.85 | Ineligible |
| T-DM1 | Pneumonia bacterial | breast_cancer_primary | 5 | 2.83 (1.16-6.94) | 0.98 | Ineligible |
| T-DM1 | Pneumonia bacterial | breast_cancer_primary | 5 | 2.83 (1.16-6.94) | 0.98 | Ineligible |
| T-DM1 | Posterior reversible encephalopathy syndrome | breast_cancer_primary | 5 | 2.37 (0.97-5.80) | 0.90 | Ineligible |
| T-DM1 | Posterior reversible encephalopathy syndrome | breast_cancer_primary | 5 | 2.37 (0.97-5.80) | 0.90 | Ineligible |
| T-DM1 | Proteinuria | breast_cancer_primary | 5 | 1.50 (0.62-3.65) | 0.70 | Ineligible |
| T-DM1 | Proteinuria | breast_cancer_primary | 5 | 1.50 (0.62-3.65) | 0.70 | Ineligible |
| T-DM1 | Rash maculo-papular | breast_cancer_primary | 5 | 0.97 (0.40-2.36) | 0.52 | Ineligible |
| T-DM1 | Rash maculo-papular | breast_cancer_primary | 5 | 0.97 (0.40-2.36) | 0.52 | Ineligible |
| T-DM1 | Rib fracture | breast_cancer_primary | 5 | 1.01 (0.42-2.46) | 0.54 | Ineligible |
| T-DM1 | Rib fracture | breast_cancer_primary | 5 | 1.01 (0.42-2.46) | 0.54 | Ineligible |
| T-DM1 | Surgery | breast_cancer_primary | 5 | 0.79 (0.33-1.91) | 0.45 | Ineligible |
| T-DM1 | Surgery | breast_cancer_primary | 5 | 0.79 (0.33-1.91) | 0.45 | Ineligible |
| T-DM1 | Swelling face | breast_cancer_primary | 5 | 0.57 (0.24-1.37) | 0.35 | Ineligible |
| T-DM1 | Swelling face | breast_cancer_primary | 5 | 0.57 (0.24-1.37) | 0.35 | Ineligible |
| T-DM1 | Systemic lupus erythematosus | breast_cancer_primary | 5 | 3.24 (1.32-7.96) | 1.04 | Ineligible |
| T-DM1 | Systemic lupus erythematosus | breast_cancer_primary | 5 | 3.24 (1.32-7.96) | 1.04 | Ineligible |
| T-DM1 | Taste disorder | breast_cancer_primary | 5 | 0.37 (0.15-0.90) | 0.25 | Ineligible |
| T-DM1 | Taste disorder | breast_cancer_primary | 5 | 0.37 (0.15-0.90) | 0.25 | Ineligible |
| T-DM1 | Unevaluable event | breast_cancer_primary | 5 | 0.83 (0.34-2.00) | 0.47 | Ineligible |
| T-DM1 | Unevaluable event | breast_cancer_primary | 5 | 0.83 (0.34-2.00) | 0.47 | Ineligible |
| T-DM1 | Vaginal haemorrhage | breast_cancer_primary | 5 | 1.11 (0.46-2.68) | 0.57 | Ineligible |
| T-DM1 | Vaginal haemorrhage | breast_cancer_primary | 5 | 1.11 (0.46-2.68) | 0.57 | Ineligible |
| T-DM1 | Vascular rupture | breast_cancer_primary | 5 | 25.95 (9.25-72.83) | 6.20 | Ineligible |
| T-DM1 | Vascular rupture | breast_cancer_primary | 5 | 25.95 (9.25-72.83) | 6.20 | Ineligible |
| T-DM1 | Acne | breast_cancer_primary | 4 | 0.56 (0.21-1.51) | 0.34 | Ineligible |
| T-DM1 | Acne | breast_cancer_primary | 4 | 0.56 (0.21-1.51) | 0.34 | Ineligible |
| T-DM1 | Adrenal insufficiency | breast_cancer_primary | 4 | 0.78 (0.29-2.09) | 0.43 | Ineligible |
| T-DM1 | Adrenal insufficiency | breast_cancer_primary | 4 | 0.78 (0.29-2.09) | 0.43 | Ineligible |
| T-DM1 | Ankle fracture | breast_cancer_primary | 4 | 2.19 (0.81-5.94) | 0.79 | Ineligible |
| T-DM1 | Ankle fracture | breast_cancer_primary | 4 | 2.19 (0.81-5.94) | 0.79 | Ineligible |
| T-DM1 | Aortic valve incompetence | breast_cancer_primary | 4 | 2.57 (0.95-6.98) | 0.85 | Ineligible |
| T-DM1 | Aortic valve incompetence | breast_cancer_primary | 4 | 2.57 (0.95-6.98) | 0.85 | Ineligible |
| T-DM1 | Astigmatism | breast_cancer_primary | 4 | 9.64 (3.38-27.49) | 1.56 | Ineligible |
| T-DM1 | Astigmatism | breast_cancer_primary | 4 | 9.64 (3.38-27.49) | 1.56 | Ineligible |
| T-DM1 | Bacteraemia | breast_cancer_primary | 4 | 1.59 (0.59-4.28) | 0.67 | Ineligible |
| T-DM1 | Bacteraemia | breast_cancer_primary | 4 | 1.59 (0.59-4.28) | 0.67 | Ineligible |
| T-DM1 | Blindness unilateral | breast_cancer_primary | 4 | 3.25 (1.19-8.87) | 0.93 | Ineligible |
| T-DM1 | Blindness unilateral | breast_cancer_primary | 4 | 3.25 (1.19-8.87) | 0.93 | Ineligible |
| T-DM1 | Blood bilirubin decreased | breast_cancer_primary | 4 | 7.71 (2.74-21.70) | 1.37 | Ineligible |
| T-DM1 | Blood bilirubin decreased | breast_cancer_primary | 4 | 7.71 (2.74-21.70) | 1.37 | Ineligible |
| T-DM1 | Blood creatine phosphokinase increased | breast_cancer_primary | 4 | 1.54 (0.57-4.15) | 0.66 | Ineligible |
| T-DM1 | Blood creatine phosphokinase increased | breast_cancer_primary | 4 | 1.54 (0.57-4.15) | 0.66 | Ineligible |
| T-DM1 | Bone lesion | breast_cancer_primary | 4 | 0.53 (0.20-1.42) | 0.33 | Ineligible |
| T-DM1 | Bone lesion | breast_cancer_primary | 4 | 0.53 (0.20-1.42) | 0.33 | Ineligible |
| T-DM1 | Candida infection | breast_cancer_primary | 4 | 0.82 (0.30-2.19) | 0.44 | Ineligible |
| T-DM1 | Candida infection | breast_cancer_primary | 4 | 0.82 (0.30-2.19) | 0.44 | Ineligible |
| T-DM1 | Carbohydrate antigen 125 increased | breast_cancer_primary | 4 | 3.17 (1.16-8.66) | 0.93 | Ineligible |
| T-DM1 | Carbohydrate antigen 125 increased | breast_cancer_primary | 4 | 3.17 (1.16-8.66) | 0.93 | Ineligible |
| T-DM1 | Central nervous system necrosis | breast_cancer_primary | 4 | 29.98 (9.23-97.40) | 4.61 | Ineligible |
| T-DM1 | Central nervous system necrosis | breast_cancer_primary | 4 | 29.98 (9.23-97.40) | 4.61 | Ineligible |
| T-DM1 | Cerebral infarction | breast_cancer_primary | 4 | 1.00 (0.37-2.69) | 0.51 | Ineligible |
| T-DM1 | Cerebral infarction | breast_cancer_primary | 4 | 1.00 (0.37-2.69) | 0.51 | Ineligible |
| T-DM1 | Cholangitis | breast_cancer_primary | 4 | 2.87 (1.05-7.81) | 0.89 | Ineligible |
| T-DM1 | Cholangitis | breast_cancer_primary | 4 | 2.87 (1.05-7.81) | 0.89 | Ineligible |
| T-DM1 | Chronic gastritis | breast_cancer_primary | 4 | 4.82 (1.75-13.29) | 1.10 | Ineligible |
| T-DM1 | Chronic gastritis | breast_cancer_primary | 4 | 4.82 (1.75-13.29) | 1.10 | Ineligible |
| T-DM1 | Clostridium difficile infection | breast_cancer_primary | 4 | 0.93 (0.35-2.49) | 0.48 | Ineligible |
| T-DM1 | Clostridium difficile infection | breast_cancer_primary | 4 | 0.93 (0.35-2.49) | 0.48 | Ineligible |
| T-DM1 | Coagulopathy | breast_cancer_primary | 4 | 1.53 (0.57-4.13) | 0.66 | Ineligible |
| T-DM1 | Coagulopathy | breast_cancer_primary | 4 | 1.53 (0.57-4.13) | 0.66 | Ineligible |
| T-DM1 | Corneal deposits | breast_cancer_primary | 4 | 67.46 (16.87-269.84) | 7.79 | Ineligible |
| T-DM1 | Corneal deposits | breast_cancer_primary | 4 | 67.46 (16.87-269.84) | 7.79 | Ineligible |
| T-DM1 | Coronavirus infection | breast_cancer_primary | 4 | 2.25 (0.83-6.09) | 0.80 | Ineligible |
| T-DM1 | Coronavirus infection | breast_cancer_primary | 4 | 2.25 (0.83-6.09) | 0.80 | Ineligible |
| T-DM1 | Cystitis haemorrhagic | breast_cancer_primary | 4 | 2.00 (0.74-5.40) | 0.75 | Ineligible |
| T-DM1 | Cystitis haemorrhagic | breast_cancer_primary | 4 | 2.00 (0.74-5.40) | 0.75 | Ineligible |
| T-DM1 | Duodenal ulcer | breast_cancer_primary | 4 | 2.31 (0.85-6.25) | 0.81 | Ineligible |
| T-DM1 | Duodenal ulcer | breast_cancer_primary | 4 | 2.31 (0.85-6.25) | 0.81 | Ineligible |
| T-DM1 | Dysphonia | breast_cancer_primary | 4 | 0.30 (0.11-0.81) | 0.21 | Ineligible |
| T-DM1 | Dysphonia | breast_cancer_primary | 4 | 0.30 (0.11-0.81) | 0.21 | Ineligible |
| T-DM1 | Dysuria | breast_cancer_primary | 4 | 0.44 (0.17-1.18) | 0.28 | Ineligible |
| T-DM1 | Dysuria | breast_cancer_primary | 4 | 0.44 (0.17-1.18) | 0.28 | Ineligible |
| T-DM1 | Enteritis | breast_cancer_primary | 4 | 1.97 (0.73-5.32) | 0.75 | Ineligible |
| T-DM1 | Enteritis | breast_cancer_primary | 4 | 1.97 (0.73-5.32) | 0.75 | Ineligible |
| T-DM1 | Eosinophil count increased | breast_cancer_primary | 4 | 2.93 (1.08-7.98) | 0.90 | Ineligible |
| T-DM1 | Eosinophil count increased | breast_cancer_primary | 4 | 2.93 (1.08-7.98) | 0.90 | Ineligible |
| T-DM1 | Erysipelas | breast_cancer_primary | 4 | 1.04 (0.39-2.80) | 0.52 | Ineligible |
| T-DM1 | Erysipelas | breast_cancer_primary | 4 | 1.04 (0.39-2.80) | 0.52 | Ineligible |
| T-DM1 | Erythema multiforme | breast_cancer_primary | 4 | 1.40 (0.52-3.76) | 0.62 | Ineligible |
| T-DM1 | Erythema multiforme | breast_cancer_primary | 4 | 1.40 (0.52-3.76) | 0.62 | Ineligible |
| T-DM1 | Eye haemorrhage | breast_cancer_primary | 4 | 2.84 (1.04-7.73) | 0.88 | Ineligible |
| T-DM1 | Eye haemorrhage | breast_cancer_primary | 4 | 2.84 (1.04-7.73) | 0.88 | Ineligible |
| T-DM1 | Eye swelling | breast_cancer_primary | 4 | 0.98 (0.36-2.62) | 0.50 | Ineligible |
| T-DM1 | Eye swelling | breast_cancer_primary | 4 | 0.98 (0.36-2.62) | 0.50 | Ineligible |
| T-DM1 | Faeces discoloured | breast_cancer_primary | 4 | 1.49 (0.55-4.01) | 0.65 | Ineligible |
| T-DM1 | Faeces discoloured | breast_cancer_primary | 4 | 1.49 (0.55-4.01) | 0.65 | Ineligible |
| T-DM1 | Gait inability | breast_cancer_primary | 4 | 0.47 (0.18-1.27) | 0.30 | Ineligible |
| T-DM1 | Gait inability | breast_cancer_primary | 4 | 0.47 (0.18-1.27) | 0.30 | Ineligible |
| T-DM1 | Gastric disorder | breast_cancer_primary | 4 | 0.91 (0.34-2.44) | 0.48 | Ineligible |
| T-DM1 | Gastric disorder | breast_cancer_primary | 4 | 0.91 (0.34-2.44) | 0.48 | Ineligible |
| T-DM1 | Guillain-Barre syndrome | breast_cancer_primary | 4 | 4.65 (1.69-12.82) | 1.08 | Ineligible |
| T-DM1 | Guillain-Barre syndrome | breast_cancer_primary | 4 | 4.65 (1.69-12.82) | 1.08 | Ineligible |
| T-DM1 | HER-2 positive breast cancer | breast_cancer_primary | 4 | 4.03 (1.47-11.05) | 1.02 | Ineligible |
| T-DM1 | HER-2 positive breast cancer | breast_cancer_primary | 4 | 4.03 (1.47-11.05) | 1.02 | Ineligible |
| T-DM1 | Heavy menstrual bleeding | breast_cancer_primary | 4 | 6.27 (2.25-17.49) | 1.23 | Ineligible |
| T-DM1 | Heavy menstrual bleeding | breast_cancer_primary | 4 | 6.27 (2.25-17.49) | 1.23 | Ineligible |
| T-DM1 | Henoch-Schonlein purpura | breast_cancer_primary | 4 | 10.79 (3.75-31.03) | 1.68 | Ineligible |
| T-DM1 | Henoch-Schonlein purpura | breast_cancer_primary | 4 | 10.79 (3.75-31.03) | 1.68 | Ineligible |
| T-DM1 | Hepatic neoplasm | breast_cancer_primary | 4 | 2.12 (0.78-5.75) | 0.77 | Ineligible |
| T-DM1 | Hepatic neoplasm | breast_cancer_primary | 4 | 2.12 (0.78-5.75) | 0.77 | Ineligible |
| T-DM1 | Hydrocephalus | breast_cancer_primary | 4 | 2.90 (1.07-7.90) | 0.89 | Ineligible |
| T-DM1 | Hydrocephalus | breast_cancer_primary | 4 | 2.90 (1.07-7.90) | 0.89 | Ineligible |
| T-DM1 | Hyperammonaemia | breast_cancer_primary | 4 | 10.79 (3.75-31.03) | 1.68 | Ineligible |
| T-DM1 | Hyperammonaemia | breast_cancer_primary | 4 | 10.79 (3.75-31.03) | 1.68 | Ineligible |
| T-DM1 | Hyperkalaemia | breast_cancer_primary | 4 | 0.96 (0.36-2.58) | 0.50 | Ineligible |
| T-DM1 | Hyperkalaemia | breast_cancer_primary | 4 | 0.96 (0.36-2.58) | 0.50 | Ineligible |
| T-DM1 | Hypertensive crisis | breast_cancer_primary | 4 | 0.94 (0.35-2.51) | 0.49 | Ineligible |
| T-DM1 | Hypertensive crisis | breast_cancer_primary | 4 | 0.94 (0.35-2.51) | 0.49 | Ineligible |
| T-DM1 | Hypophosphataemia | breast_cancer_primary | 4 | 1.80 (0.67-4.86) | 0.71 | Ineligible |
| T-DM1 | Hypophosphataemia | breast_cancer_primary | 4 | 1.80 (0.67-4.86) | 0.71 | Ineligible |
| T-DM1 | Hypoproteinaemia | breast_cancer_primary | 4 | 6.58 (2.36-18.38) | 1.26 | Ineligible |
| T-DM1 | Hypoproteinaemia | breast_cancer_primary | 4 | 6.58 (2.36-18.38) | 1.26 | Ineligible |
| T-DM1 | Incorrect dose administered | breast_cancer_primary | 4 | 0.44 (0.16-1.17) | 0.28 | Ineligible |
| T-DM1 | Incorrect dose administered | breast_cancer_primary | 4 | 0.44 (0.16-1.17) | 0.28 | Ineligible |
| T-DM1 | Infusion site extravasation | breast_cancer_primary | 4 | 4.65 (1.69-12.82) | 1.08 | Ineligible |
| T-DM1 | Infusion site extravasation | breast_cancer_primary | 4 | 4.65 (1.69-12.82) | 1.08 | Ineligible |
| T-DM1 | Injury | breast_cancer_primary | 4 | 0.32 (0.12-0.85) | 0.21 | Ineligible |
| T-DM1 | Injury | breast_cancer_primary | 4 | 0.32 (0.12-0.85) | 0.21 | Ineligible |
| T-DM1 | Iron deficiency anaemia | breast_cancer_primary | 4 | 2.18 (0.80-5.89) | 0.78 | Ineligible |
| T-DM1 | Iron deficiency anaemia | breast_cancer_primary | 4 | 2.18 (0.80-5.89) | 0.78 | Ineligible |
| T-DM1 | Kidney infection | breast_cancer_primary | 4 | 1.28 (0.48-3.45) | 0.59 | Ineligible |
| T-DM1 | Kidney infection | breast_cancer_primary | 4 | 1.28 (0.48-3.45) | 0.59 | Ineligible |
| T-DM1 | Laryngeal oedema | breast_cancer_primary | 4 | 2.93 (1.08-7.98) | 0.90 | Ineligible |
| T-DM1 | Laryngeal oedema | breast_cancer_primary | 4 | 2.93 (1.08-7.98) | 0.90 | Ineligible |
| T-DM1 | Laryngitis | breast_cancer_primary | 4 | 1.44 (0.54-3.88) | 0.63 | Ineligible |
| T-DM1 | Laryngitis | breast_cancer_primary | 4 | 1.44 (0.54-3.88) | 0.63 | Ineligible |
| T-DM1 | Lower gastrointestinal haemorrhage | breast_cancer_primary | 4 | 13.49 (4.61-39.49) | 1.97 | Ineligible |
| T-DM1 | Lower gastrointestinal haemorrhage | breast_cancer_primary | 4 | 13.49 (4.61-39.49) | 1.97 | Ineligible |
| T-DM1 | Lung neoplasm malignant | breast_cancer_primary | 4 | 1.19 (0.44-3.19) | 0.57 | Ineligible |
| T-DM1 | Lung neoplasm malignant | breast_cancer_primary | 4 | 1.19 (0.44-3.19) | 0.57 | Ineligible |
| T-DM1 | Mastitis | breast_cancer_primary | 4 | 1.46 (0.54-3.93) | 0.64 | Ineligible |
| T-DM1 | Mastitis | breast_cancer_primary | 4 | 1.46 (0.54-3.93) | 0.64 | Ineligible |
| T-DM1 | Mean cell haemoglobin increased | breast_cancer_primary | 4 | 1.14 (0.43-3.07) | 0.55 | Ineligible |
| T-DM1 | Mean cell haemoglobin increased | breast_cancer_primary | 4 | 1.14 (0.43-3.07) | 0.55 | Ineligible |
| T-DM1 | Myelodysplastic syndrome | breast_cancer_primary | 4 | 1.07 (0.40-2.86) | 0.53 | Ineligible |
| T-DM1 | Myelodysplastic syndrome | breast_cancer_primary | 4 | 1.07 (0.40-2.86) | 0.53 | Ineligible |
| T-DM1 | Neurological symptom | breast_cancer_primary | 4 | 3.46 (1.27-9.45) | 0.96 | Ineligible |
| T-DM1 | Neurological symptom | breast_cancer_primary | 4 | 3.46 (1.27-9.45) | 0.96 | Ineligible |
| T-DM1 | Onycholysis | breast_cancer_primary | 4 | 0.95 (0.35-2.54) | 0.49 | Ineligible |
| T-DM1 | Onycholysis | breast_cancer_primary | 4 | 0.95 (0.35-2.54) | 0.49 | Ineligible |
| T-DM1 | Onychomadesis | breast_cancer_primary | 4 | 1.12 (0.42-3.02) | 0.55 | Ineligible |
| T-DM1 | Onychomadesis | breast_cancer_primary | 4 | 1.12 (0.42-3.02) | 0.55 | Ineligible |
| T-DM1 | Paravenous drug administration | breast_cancer_primary | 4 | 269.85 (30.15-2414.95) | 9.93 | Ineligible |
| T-DM1 | Paravenous drug administration | breast_cancer_primary | 4 | 269.85 (30.15-2414.95) | 9.93 | Ineligible |
| T-DM1 | Paronychia | breast_cancer_primary | 4 | 0.88 (0.33-2.37) | 0.47 | Ineligible |
| T-DM1 | Paronychia | breast_cancer_primary | 4 | 0.88 (0.33-2.37) | 0.47 | Ineligible |
| T-DM1 | Peripheral sensorimotor neuropathy | breast_cancer_primary | 4 | 4.65 (1.69-12.82) | 1.08 | Ineligible |
| T-DM1 | Peripheral sensorimotor neuropathy | breast_cancer_primary | 4 | 4.65 (1.69-12.82) | 1.08 | Ineligible |
| T-DM1 | Photophobia | breast_cancer_primary | 4 | 1.50 (0.56-4.04) | 0.65 | Ineligible |
| T-DM1 | Photophobia | breast_cancer_primary | 4 | 1.50 (0.56-4.04) | 0.65 | Ineligible |
| T-DM1 | Pneumothorax | breast_cancer_primary | 4 | 0.68 (0.25-1.82) | 0.39 | Ineligible |
| T-DM1 | Pneumothorax | breast_cancer_primary | 4 | 0.68 (0.25-1.82) | 0.39 | Ineligible |
| T-DM1 | Post procedural haemorrhage | breast_cancer_primary | 4 | 6.75 (2.41-18.86) | 1.28 | Ineligible |
| T-DM1 | Post procedural haemorrhage | breast_cancer_primary | 4 | 6.75 (2.41-18.86) | 1.28 | Ineligible |
| T-DM1 | Product administration error | breast_cancer_primary | 4 | 2.54 (0.94-6.91) | 0.84 | Ineligible |
| T-DM1 | Product administration error | breast_cancer_primary | 4 | 2.54 (0.94-6.91) | 0.84 | Ineligible |
| T-DM1 | Prothrombin time prolonged | breast_cancer_primary | 4 | 4.65 (1.69-12.82) | 1.08 | Ineligible |
| T-DM1 | Prothrombin time prolonged | breast_cancer_primary | 4 | 4.65 (1.69-12.82) | 1.08 | Ineligible |
| T-DM1 | Pulmonary alveolar haemorrhage | breast_cancer_primary | 4 | 4.22 (1.53-11.58) | 1.04 | Ineligible |
| T-DM1 | Pulmonary alveolar haemorrhage | breast_cancer_primary | 4 | 4.22 (1.53-11.58) | 1.04 | Ineligible |
| T-DM1 | Retching | breast_cancer_primary | 4 | 0.80 (0.30-2.14) | 0.44 | Ineligible |
| T-DM1 | Retching | breast_cancer_primary | 4 | 0.80 (0.30-2.14) | 0.44 | Ineligible |
| T-DM1 | SARS-CoV-2 test positive | breast_cancer_primary | 4 | 1.21 (0.45-3.25) | 0.57 | Ineligible |
| T-DM1 | SARS-CoV-2 test positive | breast_cancer_primary | 4 | 1.21 (0.45-3.25) | 0.57 | Ineligible |
| T-DM1 | Skin discolouration | breast_cancer_primary | 4 | 0.28 (0.10-0.74) | 0.19 | Ineligible |
| T-DM1 | Skin discolouration | breast_cancer_primary | 4 | 0.28 (0.10-0.74) | 0.19 | Ineligible |
| T-DM1 | Skin infection | breast_cancer_primary | 4 | 1.43 (0.53-3.84) | 0.63 | Ineligible |
| T-DM1 | Skin infection | breast_cancer_primary | 4 | 1.43 (0.53-3.84) | 0.63 | Ineligible |
| T-DM1 | Skin necrosis | breast_cancer_primary | 4 | 2.75 (1.01-7.48) | 0.87 | Ineligible |
| T-DM1 | Skin necrosis | breast_cancer_primary | 4 | 2.75 (1.01-7.48) | 0.87 | Ineligible |
| T-DM1 | Spinal pain | breast_cancer_primary | 4 | 0.40 (0.15-1.06) | 0.26 | Ineligible |
| T-DM1 | Spinal pain | breast_cancer_primary | 4 | 0.40 (0.15-1.06) | 0.26 | Ineligible |
| T-DM1 | Staphylococcal infection | breast_cancer_primary | 4 | 1.14 (0.43-3.07) | 0.55 | Ineligible |
| T-DM1 | Staphylococcal infection | breast_cancer_primary | 4 | 1.14 (0.43-3.07) | 0.55 | Ineligible |
| T-DM1 | Subcapsular hepatic haematoma | breast_cancer_primary | 4 | 269.85 (30.15-2414.95) | 9.93 | Ineligible |
| T-DM1 | Subcapsular hepatic haematoma | breast_cancer_primary | 4 | 269.85 (30.15-2414.95) | 9.93 | Ineligible |
| T-DM1 | Systemic inflammatory response syndrome | breast_cancer_primary | 4 | 6.00 (2.16-16.68) | 1.21 | Ineligible |
| T-DM1 | Systemic inflammatory response syndrome | breast_cancer_primary | 4 | 6.00 (2.16-16.68) | 1.21 | Ineligible |
| T-DM1 | Throat irritation | breast_cancer_primary | 4 | 0.52 (0.20-1.40) | 0.32 | Ineligible |
| T-DM1 | Throat irritation | breast_cancer_primary | 4 | 0.52 (0.20-1.40) | 0.32 | Ineligible |
| T-DM1 | Tinnitus | breast_cancer_primary | 4 | 0.70 (0.26-1.87) | 0.40 | Ineligible |
| T-DM1 | Tinnitus | breast_cancer_primary | 4 | 0.70 (0.26-1.87) | 0.40 | Ineligible |
| T-DM1 | Tooth fracture | breast_cancer_primary | 4 | 1.16 (0.43-3.11) | 0.56 | Ineligible |
| T-DM1 | Tooth fracture | breast_cancer_primary | 4 | 1.16 (0.43-3.11) | 0.56 | Ineligible |
| T-DM1 | Toothache | breast_cancer_primary | 4 | 0.51 (0.19-1.35) | 0.31 | Ineligible |
| T-DM1 | Toothache | breast_cancer_primary | 4 | 0.51 (0.19-1.35) | 0.31 | Ineligible |
| T-DM1 | Treatment failure | breast_cancer_primary | 4 | 0.52 (0.19-1.38) | 0.32 | Ineligible |
| T-DM1 | Treatment failure | breast_cancer_primary | 4 | 0.52 (0.19-1.38) | 0.32 | Ineligible |
| T-DM1 | Ulcerative keratitis | breast_cancer_primary | 4 | 5.51 (1.99-15.26) | 1.16 | Ineligible |
| T-DM1 | Ulcerative keratitis | breast_cancer_primary | 4 | 5.51 (1.99-15.26) | 1.16 | Ineligible |
| T-DM1 | Urinary tract disorder | breast_cancer_primary | 4 | 4.09 (1.49-11.22) | 1.03 | Ineligible |
| T-DM1 | Urinary tract disorder | breast_cancer_primary | 4 | 4.09 (1.49-11.22) | 1.03 | Ineligible |
| T-DM1 | Viral upper respiratory tract infection | breast_cancer_primary | 4 | 3.55 (1.30-9.71) | 0.97 | Ineligible |
| T-DM1 | Viral upper respiratory tract infection | breast_cancer_primary | 4 | 3.55 (1.30-9.71) | 0.97 | Ineligible |
| T-DM1 | Wound | breast_cancer_primary | 4 | 0.59 (0.22-1.59) | 0.35 | Ineligible |
| T-DM1 | Wound | breast_cancer_primary | 4 | 0.59 (0.22-1.59) | 0.35 | Ineligible |
| T-DM1 | Wound dehiscence | breast_cancer_primary | 4 | 3.03 (1.11-8.26) | 0.91 | Ineligible |
| T-DM1 | Wound dehiscence | breast_cancer_primary | 4 | 3.03 (1.11-8.26) | 0.91 | Ineligible |
| T-DM1 | Wrong technique in product usage process | breast_cancer_primary | 4 | 0.58 (0.22-1.54) | 0.35 | Ineligible |
| T-DM1 | Wrong technique in product usage process | breast_cancer_primary | 4 | 0.58 (0.22-1.54) | 0.35 | Ineligible |
| T-DM1 | Abasia | breast_cancer_primary | 3 | 1.62 (0.51-5.09) | 0.61 | Ineligible |
| T-DM1 | Abasia | breast_cancer_primary | 3 | 1.62 (0.51-5.09) | 0.61 | Ineligible |
| T-DM1 | Acute pulmonary oedema | breast_cancer_primary | 3 | 1.54 (0.49-4.85) | 0.60 | Ineligible |
| T-DM1 | Acute pulmonary oedema | breast_cancer_primary | 3 | 1.54 (0.49-4.85) | 0.60 | Ineligible |
| T-DM1 | Acute respiratory failure | breast_cancer_primary | 3 | 0.69 (0.22-2.16) | 0.38 | Ineligible |
| T-DM1 | Acute respiratory failure | breast_cancer_primary | 3 | 0.69 (0.22-2.16) | 0.38 | Ineligible |
| T-DM1 | Adverse drug reaction | breast_cancer_primary | 3 | 0.36 (0.11-1.11) | 0.23 | Ineligible |
| T-DM1 | Adverse drug reaction | breast_cancer_primary | 3 | 0.36 (0.11-1.11) | 0.23 | Ineligible |
| T-DM1 | Ammonia increased | breast_cancer_primary | 3 | 6.74 (2.06-22.11) | 1.04 | Ineligible |
| T-DM1 | Ammonia increased | breast_cancer_primary | 3 | 6.74 (2.06-22.11) | 1.04 | Ineligible |
| T-DM1 | Angioedema | breast_cancer_primary | 3 | 0.84 (0.27-2.62) | 0.43 | Ineligible |
| T-DM1 | Angioedema | breast_cancer_primary | 3 | 0.84 (0.27-2.62) | 0.43 | Ineligible |
| T-DM1 | Arthritis | breast_cancer_primary | 3 | 0.26 (0.08-0.80) | 0.18 | Ineligible |
| T-DM1 | Arthritis | breast_cancer_primary | 3 | 0.26 (0.08-0.80) | 0.18 | Ineligible |
| T-DM1 | Arthritis bacterial | breast_cancer_primary | 3 | 10.65 (3.15-36.00) | 1.26 | Ineligible |
| T-DM1 | Arthritis bacterial | breast_cancer_primary | 3 | 10.65 (3.15-36.00) | 1.26 | Ineligible |
| T-DM1 | Arthritis infective | breast_cancer_primary | 3 | 10.12 (3.00-34.06) | 1.23 | Ineligible |
| T-DM1 | Arthritis infective | breast_cancer_primary | 3 | 10.12 (3.00-34.06) | 1.23 | Ineligible |
| T-DM1 | Asthenopia | breast_cancer_primary | 3 | 3.02 (0.95-9.60) | 0.79 | Ineligible |
| T-DM1 | Asthenopia | breast_cancer_primary | 3 | 3.02 (0.95-9.60) | 0.79 | Ineligible |
| T-DM1 | Atrial fibrillation | breast_cancer_primary | 3 | 0.16 (0.05-0.51) | 0.12 | Ineligible |
| T-DM1 | Atrial fibrillation | breast_cancer_primary | 3 | 0.16 (0.05-0.51) | 0.12 | Ineligible |
| T-DM1 | Bacterial infection | breast_cancer_primary | 3 | 1.09 (0.35-3.42) | 0.50 | Ineligible |
| T-DM1 | Bacterial infection | breast_cancer_primary | 3 | 1.09 (0.35-3.42) | 0.50 | Ineligible |
| T-DM1 | Bacterial sepsis | breast_cancer_primary | 3 | 10.12 (3.00-34.06) | 1.23 | Ineligible |
| T-DM1 | Bacterial sepsis | breast_cancer_primary | 3 | 10.12 (3.00-34.06) | 1.23 | Ineligible |
| T-DM1 | Bile duct obstruction | breast_cancer_primary | 3 | 9.63 (2.87-32.31) | 1.21 | Ineligible |
| T-DM1 | Bile duct obstruction | breast_cancer_primary | 3 | 9.63 (2.87-32.31) | 1.21 | Ineligible |
| T-DM1 | Blood calcium increased | breast_cancer_primary | 3 | 1.04 (0.33-3.26) | 0.49 | Ineligible |
| T-DM1 | Blood calcium increased | breast_cancer_primary | 3 | 1.04 (0.33-3.26) | 0.49 | Ineligible |
| T-DM1 | Blood cholesterol increased | breast_cancer_primary | 3 | 0.44 (0.14-1.39) | 0.28 | Ineligible |
| T-DM1 | Blood cholesterol increased | breast_cancer_primary | 3 | 0.44 (0.14-1.39) | 0.28 | Ineligible |
| T-DM1 | Blood disorder | breast_cancer_primary | 3 | 1.16 (0.37-3.64) | 0.52 | Ineligible |
| T-DM1 | Blood disorder | breast_cancer_primary | 3 | 1.16 (0.37-3.64) | 0.52 | Ineligible |
| T-DM1 | Blood phosphorus increased | breast_cancer_primary | 3 | 3.49 (1.09-11.14) | 0.83 | Ineligible |
| T-DM1 | Blood phosphorus increased | breast_cancer_primary | 3 | 3.49 (1.09-11.14) | 0.83 | Ineligible |
| T-DM1 | Blood thyroid stimulating hormone increased | breast_cancer_primary | 3 | 1.48 (0.47-4.64) | 0.59 | Ineligible |
| T-DM1 | Blood thyroid stimulating hormone increased | breast_cancer_primary | 3 | 1.48 (0.47-4.64) | 0.59 | Ineligible |
| T-DM1 | Blood urea decreased | breast_cancer_primary | 3 | 5.47 (1.69-17.74) | 0.97 | Ineligible |
| T-DM1 | Blood urea decreased | breast_cancer_primary | 3 | 5.47 (1.69-17.74) | 0.97 | Ineligible |
| T-DM1 | Blood uric acid decreased | breast_cancer_primary | 3 | 5.95 (1.83-19.38) | 1.00 | Ineligible |
| T-DM1 | Blood uric acid decreased | breast_cancer_primary | 3 | 5.95 (1.83-19.38) | 1.00 | Ineligible |
| T-DM1 | Blood urine present | breast_cancer_primary | 3 | 1.29 (0.41-4.04) | 0.55 | Ineligible |
| T-DM1 | Blood urine present | breast_cancer_primary | 3 | 1.29 (0.41-4.04) | 0.55 | Ineligible |
| T-DM1 | Bone cancer | breast_cancer_primary | 3 | 0.87 (0.28-2.71) | 0.44 | Ineligible |
| T-DM1 | Bone cancer | breast_cancer_primary | 3 | 0.87 (0.28-2.71) | 0.44 | Ineligible |
| T-DM1 | Bone neoplasm | breast_cancer_primary | 3 | 3.32 (1.04-10.57) | 0.82 | Ineligible |
| T-DM1 | Bone neoplasm | breast_cancer_primary | 3 | 3.32 (1.04-10.57) | 0.82 | Ineligible |
| T-DM1 | Brain fog | breast_cancer_primary | 3 | 0.67 (0.21-2.08) | 0.37 | Ineligible |
| T-DM1 | Brain fog | breast_cancer_primary | 3 | 0.67 (0.21-2.08) | 0.37 | Ineligible |
| T-DM1 | Breast cellulitis | breast_cancer_primary | 3 | 4.13 (1.29-13.25) | 0.88 | Ineligible |
| T-DM1 | Breast cellulitis | breast_cancer_primary | 3 | 4.13 (1.29-13.25) | 0.88 | Ineligible |
| T-DM1 | Cancer pain | breast_cancer_primary | 3 | 0.77 (0.25-2.39) | 0.41 | Ineligible |
| T-DM1 | Cancer pain | breast_cancer_primary | 3 | 0.77 (0.25-2.39) | 0.41 | Ineligible |
| T-DM1 | Cardiac tamponade | breast_cancer_primary | 3 | 2.04 (0.65-6.45) | 0.68 | Ineligible |
| T-DM1 | Cardiac tamponade | breast_cancer_primary | 3 | 2.04 (0.65-6.45) | 0.68 | Ineligible |
| T-DM1 | Catheter site erythema | breast_cancer_primary | 3 | 4.50 (1.40-14.47) | 0.91 | Ineligible |
| T-DM1 | Catheter site erythema | breast_cancer_primary | 3 | 4.50 (1.40-14.47) | 0.91 | Ineligible |
| T-DM1 | Cell death | breast_cancer_primary | 3 | 1.37 (0.44-4.29) | 0.57 | Ineligible |
| T-DM1 | Cell death | breast_cancer_primary | 3 | 1.37 (0.44-4.29) | 0.57 | Ineligible |
| T-DM1 | Cholelithiasis | breast_cancer_primary | 3 | 0.47 (0.15-1.47) | 0.29 | Ineligible |
| T-DM1 | Cholelithiasis | breast_cancer_primary | 3 | 0.47 (0.15-1.47) | 0.29 | Ineligible |
| T-DM1 | Chromaturia | breast_cancer_primary | 3 | 0.72 (0.23-2.24) | 0.39 | Ineligible |
| T-DM1 | Chromaturia | breast_cancer_primary | 3 | 0.72 (0.23-2.24) | 0.39 | Ineligible |
| T-DM1 | Chronic myeloid leukaemia | breast_cancer_primary | 3 | 3.97 (1.24-12.71) | 0.87 | Ineligible |
| T-DM1 | Chronic myeloid leukaemia | breast_cancer_primary | 3 | 3.97 (1.24-12.71) | 0.87 | Ineligible |
| T-DM1 | Cutaneous lupus erythematosus | breast_cancer_primary | 3 | 2.89 (0.91-9.18) | 0.78 | Ineligible |
| T-DM1 | Cutaneous lupus erythematosus | breast_cancer_primary | 3 | 2.89 (0.91-9.18) | 0.78 | Ineligible |
| T-DM1 | Delirium | breast_cancer_primary | 3 | 0.90 (0.29-2.81) | 0.45 | Ineligible |
| T-DM1 | Delirium | breast_cancer_primary | 3 | 0.90 (0.29-2.81) | 0.45 | Ineligible |
| T-DM1 | Dementia | breast_cancer_primary | 3 | 0.78 (0.25-2.43) | 0.41 | Ineligible |
| T-DM1 | Dementia | breast_cancer_primary | 3 | 0.78 (0.25-2.43) | 0.41 | Ineligible |
| T-DM1 | Demyelination | breast_cancer_primary | 3 | 9.20 (2.75-30.74) | 1.18 | Ineligible |
| T-DM1 | Demyelination | breast_cancer_primary | 3 | 9.20 (2.75-30.74) | 1.18 | Ineligible |
| T-DM1 | Dental caries | breast_cancer_primary | 3 | 1.02 (0.33-3.19) | 0.48 | Ineligible |
| T-DM1 | Dental caries | breast_cancer_primary | 3 | 1.02 (0.33-3.19) | 0.48 | Ineligible |
| T-DM1 | Depressed level of consciousness | breast_cancer_primary | 3 | 0.90 (0.29-2.81) | 0.45 | Ineligible |
| T-DM1 | Depressed level of consciousness | breast_cancer_primary | 3 | 0.90 (0.29-2.81) | 0.45 | Ineligible |
| T-DM1 | Device related sepsis | breast_cancer_primary | 3 | 5.78 (1.78-18.80) | 0.99 | Ineligible |
| T-DM1 | Device related sepsis | breast_cancer_primary | 3 | 5.78 (1.78-18.80) | 0.99 | Ineligible |
| T-DM1 | Disturbance in attention | breast_cancer_primary | 3 | 0.43 (0.14-1.32) | 0.27 | Ineligible |
| T-DM1 | Disturbance in attention | breast_cancer_primary | 3 | 0.43 (0.14-1.32) | 0.27 | Ineligible |
| T-DM1 | Diverticulitis | breast_cancer_primary | 3 | 0.57 (0.18-1.76) | 0.33 | Ineligible |
| T-DM1 | Diverticulitis | breast_cancer_primary | 3 | 0.57 (0.18-1.76) | 0.33 | Ineligible |
| T-DM1 | Eczema | breast_cancer_primary | 3 | 0.52 (0.17-1.61) | 0.31 | Ineligible |
| T-DM1 | Eczema | breast_cancer_primary | 3 | 0.52 (0.17-1.61) | 0.31 | Ineligible |
| T-DM1 | Electrocardiogram QT prolonged | breast_cancer_primary | 3 | 0.17 (0.06-0.54) | 0.13 | Ineligible |
| T-DM1 | Electrocardiogram QT prolonged | breast_cancer_primary | 3 | 0.17 (0.06-0.54) | 0.13 | Ineligible |
| T-DM1 | Electrolyte imbalance | breast_cancer_primary | 3 | 0.73 (0.23-2.28) | 0.39 | Ineligible |
| T-DM1 | Electrolyte imbalance | breast_cancer_primary | 3 | 0.73 (0.23-2.28) | 0.39 | Ineligible |
| T-DM1 | Endocarditis | breast_cancer_primary | 3 | 3.55 (1.11-11.34) | 0.84 | Ineligible |
| T-DM1 | Endocarditis | breast_cancer_primary | 3 | 3.55 (1.11-11.34) | 0.84 | Ineligible |
| T-DM1 | Exercise tolerance decreased | breast_cancer_primary | 3 | 3.21 (1.01-10.23) | 0.81 | Ineligible |
| T-DM1 | Exercise tolerance decreased | breast_cancer_primary | 3 | 3.21 (1.01-10.23) | 0.81 | Ineligible |
| T-DM1 | Face oedema | breast_cancer_primary | 3 | 0.82 (0.26-2.56) | 0.42 | Ineligible |
| T-DM1 | Face oedema | breast_cancer_primary | 3 | 0.82 (0.26-2.56) | 0.42 | Ineligible |
| T-DM1 | Febrile infection | breast_cancer_primary | 3 | 2.89 (0.91-9.18) | 0.78 | Ineligible |
| T-DM1 | Febrile infection | breast_cancer_primary | 3 | 2.89 (0.91-9.18) | 0.78 | Ineligible |
| T-DM1 | Feeling cold | breast_cancer_primary | 3 | 0.37 (0.12-1.15) | 0.24 | Ineligible |
| T-DM1 | Feeling cold | breast_cancer_primary | 3 | 0.37 (0.12-1.15) | 0.24 | Ineligible |
| T-DM1 | Flank pain | breast_cancer_primary | 3 | 1.17 (0.37-3.66) | 0.52 | Ineligible |
| T-DM1 | Flank pain | breast_cancer_primary | 3 | 1.17 (0.37-3.66) | 0.52 | Ineligible |
| T-DM1 | Folliculitis | breast_cancer_primary | 3 | 2.25 (0.71-7.10) | 0.71 | Ineligible |
| T-DM1 | Folliculitis | breast_cancer_primary | 3 | 2.25 (0.71-7.10) | 0.71 | Ineligible |
| T-DM1 | Food poisoning | breast_cancer_primary | 3 | 2.97 (0.94-9.46) | 0.79 | Ineligible |
| T-DM1 | Food poisoning | breast_cancer_primary | 3 | 2.97 (0.94-9.46) | 0.79 | Ineligible |
| T-DM1 | Full blood count abnormal | breast_cancer_primary | 3 | 0.26 (0.08-0.80) | 0.18 | Ineligible |
| T-DM1 | Full blood count abnormal | breast_cancer_primary | 3 | 0.26 (0.08-0.80) | 0.18 | Ineligible |
| T-DM1 | Gastric antral vascular ectasia | breast_cancer_primary | 3 | 16.86 (4.76-59.77) | 1.58 | Ineligible |
| T-DM1 | Gastric antral vascular ectasia | breast_cancer_primary | 3 | 16.86 (4.76-59.77) | 1.58 | Ineligible |
| T-DM1 | Gastric ulcer haemorrhage | breast_cancer_primary | 3 | 5.32 (1.64-17.25) | 0.96 | Ineligible |
| T-DM1 | Gastric ulcer haemorrhage | breast_cancer_primary | 3 | 5.32 (1.64-17.25) | 0.96 | Ineligible |
| T-DM1 | Gastroenteritis viral | breast_cancer_primary | 3 | 1.31 (0.42-4.12) | 0.56 | Ineligible |
| T-DM1 | Gastroenteritis viral | breast_cancer_primary | 3 | 1.31 (0.42-4.12) | 0.56 | Ineligible |
| T-DM1 | Generalised oedema | breast_cancer_primary | 3 | 0.87 (0.28-2.71) | 0.44 | Ineligible |
| T-DM1 | Generalised oedema | breast_cancer_primary | 3 | 0.87 (0.28-2.71) | 0.44 | Ineligible |
| T-DM1 | Haematoma | breast_cancer_primary | 3 | 0.65 (0.21-2.03) | 0.37 | Ineligible |
| T-DM1 | Haematoma | breast_cancer_primary | 3 | 0.65 (0.21-2.03) | 0.37 | Ineligible |
| T-DM1 | Haematotoxicity | breast_cancer_primary | 3 | 0.29 (0.09-0.91) | 0.20 | Ineligible |
| T-DM1 | Haematotoxicity | breast_cancer_primary | 3 | 0.29 (0.09-0.91) | 0.20 | Ineligible |
| T-DM1 | Hepatic congestion | breast_cancer_primary | 3 | 67.44 (13.61-334.27) | 3.47 | Ineligible |
| T-DM1 | Hepatic congestion | breast_cancer_primary | 3 | 67.44 (13.61-334.27) | 3.47 | Ineligible |
| T-DM1 | Hepatocellular carcinoma | breast_cancer_primary | 3 | 22.48 (6.08-83.07) | 1.84 | Ineligible |
| T-DM1 | Hepatocellular carcinoma | breast_cancer_primary | 3 | 22.48 (6.08-83.07) | 1.84 | Ineligible |
| T-DM1 | Hip fracture | breast_cancer_primary | 3 | 0.53 (0.17-1.65) | 0.32 | Ineligible |
| T-DM1 | Hip fracture | breast_cancer_primary | 3 | 0.53 (0.17-1.65) | 0.32 | Ineligible |
| T-DM1 | Hypermetropia | breast_cancer_primary | 3 | 15.56 (4.43-54.64) | 1.52 | Ineligible |
| T-DM1 | Hypermetropia | breast_cancer_primary | 3 | 15.56 (4.43-54.64) | 1.52 | Ineligible |
| T-DM1 | Hypoaesthesia oral | breast_cancer_primary | 3 | 1.00 (0.32-3.12) | 0.48 | Ineligible |
| T-DM1 | Hypoaesthesia oral | breast_cancer_primary | 3 | 1.00 (0.32-3.12) | 0.48 | Ineligible |
| T-DM1 | Hypomagnesaemia | breast_cancer_primary | 3 | 0.75 (0.24-2.35) | 0.40 | Ineligible |
| T-DM1 | Hypomagnesaemia | breast_cancer_primary | 3 | 0.75 (0.24-2.35) | 0.40 | Ineligible |
| T-DM1 | Hysterectomy | breast_cancer_primary | 3 | 4.30 (1.34-13.83) | 0.89 | Ineligible |
| T-DM1 | Hysterectomy | breast_cancer_primary | 3 | 4.30 (1.34-13.83) | 0.89 | Ineligible |
| T-DM1 | Immune thrombocytopenia | breast_cancer_primary | 3 | 1.81 (0.57-5.69) | 0.65 | Ineligible |
| T-DM1 | Immune thrombocytopenia | breast_cancer_primary | 3 | 1.81 (0.57-5.69) | 0.65 | Ineligible |
| T-DM1 | Immunosuppression | breast_cancer_primary | 3 | 1.14 (0.36-3.58) | 0.52 | Ineligible |
| T-DM1 | Immunosuppression | breast_cancer_primary | 3 | 1.14 (0.36-3.58) | 0.52 | Ineligible |
| T-DM1 | Infectious pleural effusion | breast_cancer_primary | 3 | 5.47 (1.69-17.74) | 0.97 | Ineligible |
| T-DM1 | Infectious pleural effusion | breast_cancer_primary | 3 | 5.47 (1.69-17.74) | 0.97 | Ineligible |
| T-DM1 | Intestinal obstruction | breast_cancer_primary | 3 | 0.56 (0.18-1.75) | 0.33 | Ineligible |
| T-DM1 | Intestinal obstruction | breast_cancer_primary | 3 | 0.56 (0.18-1.75) | 0.33 | Ineligible |
| T-DM1 | Ischaemic stroke | breast_cancer_primary | 3 | 0.44 (0.14-1.36) | 0.28 | Ineligible |
| T-DM1 | Ischaemic stroke | breast_cancer_primary | 3 | 0.44 (0.14-1.36) | 0.28 | Ineligible |
| T-DM1 | Joint injury | breast_cancer_primary | 3 | 1.28 (0.41-4.01) | 0.55 | Ineligible |
| T-DM1 | Joint injury | breast_cancer_primary | 3 | 1.28 (0.41-4.01) | 0.55 | Ineligible |
| T-DM1 | Joint stiffness | breast_cancer_primary | 3 | 0.45 (0.14-1.39) | 0.28 | Ineligible |
| T-DM1 | Joint stiffness | breast_cancer_primary | 3 | 0.45 (0.14-1.39) | 0.28 | Ineligible |
| T-DM1 | Keratitis | breast_cancer_primary | 3 | 1.87 (0.59-5.90) | 0.66 | Ineligible |
| T-DM1 | Keratitis | breast_cancer_primary | 3 | 1.87 (0.59-5.90) | 0.66 | Ineligible |
| T-DM1 | Leukoencephalopathy | breast_cancer_primary | 3 | 2.73 (0.86-8.67) | 0.76 | Ineligible |
| T-DM1 | Leukoencephalopathy | breast_cancer_primary | 3 | 2.73 (0.86-8.67) | 0.76 | Ineligible |
| T-DM1 | Lymphadenopathy mediastinal | breast_cancer_primary | 3 | 1.49 (0.47-4.67) | 0.59 | Ineligible |
| T-DM1 | Lymphadenopathy mediastinal | breast_cancer_primary | 3 | 1.49 (0.47-4.67) | 0.59 | Ineligible |
| T-DM1 | Lymphangitis | breast_cancer_primary | 3 | 3.21 (1.01-10.23) | 0.81 | Ineligible |
| T-DM1 | Lymphangitis | breast_cancer_primary | 3 | 3.21 (1.01-10.23) | 0.81 | Ineligible |
| T-DM1 | Lymphopenia | breast_cancer_primary | 3 | 0.47 (0.15-1.46) | 0.29 | Ineligible |
| T-DM1 | Lymphopenia | breast_cancer_primary | 3 | 0.47 (0.15-1.46) | 0.29 | Ineligible |
| T-DM1 | Malignant pleural effusion | breast_cancer_primary | 3 | 0.83 (0.26-2.58) | 0.43 | Ineligible |
| T-DM1 | Malignant pleural effusion | breast_cancer_primary | 3 | 0.83 (0.26-2.58) | 0.43 | Ineligible |
| T-DM1 | Mass | breast_cancer_primary | 3 | 0.60 (0.19-1.88) | 0.35 | Ineligible |
| T-DM1 | Mass | breast_cancer_primary | 3 | 0.60 (0.19-1.88) | 0.35 | Ineligible |
| T-DM1 | Maternal exposure before pregnancy | breast_cancer_primary | 3 | 7.78 (2.35-25.72) | 1.10 | Ineligible |
| T-DM1 | Maternal exposure before pregnancy | breast_cancer_primary | 3 | 7.78 (2.35-25.72) | 1.10 | Ineligible |
| T-DM1 | Mean cell haemoglobin decreased | breast_cancer_primary | 3 | 3.61 (1.13-11.55) | 0.84 | Ineligible |
| T-DM1 | Mean cell haemoglobin decreased | breast_cancer_primary | 3 | 3.61 (1.13-11.55) | 0.84 | Ineligible |
| T-DM1 | Menorrhagia | breast_cancer_primary | 3 | 5.19 (1.60-16.79) | 0.95 | Ineligible |
| T-DM1 | Menorrhagia | breast_cancer_primary | 3 | 5.19 (1.60-16.79) | 0.95 | Ineligible |
| T-DM1 | Mental status changes | breast_cancer_primary | 3 | 1.11 (0.35-3.48) | 0.51 | Ineligible |
| T-DM1 | Mental status changes | breast_cancer_primary | 3 | 1.11 (0.35-3.48) | 0.51 | Ineligible |
| T-DM1 | Metastases to spinal cord | breast_cancer_primary | 3 | 5.47 (1.69-17.74) | 0.97 | Ineligible |
| T-DM1 | Metastases to spinal cord | breast_cancer_primary | 3 | 5.47 (1.69-17.74) | 0.97 | Ineligible |
| T-DM1 | Mitral valve incompetence | breast_cancer_primary | 3 | 0.56 (0.18-1.73) | 0.33 | Ineligible |
| T-DM1 | Mitral valve incompetence | breast_cancer_primary | 3 | 0.56 (0.18-1.73) | 0.33 | Ineligible |
| T-DM1 | Motor dysfunction | breast_cancer_primary | 3 | 1.84 (0.58-5.79) | 0.65 | Ineligible |
| T-DM1 | Motor dysfunction | breast_cancer_primary | 3 | 1.84 (0.58-5.79) | 0.65 | Ineligible |
| T-DM1 | Multi-organ failure | breast_cancer_primary | 3 | 2.66 (0.84-8.44) | 0.76 | Ineligible |
| T-DM1 | Multi-organ failure | breast_cancer_primary | 3 | 2.66 (0.84-8.44) | 0.76 | Ineligible |
| T-DM1 | Muscle twitching | breast_cancer_primary | 3 | 1.48 (0.47-4.64) | 0.59 | Ineligible |
| T-DM1 | Muscle twitching | breast_cancer_primary | 3 | 1.48 (0.47-4.64) | 0.59 | Ineligible |
| T-DM1 | Musculoskeletal chest pain | breast_cancer_primary | 3 | 0.29 (0.09-0.91) | 0.20 | Ineligible |
| T-DM1 | Musculoskeletal chest pain | breast_cancer_primary | 3 | 0.29 (0.09-0.91) | 0.20 | Ineligible |
| T-DM1 | Myopia | breast_cancer_primary | 3 | 4.13 (1.29-13.25) | 0.88 | Ineligible |
| T-DM1 | Myopia | breast_cancer_primary | 3 | 4.13 (1.29-13.25) | 0.88 | Ineligible |
| T-DM1 | Myositis | breast_cancer_primary | 3 | 1.14 (0.36-3.58) | 0.52 | Ineligible |
| T-DM1 | Myositis | breast_cancer_primary | 3 | 1.14 (0.36-3.58) | 0.52 | Ineligible |
| T-DM1 | Nail disorder | breast_cancer_primary | 3 | 0.24 (0.08-0.75) | 0.17 | Ineligible |
| T-DM1 | Nail disorder | breast_cancer_primary | 3 | 0.24 (0.08-0.75) | 0.17 | Ineligible |
| T-DM1 | Nasal congestion | breast_cancer_primary | 3 | 0.43 (0.14-1.35) | 0.27 | Ineligible |
| T-DM1 | Nasal congestion | breast_cancer_primary | 3 | 0.43 (0.14-1.35) | 0.27 | Ineligible |
| T-DM1 | Nephrotic syndrome | breast_cancer_primary | 3 | 2.63 (0.83-8.33) | 0.75 | Ineligible |
| T-DM1 | Nephrotic syndrome | breast_cancer_primary | 3 | 2.63 (0.83-8.33) | 0.75 | Ineligible |
| T-DM1 | Night sweats | breast_cancer_primary | 3 | 0.40 (0.13-1.24) | 0.26 | Ineligible |
| T-DM1 | Night sweats | breast_cancer_primary | 3 | 0.40 (0.13-1.24) | 0.26 | Ineligible |
| T-DM1 | No therapeutic response | breast_cancer_primary | 3 | 2.73 (0.86-8.67) | 0.76 | Ineligible |
| T-DM1 | No therapeutic response | breast_cancer_primary | 3 | 2.73 (0.86-8.67) | 0.76 | Ineligible |
| T-DM1 | Nodule | breast_cancer_primary | 3 | 0.67 (0.21-2.09) | 0.37 | Ineligible |
| T-DM1 | Nodule | breast_cancer_primary | 3 | 0.67 (0.21-2.09) | 0.37 | Ineligible |
| T-DM1 | Optic neuritis | breast_cancer_primary | 3 | 3.43 (1.07-10.94) | 0.83 | Ineligible |
| T-DM1 | Optic neuritis | breast_cancer_primary | 3 | 3.43 (1.07-10.94) | 0.83 | Ineligible |
| T-DM1 | Oral herpes | breast_cancer_primary | 3 | 0.71 (0.23-2.21) | 0.39 | Ineligible |
| T-DM1 | Oral herpes | breast_cancer_primary | 3 | 0.71 (0.23-2.21) | 0.39 | Ineligible |
| T-DM1 | Organising pneumonia | breast_cancer_primary | 3 | 0.58 (0.19-1.81) | 0.34 | Ineligible |
| T-DM1 | Organising pneumonia | breast_cancer_primary | 3 | 0.58 (0.19-1.81) | 0.34 | Ineligible |
| T-DM1 | Osteoarthritis | breast_cancer_primary | 3 | 0.37 (0.12-1.15) | 0.24 | Ineligible |
| T-DM1 | Osteoarthritis | breast_cancer_primary | 3 | 0.37 (0.12-1.15) | 0.24 | Ineligible |
| T-DM1 | Otitis media | breast_cancer_primary | 3 | 6.98 (2.12-22.91) | 1.06 | Ineligible |
| T-DM1 | Otitis media | breast_cancer_primary | 3 | 6.98 (2.12-22.91) | 1.06 | Ineligible |
| T-DM1 | Pain of skin | breast_cancer_primary | 3 | 0.67 (0.21-2.09) | 0.37 | Ineligible |
| T-DM1 | Pain of skin | breast_cancer_primary | 3 | 0.67 (0.21-2.09) | 0.37 | Ineligible |
| T-DM1 | Pallor | breast_cancer_primary | 3 | 0.67 (0.22-2.10) | 0.37 | Ineligible |
| T-DM1 | Pallor | breast_cancer_primary | 3 | 0.67 (0.22-2.10) | 0.37 | Ineligible |
| T-DM1 | Performance status decreased | breast_cancer_primary | 3 | 1.48 (0.47-4.64) | 0.59 | Ineligible |
| T-DM1 | Performance status decreased | breast_cancer_primary | 3 | 1.48 (0.47-4.64) | 0.59 | Ineligible |
| T-DM1 | Periorbital oedema | breast_cancer_primary | 3 | 3.11 (0.98-9.91) | 0.80 | Ineligible |
| T-DM1 | Periorbital oedema | breast_cancer_primary | 3 | 3.11 (0.98-9.91) | 0.80 | Ineligible |
| T-DM1 | Pharyngitis | breast_cancer_primary | 3 | 1.04 (0.33-3.26) | 0.49 | Ineligible |
| T-DM1 | Pharyngitis | breast_cancer_primary | 3 | 1.04 (0.33-3.26) | 0.49 | Ineligible |
| T-DM1 | Platelet count abnormal | breast_cancer_primary | 3 | 1.12 (0.36-3.50) | 0.51 | Ineligible |
| T-DM1 | Platelet count abnormal | breast_cancer_primary | 3 | 1.12 (0.36-3.50) | 0.51 | Ineligible |
| T-DM1 | Platelet count increased | breast_cancer_primary | 3 | 0.98 (0.31-3.05) | 0.47 | Ineligible |
| T-DM1 | Platelet count increased | breast_cancer_primary | 3 | 0.98 (0.31-3.05) | 0.47 | Ineligible |
| T-DM1 | Pneumonia viral | breast_cancer_primary | 3 | 6.13 (1.88-20.00) | 1.01 | Ineligible |
| T-DM1 | Pneumonia viral | breast_cancer_primary | 3 | 6.13 (1.88-20.00) | 1.01 | Ineligible |
| T-DM1 | Postmastectomy lymphoedema syndrome | breast_cancer_primary | 3 | 67.44 (13.61-334.27) | 3.47 | Ineligible |
| T-DM1 | Postmastectomy lymphoedema syndrome | breast_cancer_primary | 3 | 67.44 (13.61-334.27) | 3.47 | Ineligible |
| T-DM1 | Premature delivery | breast_cancer_primary | 3 | 1.54 (0.49-4.85) | 0.60 | Ineligible |
| T-DM1 | Premature delivery | breast_cancer_primary | 3 | 1.54 (0.49-4.85) | 0.60 | Ineligible |
| T-DM1 | Product preparation error | breast_cancer_primary | 3 | 3.11 (0.98-9.91) | 0.80 | Ineligible |
| T-DM1 | Product preparation error | breast_cancer_primary | 3 | 3.11 (0.98-9.91) | 0.80 | Ineligible |
| T-DM1 | Psychotic disorder | breast_cancer_primary | 3 | 1.27 (0.41-3.99) | 0.55 | Ineligible |
| T-DM1 | Psychotic disorder | breast_cancer_primary | 3 | 1.27 (0.41-3.99) | 0.55 | Ineligible |
| T-DM1 | Pulmonary haemorrhage | breast_cancer_primary | 3 | 4.70 (1.46-15.17) | 0.92 | Ineligible |
| T-DM1 | Pulmonary haemorrhage | breast_cancer_primary | 3 | 4.70 (1.46-15.17) | 0.92 | Ineligible |
| T-DM1 | Purpura | breast_cancer_primary | 3 | 2.53 (0.80-8.01) | 0.74 | Ineligible |
| T-DM1 | Purpura | breast_cancer_primary | 3 | 2.53 (0.80-8.01) | 0.74 | Ineligible |
| T-DM1 | Radiation injury | breast_cancer_primary | 3 | 2.09 (0.66-6.58) | 0.69 | Ineligible |
| T-DM1 | Radiation injury | breast_cancer_primary | 3 | 2.09 (0.66-6.58) | 0.69 | Ineligible |
| T-DM1 | Rash pustular | breast_cancer_primary | 3 | 1.71 (0.54-5.39) | 0.63 | Ineligible |
| T-DM1 | Rash pustular | breast_cancer_primary | 3 | 1.71 (0.54-5.39) | 0.63 | Ineligible |
| T-DM1 | Renal failure acute | breast_cancer_primary | 3 | 1.77 (0.56-5.59) | 0.64 | Ineligible |
| T-DM1 | Renal failure acute | breast_cancer_primary | 3 | 1.77 (0.56-5.59) | 0.64 | Ineligible |
| T-DM1 | Respiratory arrest | breast_cancer_primary | 3 | 1.87 (0.59-5.90) | 0.66 | Ineligible |
| T-DM1 | Respiratory arrest | breast_cancer_primary | 3 | 1.87 (0.59-5.90) | 0.66 | Ineligible |
| T-DM1 | Respiratory disorder | breast_cancer_primary | 3 | 0.60 (0.19-1.86) | 0.34 | Ineligible |
| T-DM1 | Respiratory disorder | breast_cancer_primary | 3 | 0.60 (0.19-1.86) | 0.34 | Ineligible |
| T-DM1 | Respiratory distress | breast_cancer_primary | 3 | 0.64 (0.20-1.99) | 0.36 | Ineligible |
| T-DM1 | Respiratory distress | breast_cancer_primary | 3 | 0.64 (0.20-1.99) | 0.36 | Ineligible |
| T-DM1 | Rheumatoid arthritis | breast_cancer_primary | 3 | 1.00 (0.32-3.12) | 0.48 | Ineligible |
| T-DM1 | Rheumatoid arthritis | breast_cancer_primary | 3 | 1.00 (0.32-3.12) | 0.48 | Ineligible |
| T-DM1 | Scab | breast_cancer_primary | 3 | 1.40 (0.45-4.41) | 0.57 | Ineligible |
| T-DM1 | Scab | breast_cancer_primary | 3 | 1.40 (0.45-4.41) | 0.57 | Ineligible |
| T-DM1 | Scratch | breast_cancer_primary | 3 | 2.11 (0.67-6.65) | 0.69 | Ineligible |
| T-DM1 | Scratch | breast_cancer_primary | 3 | 2.11 (0.67-6.65) | 0.69 | Ineligible |
| T-DM1 | Sensory disturbance | breast_cancer_primary | 3 | 0.78 (0.25-2.45) | 0.41 | Ineligible |
| T-DM1 | Sensory disturbance | breast_cancer_primary | 3 | 0.78 (0.25-2.45) | 0.41 | Ineligible |
| T-DM1 | Serum ferritin decreased | breast_cancer_primary | 3 | 15.56 (4.43-54.64) | 1.52 | Ineligible |
| T-DM1 | Serum ferritin decreased | breast_cancer_primary | 3 | 15.56 (4.43-54.64) | 1.52 | Ineligible |
| T-DM1 | Sinusitis | breast_cancer_primary | 3 | 0.28 (0.09-0.86) | 0.19 | Ineligible |
| T-DM1 | Sinusitis | breast_cancer_primary | 3 | 0.28 (0.09-0.86) | 0.19 | Ineligible |
| T-DM1 | Sjogren's syndrome | breast_cancer_primary | 3 | 2.27 (0.72-7.18) | 0.71 | Ineligible |
| T-DM1 | Sjogren's syndrome | breast_cancer_primary | 3 | 2.27 (0.72-7.18) | 0.71 | Ineligible |
| T-DM1 | Skin burning sensation | breast_cancer_primary | 3 | 0.79 (0.25-2.47) | 0.41 | Ineligible |
| T-DM1 | Skin burning sensation | breast_cancer_primary | 3 | 0.79 (0.25-2.47) | 0.41 | Ineligible |
| T-DM1 | Skin irritation | breast_cancer_primary | 3 | 0.90 (0.29-2.82) | 0.45 | Ineligible |
| T-DM1 | Skin irritation | breast_cancer_primary | 3 | 0.90 (0.29-2.82) | 0.45 | Ineligible |
| T-DM1 | Skin lesion | breast_cancer_primary | 3 | 0.36 (0.12-1.12) | 0.24 | Ineligible |
| T-DM1 | Skin lesion | breast_cancer_primary | 3 | 0.36 (0.12-1.12) | 0.24 | Ineligible |
| T-DM1 | Subdural haematoma | breast_cancer_primary | 3 | 3.75 (1.17-11.99) | 0.85 | Ineligible |
| T-DM1 | Subdural haematoma | breast_cancer_primary | 3 | 3.75 (1.17-11.99) | 0.85 | Ineligible |
| T-DM1 | Subileus | breast_cancer_primary | 3 | 3.49 (1.09-11.14) | 0.83 | Ineligible |
| T-DM1 | Subileus | breast_cancer_primary | 3 | 3.49 (1.09-11.14) | 0.83 | Ineligible |
| T-DM1 | Therapeutic product effect decreased | breast_cancer_primary | 3 | 2.63 (0.83-8.33) | 0.75 | Ineligible |
| T-DM1 | Therapeutic product effect decreased | breast_cancer_primary | 3 | 2.63 (0.83-8.33) | 0.75 | Ineligible |
| T-DM1 | Therapeutic response decreased | breast_cancer_primary | 3 | 0.61 (0.19-1.89) | 0.35 | Ineligible |
| T-DM1 | Therapeutic response decreased | breast_cancer_primary | 3 | 0.61 (0.19-1.89) | 0.35 | Ineligible |
| T-DM1 | Therapy non-responder | breast_cancer_primary | 3 | 0.56 (0.18-1.73) | 0.33 | Ineligible |
| T-DM1 | Therapy non-responder | breast_cancer_primary | 3 | 0.56 (0.18-1.73) | 0.33 | Ineligible |
| T-DM1 | Therapy partial responder | breast_cancer_primary | 3 | 0.33 (0.11-1.03) | 0.22 | Ineligible |
| T-DM1 | Therapy partial responder | breast_cancer_primary | 3 | 0.33 (0.11-1.03) | 0.22 | Ineligible |
| T-DM1 | Thermal burn | breast_cancer_primary | 3 | 2.02 (0.64-6.38) | 0.68 | Ineligible |
| T-DM1 | Thermal burn | breast_cancer_primary | 3 | 2.02 (0.64-6.38) | 0.68 | Ineligible |
| T-DM1 | Thrombosis in device | breast_cancer_primary | 3 | 8.09 (2.44-26.81) | 1.12 | Ineligible |
| T-DM1 | Thrombosis in device | breast_cancer_primary | 3 | 8.09 (2.44-26.81) | 1.12 | Ineligible |
| T-DM1 | Tooth loss | breast_cancer_primary | 3 | 0.93 (0.30-2.91) | 0.46 | Ineligible |
| T-DM1 | Tooth loss | breast_cancer_primary | 3 | 0.93 (0.30-2.91) | 0.46 | Ineligible |
| T-DM1 | Transient ischaemic attack | breast_cancer_primary | 3 | 0.78 (0.25-2.45) | 0.41 | Ineligible |
| T-DM1 | Transient ischaemic attack | breast_cancer_primary | 3 | 0.78 (0.25-2.45) | 0.41 | Ineligible |
| T-DM1 | Ulcer | breast_cancer_primary | 3 | 0.83 (0.27-2.59) | 0.43 | Ineligible |
| T-DM1 | Ulcer | breast_cancer_primary | 3 | 0.83 (0.27-2.59) | 0.43 | Ineligible |
| T-DM1 | Upper-airway cough syndrome | breast_cancer_primary | 3 | 2.20 (0.70-6.95) | 0.70 | Ineligible |
| T-DM1 | Upper-airway cough syndrome | breast_cancer_primary | 3 | 2.20 (0.70-6.95) | 0.70 | Ineligible |
| T-DM1 | Varicose vein | breast_cancer_primary | 3 | 1.61 (0.51-5.05) | 0.61 | Ineligible |
| T-DM1 | Varicose vein | breast_cancer_primary | 3 | 1.61 (0.51-5.05) | 0.61 | Ineligible |
| T-DM1 | Vascular device infection | breast_cancer_primary | 3 | 1.03 (0.33-3.23) | 0.49 | Ineligible |
| T-DM1 | Vascular device infection | breast_cancer_primary | 3 | 1.03 (0.33-3.23) | 0.49 | Ineligible |
| T-DM1 | Vasculitis | breast_cancer_primary | 3 | 1.34 (0.43-4.20) | 0.56 | Ineligible |
| T-DM1 | Vasculitis | breast_cancer_primary | 3 | 1.34 (0.43-4.20) | 0.56 | Ineligible |
| T-DM1 | Venous thrombosis | breast_cancer_primary | 3 | 1.84 (0.58-5.79) | 0.65 | Ineligible |
| T-DM1 | Venous thrombosis | breast_cancer_primary | 3 | 1.84 (0.58-5.79) | 0.65 | Ineligible |
| T-DM1 | Ventricular extrasystoles | breast_cancer_primary | 3 | 1.12 (0.36-3.52) | 0.51 | Ineligible |
| T-DM1 | Ventricular extrasystoles | breast_cancer_primary | 3 | 1.12 (0.36-3.52) | 0.51 | Ineligible |
| T-DM1 | Ventricular fibrillation | breast_cancer_primary | 3 | 2.32 (0.74-7.35) | 0.72 | Ineligible |
| T-DM1 | Ventricular fibrillation | breast_cancer_primary | 3 | 2.32 (0.74-7.35) | 0.72 | Ineligible |
| T-DM1 | Ventricular tachycardia | breast_cancer_primary | 3 | 1.82 (0.58-5.74) | 0.65 | Ineligible |
| T-DM1 | Ventricular tachycardia | breast_cancer_primary | 3 | 1.82 (0.58-5.74) | 0.65 | Ineligible |
| T-DM1 | Vitamin D deficiency | breast_cancer_primary | 3 | 1.20 (0.38-3.77) | 0.53 | Ineligible |
| T-DM1 | Vitamin D deficiency | breast_cancer_primary | 3 | 1.20 (0.38-3.77) | 0.53 | Ineligible |
| T-DM1 | Weight fluctuation | breast_cancer_primary | 3 | 0.64 (0.20-1.99) | 0.36 | Ineligible |
| T-DM1 | Weight fluctuation | breast_cancer_primary | 3 | 0.64 (0.20-1.99) | 0.36 | Ineligible |
| T-DXd | Nausea | breast_cancer_primary | 795 | 1.74 (1.61-1.87) | 1.52 | Eligible |
| T-DXd | Death | breast_cancer_primary | 700 | 1.82 (1.68-1.97) | 1.59 | Eligible |
| T-DXd | Interstitial lung disease | breast_cancer_primary | 631 | 11.86 (10.83-13.00) | 8.18 | Eligible |
| T-DXd | Disease progression | breast_cancer_primary | 529 | 2.82 (2.57-3.09) | 2.38 | Eligible |
| T-DXd | Fatigue | breast_cancer_primary | 528 | 0.91 (0.83-0.99) | 0.85 | Ineligible |
| T-DXd | Fatigue | breast_cancer_primary | 528 | 0.91 (0.83-0.99) | 0.85 | Ineligible |
| T-DXd | Vomiting | breast_cancer_primary | 359 | 1.35 (1.21-1.51) | 1.21 | Eligible |
| T-DXd | Diarrhoea | breast_cancer_primary | 328 | 0.59 (0.53-0.66) | 0.57 | Ineligible |
| T-DXd | Diarrhoea | breast_cancer_primary | 328 | 0.59 (0.53-0.66) | 0.57 | Ineligible |
| T-DXd | Off label use | breast_cancer_primary | 304 | 1.10 (0.98-1.24) | 0.99 | Ineligible |
| T-DXd | Off label use | breast_cancer_primary | 304 | 1.10 (0.98-1.24) | 0.99 | Ineligible |
| T-DXd | Pneumonitis | breast_cancer_primary | 258 | 5.18 (4.54-5.91) | 4.12 | Eligible |
| T-DXd | Myelosuppression | breast_cancer_primary | 255 | 1.99 (1.75-2.26) | 1.70 | Eligible |
| T-DXd | Asthenia | breast_cancer_primary | 240 | 0.98 (0.86-1.12) | 0.88 | Ineligible |
| T-DXd | Asthenia | breast_cancer_primary | 240 | 0.98 (0.86-1.12) | 0.88 | Ineligible |
| T-DXd | Alopecia | breast_cancer_primary | 238 | 0.34 (0.30-0.39) | 0.34 | Ineligible |
| T-DXd | Alopecia | breast_cancer_primary | 238 | 0.34 (0.30-0.39) | 0.34 | Ineligible |
| T-DXd | Dyspnoea | breast_cancer_primary | 209 | 0.83 (0.72-0.95) | 0.75 | Ineligible |
| T-DXd | Dyspnoea | breast_cancer_primary | 209 | 0.83 (0.72-0.95) | 0.75 | Ineligible |
| T-DXd | Decreased appetite | breast_cancer_primary | 198 | 0.98 (0.85-1.13) | 0.87 | Ineligible |
| T-DXd | Decreased appetite | breast_cancer_primary | 198 | 0.98 (0.85-1.13) | 0.87 | Ineligible |
| T-DXd | Intentional product use issue | breast_cancer_primary | 198 | 11.70 (9.97-13.72) | 8.03 | Eligible |
| T-DXd | Malignant neoplasm progression | breast_cancer_primary | 182 | 0.68 (0.58-0.78) | 0.61 | Ineligible |
| T-DXd | Malignant neoplasm progression | breast_cancer_primary | 182 | 0.68 (0.58-0.78) | 0.61 | Ineligible |
| T-DXd | Neutropenia | breast_cancer_primary | 167 | 0.52 (0.45-0.61) | 0.48 | Ineligible |
| T-DXd | Neutropenia | breast_cancer_primary | 167 | 0.52 (0.45-0.61) | 0.48 | Ineligible |
| T-DXd | Malaise | breast_cancer_primary | 165 | 0.95 (0.81-1.11) | 0.83 | Ineligible |
| T-DXd | Malaise | breast_cancer_primary | 165 | 0.95 (0.81-1.11) | 0.83 | Ineligible |
| T-DXd | Toxicity to various agents | breast_cancer_primary | 161 | 5.43 (4.60-6.41) | 4.21 | Eligible |
| T-DXd | Weight decreased | breast_cancer_primary | 157 | 1.19 (1.02-1.40) | 1.03 | Eligible |
| T-DXd | Pneumonia | breast_cancer_primary | 154 | 1.42 (1.21-1.67) | 1.21 | Eligible |
| T-DXd | Constipation | breast_cancer_primary | 146 | 1.02 (0.86-1.20) | 0.88 | Ineligible |
| T-DXd | Constipation | breast_cancer_primary | 146 | 1.02 (0.86-1.20) | 0.88 | Ineligible |
| T-DXd | Anaemia | breast_cancer_primary | 135 | 0.75 (0.63-0.89) | 0.66 | Ineligible |
| T-DXd | Anaemia | breast_cancer_primary | 135 | 0.75 (0.63-0.89) | 0.66 | Ineligible |
| T-DXd | Metastases to central nervous system | breast_cancer_primary | 124 | 2.28 (1.90-2.74) | 1.85 | Eligible |
| T-DXd | Cough | breast_cancer_primary | 114 | 0.79 (0.66-0.96) | 0.69 | Ineligible |
| T-DXd | Cough | breast_cancer_primary | 114 | 0.79 (0.66-0.96) | 0.69 | Ineligible |
| T-DXd | General physical health deterioration | breast_cancer_primary | 112 | 1.49 (1.23-1.80) | 1.23 | Eligible |
| T-DXd | Pyrexia | breast_cancer_primary | 103 | 0.55 (0.45-0.66) | 0.48 | Ineligible |
| T-DXd | Pyrexia | breast_cancer_primary | 103 | 0.55 (0.45-0.66) | 0.48 | Ineligible |
| T-DXd | No adverse event | breast_cancer_primary | 102 | 3.31 (2.70-4.06) | 2.59 | Eligible |
| T-DXd | Pleural effusion | breast_cancer_primary | 99 | 1.30 (1.06-1.59) | 1.07 | Eligible |
| T-DXd | Thrombocytopenia | breast_cancer_primary | 98 | 0.89 (0.73-1.09) | 0.75 | Ineligible |
| T-DXd | Thrombocytopenia | breast_cancer_primary | 98 | 0.89 (0.73-1.09) | 0.75 | Ineligible |
| T-DXd | COVID-19 | breast_cancer_primary | 97 | 1.66 (1.35-2.03) | 1.34 | Eligible |
| T-DXd | Pain | breast_cancer_primary | 94 | 0.32 (0.26-0.40) | 0.29 | Ineligible |
| T-DXd | Pain | breast_cancer_primary | 94 | 0.32 (0.26-0.40) | 0.29 | Ineligible |
| T-DXd | Metastases to liver | breast_cancer_primary | 89 | 1.07 (0.87-1.33) | 0.89 | Ineligible |
| T-DXd | Metastases to liver | breast_cancer_primary | 89 | 1.07 (0.87-1.33) | 0.89 | Ineligible |
| T-DXd | Inappropriate schedule of product administration | breast_cancer_primary | 87 | 2.45 (1.97-3.04) | 1.90 | Eligible |
| T-DXd | Neutrophil count decreased | breast_cancer_primary | 87 | 0.86 (0.69-1.06) | 0.72 | Ineligible |
| T-DXd | Neutrophil count decreased | breast_cancer_primary | 87 | 0.86 (0.69-1.06) | 0.72 | Ineligible |
| T-DXd | Drug ineffective | breast_cancer_primary | 85 | 0.59 (0.48-0.74) | 0.51 | Ineligible |
| T-DXd | Drug ineffective | breast_cancer_primary | 85 | 0.59 (0.48-0.74) | 0.51 | Ineligible |
| T-DXd | Headache | breast_cancer_primary | 82 | 0.47 (0.38-0.59) | 0.41 | Ineligible |
| T-DXd | Headache | breast_cancer_primary | 82 | 0.47 (0.38-0.59) | 0.41 | Ineligible |
| T-DXd | Ascites | breast_cancer_primary | 79 | 2.38 (1.89-2.99) | 1.83 | Eligible |
| T-DXd | Dehydration | breast_cancer_primary | 79 | 1.01 (0.81-1.26) | 0.83 | Ineligible |
| T-DXd | Dehydration | breast_cancer_primary | 79 | 1.01 (0.81-1.26) | 0.83 | Ineligible |
| T-DXd | Platelet count decreased | breast_cancer_primary | 79 | 0.75 (0.60-0.93) | 0.62 | Ineligible |
| T-DXd | Platelet count decreased | breast_cancer_primary | 79 | 0.75 (0.60-0.93) | 0.62 | Ineligible |
| T-DXd | Ejection fraction decreased | breast_cancer_primary | 74 | 1.50 (1.19-1.90) | 1.19 | Eligible |
| T-DXd | Pulmonary embolism | breast_cancer_primary | 72 | 1.46 (1.15-1.85) | 1.16 | Eligible |
| T-DXd | Dizziness | breast_cancer_primary | 71 | 0.49 (0.39-0.62) | 0.41 | Ineligible |
| T-DXd | Dizziness | breast_cancer_primary | 71 | 0.49 (0.39-0.62) | 0.41 | Ineligible |
| T-DXd | Infection | breast_cancer_primary | 71 | 1.47 (1.16-1.87) | 1.16 | Eligible |
| T-DXd | Pneumocystis jirovecii pneumonia | breast_cancer_primary | 71 | 6.39 (4.97-8.21) | 4.55 | Eligible |
| T-DXd | Neuropathy peripheral | breast_cancer_primary | 70 | 0.56 (0.44-0.71) | 0.47 | Ineligible |
| T-DXd | Neuropathy peripheral | breast_cancer_primary | 70 | 0.56 (0.44-0.71) | 0.47 | Ineligible |
| T-DXd | Lung disorder | breast_cancer_primary | 68 | 2.83 (2.21-3.63) | 2.12 | Eligible |
| T-DXd | Respiratory failure | breast_cancer_primary | 63 | 2.23 (1.72-2.87) | 1.67 | Eligible |
| T-DXd | Sepsis | breast_cancer_primary | 62 | 1.51 (1.17-1.95) | 1.17 | Eligible |
| T-DXd | White blood cell count decreased | breast_cancer_primary | 62 | 0.20 (0.16-0.26) | 0.17 | Ineligible |
| T-DXd | White blood cell count decreased | breast_cancer_primary | 62 | 0.20 (0.16-0.26) | 0.17 | Ineligible |
| T-DXd | Fall | breast_cancer_primary | 60 | 0.84 (0.65-1.08) | 0.68 | Ineligible |
| T-DXd | Fall | breast_cancer_primary | 60 | 0.84 (0.65-1.08) | 0.68 | Ineligible |
| T-DXd | Product dose omission issue | breast_cancer_primary | 60 | 0.98 (0.76-1.26) | 0.78 | Ineligible |
| T-DXd | Product dose omission issue | breast_cancer_primary | 60 | 0.98 (0.76-1.26) | 0.78 | Ineligible |
| T-DXd | Underdose | breast_cancer_primary | 60 | 7.04 (5.35-9.27) | 4.85 | Eligible |
| T-DXd | Abdominal pain | breast_cancer_primary | 59 | 0.65 (0.50-0.84) | 0.53 | Ineligible |
| T-DXd | Abdominal pain | breast_cancer_primary | 59 | 0.65 (0.50-0.84) | 0.53 | Ineligible |
| T-DXd | Febrile neutropenia | breast_cancer_primary | 59 | 0.59 (0.45-0.76) | 0.48 | Ineligible |
| T-DXd | Febrile neutropenia | breast_cancer_primary | 59 | 0.59 (0.45-0.76) | 0.48 | Ineligible |
| T-DXd | Gastrointestinal disorder | breast_cancer_primary | 57 | 1.64 (1.25-2.13) | 1.24 | Eligible |
| T-DXd | Haemoglobin decreased | breast_cancer_primary | 57 | 0.71 (0.55-0.92) | 0.58 | Ineligible |
| T-DXd | Haemoglobin decreased | breast_cancer_primary | 57 | 0.71 (0.55-0.92) | 0.58 | Ineligible |
| T-DXd | Metastases to bone | breast_cancer_primary | 54 | 0.49 (0.38-0.65) | 0.41 | Ineligible |
| T-DXd | Metastases to bone | breast_cancer_primary | 54 | 0.49 (0.38-0.65) | 0.41 | Ineligible |
| T-DXd | Pancytopenia | breast_cancer_primary | 54 | 1.32 (1.01-1.73) | 1.02 | Eligible |
| T-DXd | Incorrect dose administered | breast_cancer_primary | 52 | 3.78 (2.84-5.02) | 2.71 | Eligible |
| T-DXd | Pulmonary toxicity | breast_cancer_primary | 52 | 7.30 (5.43-9.82) | 4.91 | Eligible |
| T-DXd | Hepatic failure | breast_cancer_primary | 51 | 2.06 (1.55-2.73) | 1.50 | Eligible |
| T-DXd | Acute kidney injury | breast_cancer_primary | 50 | 1.03 (0.78-1.36) | 0.80 | Ineligible |
| T-DXd | Acute kidney injury | breast_cancer_primary | 50 | 1.03 (0.78-1.36) | 0.80 | Ineligible |
| T-DXd | Adverse drug reaction | breast_cancer_primary | 48 | 3.81 (2.83-5.12) | 2.70 | Eligible |
| T-DXd | Drug intolerance | breast_cancer_primary | 47 | 1.15 (0.86-1.53) | 0.88 | Ineligible |
| T-DXd | Drug intolerance | breast_cancer_primary | 47 | 1.15 (0.86-1.53) | 0.88 | Ineligible |
| T-DXd | Hypotension | breast_cancer_primary | 47 | 0.93 (0.69-1.24) | 0.72 | Ineligible |
| T-DXd | Hypotension | breast_cancer_primary | 47 | 0.93 (0.69-1.24) | 0.72 | Ineligible |
| T-DXd | Hepatic cytolysis | breast_cancer_primary | 46 | 2.31 (1.71-3.11) | 1.64 | Eligible |
| T-DXd | Feeling abnormal | breast_cancer_primary | 43 | 0.73 (0.54-0.99) | 0.57 | Ineligible |
| T-DXd | Feeling abnormal | breast_cancer_primary | 43 | 0.73 (0.54-0.99) | 0.57 | Ineligible |
| T-DXd | Breast cancer | breast_cancer_primary | 42 | 0.40 (0.29-0.54) | 0.32 | Ineligible |
| T-DXd | Breast cancer | breast_cancer_primary | 42 | 0.40 (0.29-0.54) | 0.32 | Ineligible |
| T-DXd | Back pain | breast_cancer_primary | 41 | 0.34 (0.25-0.46) | 0.28 | Ineligible |
| T-DXd | Back pain | breast_cancer_primary | 41 | 0.34 (0.25-0.46) | 0.28 | Ineligible |
| T-DXd | Pain in extremity | breast_cancer_primary | 41 | 0.33 (0.24-0.45) | 0.27 | Ineligible |
| T-DXd | Pain in extremity | breast_cancer_primary | 41 | 0.33 (0.24-0.45) | 0.27 | Ineligible |
| T-DXd | Seizure | breast_cancer_primary | 41 | 1.56 (1.14-2.14) | 1.13 | Eligible |
| T-DXd | Hypokalaemia | breast_cancer_primary | 40 | 1.04 (0.76-1.42) | 0.78 | Ineligible |
| T-DXd | Hypokalaemia | breast_cancer_primary | 40 | 1.04 (0.76-1.42) | 0.78 | Ineligible |
| T-DXd | Vision blurred | breast_cancer_primary | 40 | 1.24 (0.91-1.71) | 0.92 | Ineligible |
| T-DXd | Vision blurred | breast_cancer_primary | 40 | 1.24 (0.91-1.71) | 0.92 | Ineligible |
| T-DXd | Cardiac failure | breast_cancer_primary | 39 | 0.78 (0.56-1.07) | 0.60 | Ineligible |
| T-DXd | Cardiac failure | breast_cancer_primary | 39 | 0.78 (0.56-1.07) | 0.60 | Ineligible |
| T-DXd | Condition aggravated | breast_cancer_primary | 39 | 0.73 (0.53-1.00) | 0.57 | Ineligible |
| T-DXd | Condition aggravated | breast_cancer_primary | 39 | 0.73 (0.53-1.00) | 0.57 | Ineligible |
| T-DXd | Hepatotoxicity | breast_cancer_primary | 38 | 1.32 (0.96-1.83) | 0.96 | Ineligible |
| T-DXd | Hepatotoxicity | breast_cancer_primary | 38 | 1.32 (0.96-1.83) | 0.96 | Ineligible |
| T-DXd | Illness | breast_cancer_primary | 38 | 0.90 (0.65-1.25) | 0.68 | Ineligible |
| T-DXd | Illness | breast_cancer_primary | 38 | 0.90 (0.65-1.25) | 0.68 | Ineligible |
| T-DXd | Rash | breast_cancer_primary | 38 | 0.21 (0.15-0.28) | 0.17 | Ineligible |
| T-DXd | Rash | breast_cancer_primary | 38 | 0.21 (0.15-0.28) | 0.17 | Ineligible |
| T-DXd | Therapy change | breast_cancer_primary | 38 | 5.47 (3.90-7.69) | 3.67 | Eligible |
| T-DXd | Hepatic function abnormal | breast_cancer_primary | 37 | 0.89 (0.65-1.24) | 0.68 | Ineligible |
| T-DXd | Hepatic function abnormal | breast_cancer_primary | 37 | 0.89 (0.65-1.24) | 0.68 | Ineligible |
| T-DXd | Urinary tract infection | breast_cancer_primary | 37 | 0.50 (0.36-0.69) | 0.40 | Ineligible |
| T-DXd | Urinary tract infection | breast_cancer_primary | 37 | 0.50 (0.36-0.69) | 0.40 | Ineligible |
| T-DXd | Septic shock | breast_cancer_primary | 36 | 2.31 (1.65-3.23) | 1.57 | Eligible |
| T-DXd | Hepatic enzyme increased | breast_cancer_primary | 35 | 0.98 (0.70-1.37) | 0.73 | Ineligible |
| T-DXd | Hepatic enzyme increased | breast_cancer_primary | 35 | 0.98 (0.70-1.37) | 0.73 | Ineligible |
| T-DXd | Oedema peripheral | breast_cancer_primary | 35 | 0.72 (0.51-1.00) | 0.55 | Ineligible |
| T-DXd | Oedema peripheral | breast_cancer_primary | 35 | 0.72 (0.51-1.00) | 0.55 | Ineligible |
| T-DXd | Renal failure | breast_cancer_primary | 35 | 1.02 (0.73-1.43) | 0.76 | Ineligible |
| T-DXd | Renal failure | breast_cancer_primary | 35 | 1.02 (0.73-1.43) | 0.76 | Ineligible |
| T-DXd | Abdominal pain upper | breast_cancer_primary | 34 | 0.43 (0.31-0.61) | 0.34 | Ineligible |
| T-DXd | Abdominal pain upper | breast_cancer_primary | 34 | 0.43 (0.31-0.61) | 0.34 | Ineligible |
| T-DXd | Adverse event | breast_cancer_primary | 33 | 3.11 (2.18-4.43) | 2.04 | Eligible |
| T-DXd | Blood bilirubin increased | breast_cancer_primary | 33 | 1.44 (1.02-2.04) | 1.01 | Eligible |
| T-DXd | Hyponatraemia | breast_cancer_primary | 32 | 1.46 (1.03-2.08) | 1.02 | Eligible |
| T-DXd | Liver disorder | breast_cancer_primary | 32 | 1.24 (0.87-1.76) | 0.88 | Ineligible |
| T-DXd | Liver disorder | breast_cancer_primary | 32 | 1.24 (0.87-1.76) | 0.88 | Ineligible |
| T-DXd | Dyspepsia | breast_cancer_primary | 31 | 0.63 (0.44-0.91) | 0.48 | Ineligible |
| T-DXd | Dyspepsia | breast_cancer_primary | 31 | 0.63 (0.44-0.91) | 0.48 | Ineligible |
| T-DXd | Peripheral swelling | breast_cancer_primary | 31 | 0.46 (0.32-0.66) | 0.36 | Ineligible |
| T-DXd | Peripheral swelling | breast_cancer_primary | 31 | 0.46 (0.32-0.66) | 0.36 | Ineligible |
| T-DXd | Cholestasis | breast_cancer_primary | 30 | 2.44 (1.69-3.53) | 1.58 | Eligible |
| T-DXd | Metastases to lung | breast_cancer_primary | 30 | 0.57 (0.40-0.82) | 0.43 | Ineligible |
| T-DXd | Metastases to lung | breast_cancer_primary | 30 | 0.57 (0.40-0.82) | 0.43 | Ineligible |
| T-DXd | Weight increased | breast_cancer_primary | 30 | 0.55 (0.38-0.79) | 0.42 | Ineligible |
| T-DXd | Weight increased | breast_cancer_primary | 30 | 0.55 (0.38-0.79) | 0.42 | Ineligible |
| T-DXd | Abdominal discomfort | breast_cancer_primary | 29 | 0.47 (0.32-0.67) | 0.36 | Ineligible |
| T-DXd | Abdominal discomfort | breast_cancer_primary | 29 | 0.47 (0.32-0.67) | 0.36 | Ineligible |
| T-DXd | Abdominal distension | breast_cancer_primary | 29 | 0.83 (0.58-1.21) | 0.61 | Ineligible |
| T-DXd | Abdominal distension | breast_cancer_primary | 29 | 0.83 (0.58-1.21) | 0.61 | Ineligible |
| T-DXd | Chills | breast_cancer_primary | 29 | 0.49 (0.34-0.70) | 0.37 | Ineligible |
| T-DXd | Chills | breast_cancer_primary | 29 | 0.49 (0.34-0.70) | 0.37 | Ineligible |
| T-DXd | Hospitalisation | breast_cancer_primary | 29 | 1.24 (0.86-1.80) | 0.87 | Ineligible |
| T-DXd | Hospitalisation | breast_cancer_primary | 29 | 1.24 (0.86-1.80) | 0.87 | Ineligible |
| T-DXd | Lung opacity | breast_cancer_primary | 29 | 9.25 (6.18-13.85) | 5.40 | Eligible |
| T-DXd | Muscle spasms | breast_cancer_primary | 29 | 0.55 (0.38-0.79) | 0.41 | Ineligible |
| T-DXd | Muscle spasms | breast_cancer_primary | 29 | 0.55 (0.38-0.79) | 0.41 | Ineligible |
| T-DXd | Pruritus | breast_cancer_primary | 29 | 0.24 (0.17-0.35) | 0.19 | Ineligible |
| T-DXd | Pruritus | breast_cancer_primary | 29 | 0.24 (0.17-0.35) | 0.19 | Ineligible |
| T-DXd | Somnolence | breast_cancer_primary | 29 | 0.72 (0.50-1.05) | 0.54 | Ineligible |
| T-DXd | Somnolence | breast_cancer_primary | 29 | 0.72 (0.50-1.05) | 0.54 | Ineligible |
| T-DXd | Stomatitis | breast_cancer_primary | 29 | 0.24 (0.16-0.34) | 0.19 | Ineligible |
| T-DXd | Stomatitis | breast_cancer_primary | 29 | 0.24 (0.16-0.34) | 0.19 | Ineligible |
| T-DXd | Thrombosis | breast_cancer_primary | 29 | 0.83 (0.57-1.20) | 0.61 | Ineligible |
| T-DXd | Thrombosis | breast_cancer_primary | 29 | 0.83 (0.57-1.20) | 0.61 | Ineligible |
| T-DXd | Anxiety | breast_cancer_primary | 28 | 0.17 (0.12-0.25) | 0.14 | Ineligible |
| T-DXd | Anxiety | breast_cancer_primary | 28 | 0.17 (0.12-0.25) | 0.14 | Ineligible |
| T-DXd | Colitis | breast_cancer_primary | 28 | 1.07 (0.73-1.55) | 0.76 | Ineligible |
| T-DXd | Colitis | breast_cancer_primary | 28 | 1.07 (0.73-1.55) | 0.76 | Ineligible |
| T-DXd | Oxygen saturation decreased | breast_cancer_primary | 28 | 1.34 (0.92-1.95) | 0.92 | Ineligible |
| T-DXd | Oxygen saturation decreased | breast_cancer_primary | 28 | 1.34 (0.92-1.95) | 0.92 | Ineligible |
| T-DXd | Paraesthesia | breast_cancer_primary | 28 | 0.42 (0.29-0.62) | 0.33 | Ineligible |
| T-DXd | Paraesthesia | breast_cancer_primary | 28 | 0.42 (0.29-0.62) | 0.33 | Ineligible |
| T-DXd | Product use issue | breast_cancer_primary | 28 | 0.60 (0.41-0.87) | 0.45 | Ineligible |
| T-DXd | Product use issue | breast_cancer_primary | 28 | 0.60 (0.41-0.87) | 0.45 | Ineligible |
| T-DXd | Unevaluable event | breast_cancer_primary | 28 | 2.99 (2.04-4.39) | 1.88 | Eligible |
| T-DXd | Arthralgia | breast_cancer_primary | 27 | 0.14 (0.10-0.21) | 0.12 | Ineligible |
| T-DXd | Arthralgia | breast_cancer_primary | 27 | 0.14 (0.10-0.21) | 0.12 | Ineligible |
| T-DXd | Gastrointestinal toxicity | breast_cancer_primary | 27 | 4.05 (2.73-6.02) | 2.55 | Eligible |
| T-DXd | Metastases to meninges | breast_cancer_primary | 27 | 3.60 (2.43-5.35) | 2.26 | Eligible |
| T-DXd | Myalgia | breast_cancer_primary | 27 | 0.37 (0.25-0.54) | 0.29 | Ineligible |
| T-DXd | Myalgia | breast_cancer_primary | 27 | 0.37 (0.25-0.54) | 0.29 | Ineligible |
| T-DXd | Pulmonary fibrosis | breast_cancer_primary | 27 | 3.31 (2.24-4.91) | 2.07 | Eligible |
| T-DXd | Pulmonary oedema | breast_cancer_primary | 27 | 0.92 (0.63-1.35) | 0.66 | Ineligible |
| T-DXd | Pulmonary oedema | breast_cancer_primary | 27 | 0.92 (0.63-1.35) | 0.66 | Ineligible |
| T-DXd | Visual impairment | breast_cancer_primary | 26 | 0.71 (0.48-1.05) | 0.52 | Ineligible |
| T-DXd | Visual impairment | breast_cancer_primary | 26 | 0.71 (0.48-1.05) | 0.52 | Ineligible |
| T-DXd | Alanine aminotransferase increased | breast_cancer_primary | 25 | 0.46 (0.31-0.68) | 0.34 | Ineligible |
| T-DXd | Alanine aminotransferase increased | breast_cancer_primary | 25 | 0.46 (0.31-0.68) | 0.34 | Ineligible |
| T-DXd | Haematotoxicity | breast_cancer_primary | 25 | 1.53 (1.03-2.29) | 1.01 | Eligible |
| T-DXd | Hepatic cirrhosis | breast_cancer_primary | 25 | 2.62 (1.75-3.93) | 1.61 | Eligible |
| T-DXd | Loss of consciousness | breast_cancer_primary | 24 | 0.98 (0.66-1.47) | 0.68 | Ineligible |
| T-DXd | Loss of consciousness | breast_cancer_primary | 24 | 0.98 (0.66-1.47) | 0.68 | Ineligible |
| T-DXd | Cell death | breast_cancer_primary | 23 | 7.39 (4.74-11.53) | 4.31 | Eligible |
| T-DXd | Chest pain | breast_cancer_primary | 23 | 0.40 (0.27-0.61) | 0.30 | Ineligible |
| T-DXd | Chest pain | breast_cancer_primary | 23 | 0.40 (0.27-0.61) | 0.30 | Ineligible |
| T-DXd | Mucosal inflammation | breast_cancer_primary | 23 | 0.41 (0.27-0.63) | 0.31 | Ineligible |
| T-DXd | Mucosal inflammation | breast_cancer_primary | 23 | 0.41 (0.27-0.63) | 0.31 | Ineligible |
| T-DXd | Quality of life decreased | breast_cancer_primary | 23 | 6.02 (3.89-9.34) | 3.63 | Eligible |
| T-DXd | Aspartate aminotransferase increased | breast_cancer_primary | 22 | 0.43 (0.28-0.65) | 0.32 | Ineligible |
| T-DXd | Aspartate aminotransferase increased | breast_cancer_primary | 22 | 0.43 (0.28-0.65) | 0.32 | Ineligible |
| T-DXd | Erythema | breast_cancer_primary | 22 | 0.27 (0.17-0.41) | 0.20 | Ineligible |
| T-DXd | Erythema | breast_cancer_primary | 22 | 0.27 (0.17-0.41) | 0.20 | Ineligible |
| T-DXd | Renal impairment | breast_cancer_primary | 22 | 0.61 (0.40-0.94) | 0.44 | Ineligible |
| T-DXd | Renal impairment | breast_cancer_primary | 22 | 0.61 (0.40-0.94) | 0.44 | Ineligible |
| T-DXd | Taste disorder | breast_cancer_primary | 22 | 1.02 (0.67-1.56) | 0.69 | Ineligible |
| T-DXd | Taste disorder | breast_cancer_primary | 22 | 1.02 (0.67-1.56) | 0.69 | Ineligible |
| T-DXd | Cataract | breast_cancer_primary | 21 | 0.97 (0.63-1.50) | 0.66 | Ineligible |
| T-DXd | Cataract | breast_cancer_primary | 21 | 0.97 (0.63-1.50) | 0.66 | Ineligible |
| T-DXd | Dry eye | breast_cancer_primary | 21 | 0.94 (0.61-1.45) | 0.64 | Ineligible |
| T-DXd | Dry eye | breast_cancer_primary | 21 | 0.94 (0.61-1.45) | 0.64 | Ineligible |
| T-DXd | Gastrooesophageal reflux disease | breast_cancer_primary | 21 | 0.76 (0.49-1.17) | 0.53 | Ineligible |
| T-DXd | Gastrooesophageal reflux disease | breast_cancer_primary | 21 | 0.76 (0.49-1.17) | 0.53 | Ineligible |
| T-DXd | Lung infiltration | breast_cancer_primary | 21 | 2.93 (1.88-4.57) | 1.70 | Eligible |
| T-DXd | Mobility decreased | breast_cancer_primary | 21 | 1.28 (0.83-1.98) | 0.84 | Ineligible |
| T-DXd | Mobility decreased | breast_cancer_primary | 21 | 1.28 (0.83-1.98) | 0.84 | Ineligible |
| T-DXd | Pneumonia bacterial | breast_cancer_primary | 21 | 8.38 (5.24-13.41) | 4.67 | Eligible |
| T-DXd | Brain fog | breast_cancer_primary | 20 | 2.87 (1.82-4.52) | 1.64 | Eligible |
| T-DXd | Chest discomfort | breast_cancer_primary | 20 | 0.45 (0.29-0.71) | 0.33 | Ineligible |
| T-DXd | Chest discomfort | breast_cancer_primary | 20 | 0.45 (0.29-0.71) | 0.33 | Ineligible |
| T-DXd | Feeding disorder | breast_cancer_primary | 20 | 1.10 (0.71-1.72) | 0.73 | Ineligible |
| T-DXd | Feeding disorder | breast_cancer_primary | 20 | 1.10 (0.71-1.72) | 0.73 | Ineligible |
| T-DXd | Insomnia | breast_cancer_primary | 20 | 0.24 (0.16-0.37) | 0.18 | Ineligible |
| T-DXd | Insomnia | breast_cancer_primary | 20 | 0.24 (0.16-0.37) | 0.18 | Ineligible |
| T-DXd | Leukopenia | breast_cancer_primary | 20 | 0.19 (0.12-0.30) | 0.15 | Ineligible |
| T-DXd | Leukopenia | breast_cancer_primary | 20 | 0.19 (0.12-0.30) | 0.15 | Ineligible |
| T-DXd | Lymphoedema | breast_cancer_primary | 20 | 0.83 (0.53-1.29) | 0.57 | Ineligible |
| T-DXd | Lymphoedema | breast_cancer_primary | 20 | 0.83 (0.53-1.29) | 0.57 | Ineligible |
| T-DXd | Muscular weakness | breast_cancer_primary | 20 | 0.64 (0.41-0.99) | 0.45 | Ineligible |
| T-DXd | Muscular weakness | breast_cancer_primary | 20 | 0.64 (0.41-0.99) | 0.45 | Ineligible |
| T-DXd | Product administration error | breast_cancer_primary | 20 | 9.14 (5.62-14.84) | 4.93 | Eligible |
| T-DXd | Respiratory disorder | breast_cancer_primary | 20 | 2.55 (1.62-4.01) | 1.47 | Eligible |
| T-DXd | Tumour marker increased | breast_cancer_primary | 20 | 0.57 (0.37-0.89) | 0.41 | Ineligible |
| T-DXd | Tumour marker increased | breast_cancer_primary | 20 | 0.57 (0.37-0.89) | 0.41 | Ineligible |
| T-DXd | Acute respiratory distress syndrome | breast_cancer_primary | 19 | 2.47 (1.55-3.93) | 1.41 | Eligible |
| T-DXd | Confusional state | breast_cancer_primary | 19 | 0.55 (0.35-0.86) | 0.39 | Ineligible |
| T-DXd | Confusional state | breast_cancer_primary | 19 | 0.55 (0.35-0.86) | 0.39 | Ineligible |
| T-DXd | Hypoaesthesia | breast_cancer_primary | 19 | 0.29 (0.19-0.46) | 0.22 | Ineligible |
| T-DXd | Hypoaesthesia | breast_cancer_primary | 19 | 0.29 (0.19-0.46) | 0.22 | Ineligible |
| T-DXd | Nasopharyngitis | breast_cancer_primary | 19 | 0.32 (0.20-0.51) | 0.24 | Ineligible |
| T-DXd | Nasopharyngitis | breast_cancer_primary | 19 | 0.32 (0.20-0.51) | 0.24 | Ineligible |
| T-DXd | Acute respiratory failure | breast_cancer_primary | 18 | 2.67 (1.66-4.30) | 1.48 | Eligible |
| T-DXd | Brain oedema | breast_cancer_primary | 18 | 2.58 (1.60-4.15) | 1.43 | Eligible |
| T-DXd | Drug-induced liver injury | breast_cancer_primary | 18 | 0.85 (0.53-1.35) | 0.57 | Ineligible |
| T-DXd | Drug-induced liver injury | breast_cancer_primary | 18 | 0.85 (0.53-1.35) | 0.57 | Ineligible |
| T-DXd | Dry skin | breast_cancer_primary | 18 | 0.26 (0.16-0.41) | 0.19 | Ineligible |
| T-DXd | Dry skin | breast_cancer_primary | 18 | 0.26 (0.16-0.41) | 0.19 | Ineligible |
| T-DXd | Infusion related reaction | breast_cancer_primary | 18 | 0.58 (0.37-0.93) | 0.41 | Ineligible |
| T-DXd | Infusion related reaction | breast_cancer_primary | 18 | 0.58 (0.37-0.93) | 0.41 | Ineligible |
| T-DXd | Oropharyngeal pain | breast_cancer_primary | 18 | 0.49 (0.31-0.78) | 0.35 | Ineligible |
| T-DXd | Oropharyngeal pain | breast_cancer_primary | 18 | 0.49 (0.31-0.78) | 0.35 | Ineligible |
| T-DXd | Pneumothorax | breast_cancer_primary | 18 | 1.93 (1.20-3.10) | 1.13 | Eligible |
| T-DXd | Syncope | breast_cancer_primary | 18 | 0.63 (0.40-1.01) | 0.44 | Ineligible |
| T-DXd | Syncope | breast_cancer_primary | 18 | 0.63 (0.40-1.01) | 0.44 | Ineligible |
| T-DXd | Therapeutic response unexpected | breast_cancer_primary | 18 | 2.97 (1.84-4.79) | 1.63 | Eligible |
| T-DXd | Transaminases increased | breast_cancer_primary | 18 | 0.71 (0.44-1.13) | 0.49 | Ineligible |
| T-DXd | Transaminases increased | breast_cancer_primary | 18 | 0.71 (0.44-1.13) | 0.49 | Ineligible |
| T-DXd | Blood potassium decreased | breast_cancer_primary | 17 | 0.95 (0.59-1.54) | 0.62 | Ineligible |
| T-DXd | Blood potassium decreased | breast_cancer_primary | 17 | 0.95 (0.59-1.54) | 0.62 | Ineligible |
| T-DXd | Cardiac disorder | breast_cancer_primary | 17 | 0.74 (0.46-1.20) | 0.50 | Ineligible |
| T-DXd | Cardiac disorder | breast_cancer_primary | 17 | 0.74 (0.46-1.20) | 0.50 | Ineligible |
| T-DXd | Cardiotoxicity | breast_cancer_primary | 17 | 0.54 (0.33-0.87) | 0.38 | Ineligible |
| T-DXd | Cardiotoxicity | breast_cancer_primary | 17 | 0.54 (0.33-0.87) | 0.38 | Ineligible |
| T-DXd | Dysgeusia | breast_cancer_primary | 17 | 0.43 (0.27-0.70) | 0.31 | Ineligible |
| T-DXd | Dysgeusia | breast_cancer_primary | 17 | 0.43 (0.27-0.70) | 0.31 | Ineligible |
| T-DXd | Epistaxis | breast_cancer_primary | 17 | 0.26 (0.16-0.41) | 0.19 | Ineligible |
| T-DXd | Epistaxis | breast_cancer_primary | 17 | 0.26 (0.16-0.41) | 0.19 | Ineligible |
| T-DXd | Gait disturbance | breast_cancer_primary | 17 | 0.27 (0.17-0.44) | 0.20 | Ineligible |
| T-DXd | Gait disturbance | breast_cancer_primary | 17 | 0.27 (0.17-0.44) | 0.20 | Ineligible |
| T-DXd | Hypersomnia | breast_cancer_primary | 17 | 2.00 (1.23-3.26) | 1.14 | Eligible |
| T-DXd | Metastases to lymph nodes | breast_cancer_primary | 17 | 0.60 (0.37-0.97) | 0.42 | Ineligible |
| T-DXd | Metastases to lymph nodes | breast_cancer_primary | 17 | 0.60 (0.37-0.97) | 0.42 | Ineligible |
| T-DXd | Radiation necrosis | breast_cancer_primary | 17 | 7.28 (4.34-12.20) | 3.95 | Eligible |
| T-DXd | Blood pressure decreased | breast_cancer_primary | 16 | 0.97 (0.59-1.59) | 0.62 | Ineligible |
| T-DXd | Blood pressure decreased | breast_cancer_primary | 16 | 0.97 (0.59-1.59) | 0.62 | Ineligible |
| T-DXd | Blood pressure increased | breast_cancer_primary | 16 | 0.46 (0.28-0.76) | 0.33 | Ineligible |
| T-DXd | Blood pressure increased | breast_cancer_primary | 16 | 0.46 (0.28-0.76) | 0.33 | Ineligible |
| T-DXd | Cardiac dysfunction | breast_cancer_primary | 16 | 1.55 (0.94-2.55) | 0.91 | Ineligible |
| T-DXd | Cardiac dysfunction | breast_cancer_primary | 16 | 1.55 (0.94-2.55) | 0.91 | Ineligible |
| T-DXd | Cellulitis | breast_cancer_primary | 16 | 0.61 (0.37-1.00) | 0.42 | Ineligible |
| T-DXd | Cellulitis | breast_cancer_primary | 16 | 0.61 (0.37-1.00) | 0.42 | Ineligible |
| T-DXd | Cytopenia | breast_cancer_primary | 16 | 1.18 (0.72-1.95) | 0.74 | Ineligible |
| T-DXd | Cytopenia | breast_cancer_primary | 16 | 1.18 (0.72-1.95) | 0.74 | Ineligible |
| T-DXd | Dysphagia | breast_cancer_primary | 16 | 0.37 (0.23-0.61) | 0.27 | Ineligible |
| T-DXd | Dysphagia | breast_cancer_primary | 16 | 0.37 (0.23-0.61) | 0.27 | Ineligible |
| T-DXd | Haemorrhage | breast_cancer_primary | 16 | 0.72 (0.44-1.18) | 0.48 | Ineligible |
| T-DXd | Haemorrhage | breast_cancer_primary | 16 | 0.72 (0.44-1.18) | 0.48 | Ineligible |
| T-DXd | Influenza | breast_cancer_primary | 16 | 0.53 (0.32-0.86) | 0.37 | Ineligible |
| T-DXd | Influenza | breast_cancer_primary | 16 | 0.53 (0.32-0.86) | 0.37 | Ineligible |
| T-DXd | Liver function test increased | breast_cancer_primary | 16 | 0.77 (0.47-1.27) | 0.51 | Ineligible |
| T-DXd | Liver function test increased | breast_cancer_primary | 16 | 0.77 (0.47-1.27) | 0.51 | Ineligible |
| T-DXd | Bedridden | breast_cancer_primary | 15 | 6.70 (3.88-11.57) | 3.54 | Eligible |
| T-DXd | Clostridium difficile infection | breast_cancer_primary | 15 | 2.20 (1.31-3.70) | 1.18 | Eligible |
| T-DXd | Flatulence | breast_cancer_primary | 15 | 0.95 (0.57-1.58) | 0.60 | Ineligible |
| T-DXd | Flatulence | breast_cancer_primary | 15 | 0.95 (0.57-1.58) | 0.60 | Ineligible |
| T-DXd | Hypoxia | breast_cancer_primary | 15 | 0.93 (0.56-1.55) | 0.59 | Ineligible |
| T-DXd | Hypoxia | breast_cancer_primary | 15 | 0.93 (0.56-1.55) | 0.59 | Ineligible |
| T-DXd | Prescribed underdose | breast_cancer_primary | 15 | 7.52 (4.33-13.04) | 3.90 | Eligible |
| T-DXd | Weight abnormal | breast_cancer_primary | 15 | 13.40 (7.48-24.01) | 5.92 | Eligible |
| T-DXd | Blood alkaline phosphatase increased | breast_cancer_primary | 14 | 0.64 (0.38-1.09) | 0.43 | Ineligible |
| T-DXd | Blood alkaline phosphatase increased | breast_cancer_primary | 14 | 0.64 (0.38-1.09) | 0.43 | Ineligible |
| T-DXd | Blood glucose increased | breast_cancer_primary | 14 | 0.35 (0.20-0.59) | 0.25 | Ineligible |
| T-DXd | Blood glucose increased | breast_cancer_primary | 14 | 0.35 (0.20-0.59) | 0.25 | Ineligible |
| T-DXd | Bone pain | breast_cancer_primary | 14 | 0.15 (0.09-0.25) | 0.11 | Ineligible |
| T-DXd | Bone pain | breast_cancer_primary | 14 | 0.15 (0.09-0.25) | 0.11 | Ineligible |
| T-DXd | Chromaturia | breast_cancer_primary | 14 | 2.13 (1.24-3.64) | 1.12 | Eligible |
| T-DXd | Dysarthria | breast_cancer_primary | 14 | 2.52 (1.47-4.33) | 1.29 | Eligible |
| T-DXd | Femur fracture | breast_cancer_primary | 14 | 1.22 (0.71-2.07) | 0.73 | Ineligible |
| T-DXd | Femur fracture | breast_cancer_primary | 14 | 1.22 (0.71-2.07) | 0.73 | Ineligible |
| T-DXd | Lymphangiosis carcinomatosa | breast_cancer_primary | 14 | 3.09 (1.79-5.32) | 1.53 | Eligible |
| T-DXd | Neoplasm | breast_cancer_primary | 14 | 1.08 (0.64-1.84) | 0.66 | Ineligible |
| T-DXd | Neoplasm | breast_cancer_primary | 14 | 1.08 (0.64-1.84) | 0.66 | Ineligible |
| T-DXd | Sleep disorder | breast_cancer_primary | 14 | 0.60 (0.36-1.02) | 0.40 | Ineligible |
| T-DXd | Sleep disorder | breast_cancer_primary | 14 | 0.60 (0.36-1.02) | 0.40 | Ineligible |
| T-DXd | Bacterial infection | breast_cancer_primary | 13 | 3.05 (1.74-5.36) | 1.47 | Eligible |
| T-DXd | Cardiac arrest | breast_cancer_primary | 13 | 0.90 (0.52-1.56) | 0.56 | Ineligible |
| T-DXd | Cardiac arrest | breast_cancer_primary | 13 | 0.90 (0.52-1.56) | 0.56 | Ineligible |
| T-DXd | Clostridium difficile colitis | breast_cancer_primary | 13 | 3.49 (1.98-6.15) | 1.67 | Eligible |
| T-DXd | Depression | breast_cancer_primary | 13 | 0.19 (0.11-0.32) | 0.14 | Ineligible |
| T-DXd | Depression | breast_cancer_primary | 13 | 0.19 (0.11-0.32) | 0.14 | Ineligible |
| T-DXd | Dry mouth | breast_cancer_primary | 13 | 0.35 (0.20-0.60) | 0.24 | Ineligible |
| T-DXd | Dry mouth | breast_cancer_primary | 13 | 0.35 (0.20-0.60) | 0.24 | Ineligible |
| T-DXd | Flushing | breast_cancer_primary | 13 | 0.44 (0.25-0.76) | 0.30 | Ineligible |
| T-DXd | Flushing | breast_cancer_primary | 13 | 0.44 (0.25-0.76) | 0.30 | Ineligible |
| T-DXd | Gamma-glutamyltransferase increased | breast_cancer_primary | 13 | 0.55 (0.32-0.95) | 0.37 | Ineligible |
| T-DXd | Gamma-glutamyltransferase increased | breast_cancer_primary | 13 | 0.55 (0.32-0.95) | 0.37 | Ineligible |
| T-DXd | Haematological infection | breast_cancer_primary | 13 | 10.90 (5.91-20.10) | 4.93 | Eligible |
| T-DXd | Heart rate increased | breast_cancer_primary | 13 | 0.52 (0.30-0.91) | 0.35 | Ineligible |
| T-DXd | Heart rate increased | breast_cancer_primary | 13 | 0.52 (0.30-0.91) | 0.35 | Ineligible |
| T-DXd | Impaired quality of life | breast_cancer_primary | 13 | 0.18 (0.10-0.31) | 0.13 | Ineligible |
| T-DXd | Impaired quality of life | breast_cancer_primary | 13 | 0.18 (0.10-0.31) | 0.13 | Ineligible |
| T-DXd | Intestinal obstruction | breast_cancer_primary | 13 | 1.52 (0.88-2.65) | 0.85 | Ineligible |
| T-DXd | Intestinal obstruction | breast_cancer_primary | 13 | 1.52 (0.88-2.65) | 0.85 | Ineligible |
| T-DXd | Neoplasm progression | breast_cancer_primary | 13 | 0.06 (0.04-0.11) | 0.05 | Ineligible |
| T-DXd | Neoplasm progression | breast_cancer_primary | 13 | 0.06 (0.04-0.11) | 0.05 | Ineligible |
| T-DXd | Palpitations | breast_cancer_primary | 13 | 0.42 (0.24-0.72) | 0.29 | Ineligible |
| T-DXd | Palpitations | breast_cancer_primary | 13 | 0.42 (0.24-0.72) | 0.29 | Ineligible |
| T-DXd | Pericardial effusion | breast_cancer_primary | 13 | 0.71 (0.41-1.22) | 0.46 | Ineligible |
| T-DXd | Pericardial effusion | breast_cancer_primary | 13 | 0.71 (0.41-1.22) | 0.46 | Ineligible |
| T-DXd | Tachycardia | breast_cancer_primary | 13 | 0.41 (0.24-0.71) | 0.29 | Ineligible |
| T-DXd | Tachycardia | breast_cancer_primary | 13 | 0.41 (0.24-0.71) | 0.29 | Ineligible |
| T-DXd | Wrong technique in product usage process | breast_cancer_primary | 13 | 1.16 (0.67-2.02) | 0.69 | Ineligible |
| T-DXd | Wrong technique in product usage process | breast_cancer_primary | 13 | 1.16 (0.67-2.02) | 0.69 | Ineligible |
| T-DXd | Balance disorder | breast_cancer_primary | 12 | 0.55 (0.31-0.97) | 0.36 | Ineligible |
| T-DXd | Balance disorder | breast_cancer_primary | 12 | 0.55 (0.31-0.97) | 0.36 | Ineligible |
| T-DXd | COVID-19 pneumonia | breast_cancer_primary | 12 | 4.69 (2.58-8.53) | 2.20 | Eligible |
| T-DXd | Cerebral haemorrhage | breast_cancer_primary | 12 | 1.56 (0.88-2.78) | 0.84 | Ineligible |
| T-DXd | Cerebral haemorrhage | breast_cancer_primary | 12 | 1.56 (0.88-2.78) | 0.84 | Ineligible |
| T-DXd | Hypertension | breast_cancer_primary | 12 | 0.17 (0.10-0.31) | 0.13 | Ineligible |
| T-DXd | Hypertension | breast_cancer_primary | 12 | 0.17 (0.10-0.31) | 0.13 | Ineligible |
| T-DXd | Loss of personal independence in daily activities | breast_cancer_primary | 12 | 1.41 (0.80-2.52) | 0.78 | Ineligible |
| T-DXd | Loss of personal independence in daily activities | breast_cancer_primary | 12 | 1.41 (0.80-2.52) | 0.78 | Ineligible |
| T-DXd | Lower respiratory tract infection | breast_cancer_primary | 12 | 1.01 (0.57-1.79) | 0.60 | Ineligible |
| T-DXd | Lower respiratory tract infection | breast_cancer_primary | 12 | 1.01 (0.57-1.79) | 0.60 | Ineligible |
| T-DXd | Multiple organ dysfunction syndrome | breast_cancer_primary | 12 | 1.01 (0.57-1.80) | 0.61 | Ineligible |
| T-DXd | Multiple organ dysfunction syndrome | breast_cancer_primary | 12 | 1.01 (0.57-1.80) | 0.61 | Ineligible |
| T-DXd | Myocardial infarction | breast_cancer_primary | 12 | 0.63 (0.36-1.12) | 0.41 | Ineligible |
| T-DXd | Myocardial infarction | breast_cancer_primary | 12 | 0.63 (0.36-1.12) | 0.41 | Ineligible |
| T-DXd | Oedema | breast_cancer_primary | 12 | 0.59 (0.33-1.04) | 0.39 | Ineligible |
| T-DXd | Oedema | breast_cancer_primary | 12 | 0.59 (0.33-1.04) | 0.39 | Ineligible |
| T-DXd | Osteonecrosis of jaw | breast_cancer_primary | 12 | 0.17 (0.09-0.29) | 0.12 | Ineligible |
| T-DXd | Osteonecrosis of jaw | breast_cancer_primary | 12 | 0.17 (0.09-0.29) | 0.12 | Ineligible |
| T-DXd | Pulmonary thrombosis | breast_cancer_primary | 12 | 2.04 (1.14-3.65) | 1.03 | Eligible |
| T-DXd | Radiation pneumonitis | breast_cancer_primary | 12 | 3.12 (1.73-5.61) | 1.44 | Eligible |
| T-DXd | Red blood cell count decreased | breast_cancer_primary | 12 | 0.21 (0.12-0.38) | 0.15 | Ineligible |
| T-DXd | Red blood cell count decreased | breast_cancer_primary | 12 | 0.21 (0.12-0.38) | 0.15 | Ineligible |
| T-DXd | Respiratory distress | breast_cancer_primary | 12 | 1.60 (0.90-2.85) | 0.86 | Ineligible |
| T-DXd | Respiratory distress | breast_cancer_primary | 12 | 1.60 (0.90-2.85) | 0.86 | Ineligible |
| T-DXd | Therapy cessation | breast_cancer_primary | 12 | 0.55 (0.31-0.97) | 0.36 | Ineligible |
| T-DXd | Therapy cessation | breast_cancer_primary | 12 | 0.55 (0.31-0.97) | 0.36 | Ineligible |
| T-DXd | Blood creatinine increased | breast_cancer_primary | 11 | 0.24 (0.13-0.44) | 0.17 | Ineligible |
| T-DXd | Blood creatinine increased | breast_cancer_primary | 11 | 0.24 (0.13-0.44) | 0.17 | Ineligible |
| T-DXd | Breast cancer metastatic | breast_cancer_primary | 11 | 0.09 (0.05-0.16) | 0.07 | Ineligible |
| T-DXd | Breast cancer metastatic | breast_cancer_primary | 11 | 0.09 (0.05-0.16) | 0.07 | Ineligible |
| T-DXd | Deep vein thrombosis | breast_cancer_primary | 11 | 0.49 (0.27-0.89) | 0.32 | Ineligible |
| T-DXd | Deep vein thrombosis | breast_cancer_primary | 11 | 0.49 (0.27-0.89) | 0.32 | Ineligible |
| T-DXd | Disease complication | breast_cancer_primary | 11 | 8.07 (4.22-15.40) | 3.69 | Eligible |
| T-DXd | Electrolyte imbalance | breast_cancer_primary | 11 | 1.68 (0.92-3.07) | 0.86 | Ineligible |
| T-DXd | Electrolyte imbalance | breast_cancer_primary | 11 | 1.68 (0.92-3.07) | 0.86 | Ineligible |
| T-DXd | Gastrointestinal haemorrhage | breast_cancer_primary | 11 | 1.12 (0.61-2.04) | 0.64 | Ineligible |
| T-DXd | Gastrointestinal haemorrhage | breast_cancer_primary | 11 | 1.12 (0.61-2.04) | 0.64 | Ineligible |
| T-DXd | Hepatic lesion | breast_cancer_primary | 11 | 1.02 (0.56-1.85) | 0.59 | Ineligible |
| T-DXd | Hepatic lesion | breast_cancer_primary | 11 | 1.02 (0.56-1.85) | 0.59 | Ineligible |
| T-DXd | Hypoglycaemia | breast_cancer_primary | 11 | 1.46 (0.80-2.67) | 0.78 | Ineligible |
| T-DXd | Hypoglycaemia | breast_cancer_primary | 11 | 1.46 (0.80-2.67) | 0.78 | Ineligible |
| T-DXd | Hypophagia | breast_cancer_primary | 11 | 0.88 (0.49-1.60) | 0.53 | Ineligible |
| T-DXd | Hypophagia | breast_cancer_primary | 11 | 0.88 (0.49-1.60) | 0.53 | Ineligible |
| T-DXd | Immunodeficiency | breast_cancer_primary | 11 | 1.29 (0.71-2.36) | 0.71 | Ineligible |
| T-DXd | Immunodeficiency | breast_cancer_primary | 11 | 1.29 (0.71-2.36) | 0.71 | Ineligible |
| T-DXd | Lethargy | breast_cancer_primary | 11 | 0.79 (0.43-1.43) | 0.48 | Ineligible |
| T-DXd | Lethargy | breast_cancer_primary | 11 | 0.79 (0.43-1.43) | 0.48 | Ineligible |
| T-DXd | Memory impairment | breast_cancer_primary | 11 | 0.21 (0.12-0.38) | 0.15 | Ineligible |
| T-DXd | Memory impairment | breast_cancer_primary | 11 | 0.21 (0.12-0.38) | 0.15 | Ineligible |
| T-DXd | Mental disorder | breast_cancer_primary | 11 | 1.12 (0.62-2.05) | 0.64 | Ineligible |
| T-DXd | Mental disorder | breast_cancer_primary | 11 | 1.12 (0.62-2.05) | 0.64 | Ineligible |
| T-DXd | Palmar-plantar erythrodysaesthesia syndrome | breast_cancer_primary | 11 | 0.16 (0.09-0.28) | 0.11 | Ineligible |
| T-DXd | Palmar-plantar erythrodysaesthesia syndrome | breast_cancer_primary | 11 | 0.16 (0.09-0.28) | 0.11 | Ineligible |
| T-DXd | Pulmonary mass | breast_cancer_primary | 11 | 0.65 (0.36-1.18) | 0.41 | Ineligible |
| T-DXd | Pulmonary mass | breast_cancer_primary | 11 | 0.65 (0.36-1.18) | 0.41 | Ineligible |
| T-DXd | Skin discolouration | breast_cancer_primary | 11 | 0.46 (0.26-0.84) | 0.31 | Ineligible |
| T-DXd | Skin discolouration | breast_cancer_primary | 11 | 0.46 (0.26-0.84) | 0.31 | Ineligible |
| T-DXd | Therapy partial responder | breast_cancer_primary | 11 | 0.75 (0.41-1.36) | 0.47 | Ineligible |
| T-DXd | Therapy partial responder | breast_cancer_primary | 11 | 0.75 (0.41-1.36) | 0.47 | Ineligible |
| T-DXd | Agranulocytosis | breast_cancer_primary | 10 | 0.37 (0.20-0.70) | 0.25 | Ineligible |
| T-DXd | Agranulocytosis | breast_cancer_primary | 10 | 0.37 (0.20-0.70) | 0.25 | Ineligible |
| T-DXd | Atrial fibrillation | breast_cancer_primary | 10 | 0.34 (0.18-0.63) | 0.23 | Ineligible |
| T-DXd | Atrial fibrillation | breast_cancer_primary | 10 | 0.34 (0.18-0.63) | 0.23 | Ineligible |
| T-DXd | Blood lactate dehydrogenase increased | breast_cancer_primary | 10 | 0.80 (0.43-1.50) | 0.48 | Ineligible |
| T-DXd | Blood lactate dehydrogenase increased | breast_cancer_primary | 10 | 0.80 (0.43-1.50) | 0.48 | Ineligible |
| T-DXd | Cardiomyopathy | breast_cancer_primary | 10 | 0.68 (0.36-1.27) | 0.42 | Ineligible |
| T-DXd | Cardiomyopathy | breast_cancer_primary | 10 | 0.68 (0.36-1.27) | 0.42 | Ineligible |
| T-DXd | Device related infection | breast_cancer_primary | 10 | 1.05 (0.56-1.98) | 0.59 | Ineligible |
| T-DXd | Device related infection | breast_cancer_primary | 10 | 1.05 (0.56-1.98) | 0.59 | Ineligible |
| T-DXd | Discomfort | breast_cancer_primary | 10 | 0.33 (0.18-0.61) | 0.22 | Ineligible |
| T-DXd | Discomfort | breast_cancer_primary | 10 | 0.33 (0.18-0.61) | 0.22 | Ineligible |
| T-DXd | Enterocolitis | breast_cancer_primary | 10 | 2.03 (1.08-3.83) | 0.96 | Eligible |
| T-DXd | Full blood count decreased | breast_cancer_primary | 10 | 0.25 (0.14-0.47) | 0.18 | Ineligible |
| T-DXd | Full blood count decreased | breast_cancer_primary | 10 | 0.25 (0.14-0.47) | 0.18 | Ineligible |
| T-DXd | Generalised oedema | breast_cancer_primary | 10 | 1.82 (0.96-3.42) | 0.88 | Ineligible |
| T-DXd | Generalised oedema | breast_cancer_primary | 10 | 1.82 (0.96-3.42) | 0.88 | Ineligible |
| T-DXd | Haemolytic anaemia | breast_cancer_primary | 10 | 3.02 (1.59-5.74) | 1.29 | Eligible |
| T-DXd | Hepatitis | breast_cancer_primary | 10 | 0.69 (0.37-1.28) | 0.42 | Ineligible |
| T-DXd | Hepatitis | breast_cancer_primary | 10 | 0.69 (0.37-1.28) | 0.42 | Ineligible |
| T-DXd | Herpes zoster | breast_cancer_primary | 10 | 0.60 (0.32-1.12) | 0.38 | Ineligible |
| T-DXd | Herpes zoster | breast_cancer_primary | 10 | 0.60 (0.32-1.12) | 0.38 | Ineligible |
| T-DXd | Hyperglycaemia | breast_cancer_primary | 10 | 0.21 (0.11-0.39) | 0.15 | Ineligible |
| T-DXd | Hyperglycaemia | breast_cancer_primary | 10 | 0.21 (0.11-0.39) | 0.15 | Ineligible |
| T-DXd | Hypersensitivity | breast_cancer_primary | 10 | 0.18 (0.09-0.33) | 0.12 | Ineligible |
| T-DXd | Hypersensitivity | breast_cancer_primary | 10 | 0.18 (0.09-0.33) | 0.12 | Ineligible |
| T-DXd | Hypomagnesaemia | breast_cancer_primary | 10 | 1.57 (0.84-2.96) | 0.80 | Ineligible |
| T-DXd | Hypomagnesaemia | breast_cancer_primary | 10 | 1.57 (0.84-2.96) | 0.80 | Ineligible |
| T-DXd | Keratitis | breast_cancer_primary | 10 | 4.06 (2.12-7.79) | 1.69 | Eligible |
| T-DXd | Liver function test abnormal | breast_cancer_primary | 10 | 0.88 (0.47-1.64) | 0.51 | Ineligible |
| T-DXd | Liver function test abnormal | breast_cancer_primary | 10 | 0.88 (0.47-1.64) | 0.51 | Ineligible |
| T-DXd | Liver injury | breast_cancer_primary | 10 | 0.62 (0.33-1.15) | 0.39 | Ineligible |
| T-DXd | Liver injury | breast_cancer_primary | 10 | 0.62 (0.33-1.15) | 0.39 | Ineligible |
| T-DXd | Metastases to skin | breast_cancer_primary | 10 | 1.11 (0.59-2.09) | 0.62 | Ineligible |
| T-DXd | Metastases to skin | breast_cancer_primary | 10 | 1.11 (0.59-2.09) | 0.62 | Ineligible |
| T-DXd | Ocular toxicity | breast_cancer_primary | 10 | 22.81 (10.53-49.44) | 6.74 | Eligible |
| T-DXd | Pneumocystis jirovecii infection | breast_cancer_primary | 10 | 9.12 (4.60-18.11) | 3.91 | Eligible |
| T-DXd | Therapy interrupted | breast_cancer_primary | 10 | 0.51 (0.27-0.95) | 0.33 | Ineligible |
| T-DXd | Therapy interrupted | breast_cancer_primary | 10 | 0.51 (0.27-0.95) | 0.33 | Ineligible |
| T-DXd | Treatment noncompliance | breast_cancer_primary | 10 | 2.60 (1.37-4.92) | 1.14 | Eligible |
| T-DXd | Tremor | breast_cancer_primary | 10 | 0.29 (0.16-0.55) | 0.20 | Ineligible |
| T-DXd | Tremor | breast_cancer_primary | 10 | 0.29 (0.16-0.55) | 0.20 | Ineligible |
| T-DXd | Wheezing | breast_cancer_primary | 10 | 1.13 (0.60-2.12) | 0.63 | Ineligible |
| T-DXd | Wheezing | breast_cancer_primary | 10 | 1.13 (0.60-2.12) | 0.63 | Ineligible |
| T-DXd | Ageusia | breast_cancer_primary | 9 | 0.60 (0.31-1.16) | 0.37 | Ineligible |
| T-DXd | Ageusia | breast_cancer_primary | 9 | 0.60 (0.31-1.16) | 0.37 | Ineligible |
| T-DXd | Aphasia | breast_cancer_primary | 9 | 1.21 (0.63-2.36) | 0.64 | Ineligible |
| T-DXd | Aphasia | breast_cancer_primary | 9 | 1.21 (0.63-2.36) | 0.64 | Ineligible |
| T-DXd | Asthma | breast_cancer_primary | 9 | 0.90 (0.47-1.75) | 0.51 | Ineligible |
| T-DXd | Asthma | breast_cancer_primary | 9 | 0.90 (0.47-1.75) | 0.51 | Ineligible |
| T-DXd | Blood glucose abnormal | breast_cancer_primary | 9 | 1.97 (1.01-3.86) | 0.90 | Ineligible |
| T-DXd | Blood glucose abnormal | breast_cancer_primary | 9 | 1.97 (1.01-3.86) | 0.90 | Ineligible |
| T-DXd | Blood test abnormal | breast_cancer_primary | 9 | 0.62 (0.32-1.19) | 0.38 | Ineligible |
| T-DXd | Blood test abnormal | breast_cancer_primary | 9 | 0.62 (0.32-1.19) | 0.38 | Ineligible |
| T-DXd | C-reactive protein increased | breast_cancer_primary | 9 | 0.50 (0.26-0.97) | 0.32 | Ineligible |
| T-DXd | C-reactive protein increased | breast_cancer_primary | 9 | 0.50 (0.26-0.97) | 0.32 | Ineligible |
| T-DXd | Diabetes mellitus | breast_cancer_primary | 9 | 0.33 (0.17-0.64) | 0.22 | Ineligible |
| T-DXd | Diabetes mellitus | breast_cancer_primary | 9 | 0.33 (0.17-0.64) | 0.22 | Ineligible |
| T-DXd | Eating disorder | breast_cancer_primary | 9 | 0.50 (0.26-0.97) | 0.32 | Ineligible |
| T-DXd | Eating disorder | breast_cancer_primary | 9 | 0.50 (0.26-0.97) | 0.32 | Ineligible |
| T-DXd | Failure to thrive | breast_cancer_primary | 9 | 8.59 (4.19-17.63) | 3.53 | Ineligible |
| T-DXd | Failure to thrive | breast_cancer_primary | 9 | 8.59 (4.19-17.63) | 3.53 | Ineligible |
| T-DXd | Full blood count abnormal | breast_cancer_primary | 9 | 0.48 (0.25-0.92) | 0.30 | Ineligible |
| T-DXd | Full blood count abnormal | breast_cancer_primary | 9 | 0.48 (0.25-0.92) | 0.30 | Ineligible |
| T-DXd | Gait inability | breast_cancer_primary | 9 | 0.65 (0.34-1.27) | 0.40 | Ineligible |
| T-DXd | Gait inability | breast_cancer_primary | 9 | 0.65 (0.34-1.27) | 0.40 | Ineligible |
| T-DXd | General physical condition abnormal | breast_cancer_primary | 9 | 2.51 (1.28-4.93) | 1.06 | Ineligible |
| T-DXd | General physical condition abnormal | breast_cancer_primary | 9 | 2.51 (1.28-4.93) | 1.06 | Ineligible |
| T-DXd | Hip fracture | breast_cancer_primary | 9 | 0.98 (0.51-1.91) | 0.55 | Ineligible |
| T-DXd | Hip fracture | breast_cancer_primary | 9 | 0.98 (0.51-1.91) | 0.55 | Ineligible |
| T-DXd | Hypercalcaemia | breast_cancer_primary | 9 | 1.01 (0.52-1.96) | 0.56 | Ineligible |
| T-DXd | Hypercalcaemia | breast_cancer_primary | 9 | 1.01 (0.52-1.96) | 0.56 | Ineligible |
| T-DXd | Immune system disorder | breast_cancer_primary | 9 | 1.03 (0.53-1.99) | 0.57 | Ineligible |
| T-DXd | Immune system disorder | breast_cancer_primary | 9 | 1.03 (0.53-1.99) | 0.57 | Ineligible |
| T-DXd | Intestinal ischaemia | breast_cancer_primary | 9 | 4.06 (2.05-8.06) | 1.59 | Ineligible |
| T-DXd | Intestinal ischaemia | breast_cancer_primary | 9 | 4.06 (2.05-8.06) | 1.59 | Ineligible |
| T-DXd | Intra-abdominal fluid collection | breast_cancer_primary | 9 | 8.80 (4.28-18.08) | 3.60 | Ineligible |
| T-DXd | Intra-abdominal fluid collection | breast_cancer_primary | 9 | 8.80 (4.28-18.08) | 3.60 | Ineligible |
| T-DXd | Jaundice | breast_cancer_primary | 9 | 0.59 (0.31-1.14) | 0.37 | Ineligible |
| T-DXd | Jaundice | breast_cancer_primary | 9 | 0.59 (0.31-1.14) | 0.37 | Ineligible |
| T-DXd | Laboratory test abnormal | breast_cancer_primary | 9 | 0.58 (0.30-1.12) | 0.36 | Ineligible |
| T-DXd | Laboratory test abnormal | breast_cancer_primary | 9 | 0.58 (0.30-1.12) | 0.36 | Ineligible |
| T-DXd | Lymphocyte count decreased | breast_cancer_primary | 9 | 0.56 (0.29-1.08) | 0.35 | Ineligible |
| T-DXd | Lymphocyte count decreased | breast_cancer_primary | 9 | 0.56 (0.29-1.08) | 0.35 | Ineligible |
| T-DXd | Mental impairment | breast_cancer_primary | 9 | 2.00 (1.02-3.90) | 0.91 | Ineligible |
| T-DXd | Mental impairment | breast_cancer_primary | 9 | 2.00 (1.02-3.90) | 0.91 | Ineligible |
| T-DXd | Metastases to spine | breast_cancer_primary | 9 | 0.51 (0.27-0.99) | 0.33 | Ineligible |
| T-DXd | Metastases to spine | breast_cancer_primary | 9 | 0.51 (0.27-0.99) | 0.33 | Ineligible |
| T-DXd | Neoplasm malignant | breast_cancer_primary | 9 | 0.57 (0.29-1.09) | 0.35 | Ineligible |
| T-DXd | Neoplasm malignant | breast_cancer_primary | 9 | 0.57 (0.29-1.09) | 0.35 | Ineligible |
| T-DXd | Pseudocirrhosis | breast_cancer_primary | 9 | 1.85 (0.95-3.60) | 0.86 | Ineligible |
| T-DXd | Pseudocirrhosis | breast_cancer_primary | 9 | 1.85 (0.95-3.60) | 0.86 | Ineligible |
| T-DXd | Pulmonary hypertension | breast_cancer_primary | 9 | 1.46 (0.75-2.85) | 0.73 | Ineligible |
| T-DXd | Pulmonary hypertension | breast_cancer_primary | 9 | 1.46 (0.75-2.85) | 0.73 | Ineligible |
| T-DXd | Rash pruritic | breast_cancer_primary | 9 | 0.51 (0.26-0.98) | 0.32 | Ineligible |
| T-DXd | Rash pruritic | breast_cancer_primary | 9 | 0.51 (0.26-0.98) | 0.32 | Ineligible |
| T-DXd | Rhinorrhoea | breast_cancer_primary | 9 | 0.36 (0.19-0.70) | 0.24 | Ineligible |
| T-DXd | Rhinorrhoea | breast_cancer_primary | 9 | 0.36 (0.19-0.70) | 0.24 | Ineligible |
| T-DXd | Speech disorder | breast_cancer_primary | 9 | 0.60 (0.31-1.16) | 0.37 | Ineligible |
| T-DXd | Speech disorder | breast_cancer_primary | 9 | 0.60 (0.31-1.16) | 0.37 | Ineligible |
| T-DXd | Swelling | breast_cancer_primary | 9 | 0.37 (0.19-0.72) | 0.25 | Ineligible |
| T-DXd | Swelling | breast_cancer_primary | 9 | 0.37 (0.19-0.72) | 0.25 | Ineligible |
| T-DXd | Tumour lysis syndrome | breast_cancer_primary | 9 | 2.11 (1.08-4.13) | 0.94 | Ineligible |
| T-DXd | Tumour lysis syndrome | breast_cancer_primary | 9 | 2.11 (1.08-4.13) | 0.94 | Ineligible |
| T-DXd | Urosepsis | breast_cancer_primary | 9 | 1.97 (1.01-3.86) | 0.90 | Ineligible |
| T-DXd | Urosepsis | breast_cancer_primary | 9 | 1.97 (1.01-3.86) | 0.90 | Ineligible |
| T-DXd | Altered state of consciousness | breast_cancer_primary | 8 | 1.84 (0.91-3.75) | 0.82 | Ineligible |
| T-DXd | Altered state of consciousness | breast_cancer_primary | 8 | 1.84 (0.91-3.75) | 0.82 | Ineligible |
| T-DXd | Arrhythmia | breast_cancer_primary | 8 | 0.51 (0.25-1.02) | 0.31 | Ineligible |
| T-DXd | Arrhythmia | breast_cancer_primary | 8 | 0.51 (0.25-1.02) | 0.31 | Ineligible |
| T-DXd | Blood magnesium decreased | breast_cancer_primary | 8 | 1.10 (0.55-2.22) | 0.58 | Ineligible |
| T-DXd | Blood magnesium decreased | breast_cancer_primary | 8 | 1.10 (0.55-2.22) | 0.58 | Ineligible |
| T-DXd | Brain neoplasm | breast_cancer_primary | 8 | 1.09 (0.54-2.19) | 0.57 | Ineligible |
| T-DXd | Brain neoplasm | breast_cancer_primary | 8 | 1.09 (0.54-2.19) | 0.57 | Ineligible |
| T-DXd | Cardiac failure congestive | breast_cancer_primary | 8 | 0.46 (0.23-0.92) | 0.29 | Ineligible |
| T-DXd | Cardiac failure congestive | breast_cancer_primary | 8 | 0.46 (0.23-0.92) | 0.29 | Ineligible |
| T-DXd | Coma | breast_cancer_primary | 8 | 1.50 (0.74-3.04) | 0.71 | Ineligible |
| T-DXd | Coma | breast_cancer_primary | 8 | 1.50 (0.74-3.04) | 0.71 | Ineligible |
| T-DXd | Computerised tomogram abnormal | breast_cancer_primary | 8 | 5.13 (2.46-10.70) | 1.86 | Ineligible |
| T-DXd | Computerised tomogram abnormal | breast_cancer_primary | 8 | 5.13 (2.46-10.70) | 1.86 | Ineligible |
| T-DXd | Cystitis | breast_cancer_primary | 8 | 0.50 (0.25-1.01) | 0.31 | Ineligible |
| T-DXd | Cystitis | breast_cancer_primary | 8 | 0.50 (0.25-1.01) | 0.31 | Ineligible |
| T-DXd | Dysphonia | breast_cancer_primary | 8 | 0.37 (0.19-0.75) | 0.24 | Ineligible |
| T-DXd | Dysphonia | breast_cancer_primary | 8 | 0.37 (0.19-0.75) | 0.24 | Ineligible |
| T-DXd | Enteritis | breast_cancer_primary | 8 | 2.47 (1.21-5.04) | 0.99 | Ineligible |
| T-DXd | Enteritis | breast_cancer_primary | 8 | 2.47 (1.21-5.04) | 0.99 | Ineligible |
| T-DXd | Epilepsy | breast_cancer_primary | 8 | 1.01 (0.50-2.04) | 0.54 | Ineligible |
| T-DXd | Epilepsy | breast_cancer_primary | 8 | 1.01 (0.50-2.04) | 0.54 | Ineligible |
| T-DXd | Hypocalcaemia | breast_cancer_primary | 8 | 0.56 (0.28-1.13) | 0.34 | Ineligible |
| T-DXd | Hypocalcaemia | breast_cancer_primary | 8 | 0.56 (0.28-1.13) | 0.34 | Ineligible |
| T-DXd | Immune thrombocytopenia | breast_cancer_primary | 8 | 3.07 (1.50-6.30) | 1.16 | Ineligible |
| T-DXd | Immune thrombocytopenia | breast_cancer_primary | 8 | 3.07 (1.50-6.30) | 1.16 | Ineligible |
| T-DXd | Immunosuppression | breast_cancer_primary | 8 | 1.91 (0.94-3.88) | 0.84 | Ineligible |
| T-DXd | Immunosuppression | breast_cancer_primary | 8 | 1.91 (0.94-3.88) | 0.84 | Ineligible |
| T-DXd | Intentional dose omission | breast_cancer_primary | 8 | 1.58 (0.78-3.20) | 0.74 | Ineligible |
| T-DXd | Intentional dose omission | breast_cancer_primary | 8 | 1.58 (0.78-3.20) | 0.74 | Ineligible |
| T-DXd | Limb discomfort | breast_cancer_primary | 8 | 0.61 (0.30-1.23) | 0.37 | Ineligible |
| T-DXd | Limb discomfort | breast_cancer_primary | 8 | 0.61 (0.30-1.23) | 0.37 | Ineligible |
| T-DXd | Lung neoplasm | breast_cancer_primary | 8 | 3.73 (1.81-7.70) | 1.36 | Ineligible |
| T-DXd | Lung neoplasm | breast_cancer_primary | 8 | 3.73 (1.81-7.70) | 1.36 | Ineligible |
| T-DXd | Metabolic acidosis | breast_cancer_primary | 8 | 1.82 (0.90-3.70) | 0.81 | Ineligible |
| T-DXd | Metabolic acidosis | breast_cancer_primary | 8 | 1.82 (0.90-3.70) | 0.81 | Ineligible |
| T-DXd | Nervous system disorder | breast_cancer_primary | 8 | 1.02 (0.50-2.06) | 0.54 | Ineligible |
| T-DXd | Nervous system disorder | breast_cancer_primary | 8 | 1.02 (0.50-2.06) | 0.54 | Ineligible |
| T-DXd | Organising pneumonia | breast_cancer_primary | 8 | 0.96 (0.47-1.93) | 0.52 | Ineligible |
| T-DXd | Organising pneumonia | breast_cancer_primary | 8 | 0.96 (0.47-1.93) | 0.52 | Ineligible |
| T-DXd | Pathological fracture | breast_cancer_primary | 8 | 1.05 (0.52-2.13) | 0.56 | Ineligible |
| T-DXd | Pathological fracture | breast_cancer_primary | 8 | 1.05 (0.52-2.13) | 0.56 | Ineligible |
| T-DXd | Portal hypertension | breast_cancer_primary | 8 | 1.90 (0.93-3.86) | 0.83 | Ineligible |
| T-DXd | Portal hypertension | breast_cancer_primary | 8 | 1.90 (0.93-3.86) | 0.83 | Ineligible |
| T-DXd | Upper gastrointestinal haemorrhage | breast_cancer_primary | 8 | 3.28 (1.60-6.75) | 1.23 | Ineligible |
| T-DXd | Upper gastrointestinal haemorrhage | breast_cancer_primary | 8 | 3.28 (1.60-6.75) | 1.23 | Ineligible |
| T-DXd | Visual acuity reduced | breast_cancer_primary | 8 | 1.09 (0.54-2.21) | 0.57 | Ineligible |
| T-DXd | Visual acuity reduced | breast_cancer_primary | 8 | 1.09 (0.54-2.21) | 0.57 | Ineligible |
| T-DXd | Abdominal cavity drainage | breast_cancer_primary | 7 | 57.46 (18.23-181.09) | 7.38 | Ineligible |
| T-DXd | Abdominal cavity drainage | breast_cancer_primary | 7 | 57.46 (18.23-181.09) | 7.38 | Ineligible |
| T-DXd | Adverse reaction | breast_cancer_primary | 7 | 3.46 (1.60-7.49) | 1.18 | Ineligible |
| T-DXd | Adverse reaction | breast_cancer_primary | 7 | 3.46 (1.60-7.49) | 1.18 | Ineligible |
| T-DXd | Blood albumin decreased | breast_cancer_primary | 7 | 1.60 (0.75-3.41) | 0.71 | Ineligible |
| T-DXd | Blood albumin decreased | breast_cancer_primary | 7 | 1.60 (0.75-3.41) | 0.71 | Ineligible |
| T-DXd | Blood sodium decreased | breast_cancer_primary | 7 | 0.77 (0.36-1.63) | 0.43 | Ineligible |
| T-DXd | Blood sodium decreased | breast_cancer_primary | 7 | 0.77 (0.36-1.63) | 0.43 | Ineligible |
| T-DXd | Breast haemorrhage | breast_cancer_primary | 7 | 4.22 (1.94-9.20) | 1.39 | Ineligible |
| T-DXd | Breast haemorrhage | breast_cancer_primary | 7 | 4.22 (1.94-9.20) | 1.39 | Ineligible |
| T-DXd | Cancer pain | breast_cancer_primary | 7 | 1.10 (0.52-2.34) | 0.55 | Ineligible |
| T-DXd | Cancer pain | breast_cancer_primary | 7 | 1.10 (0.52-2.34) | 0.55 | Ineligible |
| T-DXd | Cerebellar infarction | breast_cancer_primary | 7 | 35.91 (13.02-99.06) | 6.55 | Ineligible |
| T-DXd | Cerebellar infarction | breast_cancer_primary | 7 | 35.91 (13.02-99.06) | 6.55 | Ineligible |
| T-DXd | Cerebral disorder | breast_cancer_primary | 7 | 1.49 (0.70-3.16) | 0.67 | Ineligible |
| T-DXd | Cerebral disorder | breast_cancer_primary | 7 | 1.49 (0.70-3.16) | 0.67 | Ineligible |
| T-DXd | Cystitis haemorrhagic | breast_cancer_primary | 7 | 2.18 (1.02-4.65) | 0.86 | Ineligible |
| T-DXd | Cystitis haemorrhagic | breast_cancer_primary | 7 | 2.18 (1.02-4.65) | 0.86 | Ineligible |
| T-DXd | Drug hypersensitivity | breast_cancer_primary | 7 | 0.32 (0.15-0.68) | 0.21 | Ineligible |
| T-DXd | Drug hypersensitivity | breast_cancer_primary | 7 | 0.32 (0.15-0.68) | 0.21 | Ineligible |
| T-DXd | Dyspnoea exertional | breast_cancer_primary | 7 | 0.31 (0.15-0.64) | 0.20 | Ineligible |
| T-DXd | Dyspnoea exertional | breast_cancer_primary | 7 | 0.31 (0.15-0.64) | 0.20 | Ineligible |
| T-DXd | Emotional distress | breast_cancer_primary | 7 | 0.06 (0.03-0.13) | 0.05 | Ineligible |
| T-DXd | Emotional distress | breast_cancer_primary | 7 | 0.06 (0.03-0.13) | 0.05 | Ineligible |
| T-DXd | Haematemesis | breast_cancer_primary | 7 | 0.92 (0.44-1.95) | 0.49 | Ineligible |
| T-DXd | Haematemesis | breast_cancer_primary | 7 | 0.92 (0.44-1.95) | 0.49 | Ineligible |
| T-DXd | Haematocrit decreased | breast_cancer_primary | 7 | 0.41 (0.19-0.85) | 0.26 | Ineligible |
| T-DXd | Haematocrit decreased | breast_cancer_primary | 7 | 0.41 (0.19-0.85) | 0.26 | Ineligible |
| T-DXd | Hair growth abnormal | breast_cancer_primary | 7 | 1.04 (0.49-2.19) | 0.53 | Ineligible |
| T-DXd | Hair growth abnormal | breast_cancer_primary | 7 | 1.04 (0.49-2.19) | 0.53 | Ineligible |
| T-DXd | Hepatic encephalopathy | breast_cancer_primary | 7 | 2.32 (1.08-4.96) | 0.89 | Ineligible |
| T-DXd | Hepatic encephalopathy | breast_cancer_primary | 7 | 2.32 (1.08-4.96) | 0.89 | Ineligible |
| T-DXd | Hyperbilirubinaemia | breast_cancer_primary | 7 | 0.73 (0.34-1.53) | 0.41 | Ineligible |
| T-DXd | Hyperbilirubinaemia | breast_cancer_primary | 7 | 0.73 (0.34-1.53) | 0.41 | Ineligible |
| T-DXd | Hypoalbuminaemia | breast_cancer_primary | 7 | 1.24 (0.59-2.64) | 0.60 | Ineligible |
| T-DXd | Hypoalbuminaemia | breast_cancer_primary | 7 | 1.24 (0.59-2.64) | 0.60 | Ineligible |
| T-DXd | Inflammation | breast_cancer_primary | 7 | 0.35 (0.17-0.74) | 0.23 | Ineligible |
| T-DXd | Inflammation | breast_cancer_primary | 7 | 0.35 (0.17-0.74) | 0.23 | Ineligible |
| T-DXd | Intentional product misuse | breast_cancer_primary | 7 | 0.60 (0.28-1.26) | 0.35 | Ineligible |
| T-DXd | Intentional product misuse | breast_cancer_primary | 7 | 0.60 (0.28-1.26) | 0.35 | Ineligible |
| T-DXd | Melaena | breast_cancer_primary | 7 | 1.57 (0.74-3.34) | 0.70 | Ineligible |
| T-DXd | Melaena | breast_cancer_primary | 7 | 1.57 (0.74-3.34) | 0.70 | Ineligible |
| T-DXd | Metastases to peritoneum | breast_cancer_primary | 7 | 1.20 (0.57-2.55) | 0.59 | Ineligible |
| T-DXd | Metastases to peritoneum | breast_cancer_primary | 7 | 1.20 (0.57-2.55) | 0.59 | Ineligible |
| T-DXd | Migraine | breast_cancer_primary | 7 | 0.48 (0.23-1.00) | 0.29 | Ineligible |
| T-DXd | Migraine | breast_cancer_primary | 7 | 0.48 (0.23-1.00) | 0.29 | Ineligible |
| T-DXd | Ocular icterus | breast_cancer_primary | 7 | 4.79 (2.19-10.48) | 1.57 | Ineligible |
| T-DXd | Ocular icterus | breast_cancer_primary | 7 | 4.79 (2.19-10.48) | 1.57 | Ineligible |
| T-DXd | Osteomyelitis | breast_cancer_primary | 7 | 0.85 (0.40-1.80) | 0.46 | Ineligible |
| T-DXd | Osteomyelitis | breast_cancer_primary | 7 | 0.85 (0.40-1.80) | 0.46 | Ineligible |
| T-DXd | Product preparation error | breast_cancer_primary | 7 | 4.71 (2.15-10.30) | 1.54 | Ineligible |
| T-DXd | Product preparation error | breast_cancer_primary | 7 | 4.71 (2.15-10.30) | 1.54 | Ineligible |
| T-DXd | Product use complaint | breast_cancer_primary | 7 | 2.54 (1.18-5.46) | 0.95 | Ineligible |
| T-DXd | Product use complaint | breast_cancer_primary | 7 | 2.54 (1.18-5.46) | 0.95 | Ineligible |
| T-DXd | Productive cough | breast_cancer_primary | 7 | 0.43 (0.21-0.91) | 0.27 | Ineligible |
| T-DXd | Productive cough | breast_cancer_primary | 7 | 0.43 (0.21-0.91) | 0.27 | Ineligible |
| T-DXd | Pyelonephritis | breast_cancer_primary | 7 | 1.78 (0.84-3.80) | 0.76 | Ineligible |
| T-DXd | Pyelonephritis | breast_cancer_primary | 7 | 1.78 (0.84-3.80) | 0.76 | Ineligible |
| T-DXd | Restlessness | breast_cancer_primary | 7 | 0.94 (0.45-2.00) | 0.50 | Ineligible |
| T-DXd | Restlessness | breast_cancer_primary | 7 | 0.94 (0.45-2.00) | 0.50 | Ineligible |
| T-DXd | Skin lesion | breast_cancer_primary | 7 | 0.51 (0.24-1.08) | 0.31 | Ineligible |
| T-DXd | Skin lesion | breast_cancer_primary | 7 | 0.51 (0.24-1.08) | 0.31 | Ineligible |
| T-DXd | Skin toxicity | breast_cancer_primary | 7 | 0.54 (0.26-1.13) | 0.32 | Ineligible |
| T-DXd | Skin toxicity | breast_cancer_primary | 7 | 0.54 (0.26-1.13) | 0.32 | Ineligible |
| T-DXd | Spinal compression fracture | breast_cancer_primary | 7 | 1.67 (0.78-3.56) | 0.73 | Ineligible |
| T-DXd | Spinal compression fracture | breast_cancer_primary | 7 | 1.67 (0.78-3.56) | 0.73 | Ineligible |
| T-DXd | Splenomegaly | breast_cancer_primary | 7 | 2.33 (1.09-5.00) | 0.90 | Ineligible |
| T-DXd | Splenomegaly | breast_cancer_primary | 7 | 2.33 (1.09-5.00) | 0.90 | Ineligible |
| T-DXd | Terminal state | breast_cancer_primary | 7 | 2.21 (1.03-4.73) | 0.87 | Ineligible |
| T-DXd | Terminal state | breast_cancer_primary | 7 | 2.21 (1.03-4.73) | 0.87 | Ineligible |
| T-DXd | Transient ischaemic attack | breast_cancer_primary | 7 | 1.13 (0.53-2.40) | 0.56 | Ineligible |
| T-DXd | Transient ischaemic attack | breast_cancer_primary | 7 | 1.13 (0.53-2.40) | 0.56 | Ineligible |
| T-DXd | Unresponsive to stimuli | breast_cancer_primary | 7 | 2.19 (1.02-4.69) | 0.86 | Ineligible |
| T-DXd | Unresponsive to stimuli | breast_cancer_primary | 7 | 2.19 (1.02-4.69) | 0.86 | Ineligible |
| T-DXd | Vertigo | breast_cancer_primary | 7 | 0.33 (0.16-0.69) | 0.21 | Ineligible |
| T-DXd | Vertigo | breast_cancer_primary | 7 | 0.33 (0.16-0.69) | 0.21 | Ineligible |
| T-DXd | Viral infection | breast_cancer_primary | 7 | 0.75 (0.36-1.59) | 0.42 | Ineligible |
| T-DXd | Viral infection | breast_cancer_primary | 7 | 0.75 (0.36-1.59) | 0.42 | Ineligible |
| T-DXd | Atelectasis | breast_cancer_primary | 6 | 0.66 (0.30-1.49) | 0.37 | Ineligible |
| T-DXd | Atelectasis | breast_cancer_primary | 6 | 0.66 (0.30-1.49) | 0.37 | Ineligible |
| T-DXd | Blindness | breast_cancer_primary | 6 | 0.62 (0.28-1.38) | 0.35 | Ineligible |
| T-DXd | Blindness | breast_cancer_primary | 6 | 0.62 (0.28-1.38) | 0.35 | Ineligible |
| T-DXd | Blood alkaline phosphatase abnormal | breast_cancer_primary | 6 | 9.12 (3.76-22.09) | 2.70 | Ineligible |
| T-DXd | Blood alkaline phosphatase abnormal | breast_cancer_primary | 6 | 9.12 (3.76-22.09) | 2.70 | Ineligible |
| T-DXd | Blood calcium decreased | breast_cancer_primary | 6 | 0.62 (0.28-1.39) | 0.35 | Ineligible |
| T-DXd | Blood calcium decreased | breast_cancer_primary | 6 | 0.62 (0.28-1.39) | 0.35 | Ineligible |
| T-DXd | Bone lesion | breast_cancer_primary | 6 | 0.49 (0.22-1.09) | 0.29 | Ineligible |
| T-DXd | Bone lesion | breast_cancer_primary | 6 | 0.49 (0.22-1.09) | 0.29 | Ineligible |
| T-DXd | Cholangitis | breast_cancer_primary | 6 | 2.68 (1.17-6.11) | 0.91 | Ineligible |
| T-DXd | Cholangitis | breast_cancer_primary | 6 | 2.68 (1.17-6.11) | 0.91 | Ineligible |
| T-DXd | Cognitive disorder | breast_cancer_primary | 6 | 0.40 (0.18-0.89) | 0.24 | Ineligible |
| T-DXd | Cognitive disorder | breast_cancer_primary | 6 | 0.40 (0.18-0.89) | 0.24 | Ineligible |
| T-DXd | Contusion | breast_cancer_primary | 6 | 0.25 (0.11-0.57) | 0.17 | Ineligible |
| T-DXd | Contusion | breast_cancer_primary | 6 | 0.25 (0.11-0.57) | 0.17 | Ineligible |
| T-DXd | Depressed mood | breast_cancer_primary | 6 | 0.26 (0.12-0.57) | 0.17 | Ineligible |
| T-DXd | Depressed mood | breast_cancer_primary | 6 | 0.26 (0.12-0.57) | 0.17 | Ineligible |
| T-DXd | Diplopia | breast_cancer_primary | 6 | 0.81 (0.36-1.82) | 0.43 | Ineligible |
| T-DXd | Diplopia | breast_cancer_primary | 6 | 0.81 (0.36-1.82) | 0.43 | Ineligible |
| T-DXd | Disseminated intravascular coagulation | breast_cancer_primary | 6 | 1.10 (0.49-2.48) | 0.53 | Ineligible |
| T-DXd | Disseminated intravascular coagulation | breast_cancer_primary | 6 | 1.10 (0.49-2.48) | 0.53 | Ineligible |
| T-DXd | Ear discomfort | breast_cancer_primary | 6 | 3.57 (1.55-8.22) | 1.10 | Ineligible |
| T-DXd | Ear discomfort | breast_cancer_primary | 6 | 3.57 (1.55-8.22) | 1.10 | Ineligible |
| T-DXd | Eye haematoma | breast_cancer_primary | 6 | 246.22 (29.64-2045.56) | 7.78 | Ineligible |
| T-DXd | Eye haematoma | breast_cancer_primary | 6 | 246.22 (29.64-2045.56) | 7.78 | Ineligible |
| T-DXd | Faeces discoloured | breast_cancer_primary | 6 | 1.37 (0.61-3.10) | 0.61 | Ineligible |
| T-DXd | Faeces discoloured | breast_cancer_primary | 6 | 1.37 (0.61-3.10) | 0.61 | Ineligible |
| T-DXd | Femoral neck fracture | breast_cancer_primary | 6 | 2.22 (0.97-5.04) | 0.81 | Ineligible |
| T-DXd | Femoral neck fracture | breast_cancer_primary | 6 | 2.22 (0.97-5.04) | 0.81 | Ineligible |
| T-DXd | Food aversion | breast_cancer_primary | 6 | 4.24 (1.83-9.84) | 1.26 | Ineligible |
| T-DXd | Food aversion | breast_cancer_primary | 6 | 4.24 (1.83-9.84) | 1.26 | Ineligible |
| T-DXd | Gastritis | breast_cancer_primary | 6 | 0.38 (0.17-0.85) | 0.24 | Ineligible |
| T-DXd | Gastritis | breast_cancer_primary | 6 | 0.38 (0.17-0.85) | 0.24 | Ineligible |
| T-DXd | Gastroenteritis listeria | breast_cancer_primary | 6 | 533.45 (30.05-9470.69) | 8.23 | Ineligible |
| T-DXd | Gastroenteritis listeria | breast_cancer_primary | 6 | 533.45 (30.05-9470.69) | 8.23 | Ineligible |
| T-DXd | Haemoptysis | breast_cancer_primary | 6 | 1.08 (0.48-2.43) | 0.52 | Ineligible |
| T-DXd | Haemoptysis | breast_cancer_primary | 6 | 1.08 (0.48-2.43) | 0.52 | Ineligible |
| T-DXd | Hepatic cyst | breast_cancer_primary | 6 | 1.52 (0.67-3.43) | 0.64 | Ineligible |
| T-DXd | Hepatic cyst | breast_cancer_primary | 6 | 1.52 (0.67-3.43) | 0.64 | Ineligible |
| T-DXd | Hepatomegaly | breast_cancer_primary | 6 | 1.00 (0.45-2.26) | 0.49 | Ineligible |
| T-DXd | Hepatomegaly | breast_cancer_primary | 6 | 1.00 (0.45-2.26) | 0.49 | Ineligible |
| T-DXd | Hospice care | breast_cancer_primary | 6 | 0.68 (0.30-1.51) | 0.37 | Ineligible |
| T-DXd | Hospice care | breast_cancer_primary | 6 | 0.68 (0.30-1.51) | 0.37 | Ineligible |
| T-DXd | Hyperkalaemia | breast_cancer_primary | 6 | 0.88 (0.39-1.99) | 0.45 | Ineligible |
| T-DXd | Hyperkalaemia | breast_cancer_primary | 6 | 0.88 (0.39-1.99) | 0.45 | Ineligible |
| T-DXd | Hypertransaminasaemia | breast_cancer_primary | 6 | 0.51 (0.23-1.14) | 0.30 | Ineligible |
| T-DXd | Hypertransaminasaemia | breast_cancer_primary | 6 | 0.51 (0.23-1.14) | 0.30 | Ineligible |
| T-DXd | Infusion site extravasation | breast_cancer_primary | 6 | 4.40 (1.89-10.21) | 1.29 | Ineligible |
| T-DXd | Infusion site extravasation | breast_cancer_primary | 6 | 4.40 (1.89-10.21) | 1.29 | Ineligible |
| T-DXd | Joint swelling | breast_cancer_primary | 6 | 0.29 (0.13-0.64) | 0.19 | Ineligible |
| T-DXd | Joint swelling | breast_cancer_primary | 6 | 0.29 (0.13-0.64) | 0.19 | Ineligible |
| T-DXd | Lymphopenia | breast_cancer_primary | 6 | 0.57 (0.26-1.28) | 0.33 | Ineligible |
| T-DXd | Lymphopenia | breast_cancer_primary | 6 | 0.57 (0.26-1.28) | 0.33 | Ineligible |
| T-DXd | Medication error | breast_cancer_primary | 6 | 1.57 (0.69-3.54) | 0.66 | Ineligible |
| T-DXd | Medication error | breast_cancer_primary | 6 | 1.57 (0.69-3.54) | 0.66 | Ineligible |
| T-DXd | Mouth ulceration | breast_cancer_primary | 6 | 0.33 (0.15-0.73) | 0.21 | Ineligible |
| T-DXd | Mouth ulceration | breast_cancer_primary | 6 | 0.33 (0.15-0.73) | 0.21 | Ineligible |
| T-DXd | Necrotising fasciitis | breast_cancer_primary | 6 | 4.04 (1.74-9.34) | 1.21 | Ineligible |
| T-DXd | Necrotising fasciitis | breast_cancer_primary | 6 | 4.04 (1.74-9.34) | 1.21 | Ineligible |
| T-DXd | Neurological symptom | breast_cancer_primary | 6 | 3.24 (1.41-7.44) | 1.03 | Ineligible |
| T-DXd | Neurological symptom | breast_cancer_primary | 6 | 3.24 (1.41-7.44) | 1.03 | Ineligible |
| T-DXd | Parosmia | breast_cancer_primary | 6 | 1.56 (0.69-3.52) | 0.65 | Ineligible |
| T-DXd | Parosmia | breast_cancer_primary | 6 | 1.56 (0.69-3.52) | 0.65 | Ineligible |
| T-DXd | Pneumonia cytomegaloviral | breast_cancer_primary | 6 | 27.36 (9.73-76.88) | 5.39 | Ineligible |
| T-DXd | Pneumonia cytomegaloviral | breast_cancer_primary | 6 | 27.36 (9.73-76.88) | 5.39 | Ineligible |
| T-DXd | Rash vesicular | breast_cancer_primary | 6 | 3.20 (1.39-7.34) | 1.02 | Ineligible |
| T-DXd | Rash vesicular | breast_cancer_primary | 6 | 3.20 (1.39-7.34) | 1.02 | Ineligible |
| T-DXd | Respiratory tract infection | breast_cancer_primary | 6 | 0.77 (0.34-1.74) | 0.41 | Ineligible |
| T-DXd | Respiratory tract infection | breast_cancer_primary | 6 | 0.77 (0.34-1.74) | 0.41 | Ineligible |
| T-DXd | Skin fissures | breast_cancer_primary | 6 | 0.46 (0.21-1.03) | 0.28 | Ineligible |
| T-DXd | Skin fissures | breast_cancer_primary | 6 | 0.46 (0.21-1.03) | 0.28 | Ineligible |
| T-DXd | Skin haemorrhage | breast_cancer_primary | 6 | 1.91 (0.84-4.33) | 0.74 | Ineligible |
| T-DXd | Skin haemorrhage | breast_cancer_primary | 6 | 1.91 (0.84-4.33) | 0.74 | Ineligible |
| T-DXd | Urinary retention | breast_cancer_primary | 6 | 0.93 (0.41-2.09) | 0.47 | Ineligible |
| T-DXd | Urinary retention | breast_cancer_primary | 6 | 0.93 (0.41-2.09) | 0.47 | Ineligible |
| T-DXd | Weight fluctuation | breast_cancer_primary | 6 | 0.78 (0.35-1.76) | 0.42 | Ineligible |
| T-DXd | Weight fluctuation | breast_cancer_primary | 6 | 0.78 (0.35-1.76) | 0.42 | Ineligible |
| T-DXd | White blood cell count increased | breast_cancer_primary | 6 | 0.50 (0.23-1.13) | 0.30 | Ineligible |
| T-DXd | White blood cell count increased | breast_cancer_primary | 6 | 0.50 (0.23-1.13) | 0.30 | Ineligible |
| T-DXd | Agitation | breast_cancer_primary | 5 | 0.63 (0.26-1.52) | 0.34 | Ineligible |
| T-DXd | Agitation | breast_cancer_primary | 5 | 0.63 (0.26-1.52) | 0.34 | Ineligible |
| T-DXd | Amnesia | breast_cancer_primary | 5 | 0.32 (0.13-0.76) | 0.20 | Ineligible |
| T-DXd | Amnesia | breast_cancer_primary | 5 | 0.32 (0.13-0.76) | 0.20 | Ineligible |
| T-DXd | Anticoagulation drug level below therapeutic | breast_cancer_primary | 5 | 451.31 (24.95-8162.99) | 6.98 | Ineligible |
| T-DXd | Anticoagulation drug level below therapeutic | breast_cancer_primary | 5 | 451.31 (24.95-8162.99) | 6.98 | Ineligible |
| T-DXd | Appetite disorder | breast_cancer_primary | 5 | 1.17 (0.48-2.85) | 0.51 | Ineligible |
| T-DXd | Appetite disorder | breast_cancer_primary | 5 | 1.17 (0.48-2.85) | 0.51 | Ineligible |
| T-DXd | Arteriosclerosis coronary artery | breast_cancer_primary | 5 | 2.77 (1.12-6.86) | 0.84 | Ineligible |
| T-DXd | Arteriosclerosis coronary artery | breast_cancer_primary | 5 | 2.77 (1.12-6.86) | 0.84 | Ineligible |
| T-DXd | Bacteraemia | breast_cancer_primary | 5 | 1.21 (0.50-2.95) | 0.53 | Ineligible |
| T-DXd | Bacteraemia | breast_cancer_primary | 5 | 1.21 (0.50-2.95) | 0.53 | Ineligible |
| T-DXd | Blister | breast_cancer_primary | 5 | 0.21 (0.09-0.50) | 0.14 | Ineligible |
| T-DXd | Blister | breast_cancer_primary | 5 | 0.21 (0.09-0.50) | 0.14 | Ineligible |
| T-DXd | Bone marrow failure | breast_cancer_primary | 5 | 0.12 (0.05-0.28) | 0.08 | Ineligible |
| T-DXd | Bone marrow failure | breast_cancer_primary | 5 | 0.12 (0.05-0.28) | 0.08 | Ineligible |
| T-DXd | Bronchitis | breast_cancer_primary | 5 | 0.29 (0.12-0.69) | 0.18 | Ineligible |
| T-DXd | Bronchitis | breast_cancer_primary | 5 | 0.29 (0.12-0.69) | 0.18 | Ineligible |
| T-DXd | Burning sensation | breast_cancer_primary | 5 | 0.23 (0.09-0.55) | 0.15 | Ineligible |
| T-DXd | Burning sensation | breast_cancer_primary | 5 | 0.23 (0.09-0.55) | 0.15 | Ineligible |
| T-DXd | Cardio-respiratory arrest | breast_cancer_primary | 5 | 0.64 (0.27-1.55) | 0.34 | Ineligible |
| T-DXd | Cardio-respiratory arrest | breast_cancer_primary | 5 | 0.64 (0.27-1.55) | 0.34 | Ineligible |
| T-DXd | Central nervous system lesion | breast_cancer_primary | 5 | 0.81 (0.33-1.96) | 0.41 | Ineligible |
| T-DXd | Central nervous system lesion | breast_cancer_primary | 5 | 0.81 (0.33-1.96) | 0.41 | Ineligible |
| T-DXd | Cholelithiasis | breast_cancer_primary | 5 | 0.48 (0.20-1.16) | 0.28 | Ineligible |
| T-DXd | Cholelithiasis | breast_cancer_primary | 5 | 0.48 (0.20-1.16) | 0.28 | Ineligible |
| T-DXd | Chronic obstructive pulmonary disease | breast_cancer_primary | 5 | 0.69 (0.29-1.68) | 0.36 | Ineligible |
| T-DXd | Chronic obstructive pulmonary disease | breast_cancer_primary | 5 | 0.69 (0.29-1.68) | 0.36 | Ineligible |
| T-DXd | Complication associated with device | breast_cancer_primary | 5 | 3.87 (1.55-9.68) | 1.03 | Ineligible |
| T-DXd | Complication associated with device | breast_cancer_primary | 5 | 3.87 (1.55-9.68) | 1.03 | Ineligible |
| T-DXd | Coronavirus infection | breast_cancer_primary | 5 | 1.72 (0.70-4.22) | 0.64 | Ineligible |
| T-DXd | Coronavirus infection | breast_cancer_primary | 5 | 1.72 (0.70-4.22) | 0.64 | Ineligible |
| T-DXd | Delirium | breast_cancer_primary | 5 | 0.92 (0.38-2.23) | 0.44 | Ineligible |
| T-DXd | Delirium | breast_cancer_primary | 5 | 0.92 (0.38-2.23) | 0.44 | Ineligible |
| T-DXd | Device defective | breast_cancer_primary | 5 | 34.19 (10.43-112.07) | 5.03 | Ineligible |
| T-DXd | Device defective | breast_cancer_primary | 5 | 34.19 (10.43-112.07) | 5.03 | Ineligible |
| T-DXd | Device occlusion | breast_cancer_primary | 5 | 1.59 (0.65-3.89) | 0.61 | Ineligible |
| T-DXd | Device occlusion | breast_cancer_primary | 5 | 1.59 (0.65-3.89) | 0.61 | Ineligible |
| T-DXd | Disorientation | breast_cancer_primary | 5 | 0.78 (0.32-1.90) | 0.40 | Ineligible |
| T-DXd | Disorientation | breast_cancer_primary | 5 | 0.78 (0.32-1.90) | 0.40 | Ineligible |
| T-DXd | Diverticulitis | breast_cancer_primary | 5 | 0.58 (0.24-1.40) | 0.32 | Ineligible |
| T-DXd | Diverticulitis | breast_cancer_primary | 5 | 0.58 (0.24-1.40) | 0.32 | Ineligible |
| T-DXd | Drug effect less than expected | breast_cancer_primary | 5 | 12.82 (4.70-35.01) | 3.01 | Ineligible |
| T-DXd | Drug effect less than expected | breast_cancer_primary | 5 | 12.82 (4.70-35.01) | 3.01 | Ineligible |
| T-DXd | Dysstasia | breast_cancer_primary | 5 | 0.46 (0.19-1.10) | 0.27 | Ineligible |
| T-DXd | Dysstasia | breast_cancer_primary | 5 | 0.46 (0.19-1.10) | 0.27 | Ineligible |
| T-DXd | Fluid intake reduced | breast_cancer_primary | 5 | 2.89 (1.17-7.16) | 0.86 | Ineligible |
| T-DXd | Fluid intake reduced | breast_cancer_primary | 5 | 2.89 (1.17-7.16) | 0.86 | Ineligible |
| T-DXd | Fluid retention | breast_cancer_primary | 5 | 0.40 (0.17-0.97) | 0.24 | Ineligible |
| T-DXd | Fluid retention | breast_cancer_primary | 5 | 0.40 (0.17-0.97) | 0.24 | Ineligible |
| T-DXd | Fracture | breast_cancer_primary | 5 | 0.56 (0.23-1.36) | 0.31 | Ineligible |
| T-DXd | Fracture | breast_cancer_primary | 5 | 0.56 (0.23-1.36) | 0.31 | Ineligible |
| T-DXd | Fungal infection | breast_cancer_primary | 5 | 0.72 (0.30-1.74) | 0.37 | Ineligible |
| T-DXd | Fungal infection | breast_cancer_primary | 5 | 0.72 (0.30-1.74) | 0.37 | Ineligible |
| T-DXd | Gastroenteritis | breast_cancer_primary | 5 | 0.81 (0.33-1.96) | 0.41 | Ineligible |
| T-DXd | Gastroenteritis | breast_cancer_primary | 5 | 0.81 (0.33-1.96) | 0.41 | Ineligible |
| T-DXd | Glomerular filtration rate decreased | breast_cancer_primary | 5 | 0.56 (0.23-1.36) | 0.31 | Ineligible |
| T-DXd | Glomerular filtration rate decreased | breast_cancer_primary | 5 | 0.56 (0.23-1.36) | 0.31 | Ineligible |
| T-DXd | Hepatic steatosis | breast_cancer_primary | 5 | 0.38 (0.16-0.92) | 0.23 | Ineligible |
| T-DXd | Hepatic steatosis | breast_cancer_primary | 5 | 0.38 (0.16-0.92) | 0.23 | Ineligible |
| T-DXd | Hunger | breast_cancer_primary | 5 | 1.71 (0.70-4.18) | 0.64 | Ineligible |
| T-DXd | Hunger | breast_cancer_primary | 5 | 1.71 (0.70-4.18) | 0.64 | Ineligible |
| T-DXd | Inappropriate antidiuretic hormone secretion | breast_cancer_primary | 5 | 1.97 (0.80-4.84) | 0.69 | Ineligible |
| T-DXd | Inappropriate antidiuretic hormone secretion | breast_cancer_primary | 5 | 1.97 (0.80-4.84) | 0.69 | Ineligible |
| T-DXd | Influenza like illness | breast_cancer_primary | 5 | 0.28 (0.12-0.69) | 0.18 | Ineligible |
| T-DXd | Influenza like illness | breast_cancer_primary | 5 | 0.28 (0.12-0.69) | 0.18 | Ineligible |
| T-DXd | Intracranial pressure increased | breast_cancer_primary | 5 | 2.33 (0.95-5.74) | 0.76 | Ineligible |
| T-DXd | Intracranial pressure increased | breast_cancer_primary | 5 | 2.33 (0.95-5.74) | 0.76 | Ineligible |
| T-DXd | Klebsiella infection | breast_cancer_primary | 5 | 3.36 (1.35-8.37) | 0.94 | Ineligible |
| T-DXd | Klebsiella infection | breast_cancer_primary | 5 | 3.36 (1.35-8.37) | 0.94 | Ineligible |
| T-DXd | Lower limb fracture | breast_cancer_primary | 5 | 1.00 (0.41-2.42) | 0.46 | Ineligible |
| T-DXd | Lower limb fracture | breast_cancer_primary | 5 | 1.00 (0.41-2.42) | 0.46 | Ineligible |
| T-DXd | Malignant pleural effusion | breast_cancer_primary | 5 | 0.84 (0.35-2.05) | 0.42 | Ineligible |
| T-DXd | Malignant pleural effusion | breast_cancer_primary | 5 | 0.84 (0.35-2.05) | 0.42 | Ineligible |
| T-DXd | Metastasis | breast_cancer_primary | 5 | 0.19 (0.08-0.47) | 0.13 | Ineligible |
| T-DXd | Metastasis | breast_cancer_primary | 5 | 0.19 (0.08-0.47) | 0.13 | Ineligible |
| T-DXd | Mitral valve replacement | breast_cancer_primary | 5 | 51.29 (13.77-191.04) | 5.57 | Ineligible |
| T-DXd | Mitral valve replacement | breast_cancer_primary | 5 | 51.29 (13.77-191.04) | 5.57 | Ineligible |
| T-DXd | Nasal obstruction | breast_cancer_primary | 5 | 11.40 (4.23-30.71) | 2.70 | Ineligible |
| T-DXd | Nasal obstruction | breast_cancer_primary | 5 | 11.40 (4.23-30.71) | 2.70 | Ineligible |
| T-DXd | Nephrolithiasis | breast_cancer_primary | 5 | 0.63 (0.26-1.52) | 0.34 | Ineligible |
| T-DXd | Nephrolithiasis | breast_cancer_primary | 5 | 0.63 (0.26-1.52) | 0.34 | Ineligible |
| T-DXd | Nervousness | breast_cancer_primary | 5 | 0.38 (0.16-0.93) | 0.23 | Ineligible |
| T-DXd | Nervousness | breast_cancer_primary | 5 | 0.38 (0.16-0.93) | 0.23 | Ineligible |
| T-DXd | Neurotoxicity | breast_cancer_primary | 5 | 0.40 (0.17-0.98) | 0.24 | Ineligible |
| T-DXd | Neurotoxicity | breast_cancer_primary | 5 | 0.40 (0.17-0.98) | 0.24 | Ineligible |
| T-DXd | Oesophageal varices haemorrhage | breast_cancer_primary | 5 | 4.56 (1.81-11.49) | 1.16 | Ineligible |
| T-DXd | Oesophageal varices haemorrhage | breast_cancer_primary | 5 | 4.56 (1.81-11.49) | 1.16 | Ineligible |
| T-DXd | Oncologic complication | breast_cancer_primary | 5 | 5.26 (2.07-13.35) | 1.29 | Ineligible |
| T-DXd | Oncologic complication | breast_cancer_primary | 5 | 5.26 (2.07-13.35) | 1.29 | Ineligible |
| T-DXd | Polyneuropathy | breast_cancer_primary | 5 | 0.20 (0.08-0.47) | 0.13 | Ineligible |
| T-DXd | Polyneuropathy | breast_cancer_primary | 5 | 0.20 (0.08-0.47) | 0.13 | Ineligible |
| T-DXd | Prescribed overdose | breast_cancer_primary | 5 | 5.54 (2.18-14.11) | 1.35 | Ineligible |
| T-DXd | Prescribed overdose | breast_cancer_primary | 5 | 5.54 (2.18-14.11) | 1.35 | Ineligible |
| T-DXd | Product use in unapproved indication | breast_cancer_primary | 5 | 0.14 (0.06-0.34) | 0.10 | Ineligible |
| T-DXd | Product use in unapproved indication | breast_cancer_primary | 5 | 0.14 (0.06-0.34) | 0.10 | Ineligible |
| T-DXd | Red cell distribution width increased | breast_cancer_primary | 5 | 0.51 (0.21-1.23) | 0.29 | Ineligible |
| T-DXd | Red cell distribution width increased | breast_cancer_primary | 5 | 0.51 (0.21-1.23) | 0.29 | Ineligible |
| T-DXd | Respiratory symptom | breast_cancer_primary | 5 | 7.07 (2.74-18.28) | 1.67 | Ineligible |
| T-DXd | Respiratory symptom | breast_cancer_primary | 5 | 7.07 (2.74-18.28) | 1.67 | Ineligible |
| T-DXd | Respiratory syncytial virus infection | breast_cancer_primary | 5 | 3.26 (1.31-8.10) | 0.92 | Ineligible |
| T-DXd | Respiratory syncytial virus infection | breast_cancer_primary | 5 | 3.26 (1.31-8.10) | 0.92 | Ineligible |
| T-DXd | Retching | breast_cancer_primary | 5 | 0.61 (0.25-1.47) | 0.33 | Ineligible |
| T-DXd | Retching | breast_cancer_primary | 5 | 0.61 (0.25-1.47) | 0.33 | Ineligible |
| T-DXd | Scab | breast_cancer_primary | 5 | 1.44 (0.59-3.52) | 0.58 | Ineligible |
| T-DXd | Scab | breast_cancer_primary | 5 | 1.44 (0.59-3.52) | 0.58 | Ineligible |
| T-DXd | Sinus tachycardia | breast_cancer_primary | 5 | 0.81 (0.33-1.96) | 0.40 | Ineligible |
| T-DXd | Sinus tachycardia | breast_cancer_primary | 5 | 0.81 (0.33-1.96) | 0.40 | Ineligible |
| T-DXd | Skin exfoliation | breast_cancer_primary | 5 | 0.13 (0.06-0.32) | 0.09 | Ineligible |
| T-DXd | Skin exfoliation | breast_cancer_primary | 5 | 0.13 (0.06-0.32) | 0.09 | Ineligible |
| T-DXd | Skin hyperpigmentation | breast_cancer_primary | 5 | 1.31 (0.54-3.18) | 0.55 | Ineligible |
| T-DXd | Skin hyperpigmentation | breast_cancer_primary | 5 | 1.31 (0.54-3.18) | 0.55 | Ineligible |
| T-DXd | Suicidal ideation | breast_cancer_primary | 5 | 0.71 (0.29-1.72) | 0.37 | Ineligible |
| T-DXd | Suicidal ideation | breast_cancer_primary | 5 | 0.71 (0.29-1.72) | 0.37 | Ineligible |
| T-DXd | Swelling face | breast_cancer_primary | 5 | 0.35 (0.14-0.84) | 0.21 | Ineligible |
| T-DXd | Swelling face | breast_cancer_primary | 5 | 0.35 (0.14-0.84) | 0.21 | Ineligible |
| T-DXd | Vascular device infection | breast_cancer_primary | 5 | 1.06 (0.43-2.57) | 0.48 | Ineligible |
| T-DXd | Vascular device infection | breast_cancer_primary | 5 | 1.06 (0.43-2.57) | 0.48 | Ineligible |
| T-DXd | Ankle fracture | breast_cancer_primary | 4 | 1.33 (0.49-3.61) | 0.51 | Ineligible |
| T-DXd | Ankle fracture | breast_cancer_primary | 4 | 1.33 (0.49-3.61) | 0.51 | Ineligible |
| T-DXd | Aortic arteriosclerosis | breast_cancer_primary | 4 | 1.62 (0.60-4.41) | 0.57 | Ineligible |
| T-DXd | Aortic arteriosclerosis | breast_cancer_primary | 4 | 1.62 (0.60-4.41) | 0.57 | Ineligible |
| T-DXd | Asphyxia | breast_cancer_primary | 4 | 1.09 (0.40-2.95) | 0.46 | Ineligible |
| T-DXd | Asphyxia | breast_cancer_primary | 4 | 1.09 (0.40-2.95) | 0.46 | Ineligible |
| T-DXd | Bartholin's cyst removal | breast_cancer_primary | 4 | 369.19 (19.87-6858.35) | 5.56 | Ineligible |
| T-DXd | Bartholin's cyst removal | breast_cancer_primary | 4 | 369.19 (19.87-6858.35) | 5.56 | Ineligible |
| T-DXd | Blindness unilateral | breast_cancer_primary | 4 | 1.98 (0.72-5.39) | 0.62 | Ineligible |
| T-DXd | Blindness unilateral | breast_cancer_primary | 4 | 1.98 (0.72-5.39) | 0.62 | Ineligible |
| T-DXd | Bradycardia | breast_cancer_primary | 4 | 0.63 (0.23-1.69) | 0.32 | Ineligible |
| T-DXd | Bradycardia | breast_cancer_primary | 4 | 0.63 (0.23-1.69) | 0.32 | Ineligible |
| T-DXd | Breast discomfort | breast_cancer_primary | 4 | 2.98 (1.08-8.23) | 0.77 | Ineligible |
| T-DXd | Breast discomfort | breast_cancer_primary | 4 | 2.98 (1.08-8.23) | 0.77 | Ineligible |
| T-DXd | Cachexia | breast_cancer_primary | 4 | 1.16 (0.43-3.12) | 0.47 | Ineligible |
| T-DXd | Cachexia | breast_cancer_primary | 4 | 1.16 (0.43-3.12) | 0.47 | Ineligible |
| T-DXd | Candida infection | breast_cancer_primary | 4 | 0.50 (0.19-1.33) | 0.27 | Ineligible |
| T-DXd | Candida infection | breast_cancer_primary | 4 | 0.50 (0.19-1.33) | 0.27 | Ineligible |
| T-DXd | Carcinoembryonic antigen increased | breast_cancer_primary | 4 | 0.46 (0.17-1.23) | 0.26 | Ineligible |
| T-DXd | Carcinoembryonic antigen increased | breast_cancer_primary | 4 | 0.46 (0.17-1.23) | 0.26 | Ineligible |
| T-DXd | Cardiac failure chronic | breast_cancer_primary | 4 | 1.84 (0.68-5.02) | 0.60 | Ineligible |
| T-DXd | Cardiac failure chronic | breast_cancer_primary | 4 | 1.84 (0.68-5.02) | 0.60 | Ineligible |
| T-DXd | Cerebral infarction | breast_cancer_primary | 4 | 0.61 (0.23-1.64) | 0.32 | Ineligible |
| T-DXd | Cerebral infarction | breast_cancer_primary | 4 | 0.61 (0.23-1.64) | 0.32 | Ineligible |
| T-DXd | Cerebrovascular accident | breast_cancer_primary | 4 | 0.13 (0.05-0.36) | 0.09 | Ineligible |
| T-DXd | Cerebrovascular accident | breast_cancer_primary | 4 | 0.13 (0.05-0.36) | 0.09 | Ineligible |
| T-DXd | Coronavirus pneumonia | breast_cancer_primary | 4 | 32.82 (8.81-122.25) | 3.89 | Ineligible |
| T-DXd | Coronavirus pneumonia | breast_cancer_primary | 4 | 32.82 (8.81-122.25) | 3.89 | Ineligible |
| T-DXd | Creatinine renal clearance increased | breast_cancer_primary | 4 | 5.66 (1.99-16.10) | 1.12 | Ineligible |
| T-DXd | Creatinine renal clearance increased | breast_cancer_primary | 4 | 5.66 (1.99-16.10) | 1.12 | Ineligible |
| T-DXd | Dermatitis bullous | breast_cancer_primary | 4 | 0.95 (0.35-2.57) | 0.42 | Ineligible |
| T-DXd | Dermatitis bullous | breast_cancer_primary | 4 | 0.95 (0.35-2.57) | 0.42 | Ineligible |
| T-DXd | Disturbance in attention | breast_cancer_primary | 4 | 0.35 (0.13-0.92) | 0.21 | Ineligible |
| T-DXd | Disturbance in attention | breast_cancer_primary | 4 | 0.35 (0.13-0.92) | 0.21 | Ineligible |
| T-DXd | Eastern Cooperative Oncology Group performance status worsened | breast_cancer_primary | 4 | 0.85 (0.31-2.28) | 0.39 | Ineligible |
| T-DXd | Eastern Cooperative Oncology Group performance status worsened | breast_cancer_primary | 4 | 0.85 (0.31-2.28) | 0.39 | Ineligible |
| T-DXd | Echocardiogram abnormal | breast_cancer_primary | 4 | 3.10 (1.12-8.56) | 0.78 | Ineligible |
| T-DXd | Echocardiogram abnormal | breast_cancer_primary | 4 | 3.10 (1.12-8.56) | 0.78 | Ineligible |
| T-DXd | Eczema | breast_cancer_primary | 4 | 0.42 (0.16-1.13) | 0.24 | Ineligible |
| T-DXd | Eczema | breast_cancer_primary | 4 | 0.42 (0.16-1.13) | 0.24 | Ineligible |
| T-DXd | Ejection fraction abnormal | breast_cancer_primary | 4 | 2.78 (1.01-7.66) | 0.74 | Ineligible |
| T-DXd | Ejection fraction abnormal | breast_cancer_primary | 4 | 2.78 (1.01-7.66) | 0.74 | Ineligible |
| T-DXd | Electrocardiogram QT prolonged | breast_cancer_primary | 4 | 0.14 (0.05-0.38) | 0.10 | Ineligible |
| T-DXd | Electrocardiogram QT prolonged | breast_cancer_primary | 4 | 0.14 (0.05-0.38) | 0.10 | Ineligible |
| T-DXd | Emergency care | breast_cancer_primary | 4 | 4.32 (1.54-12.10) | 0.94 | Ineligible |
| T-DXd | Emergency care | breast_cancer_primary | 4 | 4.32 (1.54-12.10) | 0.94 | Ineligible |
| T-DXd | Eructation | breast_cancer_primary | 4 | 0.86 (0.32-2.32) | 0.40 | Ineligible |
| T-DXd | Eructation | breast_cancer_primary | 4 | 0.86 (0.32-2.32) | 0.40 | Ineligible |
| T-DXd | Escherichia infection | breast_cancer_primary | 4 | 1.64 (0.60-4.46) | 0.57 | Ineligible |
| T-DXd | Escherichia infection | breast_cancer_primary | 4 | 1.64 (0.60-4.46) | 0.57 | Ineligible |
| T-DXd | Eyelid ptosis | breast_cancer_primary | 4 | 2.45 (0.89-6.72) | 0.69 | Ineligible |
| T-DXd | Eyelid ptosis | breast_cancer_primary | 4 | 2.45 (0.89-6.72) | 0.69 | Ineligible |
| T-DXd | Febrile bone marrow aplasia | breast_cancer_primary | 4 | 0.60 (0.23-1.62) | 0.31 | Ineligible |
| T-DXd | Febrile bone marrow aplasia | breast_cancer_primary | 4 | 0.60 (0.23-1.62) | 0.31 | Ineligible |
| T-DXd | Feeling hot | breast_cancer_primary | 4 | 0.19 (0.07-0.51) | 0.13 | Ineligible |
| T-DXd | Feeling hot | breast_cancer_primary | 4 | 0.19 (0.07-0.51) | 0.13 | Ineligible |
| T-DXd | Fluid replacement | breast_cancer_primary | 4 | 12.62 (4.11-38.72) | 2.08 | Ineligible |
| T-DXd | Fluid replacement | breast_cancer_primary | 4 | 12.62 (4.11-38.72) | 2.08 | Ineligible |
| T-DXd | Focal nodular hyperplasia | breast_cancer_primary | 4 | 7.46 (2.57-21.65) | 1.36 | Ineligible |
| T-DXd | Focal nodular hyperplasia | breast_cancer_primary | 4 | 7.46 (2.57-21.65) | 1.36 | Ineligible |
| T-DXd | Gastrointestinal infection | breast_cancer_primary | 4 | 1.44 (0.53-3.90) | 0.53 | Ineligible |
| T-DXd | Gastrointestinal infection | breast_cancer_primary | 4 | 1.44 (0.53-3.90) | 0.53 | Ineligible |
| T-DXd | Generalised tonic-clonic seizure | breast_cancer_primary | 4 | 1.91 (0.70-5.20) | 0.61 | Ineligible |
| T-DXd | Generalised tonic-clonic seizure | breast_cancer_primary | 4 | 1.91 (0.70-5.20) | 0.61 | Ineligible |
| T-DXd | Haematuria | breast_cancer_primary | 4 | 0.67 (0.25-1.80) | 0.34 | Ineligible |
| T-DXd | Haematuria | breast_cancer_primary | 4 | 0.67 (0.25-1.80) | 0.34 | Ineligible |
| T-DXd | Hemiparesis | breast_cancer_primary | 4 | 1.07 (0.40-2.89) | 0.45 | Ineligible |
| T-DXd | Hemiparesis | breast_cancer_primary | 4 | 1.07 (0.40-2.89) | 0.45 | Ineligible |
| T-DXd | Hot flush | breast_cancer_primary | 4 | 0.05 (0.02-0.13) | 0.03 | Ineligible |
| T-DXd | Hot flush | breast_cancer_primary | 4 | 0.05 (0.02-0.13) | 0.03 | Ineligible |
| T-DXd | Hydrocephalus | breast_cancer_primary | 4 | 1.76 (0.65-4.80) | 0.59 | Ineligible |
| T-DXd | Hydrocephalus | breast_cancer_primary | 4 | 1.76 (0.65-4.80) | 0.59 | Ineligible |
| T-DXd | Hypokinesia | breast_cancer_primary | 4 | 0.62 (0.23-1.66) | 0.32 | Ineligible |
| T-DXd | Hypokinesia | breast_cancer_primary | 4 | 0.62 (0.23-1.66) | 0.32 | Ineligible |
| T-DXd | Hypothyroidism | breast_cancer_primary | 4 | 0.22 (0.08-0.60) | 0.14 | Ineligible |
| T-DXd | Hypothyroidism | breast_cancer_primary | 4 | 0.22 (0.08-0.60) | 0.14 | Ineligible |
| T-DXd | Hypovolaemia | breast_cancer_primary | 4 | 1.73 (0.63-4.70) | 0.58 | Ineligible |
| T-DXd | Hypovolaemia | breast_cancer_primary | 4 | 1.73 (0.63-4.70) | 0.58 | Ineligible |
| T-DXd | Incorrect product administration duration | breast_cancer_primary | 4 | 4.56 (1.62-12.81) | 0.97 | Ineligible |
| T-DXd | Incorrect product administration duration | breast_cancer_primary | 4 | 4.56 (1.62-12.81) | 0.97 | Ineligible |
| T-DXd | Intestinal perforation | breast_cancer_primary | 4 | 1.09 (0.40-2.93) | 0.46 | Ineligible |
| T-DXd | Intestinal perforation | breast_cancer_primary | 4 | 1.09 (0.40-2.93) | 0.46 | Ineligible |
| T-DXd | Left ventricular dysfunction | breast_cancer_primary | 4 | 0.29 (0.11-0.78) | 0.18 | Ineligible |
| T-DXd | Left ventricular dysfunction | breast_cancer_primary | 4 | 0.29 (0.11-0.78) | 0.18 | Ineligible |
| T-DXd | Leukocytosis | breast_cancer_primary | 4 | 0.61 (0.23-1.64) | 0.32 | Ineligible |
| T-DXd | Leukocytosis | breast_cancer_primary | 4 | 0.61 (0.23-1.64) | 0.32 | Ineligible |
| T-DXd | Lip swelling | breast_cancer_primary | 4 | 0.56 (0.21-1.49) | 0.30 | Ineligible |
| T-DXd | Lip swelling | breast_cancer_primary | 4 | 0.56 (0.21-1.49) | 0.30 | Ineligible |
| T-DXd | Localised oedema | breast_cancer_primary | 4 | 2.38 (0.87-6.52) | 0.68 | Ineligible |
| T-DXd | Localised oedema | breast_cancer_primary | 4 | 2.38 (0.87-6.52) | 0.68 | Ineligible |
| T-DXd | Lymphadenopathy | breast_cancer_primary | 4 | 0.16 (0.06-0.43) | 0.11 | Ineligible |
| T-DXd | Lymphadenopathy | breast_cancer_primary | 4 | 0.16 (0.06-0.43) | 0.11 | Ineligible |
| T-DXd | Madarosis | breast_cancer_primary | 4 | 0.03 (0.01-0.08) | 0.02 | Ineligible |
| T-DXd | Madarosis | breast_cancer_primary | 4 | 0.03 (0.01-0.08) | 0.02 | Ineligible |
| T-DXd | Mass | breast_cancer_primary | 4 | 0.49 (0.18-1.32) | 0.27 | Ineligible |
| T-DXd | Mass | breast_cancer_primary | 4 | 0.49 (0.18-1.32) | 0.27 | Ineligible |
| T-DXd | Mitral valve incompetence | breast_cancer_primary | 4 | 0.45 (0.17-1.21) | 0.26 | Ineligible |
| T-DXd | Mitral valve incompetence | breast_cancer_primary | 4 | 0.45 (0.17-1.21) | 0.26 | Ineligible |
| T-DXd | Nail disorder | breast_cancer_primary | 4 | 0.20 (0.07-0.52) | 0.13 | Ineligible |
| T-DXd | Nail disorder | breast_cancer_primary | 4 | 0.20 (0.07-0.52) | 0.13 | Ineligible |
| T-DXd | Neuralgia | breast_cancer_primary | 4 | 0.35 (0.13-0.93) | 0.21 | Ineligible |
| T-DXd | Neuralgia | breast_cancer_primary | 4 | 0.35 (0.13-0.93) | 0.21 | Ineligible |
| T-DXd | Onychoclasis | breast_cancer_primary | 4 | 0.27 (0.10-0.72) | 0.17 | Ineligible |
| T-DXd | Onychoclasis | breast_cancer_primary | 4 | 0.27 (0.10-0.72) | 0.17 | Ineligible |
| T-DXd | Overdose | breast_cancer_primary | 4 | 0.37 (0.14-1.00) | 0.22 | Ineligible |
| T-DXd | Overdose | breast_cancer_primary | 4 | 0.37 (0.14-1.00) | 0.22 | Ineligible |
| T-DXd | Palliative care | breast_cancer_primary | 4 | 13.67 (4.41-42.41) | 2.23 | Ineligible |
| T-DXd | Palliative care | breast_cancer_primary | 4 | 13.67 (4.41-42.41) | 2.23 | Ineligible |
| T-DXd | Paranoia | breast_cancer_primary | 4 | 2.73 (0.99-7.53) | 0.73 | Ineligible |
| T-DXd | Paranoia | breast_cancer_primary | 4 | 2.73 (0.99-7.53) | 0.73 | Ineligible |
| T-DXd | Pneumomediastinum | breast_cancer_primary | 4 | 6.08 (2.13-17.37) | 1.17 | Ineligible |
| T-DXd | Pneumomediastinum | breast_cancer_primary | 4 | 6.08 (2.13-17.37) | 1.17 | Ineligible |
| T-DXd | Pneumonia aspiration | breast_cancer_primary | 4 | 1.37 (0.50-3.70) | 0.52 | Ineligible |
| T-DXd | Pneumonia aspiration | breast_cancer_primary | 4 | 1.37 (0.50-3.70) | 0.52 | Ineligible |
| T-DXd | Pneumonitis chemical | breast_cancer_primary | 4 | 5.13 (1.81-14.50) | 1.05 | Ineligible |
| T-DXd | Pneumonitis chemical | breast_cancer_primary | 4 | 5.13 (1.81-14.50) | 1.05 | Ineligible |
| T-DXd | Presyncope | breast_cancer_primary | 4 | 0.67 (0.25-1.79) | 0.34 | Ineligible |
| T-DXd | Presyncope | breast_cancer_primary | 4 | 0.67 (0.25-1.79) | 0.34 | Ineligible |
| T-DXd | Pulmonary alveolar haemorrhage | breast_cancer_primary | 4 | 2.56 (0.93-7.04) | 0.71 | Ineligible |
| T-DXd | Pulmonary alveolar haemorrhage | breast_cancer_primary | 4 | 2.56 (0.93-7.04) | 0.71 | Ineligible |
| T-DXd | Rash erythematous | breast_cancer_primary | 4 | 0.32 (0.12-0.87) | 0.20 | Ineligible |
| T-DXd | Rash erythematous | breast_cancer_primary | 4 | 0.32 (0.12-0.87) | 0.20 | Ineligible |
| T-DXd | Rectal haemorrhage | breast_cancer_primary | 4 | 0.36 (0.13-0.96) | 0.21 | Ineligible |
| T-DXd | Rectal haemorrhage | breast_cancer_primary | 4 | 0.36 (0.13-0.96) | 0.21 | Ineligible |
| T-DXd | Renal disorder | breast_cancer_primary | 4 | 0.28 (0.10-0.74) | 0.17 | Ineligible |
| T-DXd | Renal disorder | breast_cancer_primary | 4 | 0.28 (0.10-0.74) | 0.17 | Ineligible |
| T-DXd | SARS-CoV-2 test positive | breast_cancer_primary | 4 | 0.74 (0.27-1.98) | 0.36 | Ineligible |
| T-DXd | SARS-CoV-2 test positive | breast_cancer_primary | 4 | 0.74 (0.27-1.98) | 0.36 | Ineligible |
| T-DXd | Skin abrasion | breast_cancer_primary | 4 | 2.22 (0.81-6.07) | 0.66 | Ineligible |
| T-DXd | Skin abrasion | breast_cancer_primary | 4 | 2.22 (0.81-6.07) | 0.66 | Ineligible |
| T-DXd | Staphylococcal bacteraemia | breast_cancer_primary | 4 | 2.60 (0.95-7.16) | 0.72 | Ineligible |
| T-DXd | Staphylococcal bacteraemia | breast_cancer_primary | 4 | 2.60 (0.95-7.16) | 0.72 | Ineligible |
| T-DXd | Starvation | breast_cancer_primary | 4 | 18.23 (5.61-59.22) | 2.83 | Ineligible |
| T-DXd | Starvation | breast_cancer_primary | 4 | 18.23 (5.61-59.22) | 2.83 | Ineligible |
| T-DXd | Stress | breast_cancer_primary | 4 | 0.20 (0.08-0.54) | 0.13 | Ineligible |
| T-DXd | Stress | breast_cancer_primary | 4 | 0.20 (0.08-0.54) | 0.13 | Ineligible |
| T-DXd | Surgery | breast_cancer_primary | 4 | 0.38 (0.14-1.03) | 0.23 | Ineligible |
| T-DXd | Surgery | breast_cancer_primary | 4 | 0.38 (0.14-1.03) | 0.23 | Ineligible |
| T-DXd | Toxic skin eruption | breast_cancer_primary | 4 | 0.72 (0.27-1.93) | 0.35 | Ineligible |
| T-DXd | Toxic skin eruption | breast_cancer_primary | 4 | 0.72 (0.27-1.93) | 0.35 | Ineligible |
| T-DXd | Type 2 diabetes mellitus | breast_cancer_primary | 4 | 0.67 (0.25-1.80) | 0.34 | Ineligible |
| T-DXd | Type 2 diabetes mellitus | breast_cancer_primary | 4 | 0.67 (0.25-1.80) | 0.34 | Ineligible |
| T-DXd | Urinary tract stoma complication | breast_cancer_primary | 4 | 82.05 (15.03-448.05) | 4.82 | Ineligible |
| T-DXd | Urinary tract stoma complication | breast_cancer_primary | 4 | 82.05 (15.03-448.05) | 4.82 | Ineligible |
| T-DXd | Varices oesophageal | breast_cancer_primary | 4 | 2.16 (0.79-5.90) | 0.65 | Ineligible |
| T-DXd | Varices oesophageal | breast_cancer_primary | 4 | 2.16 (0.79-5.90) | 0.65 | Ineligible |
| T-DXd | Ventricular dysfunction | breast_cancer_primary | 4 | 0.99 (0.37-2.66) | 0.43 | Ineligible |
| T-DXd | Ventricular dysfunction | breast_cancer_primary | 4 | 0.99 (0.37-2.66) | 0.43 | Ineligible |
| T-DXd | Vocal cord paralysis | breast_cancer_primary | 4 | 1.69 (0.62-4.60) | 0.58 | Ineligible |
| T-DXd | Vocal cord paralysis | breast_cancer_primary | 4 | 1.69 (0.62-4.60) | 0.58 | Ineligible |
| T-DXd | Acute interstitial pneumonitis | breast_cancer_primary | 3 | 2.12 (0.66-6.77) | 0.56 | Ineligible |
| T-DXd | Acute interstitial pneumonitis | breast_cancer_primary | 3 | 2.12 (0.66-6.77) | 0.56 | Ineligible |
| T-DXd | Acute myocardial infarction | breast_cancer_primary | 3 | 0.57 (0.18-1.77) | 0.28 | Ineligible |
| T-DXd | Acute myocardial infarction | breast_cancer_primary | 3 | 0.57 (0.18-1.77) | 0.28 | Ineligible |
| T-DXd | Administration site bruise | breast_cancer_primary | 3 | 123.05 (12.80-1183.20) | 3.07 | Ineligible |
| T-DXd | Administration site bruise | breast_cancer_primary | 3 | 123.05 (12.80-1183.20) | 3.07 | Ineligible |
| T-DXd | Anaphylactic reaction | breast_cancer_primary | 3 | 0.22 (0.07-0.69) | 0.14 | Ineligible |
| T-DXd | Anaphylactic reaction | breast_cancer_primary | 3 | 0.22 (0.07-0.69) | 0.14 | Ineligible |
| T-DXd | Anger | breast_cancer_primary | 3 | 0.85 (0.27-2.68) | 0.36 | Ineligible |
| T-DXd | Anger | breast_cancer_primary | 3 | 0.85 (0.27-2.68) | 0.36 | Ineligible |
| T-DXd | Angina pectoris | breast_cancer_primary | 3 | 0.45 (0.14-1.41) | 0.24 | Ineligible |
| T-DXd | Angina pectoris | breast_cancer_primary | 3 | 0.45 (0.14-1.41) | 0.24 | Ineligible |
| T-DXd | Aplasia | breast_cancer_primary | 3 | 2.20 (0.69-7.02) | 0.57 | Ineligible |
| T-DXd | Aplasia | breast_cancer_primary | 3 | 2.20 (0.69-7.02) | 0.57 | Ineligible |
| T-DXd | Arthritis bacterial | breast_cancer_primary | 3 | 6.48 (1.92-21.89) | 0.93 | Ineligible |
| T-DXd | Arthritis bacterial | breast_cancer_primary | 3 | 6.48 (1.92-21.89) | 0.93 | Ineligible |
| T-DXd | Arthropathy | breast_cancer_primary | 3 | 0.25 (0.08-0.77) | 0.16 | Ineligible |
| T-DXd | Arthropathy | breast_cancer_primary | 3 | 0.25 (0.08-0.77) | 0.16 | Ineligible |
| T-DXd | Aspiration | breast_cancer_primary | 3 | 1.89 (0.59-6.02) | 0.53 | Ineligible |
| T-DXd | Aspiration | breast_cancer_primary | 3 | 1.89 (0.59-6.02) | 0.53 | Ineligible |
| T-DXd | Blood chloride increased | breast_cancer_primary | 3 | 2.56 (0.80-8.23) | 0.61 | Ineligible |
| T-DXd | Blood chloride increased | breast_cancer_primary | 3 | 2.56 (0.80-8.23) | 0.61 | Ineligible |
| T-DXd | Blood lactic acid abnormal | breast_cancer_primary | 3 | 123.05 (12.80-1183.20) | 3.07 | Ineligible |
| T-DXd | Blood lactic acid abnormal | breast_cancer_primary | 3 | 123.05 (12.80-1183.20) | 3.07 | Ineligible |
| T-DXd | Blood lactic acid increased | breast_cancer_primary | 3 | 6.15 (1.83-20.71) | 0.91 | Ineligible |
| T-DXd | Blood lactic acid increased | breast_cancer_primary | 3 | 6.15 (1.83-20.71) | 0.91 | Ineligible |
| T-DXd | Brain injury | breast_cancer_primary | 3 | 1.66 (0.52-5.27) | 0.50 | Ineligible |
| T-DXd | Brain injury | breast_cancer_primary | 3 | 1.66 (0.52-5.27) | 0.50 | Ineligible |
| T-DXd | Bronchopulmonary aspergillosis | breast_cancer_primary | 3 | 3.42 (1.05-11.10) | 0.69 | Ineligible |
| T-DXd | Bronchopulmonary aspergillosis | breast_cancer_primary | 3 | 3.42 (1.05-11.10) | 0.69 | Ineligible |
| T-DXd | Cardiogenic shock | breast_cancer_primary | 3 | 1.00 (0.32-3.14) | 0.40 | Ineligible |
| T-DXd | Cardiogenic shock | breast_cancer_primary | 3 | 1.00 (0.32-3.14) | 0.40 | Ineligible |
| T-DXd | Cardiomegaly | breast_cancer_primary | 3 | 0.59 (0.19-1.86) | 0.29 | Ineligible |
| T-DXd | Cardiomegaly | breast_cancer_primary | 3 | 0.59 (0.19-1.86) | 0.29 | Ineligible |
| T-DXd | Catheter site infection | breast_cancer_primary | 3 | 1.58 (0.50-5.00) | 0.49 | Ineligible |
| T-DXd | Catheter site infection | breast_cancer_primary | 3 | 1.58 (0.50-5.00) | 0.49 | Ineligible |
| T-DXd | Colitis ischaemic | breast_cancer_primary | 3 | 0.97 (0.31-3.04) | 0.39 | Ineligible |
| T-DXd | Colitis ischaemic | breast_cancer_primary | 3 | 0.97 (0.31-3.04) | 0.39 | Ineligible |
| T-DXd | Colorectal cancer stage IV | breast_cancer_primary | 3 | 30.76 (6.88-137.48) | 2.06 | Ineligible |
| T-DXd | Colorectal cancer stage IV | breast_cancer_primary | 3 | 30.76 (6.88-137.48) | 2.06 | Ineligible |
| T-DXd | Conjunctivitis | breast_cancer_primary | 3 | 0.46 (0.15-1.43) | 0.25 | Ineligible |
| T-DXd | Conjunctivitis | breast_cancer_primary | 3 | 0.46 (0.15-1.43) | 0.25 | Ineligible |
| T-DXd | Crying | breast_cancer_primary | 3 | 0.38 (0.12-1.20) | 0.22 | Ineligible |
| T-DXd | Crying | breast_cancer_primary | 3 | 0.38 (0.12-1.20) | 0.22 | Ineligible |
| T-DXd | Cyst | breast_cancer_primary | 3 | 1.01 (0.32-3.17) | 0.40 | Ineligible |
| T-DXd | Cyst | breast_cancer_primary | 3 | 1.01 (0.32-3.17) | 0.40 | Ineligible |
| T-DXd | Deafness | breast_cancer_primary | 3 | 0.35 (0.11-1.08) | 0.20 | Ineligible |
| T-DXd | Deafness | breast_cancer_primary | 3 | 0.35 (0.11-1.08) | 0.20 | Ineligible |
| T-DXd | Dental caries | breast_cancer_primary | 3 | 0.62 (0.20-1.94) | 0.30 | Ineligible |
| T-DXd | Dental caries | breast_cancer_primary | 3 | 0.62 (0.20-1.94) | 0.30 | Ineligible |
| T-DXd | Dermatitis | breast_cancer_primary | 3 | 0.29 (0.09-0.91) | 0.18 | Ineligible |
| T-DXd | Dermatitis | breast_cancer_primary | 3 | 0.29 (0.09-0.91) | 0.18 | Ineligible |
| T-DXd | Device malfunction | breast_cancer_primary | 3 | 0.76 (0.24-2.38) | 0.34 | Ineligible |
| T-DXd | Device malfunction | breast_cancer_primary | 3 | 0.76 (0.24-2.38) | 0.34 | Ineligible |
| T-DXd | Diabetes mellitus inadequate control | breast_cancer_primary | 3 | 1.23 (0.39-3.88) | 0.44 | Ineligible |
| T-DXd | Diabetes mellitus inadequate control | breast_cancer_primary | 3 | 1.23 (0.39-3.88) | 0.44 | Ineligible |
| T-DXd | Diabetic metabolic decompensation | breast_cancer_primary | 3 | 5.13 (1.54-17.03) | 0.83 | Ineligible |
| T-DXd | Diabetic metabolic decompensation | breast_cancer_primary | 3 | 5.13 (1.54-17.03) | 0.83 | Ineligible |
| T-DXd | Diverticular perforation | breast_cancer_primary | 3 | 6.15 (1.83-20.71) | 0.91 | Ineligible |
| T-DXd | Diverticular perforation | breast_cancer_primary | 3 | 6.15 (1.83-20.71) | 0.91 | Ineligible |
| T-DXd | Drug interaction | breast_cancer_primary | 3 | 0.09 (0.03-0.29) | 0.07 | Ineligible |
| T-DXd | Drug interaction | breast_cancer_primary | 3 | 0.09 (0.03-0.29) | 0.07 | Ineligible |
| T-DXd | Drug resistance | breast_cancer_primary | 3 | 0.30 (0.10-0.94) | 0.18 | Ineligible |
| T-DXd | Drug resistance | breast_cancer_primary | 3 | 0.30 (0.10-0.94) | 0.18 | Ineligible |
| T-DXd | Duodenal ulcer | breast_cancer_primary | 3 | 1.04 (0.33-3.28) | 0.40 | Ineligible |
| T-DXd | Duodenal ulcer | breast_cancer_primary | 3 | 1.04 (0.33-3.28) | 0.40 | Ineligible |
| T-DXd | Dysuria | breast_cancer_primary | 3 | 0.20 (0.06-0.63) | 0.13 | Ineligible |
| T-DXd | Dysuria | breast_cancer_primary | 3 | 0.20 (0.06-0.63) | 0.13 | Ineligible |
| T-DXd | Ecchymosis | breast_cancer_primary | 3 | 1.52 (0.48-4.81) | 0.48 | Ineligible |
| T-DXd | Ecchymosis | breast_cancer_primary | 3 | 1.52 (0.48-4.81) | 0.48 | Ineligible |
| T-DXd | Embolism | breast_cancer_primary | 3 | 0.57 (0.18-1.78) | 0.28 | Ineligible |
| T-DXd | Embolism | breast_cancer_primary | 3 | 0.57 (0.18-1.78) | 0.28 | Ineligible |
| T-DXd | Emotional disorder | breast_cancer_primary | 3 | 0.33 (0.11-1.04) | 0.20 | Ineligible |
| T-DXd | Emotional disorder | breast_cancer_primary | 3 | 0.33 (0.11-1.04) | 0.20 | Ineligible |
| T-DXd | Encephalitis | breast_cancer_primary | 3 | 1.41 (0.45-4.47) | 0.47 | Ineligible |
| T-DXd | Encephalitis | breast_cancer_primary | 3 | 1.41 (0.45-4.47) | 0.47 | Ineligible |
| T-DXd | Encephalopathy | breast_cancer_primary | 3 | 0.71 (0.23-2.23) | 0.33 | Ineligible |
| T-DXd | Encephalopathy | breast_cancer_primary | 3 | 0.71 (0.23-2.23) | 0.33 | Ineligible |
| T-DXd | Erysipelas | breast_cancer_primary | 3 | 0.47 (0.15-1.48) | 0.25 | Ineligible |
| T-DXd | Erysipelas | breast_cancer_primary | 3 | 0.47 (0.15-1.48) | 0.25 | Ineligible |
| T-DXd | Extravasation | breast_cancer_primary | 3 | 1.27 (0.40-4.00) | 0.45 | Ineligible |
| T-DXd | Extravasation | breast_cancer_primary | 3 | 1.27 (0.40-4.00) | 0.45 | Ineligible |
| T-DXd | Face oedema | breast_cancer_primary | 3 | 0.50 (0.16-1.55) | 0.26 | Ineligible |
| T-DXd | Face oedema | breast_cancer_primary | 3 | 0.50 (0.16-1.55) | 0.26 | Ineligible |
| T-DXd | Facial discomfort | breast_cancer_primary | 3 | 8.20 (2.37-28.34) | 1.05 | Ineligible |
| T-DXd | Facial discomfort | breast_cancer_primary | 3 | 8.20 (2.37-28.34) | 1.05 | Ineligible |
| T-DXd | Facial paralysis | breast_cancer_primary | 3 | 0.92 (0.29-2.90) | 0.38 | Ineligible |
| T-DXd | Facial paralysis | breast_cancer_primary | 3 | 0.92 (0.29-2.90) | 0.38 | Ineligible |
| T-DXd | Feeling jittery | breast_cancer_primary | 3 | 1.84 (0.58-5.84) | 0.53 | Ineligible |
| T-DXd | Feeling jittery | breast_cancer_primary | 3 | 1.84 (0.58-5.84) | 0.53 | Ineligible |
| T-DXd | Fluid imbalance | breast_cancer_primary | 3 | 20.51 (5.13-82.02) | 1.70 | Ineligible |
| T-DXd | Fluid imbalance | breast_cancer_primary | 3 | 20.51 (5.13-82.02) | 1.70 | Ineligible |
| T-DXd | Gastric dilatation | breast_cancer_primary | 3 | 5.86 (1.75-19.65) | 0.89 | Ineligible |
| T-DXd | Gastric dilatation | breast_cancer_primary | 3 | 5.86 (1.75-19.65) | 0.89 | Ineligible |
| T-DXd | Gastrointestinal oedema | breast_cancer_primary | 3 | 4.10 (1.25-13.44) | 0.75 | Ineligible |
| T-DXd | Gastrointestinal oedema | breast_cancer_primary | 3 | 4.10 (1.25-13.44) | 0.75 | Ineligible |
| T-DXd | Haemolysis | breast_cancer_primary | 3 | 1.66 (0.52-5.27) | 0.50 | Ineligible |
| T-DXd | Haemolysis | breast_cancer_primary | 3 | 1.66 (0.52-5.27) | 0.50 | Ineligible |
| T-DXd | Haemorrhoids | breast_cancer_primary | 3 | 0.29 (0.09-0.90) | 0.17 | Ineligible |
| T-DXd | Haemorrhoids | breast_cancer_primary | 3 | 0.29 (0.09-0.90) | 0.17 | Ineligible |
| T-DXd | Head and neck cancer | breast_cancer_primary | 3 | 24.61 (5.88-103.00) | 1.86 | Ineligible |
| T-DXd | Head and neck cancer | breast_cancer_primary | 3 | 24.61 (5.88-103.00) | 1.86 | Ineligible |
| T-DXd | Heart failure with reduced ejection fraction | breast_cancer_primary | 3 | 4.24 (1.29-13.93) | 0.76 | Ineligible |
| T-DXd | Heart failure with reduced ejection fraction | breast_cancer_primary | 3 | 4.24 (1.29-13.93) | 0.76 | Ineligible |
| T-DXd | Heart rate decreased | breast_cancer_primary | 3 | 0.52 (0.17-1.61) | 0.27 | Ineligible |
| T-DXd | Heart rate decreased | breast_cancer_primary | 3 | 0.52 (0.17-1.61) | 0.27 | Ineligible |
| T-DXd | Heart rate irregular | breast_cancer_primary | 3 | 0.68 (0.22-2.13) | 0.32 | Ineligible |
| T-DXd | Heart rate irregular | breast_cancer_primary | 3 | 0.68 (0.22-2.13) | 0.32 | Ineligible |
| T-DXd | Hepatic fibrosis | breast_cancer_primary | 3 | 3.33 (1.03-10.79) | 0.68 | Ineligible |
| T-DXd | Hepatic fibrosis | breast_cancer_primary | 3 | 3.33 (1.03-10.79) | 0.68 | Ineligible |
| T-DXd | Hepatocellular injury | breast_cancer_primary | 3 | 0.29 (0.09-0.89) | 0.17 | Ineligible |
| T-DXd | Hepatocellular injury | breast_cancer_primary | 3 | 0.29 (0.09-0.89) | 0.17 | Ineligible |
| T-DXd | Hip arthroplasty | breast_cancer_primary | 3 | 1.17 (0.37-3.69) | 0.43 | Ineligible |
| T-DXd | Hip arthroplasty | breast_cancer_primary | 3 | 1.17 (0.37-3.69) | 0.43 | Ineligible |
| T-DXd | Hydronephrosis | breast_cancer_primary | 3 | 0.51 (0.16-1.59) | 0.27 | Ineligible |
| T-DXd | Hydronephrosis | breast_cancer_primary | 3 | 0.51 (0.16-1.59) | 0.27 | Ineligible |
| T-DXd | Hyperhidrosis | breast_cancer_primary | 3 | 0.10 (0.03-0.32) | 0.07 | Ineligible |
| T-DXd | Hyperhidrosis | breast_cancer_primary | 3 | 0.10 (0.03-0.32) | 0.07 | Ineligible |
| T-DXd | Hypervolaemia | breast_cancer_primary | 3 | 4.10 (1.25-13.44) | 0.75 | Ineligible |
| T-DXd | Hypervolaemia | breast_cancer_primary | 3 | 4.10 (1.25-13.44) | 0.75 | Ineligible |
| T-DXd | Hypoacusis | breast_cancer_primary | 3 | 0.17 (0.05-0.53) | 0.11 | Ineligible |
| T-DXd | Hypoacusis | breast_cancer_primary | 3 | 0.17 (0.05-0.53) | 0.11 | Ineligible |
| T-DXd | Ill-defined disorder | breast_cancer_primary | 3 | 0.16 (0.05-0.50) | 0.11 | Ineligible |
| T-DXd | Ill-defined disorder | breast_cancer_primary | 3 | 0.16 (0.05-0.50) | 0.11 | Ineligible |
| T-DXd | Impaired driving ability | breast_cancer_primary | 3 | 2.62 (0.81-8.41) | 0.61 | Ineligible |
| T-DXd | Impaired driving ability | breast_cancer_primary | 3 | 2.62 (0.81-8.41) | 0.61 | Ineligible |
| T-DXd | Impaired gastric emptying | breast_cancer_primary | 3 | 1.84 (0.58-5.84) | 0.53 | Ineligible |
| T-DXd | Impaired gastric emptying | breast_cancer_primary | 3 | 1.84 (0.58-5.84) | 0.53 | Ineligible |
| T-DXd | Incontinence | breast_cancer_primary | 3 | 0.87 (0.28-2.74) | 0.37 | Ineligible |
| T-DXd | Incontinence | breast_cancer_primary | 3 | 0.87 (0.28-2.74) | 0.37 | Ineligible |
| T-DXd | Incorrect drug administration rate | breast_cancer_primary | 3 | 5.13 (1.54-17.03) | 0.83 | Ineligible |
| T-DXd | Incorrect drug administration rate | breast_cancer_primary | 3 | 5.13 (1.54-17.03) | 0.83 | Ineligible |
| T-DXd | Intentional underdose | breast_cancer_primary | 3 | 4.24 (1.29-13.93) | 0.76 | Ineligible |
| T-DXd | Intentional underdose | breast_cancer_primary | 3 | 4.24 (1.29-13.93) | 0.76 | Ineligible |
| T-DXd | Interstitial lung abnormality | breast_cancer_primary | 3 | 12.31 (3.39-44.72) | 1.31 | Ineligible |
| T-DXd | Interstitial lung abnormality | breast_cancer_primary | 3 | 12.31 (3.39-44.72) | 1.31 | Ineligible |
| T-DXd | Intracardiac thrombus | breast_cancer_primary | 3 | 2.56 (0.80-8.23) | 0.61 | Ineligible |
| T-DXd | Intracardiac thrombus | breast_cancer_primary | 3 | 2.56 (0.80-8.23) | 0.61 | Ineligible |
| T-DXd | Jaundice cholestatic | breast_cancer_primary | 3 | 1.52 (0.48-4.81) | 0.48 | Ineligible |
| T-DXd | Jaundice cholestatic | breast_cancer_primary | 3 | 1.52 (0.48-4.81) | 0.48 | Ineligible |
| T-DXd | Jugular vein thrombosis | breast_cancer_primary | 3 | 1.24 (0.39-3.92) | 0.44 | Ineligible |
| T-DXd | Jugular vein thrombosis | breast_cancer_primary | 3 | 1.24 (0.39-3.92) | 0.44 | Ineligible |
| T-DXd | Klebsiella test positive | breast_cancer_primary | 3 | 11.19 (3.12-40.11) | 1.24 | Ineligible |
| T-DXd | Klebsiella test positive | breast_cancer_primary | 3 | 11.19 (3.12-40.11) | 1.24 | Ineligible |
| T-DXd | Language disorder | breast_cancer_primary | 3 | 3.97 (1.21-12.99) | 0.74 | Ineligible |
| T-DXd | Language disorder | breast_cancer_primary | 3 | 3.97 (1.21-12.99) | 0.74 | Ineligible |
| T-DXd | Leukoencephalopathy | breast_cancer_primary | 3 | 1.66 (0.52-5.27) | 0.50 | Ineligible |
| T-DXd | Leukoencephalopathy | breast_cancer_primary | 3 | 1.66 (0.52-5.27) | 0.50 | Ineligible |
| T-DXd | Lung consolidation | breast_cancer_primary | 3 | 2.05 (0.64-6.54) | 0.55 | Ineligible |
| T-DXd | Lung consolidation | breast_cancer_primary | 3 | 2.05 (0.64-6.54) | 0.55 | Ineligible |
| T-DXd | Maculopathy | breast_cancer_primary | 3 | 1.73 (0.55-5.50) | 0.51 | Ineligible |
| T-DXd | Maculopathy | breast_cancer_primary | 3 | 1.73 (0.55-5.50) | 0.51 | Ineligible |
| T-DXd | Malnutrition | breast_cancer_primary | 3 | 0.64 (0.20-1.99) | 0.31 | Ineligible |
| T-DXd | Malnutrition | breast_cancer_primary | 3 | 0.64 (0.20-1.99) | 0.31 | Ineligible |
| T-DXd | Mental fatigue | breast_cancer_primary | 3 | 1.92 (0.60-6.12) | 0.54 | Ineligible |
| T-DXd | Mental fatigue | breast_cancer_primary | 3 | 1.92 (0.60-6.12) | 0.54 | Ineligible |
| T-DXd | Metastases to pleura | breast_cancer_primary | 3 | 0.39 (0.13-1.22) | 0.22 | Ineligible |
| T-DXd | Metastases to pleura | breast_cancer_primary | 3 | 0.39 (0.13-1.22) | 0.22 | Ineligible |
| T-DXd | Motor dysfunction | breast_cancer_primary | 3 | 1.12 (0.36-3.52) | 0.42 | Ineligible |
| T-DXd | Motor dysfunction | breast_cancer_primary | 3 | 1.12 (0.36-3.52) | 0.42 | Ineligible |
| T-DXd | Musculoskeletal discomfort | breast_cancer_primary | 3 | 0.37 (0.12-1.15) | 0.21 | Ineligible |
| T-DXd | Musculoskeletal discomfort | breast_cancer_primary | 3 | 0.37 (0.12-1.15) | 0.21 | Ineligible |
| T-DXd | Musculoskeletal pain | breast_cancer_primary | 3 | 0.14 (0.05-0.45) | 0.10 | Ineligible |
| T-DXd | Musculoskeletal pain | breast_cancer_primary | 3 | 0.14 (0.05-0.45) | 0.10 | Ineligible |
| T-DXd | Myelodysplastic syndrome | breast_cancer_primary | 3 | 0.48 (0.16-1.51) | 0.26 | Ineligible |
| T-DXd | Myelodysplastic syndrome | breast_cancer_primary | 3 | 0.48 (0.16-1.51) | 0.26 | Ineligible |
| T-DXd | Nail bed inflammation | breast_cancer_primary | 3 | 2.62 (0.81-8.41) | 0.61 | Ineligible |
| T-DXd | Nail bed inflammation | breast_cancer_primary | 3 | 2.62 (0.81-8.41) | 0.61 | Ineligible |
| T-DXd | Nasal mucosal disorder | breast_cancer_primary | 3 | 2.86 (0.89-9.23) | 0.64 | Ineligible |
| T-DXd | Nasal mucosal disorder | breast_cancer_primary | 3 | 2.86 (0.89-9.23) | 0.64 | Ineligible |
| T-DXd | Neck pain | breast_cancer_primary | 3 | 0.18 (0.06-0.55) | 0.12 | Ineligible |
| T-DXd | Neck pain | breast_cancer_primary | 3 | 0.18 (0.06-0.55) | 0.12 | Ineligible |
| T-DXd | Necrosis | breast_cancer_primary | 3 | 0.97 (0.31-3.04) | 0.39 | Ineligible |
| T-DXd | Necrosis | breast_cancer_primary | 3 | 0.97 (0.31-3.04) | 0.39 | Ineligible |
| T-DXd | Neurological decompensation | breast_cancer_primary | 3 | 2.20 (0.69-7.02) | 0.57 | Ineligible |
| T-DXd | Neurological decompensation | breast_cancer_primary | 3 | 2.20 (0.69-7.02) | 0.57 | Ineligible |
| T-DXd | Neutropenic sepsis | breast_cancer_primary | 3 | 0.29 (0.09-0.91) | 0.18 | Ineligible |
| T-DXd | Neutropenic sepsis | breast_cancer_primary | 3 | 0.29 (0.09-0.91) | 0.18 | Ineligible |
| T-DXd | Obstruction gastric | breast_cancer_primary | 3 | 6.48 (1.92-21.89) | 0.93 | Ineligible |
| T-DXd | Obstruction gastric | breast_cancer_primary | 3 | 6.48 (1.92-21.89) | 0.93 | Ineligible |
| T-DXd | Ocular discomfort | breast_cancer_primary | 3 | 1.41 (0.45-4.47) | 0.47 | Ineligible |
| T-DXd | Ocular discomfort | breast_cancer_primary | 3 | 1.41 (0.45-4.47) | 0.47 | Ineligible |
| T-DXd | Oesophageal ulcer | breast_cancer_primary | 3 | 2.56 (0.80-8.23) | 0.61 | Ineligible |
| T-DXd | Oesophageal ulcer | breast_cancer_primary | 3 | 2.56 (0.80-8.23) | 0.61 | Ineligible |
| T-DXd | Opportunistic infection | breast_cancer_primary | 3 | 17.58 (4.54-68.00) | 1.57 | Ineligible |
| T-DXd | Opportunistic infection | breast_cancer_primary | 3 | 17.58 (4.54-68.00) | 1.57 | Ineligible |
| T-DXd | Oral candidiasis | breast_cancer_primary | 3 | 0.48 (0.15-1.50) | 0.25 | Ineligible |
| T-DXd | Oral candidiasis | breast_cancer_primary | 3 | 0.48 (0.15-1.50) | 0.25 | Ineligible |
| T-DXd | Oral herpes | breast_cancer_primary | 3 | 0.43 (0.14-1.34) | 0.24 | Ineligible |
| T-DXd | Oral herpes | breast_cancer_primary | 3 | 0.43 (0.14-1.34) | 0.24 | Ineligible |
| T-DXd | Oral pain | breast_cancer_primary | 3 | 0.10 (0.03-0.32) | 0.07 | Ineligible |
| T-DXd | Oral pain | breast_cancer_primary | 3 | 0.10 (0.03-0.32) | 0.07 | Ineligible |
| T-DXd | Orthostatic hypotension | breast_cancer_primary | 3 | 1.37 (0.43-4.32) | 0.46 | Ineligible |
| T-DXd | Orthostatic hypotension | breast_cancer_primary | 3 | 1.37 (0.43-4.32) | 0.46 | Ineligible |
| T-DXd | Orthostatic intolerance | breast_cancer_primary | 3 | 15.38 (4.08-57.99) | 1.47 | Ineligible |
| T-DXd | Orthostatic intolerance | breast_cancer_primary | 3 | 15.38 (4.08-57.99) | 1.47 | Ineligible |
| T-DXd | Ovarian cyst | breast_cancer_primary | 3 | 1.10 (0.35-3.46) | 0.42 | Ineligible |
| T-DXd | Ovarian cyst | breast_cancer_primary | 3 | 1.10 (0.35-3.46) | 0.42 | Ineligible |
| T-DXd | Pain in jaw | breast_cancer_primary | 3 | 0.19 (0.06-0.61) | 0.13 | Ineligible |
| T-DXd | Pain in jaw | breast_cancer_primary | 3 | 0.19 (0.06-0.61) | 0.13 | Ineligible |
| T-DXd | Pancreatitis | breast_cancer_primary | 3 | 0.37 (0.12-1.15) | 0.21 | Ineligible |
| T-DXd | Pancreatitis | breast_cancer_primary | 3 | 0.37 (0.12-1.15) | 0.21 | Ineligible |
| T-DXd | Pancreatitis acute | breast_cancer_primary | 3 | 0.50 (0.16-1.55) | 0.26 | Ineligible |
| T-DXd | Pancreatitis acute | breast_cancer_primary | 3 | 0.50 (0.16-1.55) | 0.26 | Ineligible |
| T-DXd | Paraesthesia oral | breast_cancer_primary | 3 | 0.56 (0.18-1.76) | 0.28 | Ineligible |
| T-DXd | Paraesthesia oral | breast_cancer_primary | 3 | 0.56 (0.18-1.76) | 0.28 | Ineligible |
| T-DXd | Paronychia | breast_cancer_primary | 3 | 0.40 (0.13-1.25) | 0.22 | Ineligible |
| T-DXd | Paronychia | breast_cancer_primary | 3 | 0.40 (0.13-1.25) | 0.22 | Ineligible |
| T-DXd | Photophobia | breast_cancer_primary | 3 | 0.68 (0.22-2.13) | 0.32 | Ineligible |
| T-DXd | Photophobia | breast_cancer_primary | 3 | 0.68 (0.22-2.13) | 0.32 | Ineligible |
| T-DXd | Pigmentation disorder | breast_cancer_primary | 3 | 0.60 (0.19-1.88) | 0.30 | Ineligible |
| T-DXd | Pigmentation disorder | breast_cancer_primary | 3 | 0.60 (0.19-1.88) | 0.30 | Ineligible |
| T-DXd | Poor venous access | breast_cancer_primary | 3 | 1.13 (0.36-3.55) | 0.42 | Ineligible |
| T-DXd | Poor venous access | breast_cancer_primary | 3 | 1.13 (0.36-3.55) | 0.42 | Ineligible |
| T-DXd | Post procedural infection | breast_cancer_primary | 3 | 1.46 (0.46-4.63) | 0.48 | Ineligible |
| T-DXd | Post procedural infection | breast_cancer_primary | 3 | 1.46 (0.46-4.63) | 0.48 | Ineligible |
| T-DXd | Product storage error | breast_cancer_primary | 3 | 0.76 (0.24-2.38) | 0.34 | Ineligible |
| T-DXd | Product storage error | breast_cancer_primary | 3 | 0.76 (0.24-2.38) | 0.34 | Ineligible |
| T-DXd | Protein total decreased | breast_cancer_primary | 3 | 0.99 (0.32-3.12) | 0.39 | Ineligible |
| T-DXd | Protein total decreased | breast_cancer_primary | 3 | 0.99 (0.32-3.12) | 0.39 | Ineligible |
| T-DXd | Pseudomonas infection | breast_cancer_primary | 3 | 1.73 (0.55-5.50) | 0.51 | Ineligible |
| T-DXd | Pseudomonas infection | breast_cancer_primary | 3 | 1.73 (0.55-5.50) | 0.51 | Ineligible |
| T-DXd | Pulmonary arterial hypertension | breast_cancer_primary | 3 | 0.87 (0.28-2.72) | 0.37 | Ineligible |
| T-DXd | Pulmonary arterial hypertension | breast_cancer_primary | 3 | 0.87 (0.28-2.72) | 0.37 | Ineligible |
| T-DXd | Pyelonephritis acute | breast_cancer_primary | 3 | 4.56 (1.38-15.03) | 0.78 | Ineligible |
| T-DXd | Pyelonephritis acute | breast_cancer_primary | 3 | 4.56 (1.38-15.03) | 0.78 | Ineligible |
| T-DXd | Rash maculo-papular | breast_cancer_primary | 3 | 0.35 (0.11-1.10) | 0.20 | Ineligible |
| T-DXd | Rash maculo-papular | breast_cancer_primary | 3 | 0.35 (0.11-1.10) | 0.20 | Ineligible |
| T-DXd | Refusal of treatment by patient | breast_cancer_primary | 3 | 3.62 (1.11-11.79) | 0.71 | Ineligible |
| T-DXd | Refusal of treatment by patient | breast_cancer_primary | 3 | 3.62 (1.11-11.79) | 0.71 | Ineligible |
| T-DXd | Rhinitis | breast_cancer_primary | 3 | 0.79 (0.25-2.49) | 0.35 | Ineligible |
| T-DXd | Rhinitis | breast_cancer_primary | 3 | 0.79 (0.25-2.49) | 0.35 | Ineligible |
| T-DXd | Right ventricular enlargement | breast_cancer_primary | 3 | 15.38 (4.08-57.99) | 1.47 | Ineligible |
| T-DXd | Right ventricular enlargement | breast_cancer_primary | 3 | 15.38 (4.08-57.99) | 1.47 | Ineligible |
| T-DXd | Right ventricular failure | breast_cancer_primary | 3 | 1.89 (0.59-6.02) | 0.53 | Ineligible |
| T-DXd | Right ventricular failure | breast_cancer_primary | 3 | 1.89 (0.59-6.02) | 0.53 | Ineligible |
| T-DXd | Salivary hypersecretion | breast_cancer_primary | 3 | 1.41 (0.45-4.47) | 0.47 | Ineligible |
| T-DXd | Salivary hypersecretion | breast_cancer_primary | 3 | 1.41 (0.45-4.47) | 0.47 | Ineligible |
| T-DXd | Scan abnormal | breast_cancer_primary | 3 | 8.79 (2.53-30.59) | 1.09 | Ineligible |
| T-DXd | Scan abnormal | breast_cancer_primary | 3 | 8.79 (2.53-30.59) | 1.09 | Ineligible |
| T-DXd | Sluggishness | breast_cancer_primary | 3 | 1.16 (0.37-3.66) | 0.43 | Ineligible |
| T-DXd | Sluggishness | breast_cancer_primary | 3 | 1.16 (0.37-3.66) | 0.43 | Ineligible |
| T-DXd | Spinal fracture | breast_cancer_primary | 3 | 0.36 (0.12-1.13) | 0.21 | Ineligible |
| T-DXd | Spinal fracture | breast_cancer_primary | 3 | 0.36 (0.12-1.13) | 0.21 | Ineligible |
| T-DXd | Staphylococcal infection | breast_cancer_primary | 3 | 0.52 (0.17-1.62) | 0.27 | Ineligible |
| T-DXd | Staphylococcal infection | breast_cancer_primary | 3 | 0.52 (0.17-1.62) | 0.27 | Ineligible |
| T-DXd | Status epilepticus | breast_cancer_primary | 3 | 3.52 (1.08-11.43) | 0.70 | Ineligible |
| T-DXd | Status epilepticus | breast_cancer_primary | 3 | 3.52 (1.08-11.43) | 0.70 | Ineligible |
| T-DXd | Streptococcal sepsis | breast_cancer_primary | 3 | 2.09 (0.65-6.65) | 0.56 | Ineligible |
| T-DXd | Streptococcal sepsis | breast_cancer_primary | 3 | 2.09 (0.65-6.65) | 0.56 | Ineligible |
| T-DXd | Tachypnoea | breast_cancer_primary | 3 | 0.57 (0.18-1.77) | 0.28 | Ineligible |
| T-DXd | Tachypnoea | breast_cancer_primary | 3 | 0.57 (0.18-1.77) | 0.28 | Ineligible |
| T-DXd | Therapeutic product effect incomplete | breast_cancer_primary | 3 | 0.63 (0.20-1.97) | 0.30 | Ineligible |
| T-DXd | Therapeutic product effect incomplete | breast_cancer_primary | 3 | 0.63 (0.20-1.97) | 0.30 | Ineligible |
| T-DXd | Therapy non-responder | breast_cancer_primary | 3 | 0.34 (0.11-1.05) | 0.20 | Ineligible |
| T-DXd | Therapy non-responder | breast_cancer_primary | 3 | 0.34 (0.11-1.05) | 0.20 | Ineligible |
| T-DXd | Tooth disorder | breast_cancer_primary | 3 | 0.25 (0.08-0.78) | 0.16 | Ineligible |
| T-DXd | Tooth disorder | breast_cancer_primary | 3 | 0.25 (0.08-0.78) | 0.16 | Ineligible |
| T-DXd | Tumour marker abnormal | breast_cancer_primary | 3 | 0.99 (0.32-3.12) | 0.39 | Ineligible |
| T-DXd | Tumour marker abnormal | breast_cancer_primary | 3 | 0.99 (0.32-3.12) | 0.39 | Ineligible |
| T-DXd | Tumour marker decreased | breast_cancer_primary | 3 | 1.56 (0.49-4.93) | 0.49 | Ineligible |
| T-DXd | Tumour marker decreased | breast_cancer_primary | 3 | 1.56 (0.49-4.93) | 0.49 | Ineligible |
| T-DXd | Ulcer haemorrhage | breast_cancer_primary | 3 | 3.00 (0.93-9.69) | 0.65 | Ineligible |
| T-DXd | Ulcer haemorrhage | breast_cancer_primary | 3 | 3.00 (0.93-9.69) | 0.65 | Ineligible |
| T-DXd | Vascular device occlusion | breast_cancer_primary | 3 | 13.67 (3.70-50.52) | 1.38 | Ineligible |
| T-DXd | Vascular device occlusion | breast_cancer_primary | 3 | 13.67 (3.70-50.52) | 1.38 | Ineligible |
| T-DXd | Venous thrombosis limb | breast_cancer_primary | 3 | 1.60 (0.50-5.06) | 0.50 | Ineligible |
| T-DXd | Venous thrombosis limb | breast_cancer_primary | 3 | 1.60 (0.50-5.06) | 0.50 | Ineligible |
| T-DXd | Vomiting projectile | breast_cancer_primary | 3 | 4.10 (1.25-13.44) | 0.75 | Ineligible |
| T-DXd | Vomiting projectile | breast_cancer_primary | 3 | 4.10 (1.25-13.44) | 0.75 | Ineligible |
| T-DXd | White blood cell count abnormal | breast_cancer_primary | 3 | 0.25 (0.08-0.79) | 0.16 | Ineligible |
| T-DXd | White blood cell count abnormal | breast_cancer_primary | 3 | 0.25 (0.08-0.79) | 0.16 | Ineligible |
| T-DXd | White coat hypertension | breast_cancer_primary | 3 | 41.02 (8.28-203.27) | 2.32 | Ineligible |
| T-DXd | White coat hypertension | breast_cancer_primary | 3 | 41.02 (8.28-203.27) | 2.32 | Ineligible |
| Dato-DXd | Disease progression | dato_dxd_all_indication_cold_start | 70 | 53.70 (41.09-70.19) | 32.51 | Eligible |
| Dato-DXd | Disease progression | dato_dxd_all_indication_cold_start | 70 | 53.70 (41.09-70.19) | 32.51 | Eligible |
| Dato-DXd | Off label use | dato_dxd_all_indication_cold_start | 45 | 3.55 (2.58-4.87) | 2.49 | Eligible |
| Dato-DXd | Off label use | dato_dxd_all_indication_cold_start | 45 | 3.55 (2.58-4.87) | 2.49 | Eligible |
| Dato-DXd | Stomatitis | dato_dxd_all_indication_cold_start | 42 | 54.97 (39.67-76.19) | 33.80 | Eligible |
| Dato-DXd | Stomatitis | dato_dxd_all_indication_cold_start | 42 | 54.97 (39.67-76.19) | 33.80 | Eligible |
| Dato-DXd | No adverse event | dato_dxd_all_indication_cold_start | 34 | 13.81 (9.67-19.74) | 9.28 | Eligible |
| Dato-DXd | No adverse event | dato_dxd_all_indication_cold_start | 34 | 13.81 (9.67-19.74) | 9.28 | Eligible |
| Dato-DXd | Death | dato_dxd_all_indication_cold_start | 24 | 1.99 (1.31-3.02) | 1.18 | Eligible |
| Dato-DXd | Death | dato_dxd_all_indication_cold_start | 24 | 1.99 (1.31-3.02) | 1.18 | Eligible |
| Dato-DXd | Nausea | dato_dxd_all_indication_cold_start | 17 | 1.61 (0.99-2.63) | 1.06 | Ineligible |
| Dato-DXd | Nausea | dato_dxd_all_indication_cold_start | 17 | 1.61 (0.99-2.63) | 1.06 | Ineligible |
| Dato-DXd | Interstitial lung disease | dato_dxd_all_indication_cold_start | 11 | 17.32 (9.48-31.62) | 9.84 | Eligible |
| Dato-DXd | Interstitial lung disease | dato_dxd_all_indication_cold_start | 11 | 17.32 (9.48-31.62) | 9.84 | Eligible |
| Dato-DXd | Infusion related reaction | dato_dxd_all_indication_cold_start | 10 | 10.89 (5.80-20.46) | 6.40 | Eligible |
| Dato-DXd | Infusion related reaction | dato_dxd_all_indication_cold_start | 10 | 10.89 (5.80-20.46) | 6.40 | Eligible |
| Dato-DXd | Prescribed underdose | dato_dxd_all_indication_cold_start | 9 | 27.51 (14.17-53.42) | 13.57 | Ineligible |
| Dato-DXd | Prescribed underdose | dato_dxd_all_indication_cold_start | 9 | 27.51 (14.17-53.42) | 13.57 | Ineligible |
| Dato-DXd | Dry eye | dato_dxd_all_indication_cold_start | 8 | 11.47 (5.68-23.16) | 6.39 | Ineligible |
| Dato-DXd | Dry eye | dato_dxd_all_indication_cold_start | 8 | 11.47 (5.68-23.16) | 6.39 | Ineligible |
| Dato-DXd | Dyspnoea | dato_dxd_all_indication_cold_start | 7 | 0.89 (0.42-1.88) | 0.87 | Ineligible |
| Dato-DXd | Dyspnoea | dato_dxd_all_indication_cold_start | 7 | 0.89 (0.42-1.88) | 0.87 | Ineligible |
| Dato-DXd | Fatigue | dato_dxd_all_indication_cold_start | 7 | 0.60 (0.29-1.28) | 0.75 | Ineligible |
| Dato-DXd | Fatigue | dato_dxd_all_indication_cold_start | 7 | 0.60 (0.29-1.28) | 0.75 | Ineligible |
| Dato-DXd | Pyrexia | dato_dxd_all_indication_cold_start | 7 | 1.51 (0.72-3.20) | 0.98 | Ineligible |
| Dato-DXd | Pyrexia | dato_dxd_all_indication_cold_start | 7 | 1.51 (0.72-3.20) | 0.98 | Ineligible |
| Dato-DXd | Cough | dato_dxd_all_indication_cold_start | 6 | 1.48 (0.66-3.31) | 0.97 | Ineligible |
| Dato-DXd | Cough | dato_dxd_all_indication_cold_start | 6 | 1.48 (0.66-3.31) | 0.97 | Ineligible |
| Dato-DXd | Malaise | dato_dxd_all_indication_cold_start | 6 | 0.94 (0.42-2.10) | 0.89 | Ineligible |
| Dato-DXd | Malaise | dato_dxd_all_indication_cold_start | 6 | 0.94 (0.42-2.10) | 0.89 | Ineligible |
| Dato-DXd | Rash | dato_dxd_all_indication_cold_start | 6 | 0.97 (0.43-2.17) | 0.89 | Ineligible |
| Dato-DXd | Rash | dato_dxd_all_indication_cold_start | 6 | 0.97 (0.43-2.17) | 0.89 | Ineligible |
| Dato-DXd | Alopecia | dato_dxd_all_indication_cold_start | 5 | 1.67 (0.69-4.04) | 0.98 | Ineligible |
| Dato-DXd | Alopecia | dato_dxd_all_indication_cold_start | 5 | 1.67 (0.69-4.04) | 0.98 | Ineligible |
| Dato-DXd | Mucosal inflammation | dato_dxd_all_indication_cold_start | 5 | 14.46 (5.97-34.99) | 6.78 | Ineligible |
| Dato-DXd | Mucosal inflammation | dato_dxd_all_indication_cold_start | 5 | 14.46 (5.97-34.99) | 6.78 | Ineligible |
| Dato-DXd | Pneumonitis | dato_dxd_all_indication_cold_start | 5 | 13.00 (5.37-31.47) | 6.21 | Ineligible |
| Dato-DXd | Pneumonitis | dato_dxd_all_indication_cold_start | 5 | 13.00 (5.37-31.47) | 6.21 | Ineligible |
| Dato-DXd | Product dose omission issue | dato_dxd_all_indication_cold_start | 5 | 1.12 (0.46-2.71) | 0.92 | Ineligible |
| Dato-DXd | Product dose omission issue | dato_dxd_all_indication_cold_start | 5 | 1.12 (0.46-2.71) | 0.92 | Ineligible |
| Dato-DXd | Product prescribing issue | dato_dxd_all_indication_cold_start | 5 | 16.91 (6.99-40.93) | 7.67 | Ineligible |
| Dato-DXd | Product prescribing issue | dato_dxd_all_indication_cold_start | 5 | 16.91 (6.99-40.93) | 7.67 | Ineligible |
| Dato-DXd | Constipation | dato_dxd_all_indication_cold_start | 4 | 1.30 (0.48-3.48) | 0.94 | Ineligible |
| Dato-DXd | Constipation | dato_dxd_all_indication_cold_start | 4 | 1.30 (0.48-3.48) | 0.94 | Ineligible |
| Dato-DXd | Decreased appetite | dato_dxd_all_indication_cold_start | 4 | 1.18 (0.44-3.18) | 0.93 | Ineligible |
| Dato-DXd | Decreased appetite | dato_dxd_all_indication_cold_start | 4 | 1.18 (0.44-3.18) | 0.93 | Ineligible |
| Dato-DXd | Dehydration | dato_dxd_all_indication_cold_start | 4 | 2.34 (0.87-6.28) | 1.00 | Ineligible |
| Dato-DXd | Dehydration | dato_dxd_all_indication_cold_start | 4 | 2.34 (0.87-6.28) | 1.00 | Ineligible |
| Dato-DXd | Dry skin | dato_dxd_all_indication_cold_start | 4 | 1.95 (0.73-5.22) | 0.98 | Ineligible |
| Dato-DXd | Dry skin | dato_dxd_all_indication_cold_start | 4 | 1.95 (0.73-5.22) | 0.98 | Ineligible |
| Dato-DXd | Lung neoplasm malignant | dato_dxd_all_indication_cold_start | 4 | 6.49 (2.42-17.40) | 1.31 | Ineligible |
| Dato-DXd | Lung neoplasm malignant | dato_dxd_all_indication_cold_start | 4 | 6.49 (2.42-17.40) | 1.31 | Ineligible |
| Dato-DXd | Oral candidiasis | dato_dxd_all_indication_cold_start | 4 | 23.11 (8.62-62.01) | 8.72 | Ineligible |
| Dato-DXd | Oral candidiasis | dato_dxd_all_indication_cold_start | 4 | 23.11 (8.62-62.01) | 8.72 | Ineligible |
| Dato-DXd | Weight decreased | dato_dxd_all_indication_cold_start | 4 | 1.03 (0.38-2.77) | 0.91 | Ineligible |
| Dato-DXd | Weight decreased | dato_dxd_all_indication_cold_start | 4 | 1.03 (0.38-2.77) | 0.91 | Ineligible |
| Dato-DXd | Weight increased | dato_dxd_all_indication_cold_start | 4 | 1.32 (0.49-3.54) | 0.94 | Ineligible |
| Dato-DXd | Weight increased | dato_dxd_all_indication_cold_start | 4 | 1.32 (0.49-3.54) | 0.94 | Ineligible |
| Dato-DXd | Anaemia | dato_dxd_all_indication_cold_start | 3 | 1.16 (0.37-3.62) | 0.92 | Ineligible |
| Dato-DXd | Anaemia | dato_dxd_all_indication_cold_start | 3 | 1.16 (0.37-3.62) | 0.92 | Ineligible |
| Dato-DXd | Asthenia | dato_dxd_all_indication_cold_start | 3 | 0.58 (0.18-1.80) | 0.82 | Ineligible |
| Dato-DXd | Asthenia | dato_dxd_all_indication_cold_start | 3 | 0.58 (0.18-1.80) | 0.82 | Ineligible |
| Dato-DXd | Chest discomfort | dato_dxd_all_indication_cold_start | 3 | 2.17 (0.70-6.76) | 0.98 | Ineligible |
| Dato-DXd | Chest discomfort | dato_dxd_all_indication_cold_start | 3 | 2.17 (0.70-6.76) | 0.98 | Ineligible |
| Dato-DXd | Hepatic function abnormal | dato_dxd_all_indication_cold_start | 3 | 6.27 (2.01-19.55) | 1.17 | Ineligible |
| Dato-DXd | Hepatic function abnormal | dato_dxd_all_indication_cold_start | 3 | 6.27 (2.01-19.55) | 1.17 | Ineligible |
| Dato-DXd | Keratitis | dato_dxd_all_indication_cold_start | 3 | 75.13 (24.07-234.46) | 12.56 | Ineligible |
| Dato-DXd | Keratitis | dato_dxd_all_indication_cold_start | 3 | 75.13 (24.07-234.46) | 12.56 | Ineligible |
| Dato-DXd | Lacrimation increased | dato_dxd_all_indication_cold_start | 3 | 6.91 (2.22-21.55) | 1.21 | Ineligible |
| Dato-DXd | Lacrimation increased | dato_dxd_all_indication_cold_start | 3 | 6.91 (2.22-21.55) | 1.21 | Ineligible |
| Dato-DXd | Oral pain | dato_dxd_all_indication_cold_start | 3 | 9.56 (3.07-29.83) | 1.45 | Ineligible |
| Dato-DXd | Oral pain | dato_dxd_all_indication_cold_start | 3 | 9.56 (3.07-29.83) | 1.45 | Ineligible |
| Dato-DXd | Pruritus | dato_dxd_all_indication_cold_start | 3 | 0.55 (0.18-1.73) | 0.81 | Ineligible |
| Dato-DXd | Pruritus | dato_dxd_all_indication_cold_start | 3 | 0.55 (0.18-1.73) | 0.81 | Ineligible |
| Dato-DXd | Pulmonary embolism | dato_dxd_all_indication_cold_start | 3 | 2.63 (0.84-8.19) | 0.99 | Ineligible |
| Dato-DXd | Pulmonary embolism | dato_dxd_all_indication_cold_start | 3 | 2.63 (0.84-8.19) | 0.99 | Ineligible |
| Dato-DXd | Seizure | dato_dxd_all_indication_cold_start | 3 | 1.57 (0.50-4.89) | 0.95 | Ineligible |
| Dato-DXd | Seizure | dato_dxd_all_indication_cold_start | 3 | 1.57 (0.50-4.89) | 0.95 | Ineligible |
| Dato-DXd | Therapy change | dato_dxd_all_indication_cold_start | 3 | 19.02 (6.10-59.34) | 6.42 | Ineligible |
| Dato-DXd | Therapy change | dato_dxd_all_indication_cold_start | 3 | 19.02 (6.10-59.34) | 6.42 | Ineligible |
| Dato-DXd | Underdose | dato_dxd_all_indication_cold_start | 3 | 2.52 (0.81-7.84) | 0.99 | Ineligible |
| Dato-DXd | Underdose | dato_dxd_all_indication_cold_start | 3 | 2.52 (0.81-7.84) | 0.99 | Ineligible |
| Dato-DXd | Disease progression | dato_dxd_breast_cancer_descriptive | 65 | 23.89 (17.26-33.08) | 11.10 | Eligible |
| Dato-DXd | Stomatitis | dato_dxd_breast_cancer_descriptive | 29 | 13.02 (8.67-19.55) | 7.68 | Eligible |
| Dato-DXd | Off label use | dato_dxd_breast_cancer_descriptive | 21 | 3.69 (2.32-5.86) | 2.00 | Eligible |
| Dato-DXd | Nausea | dato_dxd_breast_cancer_descriptive | 13 | 1.17 (0.66-2.07) | 0.71 | Ineligible |
| Dato-DXd | No adverse event | dato_dxd_breast_cancer_descriptive | 9 | 12.74 (6.48-25.04) | 6.53 | Ineligible |
| Dato-DXd | Prescribed underdose | dato_dxd_breast_cancer_descriptive | 9 | 199.21 (98.35-403.48) | 57.46 | Ineligible |
| Dato-DXd | Infusion related reaction | dato_dxd_breast_cancer_descriptive | 6 | 8.94 (3.94-20.27) | 3.73 | Ineligible |
| Dato-DXd | Dry eye | dato_dxd_breast_cancer_descriptive | 5 | 10.14 (4.15-24.78) | 3.84 | Ineligible |
| Dato-DXd | Interstitial lung disease | dato_dxd_breast_cancer_descriptive | 5 | 3.10 (1.27-7.57) | 0.95 | Ineligible |
| Dato-DXd | Malaise | dato_dxd_breast_cancer_descriptive | 5 | 1.27 (0.52-3.09) | 0.58 | Ineligible |
| Dato-DXd | Pyrexia | dato_dxd_breast_cancer_descriptive | 5 | 1.19 (0.49-2.90) | 0.56 | Ineligible |
| Dato-DXd | Constipation | dato_dxd_breast_cancer_descriptive | 4 | 1.22 (0.45-3.29) | 0.53 | Ineligible |
| Dato-DXd | Death | dato_dxd_breast_cancer_descriptive | 4 | 0.41 (0.15-1.10) | 0.25 | Ineligible |
| Dato-DXd | Dyspnoea | dato_dxd_breast_cancer_descriptive | 4 | 0.69 (0.26-1.86) | 0.37 | Ineligible |
| Dato-DXd | Alopecia | dato_dxd_breast_cancer_descriptive | 3 | 0.19 (0.06-0.59) | 0.12 | Ineligible |
| Dato-DXd | Chest discomfort | dato_dxd_breast_cancer_descriptive | 3 | 3.06 (0.98-9.61) | 0.73 | Ineligible |
| Dato-DXd | Cough | dato_dxd_breast_cancer_descriptive | 3 | 0.92 (0.29-2.88) | 0.42 | Ineligible |
| Dato-DXd | Decreased appetite | dato_dxd_breast_cancer_descriptive | 3 | 0.64 (0.20-2.01) | 0.34 | Ineligible |
| Dato-DXd | Fatigue | dato_dxd_breast_cancer_descriptive | 3 | 0.21 (0.07-0.66) | 0.14 | Ineligible |
| Dato-DXd | Hepatic function abnormal | dato_dxd_breast_cancer_descriptive | 3 | 3.22 (1.03-10.11) | 0.74 | Ineligible |
| Dato-DXd | Pruritus | dato_dxd_breast_cancer_descriptive | 3 | 1.13 (0.36-3.54) | 0.46 | Ineligible |
| Dato-DXd | Rash | dato_dxd_breast_cancer_descriptive | 3 | 0.73 (0.23-2.29) | 0.36 | Ineligible |
| Dato-DXd | Underdose | dato_dxd_breast_cancer_descriptive | 3 | 13.68 (4.34-43.09) | 3.00 | Ineligible |
| SKB264 | Myelosuppression | skb264_all_indication_sparse | 17 | 4871.36 (1125.40-21085.94) | 472.89 | Eligible |
| SKB264 | Off label use | skb264_all_indication_sparse | 7 | 11.68 (4.60-29.67) | 7.13 | Ineligible |
| SKB264 | Myelosuppression | skb264_breast_cancer_sparse | 8 | 803.91 (46.39-13930.36) | 43.42 | Ineligible |

**Supplementary Table S3A. All-indication adjusted reporting-odds sensitivity analysis.**
Models were fitted within the all-indication FAERS cohort (N = 20,526) using T-DM1 as the reference and the same covariate structure as the primary breast-cancer analysis. Adjusted reporting odds ratios (aRORs) represent relative reporting within FAERS and should not be interpreted as comparative clinical risk. P values are nominal and were used for exploratory signal characterization. AESI, adverse event of special interest; aROR, adjusted reporting odds ratio; CI, confidence interval; FAERS, FDA Adverse Event Reporting System; SG, sacituzumab govitecan; T-DM1, trastuzumab emtansine; T-DXd, trastuzumab deruxtecan.

| **AESI domain** | **Comparison** | **aROR (95% CI)** | **P value** |
| --- | --- | --- | --- |
| Cardiac toxicity | SG vs T-DM1 | 0.15 (0.09–0.25) | <0.001 |
|  | T-DXd vs T-DM1 | 0.78 (0.57–1.08) | 0.14 |
| Gastrointestinal toxicity | SG vs T-DM1 | 2.39 (2.06–2.78) | <0.001 |
|  | T-DXd vs T-DM1 | 2.56 (2.22–2.95) | <0.001 |
| Hematologic toxicity | SG vs T-DM1 | 2.02 (1.76–2.32) | <0.001 |
|  | T-DXd vs T-DM1 | 0.76 (0.66–0.87) | <0.001 |
| Hepatobiliary toxicity | SG vs T-DM1 | 0.31 (0.24–0.40) | <0.001 |
|  | T-DXd vs T-DM1 | 0.39 (0.32–0.49) | <0.001 |
| ILD/pneumonitis | SG vs T-DM1 | 0.45 (0.34–0.60) | <0.001 |
|  | T-DXd vs T-DM1 | 3.41 (2.77–4.18) | <0.001 |
| Infection/febrile events | SG vs T-DM1 | 1.00 (0.82–1.22) | 0.995 |
|  | T-DXd vs T-DM1 | 0.88 (0.73–1.06) | 0.167 |
| Infusion/hypersensitivity | SG vs T-DM1 | 0.88 (0.56–1.39) | 0.578 |
|  | T-DXd vs T-DM1 | 0.42 (0.26–0.67) | <0.001 |
| Ocular toxicity | SG vs T-DM1 | 0.24 (0.15–0.40) | <0.001 |
|  | T-DXd vs T-DM1 | 0.58 (0.41–0.83) | 0.003 |
| Peripheral neuropathy | SG vs T-DM1 | 0.14 (0.10–0.20) | <0.001 |
|  | T-DXd vs T-DM1 | 0.18 (0.14–0.23) | <0.001 |
| Renal toxicity | SG vs T-DM1 | 1.18 (0.75–1.86) | 0.476 |
|  | T-DXd vs T-DM1 | 1.30 (0.85–2.01) | 0.226 |
| Skin toxicity | SG vs T-DM1 | 1.88 (1.47–2.41) | <0.001 |
|  | T-DXd vs T-DM1 | 1.47 (1.16–1.87) | 0.002 |

**Supplementary Table S3B. Reported time to onset for selected AESI domains by ADC in the breast-cancer primary FAERS population.**

TTO was calculated only for reports with valid treatment-start and event-onset dates. TTO completeness represents the proportion of AESI reports with valid TTO information. Medians and interquartile ranges are descriptive and should not be interpreted as comparative biological latency because of incomplete and potentially differential date reporting in FAERS. AESI, adverse event of special interest; FAERS, FDA Adverse Event Reporting System; IQR, interquartile range; ADC, antibody–drug conjugate; SG, sacituzumab govitecan; T-DM1, trastuzumab emtansine; T-DXd, trastuzumab deruxtecan; TTO, time to onset.

| **AESI domain** | **ADC** | **AESI reports, n** | **Valid TTO, n** | **TTO completeness** | **Median TTO, days** | **IQR, days** |
| --- | --- | --- | --- | --- | --- | --- |
| ILD/pneumonitis | T-DM1 | 139 | 59 | 42.40% | 109 | 30.5–205.5 |
| ILD/pneumonitis | T-DXd | 863 | 248 | 28.70% | 78 | 24–186.3 |
| ILD/pneumonitis | SG | 55 | 25 | 45.50% | 35 | 12–87 |
| Hematologic toxicity | T-DM1 | 585 | 254 | 43.40% | 25 | 6–124.5 |
| Hematologic toxicity | T-DXd | 588 | 241 | 41.00% | 21 | 7–63 |
| Hematologic toxicity | SG | 792 | 370 | 46.70% | 11.5 | 6–21 |
| Gastrointestinal toxicity | T-DM1 | 376 | 138 | 36.70% | 20.5 | 1–61 |
| Gastrointestinal toxicity | T-DXd | 1,209 | 316 | 26.10% | 11 | 1–42.3 |
| Gastrointestinal toxicity | SG | 678 | 293 | 43.20% | 11 | 5–34 |

**Supplementary Table S4. Temporal-validation feature-ablation analysis of report-level machine-learning signal prioritization.**

Drug-only baseline (M0), full contextual model including drug identity (M1), and contextual model excluding drug identity (M2). AUROC and AUPRC are reported with 95% bootstrap CIs. Δ values represent differences between models. AUPRC lift and top-10% enrichment are relative to validation prevalence. Models were intended for report-level pharmacovigilance prioritization, not patient-level risk prediction. AUPRC, area under the precision–recall curve; AUROC, area under the receiver operating characteristic curve; CI, confidence interval.

| **AESI domain** | **Validation N** | **Prevalence** | **M0 AUROC (95% CI)** | **M0 AUPRC (95% CI)** | **M1 AUROC (95% CI)** | **M1 AUPRC (95% CI)** | **M1 AUPRC lift** | **M1 calibration slope** | **M1 top-10% enrichment** | **M2 AUROC (95% CI)** | **M2 AUPRC (95% CI)** | **ΔAUROC M1−M0** | **ΔAUPRC M1−M0** | **ΔAUROC M1−M2** |
| --- | --- | --- | --- | --- | --- | --- | --- | --- | --- | --- | --- | --- | --- | --- |
| ILD/pneumonitis | 8241 | 0.089 | 0.659 (0.647-0.670) | 0.127 (0.118-0.136) | 0.772 (0.755-0.787) | 0.216 (0.197-0.238) | 2.441 | 1.078 | 2.518 | 0.706 (0.687-0.724) | 0.172 (0.156-0.193) | 0.112 | 0.089 | 0.066 |
| Hematologic toxicity | 8241 | 0.142 | 0.648 (0.631-0.663) | 0.211 (0.196-0.225) | 0.714 (0.697-0.730) | 0.279 (0.259-0.303) | 1.963 | 1.261 | 2.303 | 0.666 (0.649-0.682) | 0.230 (0.212-0.251) | 0.066 | 0.068 | 0.048 |
| Gastrointestinal toxicity | 8241 | 0.174 | 0.518 (0.505-0.531) | 0.179 (0.170-0.188) | 0.628 (0.611-0.643) | 0.268 (0.248-0.287) | 1.541 | 0.772 | 2.091 | 0.631 (0.616-0.647) | 0.269 (0.251-0.290) | 0.109 | 0.089 | -0.004 |
| Hepatobiliary toxicity | 8241 | 0.031 | 0.574 (0.537-0.610) | 0.042 (0.035-0.051) | 0.628 (0.590-0.667) | 0.069 (0.052-0.096) | 2.235 | 0.997 | 2.792 | 0.587 (0.552-0.622) | 0.046 (0.036-0.064) | 0.055 | 0.026 | 0.041 |
| Cardiac toxicity | 8241 | 0.018 | 0.562 (0.527-0.593) | 0.020 (0.017-0.024) | 0.627 (0.580-0.669) | 0.031 (0.022-0.051) | 1.753 | 0.370 | 1.779 | 0.574 (0.525-0.621) | 0.026 (0.019-0.045) | 0.065 | 0.011 | 0.053 |
| Ocular toxicity | 8241 | 0.013 | 0.502 (0.449-0.550) | 0.013 (0.011-0.016) | 0.682 (0.626-0.739) | 0.032 (0.023-0.048) | 2.483 | 0.663 | 3.393 | 0.654 (0.593-0.712) | 0.024 (0.018-0.033) | 0.180 | 0.019 | 0.027 |
| Skin toxicity | 8241 | 0.046 | 0.566 (0.539-0.591) | 0.054 (0.048-0.060) | 0.677 (0.647-0.705) | 0.094 (0.081-0.110) | 2.031 | 0.836 | 2.786 | 0.675 (0.645-0.703) | 0.093 (0.080-0.110) | 0.112 | 0.040 | 0.003 |
| Infection/febrile events | 8241 | 0.062 | 0.508 (0.486-0.531) | 0.062 (0.056-0.069) | 0.616 (0.592-0.641) | 0.103 (0.090-0.122) | 1.668 | 1.058 | 2.033 | 0.620 (0.597-0.646) | 0.105 (0.090-0.124) | 0.109 | 0.041 | -0.004 |
| Infusion/hypersensitivity | 8241 | 0.007 | 0.595 (0.533-0.656) | 0.009 (0.006-0.011) | 0.593 (0.515-0.673) | 0.014 (0.007-0.049) | 2.077 | 0.306 | 0.714 | 0.599 (0.512-0.683) | 0.014 (0.009-0.026) | -0.002 | 0.005 | -0.007 |
| Renal toxicity | 8241 | 0.014 | 0.506 (0.467-0.550) | 0.015 (0.012-0.017) | 0.701 (0.654-0.745) | 0.031 (0.023-0.045) | 2.167 | 1.029 | 2.518 | 0.708 (0.661-0.751) | 0.032 (0.023-0.047) | 0.194 | 0.017 | -0.008 |
| Peripheral neuropathy | 8241 | 0.018 | 0.575 (0.527-0.621) | 0.027 (0.021-0.035) | 0.683 (0.638-0.729) | 0.073 (0.046-0.112) | 3.986 | 0.708 | 3.308 | 0.651 (0.603-0.696) | 0.044 (0.031-0.065) | 0.108 | 0.046 | 0.032 |

**Supplementary Table S5. Institutional AESI frequencies, severity, management, and temporal outcomes by ADC.**

AESI frequencies were calculated using fixed treatment-group denominators of 31 patients for T-DM1, 50 for T-DXd, and 24 for sacituzumab govitecan. Each patient was counted once within each AESI domain. When multiple events occurred within the same domain, the highest final CTCAE v5.0 grade and the earliest valid onset date were retained. Dose modification was defined as any documented treatment interruption, dose reduction, treatment delay, or permanent discontinuation associated with the corresponding AESI. Hospitalization refers to all-cause hospitalization documented after AESI onset and does not imply causal attribution to the AESI. TTO summaries were restricted to events with valid onset information; for a single event, the observed onset day is reported, whereas distributions with multiple events are summarized using the median and IQR or range, as appropriate. Deaths were summarized separately at the overall-cohort level rather than within individual AESI domains. Twelve all-cause deaths occurred during follow-up; case-by-case source-record review attributed all 12 deaths to disease progression, and no death was attributed to an institutional AESI. ADC, antibody–drug conjugate; AESI, adverse event of special interest; CTCAE, Common Terminology Criteria for Adverse Events; IQR, interquartile range; T-DM1, trastuzumab emtansine; T-DXd, trastuzumab deruxtecan; TTO, time to onset; CI, confidence interval.

| **ADC** | **AESI domain** | **Observed proportion, n/N (95% CI)** | **Grade ≥3, n** | **Any dose modification, n** | **Hospitalization after AESI, n** | **TTO, median days (IQR/range)** | **Valid TTO, n** |
| --- | --- | --- | --- | --- | --- | --- | --- |
| T-DM1 | Hematologic toxicity | 12/31 (38.7%; 21.8–57.8) | 2 | 3 | 1 | 40.5 (30.8–45.2) | 12 |
|  | Gastrointestinal toxicity | 9/31 (29.0%; 14.2–48.0) | 1 | 2 | 0 | 17 (15–22) | 9 |
|  | Hepatobiliary toxicity | 10/31 (32.3%; 16.7–51.4) | 1 | 4 | 0 | 56.5 (44–62) | 10 |
|  | ILD/pneumonitis | 1/31 (3.2%; 0.1–16.7) | 0 | 1 | 0 | 96 | 1 |
|  | Skin toxicity | 2/31 (6.5%; 0.8–21.4) | 1 | 2 | 0 | 71 (68–74) | 2 |
|  | Infection/febrile events | 2/31 (6.5%; 0.8–21.4) | 2 | 2 | 1 | 46.5 (16–77) | 2 |
|  | Peripheral neuropathy | 5/31 (16.1%; 5.5–33.7) | 1 | 1 | 0 | 76 (71–80) | 5 |
|  | Cardiac toxicity | 1/31 (3.2%; 0.1–16.7) | 0 | 1 | 0 | 34 | 1 |
|  | Infusion/hypersensitivity | 1/31 (3.2%; 0.1–16.7) | 0 | 0 | 0 | 82 | 1 |
|  | Renal abnormality | 1/31 (3.2%; 0.1–16.7) | 0 | 0 | 0 | 11 | 1 |
|  | Ocular toxicity | 1/31 (3.2%; 0.1–16.7) | 0 | 0 | 0 | 19 | 1 |
| T-DXd | Hematologic toxicity | 22/50 (44.0%; 30.0–58.7) | 2 | 6 | 1 | 29 (22–37.5) | 22 |
|  | Gastrointestinal toxicity | 30/50 (60.0%; 45.2–73.6) | 7 | 9 | 4 | 16 (11.2–18.8) | 30 |
|  | Hepatobiliary toxicity | 8/50 (16.0%; 7.2–29.1) | 0 | 1 | 0 | 69.5 (59.5–77) | 8 |
|  | ILD/pneumonitis | 6/50 (12.0%; 4.5–24.3) | 3 | 6 | 2 | 182 (128–205.2) | 6 |
|  | Skin toxicity | 6/50 (12.0%; 4.5–24.3) | 0 | 1 | 0 | 54 (38–60.2) | 6 |
|  | Infection/febrile events | 4/50 (8.0%; 2.2–19.2) | 4 | 4 | 1 | 61 (51.8–66.8) | 4 |
|  | Peripheral neuropathy | 3/50 (6.0%; 1.3–16.5) | 0 | 2 | 0 | 32 (12–88) | 3 |
|  | Cardiac toxicity | 2/50 (4.0%; 0.5–13.7) | 1 | 1 | 0 | 79.5 (77–82) | 2 |
|  | Infusion/hypersensitivity | 2/50 (4.0%; 0.5–13.7) | 0 | 0 | 0 | 56.5 (37–76) | 2 |
|  | Renal abnormality | 2/50 (4.0%; 0.5–13.7) | 0 | 0 | 0 | 70.5 (51–90) | 2 |
|  | Ocular toxicity | 1/50 (2.0%; 0.1–10.6) | 0 | 0 | 0 | 41 | 1 |
| Sacituzumab govitecan | Hematologic toxicity | 16/24 (66.7%; 44.7–84.4) | 3 | 4 | 0 | 21 (15.2–23) | 16 |
|  | Gastrointestinal toxicity | 15/24 (62.5%; 40.6–81.2) | 2 | 2 | 1 | 10 (7.5–12.5) | 15 |
|  | Hepatobiliary toxicity | 2/24 (8.3%; 1.0–27.0) | 0 | 0 | 0 | 17.5 (10–25) | 2 |
|  | ILD/pneumonitis | 0/24 (0.0%; 0.0–14.2) | 0 | 0 | 0 | — | 0 |
|  | Skin toxicity | 4/24 (16.7%; 4.7–37.4) | 0 | 1 | 0 | 50 (26.8–78.2) | 4 |
|  | Infection/febrile events | 4/24 (16.7%; 4.7–37.4) | 4 | 4 | 3 | 47.5 (29.2–65.2) | 4 |
|  | Peripheral neuropathy | 1/24 (4.2%; 0.1–21.1) | 0 | 1 | 0 | 67 | 1 |
|  | Cardiac toxicity | 0/24 (0.0%; 0.0–14.2) | 0 | 0 | 0 | — | 0 |
|  | Infusion/hypersensitivity | 1/24 (4.2%; 0.1–21.1) | 0 | 0 | 0 | 76 | 1 |
|  | Renal abnormality | 1/24 (4.2%; 0.1–21.1) | 1 | 1 | 1 | 18 | 1 |
|  | Ocular toxicity | 0/24 (0.0%; 0.0–14.2) | 0 | 0 | 0 | — | 0 |

**Supplementary Table S6. Overall institutional burden and clinical consequences of documented AESIs**

AESIs were reconstructed from the final patient-level AESI summaries. All percentages use the full institutional cohort (N = 105) as the denominator to describe overall cohort burden and should not be interpreted as conditional rates among patients experiencing a given AESI. Grade refers to the final CTCAE v5.0 grade retained within each patient–AESI domain after the clinical data-extraction and quality-control procedures described in the Methods. Source-record re-audit was additionally performed for the previously flagged ILD/pneumonitis and ocular events before final dataset reconciliation. Dose modification was defined as any documented treatment interruption, dose reduction, treatment delay, or permanent discontinuation occurring in association with the corresponding AESI. Hospitalization represents all-cause hospitalization documented after AESI onset; temporal sequence does not establish causal attribution to the AESI. Twelve all-cause deaths occurred during cohort follow-up; case-by-case source-record review attributed all 12 deaths to disease progression, and no death was attributed to an institutional AESI. Death was therefore summarized at the cohort level rather than as an AESI-specific temporal consequence. The institutional data are descriptive and were used for clinical contextualization rather than formal comparative safety assessment. AESI, adverse event of special interest; CTCAE, Common Terminology Criteria for Adverse Events; ILD, interstitial lung disease.

| **AESI domain** | **Clinically meaningful AESI, n/105 (%)** | **Grade ≥3, n/105 (%)** | **Dose modification, n/105 (%)** | **All-cause hospitalization after AESI, n/105 (%)** |
| --- | --- | --- | --- | --- |
| Hematologic toxicity | 50/105 (47.6%) | 7/105 (6.7%) | 13/105 (12.4%) | 2/105 (1.9%) |
| Gastrointestinal toxicity | 54/105 (51.4%) | 10/105 (9.5%) | 13/105 (12.4%) | 5/105 (4.8%) |
| Hepatobiliary toxicity | 20/105 (19.0%) | 1/105 (1.0%) | 5/105 (4.8%) | 0/105 (0.0%) |
| ILD/pneumonitis | 7/105 (6.7%) | 3/105 (2.9%) | 7/105 (6.7%) | 2/105 (1.9%) |
| Skin toxicity | 12/105 (11.4%) | 1/105 (1.0%) | 4/105 (3.8%) | 0/105 (0.0%) |
| Infection/febrile events | 10/105 (9.5%) | 10/105 (9.5%) | 10/105 (9.5%) | 5/105 (4.8%) |
| Peripheral neuropathy | 9/105 (8.6%) | 1/105 (1.0%) | 4/105 (3.8%) | 0/105 (0.0%) |
| Cardiac toxicity | 3/105 (2.9%) | 1/105 (1.0%) | 2/105 (1.9%) | 0/105 (0.0%) |
| Infusion/hypersensitivity | 4/105 (3.8%) | 0/105 (0.0%) | 0/105 (0.0%) | 0/105 (0.0%) |
| Renal abnormality | 4/105 (3.8%) | 1/105 (1.0%) | 1/105 (1.0%) | 1/105 (1.0%) |
| Ocular toxicity | 2/105 (1.9%) | 0/105 (0.0%) | 0/105 (0.0%) | 0/105 (0.0%) |

**Supplementary Table S7. Institutional exact-test statistical results.**
Rows were derived from the final patient-level drug-by-AESI contingency tables. Overall three-group comparisons were performed using Fisher–Freeman–Halton exact tests. These omnibus P values were exploratory and nominal, with no multiplicity adjustment across the 11 AESI domains. Prespecified pairwise comparisons were performed using two-sided Fisher exact tests and adjusted for multiple testing using the Holm method across 12 comparisons. “Unadjusted” refers to the absence of baseline-covariate adjustment and should not be interpreted as absence of multiplicity control for the pairwise analyses. AESI, adverse event of special interest.

**Panel A. Three-group Fisher–Freeman–Halton exact tests.**

Complete conditional enumeration for 3 × 2 Fisher–Freeman–Halton tests.

| **AESI** | **Test** | **Computational implementation** | **Nominal P value** | **Exploratory interpretation** |
| --- | --- | --- | --- | --- |
| Hematologic toxicity | Fisher–Freeman–Halton exact test for 3 × 2 table | Complete conditional enumeration with fixed margins; two-sided probability ordering | 0.0954 | No clear overall group difference in the exploratory exact comparison |
| Gastrointestinal toxicity | Fisher–Freeman–Halton exact test for 3 × 2 table | Complete conditional enumeration with fixed margins; two-sided probability ordering | 0.0128 | Smallest unadjusted P value; descriptive overall group difference |
| Hepatobiliary toxicity | Fisher–Freeman–Halton exact test for 3 × 2 table | Complete conditional enumeration with fixed margins; two-sided probability ordering | 0.0723 | No clear overall group difference in the exploratory exact comparison |
| ILD/pneumonitis | Fisher–Freeman–Halton exact test for 3 × 2 table | Complete conditional enumeration with fixed margins; two-sided probability ordering | 0.1742 | No clear overall group difference in the exploratory exact comparison |
| Skin toxicity | Fisher–Freeman–Halton exact test for 3 × 2 table | Complete conditional enumeration with fixed margins; two-sided probability ordering | 0.5311 | No clear overall group difference in the exploratory exact comparison |
| Infection/febrile events | Fisher–Freeman–Halton exact test for 3 × 2 table | Complete conditional enumeration with fixed margins; two-sided probability ordering | 0.4690 | No clear overall group difference in the exploratory exact comparison |
| Peripheral neuropathy | Fisher–Freeman–Halton exact test for 3 × 2 table | Complete conditional enumeration with fixed margins; two-sided probability ordering | 0.3117 | No clear overall group difference in the exploratory exact comparison |
| Cardiac toxicity | Fisher–Freeman–Halton exact test for 3 × 2 table | Complete conditional enumeration with fixed margins; two-sided probability ordering | 1.0000 | No clear overall group difference in the exploratory exact comparison |
| Infusion/hypersensitivity | Fisher–Freeman–Halton exact test for 3 × 2 table | Complete conditional enumeration with fixed margins; two-sided probability ordering | 1.0000 | No clear overall group difference in the exploratory exact comparison |
| Renal abnormality | Fisher–Freeman–Halton exact test for 3 × 2 table | Complete conditional enumeration with fixed margins; two-sided probability ordering | 1.0000 | No clear overall group difference in the exploratory exact comparison |
| Ocular toxicity | Fisher–Freeman–Halton exact test for 3 × 2 table | Complete conditional enumeration with fixed margins; two-sided probability ordering | 1.0000 | No clear overall group difference in the exploratory exact comparison |

**Panel B. Institutional exact-test statistical results (continued).**

Pairwise two-sided Fisher exact tests with Holm adjustment across 12 prespecified comparisons.

| **AESI** | **Pairwise comparison** | **n1** | **N1** | **n2** | **N2** | **Nominal pairwise P value** | **Holm-adjusted P value** | **Exploratory interpretation** |
| --- | --- | --- | --- | --- | --- | --- | --- | --- |
| Hematologic toxicity | T-DM1 vs T-DXd | 12 | 31 | 22 | 50 | 0.8171 | 1.0000 | No robust pairwise difference after Holm adjustment; exploratory only. |
| Hematologic toxicity | T-DM1 vs Sacituzumab govitecan | 12 | 31 | 16 | 24 | 0.0577 | 0.5194 | No robust pairwise difference after Holm adjustment; exploratory only. |
| Hematologic toxicity | T-DXd vs Sacituzumab govitecan | 22 | 50 | 16 | 24 | 0.0850 | 0.6800 | No robust pairwise difference after Holm adjustment; exploratory only. |
| Gastrointestinal toxicity | T-DM1 vs T-DXd | 9 | 31 | 30 | 50 | 0.0113 | 0.1359 | Nominal unadjusted P value was small, but the contrast was not distinguishable after Holm adjustment; exploratory only. |
| Gastrointestinal toxicity | T-DM1 vs Sacituzumab govitecan | 9 | 31 | 15 | 24 | 0.0161 | 0.1776 | Nominal unadjusted P value was small, but the contrast was not distinguishable after Holm adjustment; exploratory only. |
| Gastrointestinal toxicity | T-DXd vs Sacituzumab govitecan | 30 | 50 | 15 | 24 | 1.0000 | 1.0000 | No robust pairwise difference after Holm adjustment; exploratory only. |
| Hepatobiliary toxicity | T-DM1 vs T-DXd | 10 | 31 | 8 | 50 | 0.1045 | 0.7318 | No robust pairwise difference after Holm adjustment; exploratory only. |
| Hepatobiliary toxicity | T-DM1 vs Sacituzumab govitecan | 10 | 31 | 2 | 24 | 0.0485 | 0.4851 | Nominal unadjusted P value was small, but the contrast was not distinguishable after Holm adjustment; exploratory only. |
| Hepatobiliary toxicity | T-DXd vs Sacituzumab govitecan | 8 | 50 | 2 | 24 | 0.4836 | 1.0000 | No robust pairwise difference after Holm adjustment; exploratory only. |
| ILD/pneumonitis | T-DM1 vs T-DXd | 1 | 31 | 6 | 50 | 0.2416 | 1.0000 | No robust pairwise difference after Holm adjustment; exploratory only. |
| ILD/pneumonitis | T-DM1 vs Sacituzumab govitecan | 1 | 31 | 0 | 24 | 1.0000 | 1.0000 | No robust pairwise difference after Holm adjustment; exploratory only. |
| ILD/pneumonitis | T-DXd vs Sacituzumab govitecan | 6 | 50 | 0 | 24 | 0.1682 | 1.0000 | No robust pairwise difference after Holm adjustment; exploratory only. |

**Supplementary Table S8A. Available-layer comparison of FAERS reporting patterns, adjusted reporting contrasts, and institutional observations**

Rows were derived by integrating crude FAERS AESI reporting proportions, domain-specific adjusted reporting-odds estimates, and final institutional patient-level AESI summaries. Cross-layer interpretation was descriptive only and did not involve statistical pooling across FAERS reports and institutional patients. Cross-layer interpretation represents descriptive clinical contextualization only. FAERS reporting proportions and adjusted reporting-odds estimates were not statistically pooled with institutional patient-level observations because the two sources differ in analytical unit, denominator, ascertainment, and data-generating mechanism. Domains with non-distinguishable adjusted FAERS contrasts or sparse institutional event counts were not assigned a comparative drug priority. AESI, adverse event of special interest; FAERS, FDA Adverse Event Reporting System; SG, sacituzumab govitecan; T-DM1, trastuzumab emtansine; T-DXd, trastuzumab deruxtecan.

| **AESI domain** | **FAERS crude reporting pattern** | **Adjusted FAERS interpretation** | **Institutional observation** | **Institutional event count (n)** | **Cross-layer contextual interpretation** |
| --- | --- | --- | --- | --- | --- |
| Hematologic toxicity | Sacituzumab govitecan showed the highest crude reporting proportion among the three core ADCs. | Sacituzumab govitecan demonstrated higher adjusted reporting odds than T-DM1, whereas T-DXd demonstrated lower adjusted reporting odds than T-DM1. | Hematologic toxicity occurred in 16/24 (66.7%) sacituzumab govitecan recipients, 22/50 (44.0%) T-DXd recipients, and 12/31 (38.7%) T-DM1 recipients. Seven patients had Grade ≥3 events and two were hospitalized. | 50 | Directionally concordant higher hematologic reporting was observed for sacituzumab govitecan across FAERS and institutional data. |
| Gastrointestinal toxicity | Both sacituzumab govitecan and T-DXd showed higher crude reporting proportions than T-DM1. | Both sacituzumab govitecan and T-DXd showed higher adjusted reporting odds than T-DM1; the available analyses did not establish separation between these two agents. | Gastrointestinal toxicity occurred in 15/24 (62.5%) sacituzumab govitecan recipients, 30/50 (60.0%) T-DXd recipients, and 9/31 (29.0%) T-DM1 recipients. Ten patients had Grade ≥3 events and five were hospitalized. | 54 | High gastrointestinal burden was observed for sacituzumab govitecan and T-DXd across available layers; institutional data were insufficient for comparative ranking. |
| Hepatobiliary toxicity | T-DM1 showed the highest crude reporting proportion. | T-DM1 demonstrated higher adjusted reporting odds than both T-DXd and sacituzumab govitecan. | Hepatobiliary toxicity occurred in 10/31 (32.3%) T-DM1 recipients, 8/50 (16.0%) T-DXd recipients, and 2/24 (8.3%) sacituzumab govitecan recipients. One Grade ≥3 event occurred and no hospitalization was documented. | 20 | Directionally concordant T-DM1-associated reporting pattern across FAERS and institutional observations. |
| ILD/pneumonitis | T-DXd showed the highest crude reporting proportion. | T-DXd demonstrated higher adjusted reporting odds than T-DM1, whereas sacituzumab govitecan demonstrated lower adjusted reporting odds than T-DM1. | ILD/pneumonitis occurred in 6/50 (12.0%) T-DXd recipients, 1/31 (3.2%) T-DM1 recipient, and 0/24 sacituzumab govitecan recipients. Source-record re-audit confirmed three Grade 3 events, all in the T-DXd group, with no Grade 4 or Grade 5 events; two patients were hospitalized. | 7 | Directionally concordant T-DXd-associated pattern across FAERS and institutional observations, although institutional event numbers were limited. |
| Skin toxicity | Sacituzumab govitecan showed the highest crude reporting proportion. | Both T-DXd and sacituzumab govitecan demonstrated higher adjusted reporting odds than T-DM1. | Skin toxicity occurred in 4/24 (16.7%) sacituzumab govitecan recipients, 6/50 (12.0%) T-DXd recipients, and 2/31 (6.5%) T-DM1 recipients. One Grade ≥3 event occurred and no hospitalization was documented. | 12 | The frequency pattern was directionally similar between FAERS and institutional observations, but severity events were uncommon. |
| Infection/febrile events | Crude reporting proportions differed modestly among agents. | No statistically distinguishable adjusted reporting pattern was identified. | Infection/febrile events occurred in 4/24 (16.7%) sacituzumab govitecan recipients, 4/50 (8.0%) T-DXd recipients, and 2/31 (6.5%) T-DM1 recipients. All ten events were Grade ≥3 and five were followed by hospitalization. | 10 | No consistent cross-layer pattern was identified; findings remain descriptive and hypothesis-generating. |
| Peripheral neuropathy | T-DM1 showed the highest crude reporting proportion. | T-DM1 demonstrated higher adjusted reporting odds than T-DXd and sacituzumab govitecan. | Peripheral neuropathy occurred in 5/31 (16.1%) T-DM1 recipients, 3/50 (6.0%) T-DXd recipients, and 1/24 (4.2%) sacituzumab govitecan recipient. One Grade ≥3 event occurred and no hospitalization was documented. | 9 | Directionally concordant T-DM1-associated pattern across available layers, although institutional event numbers were limited. |
| Cardiac toxicity | T-DM1 showed the highest crude reporting proportion. | Sacituzumab govitecan showed lower adjusted reporting odds than T-DM1; other contrasts were not clearly distinguishable. | Cardiac events occurred in 1/31 (3.2%) T-DM1 recipient, 2/50 (4.0%) T-DXd recipients, and 0/24 sacituzumab govitecan recipients. One Grade ≥3 event occurred and no hospitalization was documented. | 3 | No clear cross-layer pattern could be assigned because institutional events were rare. |
| Infusion/hypersensitivity | Reporting proportions were low across all agents. | T-DXd showed lower adjusted reporting odds than T-DM1; remaining contrasts were not clearly distinguishable. | Four institutional events occurred (T-DM1: 1/31; T-DXd: 2/50; sacituzumab govitecan: 1/24). No Grade ≥3 events, dose modifications, or hospitalizations occurred. | 4 | Evidence was insufficient for stable cross-layer interpretation because of limited event counts. |
| Renal abnormality | Crude reporting differences were small among agents. | No statistically distinguishable adjusted reporting pattern was identified. | Four institutional events occurred (T-DM1: 1/31; T-DXd: 2/50; sacituzumab govitecan: 1/24). One sacituzumab govitecan event was Grade ≥3 and was followed by hospitalization and permanent discontinuation. | 4 | No consistent cross-layer pattern was identified; individual cases require clinical interpretation rather than comparative inference. |
| Ocular toxicity | T-DM1 showed the highest crude reporting proportion. | T-DM1 demonstrated higher adjusted reporting odds than T-DXd and sacituzumab govitecan. | Source-record re-audit identified two ocular AESIs: one Grade 1 dry-eye event in a T-DXd recipient and one Grade 1 conjunctivitis/dry-eye event in a T-DM1 recipient; no ocular event occurred in the sacituzumab govitecan group. Neither event required dose modification or hospitalization. | 2 | Institutional ocular events were too sparse to support stable cross-layer interpretation despite the FAERS reporting contrast. |

**Supplementary Table S8B. Directional cross-layer contextualization of prespecified AESI domains in FAERS and the institutional cohort**

Cross-layer direction was assessed descriptively without statistical pooling. Directionally aligned indicates a broadly similar drug-specific pattern across adjusted FAERS and institutional observations; No clear alignment indicates differing or non-distinguishable patterns; Not assessable indicates no clear adjusted FAERS priority or insufficient institutional events for stable interpretation. These categories do not represent statistical concordance, external validation, incidence confirmation, or comparative safety. AESI, adverse event of special interest; FAERS, FDA Adverse Event Reporting System; SG, sacituzumab govitecan; T-DM1, trastuzumab emtansine; T-DXd, trastuzumab deruxtecan.

| **AESI domain** | **Crude FAERS pattern** | **Adjusted FAERS interpretation** | **Institutional pattern** | **Cross-layer direction** |
| --- | --- | --- | --- | --- |
| Hematologic toxicity | SG | SG | SG | Directionally aligned |
| Gastrointestinal toxicity | T-DXd/SG | T-DXd/SG | T-DXd/SG | Directionally aligned |
| Hepatobiliary toxicity | T-DM1 | T-DM1 | T-DM1 | Directionally aligned |
| ILD/pneumonitis | T-DXd | T-DXd | T-DXd | Directionally aligned |
| Skin toxicity | SG | T-DXd/SG | SG | Directionally aligned |
| Infection/febrile events | No clear crude priority | No priority | SG | No clear alignment |
| Peripheral neuropathy | T-DM1 | T-DM1 | T-DM1 | Directionally aligned |
| Cardiac toxicity | T-DM1 | No priority | Insufficient data | Not assessable |
| Infusion/hypersensitivity | No clear crude priority | No priority | Insufficient data | Not assessable |
| Renal abnormality | No clear crude priority | No priority | Insufficient data | Not assessable |
| Ocular toxicity | T-DM1 | T-DM1 | Insufficient data | Not assessable |
